# Fluoride‐Transfer Asymmetric Allylic Alkylation Enables Regiodivergent, Stereoselective Cross‐Electrophile Coupling

**DOI:** 10.1002/anie.202520513

**Published:** 2025-11-22

**Authors:** Jordi Duran, Vyali Georgian Moldoveanu, Claudia Barroso, Geraldo Augusto Pereira, Bart Limburg, Xavier Companyó

**Affiliations:** ^1^ Section of Organic Chemistry Department of Inorganic and Organic Chemistry University of Barcelona Carrer Martí i Franquès 1 Barcelona 08028 Spain; ^2^ Institut de Química Teòrica i Computacional (IQTC) Carrer Martí i Franquès 1 Barcelona 08028 Spain

**Keywords:** Asymmetric allylic alkylation, Asymmetric trifluoromethylation, Atom economy, Cross‐electrophile coupling, Regiodivergent catalysis

## Abstract

Asymmetric allylic alkylation (AAA) is a widely used strategy for stereoselective formation of C─C bonds. Yet, conventional methods often exhibit poor atom economy as the leaving group is generally wasted. Herein, we report a fluoride‐transfer asymmetric allylic alkylation (AAA) in which all the atoms of the reagents are incorporated into the final products. The catalytic platform enables regiodivergent, stereoselective cross‐electrophile coupling of allyl fluorides and *gem*‐difluoroalkenes, where the regiochemical outcome is directed by the selective catalytic activation of one or the other electrophilic partner. The resulting atom‐efficient AAA protocol provides a regiocontrollable access to homoallylic trifluoromethylated compounds from a common set of electrophilic starting materials.

## Graphical abstract

We report a fluoride‐transfer asymmetric allylic alkylation (AAA) in which all reagent atoms, including the leaving group, are incorporated into the products. This atom‐efficient AAA protocol enables the cross‐electrophile coupling of allyl fluorides and *gem*‐difluoroalkenes, providing a catalyst‐controlled, regiodivergent, and stereoselective access to trifluoromethylated homoallylic scaffolds.

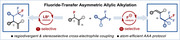

Asymmetric allylic alkylations (AAA) are amongst the most powerful catalytic strategies for constructing C─C bonds in stereoselective manner.^[^ ^1^, ^2^, ^3^, ^4^ ^]^ Conventional AAAs, catalyzed by transition‐metal complexes^[^ ^5^, ^6^, ^7^, ^8^, ^9^ ^]^ or chiral Lewis bases,^[^ ^10^, ^11^, ^12^, ^13^ ^]^ usually employ allylic electrophiles bearing carbonates or acetates as the leaving group (LG). Upon catalytic activation, the ionized leaving group deprotonates the pronucleophile, thereby becoming a by‐product of the reaction (LG─H, Figure 1a, top). In contrast, the direct use of allylic alcohols in transition‐metal catalyzed AAA offers a more efficient approach, since water is the only stoichiometric by‐product.^[^ ^14^, ^15^, ^16^ ^]^ More recently, allyl fluorides have emerged as alternative electrophiles in AAA.^[^ ^17^ ^]^ Defluorinative AAAs, first introduced by Shibata,^[^ ^18^ ^]^ employ silylated pronucleophiles that are activated via fluoride‐assisted desilylation.^[^ ^18^, ^19^, ^20^, ^21^, ^22^, ^23^, ^24^, ^25^, ^26^, ^27^, ^28^, ^29^, ^30^, ^31^ ^]^ The formation of fluorosilanes (BDE (Si─F)∼160 kcal mol^−1^) provides the thermodynamic driving force for the cleavage of the C─F bond (BDE (allyl Csp^3^─F) = 94–102 kcal mol^−1^),^[^ ^32^, ^33^, ^34^, ^35^ ^]^ and as a result, the fluorine atom is also wasted (Figure 1a, bottom). Therefore, AAA methods generally exhibit suboptimal atom economy.

**Figure 1:**
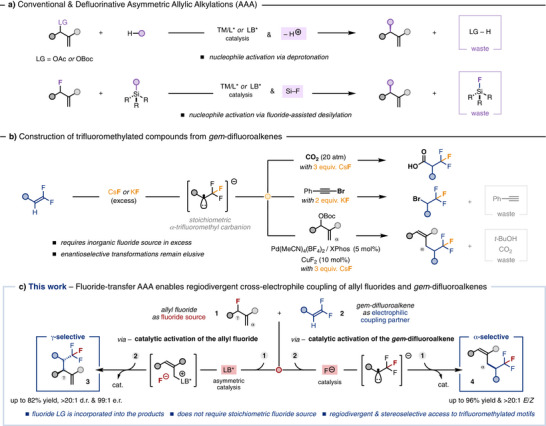
a) Common AAAs discard the leaving group. b) Racemic formation of trifluoromethylated compounds from *gem*‐difluoroalkenes. c) Fluoride‐transfer AAA enables regiodivergent and stereoselective XEC via selective catalytic activation of either electrophilic partner.

Trifluoromethylated compounds are widely used in pharmaceuticals, agrochemicals, and material science.^[^ ^36^, ^37^, ^38^, ^39^, ^40^ ^]^ The incorporation of CF_3_ groups into biologically active compounds enhances key molecular properties, such as acidity, polarity, permeability, and metabolic stability.^[^ ^36^, ^37^, ^38^, ^39^, ^40^ ^]^ Given their pivotal role and virtual absence in natural products,^[^ ^41^ ^]^ the development of novel strategies for the stereocontrolled formation of trifluoromethylated compounds is of paramount importance in contemporary chemistry.^[^ ^42^, ^43^, ^44^, ^45^, ^46^, ^47^ ^]^ In this context, *gem*‐difluoroalkenes are versatile building blocks for constructing fluorinated compounds.^[^ ^48^, ^49^ ^]^ The fluorine‐induced electronic perturbation of the alkene moiety enables distinctive reactivity profiles via radical, electrophilic, or nucleophilic pathways.^[^ ^50^, ^51^, ^52^, ^53^, ^54^ ^]^ Nucleophilic addition of fluoride anion to *gem*‐difluoroalkenes –typically alkali fluoride salts in excess– generates an α‐trifluoromethyl carbanion that can subsequently react with specific electrophiles (Figure 1b).^[^ ^50^, ^51^, ^52^, ^53^, ^54^ ^]^ For example, reaction with carbon dioxide produces α‐trifluoromethyl carboxylic acids,^[^ ^55^ ^]^ while reaction with a bromoalkyne yields α‐trifluoromethyl bromides,^[^ ^56^ ^]^ both in racemic form (Figure 1b). In this context, Loh and Feng reported the α‐selective alkylation of allyl carbonates with *gem*‐difluoroalkenes catalyzed by a cationic palladium complex (Figure 1b).^[^ ^57^ ^]^ The protocol employs 3 equiv. of CsF and 10 mol% of CuF_2_, affording a wide range of homoallylic trifluoromethylated compounds in excellent yields (Figure 1b). Yet, attempts to develop an enantioselective variant were unsuccessful: despite testing over 40 chiral ligands, the enantioselectivity could not be improved beyond 56:44 e.r.^[^ ^57^ ^]^

Herein, we report a defluorinative asymmetric allylic alkylation (AAA) in which the ionized fluoride leaving group is reincorporated into the products upon transient formation of an α‐trifluoromethyl carbanion. The fluoride‐transfer AAA strategy enables the regiodivergent^[^ ^58^, ^59^, ^60^, ^61^, ^62^, ^63^, ^64^ ^]^ cross‐electrophile coupling (XEC) of allyl fluorides **1** and *gem*‐difluoroalkenes **2** to construct homoallylic trifluoromethylated compounds in a stereoselective manner (**3** and **4**, Figure 1c). The formation of the trifluoromethyl motif (BDE (C─F_3_)∼120 kcal mol^−1^) provides the thermodynamic driving force for C─F bond cleavage.^[^ ^32^, ^33^, ^34^, ^35^ ^]^ As a result, the present catalytic platform establishes an atom‐efficient alternative to the conventional use of silylated pronucleophiles in defluorinative AAA. The key conceptual advance of this work lies in the selective catalytic activation of either electrophilic partner, which dictates the regiochemical outcome. Chiral Lewis‐base activation of the allyl fluoride **1** promotes γ‐selective XEC, affording trifluoromethylated homoallylic compounds **3** with two adjacent stereocentres (up to 82% yield, >20:1 d.r., 99:1 e.r., Figure 1c, left). Conversely, catalytic *n*‐tetrabutylammonium fluoride (TBAF) activates the *gem*‐difluoroalkene **2**, steering reactivity toward α‐selective XEC to yield racemic trifluoromethylated trisubstituted olefins **4** (up to 96% yield, >20:1 *E/Z*, Figure 1c, right).

Initially, we hypothesized that the fluoride anion, released upon Lewis‐base catalytic activation of the allyl fluoride **1**, could act as a competent nucleophile able to add to *gem*‐difluoroalkene **2**. The ensuing α‐trifluoromethyl carbanion could then undergo regioselective addition to the chiral ammonium intermediate, yielding the γ‐alkylated product **3** through an overall S_N_2′–S_N_2′ mechanism (Figure 1c, left). To test this hypothesis, we screened several chiral Lewis‐base catalysts in the reaction between *rac*‐**1a** and **2a** in THF (Table 1a). Although **5a** and **5b** did not exhibit catalytic activity (entries 1–2), **5c** afforded **3a** in 12% yield with excellent regio‐ and stereoselectivity (>20:1 **3a**/**4a**, 9:1 d.r., 94:6 e.r., entry 3). Other Lewis‐base catalysts produced **3a** with improved yields but lower selectivity (entries 4–6). Increasing the polarity of the solvent shifted the regioselectivity toward formation of **4a** (entries 7–8), whereas aromatic hydrocarbons restored γ‐alkylation (entries 9–10). By using 3.0 equiv. of **2a** in a 0.2 M solution of PhCF_3_, product **3a** was isolated in 72% yield, 8:1 d.r. and 96:4 e.r. after 72 h (entry 11). Under the optimized conditions, the reaction can be performed with equimolar amounts of **1a** and **2a** at the expenses of longer reaction time, affording **3a** in 63% yield after 168 h.^[^ ^65^ ^]^

**Table 1:** Design and optimization of the regiodivergent and stereoselective XEC of *rac*‐**1a** and **2a** via fluoride‐transfer AAA.

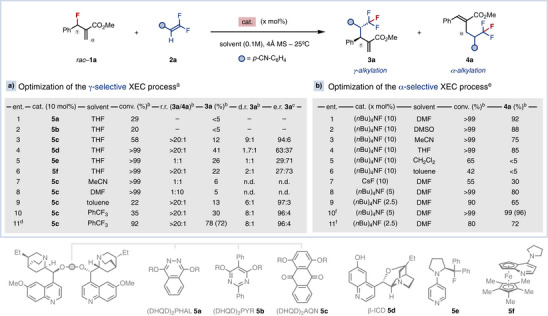

^a)^ **1a** (0.1 mmol), **2a** (0.2 mmol), cat. (10 mol%), 4 Å MS, 18 h. ^b)^Determined by ^1^H‐NMR (isolated yield).

^c)^Determined by chiral HPLC.

^d)^ **2a** (0.3 mmol) at 0.2 M, 72 h.

^e)^ **1a** (0.1 mmol), **2a** (0.1 mmol), cat. (2.5–10 mol%), 4 Å MS, 30 min. ^f)^ **2a** (0.15 mmol).

Next, we reasoned that the direct activation of the *gem*‐difluoroalkene **2** in the absence of a Lewis‐base catalyst could favor α‐selective XEC. Specifically, catalytic fluoride could transiently generate the α‐trifluoromethyl carbanion, which would then selectively attack the α‐position of the **1**. This S_N_2′ addition would form **4** and release another catalytic equivalent of fluoride to promote a subsequent α‐selective XEC (Figure 1c, right). The treatment of an equimolar mixture of *rac*‐**1a** and **2a** with 10 mol% of TBAF in DMF produced **4a** in 92% yield and excellent *E–*selectivity (Table 1b, entry 1). Other polar solvents were also suitable for this transformation (entries 2–4), while apolar solvents completely prevented reactivity (entry 5–6). Alternative fluoride sources, such as CsF, were less efficient than TBAF (entry 7).^[^ ^65^ ^]^ Finally, we evaluated the minimum loading of TBAF required to trigger the α‐selective XEC (entries 8–11). Using 5 mol% of TBAF and 1.5 equiv. of **2a**, (*E*)‐**4a** was isolated with 96% yield (entry 10).

With the regiodivergent catalytic platform in hand, we examined the generality of the γ‐selective XEC (Figure 2). The aromatic ring of **1** tolerates a variety of halogenated substituents in *para*, affording the homoallylic trifluoromethylated compounds **3b**‐**d**,**i** in good yields (68%–75%), good diastereoselectivities (8.5:1 d.r.), excellent enantioselectivities (95:5–98:2 e.r.) and complete regiocontrol. Other electron‐withdrawing groups at **1**, such as nitrile (**3e**) and ester (**3f**), are also well tolerated. Electron‐rich aryl‐substituted **1** are also competent coupling partners. The *p*‐Me (**3g**), *p*‐^t^Bu (**3h**), and *m*‐Me (**3j**) were isolated in 56%–67% yield and 5:1 d.r., while the *o*‐Me substituted product **3k** was formed in 40% yield and enhanced 10:1 d.r, maintaining excellent enantiocontrol in all cases (94:6–98:2 e.r.). Alkyl‐derived allyl fluorides **1** proved unreactive under optimized asymmetric conditions.^[^ ^65^ ^]^ Regarding the *gem*‐difluoroalkenes **2**, homoallylic trifluoromethylated products bearing electron‐withdrawing groups in the *para* position, such as ester (**3n**), acetyl (**3o**), benzoyl (**3p**), sulfonic ester (**3r**), sulfonamide (**3s**), and nitro (**3t**), were obtained in good yields (48%–75%), good diastereoselectivity (4:1–10:1 d.r.) and high enantiocontrol (92:8–99:1, Figure 2). *Ortho*‐substituted *gem*‐difluoroalkenes led to a marked increase in the diastereocontrol, providing **3u** and **3v** as a single diastereoisomer (>20:1 d.r.) and excellent enantioselectivities (95:5–96:4 e.r.). Given the relevance of the pyridine motifs,^[^ ^66^, ^67^ ^]^ we extended the γ‐selective asymmetric XEC to pyridine‐containing substrates. Both **3w** and **3x** were isolated in good yields and high enantiocontrol, albeit in diverse diastereoselectivity: while the 2‐pyridyl derivative **3w** was formed without diastereocontrol, the 3‐pyridyl analogue **3x** exhibited a 9:1 d.r. Finally, *meta*‐substituted **2** delivered only trace amounts of the corresponding products. Nevertheless, products **3y** and **3z** could be obtained as racemates with 66% and 92% yield using DABCO in THF.^[^ ^65^ ^]^ Overall, while the electronic nature of the aromatic ring of allyl fluorides **1** does not greatly affect the γ‐selective XEC, the substituents on the aryl ring of *gem*‐difluoroalkenes **2** significantly influence the catalytic process, as follows: i) electron‐withdrawing substituents in the *para* and *ortho* positions, capable of stabilizing the α‐trifluoromethyl carbanion by resonance, enable smooth asymmetric XEC; ii) electron‐withdrawing substituents unable to provide resonance stabilization, such as purely inductive groups or substituents in the *meta* position, react only under racemic catalytic conditions; iii) electron‐neutral aryl rings or alkyl‐substituted **2** are reluctant to undergo γ‐selective XEC.^[^ ^65^ ^]^ The absolute configuration of the brominated product **3i** was unambiguously assigned as (*3R,4R*) based on the X‐ray diffraction of the diol derivative **6**.^[^ ^65^, ^68^ ^]^

**Figure 2:**
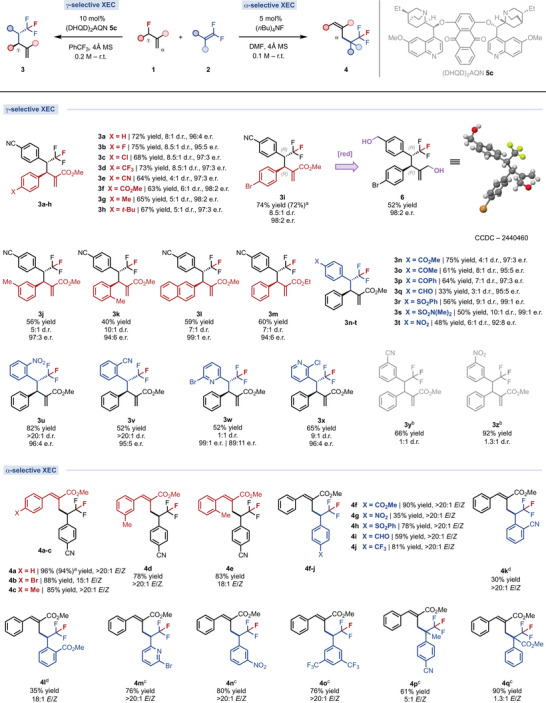
Generality of the fluoride‐transfer AAA platform. γ‐selective XEC: **1** (0.2 mmol), **2** (0.6 mmol), **5c** (10 mol%), 4 Å MS. α‐selective XEC: **1** (0.20 mmol), **2** (0.30 mmol), TBAF (5 mol%), 4 Å MS. ^a^1 mmol scale. ^b^DABCO **5g** (10 mol%) as catalyst in THF. ^c^10 mol% and ^d^20 mol% of TBAF.

We next evaluated the scope of the α‐selective XEC (Figure 2). Allyl fluorides **1** bearing diverse aryl substituents were well tolerated, affording the racemic trifluoromethylated olefins **4b**‐**e** in excellent yields (78%–96% yield) and *E/Z* selectivities (15:1‐>20:1 d.r). Then, we investigated the influence of aryl substitution on **2**. *Para*‐substituted electron‐withdrawing groups provided products **4f**‐**4i** in moderate to excellent yields (35%–90% yield) and complete *E*‐selectivity. Notably, the *p*‐CF_3_‐substituted alkene, which was unreactive in the γ‐selective XEC,^[^ ^65^ ^]^ produced **4j** in 81% yield and >20:1 *E*/*Z*. *Ortho*‐substituted **2** react slowly and require a higher catalytic loading of fluoride to afford products **4k** and **4l** in 30%–35% yield. In contrast, the 2‐pyridyl (**4m**) and the *meta*‐substituted (**4n**, **4o**) products were formed in 76%–80% yields under the effect of 10 mol% of TBAF. Finally, β,β‐disubstituted *gem*‐difluoroalkenes are compatible substrates, providing access to racemic trisubstituted olefins with a trifluoromethylated quaternary stereocenter. Products **4p** and **4q** were obtained in 61%–90% yields and reduced *E‐*selectivity. Both γ‐ and α‐selective XEC protocols are readily scaled up to 1 mmol without compromising either yield or selectivity (**3i** and **4a**, Figure 2).

To gain a fundamental understanding on the catalytic mechanisms and the factors controlling the regiodivergent selectivity, we conducted in situ spectroscopic studies, kinetic analyses, and density functional theory (DFT) calculations (Figure 3). The free energy profiles for the reaction between **1a** and **2a** were computed in the presence of either DABCO (**5g**) or fluoride as catalyst, in toluene and DMF (Figure 3a).^[^ ^69^ ^]^ First, we examined the equilibria involved in the catalytic activation of the two electrophilic coupling partners. Formation of **8**‐F, via S_N_2′ addition of catalyst **5g** to **1a**, is solvent‐dependent, being exergonic by −3.3 kcal mol^−1^ in DMF and endergonic by +9.0 kcal mol^−1^ in toluene (Figure 3ai). These values are consistent with the experimental formation of **8**‐F in the presence of 10 mol% of **5g**, occurring almost quantitatively in DMF or reaching an unfavored equilibrium in toluene (Figure 3bi, entries 1–2). In contrast, no formation of **8**‐F was detected with the chiral catalyst **5c** in PhCF_3_ (Figure 3bi, entry 3), indicating a more disfavored equilibrium. The equilibrium position of **8**‐F is a fundamental factor in the γ‐selective XEC, which determines the effective concentration of fluoride in solution. The formation of the α‐trifluoromethyl carbanion **9^−^** via nucleophilic addition of fluoride to **2a** is endergonic in all the solvents, yet less so in toluene (+3.8 kcal mol^−1^) than in DMF (+10.0 kcal mol^−1^). Therefore, although the formation of **8**‐F is less favored in toluene, resulting in a lower effective concentration of fluoride, the easier formation of carbanion **9^−^** leads to an overall feasible γ‐XEC process.

**Figure 3:**
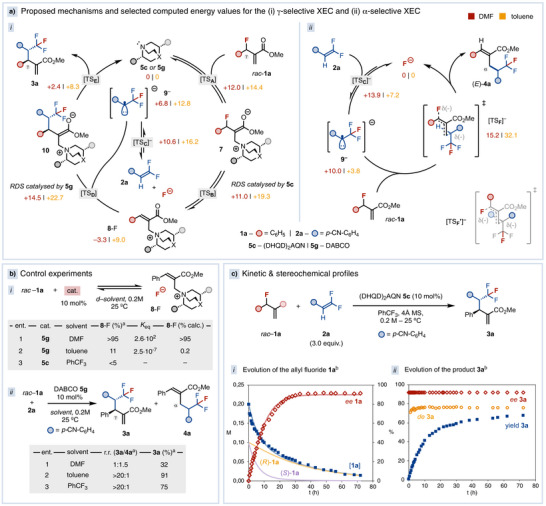
a) Proposed mechanisms and selected computed energies with **5g** as catalyst (kcal mol^−1^). b) i) Experimental and calculated formation of **8**‐F; ii) Regioselectivity of the XEC catalyzed by **5g**. c) Kinetic and stereochemical profiles of the asymmetric γ‐selective XEC. ^a^Determined by ^1^H‐NMR. ^b^Determined by GC and HPLC.

Considering the global energy profiles, C─C bond‐formation is rate‐determining in both the γ‐selective (TS_D_) and the α‐selective (TS_F_^−^) pathways (Figure 3a). Therefore, the equilibria leading to **8**‐F and **9^−^** are well‐established before the C─C bond‐formation step, whereas the thermodynamic driving force is only released upon product formation. The computed energy barriers align with the experimentally observed solvent‐dependent regioselectivity (Figure 3bii). In DMF, the energy difference between TS_D_ and TS_F_^−^ is 0.7 kcal mol^−1^, consistent with the poor regioselectivity observed (**3a**/**4a **= 1:3.7 calc. versus 1:1.5 exp.).^[^ ^65^ ^]^ In contrast, in toluene, TS_D_ is favored by 9.4 kcal mol^−1^, coherent with the excellent γ‐selectivity.^[^ ^65^ ^]^ For the less activated *gem*‐difluoroalkenes **2**, which likely exhibit a more unfavorable equilibrium between **2** to **9^−^**, a good compromise was found by using the more nucleophilic catalyst **5g** in THF. These conditions increase the concentration of F^−^ and lowers the barrier for TS_C_^−^,^[^ ^65^ ^]^ enabling the formation of products **3y** and **3z** (Figure 2).

We propose that α‐selective XEC proceeds via a concerted S_N_2′ C─C bond‐forming event, as no minimum‐energy intermediate could be located beyond TS_F_^−^ (Figure 3aii). In the optimized TS_F_^−^, the hydrogen lies proximal to the aryl group, resulting in a *trans* arrangement of the aryl and ester, which lead to the formation of (*E*)–**4a**. The barrier for TS_F_^−^ is +15.2 kcal mol^−1^ in DMF and +32.1 kcal mol^−1^ in toluene. These values are consistent with the fact that the reaction proceeds in polar solvents but is suppressed in apolar media (Table 1b). To explain the reduced *E/Z*‐selectivity with β‐disubstituted *gem*‐difluoroalkenes (**4p**‐**q**, Figure 2), we hypothesize that the β‐substituents would sterically clash with the aryl group in TS_F_^−^, thereby increasing its energy. In this scenario, the alternative TS_F_′^−^, where the allylic bond in **1** is rotated, may compete with TS_F_^−^. This rotation positions the β‐substituent toward the smaller fluorine atom, minimizing steric interaction, and leading to the formation of (*Z*)‐**4** (Figure 3aii).

Finally, we measured the kinetic and stereochemical profiles of the asymmetric γ‐selective XEC catalyzed by **5c** (Figure 3c). At the onset, *rac*‐**1a** is consumed with preferential depletion of the (*S*)‐enantiomer, eventually reaching a steady composition of 95:5 (*R/S*). Product **3a** is consistently formed with 96:4 e.r. and 8:1 d.r., indicating that the stereodetermining step involves a stereoconvergent, catalyst‐controlled C─C bond formation (Figure 3cii). Kinetic fitting supports a model^[^ ^65^ ^]^ in which product **3a** formation and the reversion of **8**‐F to **1a** occurs at comparable rates, with the rate‐limiting step being the formation of **8**‐F. These assumptions are consistent with the experimental observations showing that the formation of **8**‐F with chiral catalyst **5c** is less favored, likely bringing TS_B_ closer in energy to TS_D_. In this scenario, the enantioenrichment of **1a** up to 95:5 e.r. arises from the initially faster consumption of (*S*)‐**1a**, followed by partial racemization at later stages via reformation of *rac*‐**1a** from **8**‐F. Overall, the kinetic model is in agreement with the DFT results despite using different catalysts.

In summary, we have developed a fluoride‐transfer AAA that enables the regiodivergent, stereoselective cross‐electrophile coupling of allyl fluorides and *gem*‐difluoroalkenes to form trifluoromethylated homoallylic scaffolds. The catalytic platform offers significant advancements over current methods by: i) incorporating the fluoride leaving group into the products instead of discarding it;^[^ ^17^, ^18^, ^19^, ^20^, ^21^, ^22^, ^23^, ^24^, ^25^, ^26^, ^27^, ^28^, ^29^, ^30^, ^31^ ^]^ ii) leveraging allyl fluorides as an internal source of nucleophilic fluoride, thereby eliminating the need for stoichiometric fluoride salts; ^[^ ^50^, ^51^, ^52^, ^53^, ^54^, ^57^ ^]^ and iii) enabling the elusive catalytic enantioselective functionalization of *gem*‐difluoroalkenes.^[^ ^50^, ^51^, ^52^, ^53^, ^54^, ^57^ ^]^ Mechanistic studies reveal that the catalyst and the solvent both play a key role in controlling reactivity and selectivity. By achieving complete incorporation of all reagent atoms into the products, this atom‐efficient AAA overcomes a longstanding limitation of AAAs and expand the toolbox for the catalytic asymmetric synthesis of fluorinated building blocks.

## Supporting Information

Experimental procedures, analytical data, mechanistic investigations, computational details, NMR spectra and HPLC traces. The authors have cited additional references within the Supporting Information.^[^ ^70^, ^71^, ^72^, ^73^, ^74^, ^75^, ^76^, ^77^, ^78^, ^79^, ^80^, ^81^, ^82^, ^83^, ^84^, ^85^, ^86^, ^87^, ^88^, ^89^, ^90^, ^91^, ^92^, ^93^, ^94^, ^95^, ^96^, ^97^, ^98^, ^99^, ^100^, ^101^, ^102^, ^103^, ^104^, ^105^, ^106^, ^107^, ^108^ ^]^

## Author Contributions

J.D. optimized the methodology with the assistance of C.B. J.D., with the help of V.G.M. and G.A.P., studied the scope. J.D. and B.L. performed computational and mechanistic studies. X.C. conceived and directed the project, and wrote the manuscript with inputs of B.L. and J.D. All authors contributed to the manuscript and approved the final version.

## Conflict of Interests

The authors declare no conflict of interest.

## Supporting information



Supporting Information

Supporting Information

## Data Availability

The data that support the findings of this study are available in the supplementary material of this article.
