# Supplementary material for: Fluoride‐Transfer Asymmetric Allylic Alkylation Enables Regiodivergent, Stereoselective Cross‐Electrophile Coupling
Source: Angew Chem Int Ed Engl. 2025 Nov 22;65(2):e20513. doi: 10.1002/anie.202520513 (PMC12790306; doi:10.1002/anie.202520513)
Supplement: Supplementary file 1 — Supporting Information [file ANIE-65-e20513-s002.pdf]

Supporting Information

Fluoride-Transfer Asymmetric Allylic Alkylation Enables Regiodivergent, Stereoselective Cross-Electrophile Coupling

Jordi Duran,[a] Vyali Georgian Moldoveanu,[a] Claudia Barroso,[a] Geraldo Augusto Pereira,[a] Bart Limburg[a, b] and Xavier Companyó[a, *]

[a]*Section of Organic Chemistry, Department of Inorganic and Organic Chemistry, University of Barcelona. Carrer Martí i Franquès 1, 08028 Barcelona, Spain*

[b]*Institut de Química Teòrica i Computacional (IQTC), Carrer Martí i Franquès 1, 08028 Barcelona, Spain.*

E-mail: x.companyo@ub.edu

Table of Contents

A. General Information **4**

B. Synthesis of Starting Materials **5**

B.1. General Procedure A – Synthesis of Morita-Baylis-Hillman alcohols S3 .... *5*

B.2. General Procedure B – Synthesis of allyl fluorides 1 .... *6*

B.3. General Procedure C – Synthesis of gem-difluoroalkenes 2 .... *7*

C. γ-Selective Racemic Cross-Electrophile Catalytic Coupling between allyl fluorides (1) and gem-difluoroalkenes (2) **9**

D. Optimisation of the γ-Selective Asymmetric Cross-Electrophile Catalytic Coupling between allyl fluorides (1) and gem-difluoroalkenes (2) **13**

D.1. Optimisation – Catalyst screening *13*

D.2. Optimisation – Solvent screening *17*

D.3. Optimisation – Temperature, ratio of reagents, concentration and catalyst loading *18*

D.4. Role and fate of the gem-difluoroalkene in excess *19*

E. γ-Selective Asymmetric Cross-Electrophile Catalytic Coupling between allyl fluorides (1) and gem-difluoroalkenes (2) **22**

F. Optimisation of the α-Selective and diastereoselective Cross-Electrophile Catalytic Coupling between allyl fluorides (1) and *gem*-difluoroalkenes (2) **33**

F.1. Optimisation – Solvent screening *33*

F.2. Optimisation – Screening of the fluoride source *33*

F.3. Optimisation – Ratio of reagents and catalyst loading *34*

F.4. Optimisation – Attempt to develop the α-selective asymmetric cross-electrophile catalytic coupling between allyl fluorides (1) and gem-difluoroalkenes (2) *35*

G. α-Selective and diastereoselective Cross-Electrophile Catalytic Coupling between allyl fluorides (1) and gem-difluoroalkenes (2) **38**

H. Unsuccessful substrates **48**

H.1. Aliphatic allyl fluorides *48*

H.2. Gem-difluoroalkenes *51*

I. Mechanistic Studies **53**

I.1. Control experiments – Generation of the catalytic species with DABCO *53*

I.2. Control experiments – Generation of the catalytic species with $(DHQD)_2AQN$ *59*

I.3. Control experiments – Influence of the solvent in the regioselectivity (γ vs. α) cross-electrophile catalytic coupling between allyl fluorides (1) and gem-difluoroalkenes (2) *61*

I.4. Control experiments – Influence of the aromatic spacer of the dimeric chiral Lewis-base catalyst in the asymmetric cross-electrophile coupling under optimised conditions. *62*

I.5. Control experiments – Study of the potential erosion of the diastereoselectivity of the homoallylic trifluoromethylated product 3a by the catalyst 5c. *63*

I.6. Control experiments – Study of the potential erosion of the diastereoselectivity of the homoallylic trifluoromethylated product 3a by fluoride. *64*

I.7. Kinetic and stereochemical profiles – Evolution of the allyl fluoride 1a *65*

I.8. Kinetic and stereochemical profiles – Evolution of the homoallylic trifluoromethylated product 3a *66*

I.9. Kinetic model – Fitting the experimental data with a kinetic model *67*

I.10. Density Functional Theory *69*

J. Derivatizations of product 3i 73

J.1. Reduction of the ester and nitrile groups – formation of the diol 6 ...... *73*

J.2. 1,3-dipolar cycloaddition – Isoxazoline *75*

K. References 77

L. Determination of the absolute configuration. X-ray crystallographic data of diol 6 79

M. NMR Spectra 88

M.1. NMR spectra of gem-difluoroalkenes 2 ...... *88*

M.2. NMR spectra of γ-alkylation products 3 ...... *96*

M.3. NMR spectra of α-alkylation products 4 ...... *160*

M.4. NMR spectra of derivatisation products S4, 6 and S5 ...... *196*

N. Chiral HPLC Chromatograms 204

A. General Information

NMR experiments: NMR spectra (^{1}H, ^{13}C and ^{19}F) were recorded on a Bruker Avance III HD 400 (400 MHz) spectrometer equipped with a CryoProbe™ Prodigy or a Bruker Avance Neo 500 (500 MHz) equipped with a broadband *i*Prob. The chemical shifts (δ) for ^{1}H and ^{13}C are given in ppm relative to residual signals of the solvents ($CHCl_3$, 7.26 ppm, ^{1}H NMR, 77.16 ppm ^{13}C NMR). Coupling constants are given in Hz. The following abbreviations are used to indicate the multiplicity: s, singlet; d, doublet; t, triplet; q, quartet; m, multiplet; bs, broad signal. NMR yields were calculated by using 1,3,5-trimethoxybenzene as internal standards.

High-Resolution Mass Spectra (HRMS) were obtained with an Agilent Technologies G1969A LC/MSD-TOF instrument using electrospray ionisation (ESI^{+}).

Chiral High-Performance Liquid Chromatography (HPLC) analyses were performed on a Shimadzu LC-40D instrument with DGU-405 and a SPD-M40 UV/VIS detector using Phenomenex Lux® 5 µm Cellulose-1, Cellulose-2, Amylose-1 LC Columns 254 x 4.6 mm and DAICEL Chiralpak IC 5 µm LC Column 250 x 4.6 mm in *n*-hexane/2-propanol mixtures.

Chiral Gas Chromatography (GC) analyses were performed on a Shimadzu GC-2010 instrument using an Agilent CP-Chirasil Dex CB GC Column 25 m x 0.32 mm x 0.25 µm-20 to 200/225 C.

Gas Chromatography (GC) analyses were performed on a Shimadzu GC-2010 Pro instrument using an Agilent DB-5 GC column 30 m x 0.250 mm x 0.25 µm-60 to 325/350 C.

Chromatographic purification of products was accomplished by flash chromatography in Sigma Aldrich silica gel 60 N (spherical, particle size 63−210 mm). Thin-layer chromatography (TLC) was carried out with TLC plates with silica gel 60 F254. Visualization of the developed chromatography was performed by checking UV absorbance (254nm) as well as with aqueous ceric ammonium molybdate and potassium permanganate solutions. Organic solutions were concentrated under reduced pressure on Büchi and IKA rotary evaporators.

Optical rotations were measured in a Jasco P-2000 polarimeter employing a sodium lamp in $CHCl_3$ at 25 ºC.

Materials: Commercial grade reagents and solvents were purchased at the highest commercial quality from Sigma Aldrich, Apollo Scientific, TCI, Fluorochem and BLD Pharma and used as received, unless otherwise stated. Catalysts **5a-5d**, **5f-5h** and **5m** were commercially available from Sigma-Aldrich, TCI and Angene Chemical. Catalysts **5e**[1a], (**5i**, **5j**)[1b], **5k**[1c], **5l**[1d] and **5n**[1e] have been synthesized following reported procedures.

B. Synthesis of Starting Materials

B.1. General Procedure A** – Synthesis of Morita-Baylis-Hillman alcohols **S3

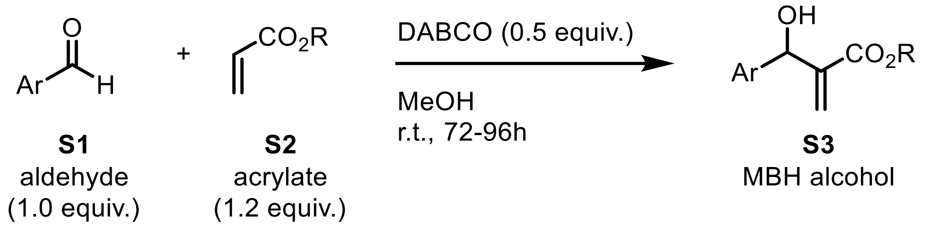

The reaction was carried out following the previously described procedure.[2a]

To a round bottom flask equipped with a magnetic stirring bar, MeOH (0.75 equiv., 37.5 mmol), the corresponding aldehyde **S1** (1.0 equiv., 50 mmol) and acrylate **S2** (1.2 equiv., 60 mmol) were added at room temperature. Then, DABCO **5g** (0.5 equiv., 25 mmol) was added, and the solution was stirred for 72-96 h until full consumption of the aldehyde, determined by TLC analysis. The crude reaction mixture was purified by flash chromatography on silica gel using mixtures of hexane/ethyl acetate as eluent to obtain alcohols **S3** in pure form.

All the spectroscopic data of the Morita-Baylis-Hillman alcohols match with the previously reported data.[2]

B.2. General Procedure B – Synthesis of allyl fluorides 1

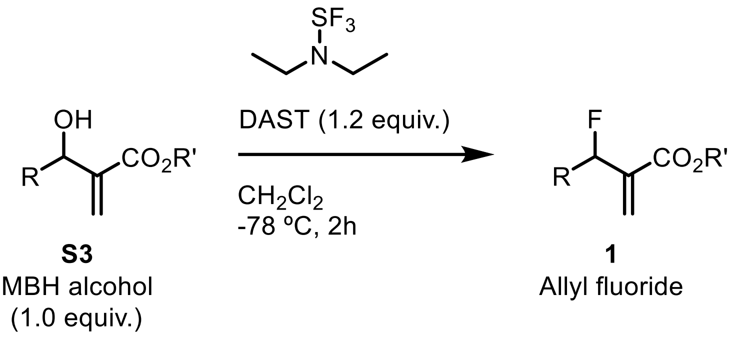

The reaction was carried out following the previously described procedure.[3b]

Into a two-necked round-bottom flask equipped with a magnetic stirring bar, the corresponding MBH alcohol **S3** (1.0 equiv., 15 mmol) was dissolved in dry CH_2Cl_2 (0.33 M) at -78 °C under nitrogen atmosphere. To this solution, DAST (1.2 equiv., 18 mmol) was added dropwise. The reaction mixture was stirred for 2 h at -78 °C and then quenched with saturated $NaHCO_3$ aqueous solution. The aqueous layer was extracted with CH_2Cl_2 (3x50 mL). The combined organic layers were dried over anhydrous $MgSO_4$ and concentrated under reduced pressure. The crude product **1** was purified by flash chromatography on silica gel using mixtures of *n*-hexane/CH_2Cl_2 to obtain allyl fluorides **1** in pure form.

*The known compounds (**1a**, **1c**, **1g**, **1i**, **1j**, **1l**)*[3a]*, (**1b**, **1n**, **1o**)*[3b]*, (**1d**, **1k**, **1m**)*[3c] *and (**1e**, **1f**, **1h**)*[3d] *were prepared according to the previous literatures, and all the spectra data are in agreement with the reports.*

B.3. General Procedure C** – Synthesis of gem-difluoroalkenes **2

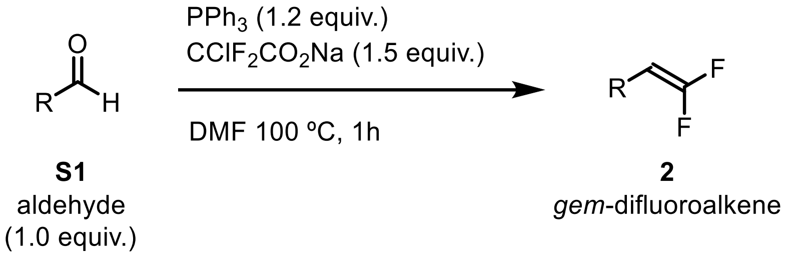

The reaction was carried out following the previously described procedure.[4b]

Into a round-bottom flask equipped with a magnetic stirring bar, the corresponding aldehyde **S1** (1.0 equiv., 15 mmol) and PPh_3 (1.2 equiv, 18 mmol) were dissolved in dry DMF (0.5 mmol/mL). The mixture was heated at 100 °C under nitrogen atmosphere. To this solution, sodium chlorodifluoroacetate (1.5 equiv.) in DMF (2 mmol/mL) was added dropwisely during 30 min. After the addition, the reaction mixture was heated for 30 min at 100 °C. After cooling to 0 °C, water was added to the reaction mixture and extracted with Et_2O. The combined organic phases were washed with water and brine, dried over anhydrous $MgSO_4$, and the resulting crude solution was concentrated under reduced pressure. The crude product **2** was purified by flash chromatography on silica gel using mixtures of *n*-hexane/ethyl acetate to obtain *gem*-difluoroalkenes **2** in pure form.

*The known compounds (**2a**, **2b**, **2h**, **2i**, **2p**, **2r**)[4a], (**2c**, **2n**, **2v**)[4b], **2d**[4c], (**2e**, **2j**)[4d], **2m**[4e], **2o**[4f], **2q**[4g], **2s**[4h], **2t**[4i], **2u**[4j] and **2w**[4k] were prepared according to the previous literatures, and all the spectra data are in agreement with the reports.*

4-(2,2-difluorovinyl)phenyl benzenesulfinate (2f)

***2f** was prepared following the abovementioned procedure.*

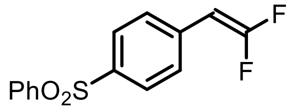

70% yield , white solid. **^{1}H NMR** (400 MHz, $CDCl_3$) δ 7.97 – 7.92 (m, 2H), 7.90 (d, J = 8.6 Hz, 2H), 7.61 – 7.54 (m, 1H), 7.54 – 7.47 (m, 2H), 7.44 (dd, J = 8.4, 0.5 Hz, 2H), 5.32 (dd, J = 25.6, 3.4 Hz, 1H) ppm; **^{13}C NMR** (101 MHz, $CDCl_3$) δ 157.1 (dd, $^1J_{CF}$ = 301.1, 292.0 Hz), 141.6, 139.9, 135.9 (t, $^3J_{CF}$ = 7.0 Hz), 133.4, 129.4, 128.3 (dd, $^4J_{CF}$ = 6.8, 3.6 Hz), 128.2, 127.7, 81.7 (dd, $^2J_{CF}$ = 30.4, 12.9 Hz) ppm; **^{19}F NMR** (471 MHz, $CDCl_3$) δ -78.1 (dd, J = 25.6, 20.9 Hz, 1F), -79.6 (dd, J = 20.9, 3.4 Hz, 1F) ppm; **HRMS (ESI)** Calculated for $[C_{14}H_{11}O_2F_2S]^+$ ($[M+H]^+$) 280.0370. Found 280.0372.

4-(2,2-difluorovinyl)-*N*,*N*-dimethylbenzenesulfonamide (2g)

2g was prepared following the abovementioned procedure.

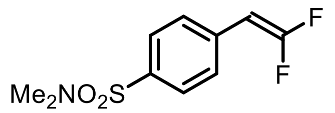

42% yield, white solid. **^{1}H NMR** (400 MHz, $CDCl_3$) δ 7.74 (d, *J* = 8.5 Hz, 1H), 7.48 (d, *J* = 8.5 Hz, 1H), 5.36 (dd, *J* = 25.6, 3.5 Hz, 1H), 2.71 (s, 6H) ppm; **^{13}C NMR** (101 MHz, $CDCl_3$) δ 157.1 (dd, $^1J_{CF}$ = 300.8, 291.7 Hz), 135.3 (t, $^3J_{CF}$ = 6.9 Hz), 133.9, 128.3, 128.01 (dd, $^4J_{CF}$ = 6.7, 3.6 Hz), 81.8 (dd, $^2J_{CF}$ = 30.3, 13.0 Hz), 38.1 ppm; **^{19}F NMR** (471 MHz, $CDCl_3$) δ -78.5 (dd, *J* = 25.6, 22.1 Hz, 1F), -80.1 (dd, *J* = 22.1, 3.4 Hz, 1F) ppm; **HRMS (ESI)** Calculated for $[C_{10}H_{11}O_2NF_2NaS]^+$ ($[M+H]^+$) 270.0371. Found 270.0373.

2-bromo-6-(2,2-difluorovinyl)pyridine (2k)

2k was prepared following the abovementioned procedure.

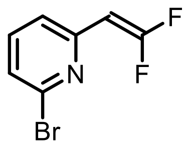

22% yield, dark brown oil. **^{1}H NMR** (500 MHz, $CDCl_3$) δ 7.52 (t, *J* = 7.8 Hz, 1H), 7.39 (dt, *J* = 7.8, 0.9 Hz, 1H), 7.33 (dt, *J* = 7.7, 0.6 Hz, 1H), 5.52 (dd, *J* = 25.5, 3.4 Hz, 1H) ppm; **^{13}C NMR** (126 MHz, $CDCl_3$) δ 158.1 (dd, $^1J_{CF}$ = 302.0, 292.5 Hz), 152.0 (dd, $^3J_{CF}$ = 11.1, 7.7 Hz), 141.8, 138.9, 126.1, 121.1 (dd, $^4J_{CF}$ = 10.4, 3.0 Hz), 83.7 (dd, $^2J_{CF}$ = 30.3, 11.5 Hz) ppm; **^{19}F NMR** (471 MHz, $CDCl_3$) δ -75.6 (dd, *J* = 25.5, 16.3 Hz, 1F), -78.6 (dd, *J* = 16.2, 3.4 Hz, 1F) ppm; **HRMS (ESI)** Calculated for $[C_7H_5NBrF_2]^+$ ($[M+H]^+$) 219.9568. Found 219.9567.

2-chloro-3-(2,2-difluorovinyl)pyridine (2l)

2l was prepared following the abovementioned procedure.

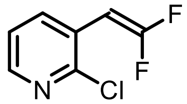

20% yield, reddish oil. **^{1}H NMR** (400 MHz, $CDCl_3$) δ 8.27 (dd, *J* = 4.7, 1.9 Hz, 1H), 7.84 (dddd, *J* = 7.8, 1.8, 1.2, 0.5 Hz, 1H), 7.29 – 7.21 (m, 1H), 5.66 (dd, *J* = 25.4, 3.1 Hz, 1H) ppm; **^{13}C NMR** (126 MHz, $CDCl_3$) δ 157.2 (dd, $^1J_{CF}$ = 299.8, 291.6 Hz), 149.7 (d, $^4J_{CF}$ = 5.6 Hz), 148.0, 137.3 (d, $^4J_{CF}$ = 9.9 Hz), 125.9 (dd, $^3J_{CF}$ = 8.2, 7.6 Hz), 122.7, 78.3 (dd, $^2J_{CF}$ = 33.5, 12.0 Hz) ppm; **^{19}F NMR** (471 MHz, $CDCl_3$) δ -78.6 (dd, *J* = 21.3, 3.1 Hz, 1F), -80.8 (dd, *J* = 25.3, 21.3 Hz, 1F) ppm; **HRMS (ESI)** Calculated for $[C_7H_5NClF_2]^+$ ($[M+H]^+$) 176.0073. Found 176.0073.

C. γ-Selective Racemic Cross-Electrophile Catalytic Coupling between allyl fluorides (1) and gem-difluoroalkenes (2)

General procedure

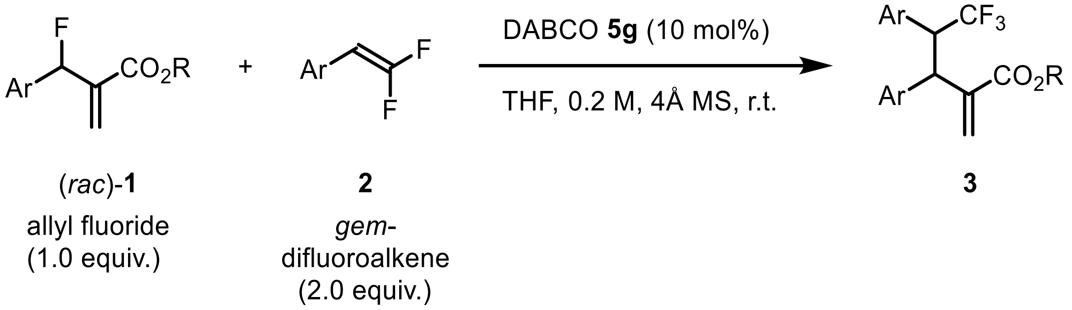

The corresponding allyl fluoride **1** (1 equiv., 0.2 mmol) was weighted into a 5 mL vial equipped with a magnetic stirring bar, 75 mg of 4Å MS, and dissolved with 1 mL of distilled THF (0.2 M). Subsequently, the corresponding *gem*-difluoroalkene **2** (2 equiv., 0.4 mmol) and a 10 mol% of DABCO **5g** were sequentially added. The reaction mixture was stirred at room temperature until full consumption of starting material **1**. The crude product **3** was directly purified by flash column chromatography on silica gel using mixtures of *n*-hexane/diethyl ether as eluent.

The characterisation of the homoallylic trifluoromethylated products **3a-3x** is shown in section E. The characterisation of the products **3y-3ac**, which were unreactive under catalytic asymmetric conditions but could be formed under catalytic racemic conditions, is shown below.

methyl 4-(3-cyanophenyl)-5,5,5-trifluoro-2-methylene-3-phenylpentanoate (3y)

*(rac)-**3y** was prepared following the general procedure described in section **C**.*

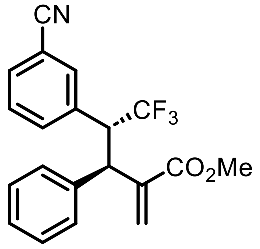

66% yield (47 mg, 0.13 mmol) (d.r. = 1.1:1), colouress oil. **^{1}H NMR** (500 MHz, $CDCl_3$) δ 7.51 (t, J = 1.8 Hz, 1H), 7.46 (dt, J = 8.1, 1.5 Hz, 1H), 7.43 (dt, J = 7.8, 1.3 Hz, 1H), 7.29 (td, J = 7.8, 0.5 Hz, 1H), 7.16 – 7.12 (m, 2H), 7.12 – 7.07 (m, 2H), 7.06 – 7.01 (m, 1H), 6.31 (s, 1H), 5.98 (d, J = 0.5 Hz, 1H), 4.70 (dq, J = 11.9, 8.4 Hz, 1H), 4.44 (d, J = 12.0 Hz, 1H), 3.76 (s, 3H) ppm; **^{13}C NMR** (126 MHz, $CDCl_3$) δ 166.2, 140.3, 138.2, 135.9, 134.1, 133.0, 131.6, 129.2, 128.8, 128.5, 127.4, 127.1, 126.1 (q, $^1J_{CF}$ = 281.0 Hz), 118.3, 112.6, 52.1, 51.3 (q, $^2J_{CF}$ = 25.3 Hz), 49.9 ppm; **^{19}F NMR** (471 MHz, $CDCl_3$) δ -65.0 (d, $^3J_{HF}$ = 8.4 Hz, 3F); **HRMS (ESI)** Calculated for $[C_{20}H_{17}O_2NF_3]^+$ ($[M+H]^+$) 360.1206. Found 360.1203.

methyl 5,5,5-trifluoro-2-methylene-4-(3-nitrophenyl)-3-phenylpentanoate (3z)

*(rac)-**3z** and (rac)-**3z'** were prepared following the general procedure described in section **C**.*

92% yield (70 mg, 0.18 mmol) of a 1.3:1 diastereomeric ratio.

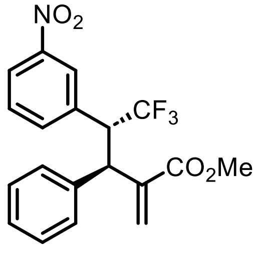

(rac)-**3z**, colouress oil. **^{1}H NMR** (500 MHz, $CDCl_3$) δ 8.12 (t, *J* = 2.1 Hz, 1H), 8.01 (ddd, *J* = 8.3, 2.2, 1.0 Hz, 1H), 7.56 (dt, *J* = 7.7, 1.4 Hz, 1H), 7.36 (t, *J* = 8.0 Hz, 1H), 7.18 – 7.15 (m, 2H), 7.08 (ddd, *J* = 7.7, 6.8, 1.2 Hz, 2H), 7.04 – 6.98 (m, 1H), 6.33 (s, 1H), 6.00 (s, 1H), 4.81 (dq, *J* = 12.0, 8.4 Hz, 1H), 4.50 (d, *J* = 12.0 Hz, 1H), 3.76 (s, 3H) ppm; **^{13}C NMR** (126 MHz, $CDCl_3$) δ 166.4, 148.2, 138.3, 136.5, 135.9, 128.9, 128.7, 127.6, 127.2, 126.3 (q, $^{1}J_{CF}$ = 281.6 Hz), 124.5, 123.2, 52.2, 51.4 (q, $^{2}J_{CF}$ = 25.7 Hz), 50.0 ppm; **^{19}F NMR** (471 MHz, $CDCl_3$) δ -65.1 (d, $^{3}J_{HF}$ = 8.3 Hz, 3F); **HRMS (ESI)** Calculated for $[C_{19}H_{17}O_4NF_3]^+$ ($[M+H]^+$) 380.1104. Found 380.1111.

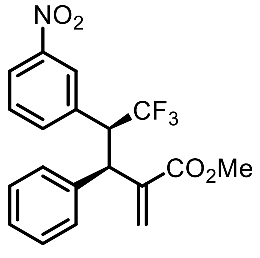

(rac)-**3z'**, colouress oil. **^{1}H NMR** (500 MHz, $CDCl_3$) δ 8.24 (t, *J* = 2.1 Hz, 1H), 8.19 (ddd, *J* = 8.3, 2.3, 1.0 Hz, 1H), 7.72 (dt, *J* = 7.7, 1.3 Hz, 1H), 7.54 (t, *J* = 8.0 Hz, 1H), 7.49 – 7.46 (m, 2H), 7.37 – 7.33 (m, 2H), 7.31 – 7.26 (m, 1H), 6.03 (s, 1H), 5.63 (s, 1H), 4.63 (d, *J* = 12.2 Hz, 1H), 4.52 (dq, *J* = 12.1, 7.9 Hz, 1H), 3.59 (s, 3H) ppm; **^{13}C NMR** (126 MHz, $CDCl_3$) δ 166.3, 148.4, 140.3, 138.9, 136.7, 135.7, 129.8, 128.8, 128.5, 127.7, 127.7, 126.0 (q, $^{1}J_{CF}$ = 281.8 Hz), 124.8, 123.5, 52.6 (q, $^{2}J_{CF}$ = 26.4 Hz), 52.2, 48.7 ppm; **^{19}F NMR** (471 MHz, $CDCl_3$) δ -64.5 (d, $^{3}J_{HF}$ = 7.9 Hz, 3F); **HRMS (ESI)** Calculated for $[C_{19}H_{17}O_4NF_3]^+$ ($[M+H]^+$) 380.1104. Found 380.1111.

methyl 5,5,5-trifluoro-2-methylene-3-phenyl-4-(4-(trifluoromethyl)phenyl)pentanoate (3aa)

*(rac)-**3aa** was prepared following the general procedure described in section **C**. This product has been characterized as a mixture of diastereoisomers* (d.r. = 1.6:1).

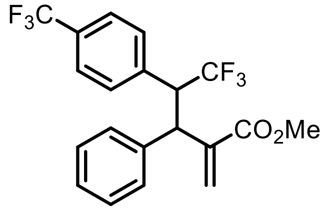

74% yield (60 mg, 0.15 mmol) (d.r. = 1.6:1), colourless oil. **^{1}H NMR** (500 MHz, $CDCl_3$) (for major diastereomer) δ 7.45 – 7.41 (m, 2H), 7.37 – 7.33 (m, 2H), 7.19 – 7.14 (m, 2H), 7.09 (ddd, *J* = 7.7, 6.8, 1.3 Hz, 2H), 7.05 – 7.00 (m, 1H), 6.32 (s, 1H), 5.98 (s, 1H), 4.77 – 4.69 (m, 1H), 4.50 (d, *J* = 11.9 Hz, 1H), 3.75 (s, 3H), (for minor diastereomer) δ 7.63 – 7.58 (m, 2H), 7.51 – 7.45 (m, 4H), 7.35 – 7.32 (m, 2H), 7.30 – 7.26 (m, 1H), 6.05 (s, 1H), 5.59 (d, *J* = 0.7 Hz, 1H), 4.67 (d, *J* = 12.3 Hz, 1H), 4.37 (dq, *J* = 12.3, 8.2 Hz, 1H), 3.59 (s, 3H); **^{13}C NMR** (126 MHz, $CDCl_3$) (for major diastereomer) δ 166.4, 140.8, 138.6, 138.4, 130.2 (q, $^{2}J_{CF}$ = 32.6 Hz), 130.1, 129.0, 128.5, 127.6, 127.1, 126.2 (q, $^{1}J_{CF}$ = 281.6 Hz), 125.4 (q, $^{3}J_{CF}$ = 3.8 Hz), 124.0 (q, $^{1}J_{CF}$ = 272.1 Hz), 52.1, 51.6 (q, $^{2}J_{CF}$ = 25.3 Hz), 49.9, (for minor diastereomer) δ 166.4, 140.4, 139.2, 138.7, 130.6 (q, $^{2}J_{CF}$ = 33.0 Hz), 130.1, 128.7, 128.6, 127.4, 126.5 (q, $^{1}J_{CF}$ = 281.5 Hz), 125.7 (q, $^{3}J_{CF}$ = 3.8 Hz), 124.1 (q, $^{1}J_{CF}$ = 272.2 Hz), 52.2, 52.9 (q, $^{2}J_{CF}$ = 25.1 Hz), 48.1; **^{19}F NMR** (471 MHz, $CDCl_3$) (for major diastereomer) δ -62.8 (s, 3F), -64.9 (d, $^{3}J_{HF}$ = 8.5 Hz, 3F), (for minor diastereomer) δ -62.7 (s, 3F), -64.3 (d, $^{3}J_{HF}$ = 8.2 Hz, 3F); **HRMS (ESI)** Calculated for $C_{20}H_{17}O_2F_6^+$ ($[M+H]^+$) 403.1127. Found 403.1138.

methyl 4-(4-(diethylcarbamoyl)phenyl)-5,5,5-trifluoro-2-methylene-3-phenylpentanoate (3ab)

*(rac)-**3ab** and (rac)-**3ab'** were prepared following the general procedure described in section **C**.*

30% yield (26 mg, 0.06 mmol) of a 1.4:1 diastereomeric ratio.

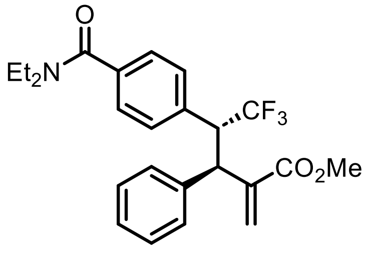

(rac)-**3ab**,white solid. **^{1}H NMR** (400 MHz, $CDCl_3$) δ 7.23 (d, *J* = 8.0 Hz, 2H), 7.19 – 7.11 (m, 4H), 7.04 (tt, *J* = 6.6, 0.9 Hz, 2H), 7.00 – 6.95 (m, 1H), 6.31 (s, 1H), 5.96 (s, 1H), 4.60 (dq, *J* = 11.8, 8.4 Hz, 1H), 4.48 (d, *J* = 11.9 Hz, 1H), 3.74 (s, 3H), 3.47 (bs, 2H), 3.03 (bs, 2H), 1.18 (bs, 3H), 0.98 (bs, 3H) ppm; **^{13}C NMR** (101 MHz, $CDCl_3$) δ 170.8, 166.5, 140.9, 138.9, 136.6, 135.4, 129.8, 129.1, 128.3, 127.1, 126.8, 126.7 (q, $^1J_{CF}$ = 281.5 Hz), 126.4, 52.1, 51.7 (q, $^2J_{CF}$ = 25.1 Hz), 49.8, 43.3, 39.4, 14.2, 13.0 ppm; **^{19}F NMR** (376 MHz, $CDCl_3$) δ -64.8 (d, $^3J_{HF}$ = 8.4 Hz, 3F); **HRMS (ESI)** Calculated for $[C_{24}H_{27}O_3NF_3]^+$ ($[M+H]^+$) 434.1938. Found 434.1934.

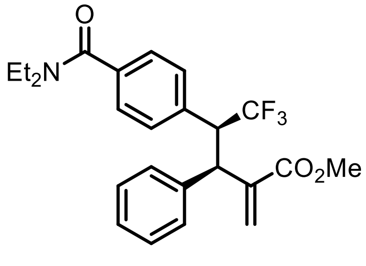

(rac)-**3ab'**, white solid. **^{1}H NMR** (400 MHz, $CDCl_3$) δ 7.48 – 7.42 (m, 2H), 7.40 – 7.29 (m, 6H), 7.28 – 7.22 (m, 1H), 6.03 (s, 1H), 5.58 (d, *J* = 0.8 Hz, 1H), 4.66 (d, *J* = 12.2 Hz, 1H), 4.29 (dq, *J* = 12.2, 8.3 Hz, 1H), 3.57 (s, 3H), 3.54 (s, 2H), 3.24 (s, 2H), 1.24 (s, 3H), 1.11 (s, 3H) ppm; **^{13}C NMR** (101 MHz, $CDCl_3$) δ 170.8, 166.4, 140.4, 139.6, 137.1, 135.7, 129.7, 128.6, 127.4, 127.1, 126.8, 126.4 (q, $^1J_{CF}$ = 281.7 Hz), 52.9 (q, $^2J_{CF}$ = 25.0 Hz), 52.1, 48.0, 43.5, 39.5, 14.3, 13.0 ppm; **^{19}F NMR** (376 MHz, $CDCl_3$) δ -64.4 (d, $^3J_{HF}$ = 8.4 Hz, 3F); **HRMS (ESI)** Calculated for $[C_{24}H_{27}O_3NF_3]^+$ ($[M+H]^+$) 434.1938. Found 434.1934.

methyl 4-(3,5-bis(trifluoromethyl)phenyl)-5,5,5-trifluoro-2-methylene-3-phenylpentanoate (3ac)

*(rac)-**3ac** was prepared following the general procedure described in section **C**. This product has been characterized as a mixture of diastereoisomers* (d.r. = 1.6:1).

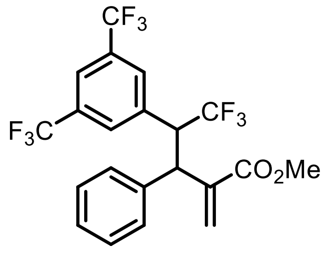

90% yield (85 mg, 0.18 mmol) (d.r. = 1.6:1), colourless oil. **^{1}H NMR** (500 MHz, $CDCl_3$) (for major diastereomer) δ 7.64 (s, 1H), 7.63 (s, 2H), 7.14 – 7.11 (m, 2H), 7.09 (ddd, J = 7.8, 6.7, 1.2 Hz, 2H), 7.05 – 7.00 (m, 1H), 6.35 (s, 1H), 6.01 (s, 1H), 4.83 (dq, J = 11.8, 8.3 Hz, 1H), 4.46 (d, J = 11.8 Hz, 1H), 3.77 (s, 3H), (for minor diastereomer) δ 7.84 (tt, J = 1.6, 0.8 Hz, 1H), 7.82 (s, 2H), 7.51 – 7.47 (m, 2H), 7.38 – 7.33 (m, 2H), 7.31 – 7.27 (m, 1H), 6.01 (s, 1H), 5.61 (s, 1H), 4.64 (dq, J = 12.0, 7.8 Hz, 1H), 4.56 (d, J = 12.1 Hz, 1H), 3.58 (s, 3H); **^{13}C NMR** (126 MHz, $CDCl_3$) (for major diastereomer) δ 166.4, 140.2, 138.1, 137.1, 131.7 (q, $^2J_{CF}$ = 33.5 Hz), 129.9, 128.8, 128.7, 127.6, 127.4, 122.0 (q, $^3J_{CF}$ = 4.0 Hz), 126.1 (q, $^1J_{CF}$ = 281.5 Hz), 123.1 (q, $^1J_{CF}$ = 272.7 Hz), 52.2, 51.6 (q, $^2J_{CF}$ = 25.7 Hz), 50.1, (for minor diastereomer) δ 166.2, 140.3, 138.8, 137.3, 132.1 (q, $^2J_{CF}$ = 33.6 Hz), 130.1, 128.9, 128.4, 128.1, 127.8, 122.4 (q, $^3J_{CF}$ = 3.8 Hz), 125.9 (q, $^1J_{CF}$ = 281.7 Hz), 123.2 (q, $^1J_{CF}$ = 272.6 Hz), 52.5 (q, $^2J_{CF}$ = 25.6 Hz), 52.2, 49.3; **^{19}F NMR** (471 MHz, $CDCl_3$) (for major diastereomer) δ -63.1 (s, 6F), -65.0 (d, $^3J_{HF}$ = 8.4 Hz, 3F), (for minor diastereomer) δ -63.0 (s, 6F), -64.6 (d, $^3J_{HF}$ = 7.7 Hz, 3F); **HRMS (ESI)** Calculated for $C_{21}H_{16}O_2F_9^+$ ([M+H]$^+$) 471.1001. Found 471.1011.

D. Optimisation of the γ-Selective Asymmetric Cross-Electrophile Catalytic Coupling between allyl fluorides (1) and gem-difluoroalkenes (2)

D.1. Optimisation – Catalyst screening

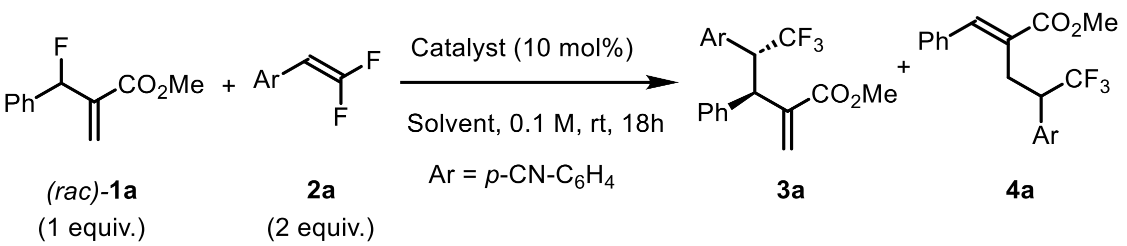

Ent.	Catalyst	Solvent	Conv. (%)[a]	3a (%)[a]	r.r. (3a:4a)[a]	d.r.[a]	e.r.[b]
**1**	(DHQD)$_2$PHAL **5a**	Toluene	10	-	-	-	-
**2**	(DHQD)$_2$PHAL **5a**	CH_2Cl_2	58	6	1:1	2:1	-
**3**	(DHQD)$_2$PHAL **5a**	THF	29	-	-	-	-
**4**	(DHQD)$_2$PHAL **5a**	MeCN	>99	10	1:1	2:1	85:15|65:35
**5**	(DHQD)$_2$PHAL **5a**	DMF	>99	5	1:5	1:1	-
**6**	(DHQD)$_2$Pyr **5b**	Toluene	10	-	-	-	-
**7**	(DHQD)$_2$Pyr **5b**	CH_2Cl_2	44	11	4.5:1	1:1	87:13|60:40
**8**	(DHQD)$_2$Pyr **5b**	THF	20	-	-	-	-
**9**	(DHQD)$_2$Pyr **5b**	MeCN	74	7	1:2	1:1	-
**10**	(DHQD)$_2$Pyr **5b**	DMF	>99	<5	1:8	1:1	-

Table S1. Optimisation of the chiral Lewis-base catalyst. [a] Determined by ^{1}H NMR spectroscopy using 1,3,5-trimethoxybenzene as the internal standard. [b] Determined by HPLC analysis using a chiral column. All the entries were performed using 4Å MS. THF: Tetrahydrofuran, DMF: *N*,*N*-dimethylformamide.

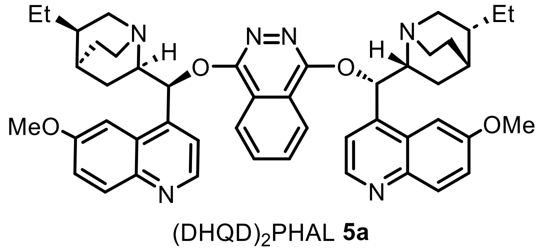

(DHQD)$_2$PHAL **5a**

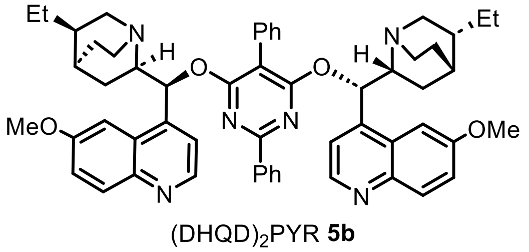

(DHQD)$_2$PYR **5b**

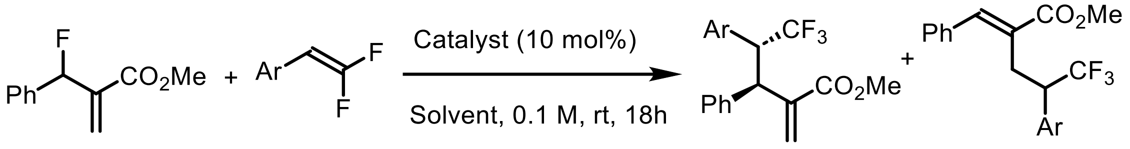

Ar = *p*-CN-C_6H_4

(rac)-**1a** (1 equiv.) **2a** (2 equiv.) **3a** **4a**

Ent	Catalyst	Solvent	Conv. (%)[a]	3a (%)[a]	r.r. (3a:4a)[a]	d.r.[a]	e.r.[b]
**1**	$(DHQD)_2AQN$ **5c**	Toluene	22	13	>20:1	6:1	97:3
**2**	$(DHQD)_2AQN$ **5c**	CH_2Cl_2	95	22	10:1	7:1	75:25
**3**	$(DHQD)_2AQN$ **5c**	THF	58	12	>20:1	9:1	94:6
**4**	$(DHQD)_2AQN$ **5c**	MeCN	>99	6	1:1	-	-
**5**	$(DHQD)_2AQN$ **5c**	DMF	>99	5	1:10	-	-
**6**	β-ICD **5d**	Toluene	70	10	>20:1	1:1	-
**7**	β-ICD **5d**	CH_2Cl_2	>99	27	1.4:1	1:1	63:37|51:49
**8**	β-ICD **5d**	THF	>99	41	>20:1	1.7:1	63:37|50:50
**9**	β-ICD **5d**	MeCN	>99	48	3.2:1	1:1	63:37|50:50
**10**	β-ICD **5d**	DMF	>99	25	1.3:1	1:1	58:42|54:46

Table S2. Optimisation of the chiral Lewis-base catalyst. [a] Determined by ^{1}H NMR spectroscopy using 1,3,5-trimethoxybenzene as the internal standard. [b] Determined by HPLC analysis using a chiral column. All the entries were performed using 4Å MS. THF: Tetrahydrofuran, DMF: *N*,*N*-dimethylformamide.

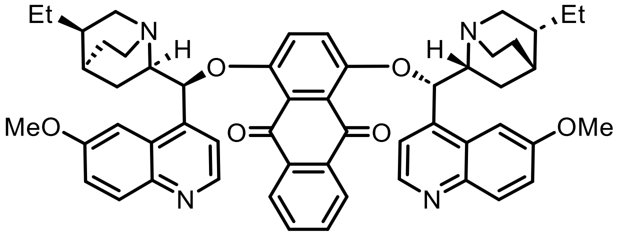

$(DHQD)_2AQN$ **5c**

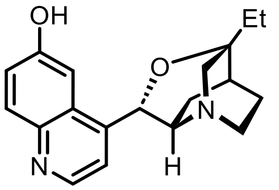

β-ICD **5d**

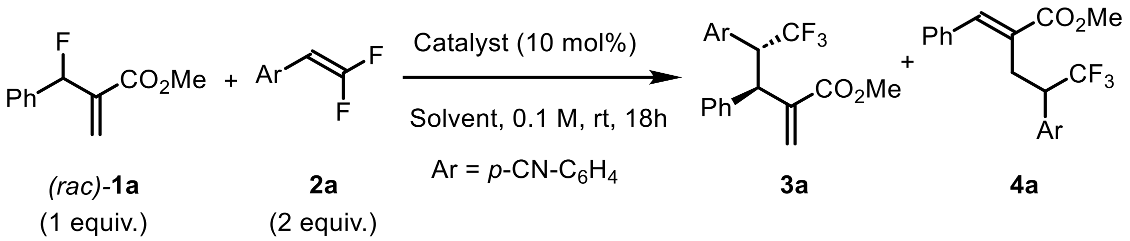

Ent.	Catalyst	Solvent	Conv. (%)[a]	3a (%)[a]	r.r. (3a:4a)[a]	d.r.[a]	e.r.[b]
**1**	**5e**	Toluene	>99	10	>20:1	1:1	-
**2**	**5e**	THF	>99	22	>20:1	2:1	27:73|62:38
**3**	PPY* **5f**	Tol	>99	30	5:1	1:1	24:76|17:83
**4**	PPY* **5f**	CH_2Cl_2	40	-	-	-	-
**5**	PPY* **5f**	THF	>99	26	1:1	1:1	29:71|19:81
**6**	$(DHQ)_2$AQN **5h**	Toluene	17	8	6:1	5:1	84:16|88:12
**7**	$(DHQ)_2$AQN **5h**	CH_2Cl_2	57	26	1.4:1	4:1	77:23|66:34
**8**	$(DHQ)_2$AQN **5h**	THF	42	13	1:1	4:1	88:12|65:35

Table S3. Optimisation of the chiral Lewis-base catalyst. [a] Determined by ^{1}H NMR spectroscopy using 1,3,5-trimethoxybenzene as the internal standard. [b] Determined by HPLC analysis using a chiral column. All the entries were performed using 4Å MS. THF: Tetrahydrofuran, DMF: *N*,*N*-dimethylformamide.

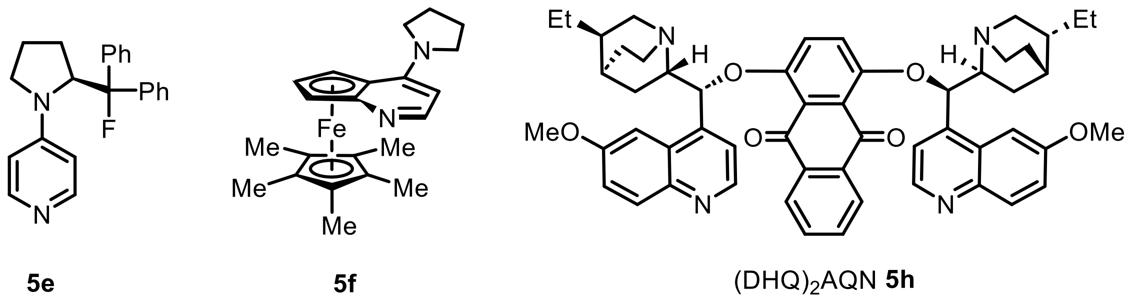

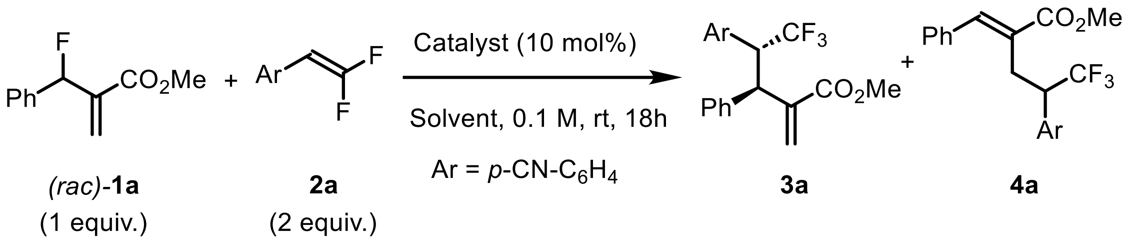

Ent.	Catalyst	Solvent	Conv. (%)[a]	3a (%)[a]	r.r. (3a:4a)[a]	d.r.[a]	e.r.[b]
**1**	Q-Thiourea **5i**	Toluene	28	-	-	-	-
**2**	Q-Thiourea **5i**	CH_2Cl_2	20	-	-	-	-
**3**	Q-Thiourea **5i**	THF	2	-	-	-	-
**4**	Q-Thiourea **5i**	MeCN	34	-	-	-	-
**5**	Q-Thiourea **5i**	DMF	26	-	-	-	-
**6**	CD-Squaramide **5j**	Toluene	26	-	-	-	-
**7**	CD-Squaramide **5j**	CH_2Cl_2	20	-	-	-	-
**8**	CD-Squaramide **5j**	THF	10	-	-	-	-
**9**	CD-Squaramide **5j**	MeCN	28	-	-	-	-
**10**	CD-Squaramide **5j**	DMF	50	-	-	-	-

Table S4. Optimisation of the chiral Lewis-base catalyst. [a] Determined by ^{1}H NMR spectroscopy using 1,3,5-trimethoxybenzene as the internal standard. [b] Determined by HPLC analysis using a chiral column. All the entries were performed using 4Å MS. THF: Tetrahydrofuran, DMF: *N*,*N*-dimethylformamide. All entries were performed using 4Å MS.

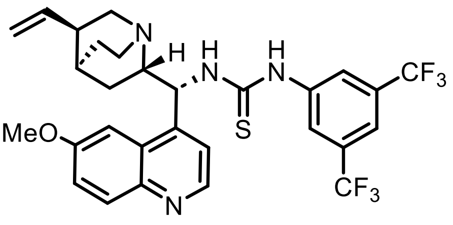

Quinine-thiourea **5i**

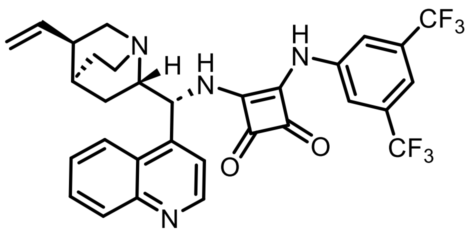

Cinchonidine-squaramide **5j**

D.2. Optimisation – Solvent screening

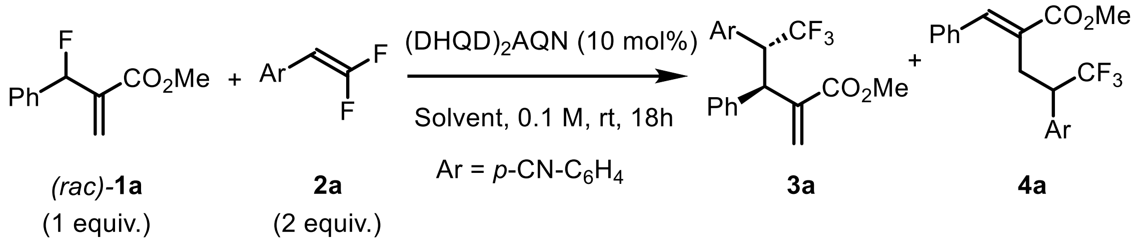

(rac)-**1a** (1 equiv.) + **2a** (2 equiv.) → **3a** + **4a**

Entry	Solvent	Conv. (%)[a]	3a (%)[a]	r.r. (3a:4a)[a]	d.r.[a]	e.r.[b]
**1**	Toluene	22	13	>20:1	6:1	97:3
**2**	CH_2Cl_2	95	22	10:1	7:1	75:25
**3**	THF	58	12	>20:1	9:1	94:6
**4**	MeCN	>99	6	1:1	-	-
**5**	DMF	>99	5	1:10	-	-
**6**	$PhCF_3$	35	30	>20:1	8:1	96:4
**7**	1,4-dioxane	19	5	>20:1	8:1	-
**8**	2-Me-THF	59	23	12:1	6:1	97:3
**9**	Et_2O	59	23	2.5:1	4:1	95:5
**10**	$PhCCl_3$	64	26	>20:1	8:1	95:5
**11**	1,2-DCE	20	-	-	-	-
**12**	1,2-DCB	54	25	>20:1	6:1	96:4

Table S5. Optimization of the solvent. [a] Determined by ^{1}H NMR spectroscopy using 1,3,5-trimethoxybenzene as the internal standard. [b] Determined by HPLC analysis using a chiral column. All the entries were performed using 4Å MS. THF:Tetrahydrofuran, DMF: *N,N*-dimethylformamide, 1,2-DCE: 1,2-dichloroethane, 1,2-DCB: 1,2-dichlorobenzene.

D.3. Optimisation – Temperature, ratio of reagents, concentration and catalyst loading

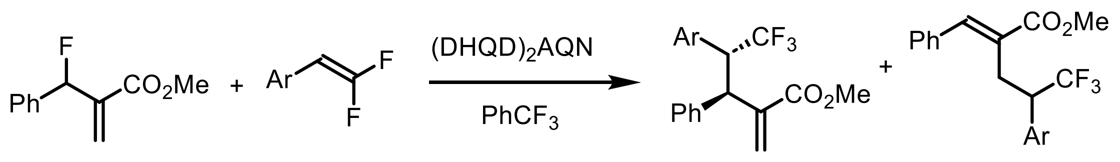

Entry	Cat. loading (mol%)	T (°C)	Conc. (M)	Time (h)	Conv. (%)[a]	3a (%)[a]	r.r. (3a:4a)[a]	d.r.[a]	e.r.[b]
**1**	10	r.t.	0.1	18	45	40	>20:1	8:1	96:4
**2**	10	4	0.1	18	45	28	>20:1	9:1	97:3
**3**	10	r.t.	0.2	18	57	50	>20:1	8:1	96:4
**4**	10	r.t.	0.3	18	80	49	>20:1	8:1	96:4
**5**	20	r.t.	0.1	18	60	53	>20:1	8:1	96:4
**6**	20	r.t.	0.2	18	76	55	>20:1	8:1	96:4
**7**	10	r.t.	0.2	24	69	55	>20:1	8:1	96:4
**8**	10	r.t.	0.2	40	78	63	>20:1	8:1	96:4
**9**	10	r.t.	0.2	64	86	69	>20:1	8:1	96:4
**10**	10	r.t	0.2	72	92	72[c]	>20:1	8:1	96:4

Table S6. Optimisation of the temperature, ratio of reagents, concentration and catalyst loading. [a] Determined by ^{1}H NMR spectroscopy using 1,3,5-trimethoxybenzene as the internal standard. [b] Determined by HPLC analysis using a chiral column. [c] Isolated yield. All the entries were performed using 4Å MS.

D.4. Role and fate of the gem-difluoroalkene in excess

We evaluated the γ-selective XEC protocol using an equimolar mixture of **1a** and **2a** under both optimized asymmetric catalytic conditions (10 mol% of $(DHQD)_2AQN$ **5c** in $PhCF_3$ 0.2M) and racemic catalytic conditions (10 mol% of DABCO **5g** in THF 0.2M).

1 equiv. of 1a and 1 equiv. of 2a under asymmetric conditions

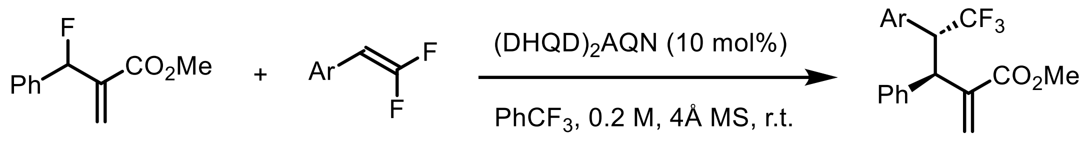

(*rac*)-**1a** (1 equiv.) | **2a** (1 equiv.) | Ar = *p*-CN-C_6H_4 | **3a**

Entry	Time (h)	Conv. (1a) (%)[a]	Conv. (2a) (%)[a]	3a (%)[a]
**1**	18	41	50	30
**2**	40	54	62	41
**3**	72	64	71	48
**4**	96	75	80	54
**5**	168	90	92	63

Table S7. Conversion and yield of the equimolar reaction at different times. [a] Determined by ^{1}H NMR spectroscopy using 1,3,5-trimethoxybenzene as the internal standard.

1 equiv. of 1a and 3 equiv. of 2a under asymmetric conditions (for comparison)

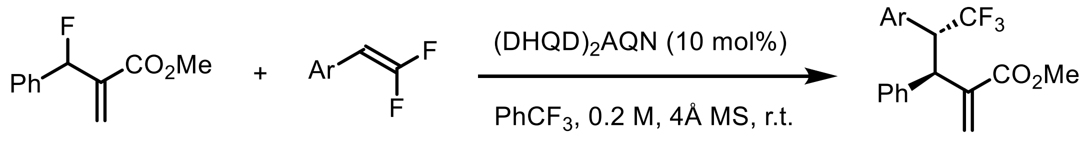

(*rac*)-**1a** (1 equiv.) | **2a** (3 equiv.) | Ar = *p*-CN-C_6H_4 | **3a**

Entry	Time (h)	Conv. (1a) (%)[a]	3a (%)[a]
**1**	18	57	50
**2**	40	78	63
**3**	72	92	72

Table S8. Conversion and yield of the reaction under optimised conditions at different times. [a] Determined by ^{1}H NMR spectroscopy using 1,3,5-trimethoxybenzene as the internal standard.

As shown in the data above, the reaction with equimolar amounts of **1a** and **2a** does proceed more slowly than the reaction with an excess of **2a**. After 18 h the equimolar reaction affords product **3a** in 30% (vs. 50% yield of the optimized reaction) and in 48% yield after 72 h (vs. 72% for the optimized reaction). After 168 h, the reaction under equimolar conditions reaches a 63% yield, after which no further conversion is observed. Therefore, while the equimolar reaction ultimately produces **3a** in only slightly lower yield (63% after 168 h with 1.0 equiv. of **2a** vs. 72% after 72h in 3.0 equiv. of **2a**), it requires significantly longer reaction times.

[The slower reactivity under equimolar conditions can be rationalized by considering the reaction mechanisms. The activation of ***1a*** *by the nucleophilic catalyst to form* ***8****–F is a highly endergonic process, particularly when the chiral catalyst* ***5c*** *is used in an apolar aromatic solvent. The extent of this equilibrium determines the effective concentration of fluoride anion available in solution to activate* ***2a****. Since the activation of* ***2a*** *to generate the nucleophilic species* ***9****$^-$ is also endergonic, and the fluoride concentration under asymmetric catalytic conditions is expected to be very low, employing an excess of gem-difluoroalkene* ***2a*** *helps to drive this second equilibrium forward, thereby increasing the concentration of carbanion* ***9****$^-$ available for C–C bond formation with the ammonium species* ***8****$^+$. Under equimolar conditions, at higher conversions, depletion of* ***2a*** *disfavours the formation of* ***9****$^-$, slowing the reaction. Accordingly, the use of an excess of* ***2a*** *maintains a higher steady-state concentration of* ***9****$^-$ throughout the reaction, enabling full conversion within 72 h.]*

1 equiv. of 1a and 1 equiv. of 2a under racemic conditions

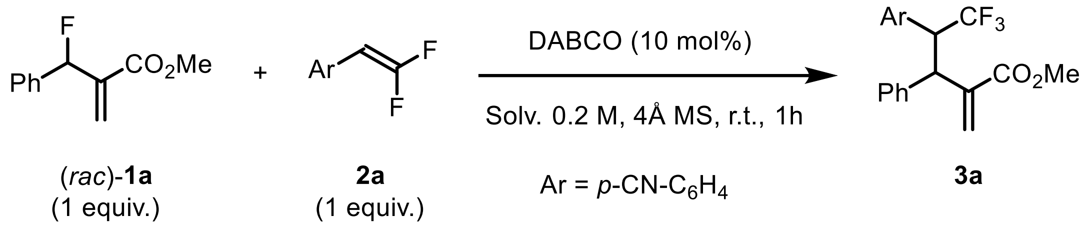

Entry	Solvent	Conv. (%)[a]	3a (%)[a]
**1**	Toluene	>99	60
**2**	$PhCF_3$	>99	61
**3**	THF	>99	80

Table S9. Equimolar racemic reactions under optimised γ-selective conditions. [a] Determined by ^{1}H NMR spectroscopy using 1,3,5-trimethoxybenzene as the internal standard.

[In the three solvents studied, the racemic product ***3a*** *was obtained in synthetically useful yields (60–80%) within 1 h. Since THF is the solvent that leads to the least endergonic activation of* ***1a*** *–and thus to a higher concentration of fluoride anion– it is consistent that this solvent affords the highest reaction yield.]*

To further understand the role of the excess reagent, we examined the fate of **2a** at the end of the reaction under the optimized catalytic conditions.

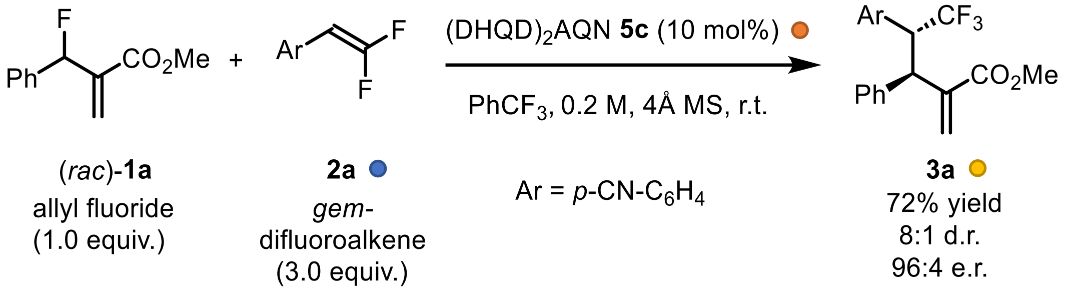

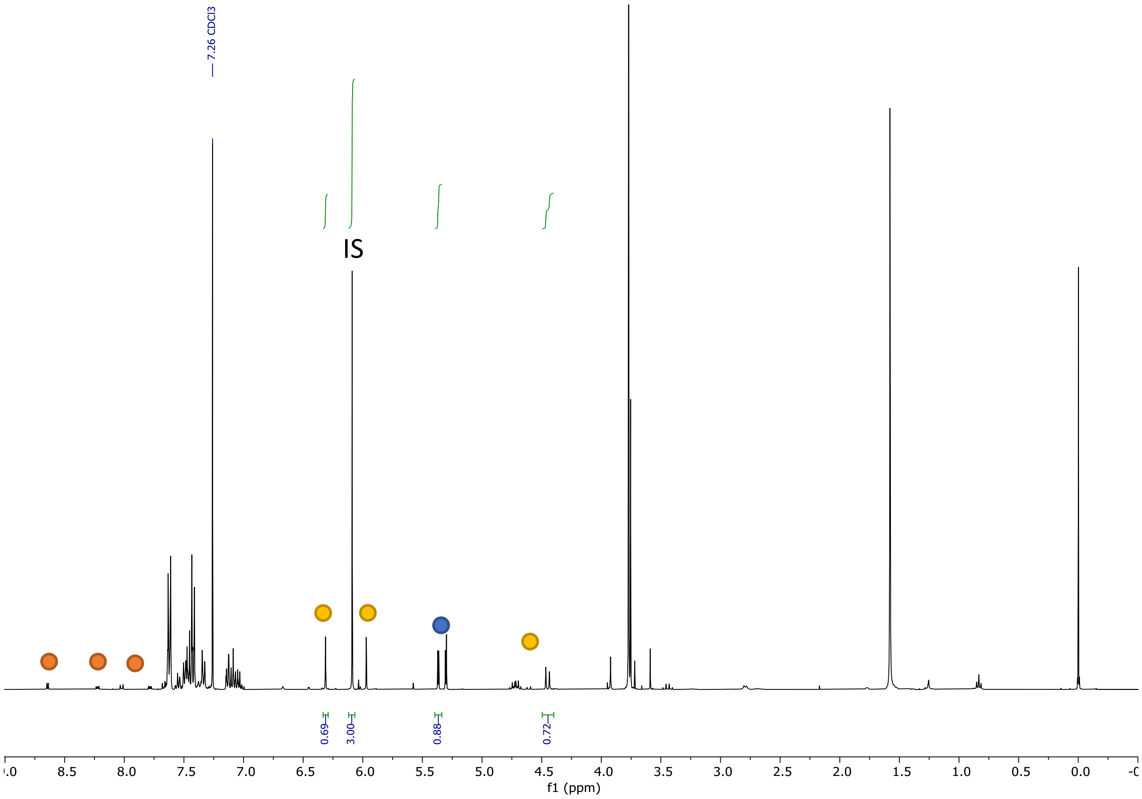

Figure S1. Crude ^{1}H NMR of the last entry of the optimization at 72 h at 400 MHz in $CDCl_3$. The NMR yield was determined using 1,3,5-trimethoxybenzene as the internal standard.

As shown in the ^{1}H NMR spectrum of the crude reaction mixture, at the end of the reaction, 1.76 equivalents of gem-difluoroalkene **2a** remain unreacted, corresponding to 88% of the 2.0 equivalents initially used in excess. In addition, after purification by flash column chromatography, the unreacted **2a** was recovered in 85% isolated yield (58 mg, 0.35 mmol).

E. γ-Selective Asymmetric Cross-Electrophile Catalytic Coupling between allyl fluorides (1) and gem-difluoroalkenes (2)

General Procedure

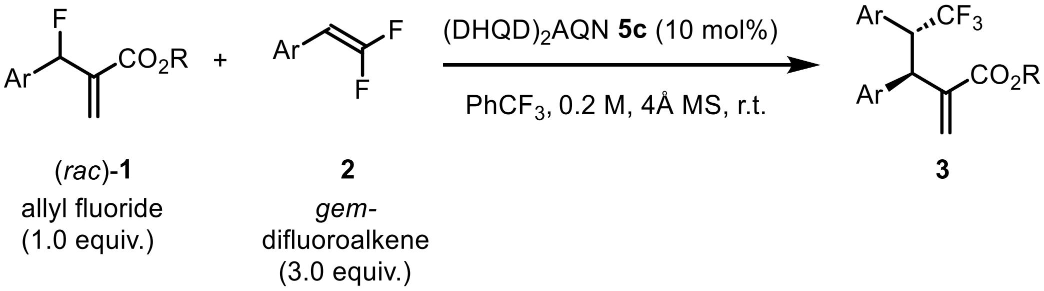

The corresponding allyl fluoride **1** (1 equiv., 0.2 mmol) was weighted into a 5 mL vial equipped with a magnetic stirring bar, 75 mg of 4Å MS, and dissolved with 1 mL of dry $PhCF_3$ (0.2 M). Subsequently, the corresponding *gem*-difluoroalkene **2** (3 equiv., 0.6 mmol) and a 10 mol% of $(DHQD)_2AQN$ **5c** were added sequentially. The reaction mixture was stirred at room temperature until full consumption of starting material **1**. The crude product **3** was directly purified by flash column chromatography on silica gel using mixtures of *n*-hexane/diethyl ether as eluent.

methyl (3*R*,4*R*)-4-(4-cyanophenyl)-5,5,5-trifluoro-2-methylene-3-phenylpentanoate (3a)

***3a** was prepared following the general procedure described in section **E**.*

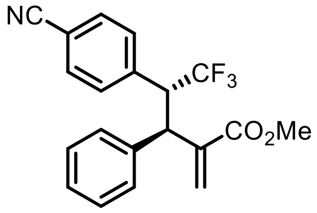

72% yield (53 mg, 0.14 mmol), colourless oil. **^{1}H NMR** (400 MHz, $CDCl_3$) δ 7.46 (d, J = 8.5 Hz, 2H), 7.34 (d, J = 8.2 Hz, 2H), 7.19 – 6.99 (m, 5H), 6.31 (s, 1H), 5.97 (s, 1H), 4.72 (dq, J = 11.9, 8.4 Hz, 1H), 4.45 (d, J = 12.0 Hz, 1H), 3.75 (s, 3H) ppm; **^{13}C NMR** (101 MHz, $CDCl_3$) δ 166.4, 140.5, 139.7 (q, $^3J_{CF}$ = 2.1 Hz), 138.4, 132.2, 130.5, 128.9, 128.6, 127.5, 127.2, 126.3 (q, $^1J_{CF}$ = 281.4 Hz), 118.4, 112.1, 52.2, 51.8 (q, $^2J_{CF}$ = 25.3 Hz), 49.9 ppm; **^{19}F NMR** (376 MHz, $CDCl_3$) δ -64.8 (d, $^3J_{HF}$ = 8.3 Hz, 3F) ppm; **HRMS (ESI)** Calculated for $[C_{20}H_{17}O_2NF_3]^+$ ($[M+H]^+$) 360.1206. Found 360.1206; **$[\alpha]_D^{25}$** = +34.2 (c = 2.66, $CHCl_3$); **HPLC** Phenomenex Lux Cellulose-1 (95:5 *n*-Hexane:2-Propanol, 1 mL/min, 245 nm); t_R (major) = 9.5 min, t_R (minor) = 7.2 min (96:4 e.r.).

methyl (3*R*,4*R*)-4-(4-cyanophenyl)-5,5,5-trifluoro-3-(4-fluorophenyl)-2-methylenepentanoate (3b)

*3b was prepared following the general procedure described in section **E**.*

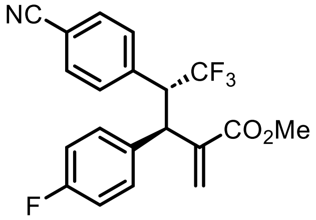

75% yield (57 mg, 0.15 mmol), colourless oil. **^{1}H NMR** (500 MHz, $CDCl_3$) δ 7.48 (d, *J* = 8.5 Hz, 2H), 7.33 (d, *J* = 8.3 Hz, 2H), 7.12 (dd, *J* = 8.8, 5.2 Hz, 2H), 6.78 (t, *J* = 8.6 Hz, 2H), 6.31 (s, 1H), 5.96 (s, 1H), 4.70 (dq, *J* = 11.9, 8.3 Hz, 1H), 4.42 (d, *J* = 11.9 Hz, 1H), 3.75 (s, 3H) ppm; **^{13}C NMR** (126 MHz, $CDCl_3$) δ 166.2, 161.6 (d, $^1J_{CF}$ = 246.5 Hz), 140.3, 139.5, 134.1 (d, $^4J_{CF}$ = 3.4 Hz), 132.3, 130.5 (d, $^3J_{CF}$ = 8.1 Hz), 130.4, 127.6, 126.2 (q, $^1J_{CF}$ = 281.9 Hz), 118.3, 115.5 (d, $^2J_{CF}$ = 21.4 Hz), 112.3, 52.2, 51.7 (q, $^2J_{CF}$ = 25.3 Hz), 49.5 ppm; **^{19}F NMR** (471 MHz, $CDCl_3$) δ -64.8 (d, $^3J_{HF}$ = 8.3 Hz, 3F), -114.9 (m, 1F) ppm; **HRMS (ESI)** Calculated for $[C_{20}H_{16}O_2NF_4]^+$ ($[M+H]^+$) 378.1112. Found 378.1115; **$[\alpha]_D^{25}$** = +32.9 (c = 3.35, $CHCl_3$); **HPLC** Phenomenex Lux Cellulose-1 (98:2 *n*-Hexane:2-Propanol, 1 mL/min, 240 nm); t_R (major) = 9.8 min, t_R (minor) = 9.2 min (95:5 e.r.).

methyl (3*R*,4*R*)-3-(4-chlorophenyl)-4-(4-cyanophenyl)-5,5,5-trifluoro-2-methylenepentanoate (3c)

*3c was prepared following the general procedure described in section **E**.*

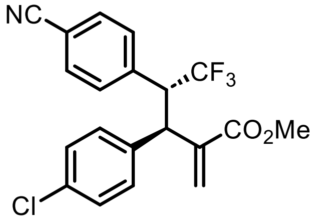

68% yield (53 mg, 0.14 mmol), colourless oil. **^{1}H NMR** (500 MHz, $CDCl_3$) δ 7.49 (d, *J* = 8.4 Hz, 2H), 7.33 (d, *J* = 8.2 Hz, 2H), 7.12 – 7.03 (m, 4H), 6.31 (s, 1H), 5.96 (s, 1H), 4.71 (dq, *J* = 11.9, 8.3 Hz, 1H), 4.41 (d, *J* = 12.0 Hz, 1H), 3.75 (s, 3H) ppm; **^{13}C NMR** (126 MHz, $CDCl_3$) δ 166.2, 140.1, 139.3, 136.8, 133.1, 132.4, 130.4, 130.3, 128.7, 127.8, 126,2 (q, $^1J_{CF}$ = 281.4 Hz), 118.3, 112.4, 52.2, 51.5 (q, $^2J_{CF}$ = 25.2 Hz), 49.6. ppm; **^{19}F NMR** (471 MHz, $CDCl_3$) δ -64.9 (d, $^3J_{HF}$ = 8.4 Hz, 3F) ppm; **HRMS (ESI)** Calculated for $[C_{20}H_{16}O_2NClF_3]^+$ ($[M+H]^+$) 394.0816. Found 394.0822; **$[\alpha]_D^{25}$** = +27.5 (c = 2.96, $CHCl_3$); **HPLC** Phenomenex Lux Cellulose-1 (98:2 *n*-Hexane:2-Propanol, 1 mL/min, 240 nm); t_R (major) = 9.7 min, t_R (minor) = 9.2 min (97:3 e.r.).

methyl (3*R*,4*R*)-4-(4-cyanophenyl)-5,5,5-trifluoro-2-methylene-3-(4-(trifluoromethyl)phenyl)pentanoate (3d)

***3d** was prepared following the general procedure described in section **E**.*

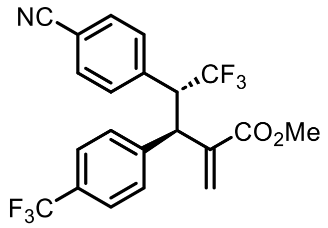

73% yield (62 mg, 0.15 mmol), colourless oil. **^{1}H NMR** (500 MHz, $CDCl_3$) δ 7.50 (d, J = 8.4 Hz, 2H), 7.39 – 7.33 (m, 4H), 7.30 (d, J = 8.3 Hz, 2H), 6.34 (s, 1H), 6.01 (s, 1H), 4.78 (dq, J = 11.9, 8.3 Hz, 1H), 4.50 (d, J = 12.0 Hz, 1H), 3.76 (s, 3H) ppm; **^{13}C NMR** (126 MHz, $CDCl_3$) δ 166.0, 142.3, 139.7, 139.1, 132.5, 130.3, 129.5 (q, $^{2}J_{CF}$ = 32.4 Hz), 129.3, 128.4, 126.2 (q, $^{1}J_{CF}$ = 281.5 Hz), 125.5 (q, $^{3}J_{CF}$ = 3.6 Hz), 123.9 (q, $^{1}J_{CF}$ = 272.4 Hz), 118.2, 112.5, 52.3, 51.3 (q, $^{2}J_{CF}$ = 25.7 Hz), 50.1 ppm; **^{19}F NMR** (471 MHz, $CDCl_3$) δ -62.7 (s, 3F), -64.9 (d, $^{3}J_{HF}$ = 8.3 Hz, 3F) ppm; **HRMS (ESI)** Calculated for $[C_{21}H_{16}O_2NF_6]^+$ ($[M+H]^+$) 428.1080. Found 428.1076; **$[\alpha]_D^{25}$** = +39.8 (c = 2.87, $CHCl_3$); **HPLC** Phenomenex Lux Amylose-1 (99:1 *n*-Hexane:2-Propanol, 1 mL/min, 240 nm); t_R (major) = 19.6 min, t_R (minor) = 17.2 min (97:3 e.r.).

methyl (3*R*,4*R*)-3,4-bis(4-cyanophenyl)-5,5,5-trifluoro-2-methylenepentanoate (3e)

***3e** was prepared following the general procedure described in section **E**.*

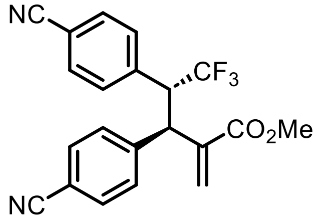

64% yield (49 mg, 0.13 mmol), yellow oil. **^{1}H NMR** (500 MHz, $CDCl_3$) δ 7.51 (d, J = 8.4 Hz, 2H), 7.40 (d, J = 8.5 Hz, 2H), 7.34 (d, J = 8.3 Hz, 2H), 7.30 (d, J = 8.4 Hz, 2H), 6.36 (s, 1H), 6.02 (s, 1H), 4.76 (dq, J = 12.0, 8.2 Hz, 1H), 4.48 (d, J = 12.0 Hz, 1H), 3.76 (s, 3H) ppm; **^{13}C NMR** (126 MHz, $CDCl_3$) δ 165.9, 143.6, 139.2, 138.8, 132.6, 132.4, 130.3, 129.8, 128.7, 126.0 (q, $^{1}J_{CF}$ = 281.4 Hz), 118.3, 118.1, 112.7, 111.4, 52.4, 51.1 (q, $^{2}J_{CF}$ = 26.0 Hz), 50.4 ppm; **^{19}F NMR** (471 MHz, $CDCl_3$) δ -64.9 (d, $^{3}J_{HF}$ = 8.2 Hz, 3F) ppm; **HRMS (ESI)** Calculated for $[C_{21}H_{16}O_2N_2F_3]^+$ ($[M+H]^+$) 385.1158. Found 385.1161; **$[\alpha]_D^{25}$** = +22.8 (c = 0.55, $CHCl_3$); **HPLC** Phenomenex Lux Cellulose-1 (95:5 *n*-Hexane:2-Propanol, 1 mL/min, 240 nm); t_R (major) = 25.9 min, t_R (minor) = 24.3 min (97:3 e.r.).

methyl 4-((3*R*,4*R*)-4-(4-cyanophenyl)-5,5,5-trifluoro-2-(methoxycarbonyl)pent-1-en-3-yl)benzoate (3f)

***3f** was prepared following the general procedure described in section **E**.*

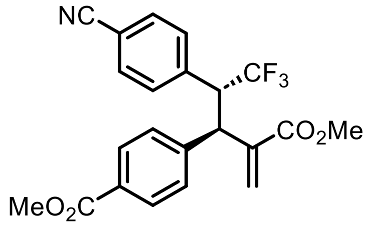

63% yield (53 mg, 0.13 mmol), colourless oil. **^{1}H NMR** (500 MHz, $CDCl_3$) δ 7.76 (d, *J* = 8.4 Hz, 2H), 7.47 (d, *J* = 8.4 Hz, 2H), 7.34 (d, *J* = 8.3 Hz, 2H), 7.23 (d, *J* = 8.4 Hz, 2H), 6.34 (s, 1H), 6.00 (s, 1H), 4.76 (dq, *J* = 12.0, 8.3 Hz, 1H), 4.51 (d, *J* = 12.0 Hz, 1H), 3.83 (s, 3H), 3.75 (s, 3H) ppm; **^{13}C NMR** (126 MHz, $CDCl_3$) δ 166.3, 165.9, 143.2, 139.5, 139.0, 132.1, 130.1, 129.6, 128.9, 128.7, 127.9, 125.9 (q, $^1J_{CF}$ = 281.6 Hz), 118.0, 112.1, 52.0, 52.0, 51.2 (q, $^2J_{CF}$ = 25.7 Hz), 49.8. ppm; **^{19}F NMR** (471 MHz, $CDCl_3$) δ -64.9 (d, $^3J_{HF}$ = 8.2 Hz, 3F) ppm; **HRMS (ESI)** Calculated for $[C_{22}H_{19}O_4NF_3]^+$ ($[M+H]^+$) 418.1261. Found 418.1266; **$[\alpha]_D^{25}$** = +19.6 (c = 0.47, $CHCl_3$); **HPLC** Phenomenex Lux Cellulose-1 (95:5 *n*-Hexane:2-Propanol, 1 mL/min, 240 nm); t_R (major) = 15.7 min, t_R (minor) = 15.0 min (98:2 e.r.).

methyl (3*R*,4*R*)-4-(4-cyanophenyl)-5,5,5-trifluoro-2-methylene-3-(p-tolyl)pentanoate (3g)

***3g** was prepared following the general procedure described in section **E**.*

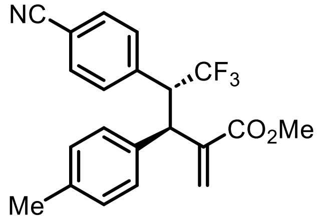

65% yield (48 mg, 0.13 mmol), colourless oil. **^{1}H NMR** (500 MHz, $CDCl_3$) δ 7.47 (d, *J* = 8.4 Hz, 2H), 7.34 (d, *J* = 8.2 Hz, 2H), 7.02 (d, *J* = 8.2 Hz, 2H), 6.89 (d, *J* = 8.1 Hz, 2H), 6.28 (s, 1H), 5.95 (s, 1H), 4.71 (dq, *J* = 11.9, 8.4 Hz, 1H), 4.42 (d, *J* = 11.9 Hz, 1H), 3.75 (s, 3H), 2.17 (s, 3H) ppm; **^{13}C NMR** (126 MHz, $CDCl_3$) δ 166.7, 141.0, 140.1, 137.1, 135.6, 132.5, 130.7, 129.5, 129.0, 127.5, δ 126.5 (q, $^1J_{CF}$ = 281.6 Hz), 118.7, 112.3, 52.4, 52.0 (q, $^2J_{CF}$ = 25.2 Hz), 49.8, 21.3 ppm; **^{19}F NMR** (471 MHz, $CDCl_3$) δ -64.8 (d, $^3J_{HF}$ = 8.5 Hz, 3F) ppm; **HRMS (ESI)** Calculated for $[C_{21}H_{19}O_2NF_3]^+$ ($[M+H]^+$) 374.1362. Found 374.1357; **$[\alpha]_D^{25}$** = +36.6 (c = 0.90, $CHCl_3$); **HPLC** Phenomenex Lux Cellulose-1 (99:1 *n*-Hexane:2-Propanol, 1 mL/min, 240 nm); t_R (major) = 12.1 min, t_R (minor) = 8.9 min (97:3 e.r.).

methyl (3*R*,4*R*)-3-(4-(tert-butyl)phenyl)-4-(4-cyanophenyl)-5,5,5-trifluoro-2-methylene pentanoate (3h)

***3h** was prepared following the general procedure described in section **E**.*

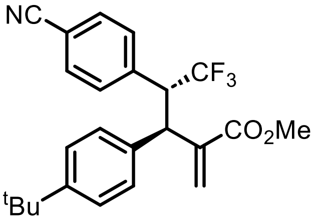

67% yield (56 mg, 0.13 mmol), colourless oil. **^{1}H NMR** (400 MHz, $CDCl_3$) δ 7.46 (d, J = 8.5 Hz, 2H), 7.33 (d, J = 8.3 Hz, 2H), 7.08 (d, J = 8.7 Hz, 2H), 7.03 (d, J = 8.5 Hz, 2H), 6.29 (s, 1H), 5.95 (s, 1H), 4.70 (dq, J = 11.9, 8.4 Hz, 1H), 4.41 (d, J = 11.9 Hz, 1H), 3.75 (s, 3H), 1.17 (s, 9H) ppm. **^{13}C NMR** (101 MHz, $CDCl_3$) δ 166.4, 150.0, 140.6, 139.9, 135.1, 132.6, 132.2, 131.1, 130.5, 128.4, 127.4, 126.4 (q, $^1J_{CF}$ = 281.6 Hz), 125.4, 118.5, 111.9, 52.2, 51.8 (q, $^2J_{CF}$ = 25.4 Hz), 49.5, 34.4, 31.3ppm; **^{19}F NMR** (376 MHz, $CDCl_3$) δ -64.7 (d, $^3J_{HF}$ = 8.4 Hz, 3F) ppm; **HRMS (ESI)** Calculated for $[C_{24}H_{25}O_2NF_3]^+$ ($[M+H]^+$) 416.1837. Found 416.1839; **$[\alpha]_D^{25}$** = +16.8 (c = 0.71, $CHCl_3$); **HPLC** Phenomenex Lux Cellulose-1 (99:1 *n*-Hexane:2-Propanol, 1 mL/min, 243 nm); t_R (major) = 12.4 min, t_R (minor) = 11.4 min (97:3 e.r.).

methyl (3*R*,4*R*)-3-(4-bromophenyl)-4-(4-cyanophenyl)-5,5,5-trifluoro-2-methylenepentanoate (3i)

***3i** was prepared following the general procedure described in section **E**.*

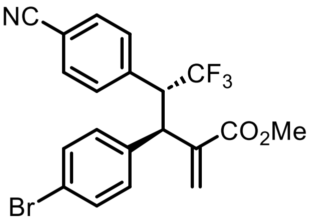

74% yield (65 mg, 0.15 mmol), colourless oil. **^{1}H NMR** (500 MHz, $CDCl_3$) δ 7.50 (d, J = 8.7 Hz, 2H), 7.33 (d, J = 8.3 Hz, 2H), 7.22 (d, J = 8.5 Hz, 2H), 7.03 (d, J = 8.8 Hz, 2H), 6.31 (s, 1H), 5.96 (s, 1H), 4.71 (dq, J = 11.9, 8.3 Hz, 1H), 4.39 (d, J = 12.0 Hz, 1H), 3.75 (s, 3H) ppm; **^{13}C NMR** (126 MHz, $CDCl_3$) δ 166.1, 140.0, 139.3, 137.4, 132.4, 131.7, 130.6, 130.4, 127.9, 126.2 (q, $^1J_{CF}$ = 281.2 Hz), 121.3, 118.3, 112.4, 52.2, 51.4 (q, $^2J_{CF}$ = 25.7 Hz), 49.7 ppm; **^{19}F NMR** (471 MHz, $CDCl_3$) δ -64.9 (d, $^3J_{HF}$ = 8.5 Hz, 3F) ppm; **HRMS (ESI)** Calculated for $[C_{20}H_{16}O_2NBrF_3]^+$ ($[M+H]^+$) 438.0311. Found 438.3016; **$[\alpha]_D^{25}$** = +22.7 (c = 3.24, $CHCl_3$); **HPLC** Phenomenex Lux Amylose-1 (98:2 *n*-Hexane:2-Propanol, 1 mL/min, 227 nm); t_R (major) = 19.6 min, t_R (minor) = 18.2 min (98:2 e.r.).

methyl (3*R*,4*R*)-4-(4-cyanophenyl)-5,5,5-trifluoro-2-methylene-3-(m-tolyl)pentanoate (3j)

***3j** was prepared following the general procedure described in section **E**.*

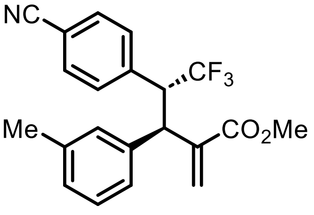

56% yield (42 mg, 0.11 mmol), colourless oil. **^{1}H NMR** (500 MHz, $CDCl_3$) δ 7.47 (d, J = 8.4 Hz, 2H), 7.34 (d, J = 8.3 Hz, 2H), 7.00 – 6.90 (m, 3H), 6.84 (dd, J = 7.1, 0.8 Hz, 1H), 6.31 (s, 1H), 5.96 (s, 1H), 4.69 (dq, J = 12.0, 8.4 Hz, 1H), 4.42 (d, J = 12.0 Hz, 1H), 3.75 (s, 3H), 2.18 (s, 3H) ppm. **^{13}C NMR** (126 MHz, $CDCl_3$) δ 166.4, 140.5, 139.8, 138.2, 138.1, 132.1, 132.0, 130.4, 129.7, 128.3, 127.9, 127.4, 126.6 (q, $^1J_{CF}$ = 281.0 Hz), 125.7, 118.4, 112.0, 52.1, 51.7 (q, $^2J_{CF}$ = 25.0 Hz) 49.6, 21.4 ppm; **^{19}F NMR** (471 MHz, $CDCl_3$) δ -64.8 (d, $^3J_{HF}$ = 8.5 Hz, 3F) ppm; **HRMS (ESI)** Calculated for $[C_{21}H_{19}O_2NF_3]^+$ ($[M+H]^+$) 374.1362. Found 374.1355; **$[\alpha]_D^{25}$** = +34.9 (c = 0.72, $CHCl_3$); **HPLC** Phenomenex Lux Cellulose-1 (99:1 *n*-Hexane:2-Propanol, 1 mL/min, 245 nm); t_R (major) = 15.9 min, t_R (minor) = 10.3 min (97:3 e.r.).

methyl (3*R*,4*R*)-4-(4-cyanophenyl)-5,5,5-trifluoro-2-methylene-3-(o-tolyl)pentanoate (3k)

***3k** was prepared following the general procedure described in section **E**.*

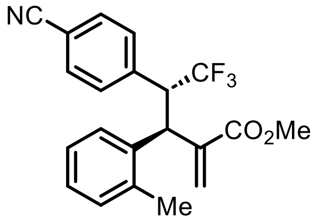

40% yield (30 mg, 0.08 mmol), colourless oil. **^{1}H NMR** (400 MHz, $CDCl_3$) δ 7.46 (d, *J* = 8.5 Hz, 2H), 7.32 (d, *J* = 8.3 Hz, 2H), 7.30 – 7.27 (m, 1H), 7.02 – 6.91 (m, 3H), 6.35 (s, 1H), 5.93 (s, 1H), 4.77 (d, *J* = 11.9 Hz, 1H), 4.68 (dq, *J* = 11.9, 8.1 Hz, 1H), 3.75 (s, 3H), 2.29 (s, 3H) ppm. **^{13}C NMR** (101 MHz, $CDCl_3$) δ 166.5, 139.9, 139.5, 136.3, 135.9, 132.2, 130.9, 130.1, 128.3, 128.2, 127.1, 126.6 (q, $^1J_{CF}$ = 281.4 Hz), 126.1, 118.4, 112.1, 52.7 (q, $^2J_{CF}$ = 25.2 Hz) 52.2, 43.8, 20.1 ppm; **^{19}F NMR** (376 MHz, $CDCl_3$) δ -64.6 (d, $^3J_{HF}$ = 8.1 Hz, 3F) ppm; **HRMS (ESI)** Calculated for $[C_{21}H_{19}O_2NF_3]^+$ ($[M+H]^+$) 374.1362. Found 374.1367; **$[\alpha]_D^{25}$** = +63.8 (c = 1.21, $CHCl_3$); **HPLC** Phenomenex Lux Cellulose-1 (99:1 *n*-Hexane:2-Propanol, 1 mL/min, 242 nm); t_R (major) = 18.2 min, t_R (minor) = 11.9 min (94:6 e.r.).

methyl (3*R*,4*R*)-4-(4-cyanophenyl)-5,5,5-trifluoro-2-methylene-3-(naphthalen-2-yl)pentanoate (3l)

***3l** was prepared following the general procedure described in section **E**.*

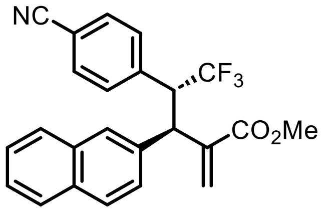

59% yield (48 mg, 0.13 mmol), yellow oil. **^{1}H NMR** (500 MHz, $CDCl_3$) δ 7.66 (ddt, *J* = 6.6, 5.0, 2.0 Hz, 2H), 7.58 (d, *J* = 9.0 Hz, 2H), 7.47 – 7.36 (m, 6H), 7.28 (dd, *J* = 8.6, 1.9 Hz, 1H), 6.33 (s, 1H), 6.04 (s, 1H), 4.86 (dq, *J* = 11.9, 8.3 Hz, 1H), 4.65 (d, *J* = 12.0 Hz, 1H), 3.74 (s, 3H) ppm; **^{13}C NMR** (126 MHz, $CDCl_3$) δ 166.4, 140.5, 139.6, 135.7, 133.2, 132.3, 132.3, 130.4, 128.3, 128.2, 127.8, 127.6, 127.6, 126.4, 126.2, 126.1 (q, $^1J_{CF}$ = 281.6 Hz), 118.3, 112.2, 52.2, 51.7 (q, $^2J_{CF}$ = 25.2 Hz), 49.8 ppm; **^{19}F NMR** (471 MHz, $CDCl_3$) δ -64.8 (d, $^3J_{HF}$ = 8.3 Hz, 3F) ppm; **HRMS (ESI)** Calculated for $[C_{24}H_{19}O_2NF_3]^+$ ($[M+H]^+$) 410.1368. Found 410.1365; **$[\alpha]_D^{25}$** = +16.5 (c = 1.21, $CHCl_3$); **HPLC** Phenomenex Lux Cellulose-1 (98:2 *n*-Hexane:2-Propanol, 1 mL/min, 254 nm); t_R (major) = 13.8 min, t_R (minor) = 14.8 min (99:1 e.r.).

ethyl (3*R*,4*R*)-4-(4-cyanophenyl)-5,5,5-trifluoro-2-methylene-3-phenylpentanoate (3m)

***3m** was prepared following the general procedure described in section **E**.*

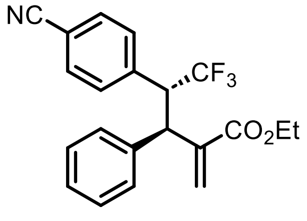

60% yield (45 mg, 0.12 mmol), colourless oil. **^{1}H NMR** (500 MHz, $CDCl_3$) δ 7.46 (d, *J* = 8.5 Hz, 2H), 7.34 (d, *J* = 8.2 Hz, 2H), 7.16 – 7.12 (m, 2H), 7.10 – 7.06 (m, 2H), 7.05 – 7.01 (m, 1H), 6.31 (s, 1H), 5.95 (s, 1H), 4.73 (dq, *J* = 12.0, 8.4 Hz, 1H), 4.45 (d, *J* = 12.0 Hz, 1H), 4.30 – 4.13 (m, 2H), 1.3 (t, *J* = 7.2 Hz, 3H) ppm; **^{13}C NMR** (126 MHz, $CDCl_3$) δ 165.9, 140.7, 139.8, 138.5, 132.2, 130.5, 128.9, 128.5, 127.2, 127.2, 126.3 (q, $^1J_{CF}$ = 281.5 Hz) 118.4, 112.0, 61.2, 51.8 (q, $^2J_{CF}$ = 25.5 Hz), 49.9, 14.2 ppm; **^{19}F NMR** (471 MHz, $CDCl_3$) δ -64.8 (d, $^3J_{HF}$ = 8.4 Hz, 3F) ppm; **HRMS (ESI)** Calculated for $[C_{21}H_{19}O_2NF_3]^+$ ($[M+H]^+$) 374.1362. Found 374.1360; **$[\alpha]_D^{25}$** = +34.5 (c = 2.26, $CHCl_3$); **HPLC** Phenomenex Lux Cellulose-1 (99:1 *n*-Hexane:2-Propanol, 1 mL/min, 240 nm); t_R (major) = 17.8 min, t_R (minor) = 11.7 min (94:6 e.r.).

methyl 4-((2*R*,3*R*)-1,1,1-trifluoro-4-(methoxycarbonyl)-3-phenylpent-4-en-2-yl)benzoate (3n)

***3n** was prepared following the general procedure described in section **E**.*

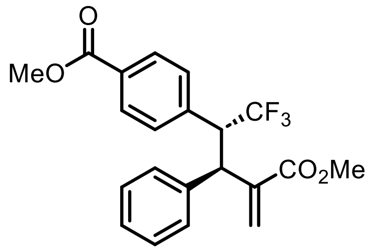

75% yield (59 mg, 0.15 mmol), white solid. **^{1}H NMR** (400 MHz, $CDCl_3$) δ 7.84 (d, *J* = 8.5 Hz, 2H), 7.30 (d, *J* = 8.3 Hz, 2H), 7.18 – 7.12 (m, 2H), 7.10 – 7.03 (m, 2H), 7.03 – 6.96 (m, 1H), 6.31 (s, 1H), 5.97 (s, 1H), 4.69 (dq, *J* = 11.9, 8.5 Hz, 1H), 4.50 (d, *J* = 11.9 Hz, 1H), 3.84 (s, 3H), 3.75 (s, 3H) ppm; **^{13}C NMR** (101 MHz, $CDCl_3$) δ 166.7, 166.4, 140.8, 139.4, 138.7, 129.7, 129.6, 128.9, 128.3, 127.3, 126.6 (q, $^1J_{CF}$ = 281.0 Hz), 126.9, 52.2, 52.1, 51.7 (q, $^2J_{CF}$ = 25.0 Hz), 49.8 ppm; **^{19}F NMR** (471 MHz, $CDCl_3$) δ -64.8 (d, $^3J_{HF}$ = 8.5 Hz, 3F) ppm; **HRMS (ESI)** Calculated for $[C_{21}H_{20}O_4F_3]^+$ ($[M+H]^+$) 393.1312. Found 393.13612; **$[\alpha]_D^{25}$** = +31.2 (c = 2.19, $CHCl_3$); **HPLC** Phenomenex Lux Cellulose-1 (98:2 *n*-Hexane:2-Propanol, 1 mL/min, 240 nm); t_R (major) = 14.3 min, t_R (minor) = 7.7 min (97:3 e.r.).

methyl (3*R*,4*R*)-4-(4-acetylphenyl)-5,5,5-trifluoro-2-methylene-3-phenylpentanoate (3o)

***3o** was prepared following the general procedure described in section **E**.*

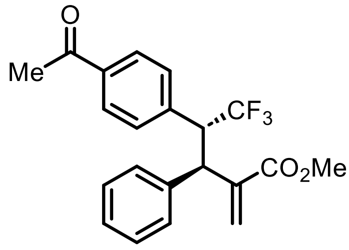

61% yield (46 mg, 0.12 mmol), yellow solid. **^{1}H NMR** (500 MHz, $CDCl_3$) δ 7.76 (d, *J* = 8.4 Hz, 2H), 7.33 (d, *J* = 8.1 Hz, 2H), 7.19 – 7.15 (m, 2H), 7.09 – 7.05 (m, 2H), 7.03 – 6.98 (m, 1H), 6.31 (s, 1H), 5.98 (s, 1H), 4.71 (dq, *J* = 11.9, 8.5 Hz, 1H), 4.51 (d, *J* = 12.0 Hz, 1H), 3.75 (s, 3H), 2.50 (s, 3H) ppm; **^{13}C NMR** (126 MHz, $CDCl_3$) δ 197.6, 166.4, 140.8, 139.6, 138.7, 136.6, 130.0, 129.0, 128.4, 127.3, 127.0, 126.5 (q, $^1J_{CF}$ = 281.4 Hz), 52.1, 51.7 (q, $^2J_{CF}$ = 25.2 Hz), 49.8, 26.7 ppm; **^{19}F NMR** (471 MHz, $CDCl_3$) δ -64.8 (d, $^3J_{HF}$ = 8.6 Hz, 3F) ppm; **HRMS (ESI)** Calculated for $[C_{21}H_{20}O_3F_3]^+$ ($[M+H]^+$) 377.1359. Found 377.1371; **$[\alpha]_D^{25}$** = +44.5 (c = 2.51, $CHCl_3$); **HPLC** Phenomenex Lux Cellulose-1 (95:5 *n*-Hexane:2-Propanol, 1 mL/min, 254 nm); t_R (major) = 11.0 min, t_R (minor) = 7.7 min (95:5 e.r.).

methyl (3*R*,4*R*)-4-(4-benzoylphenyl)-5,5,5-trifluoro-2-methylene-3-phenylpentanoate (3p)

***3p** was prepared following the general procedure described in section **E**.*

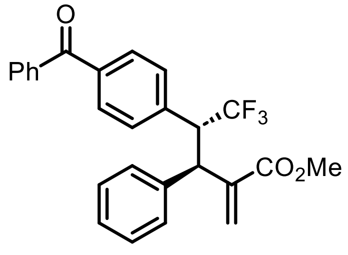

64% yield (56 mg, 0.13 mmol), colouress oil. **^{1}H NMR** (400 MHz, $CDCl_3$) δ 7.68 (dd, *J* = 8.3, 1.4 Hz, 2H), 7.62 (d, *J* = 8.3 Hz, 2H), 7.59 – 7.53 (m, 1H), 7.48 – 7.41 (m, 2H), 7.36 (d, *J* = 8.1 Hz, 2H), 7.21 – 7.16 (m, 2H), 7.09 (ddd, *J* = 7.5, 6.7, 1.3 Hz, 2H), 7.05 – 7.00 (m, 1H), 6.32 (s, 1H), 6.00 (s, 1H), 4.74 (dq, *J* = 11.9, 8.5 Hz, 1H), 4.54 (d, *J* = 11.9 Hz, 1H), 3.75 (s, 3H) ppm; **^{13}C NMR** (101 MHz, $CDCl_3$) δ 196.2, 166.4, 140.8, 138.9, 138.9, 138.7, 137.5, 137.0, 132.6, 130.1, 130.1, 129.7, 129.0, 128.4, 128.4, 127.3, 127.0 126.5 (q, $^1J_{CF}$ = 281.5 Hz), 52.1, 51.7 (q, $^2J_{CF}$ = 25.4 Hz), 49.8 ppm; **^{19}F NMR** (376 MHz, $CDCl_3$) δ -64.7 (d, $^3J_{HF}$ = 8.5 Hz, 3F) ppm; **HRMS (ESI)** Calculated for $[C_{26}H_{22}O_3F_3]^+$ ($[M+H]^+$) 439.1516. Found 439.1513; **$[\alpha]_D^{25}$** = +31.4 (c = 3.47, $CHCl_3$); **HPLC** Phenomenex Lux Cellulose-1 (95:5 *n*-Hexane:2-Propanol, 1 mL/min, 254 nm); t_R (major) = 11.9 min, t_R (minor) = 7.9 min (97:3 e.r.).

methyl (3*R*,4*R*)-5,5,5-trifluoro-4-(4-formylphenyl)-2-methylene-3-phenylpentanoate (3q)

***3q** was prepared following the general procedure described in section **E**.*

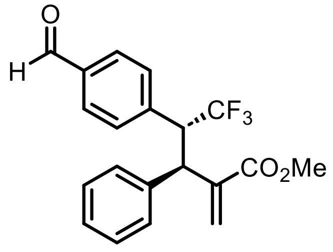

33% yield (24 mg, 0.07 mmol), colouress oil. **^{1}H NMR** (500 MHz, $CDCl_3$) δ 9.88 (s, 1H), 7.69 (d, J = 8.5 Hz, 2H), 7.41 (d, J = 8.1 Hz, 2H), 7.18 – 7.14 (m, 2H), 7.11 – 7.05 (m, 2H), 7.03 – 6.97 (m, 1H), 6.32 (s, 1H), 5.99 (d, J = 0.6 Hz, 1H), 4.74 (dq, J = 11.9, 8.4 Hz, 1H), 4.52 (d, J = 12.0 Hz, 1H), 3.75 (s, 3H) ppm; **^{13}C NMR** (126 MHz, $CDCl_3$) δ 191.8, 166.4, 141.1, 140.7, 138.6, 135.8, 130.4, 129.7, 129.0, 128.4, 127.4, 127.1, 126.4 (q, $^1J_{CF}$ = 281.2 Hz), 52.1, 51.9 (q, $^2J_{CF}$ = 25.3 Hz), 49.9 ppm; **^{19}F NMR** (471 MHz, $CDCl_3$) δ -64.7 (d, $^3J_{HF}$ = 8.4 Hz, 3F) ppm; **HRMS (ESI)** Calculated for $[C_{20}H_{18}O_3F_3]^+$ ($[M+H]^+$) 363.1203. Found 363.1194; **$[\alpha]_D^{25}$** = +37.8 (c = 1.73, $CHCl_3$); **HPLC** Phenomenex Lux Cellulose-1 (98:2 *n*-Hexane:2-Propanol, 1 mL/min, 256 nm); t_R (major) = 11.4 min, t_R (minor) = 19.4 min (96:4 e.r.).

methyl (3*R*,4*R*)-5,5,5-trifluoro-2-methylene-3-phenyl-4-(4-(phenylsulfonyl)phenyl) pentanoate (3r)

***3r** was prepared following the general procedure described in section **E**.*

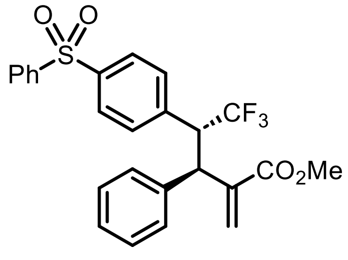

56% yield (53 mg, 0.11 mmol), white solid. **^{1}H NMR** (500 MHz, $CDCl_3$) δ 7.83 (dd, J = 8.5, 1.3 Hz, 2H), 7.73 (d, J = 8.5 Hz, 2H), 7.58 – 7.53 (m, 1H), 7.50 – 7.45 (m, 2H), 7.35 (d, J = 8.5 Hz, 2H), 7.11 – 7.07 (m, 2H), 7.06 – 7.01 (m, 2H), 7.01 – 6.96 (m, 1H), 6.30 (s, 1H), 5.95 (s, 1H), 4.69 (dq, J = 11.9, 8.4 Hz, 1H), 4.43 (d, J = 11.9 Hz, 1H), 3.74 (s, 3H) ppm; **^{13}C NMR** (126 MHz, $CDCl_3$) δ 166.4, 141.4, 141.0, 140.5, 140.1, 138.3, 133.4, 130.6, 129.4, 128.9, 128.5, 127.8, 127.7, 127.4, 127.2, 126.4 (q, $^1J_{CF}$ = 281.7 Hz), 52.2, 51.7 (q, $^2J_{CF}$ = 25.3 Hz), 49.9 ppm; **^{19}F NMR** (471 MHz, $CDCl_3$) δ -64.7 (d, $^3J_{HF}$ = 8.4 Hz, 3F) ppm; **HRMS (ESI)** Calculated for $[C_{25}H_{22}O_4F_3S]^+$ ($[M+H]^+$) 475.1185. Found 475.1186; **$[\alpha]_D^{25}$** = +10.1 (c = 2.45, $CHCl_3$); **HPLC** Phenomenex Lux Cellulose-1 (90:10 *n*-Hexane:2-Propanol, 1 mL/min, 240 nm); t_R (major) = 18.3 min, t_R (minor) = 14.9 min (99:1 e.r.).

methyl (3*R*,4*R*)-4-(4-(*N*,*N*-dimethylsulfamoyl)phenyl)-5,5,5-trifluoro-2-methylene-3-phenylpentanoate (3s)

***3s** was prepared following the general procedure described in section **E**.*

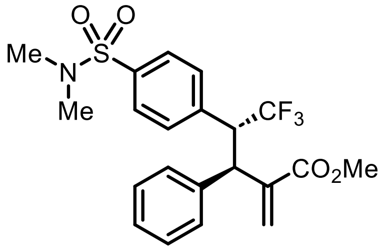

50% yield (44 mg, 0.10 mmol), yellow oil. **^{1}H NMR** (400 MHz, $CDCl_3$) δ 7.55 (d, *J* = 8.4 Hz, 2H), 7.38 (d, *J* = 8.3 Hz, 2H), 7.14 – 7.09 (m, 2H), 7.05 (ddd, *J* = 8.1, 7.0, 1.0 Hz, 2H), 7.01 – 6.96 (m, 1H), 6.33 (s, 1H), 5.99 (s, 1H), 4.71 (dq, *J* = 11.9, 8.4 Hz, 1H), 4.47 (d, *J* = 11.9 Hz, 1H), 3.76 (s, 3H), 2.55 (s, 6H) ppm; **^{13}C NMR** (101 MHz, $CDCl_3$) δ 166.4, 140.5, 139.5, 138.6, 134.7, 130.4, 129.0, 128.4, 127.7, 127.4, 127.0, 126.4 (q, $^1J_{CF}$ = 281.5 Hz), 52.2, 51.7 (q, $^2J_{CF}$ = 25.4 Hz), 50.0, 37.9 ppm; **^{19}F NMR** (471 MHz, $CDCl_3$) δ -64.7 (d, $^3J_{HF}$ = 8.5 Hz, 3F) ppm; **HRMS (ESI)** Calculated for $[C_{21}H_{23}O_4NF_3S]^+$ ($[M+H]^+$) 442.1294. Found 442.1306; **$[\alpha]_D^{25}$** = +18.0 (c = 2.28, $CHCl_3$); **HPLC** Chiralpak Daicel IC (80:20 *n*-Hexane:2-Propanol, 1 mL/min, 205 nm); t_R (major) = 24.2 min, t_R (minor) = 26.2 min (99:1 e.r.).

methyl (3*R*,4*R*)-5,5,5-trifluoro-2-methylene-4-(4-nitrophenyl)-3-phenylpentanoate (3t)

***3t** was prepared following the general procedure described in section **E**.*

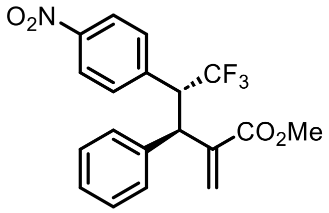

48% yield (36 mg, 0.10 mmol), yellow oil. **^{1}H NMR** (500 MHz, $CDCl_3$) δ 8.03 (d, *J* = 8.8 Hz, 2H), 7.41 (d, *J* = 8.8 Hz, 2H), 7.18 – 7.13 (m, 2H), 7.09 (ddd, *J* = 7.7, 6.8, 1.3 Hz, 2H), 7.05 – 7.00 (m, 1H), 6.32 (s, 1H), 5.99 (s, 1H), 4.81 (dq, *J* = 12.0, 8.4 Hz, 1H), 4.48 (d, *J* = 12.0 Hz, 1H), 3.76 (s, 3H) ppm; **^{13}C NMR** (126 MHz, $CDCl_3$) δ 166.4, 147.5, 141.7, 140.4, 138.3, 130.7, 128.9, 128.6, 127.6, 127.3, 126.3 (q, $^1J_{CF}$ = 281.7 Hz), 123.6, 52.2, 51.5 (q, $^2J_{CF}$ = 25.3 Hz), 50.1 ppm; **^{19}F NMR** (471 MHz, $CDCl_3$) δ -64.8 (d, $^3J_{HF}$ = 8.4 Hz, 3F) ppm; **HRMS (ESI)** Calculated for $[C_{19}H_{17}O_4NF_3]^+$ ($[M+H]^+$) 380.1104. Found 380.1113; **$[\alpha]_D^{25}$** = +32.3 (c = 0.09, $CHCl_3$); **HPLC** Phenomenex Lux Cellulose-1 (98:2 *n*-Hexane:2-Propanol, 1 mL/min, 240 nm); t_R (major) = 13.8 min, t_R (minor) = 9.1 min (92:8 e.r.).

methyl (3*R*,4*R*)-5,5,5-trifluoro-2-methylene-4-(2-nitrophenyl)-3-phenylpentanoate (3u)

***3u** was prepared following the general procedure described in section **E**.*

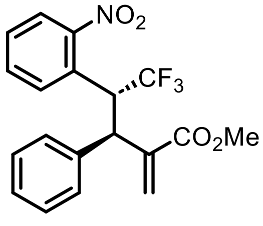

82% yield (62 mg, 0.16 mmol), yellow oil. **^{1}H NMR** (500 MHz, $CDCl_3$) δ 7.89 (dd, *J* = 8.2, 1.4 Hz, 1H), 7.68 (dt, *J* = 8.0, 1.1 Hz, 1H), 7.61 (td, *J* = 7.6, 1.4 Hz, 1H), 7.47 (ddd, *J* = 8.1, 7.3, 1.4 Hz, 1H), 7.46 – 7.42 (m, 2H), 7.37 – 7.32 (m, 2H), 7.30 – 7.25 (m, 1H), 6.08 (s, 1H), 5.83 (d, *J* = 0.7 Hz, 1H), 5.32 (dq, *J* = 12.2, 7.9 Hz, 1H), 4.85 (d, *J* = 12.2 Hz, 1H), 3.60 (s, 3H) ppm; **^{13}C NMR** (126 MHz, $CDCl_3$) δ 166.5, 151.1, 140.7, 138.9, 133.2, 129.6, 129.3, 129.0, 128.8, 128.6, 127.7, 126.7, 125.9 (q, $^1J_{CF}$ = 281.7 Hz), 125.2, 52.3, 46.7, 46.2 (q, $^2J_{CF}$ = 25.8 Hz) ppm; **^{19}F NMR** (471 MHz, $CDCl_3$) δ -64.4 (d, $^3J_{HF}$ = 7.8 Hz, 3F) ppm; **HRMS (ESI)** Calculated for $[C_{19}H_{17}O_4NF_3]^+$ ($[M+H]^+$) 380.1104. Found 380.1115; **$[\alpha]_D^{25}$** = -117.9 (c = 3.47, $CHCl_3$); **HPLC** Phenomenex Lux Cellulose-1 (98:2 *n*-Hexane:2-Propanol, 1 mL/min, 240 nm); t_R (major) = 10.7 min, t_R (minor) = 19.0 min (96:4 e.r.).

methyl (3*R*,4*R*)-4-(2-cyanophenyl)-5,5,5-trifluoro-2-methylene-3-phenylpentanoate (3v)

***3v** was prepared following the general procedure described in section **E**.*

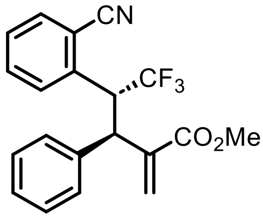

52% yield (37 mg, 0.10 mmol), yellow oil. **^{1}H NMR** (500 MHz, $CDCl_3$) δ 7.74 – 7.58 (m, 3H), 7.46 (d, *J* = 7.3 Hz, 2H), 7.43 (td, *J* = 7.6, 1.5 Hz, 1H), 7.39 – 7.33 (m, 2H), 7.32 – 7.27 (m, 1H), 6.14 (s, 1H), 5.80 (s, 1H), 4.86 (d, *J* = 12.2 Hz, 1H), 4.69 (dq, *J* = 12.2, 7.7 Hz, 1H), 3.60 (s, 3H) ppm; **^{13}C NMR** (126 MHz, CDCl3) δ 166.3, 143.3, 140.5, 138.7, 138.2, 133.4, 133.2, 128.9, 128.8, 128.7, 128.6, 128.5, 127.8, 126.4, 124.6, 124.6 (q, $^1J_{CF}$ = 282.2 Hz), 115.3, 52.3, 49.8 (q, $^2J_{CF}$ = 25.6 Hz), 46.4 ppm; **^{19}F NMR** (471 MHz, $CDCl_3$) δ -64.6 (d, $^3J_{HF}$ = 7.8 Hz, 3F) ppm; **HRMS (ESI)** Calculated for $[C_{20}H_{17}O_2NF_3]^+$ ($[M+H]^+$) 360.1206. Found 360.1213; **$[\alpha]_D^{25}$** = -128.0 (c = 2.86, $CHCl_3$); **HPLC** Phenomenex Lux Cellulose-2 (98:2 *n*-Hexane:2-Propanol, 1 mL/min, 240 nm); t_R (major) = 10.5 min, t_R (minor) = 7.3 min (95:5 e.r.).

methyl 4-(6-bromopyridin-2-yl)-5,5,5-trifluoro-2-methylene-3-phenylpentanoate

***3w** was prepared following the procedure described in section **E**.*

52% yield (43 mg, 0.10 mmol) of a 1:1 diastereomeric ratio.

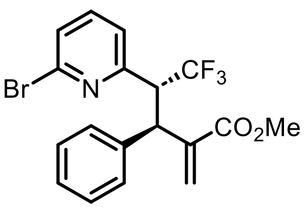

Diastereomer (3*R*,4*R*)-3w, white solid. **^{1}H NMR** (400 MHz, $CDCl_3$) δ 7.39 – 7.33 (m, 1H), 7.29 – 7.23 (m, 3H), 7.21 – 7.14 (m, 3H), 7.1 – 7.09 (m, 1H), 5.93 (s, 1H), 5.56 (s, 1H), 4.65 (d, J = 12.5 Hz, 1H), 4.32 (dqd, J = 12.5, 7.9, 1.5 Hz, 1H), 3.45 (s, 3H) ppm; **^{13}C NMR** (101 MHz, $CDCl_3$) δ 166.6, 156.1, 141.6, 140.3, 139.1, 138.9, 128.7, 128.7, 127.7, 127.5, 127.1, 125.7 (q, $^1J_{CF}$ = 282.3 Hz), 122.7, 54.8 (q, $^2J_{CF}$ = 25.1 Hz), 52.2, 47.0 ppm; **^{19}F NMR** (376 MHz, $CDCl_3$) δ -63.7 (d, $^3J_{HF}$ = 8.0 Hz, 3F) ppm; **HRMS (ESI)** Calculated for $[C_{18}H_{16}O_2NBrF_3]^+$ ($[M+H]^+$) 414.0311. Found 414.0311; **$[\alpha]_D^{25}$** = -94.6 (c = 1.05, $CHCl_3$); **HPLC** Phenomenex Lux Cellulose-1 (98:2 *n*-Hexane:2-Propanol, 1 mL/min, 240 nm); t_R (major) = 9.1 min, t_R (minor) = 8.7 min (89:11 e.r.).

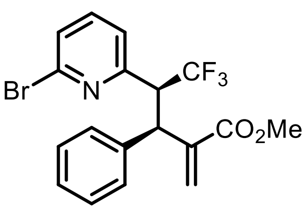

Diastereomer (3*R*,4*S*)-3w', white solid. **^{1}H NMR** (400 MHz, $CDCl_3$) δ 7.31 (d, J = 7.7 Hz, 1H), 7.24 – 7.19 (m, 3H), 7.10 (m, 3H), 7.04 – 7.00 (m, 1H), 6.32 (s, 1H), 6.04 (s, 1H), 4.82 (dq, J = 11.7, 8.0 Hz, 1H), 4.73 (d, J = 11.7 Hz, 1H), 3.74 (s, 3H) ppm; **^{13}C NMR** (101 MHz, $CDCl_3$) δ 166.5, 155.4, 141.4, 140.6, 139.1, 138.8, 138.5, 129.0, 128.7, 128.3, 127.6, 127.3, 126.9, 125.7 (q, $^1J_{CF}$ = 282.3 Hz) 124.0, 53.1 (q, $^2J_{CF}$ = 25.0 Hz), 52.1, 48.6 ppm; **^{19}F NMR** (376 MHz, $CDCl_3$) δ -64.6 (d, $^3J_{HF}$ = 7.9 Hz, 3F) ppm; **HRMS (ESI)** Calculated for $[C_{18}H_{16}O_2NBrF_3]^+$ ($[M+H]^+$) 414.0311. Found 414.0311; **$[\alpha]_D^{25}$** = +21.0 (c = 0.90, $CHCl_3$); **HPLC** Phenomenex Lux Cellulose-1 (98:2 *n*-Hexane:2-Propanol, 1 mL/min, 240 nm); t_R (major) = 8.4 min, t_R (minor) = 6.0 min (99:1 e.r.).

methyl (3*R*,4*R*)-4-(2-chloropyridin-3-yl)-5,5,5-trifluoro-2-methylene-3-phenylpentanoate (3x)

***3x** was prepared following the general procedure described in section **E**.*

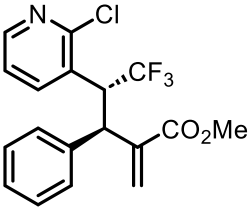

65% yield (48 mg, 0.13 mmol), colouress oil. **^{1}H NMR** (500 MHz, $CDCl_3$) δ 8.36 (dd, J = 4.7, 1.8 Hz, 1H), 7.86 (dq, J = 7.8, 1.0 Hz, 1H), 7.46 – 7.40 (m, 2H), 7.40 – 7.33 (m, 2H), 7.30 – 7.26 (m, 2H), 6.13 (s, 1H), 5.79 (s, 1H), 4.89 – 4.74 (m, 2H), 3.60 (s, 3H) ppm; **^{13}C NMR** (126 MHz, $CDCl_3$) δ 166.4, 152.7, 149.4, 140.5, 138.6, 137.8, 129.9, 128.8, 128.6, 127.8, 126.0, 125.8 (q, $^1J_{CF}$ = 282.3 Hz) 123.1, 52.4, 48.7 (q, $^2J_{CF}$ = 25.6 Hz), 46.1 ppm; **^{19}F NMR** (471 MHz, $CDCl_3$) δ -64.9 (d, $^3J_{HF}$ = 6.6 Hz, 3F) ppm; **HRMS (ESI)** Calculated for $[C_{18}H_{16}O_2NClF_3]^+$ ($[M+H]^+$) 370.0816. Found 370.0823; **$[\alpha]_D^{25}$** = -140.6 (c = 0.45, $CHCl_3$); **HPLC** Phenomenex Lux Cellulose-1 (98:2 *n*-Hexane:2-Propanol, 1 mL/min, 240 nm); t_R (major) = 14.5 min, t_R (minor) = 11.4 min (96:4 e.r.).

F. Optimisation of the α-Selective and diastereoselective Cross-Electrophile Catalytic Coupling between allyl fluorides (1) and *gem*-difluoroalkenes (2)

*F.1. **Optimisation** – Solvent screening*

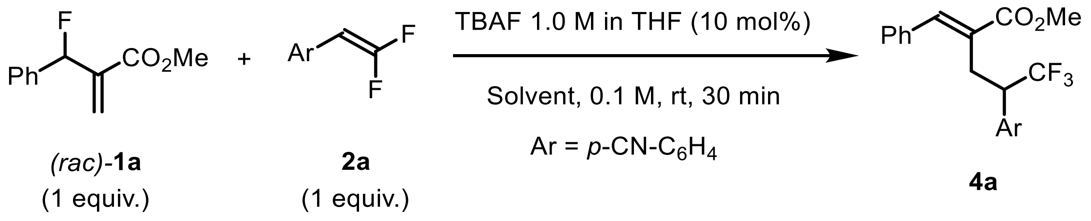

Entry	Solvent	Conv. (%)[a]	4a (%)[a]	(*E*/*Z*)[a]
**1**	Toluene	42	<5	>20:1
**2**	CH_2Cl_2	65	<5	>20:1
**3**	THF	>99	85	>20:1
**4**	MeCN	>99	75	>20:1
**5**	DMSO	>99	88	>20:1
**6**	DMF	>99	92	>20:1

Table S10. Optimisation of the solvent. [a] Determined by ^{1}H NMR spectroscopy using 1,3,5-trimethoxybenzene as the internal standard. All the entries were performed using 4Å MS. TBAF: Tetrabutylammonium fluoride, THF: Tetrahydrofuran, DMSO: Dimethylsulfoxide, DMF: *N*,*N*-dimethylformamide. n.d. = not detected.

*F.2. **Optimisation** – Screening of the fluoride source*

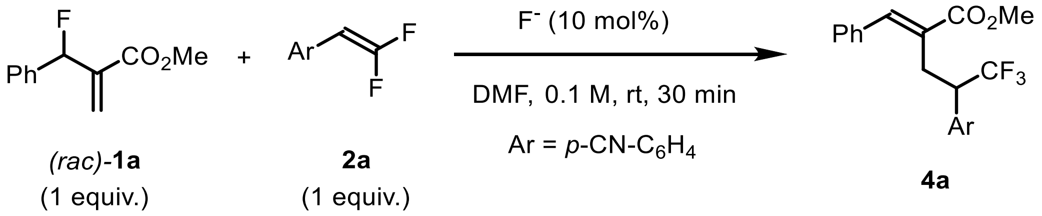

Entry	Fluoride source	Conv. (%)[a]	4a (%)[a]	(*E*/*Z*)[a]
**1**	TBAF	>99	92	>20:1
**2**	CsF	55	30	>20:1

Table S11. Optimisation of the fluoride source. [a] Determined by ^{1}H NMR spectroscopy using 1,3,5-trimethoxybenzene as the internal standard. All the entries were performed using 4Å MS. TBAF: Tetrabutylammonium fluoride, DMF: *N*,*N*-dimethylformamide.

F.3. ***Optimisation*** *– Ratio of reagents and catalyst loading*

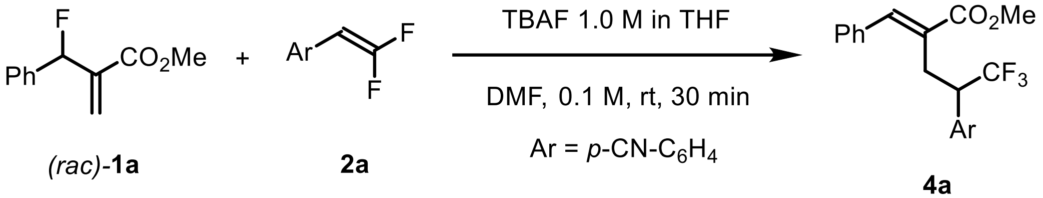

(rac)-**1a** **2a** **4a**

Entry	Cat. loading (mol%)	Ratio of reagents (1a:2a)	Conv. (%)[a]	4a (%)[a]	(*E*/*Z*)[a]
**1**	10	1:1	>99	92	>20:1
**2**	5	1:1	>99	80	>20:1
**3**	2.5	1:1	90	65	>20:1
**4**	1	1:1	60	33	>20:1
**5**	5	1:1.5	>99	99 (96)[b]	>20:1
**6**	2.5	1:1.5	80	72	>20:1

Table S12. Optimisation of the ratio of reagents and catalyst loading. [a] Determined by ^{1}H NMR spectroscopy using 1,3,5-trimethoxybenzene as the internal standard. [b] Isolated yield. All the entries were performed using 4Å MS. TBAF: Tetrabutylammonium fluoride, DMF: *N*,*N*-dimethylformamide.

***F.4. Optimisation** – Attempt to develop the α-selective asymmetric cross-electrophile catalytic coupling between allyl fluorides (1) and gem-difluoroalkenes (2)*

In order to develop an asymmetric verion of the α-selective XEC protocol, we devised the following three potential catalytic strategies:

- First, the use of a chiral ammonium fluoride as catalyst as catalysts. Such salts can be prepared via anion-exchange from the corresponding bromide salts using fluoride-loaded resins.[5a-d] However, tetraalkylammonium fluoride are highly hygroscopic, and removal of the hydration sphere that stabilises the fluoride anion leads to decomposition via Hofmann elimination due to the strong basicity of the "naked" fluoride.[5e,f] Given their cumbersome preparation, high hygroscopicity and intrinsic instability under anhydrous conditions, we ruled out this approach.
- Second, the *in situ* generation of chiral ammonium fluorides from the easy-to-handle ammonium bromides and metal fluorides.[5g] Reported methods, however, requires the inorganic fluoride source to be used in large excess with respect to the chiral ammonium bromide – typically 25- to 250-fold excess. Such conditions would in principle conflict with our fundamental catalytic strategy, which relies on using catalytic amounts of fluoride to trigger the α-selective XEC process.
- Third, the use of chiral bisurea catalyst together with a fluoride source under hydrogen-bonding phase-transfer (HB-PTC) conditions, as developed by Prof. Gouverneur and co-workers.[1d,5h] Although the initial fluoride addition in our system is not a stereodefining step, we speculated that the chiral bisurea catalyst could engage *via* hydrogen-bonding with the resulting α-trifluoromethyl carbanion $\mathbf{9}^-$ and hence govern its addition to the ammonium intermediate $\mathbf{8}^+$.

Guided by these precedents and consideration, we performed the following set of experiments:

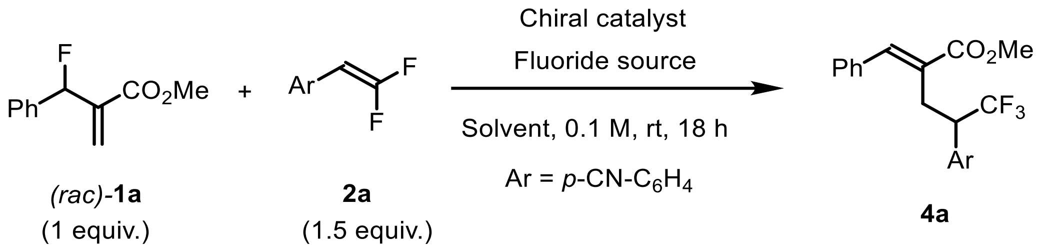

Entry	Catalyst (mol%)	Fluoride source (mol%)	Solvent	Conv. (%)[a]	4a (%)[a]	e.r.[b]
**1**	**5k** (20)	CsF (20)	CH_2Cl_2	-	-	-
**2**	**5k** (20)	CsF (20)	$PhCF_3$	-	-	-
**3**	**5m** (20)	CsF (20)	Toluene	-	-	-
**4**	**5k** (20)	CsF (20)	1,2-DFB	-	-	-
**5**	**5k** (20)	CsF (20)	DMF	60	30	50:50
**6**	**5k** (20)	CsF (20)	DMSO	56	24	50:50
**7**	**5l** (10)	CsF (20)	CH_2Cl_2	-	-	-
**8**	**5l** (10)	CsF (20)	1,2-DFB	-	-	-
**9**	**5l** (10)	TBAF (10)	CH_2Cl_2	-	-	-
**10**	**5l** (10)	TBAF (10)	1,2-DFB	-	-	-
**11**	**5l** (10)	TBAF (10)	DMF	38	19	50:50
**12**	**5l** (10)	TBAF (10)	DMSO	29	traces	-
**13**	**5l** (10)	CsF (20)	DMF	59	30	50:50
**14**	**5l** (10)	CsF (20)	DMSO	43	traces	-

Table S13. Optimisation of the α-selective asymmetric cross-electrophile coupling. [a] Determined by ^{1}H NMR spectroscopy using 1,3,5-trimethoxybenzene as the internal standard. [b] Determined by HPLC analysis using a chiral column. All the entries were performed using 4Å MS. TBAF: Tetrabutylammonium fluoride, 1,2-DFB: 1,2-difluorobenzene; DMF: *N*,*N*-dimethylformamide; DMSO: dimethylsulfoxide.

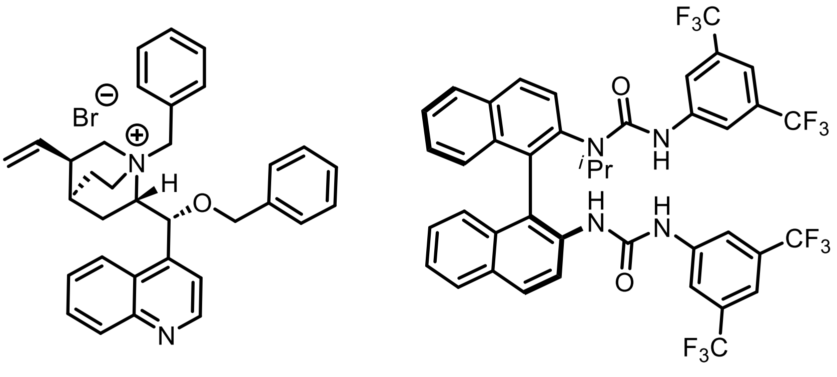

5k (*S*)-**5l**

- The reaction using 20 mol% benzylated cinchonidine bromide **5k** with 20 mol% CsF was tested in apolar solvents (CH_2Cl_2, $PhCF_3$, toluene, and 1,2-difluorobenzene; entries 1-4), which are commonly employed in phase-transfer catalysis to ensure efficient ion pairing.

  [As shown, none of these solvents provided any conversion within 18 h, consistent with our experimental results and computed energies, where the transition state of the RDS lies at 32.1 kcal/mol in toluene]

- The reaction using 20 mol% benzylated cinchonidine bromide **5k** with 20 mol% CsF in polar aprotic solvents such as DMF or DMSO gave **4a** in low yields (24-30%) but without enantiocontrol (entries 5-6).

  [This suggests that a background reaction occurs –as the computed RDS barrier in DMF is 15.2 kcal/mol– and/or that ion pairing between ***5k*** *and* ***9***$^{-}$ *is disrupted in these solvents]*

- The reaction using 10 mol% of the bisurea (*S*)-**5l** in the two optimal apolar solvents reported by Prof. Gouverneur (CH_2Cl_2 and 1,2-difluorobenzene), together with 10 mol% of either CsF or TBAF, did not produce **4a** after 18 h (entries 7-10).

  [Simlar than before, this result is consistent with the kinetic infesability of this transformation in apolar solvents]

- On the other hand, while DMSO consumed **1a** without generating the expected product (entries 12 and 14), DMF afforded **4a** in low yield and as a racemate (entries 11 and 13).

  *[These results suggest that, besides the potential operation of a background reaction, the use of polar solvents might preclude hydrogen-bonding interactions between the chiral catalyst (S)-**5l** and the α-trifluoromethyl carbanion* ***9***$^{-}$*, preventing stereocontrol over the product's absolute configuration.]*

G. α-Selective and diastereoselective Cross-Electrophile Catalytic Coupling between allyl fluorides (1) and gem-difluoroalkenes (2)

General Procedure

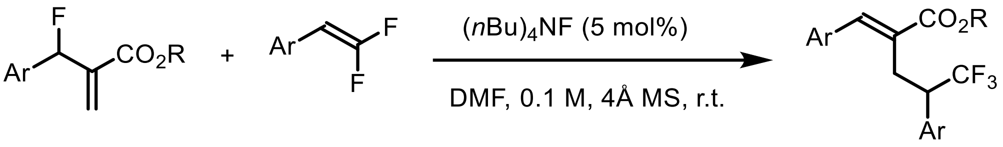

(*rac*)-**1** allyl fluoride (1.0 equiv.)

2 *gem*-difluoroalkene (3.0 equiv.)

4

The corresponding allyl fluoride **1** (1 equiv., 0.2 mmol) was weighted into a 5 mL vial equipped with a magnetic stirring bar, 75 mg of 4Å MS, and dissolved with 2 mL of dry DMF (0.1 M). Subsequently, the corresponding *gem*-difluoroalkene **2** (1.5 equiv., 0.3 mmol) and 5 mol% of $(n\text{Bu})_4\text{NF}$ **8a** 1.0 in THF were added sequentially. The reaction mixture was stirred at room temperature until full consumption of starting material **1**. The reaction mixture was washed with water and brine, dried over anhydrous $MgSO_4$, and the resulting solution was concentrated under reduced pressure. Product **4** was purified by flash column chromatography on silica gel using mixtures of *n*-hexane/CH_2Cl_2 as eluent.

methyl (*E*)-2-benzylidene-4-(4-cyanophenyl)-5,5,5-trifluoropentanoate (4a)

***4a** was prepared following the general procedure described in section **G**.*

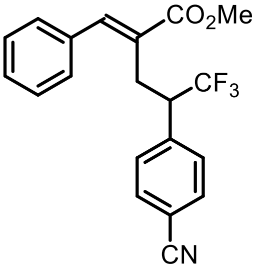

96% yield (69 mg, 0.19 mmol), yellowish solid. **^{1}H NMR** (400 MHz, $CDCl_3$) δ 7.67 (s, 1H), 7.41 (d, J = 8.4 Hz, 2H), 7.36 (dd, J = 5.1, 1.9 Hz, 2H), 7.08 – 7.01 (m, 4H), 3.85 – 3.70 (m, 4H), 3.28 (dd, J = 13.9, 10.5 Hz, 1H), 3.18 (ddd, J = 13.8, 4.8, 1.2 Hz, 1H) ppm; **^{13}C NMR** (126 MHz, $CDCl_3$) δ 167.8, 143.0, 139.2, 134.9, 132.1, 130.0, 128.9, 128.8, 128.6, 128.0, 126.2 (q, $^1J_{CF}$ = 280.7 Hz), 118.5, 112.2, 52.4, 48.8 (q, $^2J_{CF}$ = 26.7 Hz), 26.5 ppm; **^{19}F NMR** (471 MHz, $CDCl_3$) δ -69.2 (d, $^3J_{HF}$ = 9.0 Hz, 3F) ppm; **HRMS (ESI)** Calculated for $[C_{20}H_{17}O_2NF_3]^+$ ($[M+H]^+$) 360.1206. Found 360.1197.

methyl (*E*)-2-(4-bromobenzylidene)-4-(4-cyanophenyl)-5,5,5-trifluoropentanoate (4b)

***4b** was prepared following the general procedure described in section **G**.*

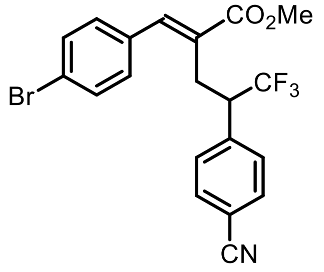

88% yield (77 mg, 0.18 mmol), yellowish solid. ^{1}**H NMR** (400 MHz, $CDCl_3$) δ 7.57 (s, 1H), 7.50 (d, *J* = 8.4 Hz, 2H), 7.47 – 7.42 (m, 2H), 7.07 (d, *J* = 8.3 Hz, 2H), 6.94 – 6.89 (m, 2H), 3.85 – 3.71 (m, 4H), 3.23 – 3.12 (m, 2H) ppm; ^{13}**C NMR** (101 MHz, $CDCl_3$) δ 167.3, 141.5, 139.0, 133.6, 132.1, 131.9, 130.1, 129.8, 128.6, 126.0 (q, $^{1}J_{CF}$ = 280.5 Hz), 123.0, 118.3, 112.3, 52.4, 48.6 (q, $^{2}J_{CF}$ = 27.1 Hz), 26.6 (q, $^{3}J_{CF}$ = 2.6 Hz) ppm; ^{19}**F NMR** (471 MHz, $CDCl_3$) δ -69.1 (d, $^{3}J_{HF}$ = 9.0 Hz, 3F) ppm; **HRMS (ESI)** Calculated for $[C_{20}H_{16}O_2NBrF_3]^+$ ($[M+H]^+$) 438.0311. Found 438.0306.

methyl (*E*)-4-(4-cyanophenyl)-5,5,5-trifluoro-2-(4-methylbenzylidene)pentanoate (4c)

***4c** was prepared following the general procedure described in section **G**.*

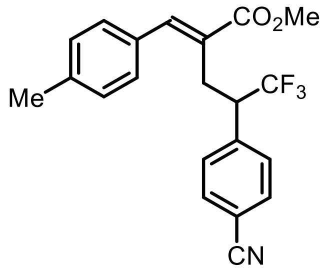

85% yield (63 mg, 0.17 mmol), yellowish solid. ^{1}**H NMR** (400 MHz, $CDCl_3$) δ 7.63 (s, 1H), 7.42 (d, *J* = 8.3 Hz, 2H), 7.18 (d, *J* = 7.8 Hz, 2H), 7.07 (d, *J* = 8.1 Hz, 2H), 6.99 (d, *J* = 7.9 Hz, 2H), 3.80 (ddd, *J* = 10.5, 8.9, 4.8 Hz, 1H), 3.75 (s, 3H), 3.31 (dd, *J* = 14.0, 10.7 Hz, 1H), 3.19 (ddd, *J* = 14.0, 4.8, 1.2 Hz, 1H), 2.40 (s, 3H) ppm; ^{13}**C NMR** (101 MHz, $CDCl_3$) δ 167.9, 143.0, 139.2, 139.2, 132.0, 132.0, 130.0, 129.5, 128.8, 127.1, 126.3 (q, $^{1}J_{CF}$ = 280.6 Hz), 118.5, 112.2, 52.3, 48.7 (q, $^{2}J_{CF}$ = 26.7 Hz), 26.4 (q, $^{3}J_{CF}$ = 2.5 Hz), 21.5 ppm; ^{19}**F NMR** (376 MHz, $CDCl_3$) δ -69.2 (d, $^{3}J_{HF}$ = 9.1 Hz, 3F) ppm; **HRMS (ESI)** Calculated for $[C_{21}H_{19}O_2NF_3]^+$ ($[M+H]^+$) 374.1362. Found 374.1362.

methyl (*E*)-4-(4-cyanophenyl)-5,5,5-trifluoro-2-(3-methylbenzylidene)pentanoate (4d)

***4d** was prepared following the general procedure described in section **G**.*

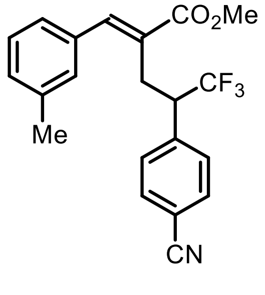

78% yield (58 mg, 0.16 mmol), yellowish solid. ^{1}**H NMR** (400 MHz, $CDCl_3$) δ 7.63 (s, 1H), 7.42 (d, *J* = 8.0 Hz, 2H), 7.26 (t, *J* = 7.6 Hz, 1H), 7.16 (d, *J* = 7.6 Hz, 1H), 7.06 (d, *J* = 7.9 Hz, 2H), 6.89 (d, *J* = 7.5 Hz, 1H), 6.80 (s, 1H), 3.79 – 3.73 (s, 4H), 3.27 (dd, *J* = 13.9, 10.6 Hz, 1H), 3.23 – 3.11 (m, 1H), 2.36 (s, 3H) ppm; ^{13}**C NMR** (101 MHz, $CDCl_3$) δ 167.8, 143.1, 139.2, 138.5, 134.8, 132.0, 130.0, 129.6, 129.2, 128.6, 127.7, 126.2 (q, $^{1}J_{CF}$ = 280.8 Hz), 125.6, 118.4, 112.0, 52.3, 48.8 (q, $^{2}J_{CF}$ = 27.0 Hz), 26.5 (q, $^{3}J_{CF}$ = 2.5 Hz), 21.5 ppm; ^{19}**F NMR** (376 MHz, $CDCl_3$) δ -69.1 (d, $^{3}J_{HF}$ = 9.0 Hz, 3F) ppm; **HRMS (ESI)** Calculated for $[C_{21}H_{19}O_2NF_3]^+$ ($[M+H]^+$) 374.1362. Found 374.1365.

methyl (*E*)-4-(4-cyanophenyl)-5,5,5-trifluoro-2-(2-methylbenzylidene)pentanoate (4e)

***4e** was prepared following the general procedure described in section **G**.*

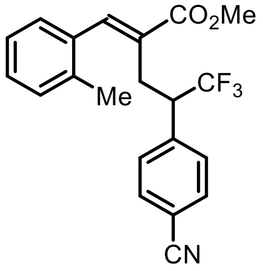

83% yield (62 mg, 0.17 mmol), yellowish solid. **^{1}H NMR** (500 MHz, $CDCl_3$) δ 7.67 (s, 1H), 7.39 (d, *J* = 8.4 Hz, 2H), 7.30 – 7.23 (m, 1H), 7.20 (td, *J* = 7.5, 1.4 Hz, 1H), 7.16 – 7.13 (m, 1H), 6.98 (dd, *J* = 8.3, 6.7 Hz, 3H), 3.83 – 3.77 (s, 4H), 3.18 (dd, *J* = 13.8, 10.9 Hz, 1H), 3.08 (ddd, *J* = 13.9, 4.4, 1.2 Hz, 1H), 1.80 (s, 3H) ppm; **^{13}C NMR** (126 MHz, $CDCl_3$) δ 167.6, 142.8, 139.2, 136.8, 134.1, 132.1, 130.2, 129.9, 128.9, 128.2, 128.1, 126.1 (q, $^1J_{CF}$ = 280.3 Hz), 125.9, 118.4, 112.2, 52.4, 48.8 (q, $^2J_{CF}$ = 26.9 Hz), 26.3 (q, $^3J_{CF}$ = 2.7 Hz), 19.6 ppm; **^{19}F NMR** (471 MHz, $CDCl_3$) δ -69.5 (d, $^3J_{HF}$ = 9.0 Hz, 3F) ppm; **HRMS (ESI)** Calculated for $[C_{21}H_{19}O_2NF_3]^+$ ($[M+H]^+$) 374.1362. Found 374.1365.

methyl (*E*)-4-(1,1,1-trifluoro-4-(methoxycarbonyl)-5-phenylpent-4-en-2-yl)benzoate (4f)

***4f** was prepared following the general procedure described in section **G**.*

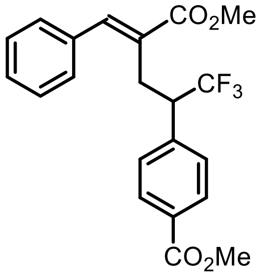

90% yield (71 mg, 0.18 mmol), colourless oil. **^{1}H NMR** (500 MHz, $CDCl_3$) δ 7.82 (d, *J* = 8.4 Hz, 2H), 7.65 (s, 1H), 7.36 – 7.30 (m, 2H), 7.08 – 7.01 (m, 4H), 3.89 (s, 3H), 3.86 – 3.76 (m, 1H), 3.74 (s, 3H), 3.26 (dd, *J* = 14.0, 10.4 Hz, 1H), 3.19 (ddd, *J* = 14.0, 5.0, 1.2 Hz, 1H) ppm; **^{13}C NMR** (126 MHz, $CDCl_3$) δ 167.8, 166.7, 142.7, 139.0, 135.0, 130.0, 129.6, 129.2, 128.7, 128.6, 128.4, 126.5 (q, $^1J_{CF}$ = 280.5 Hz), 52.2, 48.6 (q, $^2J_{CF}$ = 26.6 Hz), 26.7 (q, $^3J_{CF}$ = 3.0 Hz) ppm; **^{19}F NMR** (471 MHz, $CDCl_3$) δ -69.2 (d, $^3J_{HF}$ = 9.2 Hz, 3F) ppm; **HRMS (ESI)** Calculated for $[C_{21}H_{20}O_4F_3]^+$ ($[M+H]^+$) 393.1312. Found 393.13619.

methyl (*E*)-2-benzylidene-5,5,5-trifluoro-4-(4-nitrophenyl)pentanoate (4g)

***4g** was prepared following the general procedure described in section **G**.*

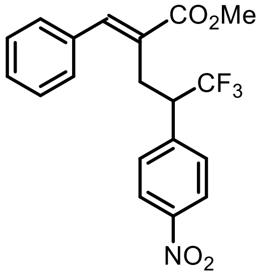

35% yield (27 mg, 0.07 mmol), yellow oil. **^{1}H NMR** (500 MHz, $CDCl_3$) δ 7.96 (d, *J* = 8.7 Hz, 2H), 7.67 (s, 1H), 7.35 (dd, *J* = 5.0, 2.0 Hz, 3H), 7.12 – 7.08 (m, 2H), 7.06 – 7.02 (m, 2H), 3.92 – 3.80 (m, 1H), 3.78 (s, 3H), 3.31 (dd, *J* = 14.0, 10.7 Hz, 1H), 3.21 (ddd, *J* = 14.0, 4.7, 1.2 Hz, 1H) ppm; **^{13}C NMR** (126 MHz, $CDCl_3$) δ 167.7, 147.8, 143.1, 141.1, 134.9, 130.1, 128.9, 128.8, 128.6, 127.9, 126.1 (q, $^1J_{CF}$ = 280.8 Hz), 123.4, 52.4, 48.6 (q, $^2J_{CF}$ = 26.9 Hz), 26.6 (q, $^3J_{CF}$ = 3.0 Hz) ppm; **^{19}F NMR** (471 MHz, $CDCl_3$) δ -69.2 (d, $^3J_{HF}$ = 9.0 Hz, 3F) ppm; **HRMS (ESI)** Calculated for $[C_{19}H_{17}O_4NF_3]^+$ ($[M+H]^+$) 380.1104. Found 380.1102.

methyl (*E*)-2-benzylidene-5,5,5-trifluoro-4-(4-(phenylsulfonyl)phenyl)pentanoate (4h)

***4h** was prepared following the general procedure described in section **G**.*

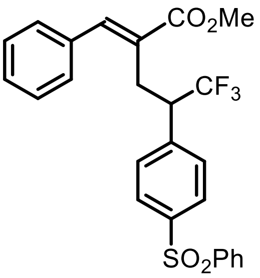

78% yield (74 mg, 0.16 mmol), yellow oil. **^{1}H NMR** (400 MHz, $CDCl_3$) δ 7.97 – 7.91 (m, 2H), 7.69 (d, *J* = 8.4 Hz, 2H), 7.63 (s, 1H), 7.62 – 7.57 (m, 1H), 7.57 – 7.49 (m, 2H), 7.33 – 7.22 (m, 3H), 7.06 (d, *J* = 8.4 Hz, 2H), 6.97 – 6.92 (m, 2H), 3.82 – 3.71 (s, 4H), 3.24 (dd, *J* = 13.8, 10.5 Hz, 1H), 3.16 (ddd, *J* = 13.9, 4.8, 1.1 Hz, 1H) ppm; **^{13}C NMR** (101 MHz, $CDCl_3$) δ 167.7, 143.0, 141.4, 141.4, 139.6, 134.8, 133.5, 130.0, 129.4, 128.7, 128.5, 128.0, 127.8, 127.7, 124.8, 123.4 (q, $^1J_{CF}$ = 280.5 Hz), 52.3, 48.7 (q, $^2J_{CF}$ = 26.9 Hz), 26.7 (q, $^3J_{CF}$ = 2.6 Hz) ppm; **^{19}F NMR** (471 MHz, $CDCl_3$) δ -69.1 (d, $^3J_{HF}$ = 9.2 Hz, 3F) ppm **HRMS (ESI)** Calculated for $[C_{25}H_{21}O_4F_3NaS]^+$ ($[M+Na]^+$) 497.1005. Found 497.0997.

methyl (*E*)-2-benzylidene-5,5,5-trifluoro-4-(4-formylphenyl)pentanoate (4i)

***4i** was prepared following the general procedure described in section **G**.*

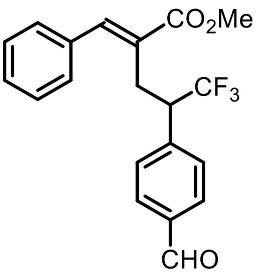

59% yield (43 mg, 0.12 mmol), yellow oil. **^{1}H NMR** (400 MHz, $CDCl_3$) δ 9.96 (s, 1H), 7.70 – 7.61 (m, 3H), 7.38 – 7.32 (m, 3H), 7.12 (d, *J* = 8.0 Hz, 2H), 7.06 (ddt, *J* = 5.6, 2.2, 0.8 Hz, 1H), 3.88 – 3.77 (m, 1H), 3.77 (s, 3H), 3.30 (dd, *J* = 14.0, 10.4 Hz, 1H), 3.20 (ddd, *J* = 14.0, 5.0, 1.2 Hz, 1H) ppm; **^{13}C NMR** (101 MHz, $CDCl_3$) δ 191.8, 167.8, 142.8, 140.7, 136.1, 135.0, 129.9, 129.7, 128.8, 128.7, 128.7, 128.3, 126.4 (q, $^1J_{CF}$ = 280.4 Hz), 52.3, 48.8 (q, $^2J_{CF}$ = 26.7 Hz), 26.7 (q, $^3J_{CF}$ = 2.6 Hz) ppm; **^{19}F NMR** (471 MHz, $CDCl_3$) δ -69.1 (d, $^3J_{HF}$ = 9.2 Hz, 3F) ppm; **HRMS (ESI)** Calculated for $[C_{20}H_{18}O_3F_3]^+$ ($[M+H]^+$) 363.1203. Found 363.1193.

methyl (*E*)-2-benzylidene-5,5,5-trifluoro-4-(4-(trifluoromethyl)phenyl)pentanoate (4j)

***4j** was prepared following the general procedure described in section **G**.*

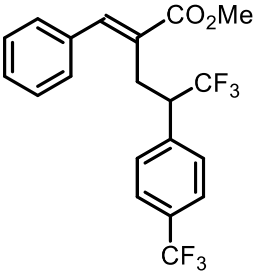

81% yield (65 mg, 0.16 mmol), yellow oil. **^{1}H NMR** (500 MHz, $CDCl_3$) δ 7.68 (s, 1H), 7.40 – 7.37 (m, 2H), 7.35 – 7.31 (m, 3H), 7.07 (d, *J* = 8.1 Hz, 2H), 7.04 – 7.00 (m, 2H), 3.78 (s, 4H), 3.28 (dd, *J* = 13.9, 10.5 Hz, 1H), 3.20 (ddd, *J* = 13.9, 4.7, 1.2 Hz, 1H) ppm; **^{13}C NMR** (126 MHz, $CDCl_3$) δ 167.9, 142.9, 138.0, 135.0, 130.4 (q, $^2J_{CF}$ = 32.5 Hz), 129.5, 128.7, 128.7, 128.7 (q, $^1J_{CF}$ = 281.3 Hz), 128.6, 128.3, 128.2, 128.1, 125.7 (q, $^3J_{CF}$ = 4.0 Hz), 125.3 (q, $^3J_{CF}$ = 3.9 Hz), 124.1 (q, $^1J_{CF}$ = 272.4 Hz), 52.3, 48.7 (q, $^2J_{CF}$ = 26.8 Hz), 26.8 (q, $^3J_{CF}$ = 2.9 Hz) ppm; **^{19}F NMR** (471 MHz, $CDCl_3$) δ -62.8 (s, 3F), -69.2 (d, $^3J_{HF}$ = 9.1 Hz, 3F) ppm; **HRMS (ESI)** Calculated for $[C_{20}H_{17}O_2F_6]^+$ ($[M+H]^+$) 403.1127. Found 403.1131.

methyl (*E*)-2-benzylidene-4-(2-cyanophenyl)-5,5,5-trifluoropentanoate (4k)

***4k** was prepared following the general procedure described in section **G**.*

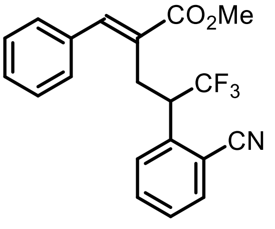

30% yield (22 mg, 0.06 mmol), yellow oil. **^{1}H NMR** (400 MHz, $CDCl_3$) δ 7.72 (s, 1H), 7.59 (ddd, *J* = 7.8, 1.5, 0.6 Hz, 1H), 7.36 – 7.28 (m, 4H), 7.26 – 7.21 (m, 1H), 7.02 (tdd, *J* = 7.1, 3.5, 1.3 Hz, 3H), 4.32 (dtd, *J* = 15.0, 8.9, 4.5 Hz, 1H), 3.85 (s, 3H), 3.31 (dd, *J* = 13.8, 10.3 Hz, 1H), 3.24 (ddd, *J* = 13.9, 4.9, 1.2 Hz, 1H) ppm; **^{13}C NMR** (101 MHz, $CDCl_3$) δ 167.6, 143.3, 137.5, 135.0, 132.9, 132.7, 128.7, 128.7, 128.6, 128.0, 126.1 (q, $^1J_{CF}$ = 281.0 Hz), 117.2, 114.7, 52.6, 46.5 (q, $^2J_{CF}$ = 27.2 Hz), 26.7 (q, $^3J_{CF}$ = 2.7 Hz) ppm; **^{19}F NMR** (471 MHz, $CDCl_3$) δ -69.0 (d, $^3J_{HF}$ = 9.0 Hz, 3F) ppm; **HRMS (ESI)** Calculated for $[C_{20}H_{16}O_2NF_3Na]^+$ ($[M+Na]^+$) 382.1025. Found 382.1018.

methyl (*E*)-2-(1,1,1-trifluoro-4-(methoxycarbonyl)-5-phenylpent-4-en-2-yl)benzoate (4l)

***4l** was prepared following the general procedure described in section **G**.*

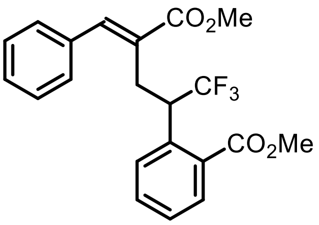

35% yield (27 mg, 0.07 mmol), yellow oil. **^{1}H NMR** (400 MHz, $CDCl_3$) δ 7.78 (ddd, *J* = 7.9, 1.6, 0.5 Hz, 1H), 7.62 (s, 1H), 7.32 – 7.27 (m, 3H), 7.25 – 7.22 (m, 1H), 7.15 (td, *J* = 7.6, 1.5 Hz, 1H), 7.06 (dt, *J* = 7.9, 1.3 Hz, 1H), 7.02 – 6.96 (m, 2H), 5.18 (ddq, *J* = 14.8, 9.7, 4.8 Hz, 1H), 3.82 (s, 3H), 3.80 (s, 3H), 3.30 – 3.15 (m, 2H) ppm; **^{13}C NMR** (101 MHz, $CDCl_3$) δ 168.0, 167.8, 142.1, 135.3, 134.9, 131.7, 130.5, 129.0, 128.8, 128.5, 128.2, 127.7, 126.9 (q, $^1J_{CF}$ = 280.8 Hz) 52.4, 52.2, 42.2 (q, $^2J_{CF}$ = 26.6 Hz), 27.2 (q, $^3J_{CF}$ = 2.8 Hz) ppm; **^{19}F NMR** (471 MHz, $CDCl_3$) δ -68.4 (d, $^3J_{HF}$ = 9.6 Hz, 3F) ppm; **HRMS (ESI)** Calculated for $[C_{21}H_{19}O_4F_3Na]^+$ ($[M+Na]^+$) 415.1128. Found 415.1121.

methyl (*E*)-2-benzylidene-4-(6-bromopyridin-2-yl)-5,5,5-trifluoropentanoate (4m)

***4m** was prepared following the general procedure described in section **G**.*

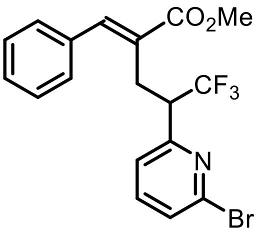

76% yield (63 mg, 0.15 mmol), colourless oil. **^{1}H NMR** (500 MHz, $CDCl_3$) δ 7.68 (s, 1H), 7.39 – 7.29 (m, 5H), 7.22 – 7.19 (m, 2H), 6.96 (p, *J* = 4.4 Hz, 1H), 3.93 (dqd, *J* = 10.4, 8.9, 4.5 Hz, 1H), 3.80 (s, 3H), 3.42 (dd, *J* = 14.0, 10.4 Hz, 1H), 3.18 (ddd, *J* = 14.0, 4.6, 1.2 Hz, 1H) ppm; **^{13}C NMR** (126 MHz, $CDCl_3$) δ 168.0, 155.3, 142.6, 141.6, 138.6, 134.9, 128.9, 128.8, 128.7, 128.2, 127.6, 126.1 (q, $^1J_{CF}$ = 280.7 Hz), 122.8, 52.3, 50.5 (q, $^2J_{CF}$ = 26.5 Hz), 26.2 (q, $^3J_{CF}$ = 2.5 Hz) ppm; **^{19}F NMR** (471 MHz, $CDCl_3$) δ -68.6 (d, $^3J_{HF}$ = 9.0 Hz, 3F) ppm; **HRMS (ESI)** Calculated for $[C_{18}H_{16}O_2NBrF_3]^+$ ($[M+H]^+$) 414.0311. Found 414.0303.

methyl (*E*)-2-benzylidene-5,5,5-trifluoro-4-(3-nitrophenyl)pentanoate (4n)

***4n** was prepared following the general procedure described in section **G**.*

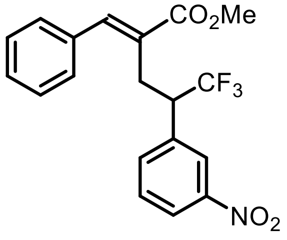

80% yield (61 mg, 0.16 mmol), colourless oil. **^{1}H NMR** (400 MHz, $CDCl_3$) δ 8.06 (ddd, J = 8.0, 2.3, 1.3 Hz, 1H), 7.80 (t, J = 2.1 Hz, 1H), 7.67 (s, 1H), 7.37 – 7.22 (m, 5H), 7.05 – 6.99 (m, 2H), 3.90 – 2.74 (m, 4H), 3.34 (dd, J = 13.9, 10.8 Hz, 1H), 3.21 (ddd, J = 14.0, 4.5, 1.2 Hz, 1H) ppm; **^{13}C NMR** (101 MHz, $CDCl_3$) δ 167.7, 148.1, 143.1, 135.9, 135.5, 134.8, 129.3, 128.9, 128.8, 128.4, 127.9, 126.2 (q, $^1J_{CF}$ = 280.5 Hz), 124.8, 124.0, 123.3, 52.4, 48.5 (q, $^2J_{CF}$ = 27.1 Hz), 26.5 (q, $^3J_{CF}$ = 2.7 Hz) ppm; **^{19}F NMR** (471 MHz, $CDCl_3$) δ -69.5 (d, $^3J_{HF}$ = 8.9 Hz, 3F) ppm; **HRMS (ESI)** Calculated for $[C_{19}H_{17}O_4NF_3]^+$ ($[M+H]^+$) 380.1080. Found 380.1090.

methyl (*E*)-2-benzylidene-4-(3,5-bis(trifluoromethyl)phenyl)-5,5,5-trifluoropentanoate (4o)

***4o** was prepared following the general procedure described in section **G**.*

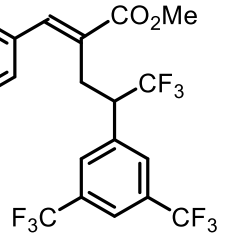

76% yield (71 mg, 0.15 mmol), orange solid. **^{1}H NMR** (400 MHz, $CDCl_3$) δ 7.75 (dt, J = 1.9, 1.0 Hz, 1H), 7.69 (s, 1H), 7.43 – 7.40 (m, 2H), 7.35 (dd, J = 5.1, 1.9 Hz, 3H), 7.09 – 7.04 (m, 2H), 4.01 – 3.83 (m, 1H), 3.78 (s, 3H), 3.32 (dd, J = 14.1, 10.5 Hz, 1H), 3.23 (ddd, J = 14.2, 5.0, 1.3 Hz, 1H) ppm; **^{13}C NMR** (101 MHz, $CDCl_3$) δ 167.7, 143.3, 136.6, 134.6, 131.9 (q, $^2J_{CF}$ = 33.6 Hz), 129.3, 129.1, 128.9, 128.5, 127.6, 126.1 (q, $^1J_{CF}$ = 280.5 Hz), 123.1 (q, $^1J_{CF}$ = 272.8 Hz), 122.5 (p, $^3J_{CF}$ = 3.9 Hz), 52.4, 48.5 (q, $^2J_{CF}$ = 27.3 Hz), 26.6 (q, $^3J_{CF}$ = 2.7 Hz) ppm; **^{19}F NMR** (471 MHz, $CDCl_3$) δ -62.9 (s, 6F), -69.4 (d, $^3J_{HF}$ = 9.0 Hz, 3F) ppm; **HRMS (ESI)** Calculated for $[C_{21}H_{16}O_2F_9]^+$ ($[M+H]^+$) 471.1001. Found 471.0986.

methyl (*E*)-2-benzylidene-4-(4-cyanophenyl)-5,5,5-trifluoro-4-methylpentanoate (4p)

***4p** was prepared following the general procedure described in section **G**.*

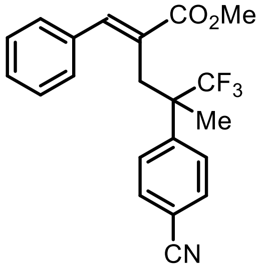

61% yield (46 mg, 0.12 mmol), colourless oil. **^{1}H NMR** (400 MHz, $CDCl_3$) δ 7.62 (s, 1H), 7.38 (d, J = 8.8 Hz, 2H), 7.35 – 7.29 (m, 5H), 7.06 – 7.02 (m, 2H), 3.61 (s, 3H), 3.48 (d, J = 13.8 Hz, 1H), 3.39 (dd, J = 13.7, 0.5 Hz, 1H), 1.38 (s, 3H) ppm; **^{13}C NMR** (101 MHz, $CDCl_3$) δ 169.0, 142.9, 142.6, 135.4, 131.5, 128.8, 128.7, 128.5, 128.4, 128.2, 127.8 (q, $^1J_{CF}$ = 284.0 Hz), 118.6, 111.7, 52.2, 49.3 (q, $^2J_{CF}$ = 23.9 Hz), 30.8 (q, $^3J_{CF}$ = 2.5 Hz), 17.6 (q, $^3J_{CF}$ = 3.0 Hz) ppm; **^{19}F NMR** (376 MHz, $CDCl_3$) δ -73.2 (s, 3F) ppm; **HRMS (ESI)** Calculated for $[C_{21}H_{19}O_2NF_3]^+$ ($[M+H]^+$) 374.1362. Found 374.1373.

dimethyl 4-benzylidene-2-phenyl-2-(trifluoromethyl)pentanedioate

***4q** was prepared following the procedure described in section **G**.*

90% yield (71 mg, 0.18 mmol) of a 1.3:1 = (*E*/*Z*).

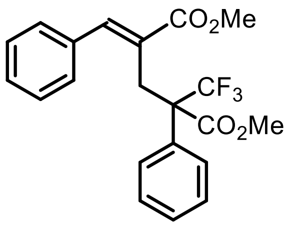

Diastereomer (*E*)-4q, yellowish solid. **^{1}H NMR** (400 MHz, $CDCl_3$) δ 7.68 (s, 1H), 7.38 – 7.27 (m, 3H), 7.25 – 7.17 (m, 5H), 7.13 – 7.07 (m, 2H), 3.86 (dd, J = 14.5, 0.7 Hz, 1H), 3.74 (d, J = 14.5 Hz, 1H), 3.68 (s, 3H), 3.56 (s, 3H) ppm; **^{13}C NMR** (101 MHz, $CDCl_3$) δ 169.2, 168.9, 142.4, 135.4, 134.0, 129.4, 128.8, 128.7, 128.6, 128.5, 128.4, 128.3, 128.3, 126.6, 123.7, 125.2 (q, $^1J_{CF}$ = 284.5 Hz), 61.1 (q, $^2J_{CF}$ = 24.3 Hz), 52.7, 52.2, 29.0 (q, $^3J_{CF}$ = 1.6 Hz) ppm; **^{19}F NMR** (376 MHz, $CDCl_3$) δ -67.2 (s, 3F) ppm; **HRMS (ESI)** Calculated for $[C_{21}H_{20}O_4F_3]^+$ ($[M+H]^+$) 393.1308. Found 393.1311.

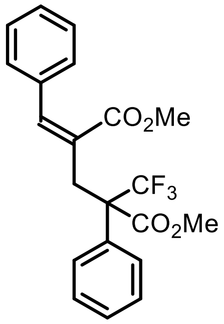

Diastereomer (*Z*)-4q', yellowish solid. **^{1}H NMR** (400 MHz, $CDCl_3$) δ 7.42 – 7.36 (m, 3H), 7.34 – 7.27 (m, 3H), 7.24 (dd, J = 5.5, 1.4 Hz, 2H), 7.11 – 7.04 (m, 2H), 6.56 (s, 1H), 3.83 (s, 3H), 3.63 (dd, J = 14.2, 0.9 Hz, 1H), 3.50 (s, 3H), 3.30 (dt, J = 14.2, 0.9 Hz, 1H) ppm; **^{13}C NMR** (101 MHz, $CDCl_3$) δ 169.3, 168.9, 140.1, 135.8, 134.4, 128.7, 128.7, 128.3, 128.2, 128.1, 128.0, 125.3 (q, $^1J_{CF}$ = 284.4 Hz), 62.1 (q, $^2J_{CF}$ = 24.3 Hz), 53.0, 51.7, 38.4 ppm; **^{19}F NMR** (376 MHz, $CDCl_3$) δ -66.2 (s, 3F) ppm; **HRMS (ESI)** Calculated for $[C_{21}H_{20}O_4F_3]^+$ ($[M+H]^+$) 393.1308. Found 393.1310.

Determination of the stereochemistry of 4b by NMR spectroscopy

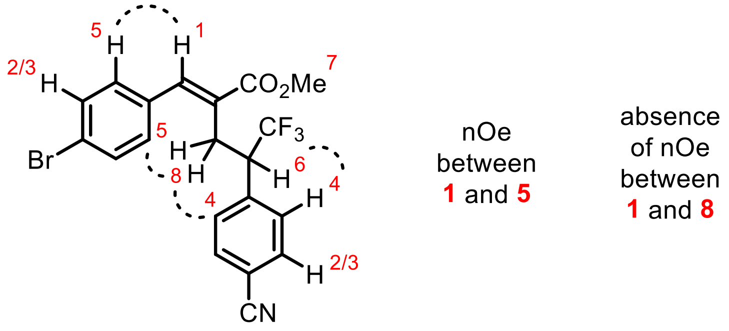

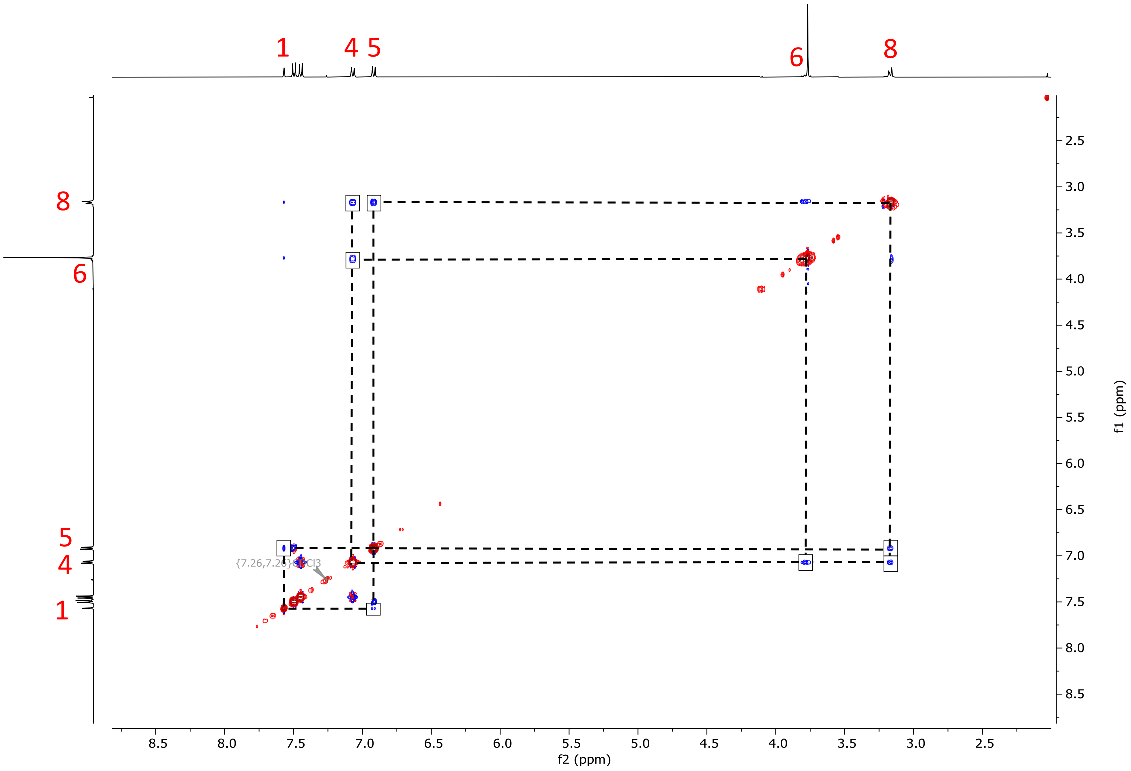

Figure S2. 2D NOESY spectrum of **4b** at 400 MHz in $CDCl_3$.

*[The nOe contact between protons **1** and **5** and the absence of nOe between protons **1** and **8** indicate that **4b** is the trans-(E)-stereoisomer.]*

Determination of the stereochemistry of 4q by NMR spectroscopy

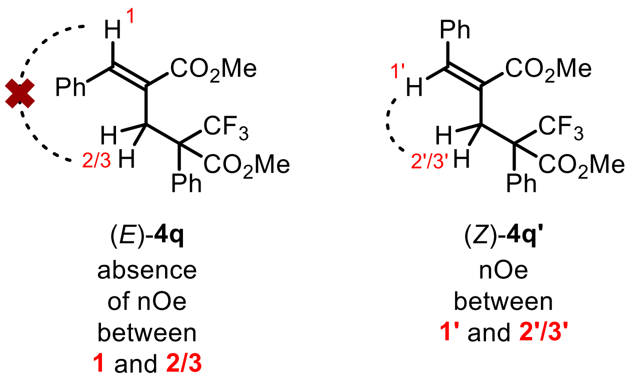

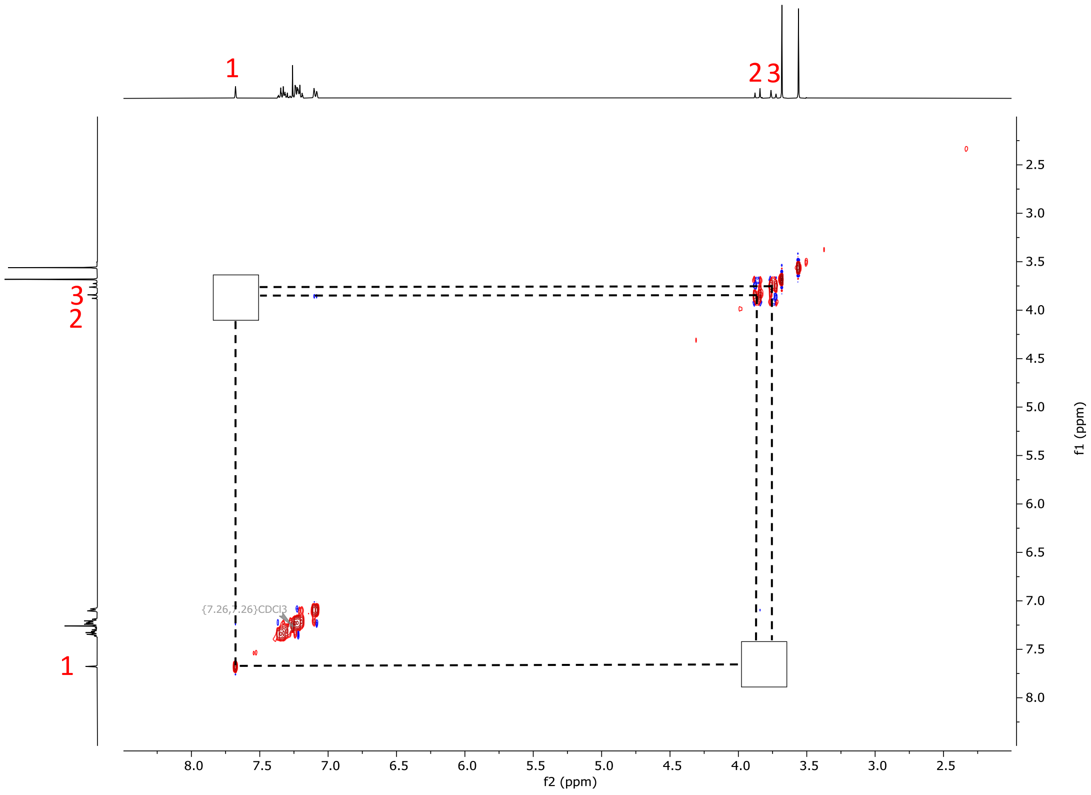

Figure S3. 2D NOESY spectrum of **4q** at 400 MHz in $CDCl_3$.

*[The absence of nOe contact between protons **1** and **2/3** indicate that **4q** is the trans-(E)-stereoisomer.]*

Determination of the stereochemistry of 4q’ by NMR spectroscopy

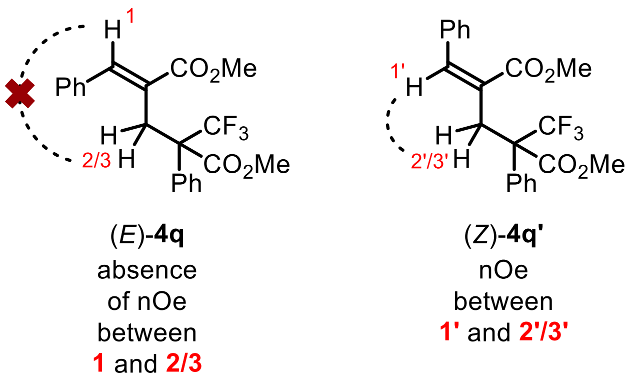

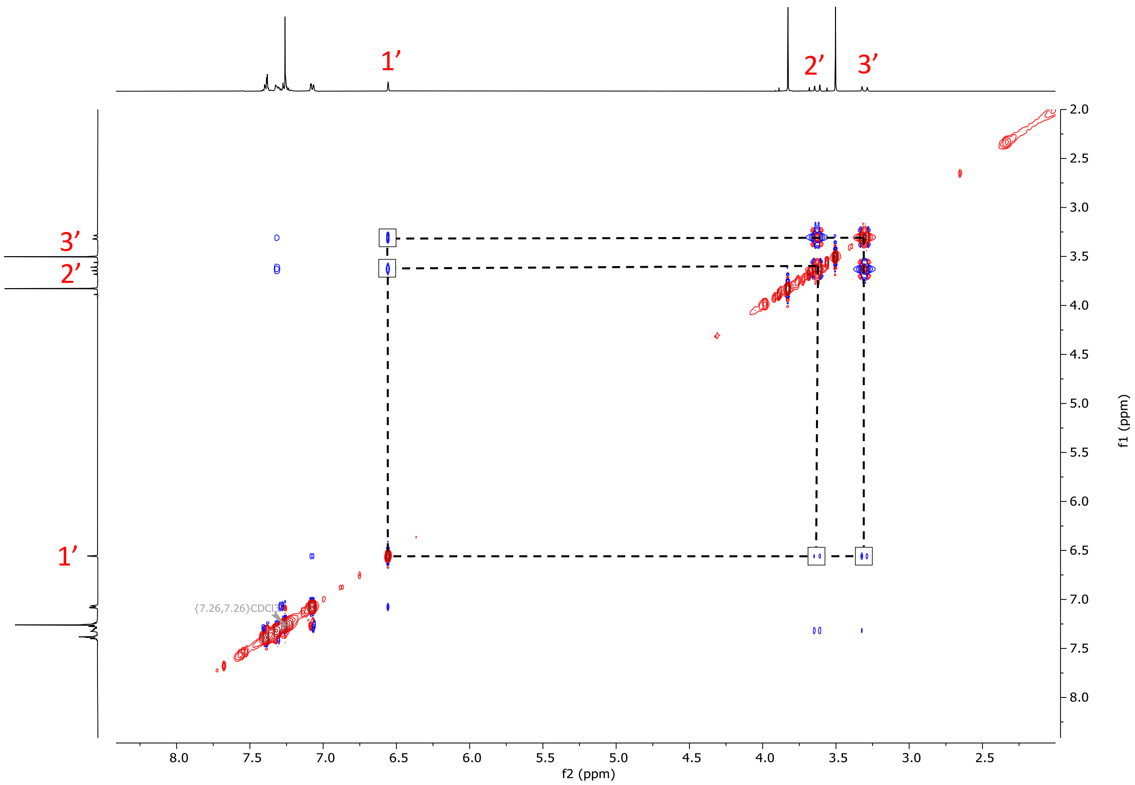

Figure S4. 2D NOESY spectrum of **4q’** at 400 MHz in $CDCl_3$.

*[The nOe contact between protons **1’** and **2’/3’** indicate that **4q’** is the cis-(Z)-stereoisomer.]*

H. Unsuccessful substrates

H.1. Aliphatic allyl fluorides

Aliphatic allyl fluorides **1n** and **1o** were evaluated under both racemic and asymmetric γ-selective catalytic conditions.

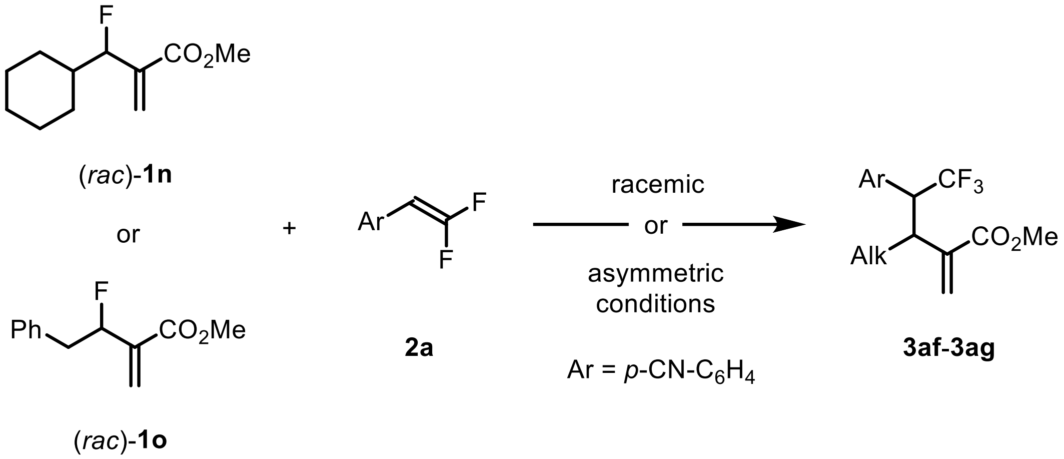

Entry	Optimised conditions	1	Conv. (%)[a]	NMR yield (%)[a]
1	DABCO **5g** in THF (1h)	**1n**	47	-
2	DABCO **5g** in THF (1h)	**1o**	>99	-
3	$(DHQD)_2AQN$ **5c** in $PhCF_3$ (18h)	**1n**	0	-
4	$(DHQD)_2AQN$ **5c** in $PhCF_3$ (18h)	**1o**	0	-

Table S14. Evaluation of the aliphatic allyl fluorides under racemic and asymmetric γ-selective conditions. [a] Determined by ^1H NMR spectroscopy using 1,3,5-trimethoxybenzene as the internal standard.

- Under racemic conditions, the product **3af** is not formed (entry 1). After 1h, allyl fluoride **1n** in partially consumed, along with the corresponding proportion of *gem*-difluoroalkene **2a**. While any by-product arising from **1n** could be identified in the reaction mixture, the protonated α-trifluoromethyl carbanion **9**-H was detected as the main by-product originating from **2a**.

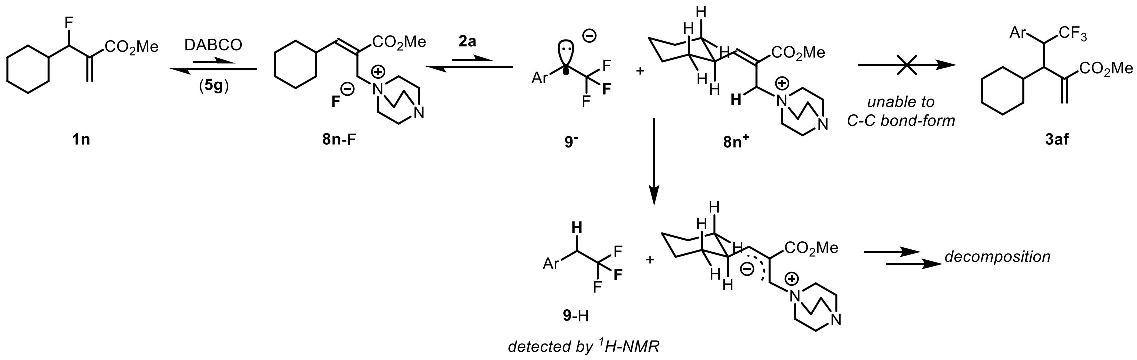

Figure S5. Proposed mechanistic for the reaction outcome between **1n** and **2a** under racemic conditions.

*[This observation suggests that DABCO is able to activate **1n**, forming the corresponding ammonium intermediate **8n**-F, and the released fluoride subsequently activates **2a**. However, the reaction outcome is consistent with the inability of the α-trifluoromethyl carbanion **9**⁻ to attack the ammonium intermediate **8n**⁺, probably due to the increased steric hindrance at the electrophilic Csp² carbon of **8n**⁺. As a result, the α-trifluoromethyl carbanion **9**⁻ is protonated in the reaction mixture while the ammonium intermediate **8n**⁺decomposes into some undefined by-product.]*

- Under racemic conditions, the product **3ag** is not formed (entry 2). After 1h, allyl fluoride **1o** is totally consumed (>99%), while the 2 equiv. of *gem*-difluoroalkene **2a** remain unreacted. The main by-product formed was isolated and characterised as the carbocycle **D**.

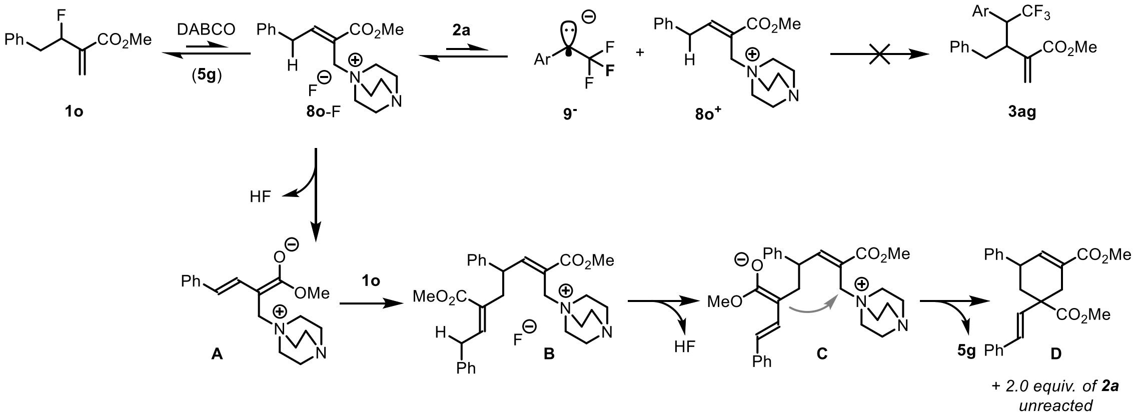

Figure S6. Proposed mechanism for the formation of carbocycle **D** under racemic conditions.

*[A plausible mechanism for the formation of **D**, arising from the dimerization of allyl fluoride **1o**, is shown above. Fluoride anion deprotonates the benzylic-allylic position of the ammonium species **8o**-F and generates the extended conjugated enolate **A**. Vinylogous addition of **A** to the α-position of another molecule of **1o** forms intermediate **B**. Subsequent deprotonation of the benzylic-allylic position of **B** by fluoride generates enolate **C**, which undergoes intramolecular S_N2 to afford the six-membered carbocycle **D** and release DABCO (**5g**).]*

^{1}H NMR

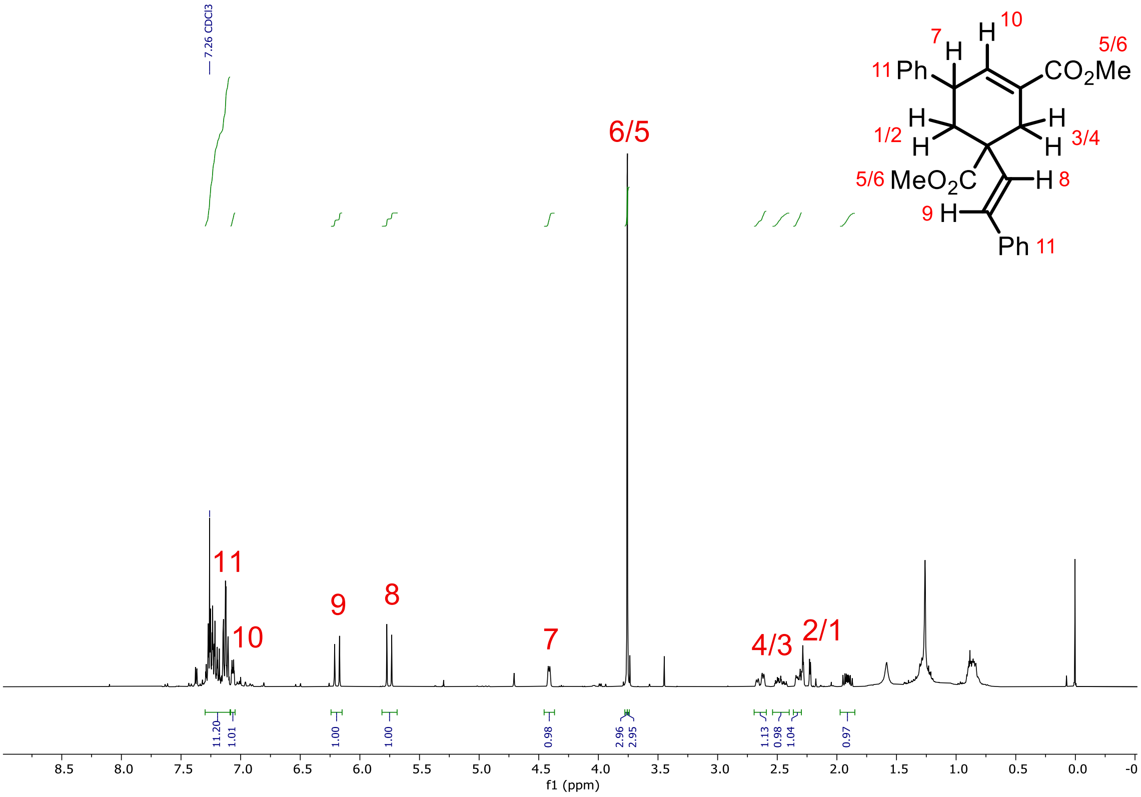

Figure S7. ^{1}H NMR spectrum of **D** at 400 MHz in $CDCl_3$.

^{13}C NMR

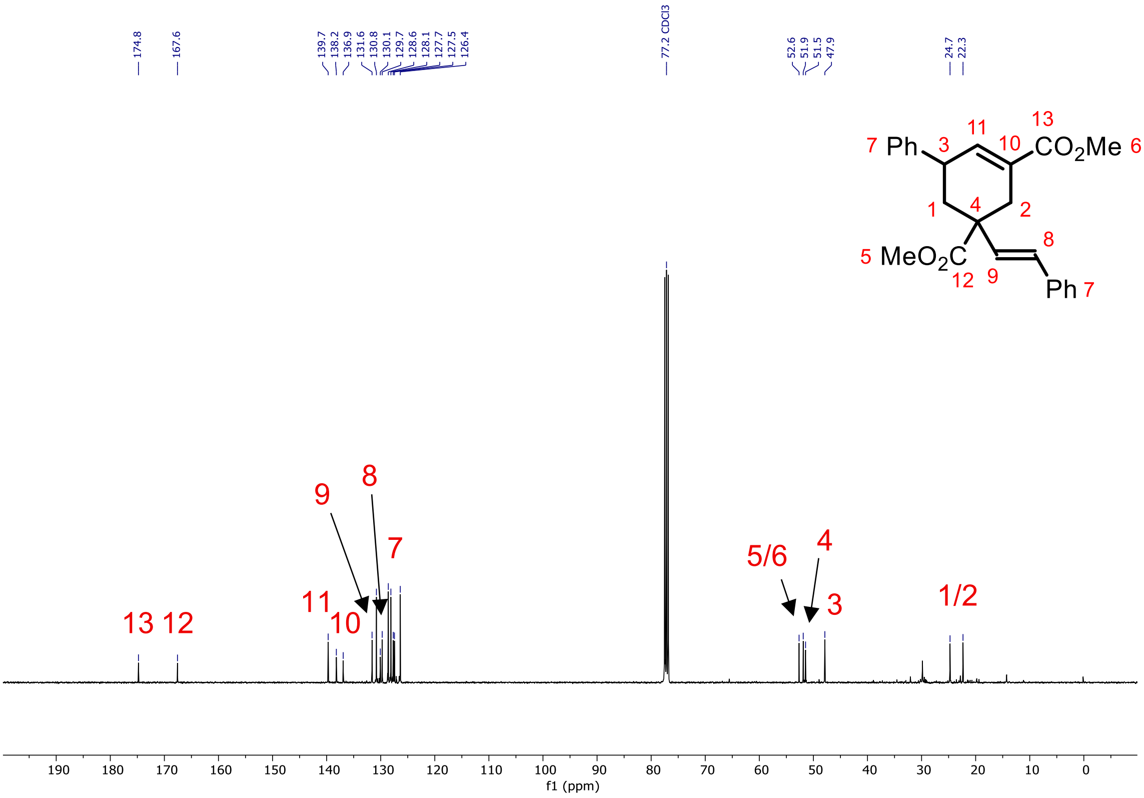

Figure S8. ^{13}C NMR spectrum of **D** at 101 MHz in $CDCl_3$.

HPLC–MS (ESI⁺): *m/z* calculated for $C_{24}H_{25}O_4$ $[M+H]^+$ = 377.18; found = 377.1.

- Under asymmetric conditions, nor product **3af** neither product **3ag** are formed (entries 3 and 4). In this case, however, both starting materials (**1n**/**1o** and **2a**) were recovered unreacted after 18h.

  *[This outcome, consistent with the mechanistic data shown in Figure 3 of the paper and in Section I of the Supporting Information, can be rationalise as follows: (i) The equilibria leading to the activation of the allyl fluoride **1a** by DABCO (**5g**) to form **8**-F is more endergonic in toluene (+9.0 kcal/mol) than in THF (+2.2 kcal/mol). (ii) The use of the bulky, less-nucleophilic chiral catalysis $(DHQD)_2AQN$ (**5c**) in $PhCF_3$, instead of DABCO **5g** in THF, further disfavours the formation of the ammonium intermediate **8**-F. (iii) Considering that in ammonium intermediates derived from alkyl allyl fluorides (e.g. see **8n**-F in Figure S5 and **8o**-F in Figure S6) the newly formed double bond is not conjugated with an aromatic ring, their formation is expected to be more thermodynamically uphill. As a result, the formation of ammonium intermediates from alkyl allyl fluorides under asymmetric catalytic conditions is not feasible, and the reaction do not proceed.]*

H.2. Gem-difluoroalkenes

To study the generality and limitations of the developed XEC protocol, in addition to the substrates shown in Figure 3 of the manuscript, we examined the reactivity aromatic *gem*-difluoroalkenes bearing electronically diverse substituents (**2m-2q**, **2v**), as well as the aliphatic *gem*-difluoroalkene **2w**.

We concluded that the nature of the substituents on the aromatic ring of the *gem*-difluoroalkenes **2** exerts a significant impact on their reactivity. This influence can be summarized as follows:

- *gem*-Difluoroalkenes **2** bearing electron-withdrawing substituents able of stabilizing the transiently formed α-trifluoromethyl carbanion $\mathbf{9}^-$ by resonance successfully participate in the catalytic asymmetric XEC protocol. For example, the *para*-substituted products **3n**-**3t** and the *ortho*-substituted products **3u**-**3v** (Figure 2 of the manuscript).
- *gem*-Difluoroalkenes **2** bearing electron-withdrawing groups unable to stabilize the α-trifluoromethyl carbanion $\mathbf{9}^-$ by resonance – either aromatic ring with purely inductive substituents (e.g. products **3aa-3ac**) or electron-withdrawing substituents with a resonance contribution located at the *meta* position of the aryl ring (e.g. products **3y** and **3z**) – exhibited sluggish reactivity under catalytic asymmetric conditions. In these cases, however, the corresponding product could be obtained in good yields using DABCO as the catalyst in THF (see figure S9).
- *gem*-Difluoroalkenes **2** with electron-neutral aryl rings, such as **3ad**, or aliphatic substituents, such as **3ae,** were reluctant to participate in the XEC protocol under both asymmetric and racemic conditions (see figure S9).

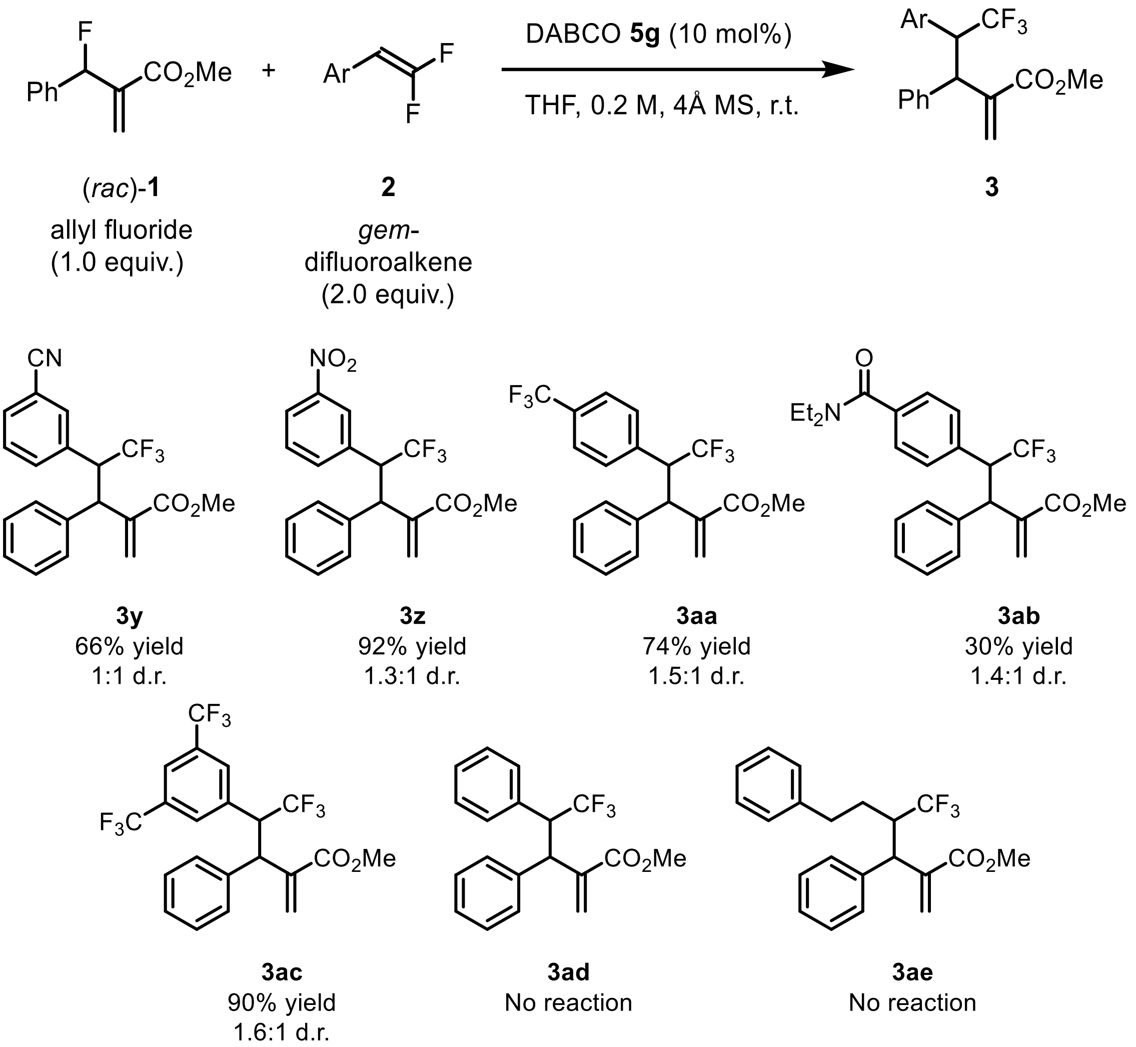

Figure S9. Reactivity of *gem*-difluoroalkenes bearing weakly activated or neutral aromatic and aliphatic substituents.

*[These results can be rationalised considering that the sequential activation of the two electrophilic coupling partners, prior to C-C bond formation, are both endergonic. In addition, the extend of the equilibrium for the activation of allyl fluoride **1** determines the effective concentration of fluoride anion in solution available for the activation of the gem-difluoroalkene **2**. In this context, formation of the corresponding α-trifluoromethyl carbanion derived from less activated gem-difluoroalkenes (i.e. bearing electron-withdrawing groups unable to stabilize the a-trifluoromethyl carbanion by ressonance) is more thermodynamically disfavoured. Under asymmetric catalytic conditions with $(DHQD)_2AQN$ **5c** in $PhCF_3$, the available fluoride concentration is insufficient to form the nucleophilic α-trifluoromethyl carbanion intermediate efficiently. In addition, the transition state for C-C bond formation lies higher in energy (TS_C, see section I.10), and the rections do not proceed. In contrast, when DABCO **5g** is employed in THF solution, larger catalytic amounts of fluoride are generated, the TS_C barrier is lowered, and the homoallylic trifluoromethylated products **3y–3ac** are formed. Finally, for non-activated gem-difluoroalkenes, formation of the corresponding a-trifluoromethyl carabnion is not feasible even under racemic catalytic conditions, and as a result products **3ad** and **3ae** are not formed.]*

I. Mechanistic Studies

I.1. ***Control experiments*** *– Generation of the catalytic species with DABCO*

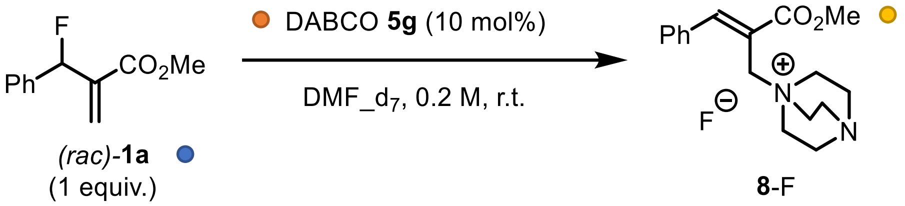

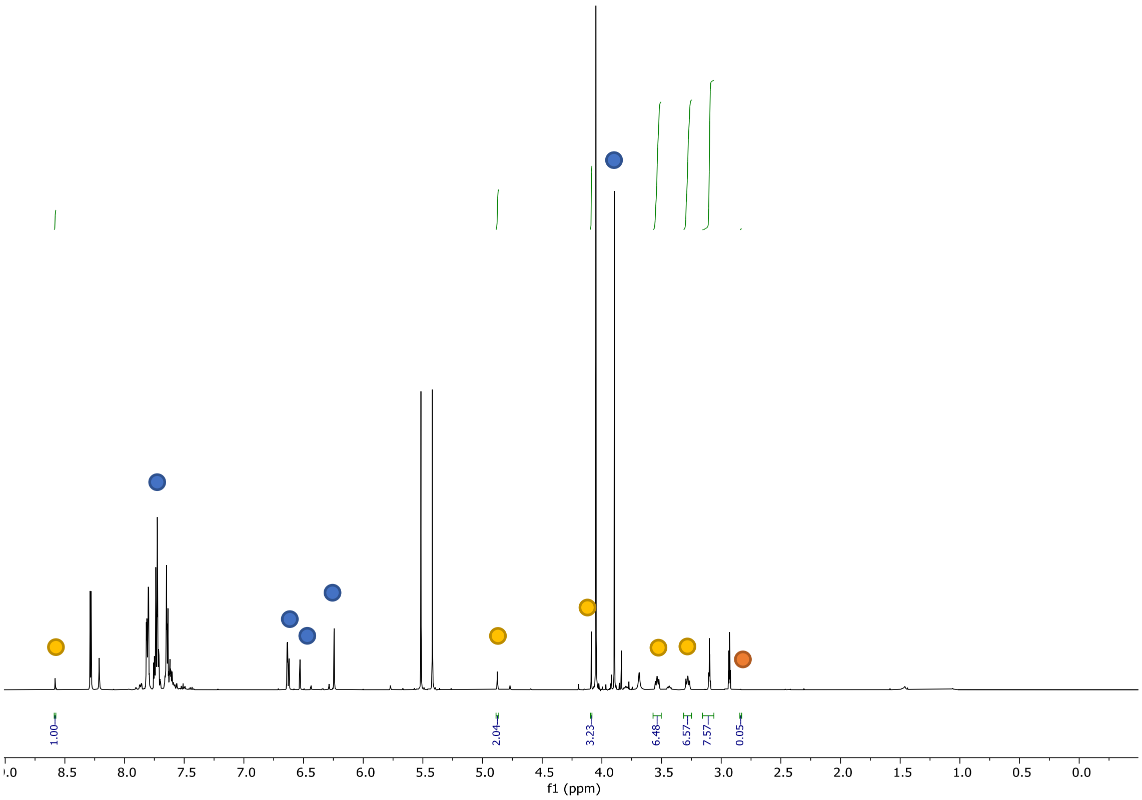

Figure S10. ^{1}H NMR spectrum at 500 MHz NMR field of 1 equiv. of allyl fluoride **1a** with 10 mol% of DABCO **5g** in DMF_d$_7$.

[In DMF_d$_7$, the use of 10 mol% of DABCO ***5g*** *(orange bubble) leads to the formation of >95% of the ammonium intermediate* ***8****-F (yellow bubbles) at the equilibration time.]*

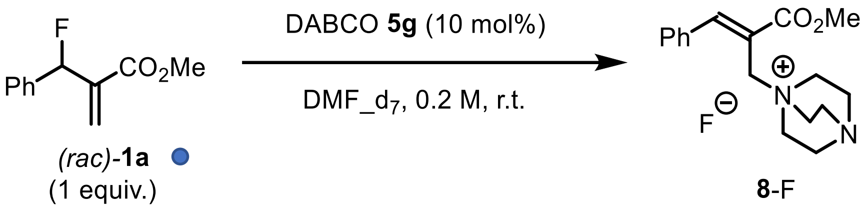

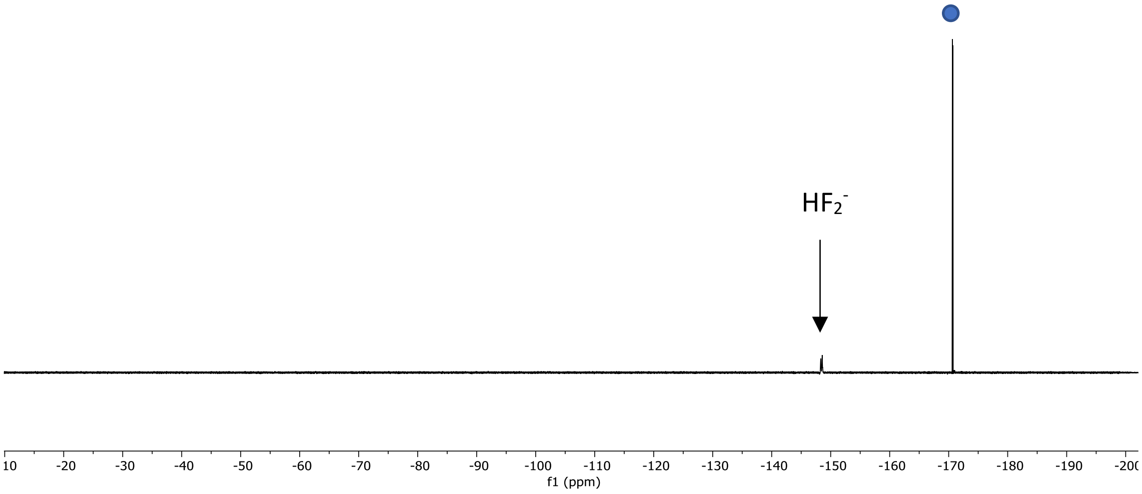

Figure S11. ^{19}F NMR spectrum at 471 MHz NMR field of 1 equiv. of allyl fluoride **1a** with 10 mol% of DABCO **5g** in DMF_d$_7$.

[In DMF_d$_7$, the resonance at -149 ppm can be attributed to HF_2^- or other hydrogen-bonded species, formed upon protonation of fluoride by protic impurities or weakly acidic C-H bonds.]

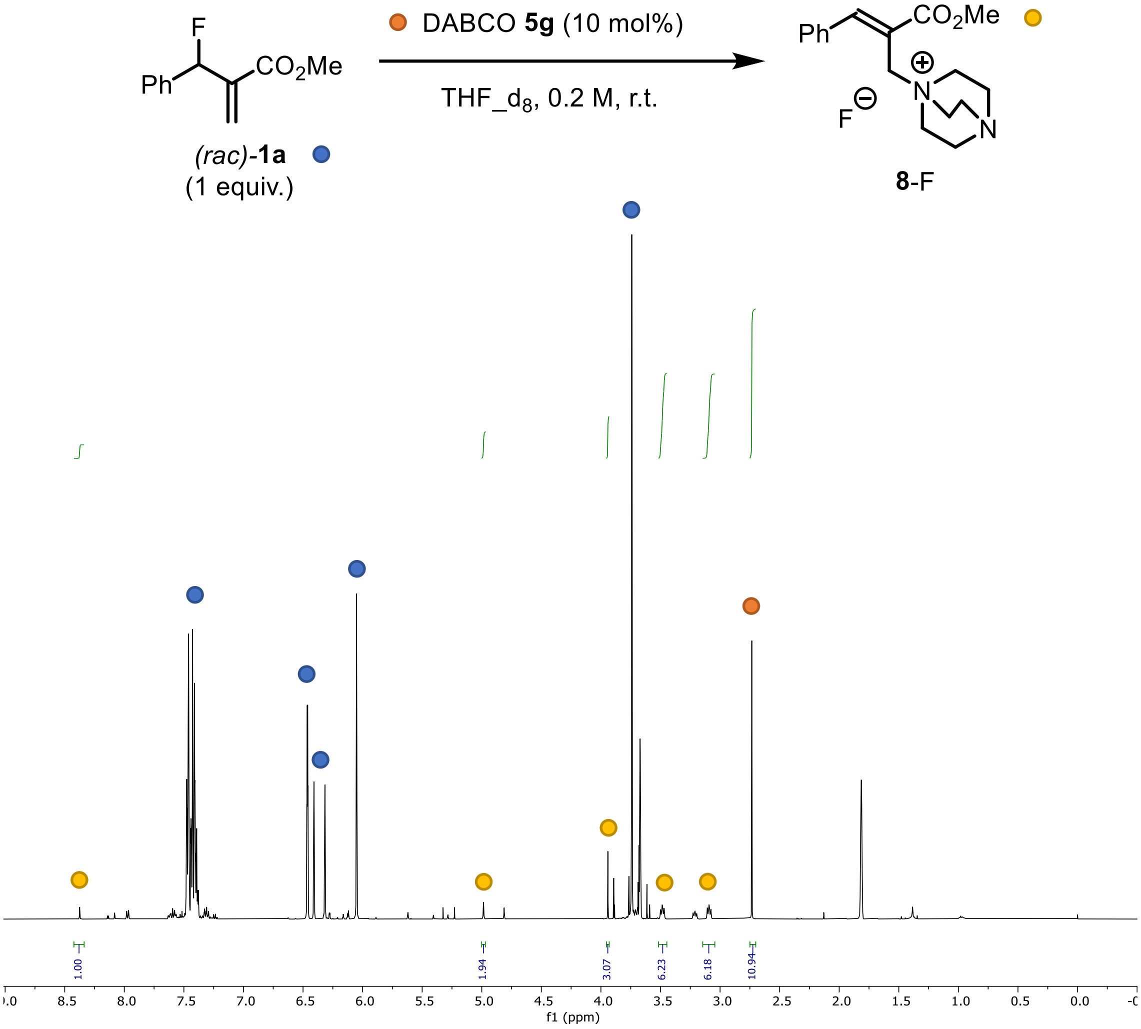

Figure S12. ^{1}H NMR spectrum at 500 MHz NMR field of 1 equiv. of allyl fluoride **1a** with 10 mol% of DABCO **5g** in THF_d₈.

[In THF_d₈, the use of 10 mol% of DABCO ***5g*** *(orange bubble) leads to the formation of 52% of the ammonium intermediate* ***8****-F (yellow bubbles) at the equilibration time.]*

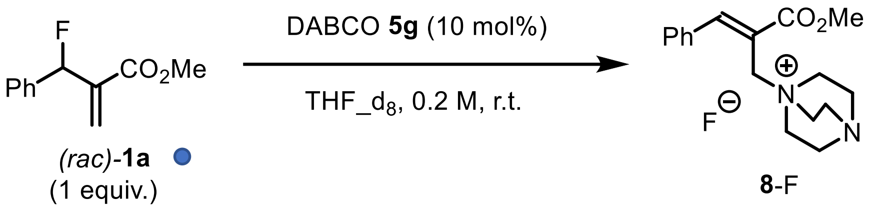

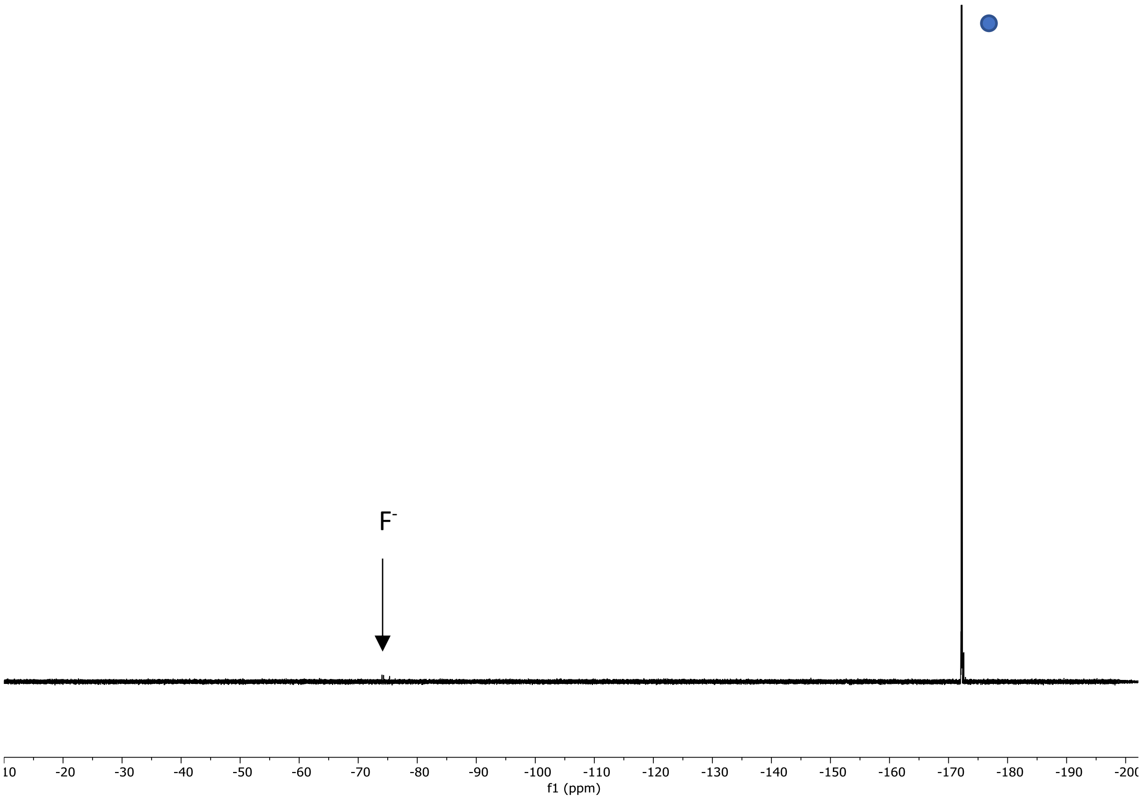

Figure S13. ^{19}F NMR spectrum at 471 MHz NMR field of 1 equiv. of allyl fluoride **1a** with 10 mol% of DABCO **5g** in THF_d_8.

[In THF_d_8, the signal around -74 ppm might be attributed to fluoride or a related fluorine-containing hydrogen-bonded species]

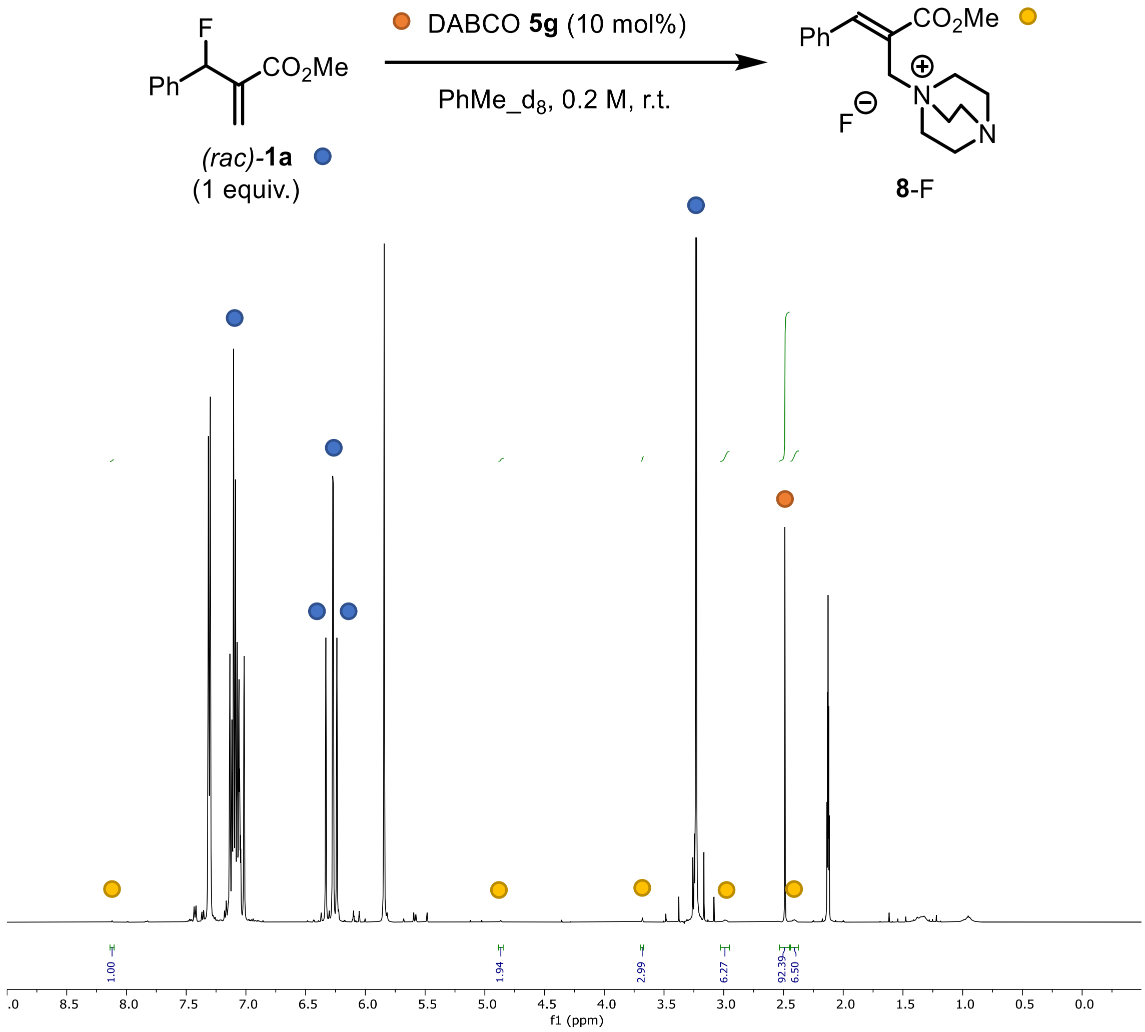

Figure S14. ^{1}H NMR spectrum at 500 MHz NMR field of 1 equiv. of allyl fluoride **1a** with 10 mol% of DABCO **5g** in toluene_d$_8$.

[In toluene_d$_8$, the use of 10 mol% of DABCO ***5g*** *(orange bubble) leads to the formation of 11% of the ammonium intermediate* ***8****-F (yellow bubbles) at the equilibration time.]*

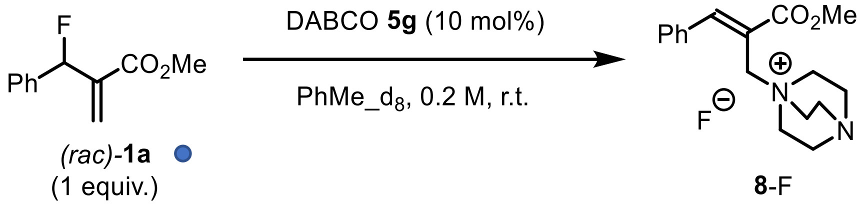

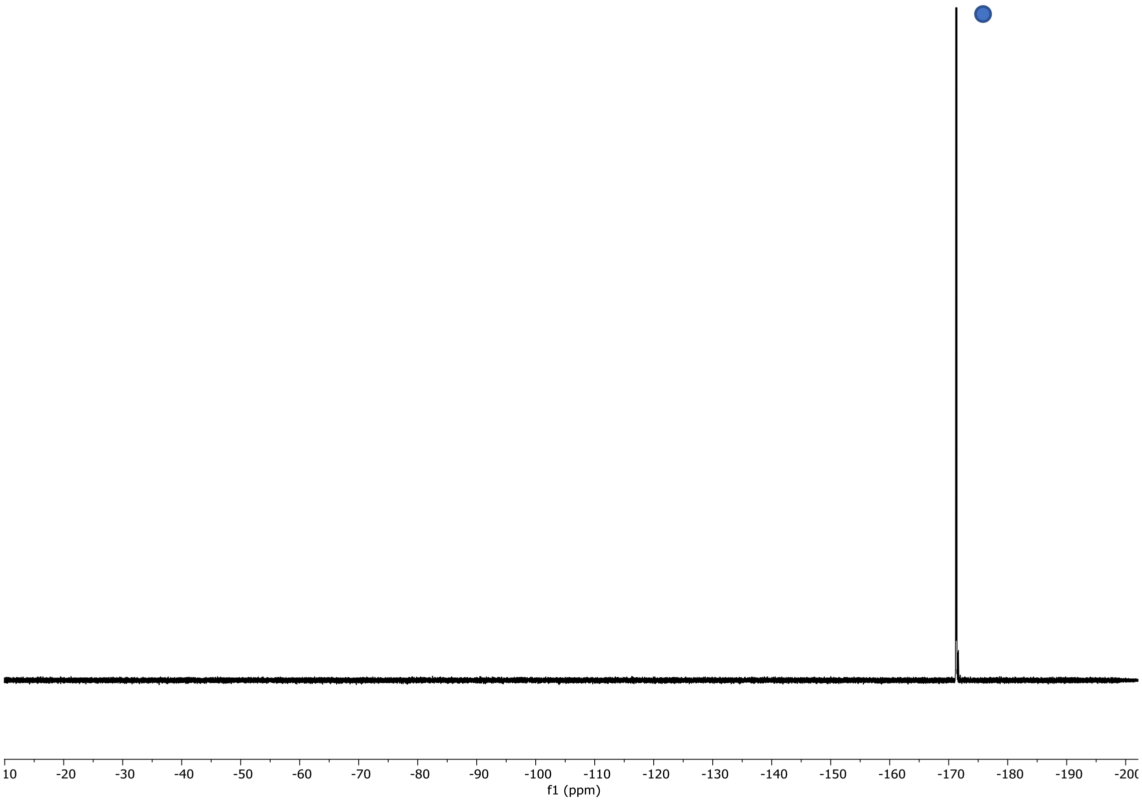

Figure S15. ^{19}F NMR spectrum at 471 MHz NMR field of 1 equiv. of allyl fluoride **1a** with 10 mol% of DABCO **5g** in toluene_d_8.

*[In toluene_d_8, the ^{19}F NMR shows no fluorine resonances other than that of **1a**. This observation may be attributed to the low effective concentration of fluoride or fluoride-related species, and/or to unpredictable chemical shifts when measuring such a hard anion in an apolar aromatic solvent.]*

I.2. Control experiments – Generation of the catalytic species with (DHQD)₂AQN

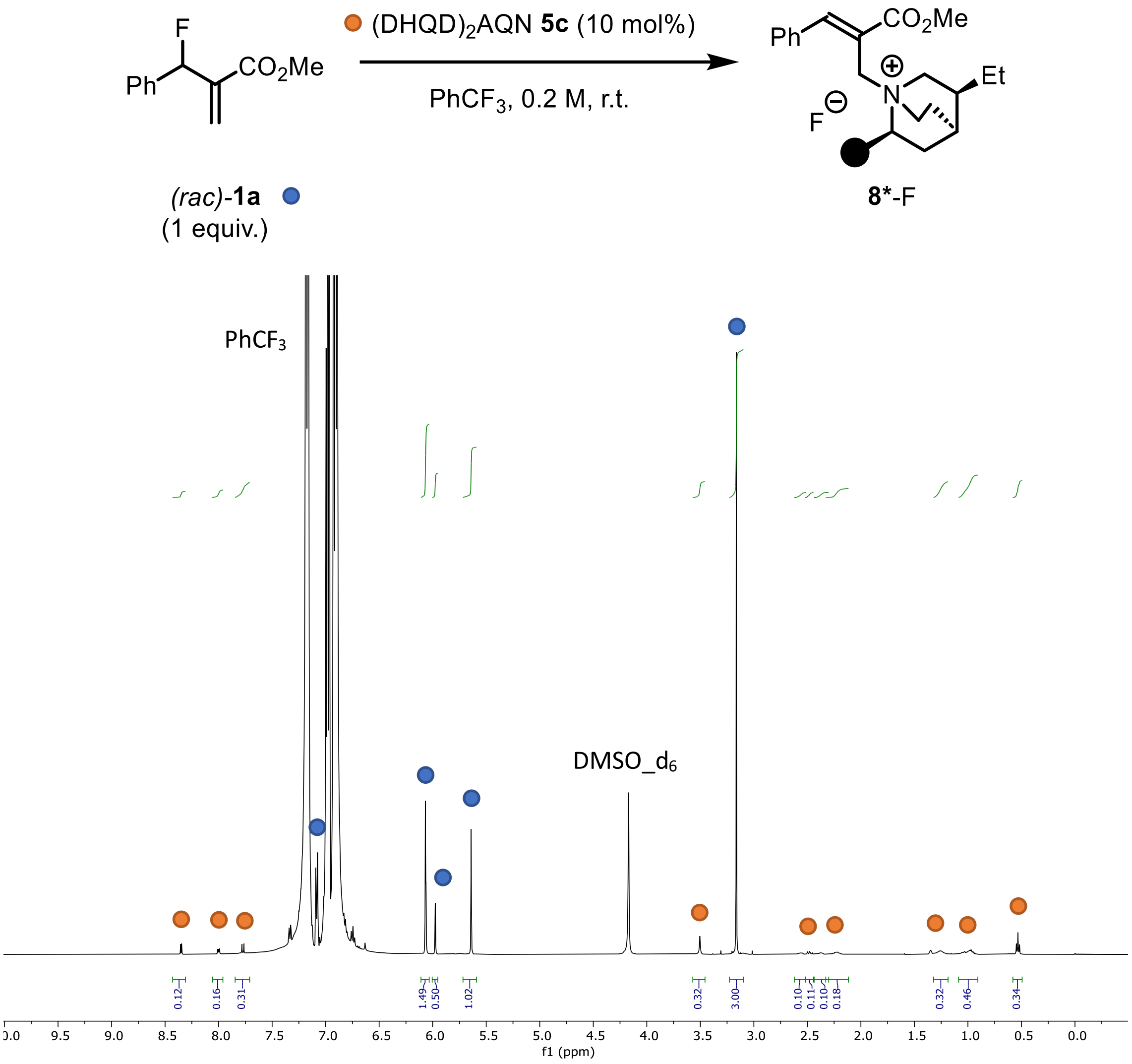

Figure S16. ^{1}H NMR spectrum at 500 MHz NMR field of 1 equiv. of allyl fluoride **1a** with10 mol% of (DHQD)$_2$AQN **5c** in non-deuterated PhCF$_3$ using a DMSO_d$_6$ inset.

*[In PhCF$_3$, the use of 10 mol% of (DHQD)$_2$AQN **5c** (orange bubble) leads to no detectable formation of the ammonium intermediate **8***-F.]*

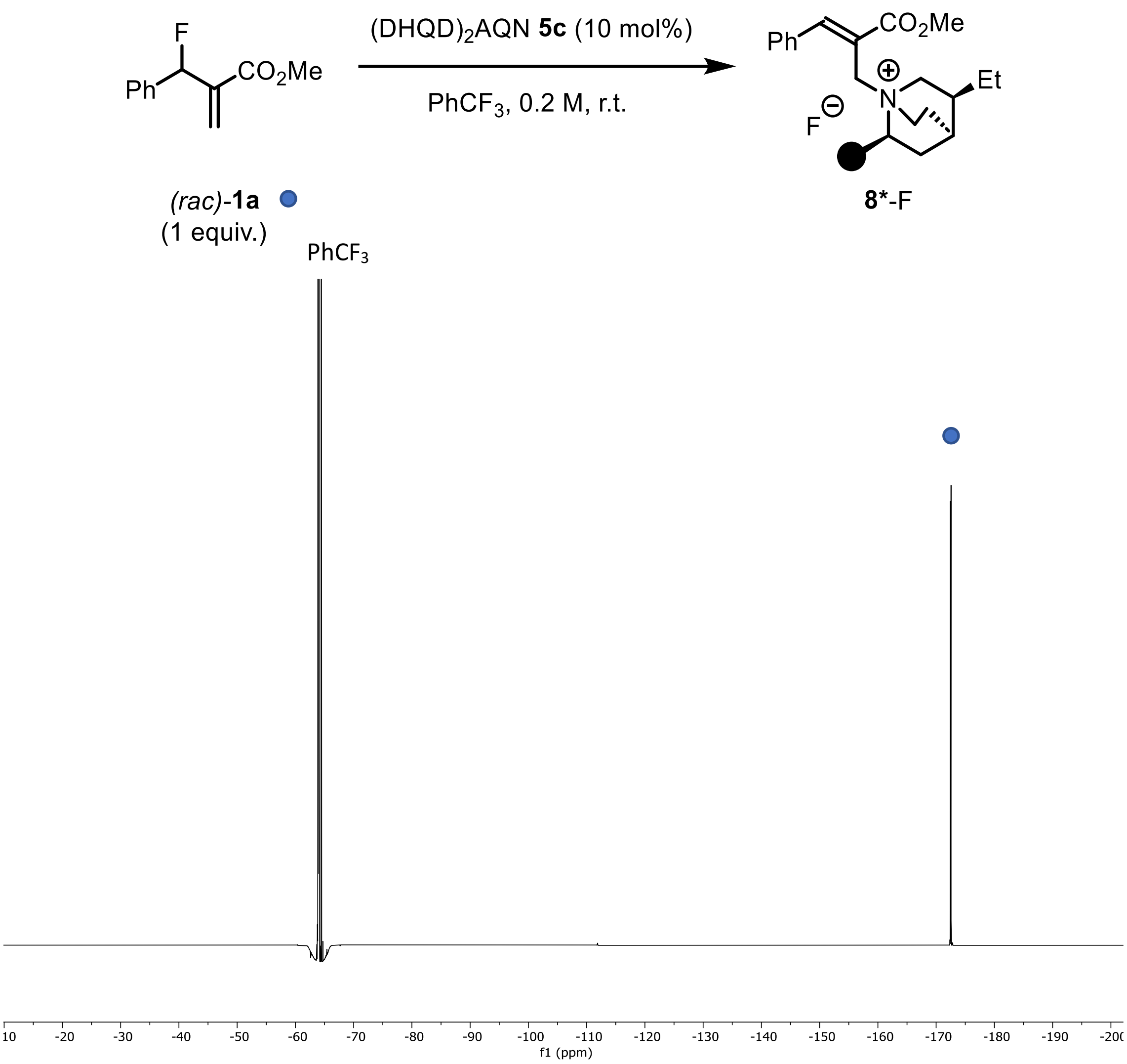

Figure S17. ^{19}F NMR spectrum at 471 MHz NMR field of 1 equiv. of allyl fluoride **1a** with 10 mol% of $(DHQD)_2AQN$ **5c** in non-deuterated $PhCF_3$ using a DMSO_d_6 inset.

[In PhCF3, the use of 10 mol% of (DHQD)2AQN **5c** *led to not detectable formation of the ammonium intermediate* ***8****-F *by* ^{1}*H NMR. Accordingly, the* ^{19}*F NMR does not show any new resonance.]*

***I.3. Control experiments** – Influence of the solvent in the regioselectivity (γ vs. α) cross-electrophile catalytic coupling between allyl fluorides (**1**) and gem-difluoroalkenes (**2**)*

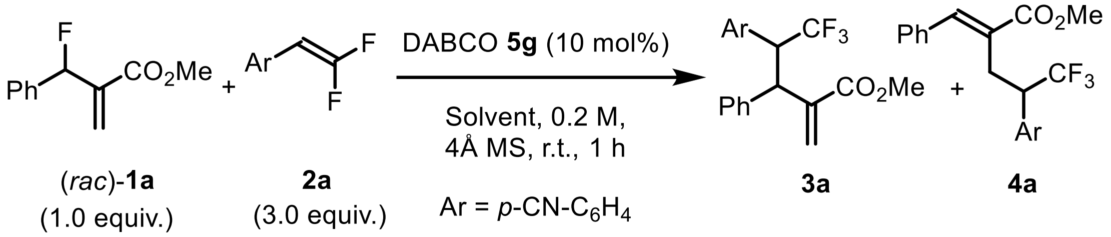

Entry	Solvent	Conv. (%)[a]	3a (%)[a]	r.r. (3a:4a)[a]	d.r.[a]
**1**	Toluene	>99	81	>20:1	2.3:1
**2**	$PhCF_3$	>99	75	>20:1	1.5:1
**3**	CH_2Cl_2	>99	46	>20:1	1.9:1
**4**	THF	>99	95	>20:1	2.3:1
**5**	MeCN	>99	44	5.5:1	1.2:1
**6**	DMF	>99	32	1:1.5	1.3:1

Table S15. Influence of the solvent in the regioselectivity. [a] Determined by ^{1}H NMR spectroscopy using 1,3,5-trimethoxybenzene as the internal standard. THF:Tetrahydrofuran, DMF: *N,N*-dimethylformamide.

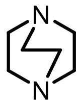

DABCO **5g**

*[Under the effect of 10 mol% of DABCO, the reaction in solvents of low or medium polarity selectively affords the γ-alkylated product **3a**. Converserly, in polar aprotic solvents such as MeCN of DMF, the regiochemical outcome is shifted towards the α-alkylation manifold, affording mixtures of **3a** and **4a**.]*

***I.4. Control experiments** – Influence of the aromatic spacer of the dimeric chiral Lewis-base catalyst in the asymmetric cross-electrophile coupling under optimised conditions.*

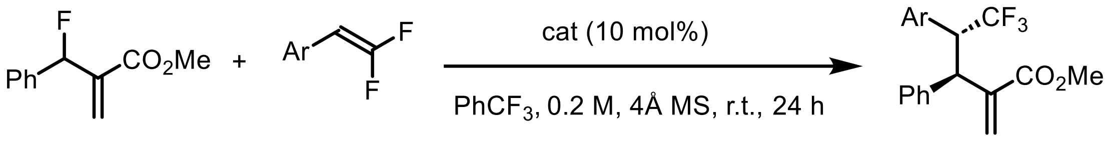

(*rac*)-**1a** (1.0 equiv.)   **2a** (3.0 equiv.)   Ar = *p*-CN-C_6H_4 **3a**

Ent.	Catalyst	Conv. (%)[a]	3a (%)[a]	r.r. (3a:4a)[a]	d.r.[a]	e.r.[b]
**1**	$(DHQD)_2$PHAL **5a**	40	<5	>20:1	n.d.	n.d.
**2**	DHQD **5m**	<5	n.d.	-	-	-
**3**	DHQD-Ph **5n**	40	15	>20:1	3:1	88:12
**4**	$(DHQD)_2$AQN **5c**	68	56	>20:1	8:1	96:4

Table S16. Influence of the aromatic spacer of the dimerci catalyst in the reaction outcome. [a] Determined by ^{1}H NMR spectroscopy using 1,3,5-trimethoxybenzene as the internal standard. [b] Determined by HPLC analysis using a chiral column. n.d.: not determined.

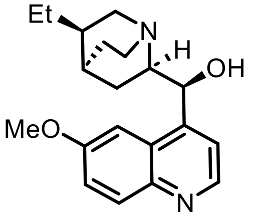

DHQD **5m**

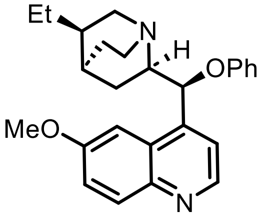

DHQD-Ph **5n**

*[Using the optimised reaction conditions (e.g. 0.2 M of $PhCF_3$ with 4Å MS at r.t.), the catalytic performance of various dihydroquinidine-based catalysts closely related to the optimal catalyst **5c** was investigated. As evidenced in the table above, the yields, diastereo- and enantioselectivities were drastically reduced. This experiment evidences the crucial role of the aromatic antraquinone spacer of **5c** in achieving the excellent levels of reactivity and stereocontrol in the asymmetric cross-electrophile coupling.]*

***I.5. Control experiments** – Study of the potential erosion of the diastereoselectivity of the homoallylic trifluoromethylated product **3a** by the catalyst **5c**.*

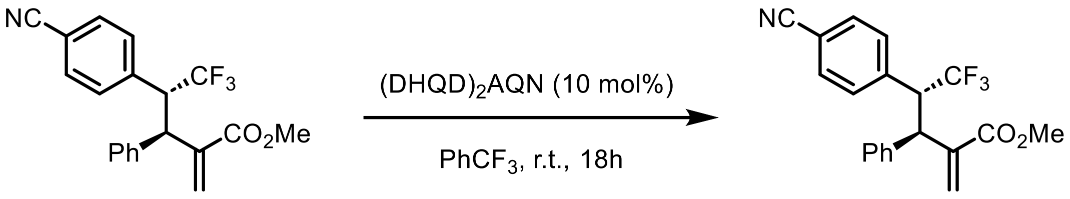

3a >20:1 d.r. (1 equiv.) → **3a** >20:1 d.r. (1 equiv.)

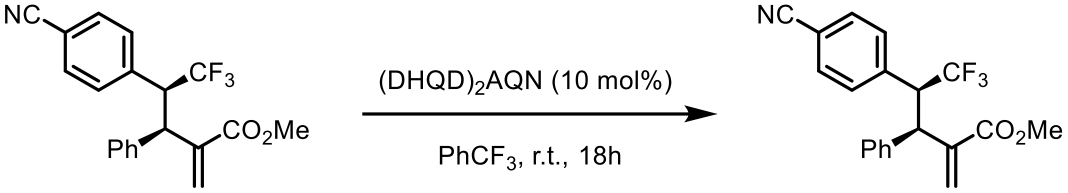

(*rac*)-**3a'** >20:1 d.r. (1 equiv.) → (*rac*)-**3a'** >20:1 d.r. (1 equiv.)

Diastereopure products **3a** and (*rac*)-**3a'** (1 equiv., 0.1 mmol, >20:1 d.r.) were weighted independently into a 5 mL vial equipped with a magnetic stirring bar and dissolved with 0.5 mL of $PhCF_3$ (0.2 M). Subsequently, 10 mol% of catalyst **5c** was added in each vial. Both mixtures were stirred for 18 h at room temperature. Then, $PhCF_3$ was dried under vacuum, each crude mixture was diluted in $CDCl_3$ and transferred into two NMR tubes for ^{1}H NMR analysis.

*[After 18 h, neither of the two diastereoisomers showed epimerisation. Therefore, the diastereoselectivity is not eroded by the effect of the catalyst **5c**]*

I.6. Control experiments *– Study of the potential erosion of the diastereoselectivity of the homoallylic trifluoromethylated product* ***3a*** *by fluoride.*

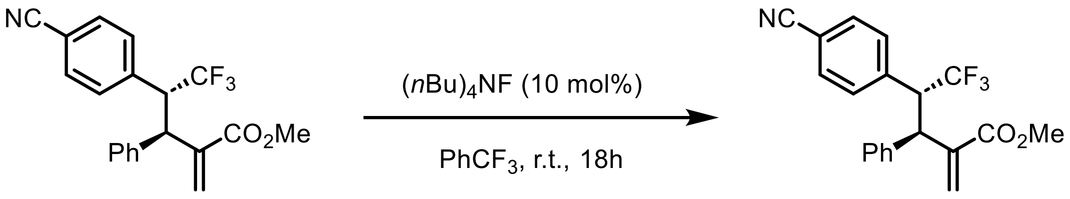

3a >20:1 d.r. (1 equiv.) → **3a** >20:1 d.r. (1 equiv.)

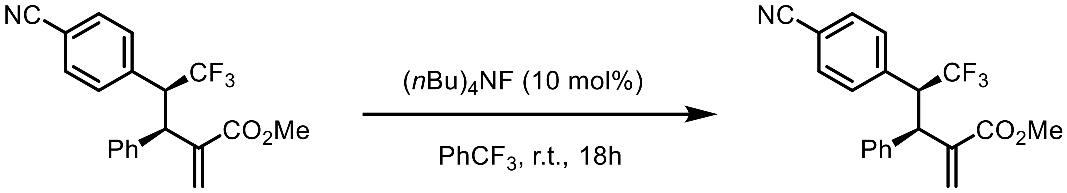

(*rac*)-**3a'** >20:1 d.r. (1 equiv.) → (*rac*)-**3a'** >20:1 d.r. (1 equiv.)

Diastereopure products **3a** and (*rac*)-**3a’** (1 equiv., 0.1 mmol, >20:1 d.r.) were weighted independently into a 5 mL vial equipped with a magnetic stirring bar and dissolved with 0.5 mL of $PhCF_3$ (0.2 M). Subsequently, 10 mol% of tetrabutylammonium fluoride 1.0 M in THF **8a** was added in each vial. Both mixtures were stirred for 18 h at room temperature. Then, $PhCF_3$ was dried under vacuum, each crude mixture was diluted in $CDCl_3$ and transferred into two NMR tubes for ^{1}H NMR analysis.

[After 18 h, neither of the two diastereoisomers showed epimerisation. Therefore, the diastereoselectivity is not eroded by the effect of the fluoride]

I.7. Kinetic and stereochemical profiles – Evolution of the allyl fluoride ***1a***

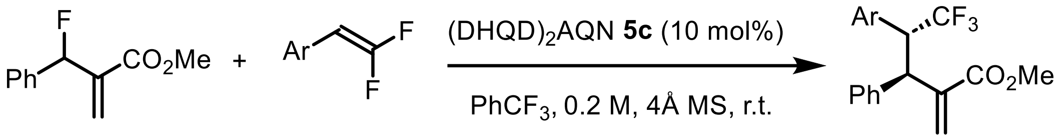

(*rac*)-**1a** (1.0 equiv.) **2a** (3.0 equiv.) Ar = *p*-CN-C_6H_4 **3a**

Allyl fluoride evolution

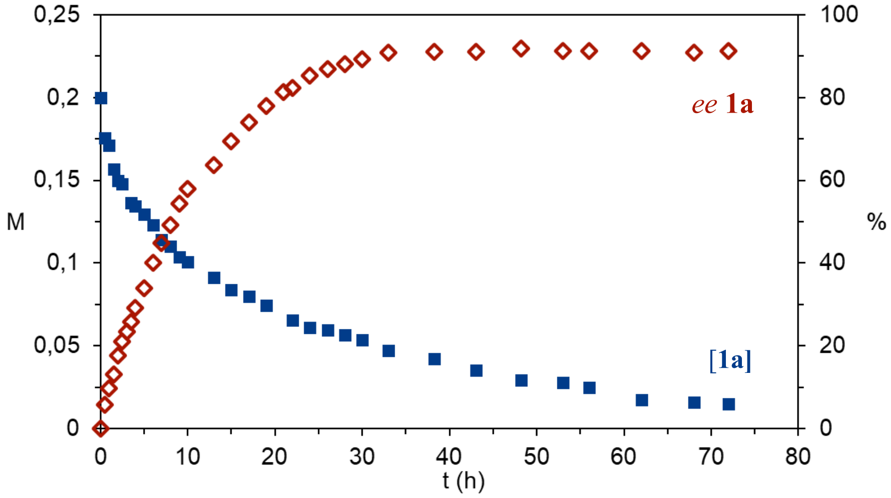

Figure S18. Kinetic and stereochemical profiles (concentration in blue, and enantiomeric excess (*ee)* in red of **1a** *vs.* time) under optimised catalytic conditions, analysed by GC.

*[The starting material (rac)-**1a** is consumed at the onset of the reaction (Figure S8, blue squares), with preferential depletion of (S)-**1a** leading to its progressive enrichment in (R) (Figure S8, red diamonds). As the (S)-**1a** becomes depleted, the reaction rate slows down and eventually plateaus at a scalemic composition of 95:5 (R/S).]*

GC Chromatogram of (*R*)-1a

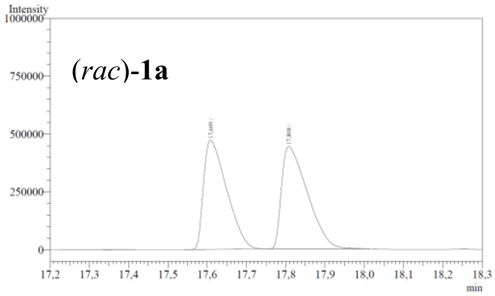

Peak#	Ret.Time	Area	Height	Conc.	Unit Mark ID#	Cmpd Name
1	17,609	1927828	470785	48,797		
2	17,808	2022880	440186	51,203		
Total		3950708	910971			

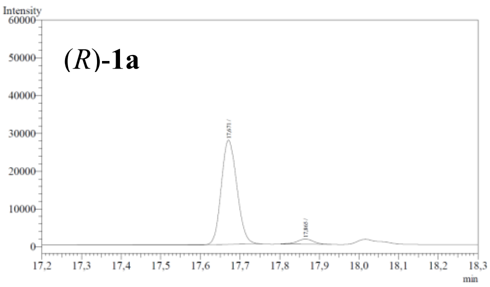

Peak#	Ret.Time	Area	Height	Conc.	Unit Mark ID#	Cmpd Name
1	17,671	75782	27535	95,160		
2	17,865	3854	1339	4,840		
Total		79636	28874			

The 90% ee of allyl fluoride **1a** was determined by chiral GC (Agilent CP-Chirasil Dex CB GC column: 60 ºC (hold 2 min), ramp 5 ºC/min to 160ºC, ramp 10 ºC/min to 215ºC, hold 2 min), and its absolute (*R*)-configuration was determined by chemical correlation.[3b]

I.8. ***Kinetic and stereochemical profiles*** *– Evolution of the homoallylic trifluoromethylated product* ***3a***

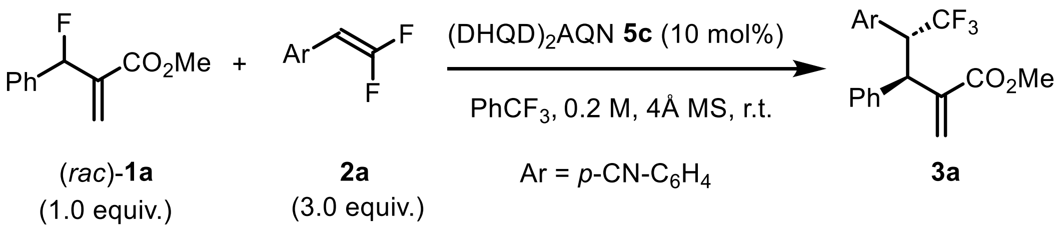

Product Formation

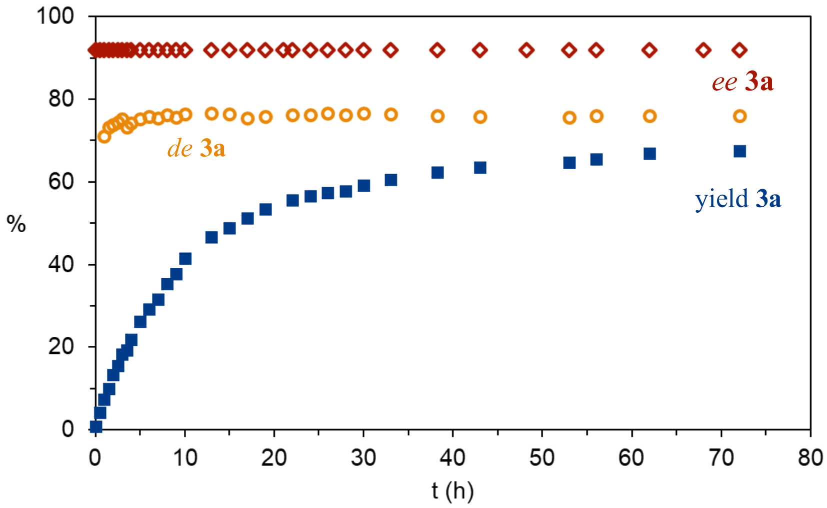

Figure S19. Kinetic and stereochemical profiles of the formation of the homoallylic trifluoromethylated product **3a** (yield in blue; diastereomeric excess (*de*) in yellow; and enantiomeric excess (*ee)* in red of **3a** *vs.* time) under optimised catalytic conditions, analysed by GC.

[The formation of product ***3a*** *exhibits two distinct kinetic regimes (Figure S9, blue squares), yet it is consistently formed with 96:4 e.r. (92% ee, Figure S9, red diamonds) and 8:1 d.r. (78% de, Figure S9, orange dots), indicating that the stereodetermining step involves a stereoconvergent, catalyst-controlled C-C bond formation event.]*

***I.9. Kinetic model** – Fitting the experimental data with a kinetic model*

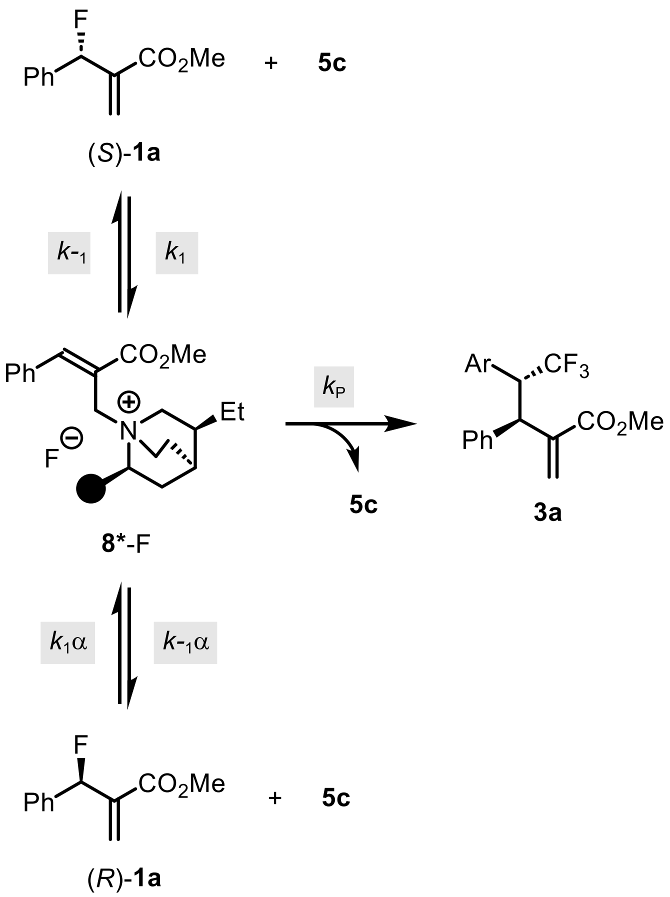

To explain the observed decay of substrate **1a** and its remaining enantiomeric excess, we globally fitted considering both the concentration and enantiomeric excess. the data using a kinetic model. For this, we considered (*S*)-**1a** to be in equilibrium with (*R*)-**1a** through the common intermediate **8***-F with an equal equilibrium constant K_1, but with different forward and backward rates, (i.e., $K_1 = \frac{k_1}{k_{-1}} = \frac{k_1 \alpha}{k_{-1} \alpha}$, where α is the asymmetry factor (0 < α < 1). Product formation occurs directly from the common intermediate **8***-F (k_p) without considering the equilibrium between **2a** and **9**$^-$. We justify this by considering that in $PhCF_3$ the formation of **8***-F is considerably uphill, and the barrier is much higher than for the addition of the fluoride anion to substrate **2a** (and because **2a** is always in excess).

Fitting of the experimental data with the kinetic model

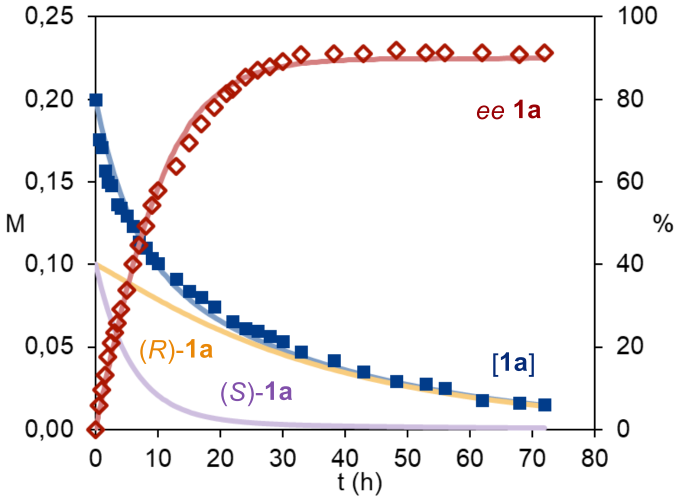

Figure S20. Kinetic and stereochemical profiles (concentration in blue squares, and enantiomeric excess (*ee)* in red diamonds of **1a** *vs.* time) under optimised catalytic conditions. The fitting of the kinetic model is repsresented in solid lines.

A great fit with the experimental data could be obtained (Figure S10, blue and red solid lines) using this simplified model when the formation of product **3a** and the reformation of substrate **1a** from intermediate **8***-F occurred at comparable rates ($k_{\mathrm{P}}/(k_{\mathrm{P}} + k_{-1} + \alpha k_{-1})$ = 0.7, so 70% product formation from **8***-F *vs.* 30% reformation of (*S/R*)-**1a**), with the overall rate-limiting step being the formation of **8***-F ($k_1 = 12\ h^{-1}, K_1 = 2.5 \cdot 10^{-5}\ \mathrm{M}^{-1}$). This is consistent with DFT calculations (Figure S11) using the achiral catalyst **5g**, where similar activation barriers for the conversion of intermediate **8-**F to either the product or the substrate were predicted, and with NMR observations which did not demonstrate formation of intermediate **8***-F. Therefore, the observed enantioenrichment up to a 90% (95:5 e.r.) is due to the initial faster consumption of (*S*)-**1a** (α = 0.12), Figure S10, violet solid line), while partial racemization at later stages due to reformation of (*R*/S)**-1a** from **8***-F prevents it complete resolution.

I.10. Density Functional Theory

Density functional theory was performed in Gaussian09 (Revision D.01)[6] with an *UltraFine* grid. Calculations were done using B3LYP[7] as the functional with dispersion correction D3BJ[8] and implicit SMD solvation[9] (DMF, THF or toluene) for both optimization and single point calculations. For geometry optimizations, 6-31+G(d,p)[10] was used as basis set. Potential energies were refined by calculation of single point energies on the previously optimized structures using the aug-cc-pVTZ[11] basis set. The final Gibbs free energies are calculated as $G_f = E_{sp} + G_{opt} - E_{opt}$ where G and E are the Gibbs free energy and electronic energy, and the subscripts *f*, *sp* and *opt* refer to the final, single point and optimization, respectively. Gibbs free energy corrections were computed at 298 K and 1 atm. Vibrational frequency calculations were carried out on all optimized structures to ensure that the structure is a minimum / transition state. In all the calculations the symmetry was disabled with the keyword *nosymm*. In specific cases, several rotamers were considered and the lowest energy rotamer was chosen for further structures on the reaction path. Gibbs free energies were corrected for reference state by raising the energy of every calculated compound by 1.89 kcal/mol. All anions were stabilized explicitly during computations by inclusion of a tetramethylammonium counterion. The free energy profiles of the reaction between allyl fluoride **1a** and *gem*-difluoroalkene **2a** were computed in the presence and of either catalyst DABCO (**5g**) or fluoride anions in three different solvents (Figure S11). As Gaussian09 does not include $PhCF_3$ as solvent, toluene and THF were chosen as the model solvents for asymmetric and racemic γ-selective XEC reactions, respectively. DMF was selected as the model solvent for α-selective XEC.

All input and output files have been uploaded to the following repository:

https://iochem-bd.bsc.es/browse/review-collection/100/478047/0f5b2a10b129d4362d493d5a

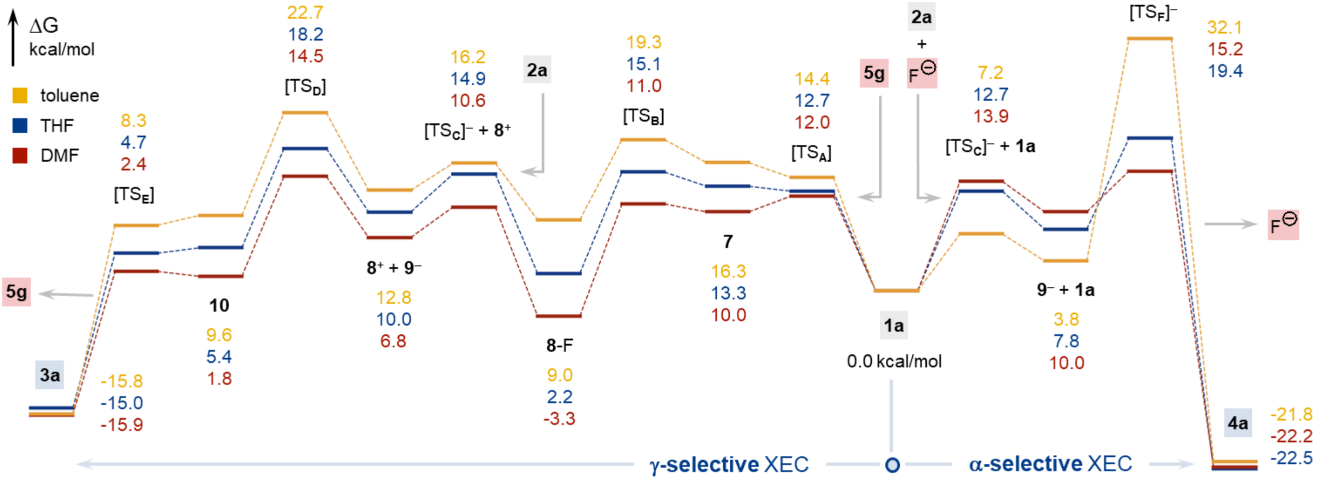

Figure S21. Computed energy values of the γ- and α-selective XEC reactions in toluene, THF and DMF.

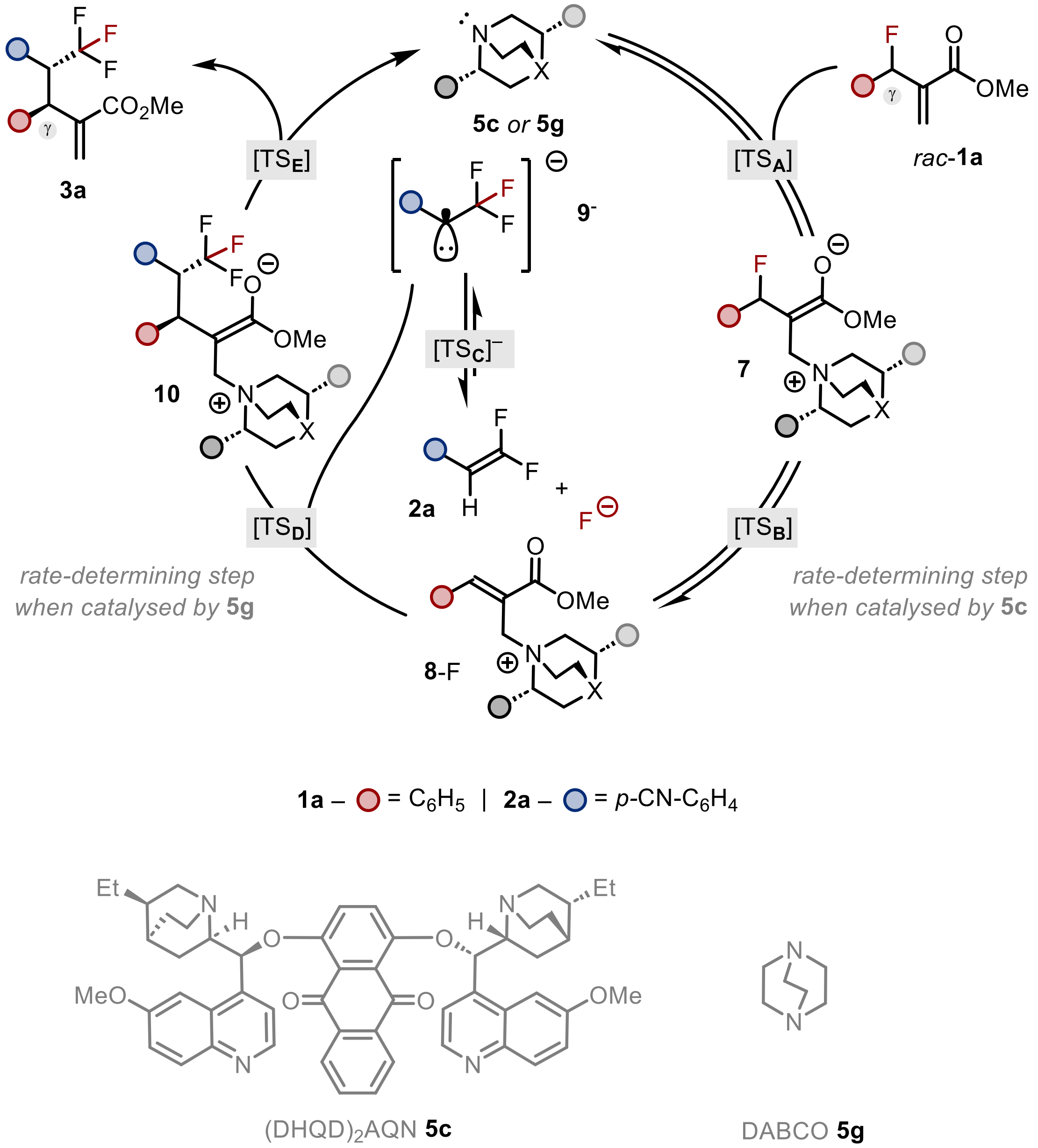

Figure S22. Proposed mechanism for *γ*-selective XEC reaction.

The addition of catalyst **5g** to **1a** results in the formation of the electrophilic intermediate **8**-F *via* a stepwise S_N2’ mechanism, accompanied by the ionisation of fluoride. DFT calculations reveal that the equilibrium position for the formation of **8**-F is highly solvent-dependent, being exergonic by –3.3 kcal/mol in DMF, but endergonic by +2.2 kcal/mol in THF and +9.0 kcal/mol in toluene (Figure S11). These energetic values are consistent with the experimental formation of **8-**F in the presence of 10 mol% of catalyst **5g** (Figures S4-S6) for DMF and THF, but not for toluene, as demonstrated by ^{1}H NMR spectroscopy in control experiments (section I.1).

The addition of fluoride — either released during the formation of $\mathbf{8}^{+}$ or introduced externally as TBAF— to *gem*-difluoroalkene **2a** generates the nucleophilic α-trifluoromethyl carbanion $\mathbf{9}^{-}$. As shown in Figure S11, this equilibrium is endergonic yet thermally accessible at room temperature in all three examined solvents. The process is less favorable in DMF than in THF, and even less so in toluene (6.2 kcal/mol difference with respect to DMF).

Considering the global energy landscape (Figure S11), the C-C bond-formation step is the rate-determining step (RDS) for both the formation of (*rac*)-**3a** (TS_D) (Figure S12) and the formation of (*rac*)-**4a** (TS_F^-) (Figure S13). The computed energy barriers correlate with the experimentally observed solvent-dependent regioselectivity (Table S10). The reaction of **1a** and **2a** in the presence of 10 mol% of **5g** produces a mixture of **3a** and **4a** in DMF (entry 6), whereas only γ-alkylated product **3a** forms in toluene and THF (entries 1, 4). In DMF, the calculated energy difference between TS_D and TS_F^- is 0.7 kcal/mol. This narrow energy gap results in the poor regioselectivity (**3a**/**4a** = 1:3.7 calculated through microkinetic analysis *vs.* 1:1.5 experimentally). Conversely, the RDS for γ-alkylation (TS_D) is 1.2 kcal/mol lower and 9.4 kcal/mol lower than TS_F^- in THF and toluene, respectively, which is consistent with the complete γ-selectivity observed in these solvents (Figure S11). Regarding the *γ*-selective XEC process, calculations were performed using DABCO (**5g**) as the catalyst, indicating that the rate-determining step (RDS) is the C-C bond-formation step (TS_D > TS_B) (Figures S11, S12). In contrast, for the asymmetric γ-selective XEC employing $(DHQD)_2AQN$ (**5c**), the RDS corresponds to the formation of **8**-F (TS_B > TS_D) (Figure S12), as demonstrated by control experiment I.2 and the kinetic model described in section I.9.

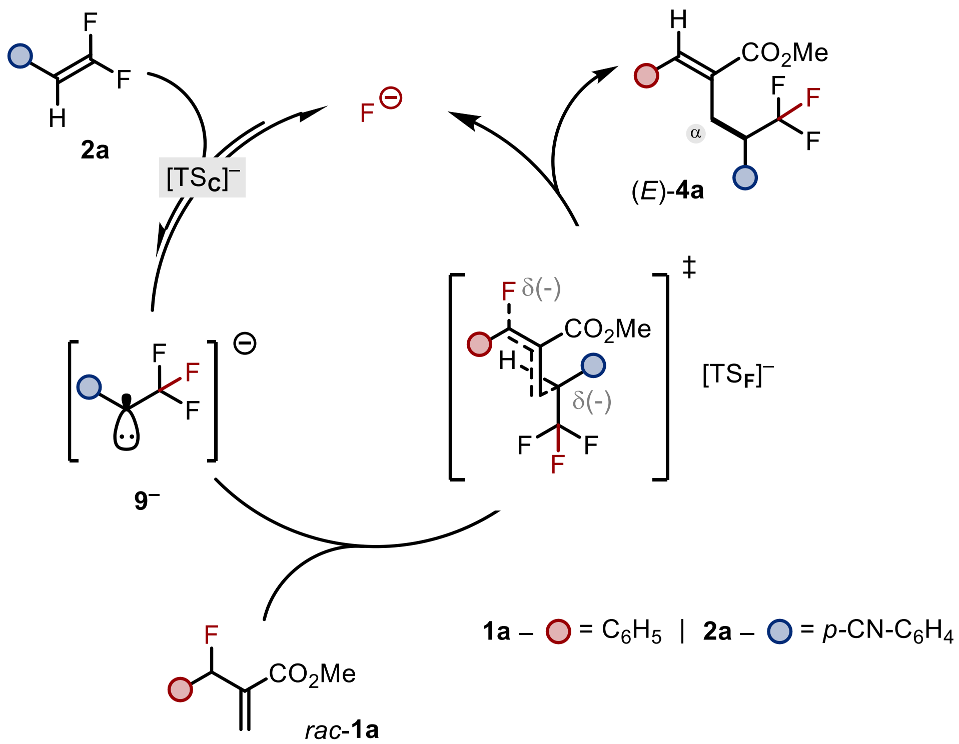

Figure S23. Proposed mechanism for α-selective XEC reaction.

With respect to the α-selective XEC, we propose that the process proceeds *via* a concerted S_N2'-type C–C bond-forming event, as no further intermediate (e.g. as **10** found for the γ-selective path) could be located beyond TS_F^-. In the optimized TS_F^- the hydrogen is located near the aryl group (red ball, Figure S13). Hence, the *trans* arrangement of the aryl and ester groups in the newly formed olefin, following C–C bond formation and simultaneous fluoride extrusion, yields product **4a** with high *E*-selectivity. The activation energy associated with this transformation varies significantly with solvent polarity: +15.2 kcal/mol in DMF, +19.4 kcal/mol in THF and +32.1 kcal/mol in toluene, making the process thermally unfeasible in toluene at room

temperature (Figure S11). These findings are consistent with experimental observations showing that the reaction proceeds in polar solvents, such as DMF and THF, but is completely suppressed in apolar media, such as toluene.

J. Derivatizations of product 3i

J.1. Reduction of the ester and nitrile groups – formation of the diol 6

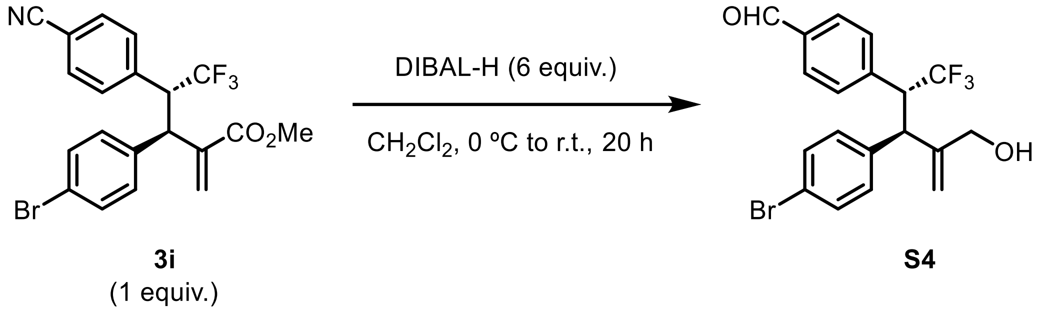

The reaction was carried out adapting the previously described procedure.[12]

A solution of DIBAL-H 1.0M in toluene (1.2 mL, 6 equiv., 1.2 mmol) was added to a solution of **3i** (87.6 mg, 1.0 equiv., 0.2 mmol) in dry CH_2Cl_2 (0.3 M) at 0 ºC. The mixture was stirred at room temperature for 20 h. Subsequently, a saturated solution of the Rochelle salt was added the the reaction and the resultimng solution was stirred for 2h. Then, the mixture was extracted three times with ethyl acetate. The combined organic layers were dried over anhydrous $MgSO_4$, filtered, and concentrated under vacuum. Purification by flash column chromatography on silica gel using *n*-hexane/ethyl acetate as eluent afforded the corresponding reduced product **S4** (62% yield, 98:2 e.r.) as a colourless oil.

4-((2*R*,3*R*)-3-(4-bromophenyl)-1,1,1-trifluoro-4-(hydroxymethyl)pent-4-en-2-yl)benzaldehyde (S4)

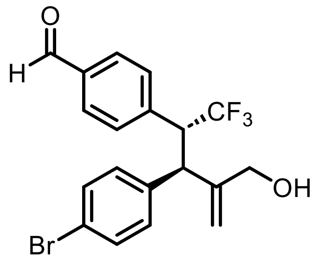

^{1}H NMR (400 MHz, $CDCl_3$) δ 9.90 (s, 1H), 7.70 (d, J = 8.4 Hz, 2H), 7.33 (d, J = 8.1 Hz, 2H), 7.21 (d, J = 8.5 Hz, 2H), 6.95 (d, J = 8.5 Hz, 2H), 5.36 (s, 1H), 5.26 (s, 1H), 4.33 (dq, J = 11.6, 8.1 Hz, 1H), 4.21 (d, J = 11.7 Hz, 1H), 4.18 – 4.04 (m, 2H) ppm; **^{13}C NMR** (101 MHz, $CDCl_3$) δ 191.7, 147.8, 140.9 (q, $^3J_{CF}$ = 2.1 Hz), 138.1, 135.9, 131.7, 130.3, 129.9, 126.3 (q, $^1J_{CF}$ = 281.6 Hz), 121.0, 114.0, 65.1, 52.7 (q, $^2J_{CF}$ = 25.1 Hz), 50.0 ppm; **^{19}F NMR** (376 MHz, $CDCl_3$) δ -63.8 (d, $^3J_{HF}$ = 7.9 Hz, 3F) ppm; **HRMS (ESI)** Calculated for $[C_{19}H_{17}O_2BrF_3]^+$ ($[M+H]^+$) 413.0364. Found 413.0368; **$[\alpha]_D^{25}$** = +7.9 (c = 0.47, $CHCl_3$); **HPLC** Chiralpak Daicel IC (90:10 *n*-Hexane:2-Propanol, 1 mL/min, 254 nm); t_R (major) = 13.6 min, t_R (minor) = 14.8 min (98:2 e.r.).

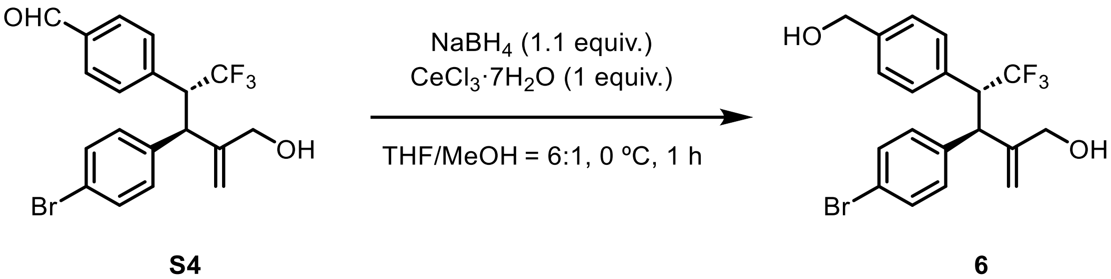

The reaction was carried out by adapting a previously described procedure.[3b]

To a solution of compound **S4** (41.3 mg, 1.0 equiv., 0.1 mmol) in a 2 mL of a mixture THF/MeOH = 6:1 (0.05 M) was added $CeCl_3 \cdot 7H_2O$ (37.3 mg, 1 equiv., 0.1 mmol). The solution was cooled to 0 °C and $NaBH_4$ (4.2 mg, 1.1 equiv., 0.11 mmol) was added. The resulting solution was stirred at 0 °C for 1 h. Then, the reaction mixture was quenched with a saturated solution of NH_4Cl (2 mL) and extracted with CH_2Cl_2 (2 x 2 mL). The organic layers were collected, dried with anhydrous $MgSO_4$ and concentrated under reduced pressure. The resulting crude was purified by flash column chromatography using hexane and ethyl acetate mixtures affording the diol **6** (84% yield, 98:2 e.r.) as a white solid.

(3*R*,4*R*)-3-(4-bromophenyl)-5,5,5-trifluoro-4-(4-(hydroxymethyl)phenyl)-2-methylenepentan-1-ol (6)

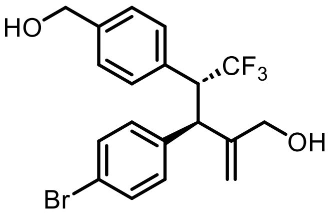

^{1}H NMR (400 MHz, Acetone_d$_6$) δ 7.32 (d, *J* = 8.0 Hz, 2H), 7.29 – 7.22 (m, 4H), 7.19 – 7.15 (m, 2H), 5.43 (dt, *J* = 1.7, 0.9 Hz, 1H), 5.26 (q, *J* = 1.7 Hz, 1H), 4.59 (dq, *J* = 12.2, 8.7 Hz, 1H), 4.49 (d, *J* = 5.9 Hz, 2H), 4.36 (d, *J* = 12.1 Hz, 1H), 4.20 (ddt, *J* = 14.8, 5.8, 1.7 Hz, 1H), 4.09 (t, *J* = 5.9 Hz, 1H), 4.03 (ddt, *J* = 14.8, 5.7, 1.6 Hz, 1H), 3.94 (t, *J* = 5.7 Hz, 1H) ppm; **^{13}C NMR** (101 MHz, Acetone_d$_6$) δ 150.6, 143.0, 141.2, 134.0, 131.7, 130.5, 128.1 (q, $^1J_{CF}$ = 280.9 Hz), 127.1, 120.4, 111.1, 64.1, 63.4, 51.5 (q, $^2J_{CF}$ = 24.5 Hz), 50.2 ppm; **^{19}F NMR** (376 MHz, Acetone_d$_6$) δ -65.1 (d, $^3J_{HF}$ = 8.8 Hz, 3F) ppm; **HRMS (ESI)** Calculated for $[C_{19}H_{18}O_2BrF_3Na]^+$ ($[M+Na]^+$) 437.0334. Found 437.0330; **$[\alpha]_D^{25}$** = +27.0 (c = 1.18, Acetone); **HPLC** Chiralpak Daicel IC (90:10 *n*-Hexane:2-Propanol, 1 mL/min, 225 nm); t_R (major) = 11.9 min, t_R (minor) = 13.1 min (98:2 e.r.).

J.2. 1,3-dipolar cycloaddition – *Isoxazoline*

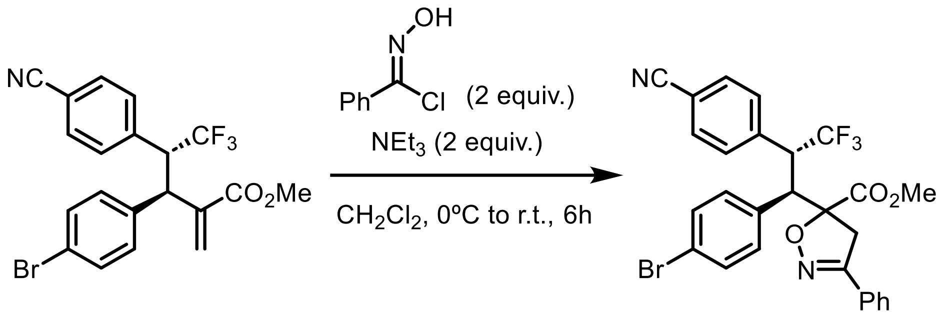

3i
(1 equiv.)

S5

The reaction was carried out by adapting a previously described procedure.[3a]

Triethylamine (28 µL, 2.0 equiv., 0.2 mmol) was added to a solution of chlorobenzaldoxime (31 mg, 2.0 equiv., 0.2 mmol) in dry CH_2Cl_2 (1 mL) at 0 °C. After stirring for 10 min, a solution of **3i** (43.8 mg, 1.0 equiv., 0.1 mmol) in dry CH_2Cl_2 (0.5 mL) was added dropwise. The mixture was stirred at room temperature for 6 h. Then the reaction was quenched with water (3 mL) and the organic layer was separated. The aqueous layer was extracted with CH_2Cl_2 (5 mL). The combined organic layers were dried with anhydrous $MgSO_4$ and concentrated. Chromatography on silica gel *n*-hexane/ethyl acetate afforded the diastereomeric mixture product **S5** as a white foam.

methyl 5-((1*R*,2*R*)-1-(4-bromophenyl)-2-(4-cyanophenyl)-3,3,3-trifluoropropyl)-3-phenyl-4,5-dihydroisoxazole-5-carboxylate

64% yield (36 mg, 0.06 mmol) of a 3:1 diastereomeric ratio.

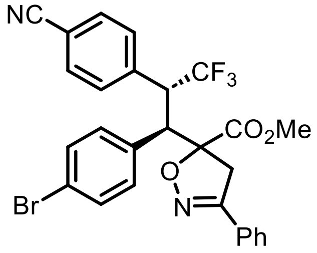

S5 white foam. **^{1}H NMR** (500 MHz, $CDCl_3$) δ 7.79 – 7.71 (m, 2H), 7.56 – 7.44 (m, 3H), 7.41 (d, *J* = 8.1 Hz, 2H), 7.25 (bs, 2H), 6.97 (bs, 2H), 6.84 (bs, 2H), 4.14 (d, *J* = 1.9 Hz, 1H), 3.99 – 3.89 (m, 2H), 3.82 (d, *J* = 18.2 Hz, 1H), 3.41 (s, 3H) ppm; **^{13}C NMR** (126 MHz, $CDCl_3$) δ 171.4, 156.7, 137.5, 134.1, 132.5, 131.6, 131.3, 131.2, 129.2, 128.0, 127.1, 126.7 (q, $^1J_{CF}$ = 280.8 Hz), 122.6, 118.5, 112.2, 91.7, 53.6, 53.0, 50.2, 49.7 (q, $^2J_{CF}$ = 26.2 Hz), 45.2 ppm; **^{19}F NMR** (471 MHz, $CDCl_3$) δ -65.9 (d, $^3J_{HF}$ = 10.3 Hz, 3F) ppm; **HRMS (ESI)** Calculated for $[C_{27}H_{21}O_3N_2BrF_3]^+$ ($[M+H]^+$) 557.0682. Found 557.0688; **$[\alpha]_D^{25}$** = +13.1 (c = 0.46, $CHCl_3$); **HPLC** Phenomenex Lux Cellulose-1 (90:10 *n*-Hexane:2-Propanol, 1 mL/min, 224 nm); t_R (major) = 22.7 min, t_R (minor) = 10.9 min (98:2 e.r.).

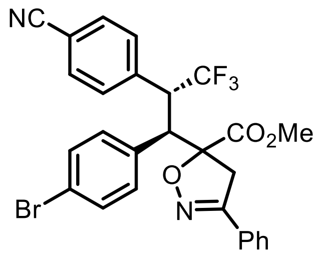

S5', white foam. **^{1}H NMR** (400 MHz, $CDCl_3$) δ 7.56 – 7.42 (m, 5H), 7.42 – 7.30 (m, 3H), 7.26 (bs, 2H), 7.12 (bs, 1H), 6.98 (bs, 1H), 6.80 (bs, 1H), 4.24 – 4.12 (m, 2H), 3.91 (s, 3H), 3.59 (d, J = 17.6 Hz, 1H), 3.03 (d, J = 17.6 Hz, 1H) ppm; **^{13}C NMR** (101 MHz, $CDCl_3$) δ 172.6, 157.2, 137.7, 133.2, 132.6, 131.8, 131.1, 129.0, 127.9, 127.1, 126.9, 126.4 (q, $^1J_{CF}$ = 281.0 Hz), 123.0, 118.5, 112.4, 91.8, 53.8, 51.3 (q, $^2J_{CF}$ = 26.9 Hz), 48.6, 43.8 ppm; **^{19}F NMR** (471 MHz, $CDCl_3$) δ -66.3 (d, $^3J_{HF}$ = 10.1 Hz, 3F) ppm; **HRMS (ESI)** Calculated for $[C_{27}H_{21}O_3N_2BrF_3]^+$ ($[M+H]^+$) 557.0682. Found 557.0689; **$[\alpha]_D^{25}$** = +123.9 (c = 0.44, $CHCl_3$); **HPLC** Chiralpak Daicel IC (90:10 *n*-Hexane:2-Propanol, 1 mL/min, 254 nm); t_R (major) = 13.9 min, t_R (minor) = 16.5 min (98:2 e.r.).

K. References

[1] (a) Y. P. Rey, L. E. Zimmer, C. Sparr, E. Tanzer, W. B. Schweizer, H. M. Senn, S. Lakhdar, R. Gilmour, *Eur. J. Org. Chem.* **2014**, *6*, 1202-1211. (b) C. Cassani, R. Martín-Rapún, E. Arceo, F. Bravo, P. Melchiorre, *Nat. Protoc*. **2013**, *8*, 325-344. (c) M. J. O'Donnell, S. Wu, J. C. Huffman, *Tetrahedron* **1994**, *50*, 4507-4518. (d) G. Pupo, F. Ibba, D. M. H. Ascough, A. C. Vicini, P. Ricci, K. E. Christensen, L. Pfeifer, J. R. Morphy, J. M. Brown, R. S. Paton, V. Gouverneur, *Angew. Chem. Int. Ed*. **2018**, *57*, 638-642. (e) R. D. Aher, A. Ishikawa, M. Yamanaka, F. Tanaka, *J. Org. Chem.* **2022**, *87*, 8151-8157.

[2] (a) M. S. Santos, F. Coelho, *RSC Adv*. **2012**, *2*, 3237-3241. (b) X. Companyó, A. Mazzanti, A. Moyano, A. Janecka, R. Rios, *Chem. Commun*. **2013**, *49*, 1184-1186. (c) X. Companyó, P. Y. Geant, A. Mazzanti, A. Moyano, R. Rios, *Tetrahedron* **2014**, *70*, 75-83. (d) M. A. Helppi, A. L. Lehmkuhler, J. M. Marchi, C. M. Schmidt, M. T. Yip-Schneider, P. V. Ramachandran, *Bioorg. Med. Chem. Lett*. **2015**, *25*, 4270-4273. (e) C. Rasson, A. Stouse, A. Boreux, V. Cirriez, O. Riant, *Chem. Eur. J.* **2018**, *24*, 9234-9237. (f) S. Paria, E. Carletti, M. Marcon, A. Cherubini-Celli, A. Mazzanti, M. Rancan, L. Dell'Amico, M. Bonchio, X. Companyó, *J. Org. Chem.* **2020***, 85,* 4463-4474. (g) N. S. Akins, N. Mishra, H. M. Harris, N. Dudhipala, S. J. Kim, A. W. Keasling, S. Majumdar, J. K. Zjawiony, J. J. Paris, N. M. Ashpole, H. V. Le, *Chem. Med. Chem*. **2022**, *17*, e202100684.

[3] (a) T. Nishimine, K. Fukushi, N. Shibata, H. Taira, E. Tokunaga, A. Yamano, M. Shiro, N. Shibata, *Angew. Chem. Int. Ed.* **2014**, *53*, 517-520. (b) J. Duran, J. Mateos, A. Moyano, X. Companyó, *Chem. Sci.* **2023**, *14*, 7147-7153. (c) T. Nishimine, H. Taira, E. Tokunaga, M. Shiro, N. Shibata, *Angew. Chem. Int. Ed.* **2016**, *55*, 359-363. (d) Y. Zi, M. Lange, C. Schultz, I. Vilotijevic, *Angew. Chem. Int. Ed.* **2019**, *58*, 10727-10731.

[4] (a) J. Liu, L. Yu, C. Zheng, G. Zhao, *Angew. Chem. Int. Ed.* **2021**, *60*, 23641-23645. (b) H. Sakaguchi, Y. Uetake, M. Ohashi, T. Niwa, S. Ogoshi, T. Hosoya, *J. Am. Chem. Soc.* **2017**, *139*, 12855-12862. (c) B. Xiong, T. Wang, H. Sun, Y. Li, S. Kramer, G. Cheng, Z. Lian, *ACS Catal.* **2020**, *10*, 13616-13623. (d) H. Lin, W. Jiao, Z. Chen, J. Han, D. Fang, M. Wang, J. Liao, Org. Lett. **2022**, *24*, 2197-2202. (e) J. Zhang, D. Hu, J. Song, H. Ren, *J. Org. Chem*. **2021**, *86*, 4646-4660. (f) J. Hu, X. Han, Y. Yuan, Z. Shi, *Angew. Chem. Int. Ed*. **2017**, *56*, 13342-13346. (g) H. F. Koch, G. Lodder, J. G. Koch, D. J. Bogdan, G. H. Brown, C. A. Carlson, A. B. Dean, R. Hage, P. Han, J. C. P. Hopman, L. A. James, P. M. Knape, E. C. Roos, M. L. Sardina, R. A. Sawyer, B. O. Scott, C. A. Testa, S. D. Wickham, *J. Am. Chem. Soc.* **1997**, *119*, 9965-9974. (h) H. Tang, L. Lin, C. Feng, T. Loh, *Angew. Chem. Int. Ed*. **2017**, *56*, 9872-9876. (i) L. Yu, M. Tang, C. Si, Z. Meng, Y. Liang, J. Han, X. Sun, *Org. Lett.* **2018**, *20*, 4579-4583. (j) J. Zheng, J. Lin, L. Yu, Y. Wei, X. Zheng, J. Xiao, *Org. Lett.* **2015**, *17*, 6150-6153. (k) H. Li, Y. Qiao, M. Li, F. Wu, H. Wu, J. Chang, D. Bai, *Angew. Chem. Int. Ed.* **2025**, *64*, e202506157.

[5] (a) I. Kuwajima, T. Murofushi, E. Nakamura*, Synthesis* **1976***,* 602-604. (b) J.-I. Hayami, N. Ono, A. Kaji, *Tetrahedron Lett.* **1968***, 9,* 1385. (c) T. Mukaiyama, T. Matsuo, M. Kobayashi, *J. Org. Chem.* **1974***, 39,* 2644-2646. (d) A. Ando, T. Miura, T. Tatematsu, T. Shioiri, *Tetrahedron Lett*. **1993***, 34,* 1507-1510. (e) H. Sun, S. G. Di Magno, *J. Am. Chem. Soc.* **2005***, 127,* 2050-2051. (f) D. C. M. Albanese, M. Penso*, Eur. J. Org. Chem*. **2023***, 26,* e202300224. (g) T. Ooi, K. Maruoka*, Acc. Chem. Res.*

2004, *37,* 526-533. (h) G. Pupo, V. Gouverneur, *J. Am. Chem. Soc.* **2022**, *144,* 5200-5213.

[6] M. J. Frisch, G. W. Trucks, H. B. Schlegel, G. E. Scuseria, M. A. Robb, J. R. Cheeseman, G. Scalmani, V. Barone, B. Mennucci, G. A. Petersson, H. Nakatsuji, M. Caricato, X. Li, H. P. Hratchian, A. F. Izmaylov, J. Bloino, G. Zheng, J. L. Sonnenberg, M. Hada, M. Ehara, K. Toyota, R. Fukuda, J. Hasegawa, M. Ishida, T. Nakajima, Y. Honda, O. Kitao, H. Nakai, T. Vreven, J. A. Montgomery Jr., J. E. Peralta, F. Ogliaro, M. Bearpark, J. J. Heyd, E. Brothers, K. N. Kudin, V. N. Staroverov, T. Keith, R. Kobayashi, J. Normand, K. Raghavachari, A. Rendell, J. C. Burant, S. S. Iyengar, J. Tomasi, M. Cossi, N. Rega, J. M. Millam, M. Klene, J. E. Knox, J. B. Cross, V. Bakken, C. Adamo, J. Jaramillo, R. Gomperts, R. E. Stratmann, O. Yazyev, A. J. Austin, R. Cammi, C. Pomelli, J. W. Ochterski, R. L. Martin, K. Morokuma, V. G. Zakrzewski, G. A. Voth, P. Salvador, J. J. Dannenberg, S. Dapprich, A. D. Daniels, Ö. Farkas, J. B. Foresman, J. V. Ortiz, J. Cioslowski, and D. J. Fox, Gaussian, Inc., Wallingford CT, 2009.

[7] A. D. Becke, *J. Chem. Phys*. **1993**, *98*, 5648-5652.

[8] S. Grimme, S. Ehrlich, L. Goerigk, *J. Comput. Chem.* **2011**, *32*, 1456-1465.

[9] A. V. Marenich, C. J. Cramer, D. G. Truhlar, *J. Phys. Chem. B* **2009**, *113*, 6378-6396.

[10] (a) W. J. Hehre, R. Ditchfield, J. A. Pople, *J. Chem. Phys.* **1972**, *56*, 2257-2261. (b) P. C. Hariharan, J. A. Pople, *Theor. Chim. Acta* **1973**, *28*, 213-222. (c) R. Krishnan, J. S. Binkley, R. Seeger, J. A. Pople, *J. Chem. Phys.* **1980**, *72*, 650-654.

[11] (a) R. A. Kendall, T. H. Dunning Jr., R. J. Harrison, *J. Chem. Phys.* **1992**, *96*, 6796-6806. (b) T. H. Dunning Jr., *J. Chem. Phys.* **1989**, *90*, 1007-1023.

[12] M. Kimura, T. Tamaki, M. Nakata, K. Tohyama, Y. Tamaru, *Angew. Chem. Int. Ed.* **2008**, *47*, 5803-5805.

L. Determination of the absolute configuration. X-ray crystallographic data of diol 6

Crystal obtention

The crystal was obtained by liquid/liquid diffusion (layering technique) from a *n*-hexane/acetone = 5:1 solution of compound **6**.

Sample preparation

A colourless block-like specimen with approximate dimensions 0.258 mm x 0.233 mm x 0.167 mm, was used for the X-ray crystallographic analysis.

Instrument and experimental conditions

The X-ray intensity data were measured at 100K on a D8 Venture system equipped with a multilayer monochromator and a Mo microfocus (λ = 0.71073 Å). The frames were integrated with the Bruker SAINT software package using a narrow-frame algorithm. The integration of the data using a tetragonal unit cell yielded a total of 23092 reflections to a maximum θ angle of 30.57° (0.70 Å resolution), of which 5368 were independent (average redundancy 4.302, completeness = 99.9%, R_{int}= 6.19%, R_{sig} = 5.84%) and 4622 (86.10%) were greater than $2\sigma(F^2)$. The final cell constants of a = 18.5243(9) Å, b = 18.5243(9) Å, c = 10.2463(8) Å, volume = 3516.0(4) Å^3, are based upon the refinement of the XYZ-centroids of reflections above 20 σ(I). Data were corrected for absorption effects using the multi-scan method (SADABS). The calculated minimum and maximum transmission coefficients (based on crystal size) are 0.3449 and 0.7461.

The structure was solved and refined using the Bruker SHELXTL Software Package, using the space group *I*4, with Z = 8 for the formula unit, $C_{19}H_{18}BrF_3O_2$. The final anisotropic full-matrix least-squares refinement on F^2 with 248 variables converged at R1 = 4.16%, for the observed data and wR2 = 11.25% for all data. The goodness-of-fit was 1.056. The largest peak in the final difference electron density synthesis was 1.163 e^-/Å^3 and the largest hole was -1.416 e^-/Å^3 with an RMS deviation of 0.107 e^-/Å^3. On the basis of the final model, the calculated density was 1.569 g/cm^3 and F(000), 1680 e^-.

Table S17. Sample and crystal data for **6**

Identification code	O47ACB211	
Chemical formula	$C_{19}H_{18}BrF_3O_2$	
Formula weight	415.24 g/mol	
Temperature	100(2) K	
Wavelength	0.71073 Å	
Crystal system	tetragonal	
Space group	I 4	
Unit cell dimensions	a = 18.5243(9) Å	α = 90°
	b = 18.5243(9) Å	β = 90°
	c = 10.2463(8) Å	γ = 90°
Volume	3516.0(4) $Å^3$	
Z	8	
Density (calculated)	1.569 g/cm^3	
Absorption coefficient	2.378 mm^{-1}	
F(000)	1680	

Table S18. Data collection and structure refinement for **6**

Theta range for data collection	2.20 to 30.57	
Index ranges	-26<=h<=22, -26<=k<=25, -14<=l<=14	
Reflections collected	23092	
Independent reflections	5368 [R(int) = 0.0619]	
Refinement method	Full-matrix least-squares on F^2	
Refinement program	SHELXL-2019/1 (Sheldrick, 2019)	
Function minimized	$\Sigma w(F_o^2 - F_c^2)^2$	
Data / restraints / parameters	5368 / 3 / 248	
Goodness-of-fit on F^2	1.056	
Final R indices	4622 data; I>2σ(I)	R1 = 0.0416, wR2 = 0.1069
	all data	R1 = 0.0494, wR2 = 0.1125
Weighting scheme	$w=1/[\sigma^2(F_o^2)+(0.0661P)^2]$<br>where $P=(F_o^2+2F_c^2)/3$	
Absolute structure parameter	0.122(7)	
Largest diff. peak and hole	1.163 and -1.416 $eÅ^{-3}$	
R.M.S. deviation from mean	0.107 $eÅ^{-3}$	

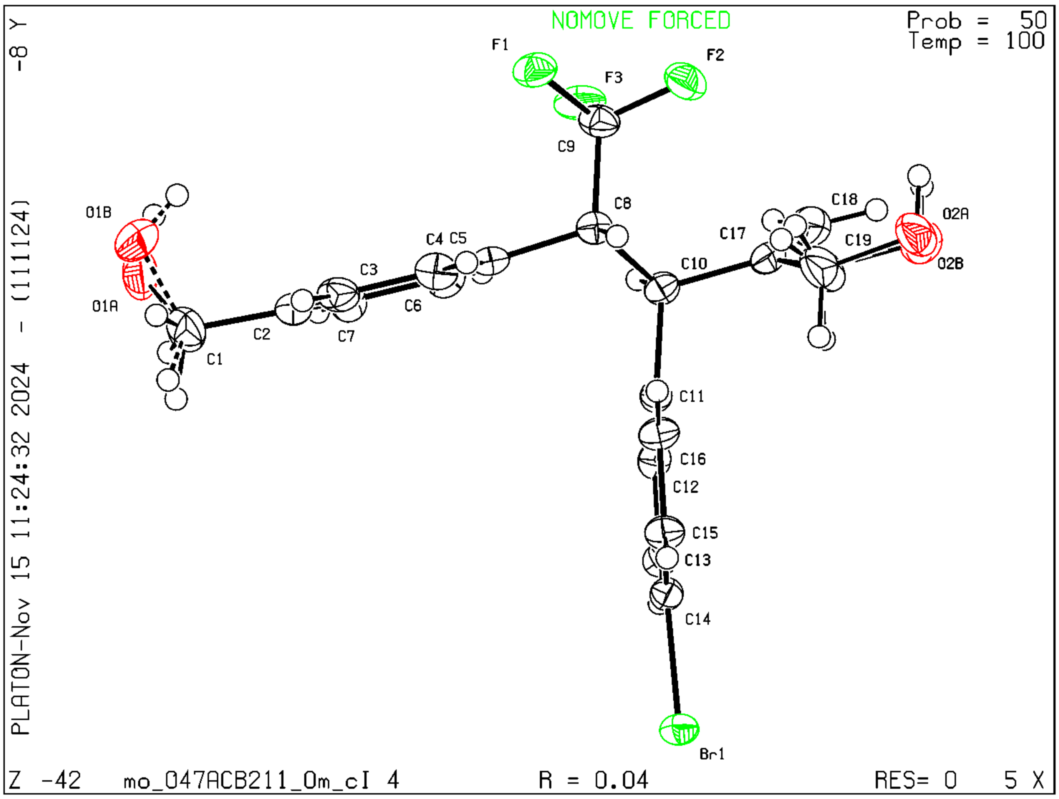

Figure S24. ORTEP diagram and atomic numbering of **6**. Thermal ellipsoids drawn at the 50% probability level.

C8	Chiral: R
C10	Chiral: R

Figure S19. Atomic coordinates and equivalent isotropic atomic displacement parameters (Å^2) for **6**.

U(eq) is defined as one third of the trace of the orthogonalized U_{ij} tensor.

	x/a	y/b	z/c	U(eq)
O1A	0.9648(5)	0.9025(5)	0.4405(12)	0.072(3)
O1B	0.9699(9)	0.9164(7)	0.572(3)	0.133(11)
C1	0.9556(3)	0.8440(3)	0.5268(5)	0.0423(13)
C2	0.0243(2)	0.8010(2)	0.5443(4)	0.0291(8)
C3	0.0475(2)	0.7804(2)	0.6666(4)	0.0301(8)
C4	0.1127(2)	0.7437(2)	0.6831(4)	0.0284(8)
C5	0.1563(2)	0.7282(2)	0.5763(3)	0.0222(7)
C6	0.1325(3)	0.7476(2)	0.4530(4)	0.0289(8)
C7	0.0672(2)	0.7839(2)	0.4372(4)	0.0306(8)
C8	0.2297(2)	0.6938(2)	0.5962(3)	0.0226(7)
C9	0.2875(2)	0.7518(2)	0.5867(4)	0.0276(7)
C10	0.24426(17)	0.63136(16)	0.4976(5)	0.0225(5)
C11	0.18594(16)	0.57361(16)	0.5023(5)	0.0217(5)
C12	0.1614(2)	0.5444(2)	0.3853(4)	0.0232(8)
C13	0.1140(2)	0.4858(2)	0.3829(4)	0.0225(8)
C14	0.09112(16)	0.45718(16)	0.5018(5)	0.0223(5)
C15	0.1122(2)	0.4865(3)	0.6192(4)	0.0252(9)
C16	0.1593(3)	0.5448(2)	0.6193(4)	0.0248(8)
C17	0.3182(2)	0.59495(18)	0.5083(6)	0.0315(8)
C18	0.3651(3)	0.5968(3)	0.4104(6)	0.0442(12)
O2A	0.4033(8)	0.5219(10)	0.6156(15)	0.058(5)
O2B	0.4002(7)	0.5258(10)	0.6689(14)	0.047(3)
C19	0.3331(3)	0.5551(3)	0.6343(7)	0.0577(17)
Br1	0.02949(2)	0.37491(2)	0.49992(8)	0.02620(12)
F1	0.27163(14)	0.80866(14)	0.6643(3)	0.0320(5)
F2	0.35283(14)	0.72919(16)	0.6261(3)	0.0370(6)
F3	0.29543(16)	0.77815(15)	0.4657(2)	0.0351(6)

Figure S20. Bond lengths (Å) for **6**

O1A-C1	1.409(10)	O1A-H1A	0.840000
O1B-C1	1.444(11)	O1B-H1B	0.840000
C1-C2	1.512(6)	C1-H1AA	0.990000
C1-H1AB	0.990000	C1-H1BA	0.990000
C1-H1BB	0.990000	C2-C3	1.378(6)

C2-C7	1.391(6)	C3-C4	1.396(6)
C3-H3	0.950000	C4-C5	1.390(5)
C4-H4	0.950000	C5-C6	1.386(5)
C5-C8	1.514(5)	C6-C7	1.394(6)
C6-H6	0.950000	C7-H7	0.950000
C8-C9	1.521(5)	C8-C10	1.559(5)
C8-H8	1.000000	C9-F3	1.340(5)
C9-F2	1.344(5)	C9-F1	1.351(5)
C10-C11	1.521(4)	C10-C17	1.531(5)
C10-H10	1.000000	C11-C12	1.391(6)
C11-C16	1.402(6)	C12-C13	1.397(6)
C12-H12	0.950000	C13-C14	1.394(6)
C13-H13	0.950000	C14-C15	1.376(6)
C14-Br1	1.904(3)	C15-C16	1.389(7)
C15-H15	0.950000	C16-H16	0.950000
C17-C18	1.328(7)	C17-C19	1.512(8)
C18-H18A	0.950000	C18-H18B	0.950000
O2A-C19	1.450(12)	O2A-H2A	0.840000
O2B-C19	1.401(15)	O2B-H2B	0.840000
C19-H19A	0.990000	C19-H19B	0.990000
C19-H19C	0.990000	C19-H19D	0.990000

Figure S21. Bond angles (°) for **6**

C1-O1A-H1A	109.500000	C1-O1B-H1B	109.500000
O1A-C1-C2	112.2(5)	O1B-C1-C2	107.3(8)
O1A-C1-H1AA	109.200000	C2-C1-H1AA	109.200000
O1A-C1-H1AB	109.200000	C2-C1-H1AB	109.200000
H1AA-C1-H1AB	107.900000	O1B-C1-H1BA	110.300000
C2-C1-H1BA	110.300000	O1B-C1-H1BB	110.300000
C2-C1-H1BB	110.300000	H1BA-C1-H1BB	108.500000
C3-C2-C7	118.4(4)	C3-C2-C1	121.1(4)
C7-C2-C1	120.4(4)	C2-C3-C4	121.0(4)
C2-C3-H3	119.500000	C4-C3-H3	119.500000
C5-C4-C3	120.5(4)	C5-C4-H4	119.700000
C3-C4-H4	119.700000	C6-C5-C4	118.6(4)
C6-C5-C8	121.1(3)	C4-C5-C8	120.2(3)
C5-C6-C7	120.5(4)	C5-C6-H6	119.800000
C7-C6-H6	119.800000	C2-C7-C6	120.9(4)
C2-C7-H7	119.500000	C6-C7-H7	119.500000

C5-C8-C9	109.0(3)	C5-C8-C10	112.3(3)
C9-C8-C10	111.1(3)	C5-C8-H8	108.100000
C9-C8-H8	108.100000	C10-C8-H8	108.100000
F3-C9-F2	107.0(3)	F3-C9-F1	106.5(3)
F2-C9-F1	105.2(3)	F3-C9-C8	113.2(3)
F2-C9-C8	113.2(3)	F1-C9-C8	111.1(3)
C11-C10-C17	108.9(3)	C11-C10-C8	112.2(3)
C17-C10-C8	115.8(4)	C11-C10-H10	106.400000
C17-C10-H10	106.400000	C8-C10-H10	106.400000
C12-C11-C16	118.3(3)	C12-C11-C10	118.6(4)
C16-C11-C10	123.0(4)	C11-C12-C13	121.5(3)
C11-C12-H12	119.200000	C13-C12-H12	119.200000
C14-C13-C12	118.1(3)	C14-C13-H13	120.900000
C12-C13-H13	120.900000	C15-C14-C13	121.8(3)
C15-C14-Br1	119.6(3)	C13-C14-Br1	118.5(3)
C14-C15-C16	119.0(3)	C14-C15-H15	120.500000
C16-C15-H15	120.500000	C15-C16-C11	121.1(4)
C15-C16-H16	119.400000	C11-C16-H16	119.400000
C18-C17-C19	122.6(5)	C18-C17-C10	121.3(5)
C19-C17-C10	116.1(5)	C17-C18-H18A	120.000000
C17-C18-H18B	120.000000	H18A-C18-H18B	120.000000
C19-O2A-H2A	109.500000	C19-O2B-H2B	109.500000
O2B-C19-C17	124.6(8)	O2A-C19-C17	105.0(8)
O2A-C19-H19A	110.800000	C17-C19-H19A	110.800000
O2A-C19-H19B	110.800000	C17-C19-H19B	110.800000
H19A-C19-H19B	108.800000	O2B-C19-H19C	106.200000
C17-C19-H19C	106.200000	O2B-C19-H19D	106.200000
C17-C19-H19D	106.200000	H19C-C19-H19D	106.400000

Figure S22. Torsion angles (°) for **6**

O1A-C1-C2-C3	134.0(7)	O1B-C1-C2-C3	72.6(15)
O1A-C1-C2-C7	-43.5(8)	O1B-C1-C2-C7	-104.8(15)
C7-C2-C3-C4	0.6(6)	C1-C2-C3-C4	-176.9(4)
C2-C3-C4-C5	0.9(6)	C3-C4-C5-C6	-2.1(6)
C3-C4-C5-C8	175.1(4)	C4-C5-C6-C7	1.8(6)
C8-C5-C6-C7	-175.4(4)	C3-C2-C7-C6	-0.9(6)
C1-C2-C7-C6	176.6(4)	C5-C6-C7-C2	-0.3(6)
C6-C5-C8-C9	76.6(4)	C4-C5-C8-C9	-100.5(4)
C6-C5-C8-C10	-47.0(5)	C4-C5-C8-C10	135.8(3)

C5-C8-C9-F3	-69.3(4)	C10-C8-C9-F3	55.1(4)
C5-C8-C9-F2	168.6(3)	C10-C8-C9-F2	-67.0(4)
C5-C8-C9-F1	50.5(4)	C10-C8-C9-F1	174.9(3)
C5-C8-C10-C11	-55.8(4)	C9-C8-C10-C11	-178.3(3)
C5-C8-C10-C17	178.4(3)	C9-C8-C10-C17	55.9(4)
C17-C10-C11-C12	-91.9(5)	C8-C10-C11-C12	138.5(4)
C17-C10-C11-C16	83.4(5)	C8-C10-C11-C16	-46.1(5)
C16-C11-C12-C13	-2.9(5)	C10-C11-C12-C13	172.8(3)
C11-C12-C13-C14	0.5(6)	C12-C13-C14-C15	2.0(5)
C12-C13-C14-Br1	-177.6(3)	C13-C14-C15-C16	-2.0(5)
Br1-C14-C15-C16	177.6(3)	C14-C15-C16-C11	-0.5(6)
C12-C11-C16-C15	2.9(5)	C10-C11-C16-C15	-172.5(4)
C11-C10-C17-C18	114.8(5)	C8-C10-C17-C18	-117.7(5)
C11-C10-C17-C19	-63.2(6)	C8-C10-C17-C19	64.3(5)
C18-C17-C19-O2B	9.9(13)	C10-C17-C19-O2B	-172.1(11)
C18-C17-C19-O2A	-1.9(10)	C10-C17-C19-O2A	176.1(8)

Figure S23. Anisotropic atomic displacement parameters (Å^2) for **6**

The anisotropic atomic displacement factor exponent takes the form: $-2\pi^2[h^2 a^{*2} U_{11} + ... + 2 h k a^* b^* U_{12}]$

	U_{11}	U_{22}	U_{33}	U_{23}	U_{13}	U_{12}
O1A	0.029(4)	0.037(4)	0.150(8)	0.025(5)	0.015(5)	0.008(3)
O1B	0.043(8)	0.024(6)	0.33(3)	-0.002(12)	-0.006(15)	-0.003(5)
C1	0.0255(19)	0.040(2)	0.061(4)	0.009(2)	0.0091(19)	0.0018(17)
C2	0.0232(18)	0.0228(18)	0.041(2)	0.0014(14)	0.0001(14)	-0.0037(15)
C3	0.0274(19)	0.0301(19)	0.0327(19)	-0.0020(16)	0.0074(15)	-0.0070(16)
C4	0.0304(19)	0.033(2)	0.0222(16)	-0.0010(14)	0.0027(14)	-0.0051(16)
C5	0.0266(18)	0.0195(16)	0.0206(16)	-0.0005(12)	-0.0008(13)	-0.0065(14)
C6	0.036(2)	0.029(2)	0.0220(16)	0.0004(14)	0.0003(15)	0.0016(17)
C7	0.031(2)	0.032(2)	0.0284(19)	0.0070(15)	-0.0013(15)	0.0021(17)
C8	0.0250(17)	0.0233(16)	0.0196(16)	0.0007(12)	-0.0012(12)	-0.0025(13)
C9	0.0261(18)	0.0305(19)	0.0261(17)	-0.0065(14)	-0.0006(14)	-0.0057(15)
C10	0.0253(14)	0.0205(13)	0.0218(13)	0.0031(18)	0.0018(18)	-0.0021(11)
C11	0.0208(13)	0.0203(12)	0.0241(13)	0.0018(18)	0.0023(18)	0.0003(10)
C12	0.025(2)	0.023(2)	0.0213(19)	0.0030(14)	0.0029(15)	0.0024(16)
C13	0.024(2)	0.0220(19)	0.0219(19)	-0.0003(15)	0.0030(14)	-0.0006(15)
C14	0.0214(13)	0.0209(13)	0.0247(13)	0.0010(19)	0.0035(18)	-0.0006(10)
C15	0.027(2)	0.026(2)	0.0226(19)	0.0030(16)	0.0046(16)	-0.0054(16)
C16	0.030(2)	0.024(2)	0.0204(18)	0.0006(14)	0.0018(16)	-0.0056(18)

C17	0.0267(16)	0.0178(14)	0.050(2)	-0.001(2)	0.003(2)	-0.0021(12)
C18	0.032(2)	0.035(2)	0.066(3)	-0.017(2)	0.008(2)	-0.0010(19)
O2A	0.049(7)	0.030(4)	0.095(13)	0.015(8)	-0.038(8)	0.002(4)
O2B	0.028(4)	0.041(6)	0.072(9)	0.024(7)	-0.014(5)	-0.002(3)
C19	0.031(2)	0.048(3)	0.095(5)	0.036(3)	-0.020(3)	-0.006(2)
Br1	0.02607(17)	0.02355(17)	0.02899(18)	0.0001(2)	0.0014(2)	-0.00599(11)
F1	0.0357(13)	0.0284(12)	0.0319(12)	-0.0093(9)	-0.0003(10)	-0.0060(10)
F2	0.0252(12)	0.0409(14)	0.0450(14)	-0.0116(11)	-0.0060(10)	-0.0025(11)
F3	0.0418(14)	0.0345(13)	0.0289(13)	-0.0014(9)	0.0057(9)	-0.0159(11)

Figure S24. Hydrogen atomic coordinates and isotropic atomic displacement parameters (Å^2) for **6**.

	**x/a**	**y/b**	**z/c**	**U(eq)**
H1A	0.0004	0.9273	0.4643	0.108000
H1B	0.0141	0.9207	0.5891	0.199000
H1AA	-0.0827	0.8118	0.4926	0.051000
H1AB	-0.0604	0.8624	0.6127	0.051000
H1BA	-0.0586	0.8447	0.4336	0.051000
H1BB	-0.0841	0.8221	0.5780	0.051000
H3	0.0187	0.7912	0.7407	0.036000
H4	0.1274	0.7293	0.7680	0.034000
H6	0.1610	0.7361	0.3787	0.035000
H7	0.0517	0.7970	0.3521	0.037000
H8	0.2312	0.6732	0.6864	0.027000
H10	0.2419	0.6533	0.4085	0.027000
H12	0.1773	0.5648	0.3052	0.028000
H13	0.0978	0.4660	0.3026	0.027000
H15	0.0947	0.4671	0.6990	0.030000
H16	0.1738	0.5655	0.7000	0.030000
H18A	0.4101	0.5725	0.4178	0.053000
H18B	0.3537	0.6225	0.3328	0.053000
H2A	0.4336	0.5538	0.5961	0.087000
H2B	0.4322	0.5577	0.6625	0.070000
H19A	0.3341	0.5889	0.7091	0.069000
H19B	0.2958	0.5179	0.6504	0.069000
H19C	0.3197	0.5885	0.7057	0.069000
H19D	0.2982	0.5147	0.6376	0.069000

Figure S25. Hydrogen Bonds (Angstrom, Deg) for **6**.

Donor – H..Aceptor [ARU]	D – H	H…A	D…A	D-H…A
O1A -- H1A .. O1A [1-y,1+x,z]	0.8400	1.9400	2.716(13)	152.00
O1A -- H1A .. O1B [1-y,1+x,z]	0.8400	2.0500	2.87(2)	163.00
O2A -- H2A .. O2A [y,1-x,z]	0.8400	1.8300	2.60(2)	151.00
O2A -- H2A .. O2B [y,1-x,z]	0.8400	2.0500	2.74(2)	140.00
O2B -- H2B .. O2A [y,1-x,z]	0.8400	1.8700	2.67(2)	157.00
O2B -- H2B .. O2B [y,1-x,z]	0.8400	1.9000	2.70(2)	158.00
C4 -- H4 .. F3 [1/2-x,3/2-y,1/2+z]	0.9500	2.4800	3.383(5)	158.00
C18 -- H18A .. O2A	0.9500	2.2400	2.617(17)	103.00

M. NMR Spectra

M.1. NMR spectra of gem-difluoroalkenes 2

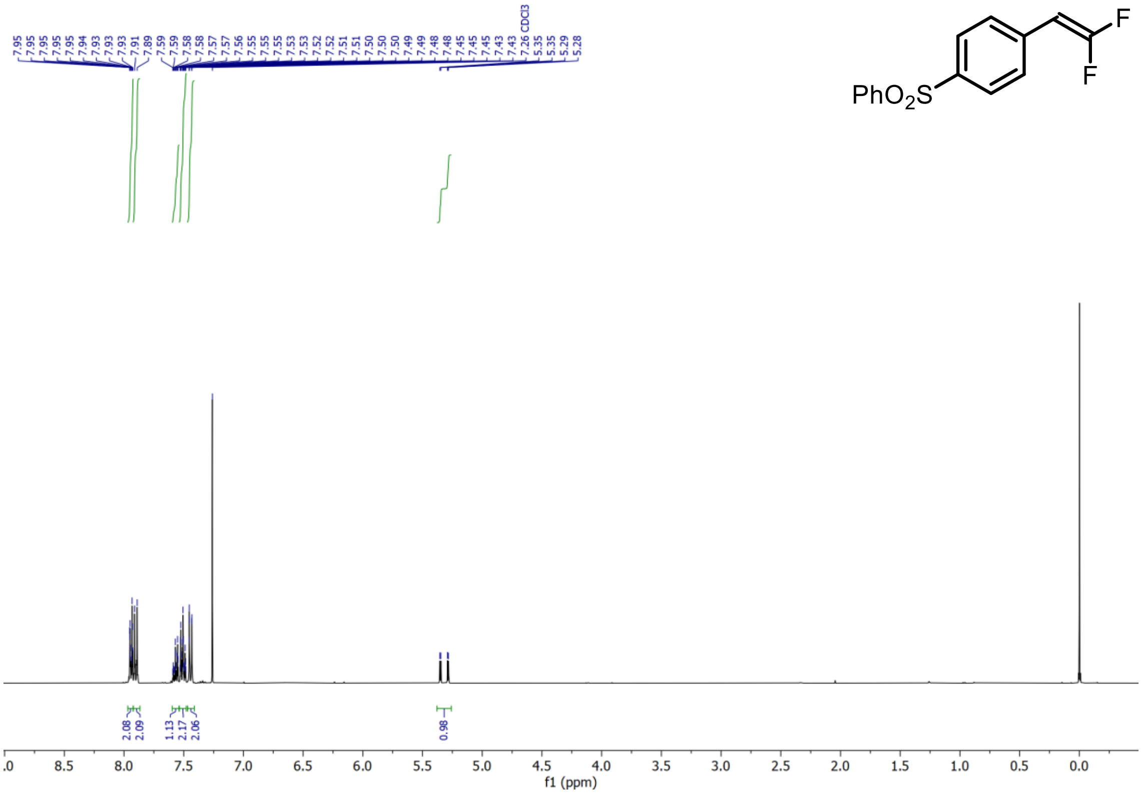

^{1}H NMR (400 MHz, CDCl$_3$) spectra of **2f**.

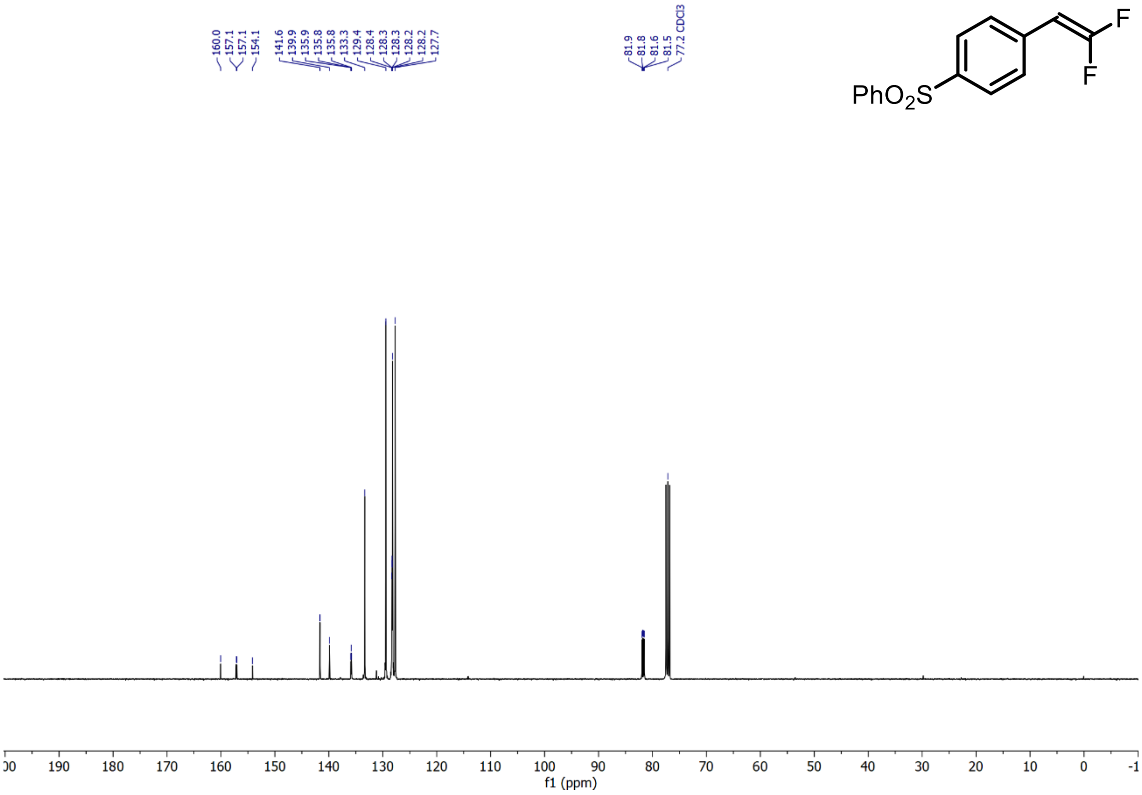

^{13}C NMR (101 MHz, CDCl$_3$) spectra of **2f**.

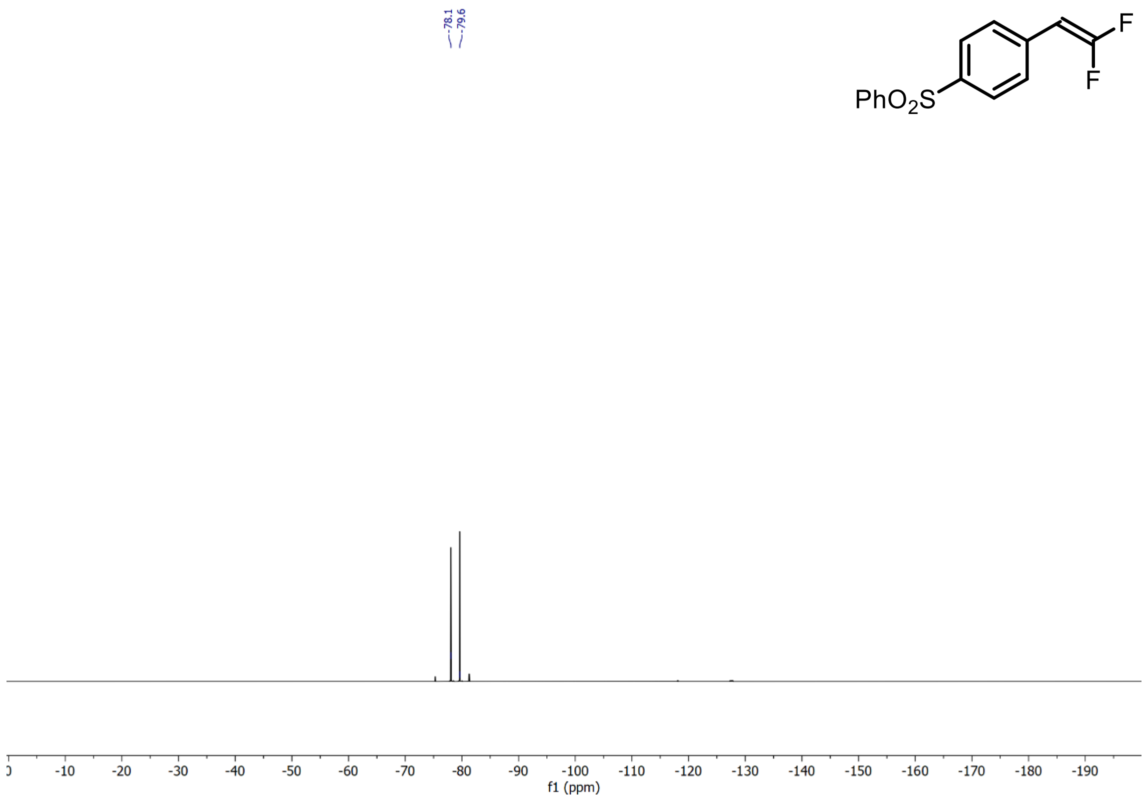

^{19}F NMR (471 MHz, $CDCl_3$) spectra of **2f**.

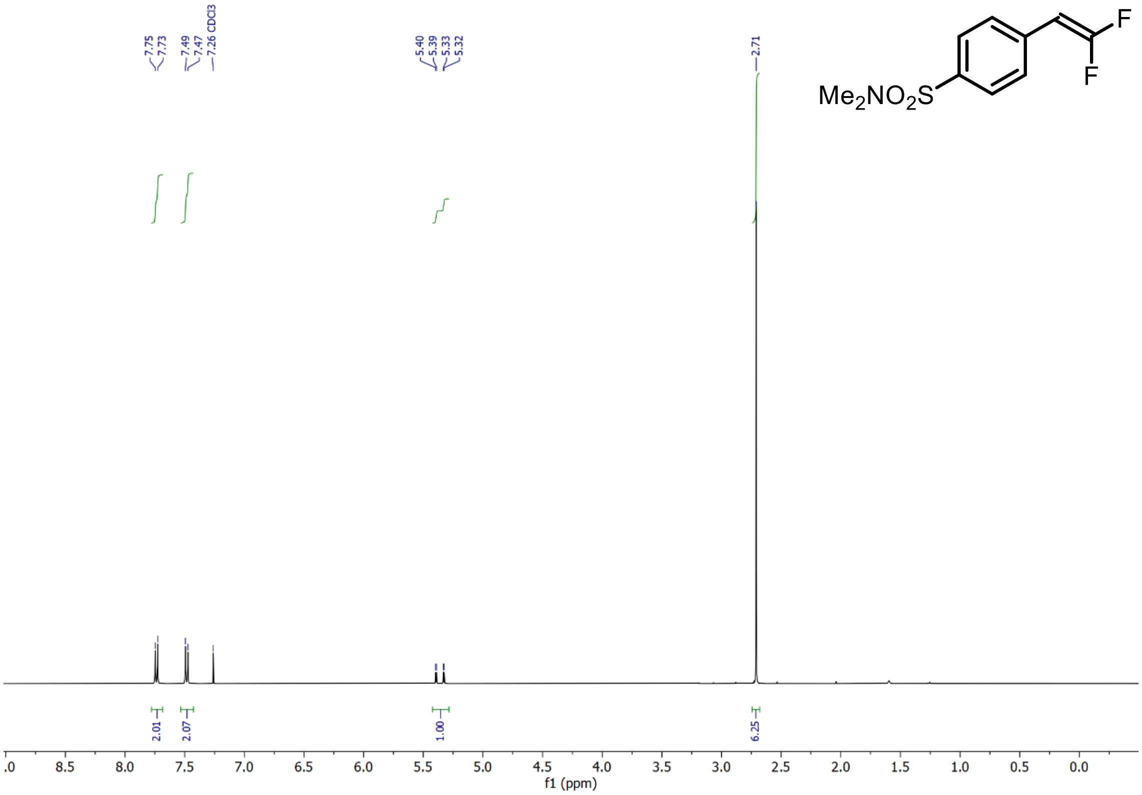

^{1}H NMR (400 MHz, $CDCl_3$) spectra of **2g**.

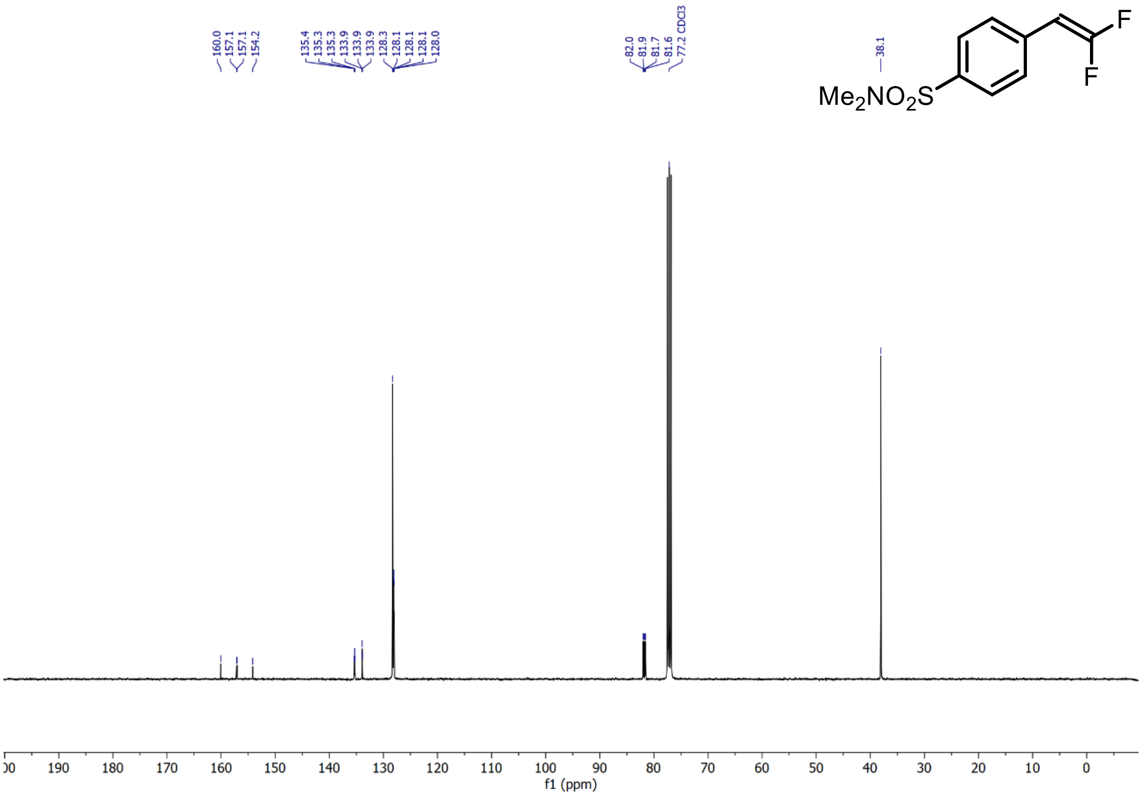

^{13}C NMR (101 MHz, $CDCl_3$) spectra of **2g**.

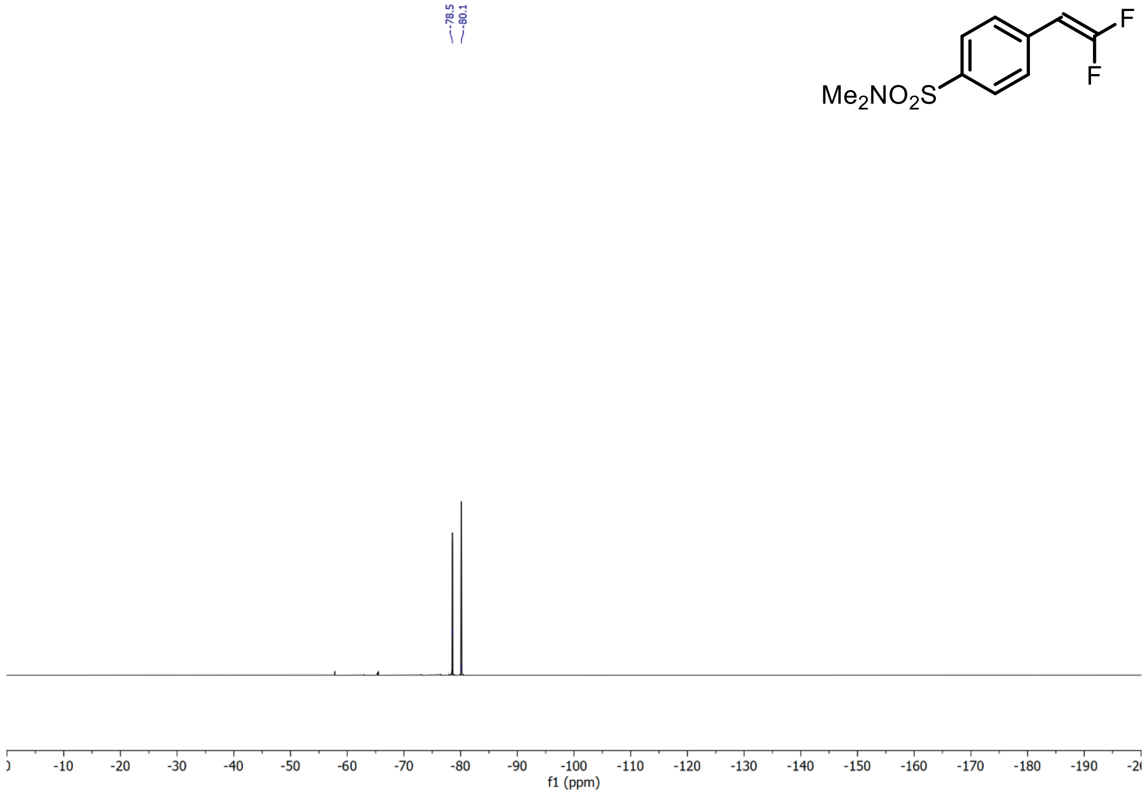

^{19}F NMR (471 MHz, $CDCl_3$) spectra of **2g**.

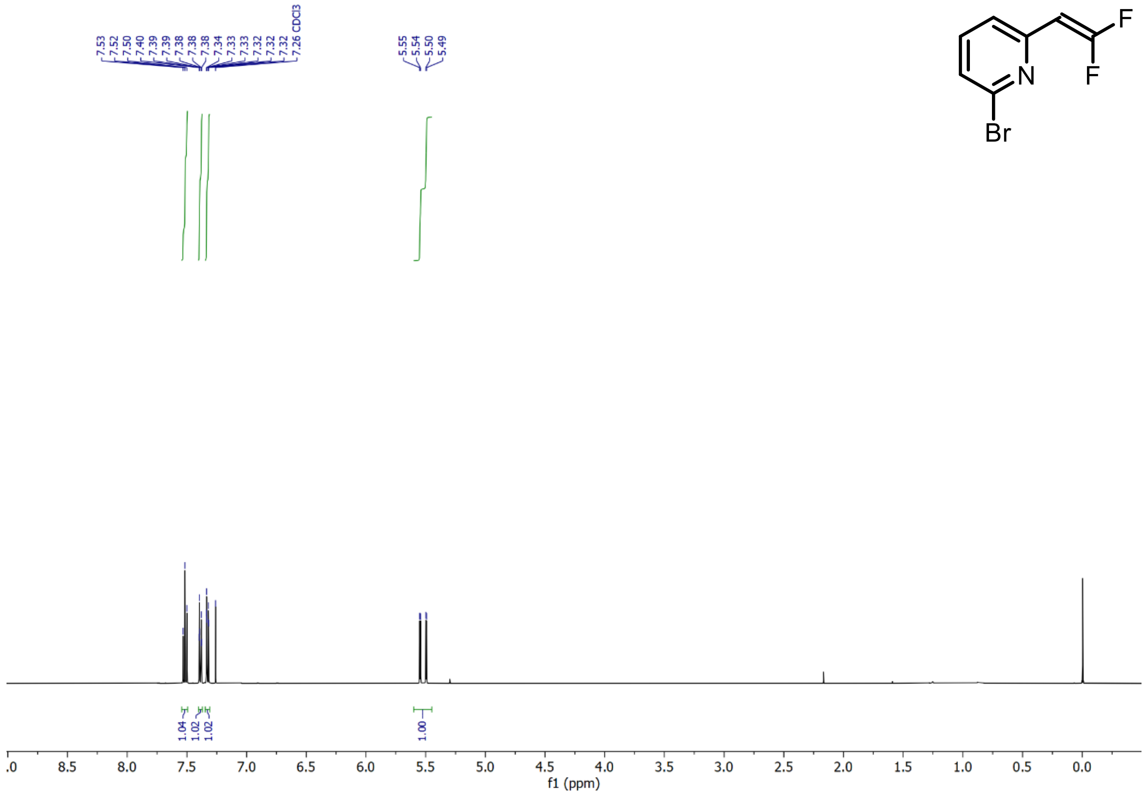

^{1}H NMR (500 MHz, $CDCl_3$) spectra of **2k**.

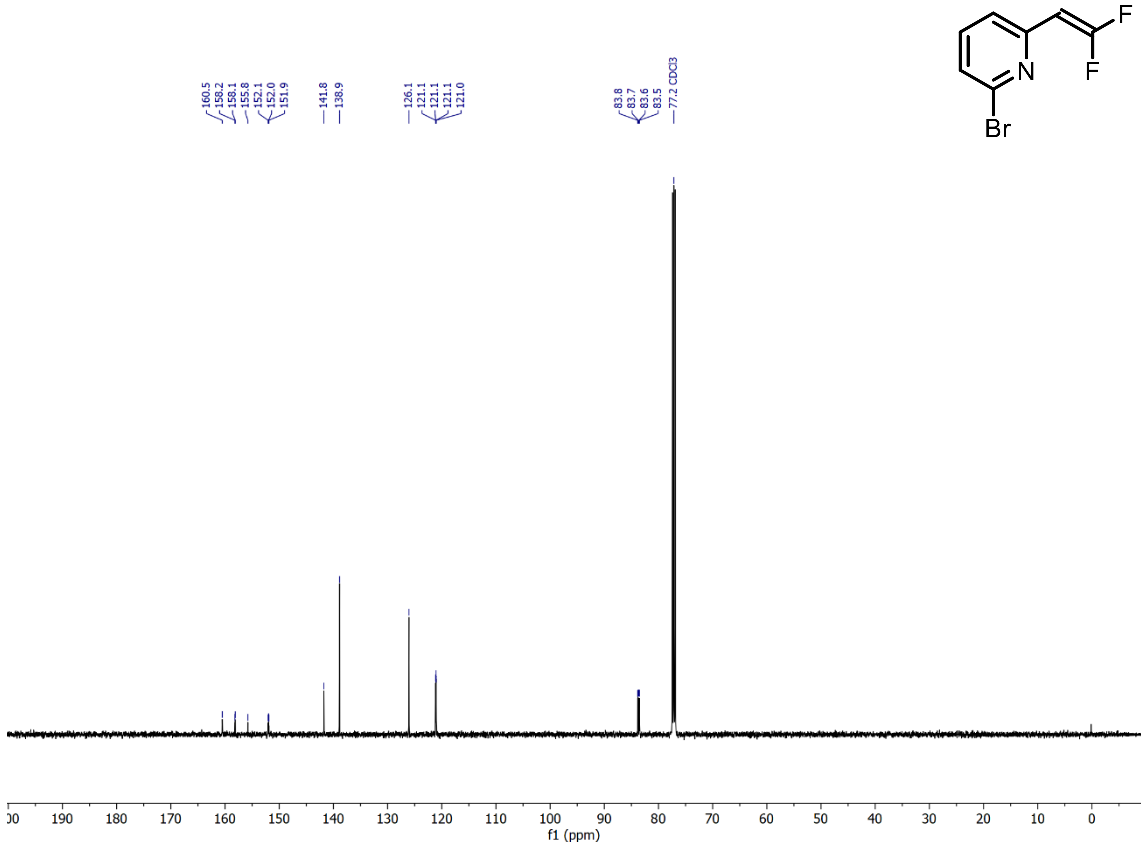

^{13}C NMR (126 MHz, $CDCl_3$) spectra of **2k**.

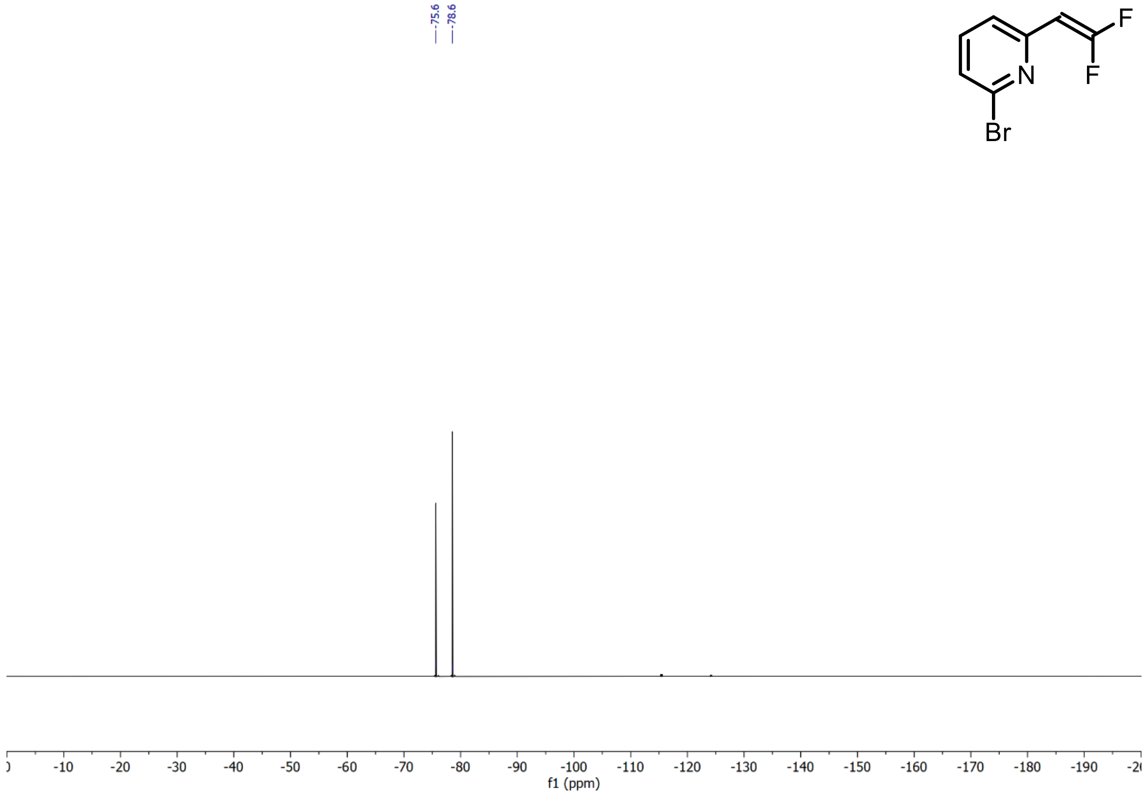

^{19}F NMR (471 MHz, $CDCl_3$) spectra of **2k**.

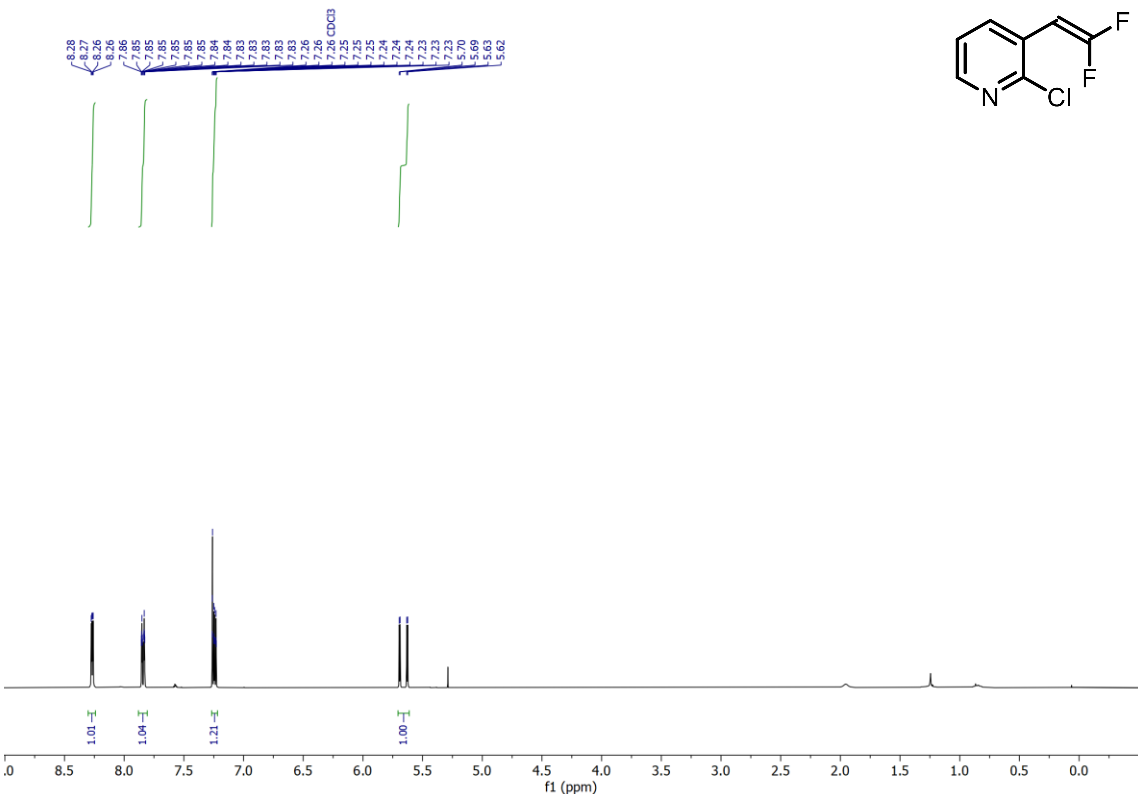

^{1}H NMR (400 MHz, $CDCl_3$) spectra of **2l**.

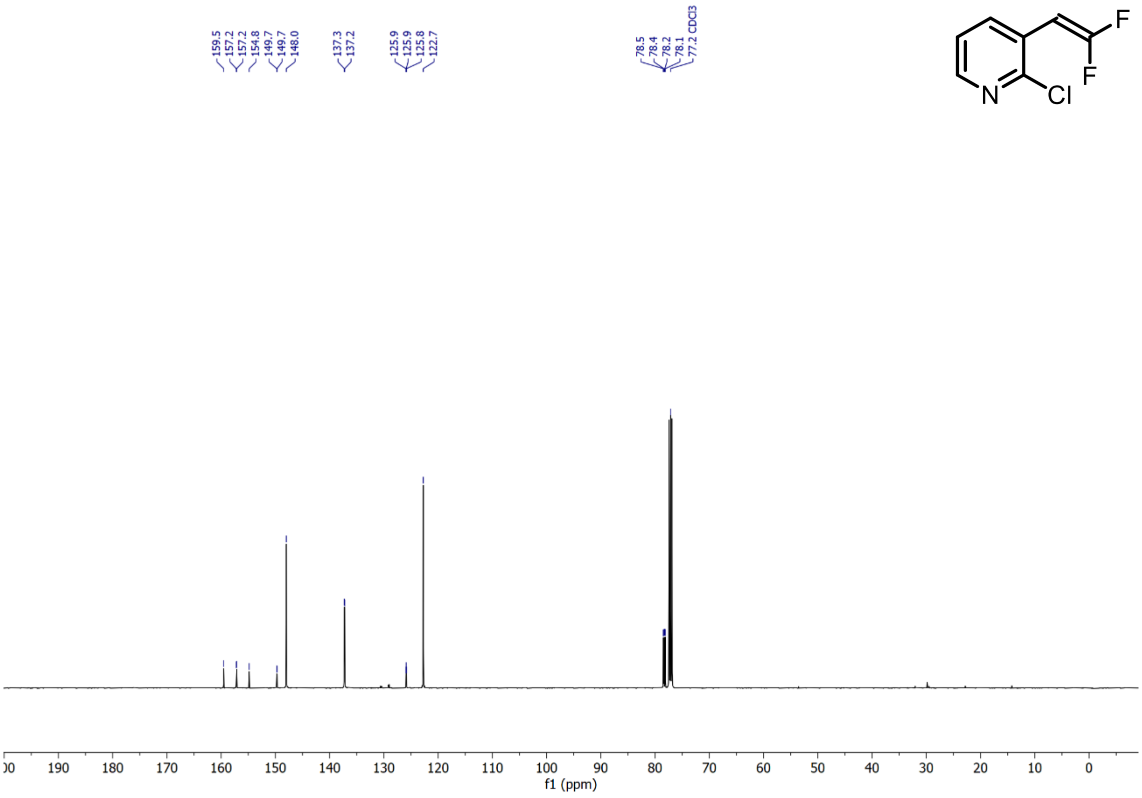

^{13}C NMR (126 MHz, $CDCl_3$) spectra of **2l**.

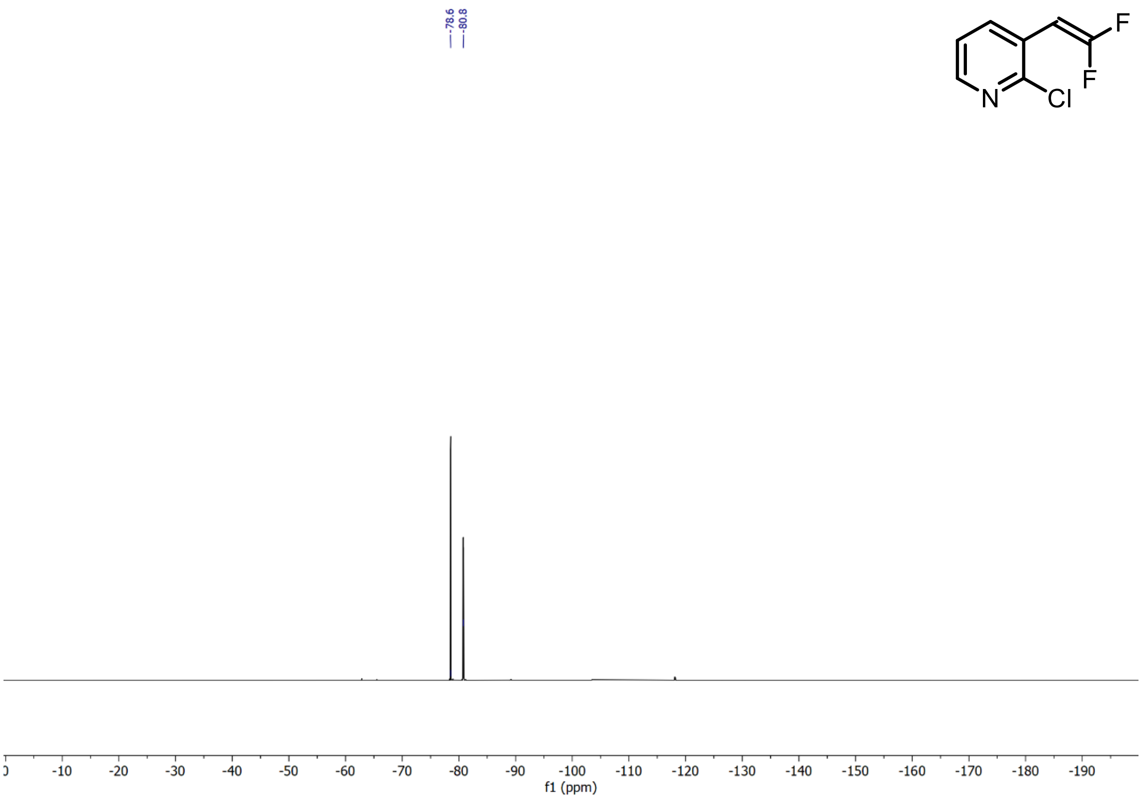

^{19}F NMR (471 MHz, $CDCl_3$) spectra of **2l**.

M.2. NMR spectra of γ-alkylation products 3

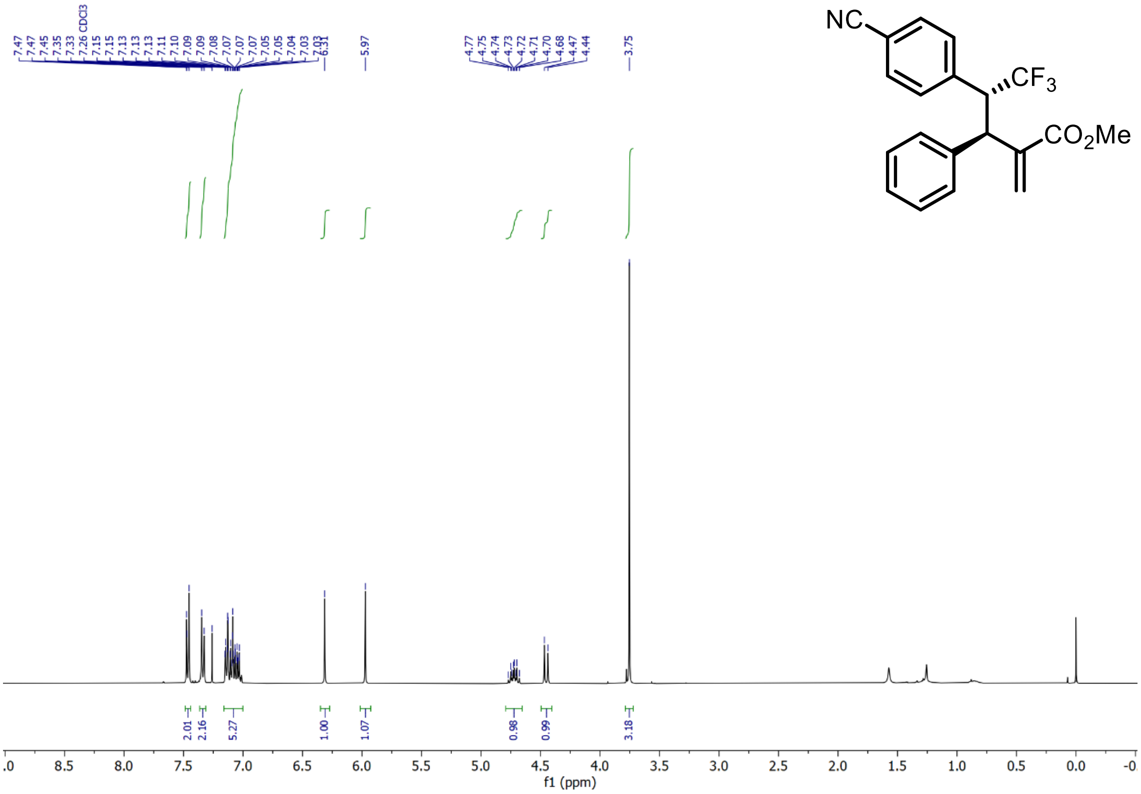

^{1}H NMR (400 MHz, $CDCl_3$) spectra of **3a**.

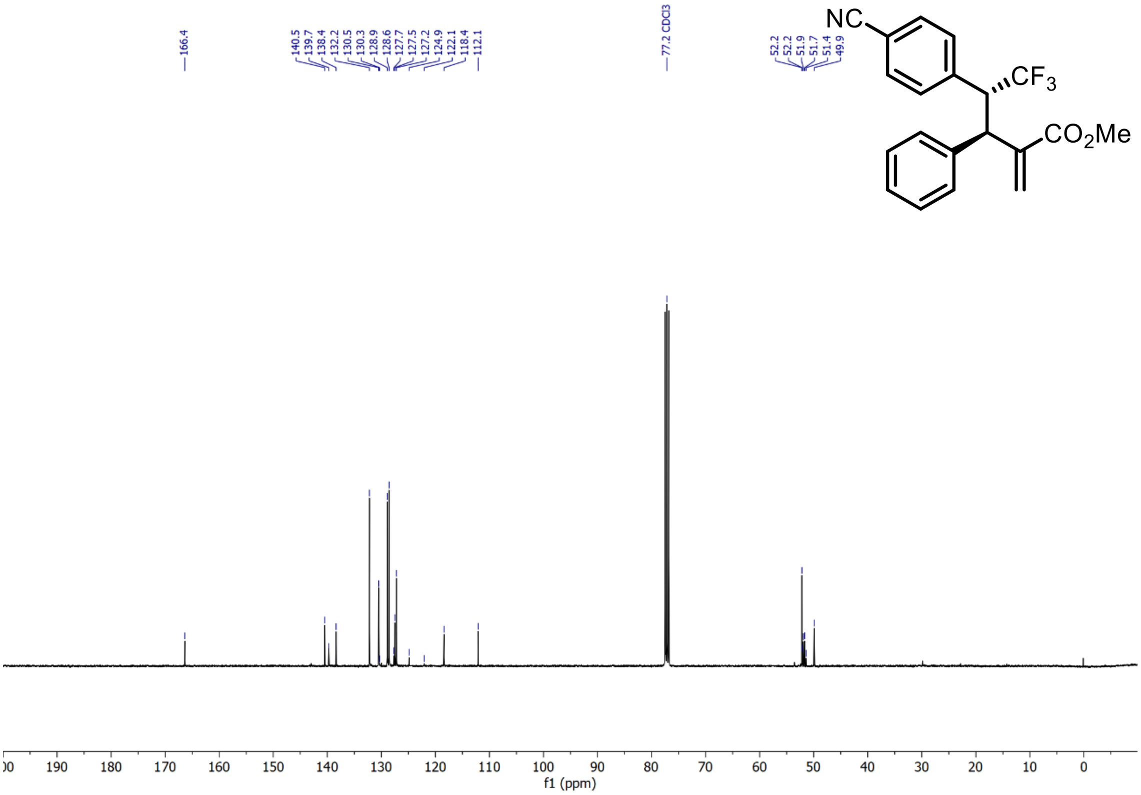

^{13}C NMR (101 MHz, $CDCl_3$) spectra of **3a**.

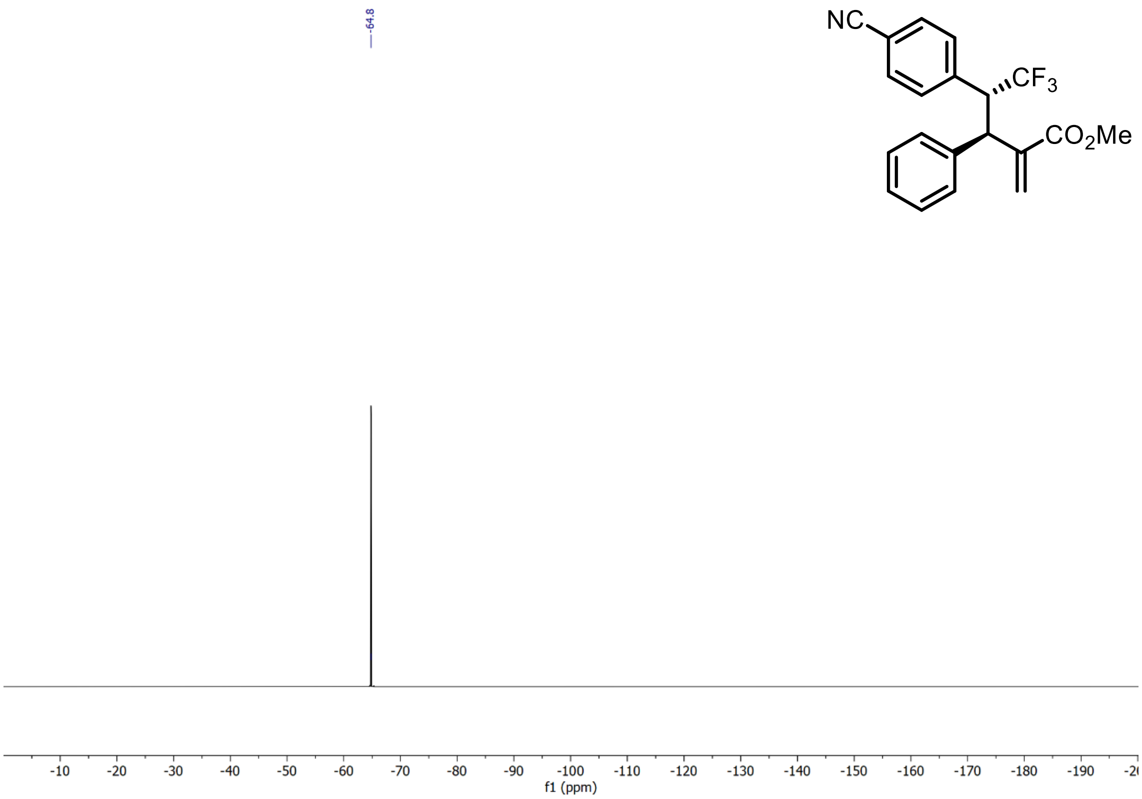

^{19}F NMR (376 MHz, $CDCl_3$) spectra of **3a**.

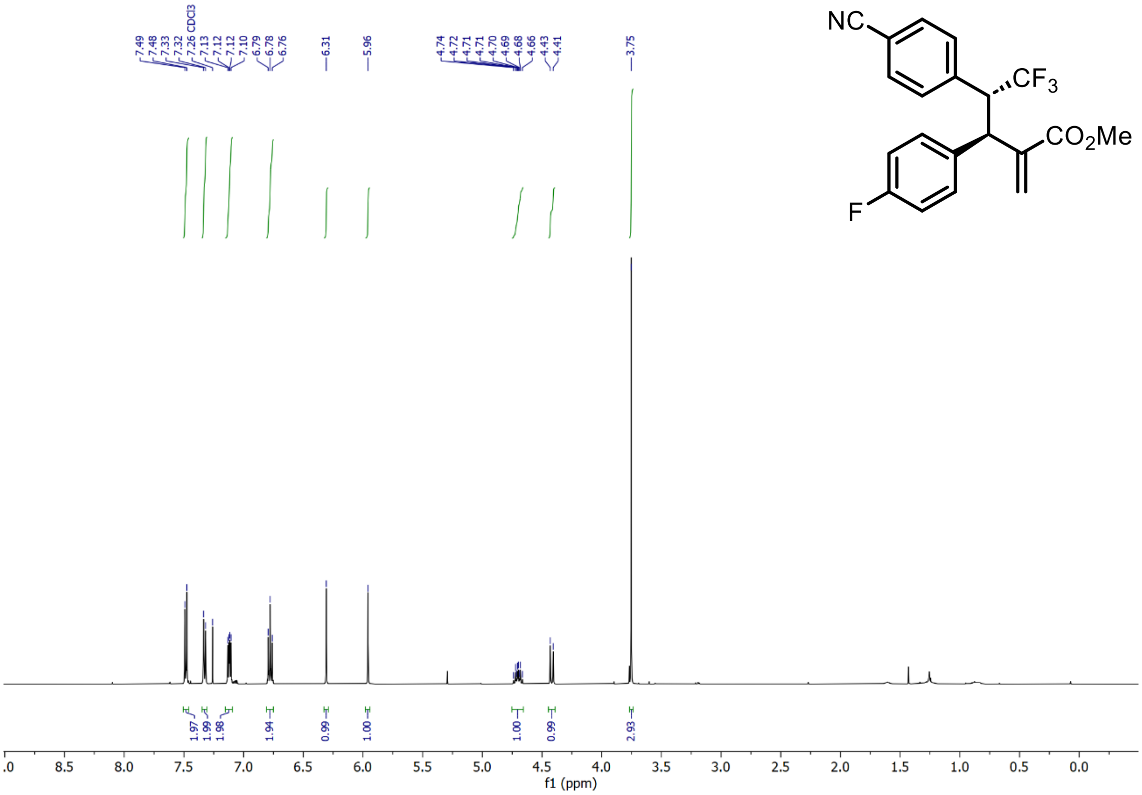

^{1}H NMR (500 MHz, $CDCl_3$) spectra of **3b**.

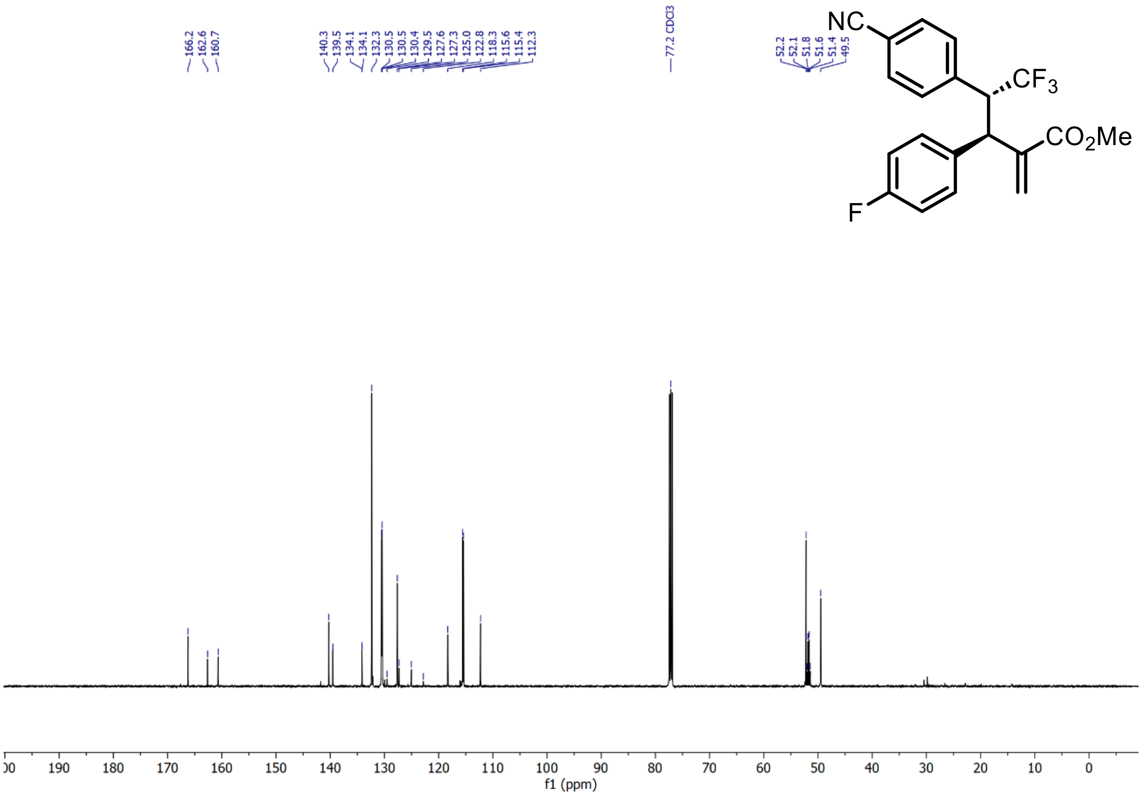

^{13}C NMR (126 MHz, $CDCl_3$) spectra of **3b**.

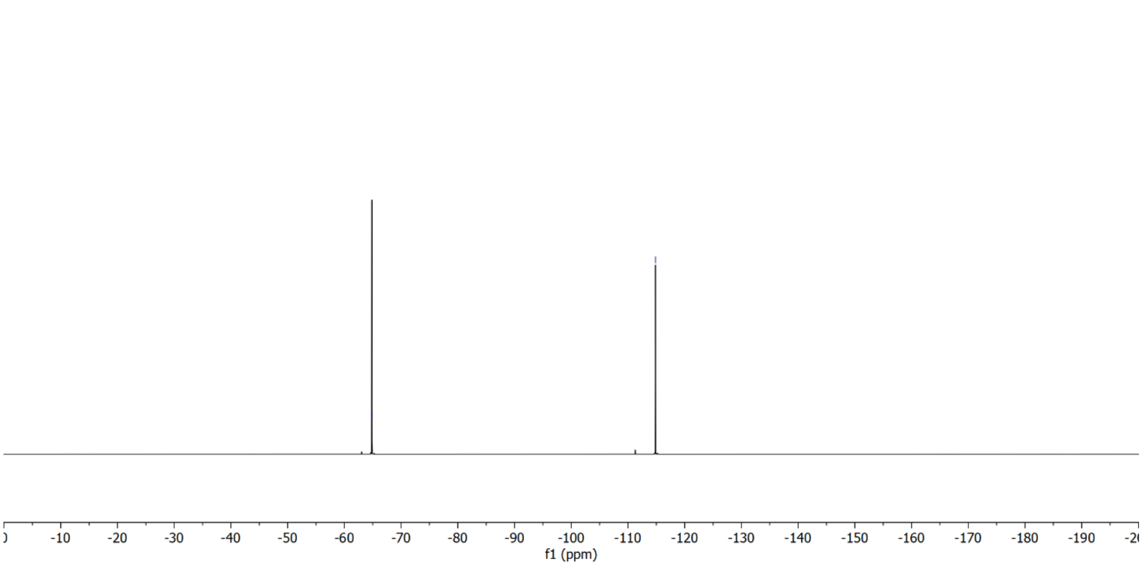

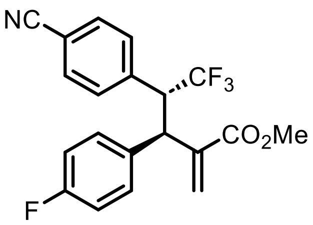

^{19}F NMR (471 MHz, $CDCl_3$) spectra of **3b**.

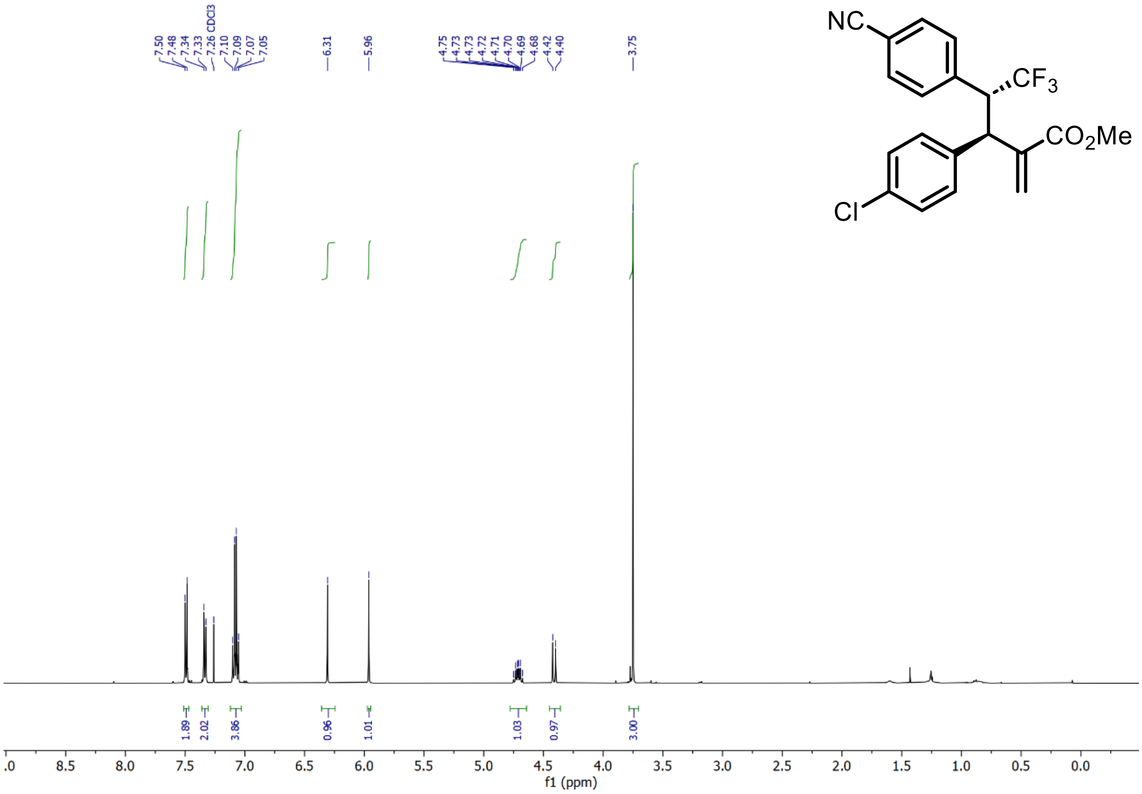

^{1}H NMR (500 MHz, $CDCl_3$) spectra of **3c**.

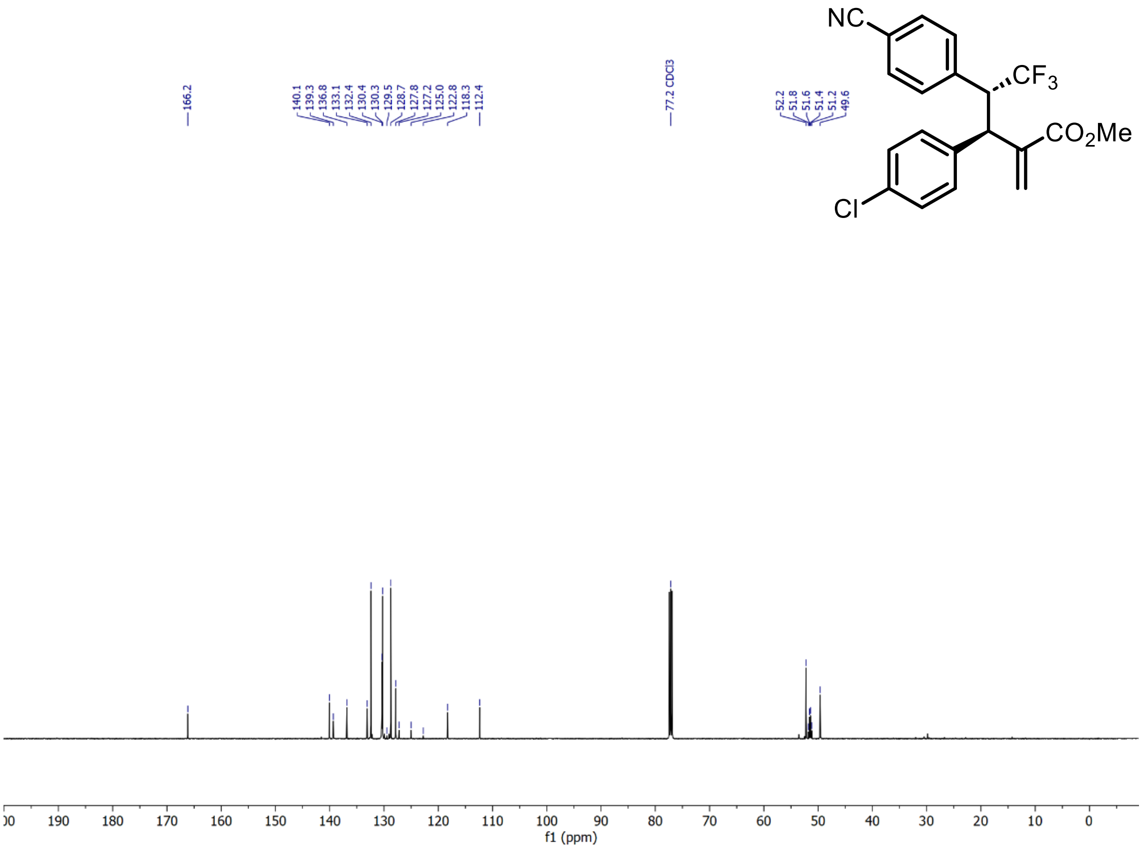

^{13}C NMR (126 MHz, $CDCl_3$) spectra of **3c**.

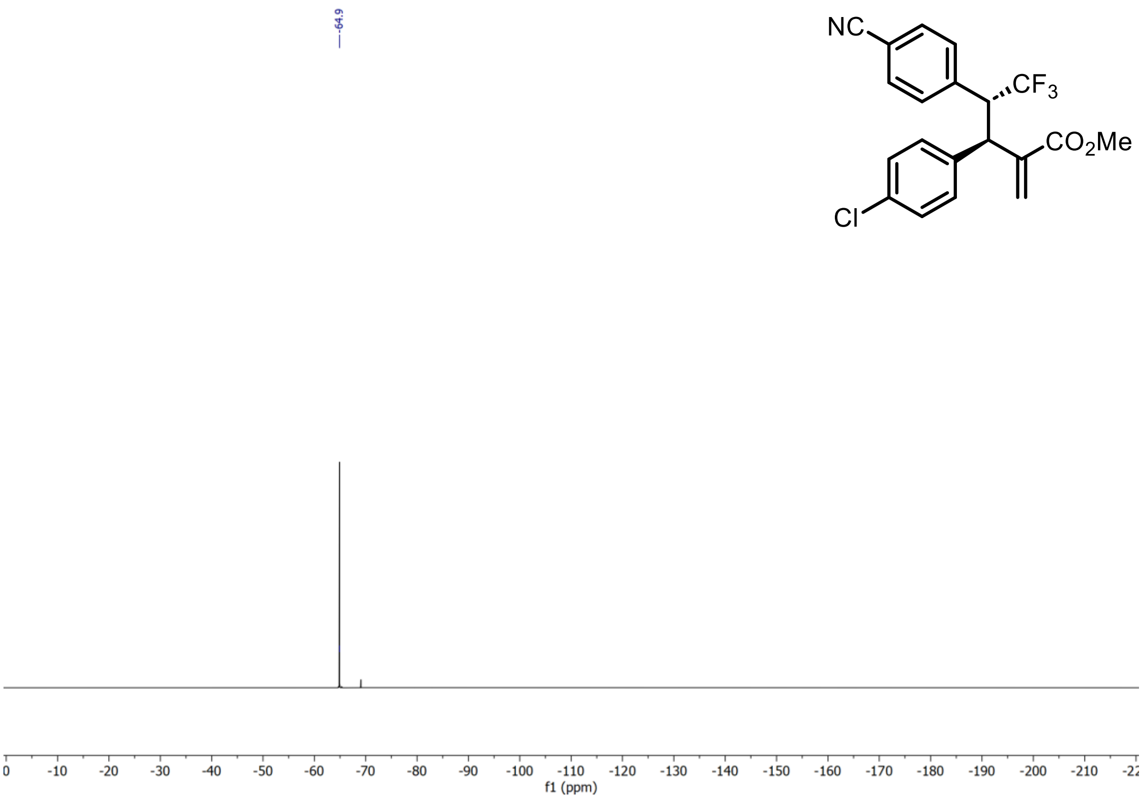

^{19}F NMR (471 MHz, $CDCl_3$) spectra of **3c.**

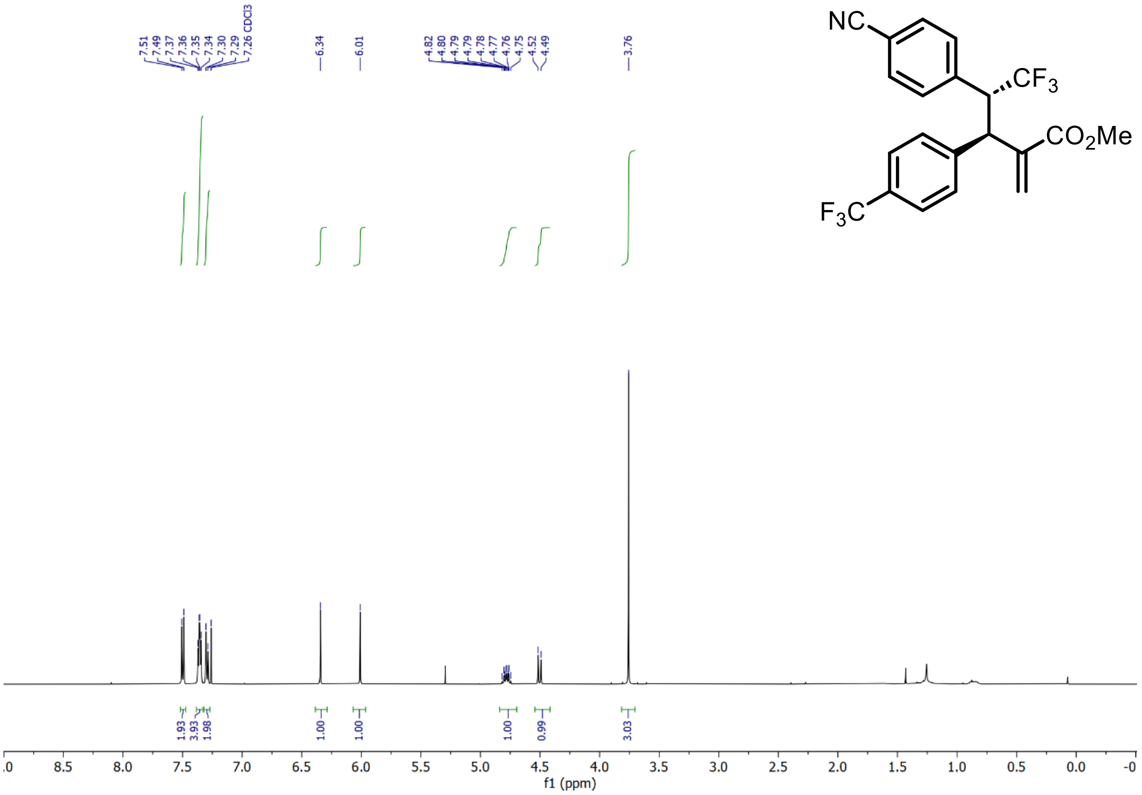

^{1}H NMR (500 MHz, $CDCl_3$) spectra of **3d**.

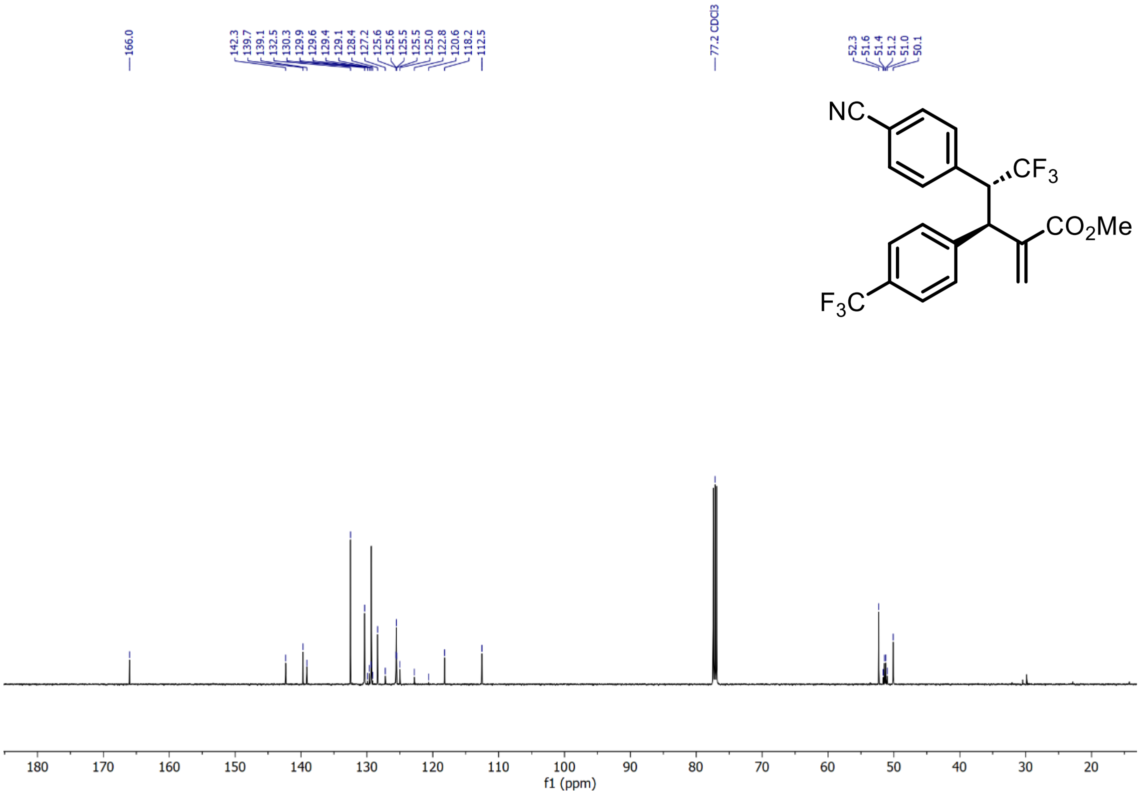

^{13}C NMR (126 MHz, $CDCl_3$) spectra of **3d**.

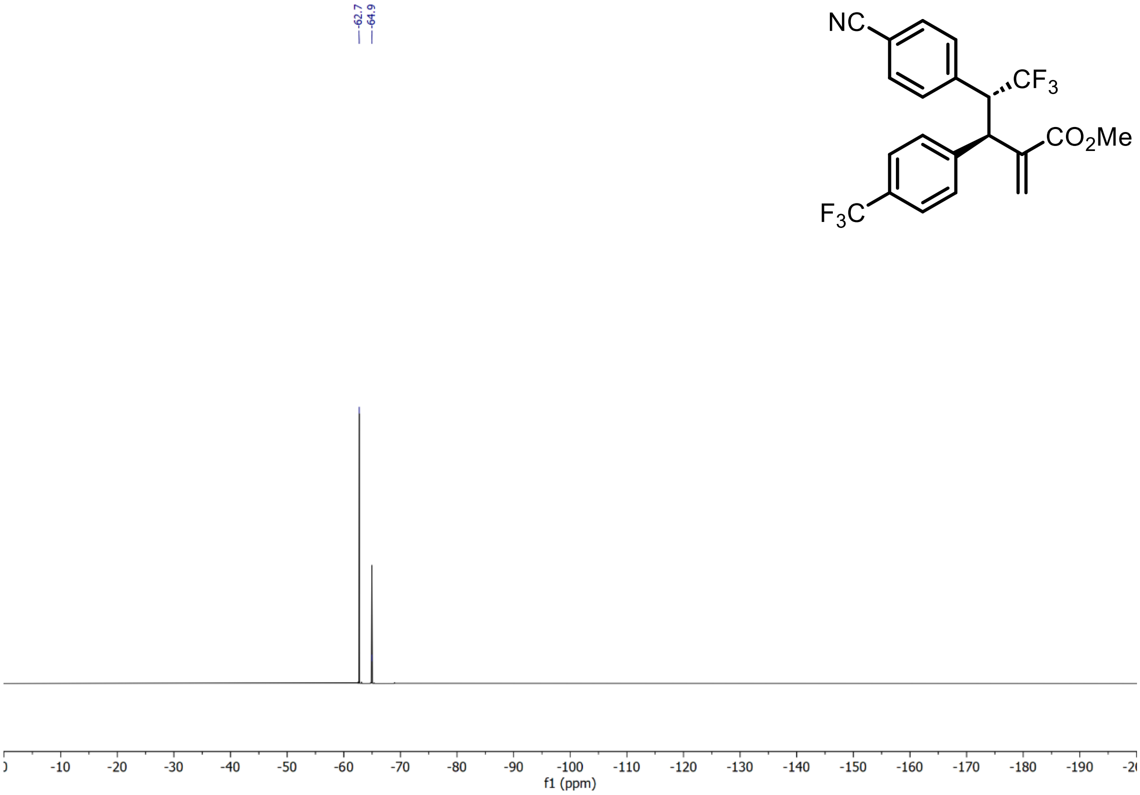

^{19}F NMR (471 MHz, $CDCl_3$) spectra of **3d.**

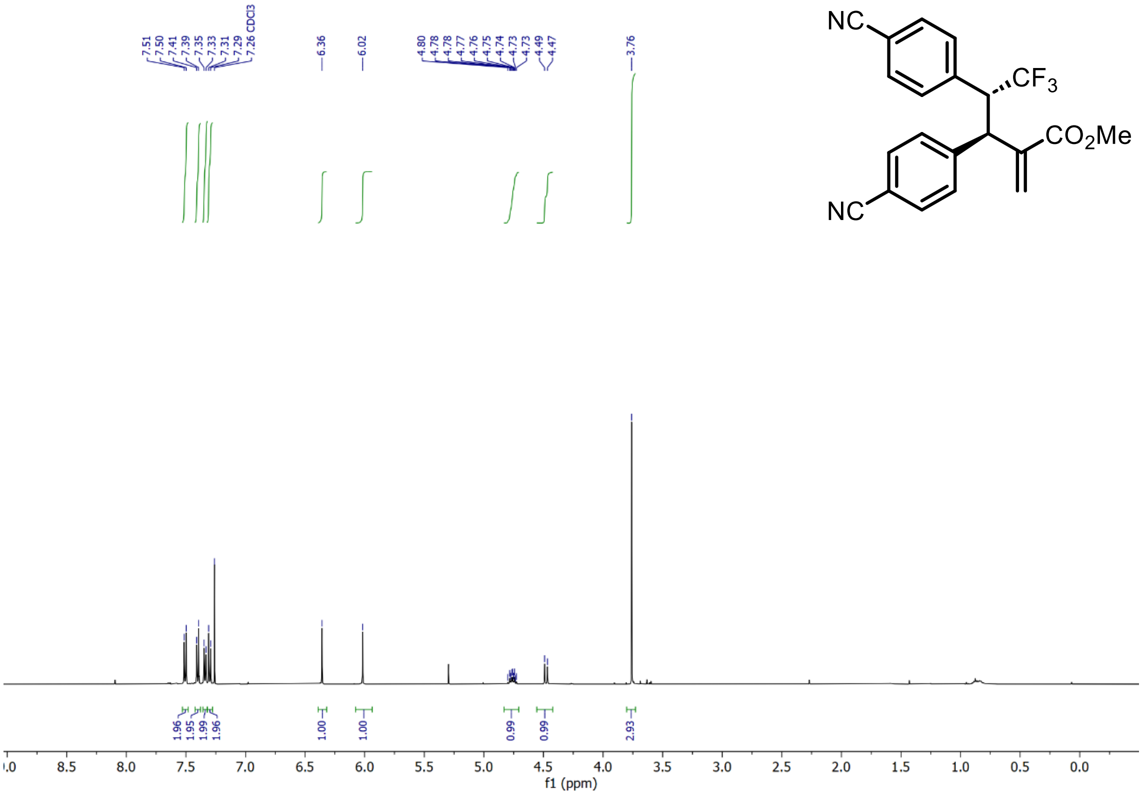

^{1}H NMR (500 MHz, $CDCl_3$) spectra of **3e**.

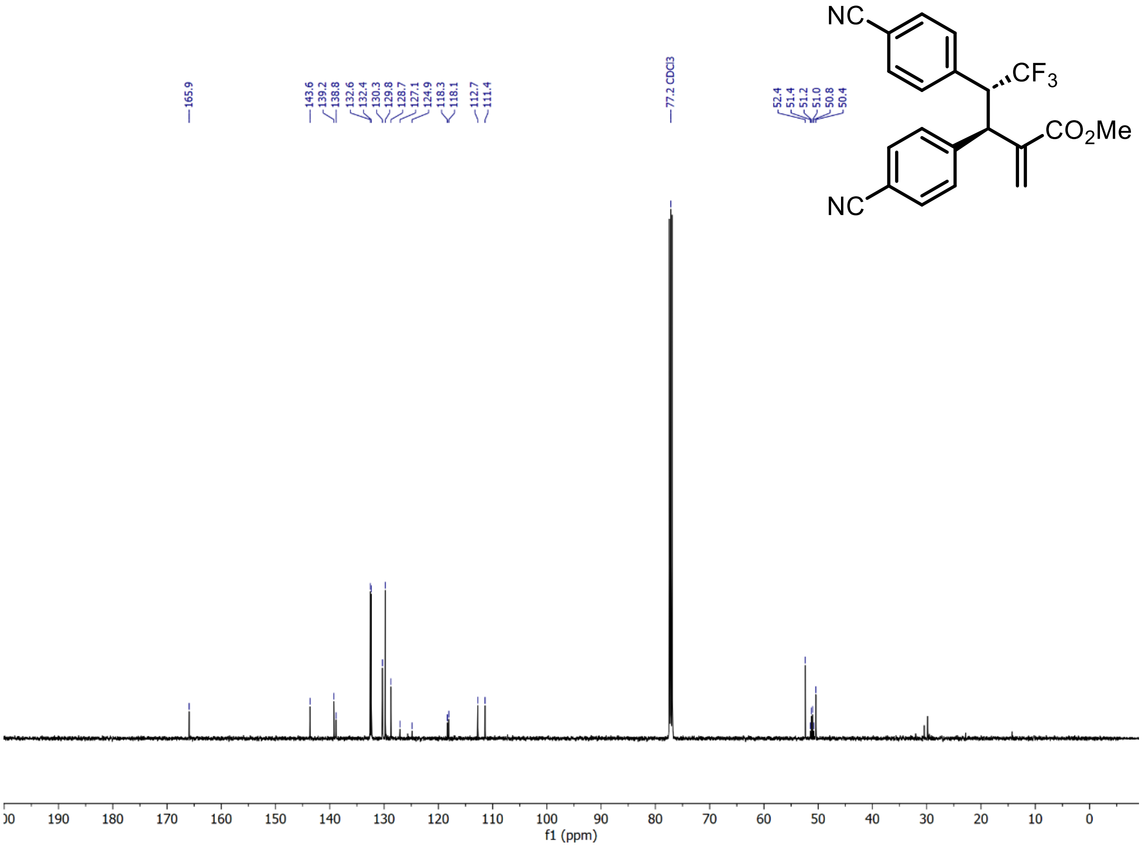

^{13}C NMR (126 MHz, $CDCl_3$) spectra of **3e**.

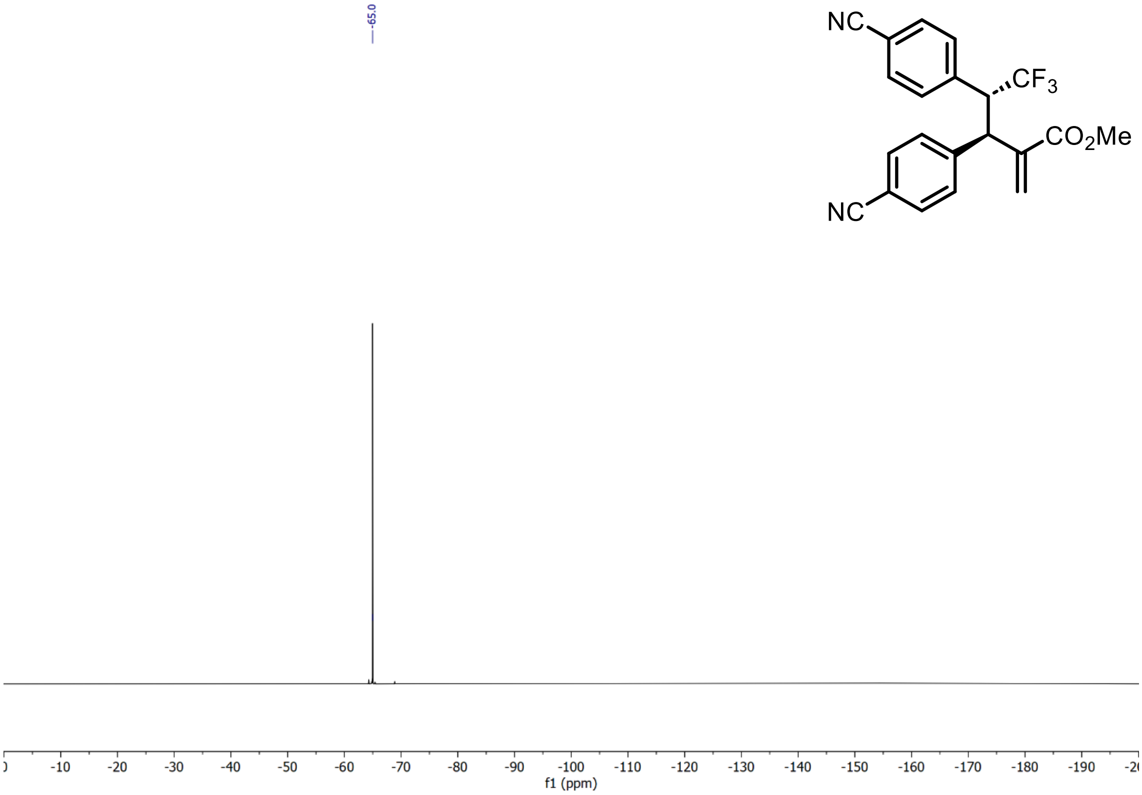

^{19}F NMR (471 MHz, $CDCl_3$) spectra of **3e.**

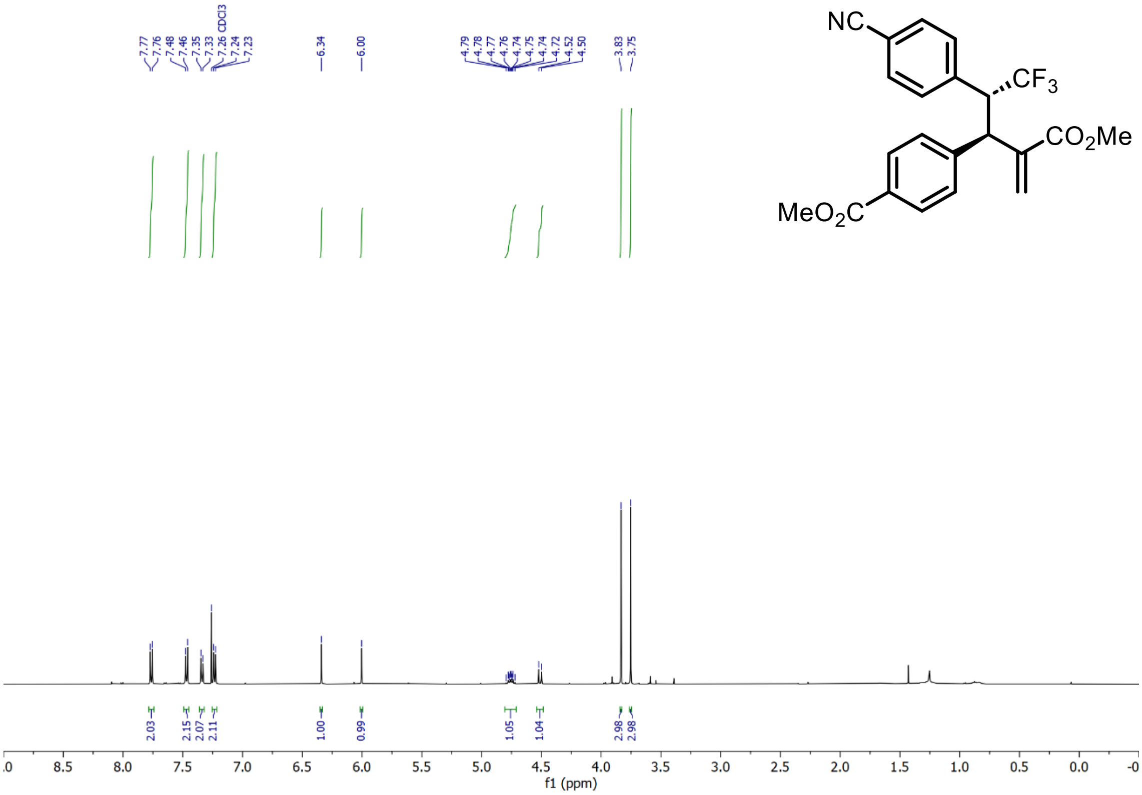

^{1}H NMR (500 MHz, $CDCl_3$) spectra of **3f**.

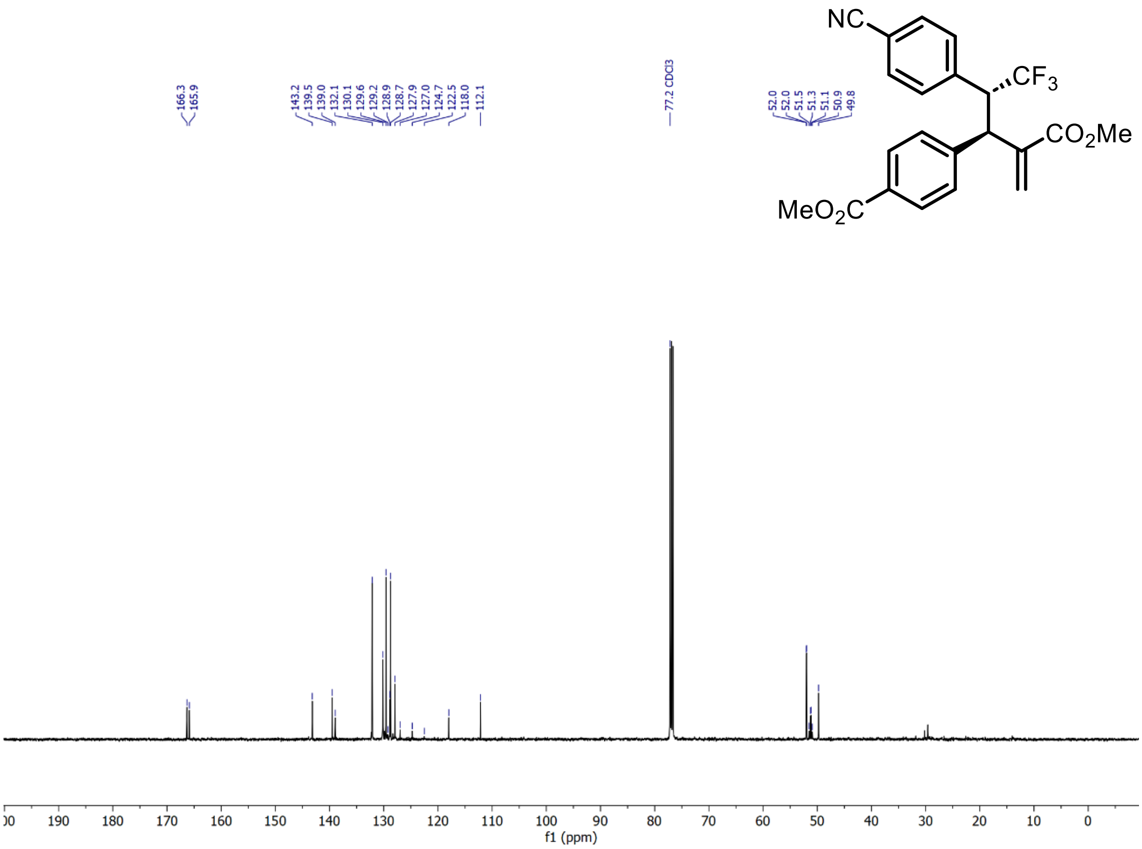

^{13}C NMR (126 MHz, $CDCl_3$) spectra of **3f**.

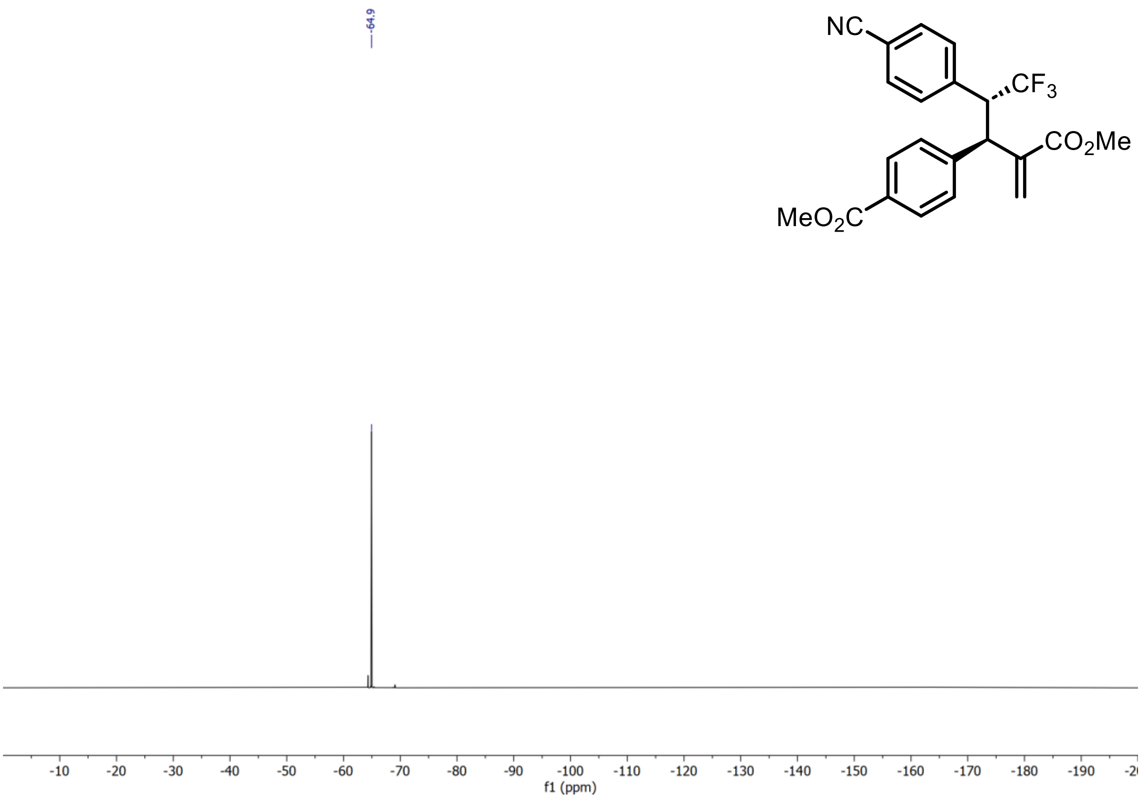

^{19}F NMR (471 MHz, $CDCl_3$) spectra of **3f**.

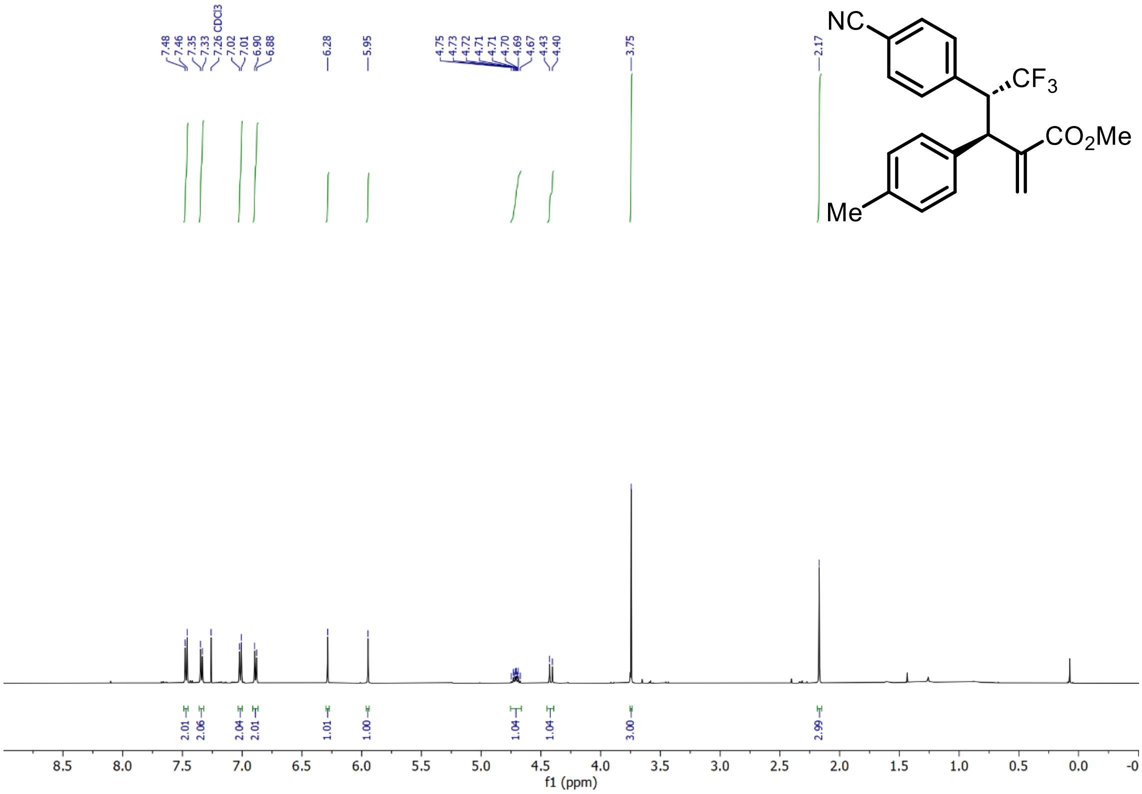

^{1}H NMR (500 MHz, $CDCl_3$) spectra of **3g**.

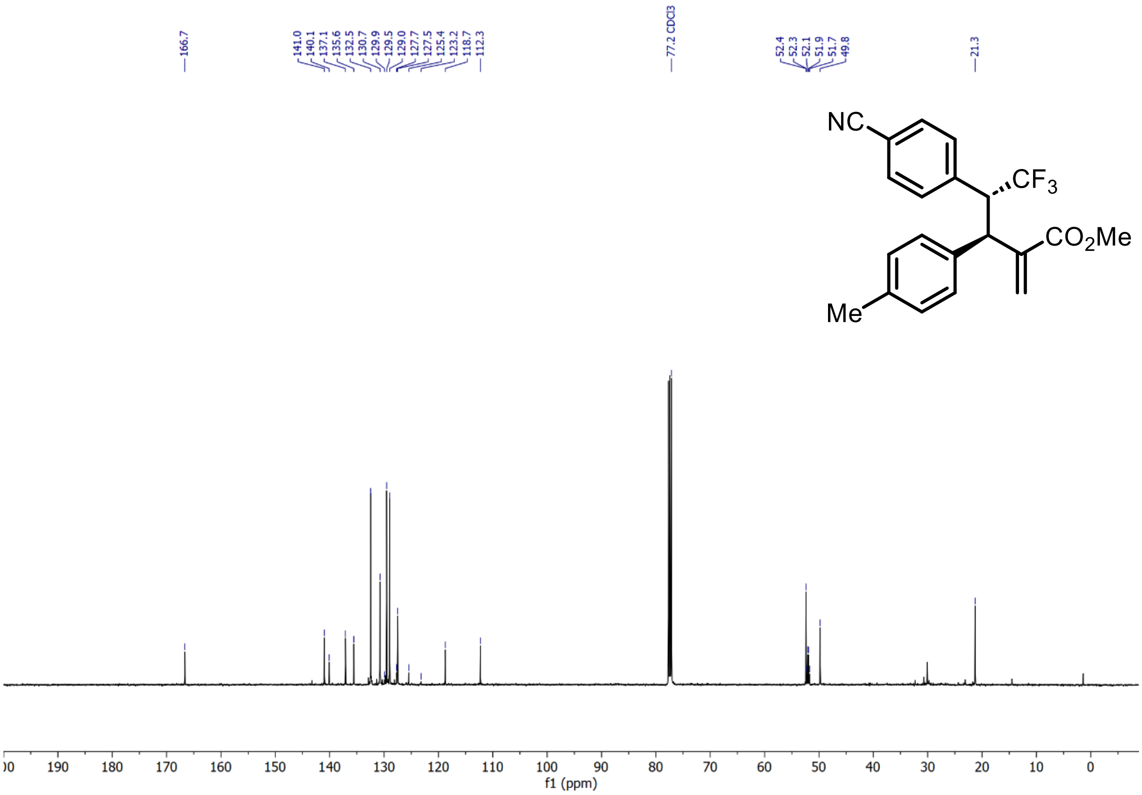

^{13}C NMR (126 MHz, $CDCl_3$) spectra of **3g**.

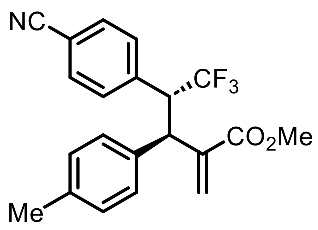

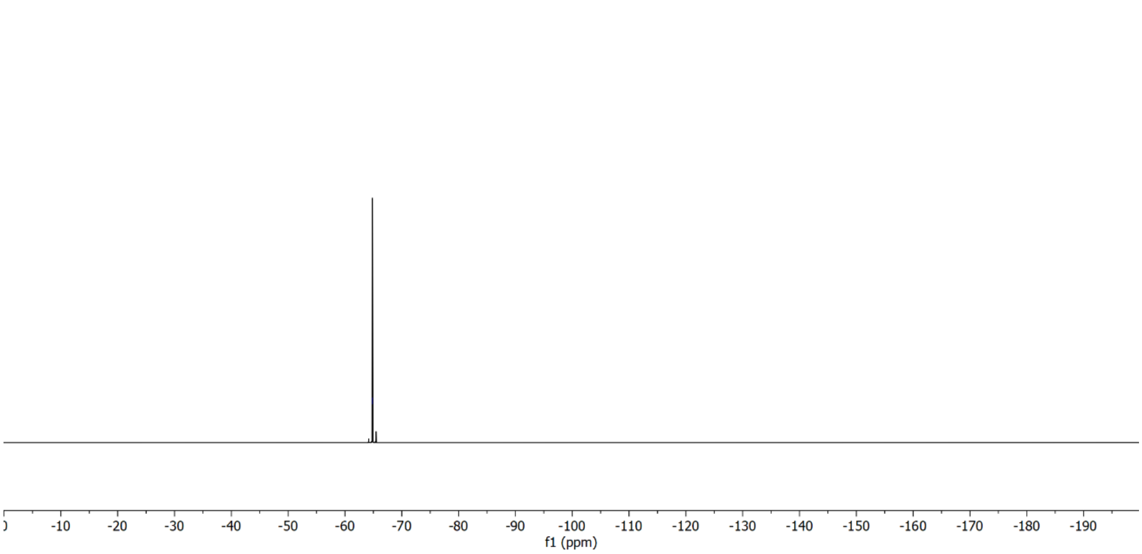

^{19}F NMR (471 MHz, $CDCl_3$) spectra of **3g**.

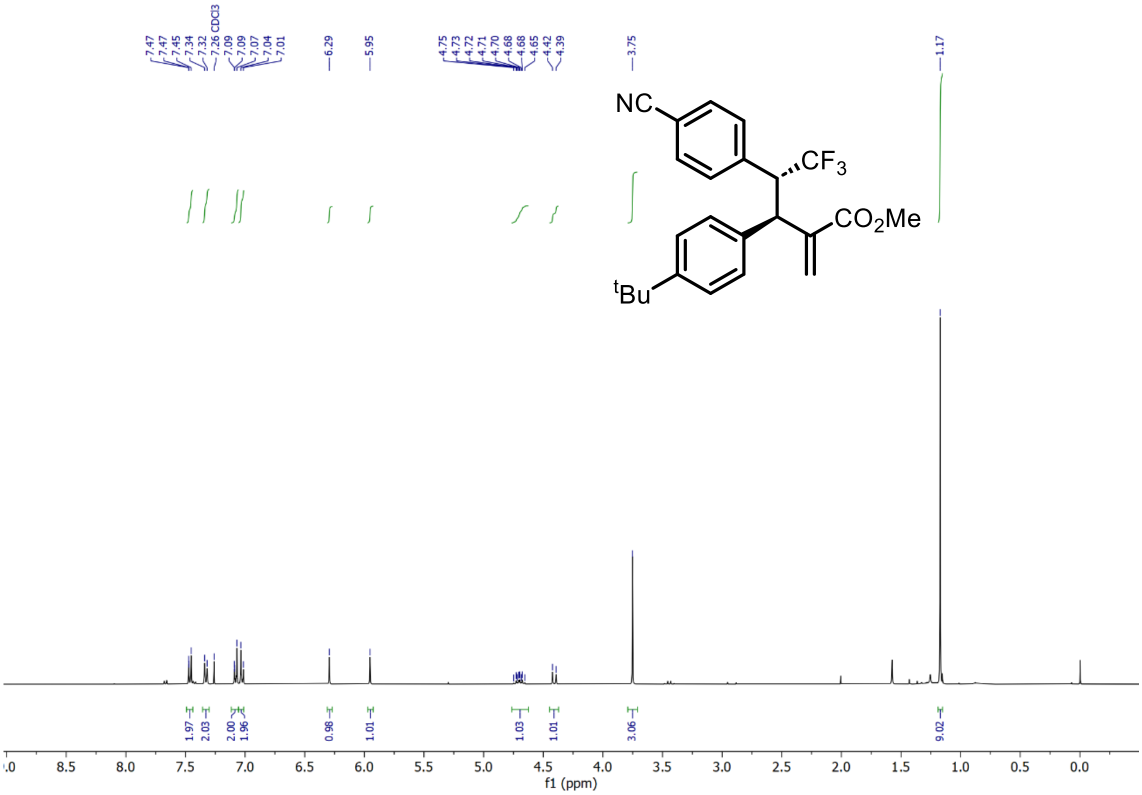

^{1}H NMR (400 MHz, $CDCl_3$) spectra of **3h**.

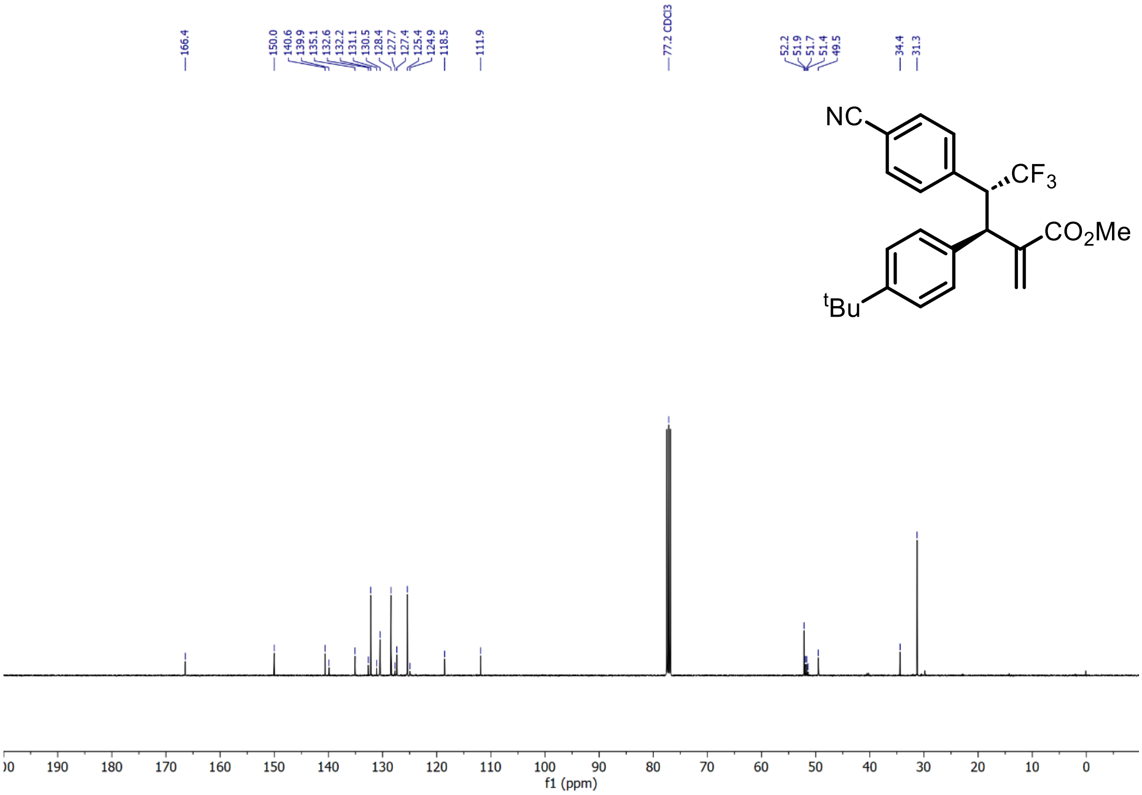

^{13}C NMR (101 MHz, $CDCl_3$) spectra of **3h**.

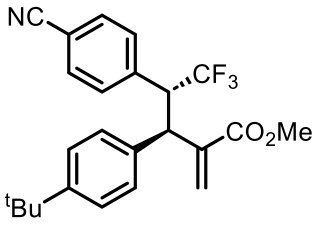

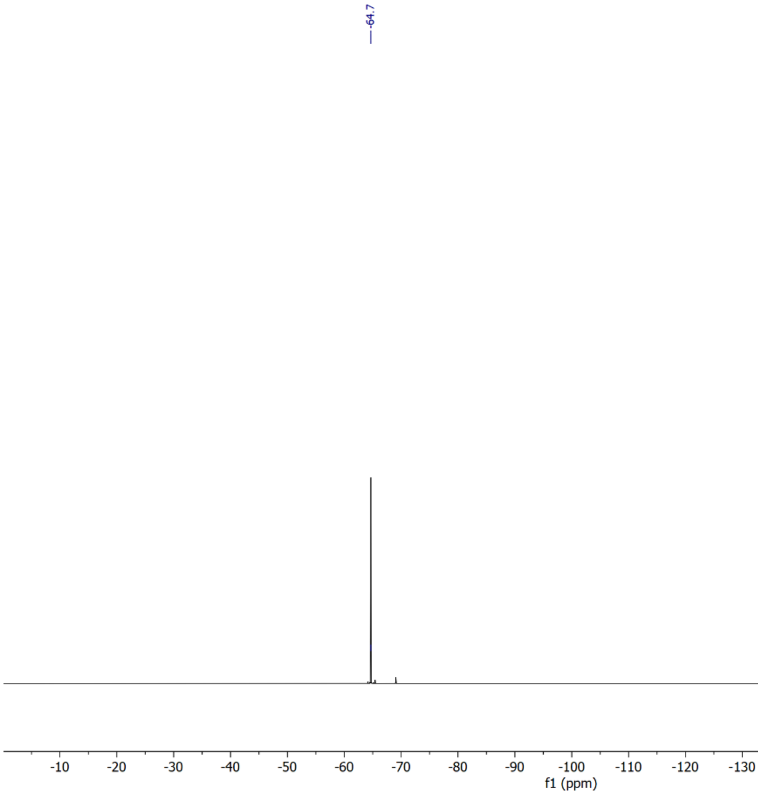

^{19}F NMR (376 MHz, $CDCl_3$) spectra of **3h**.

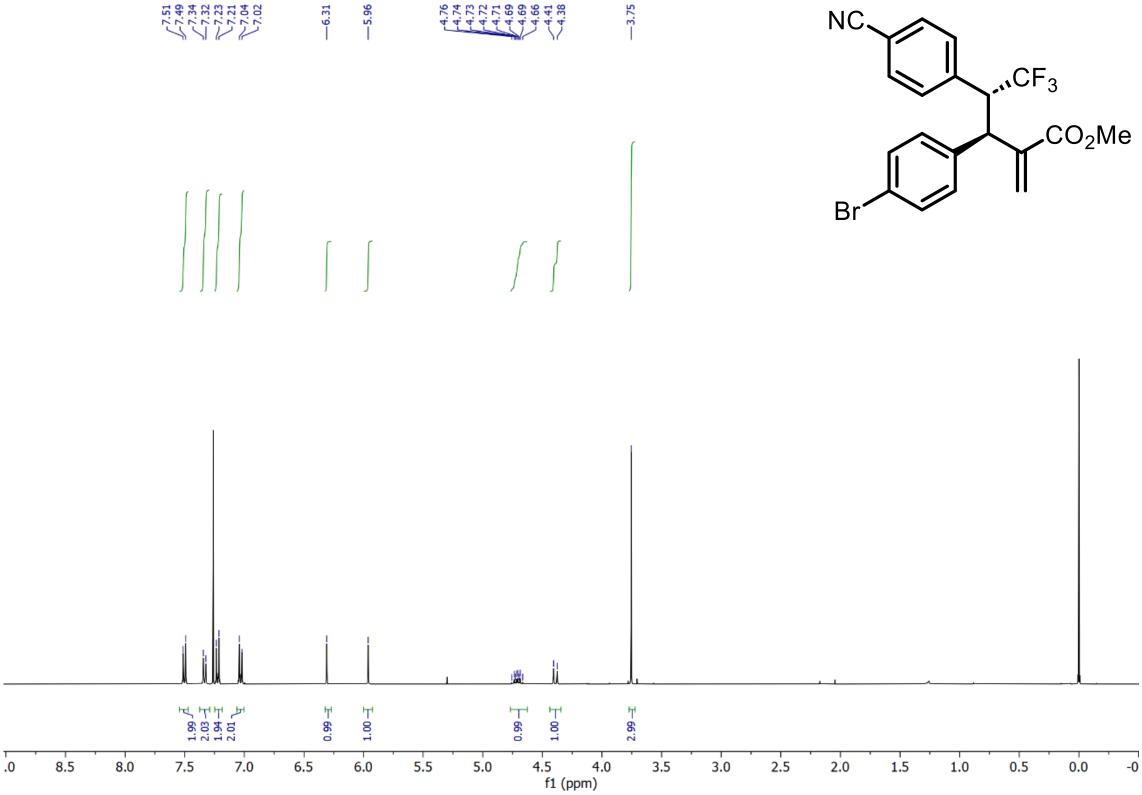

^{1}H NMR (500 MHz, $CDCl_3$) spectra of **3i**.

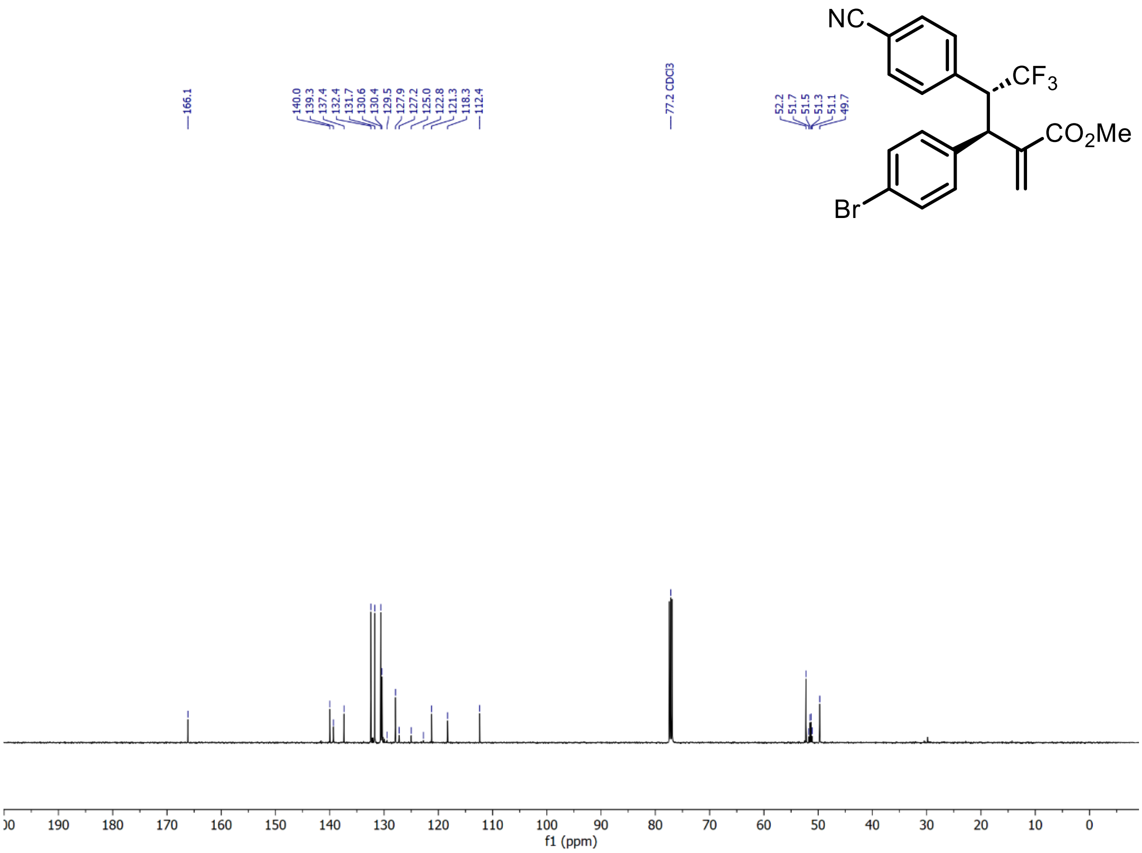

^{13}C NMR (126 MHz, $CDCl_3$) spectra of **3i**.

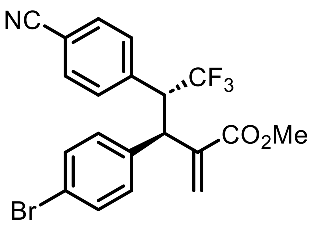

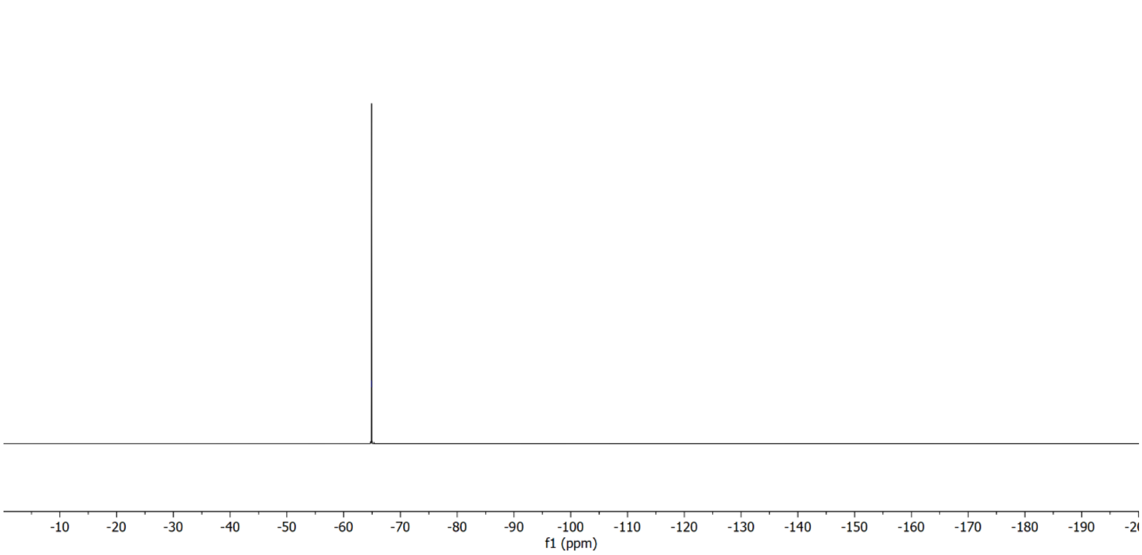

^{19}F NMR (471 MHz, $CDCl_3$) spectra of **3i.**

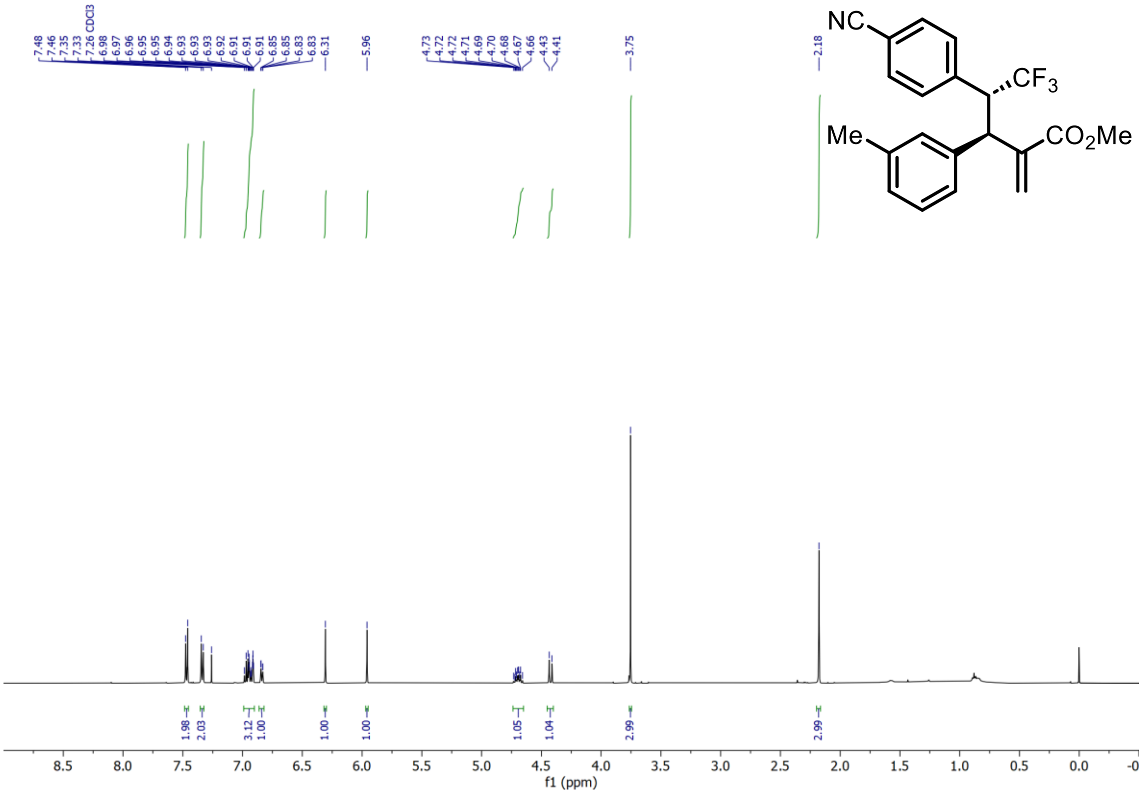

^{1}H NMR (500 MHz, $CDCl_3$) spectra of **3j**.

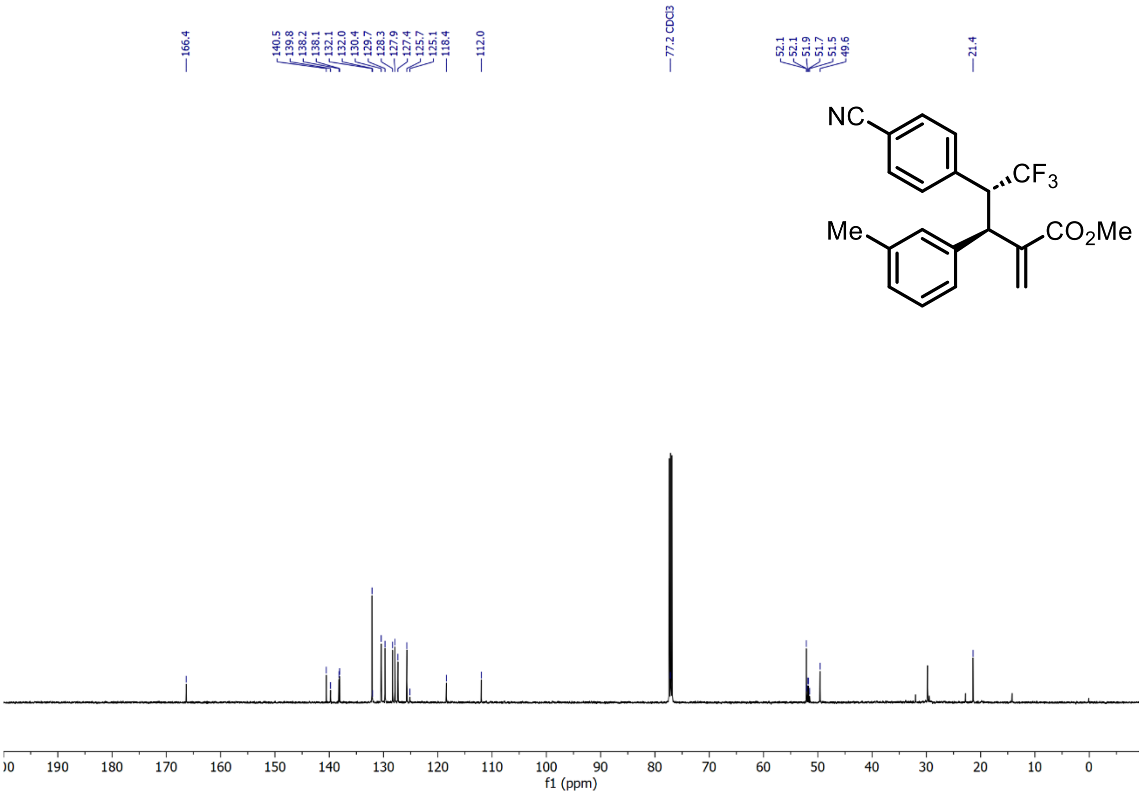

^{13}C NMR (126 MHz, $CDCl_3$) spectra of **3j**.

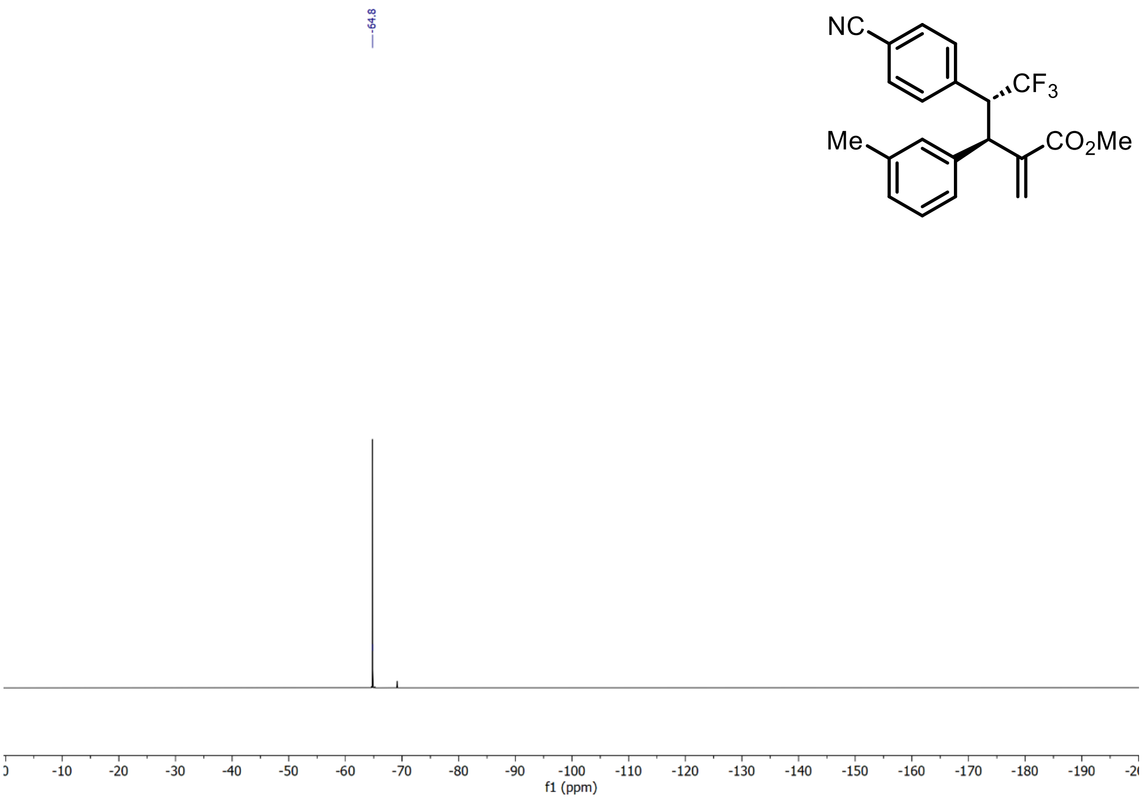

^{19}F NMR (471 MHz, $CDCl_3$) spectra of **3j**.

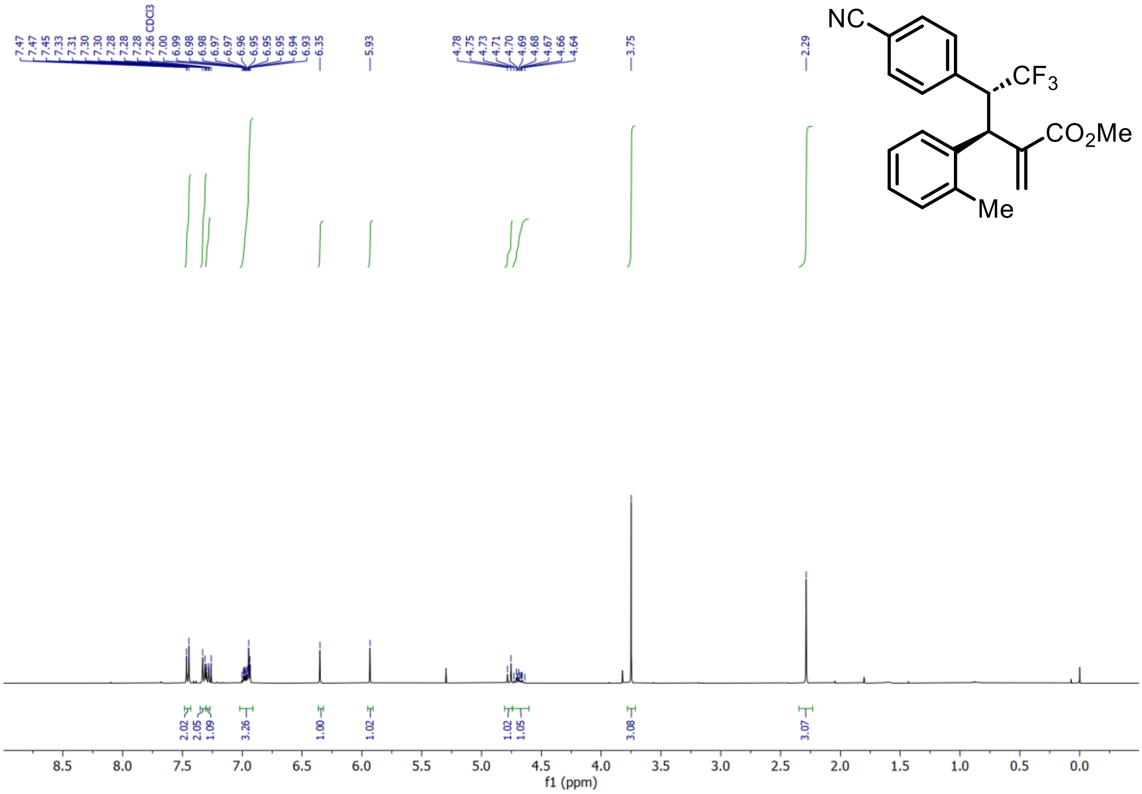

^{1}H NMR (400 MHz, $CDCl_3$) spectra of **3k**.

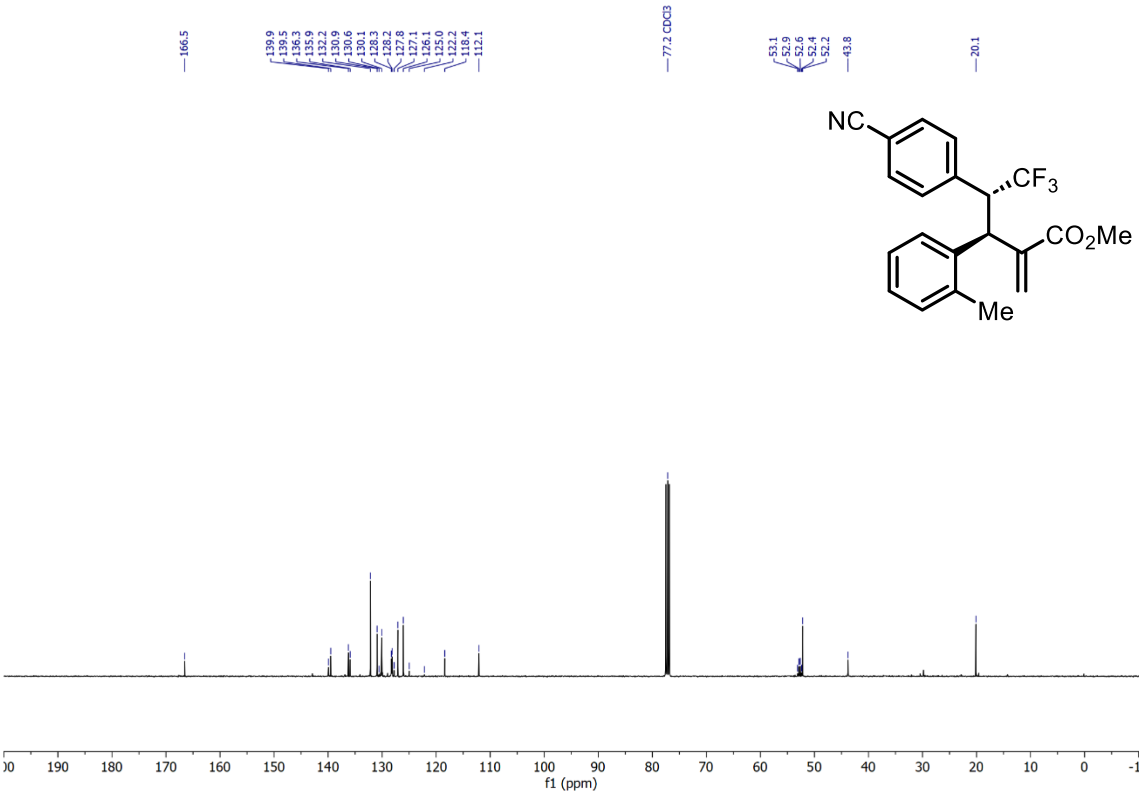

^{13}C NMR (101 MHz, $CDCl_3$) spectra of **3k**.

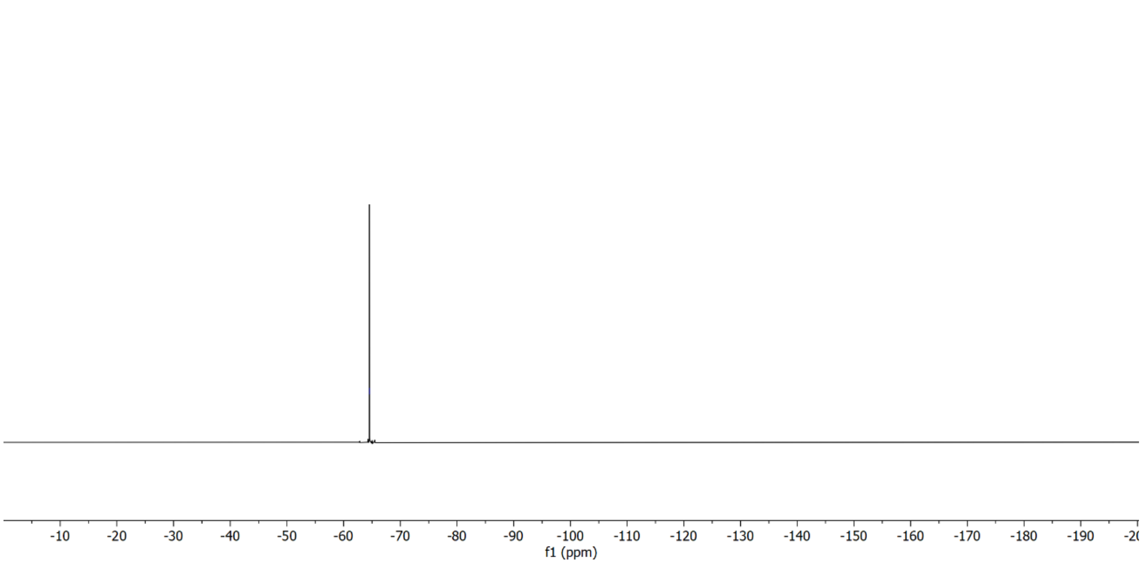

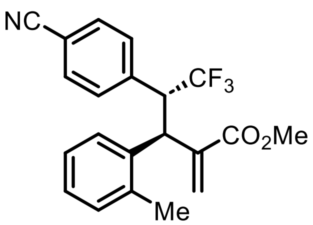

^{19}F NMR (376 MHz, $CDCl_3$) spectra of **3k**.

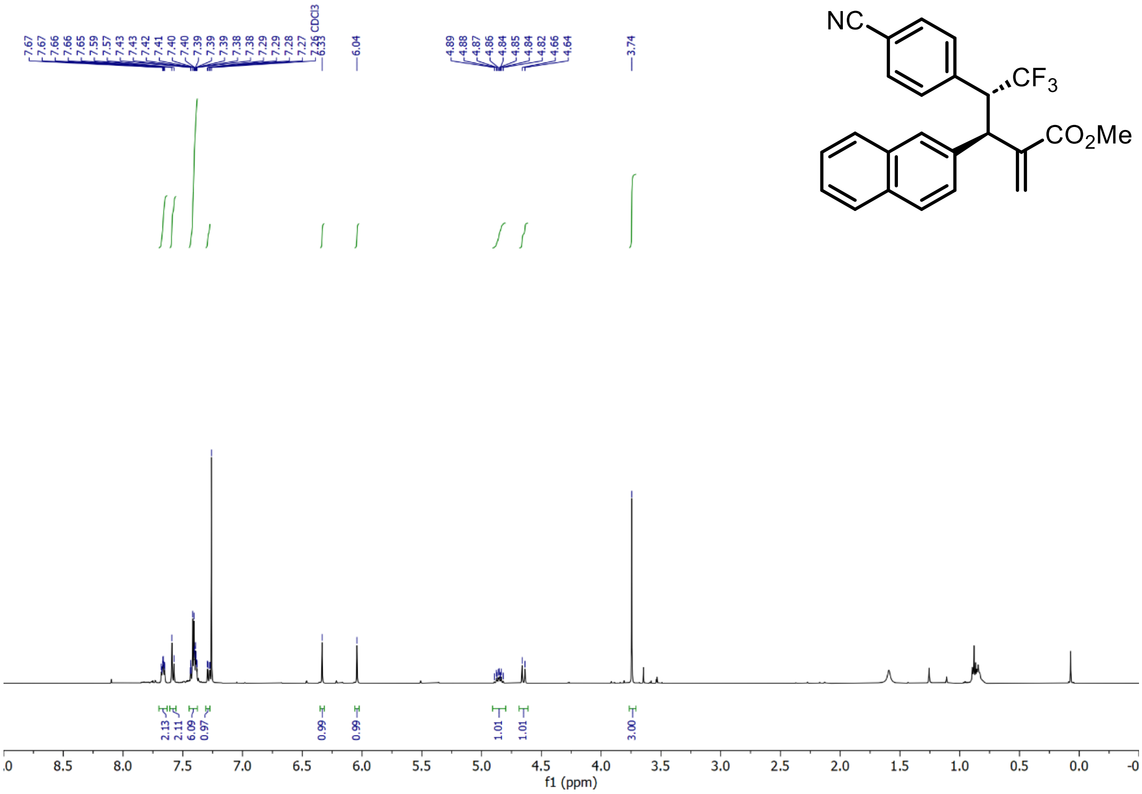

^{1}H NMR (500 MHz, $CDCl_3$) spectra of **3l**.

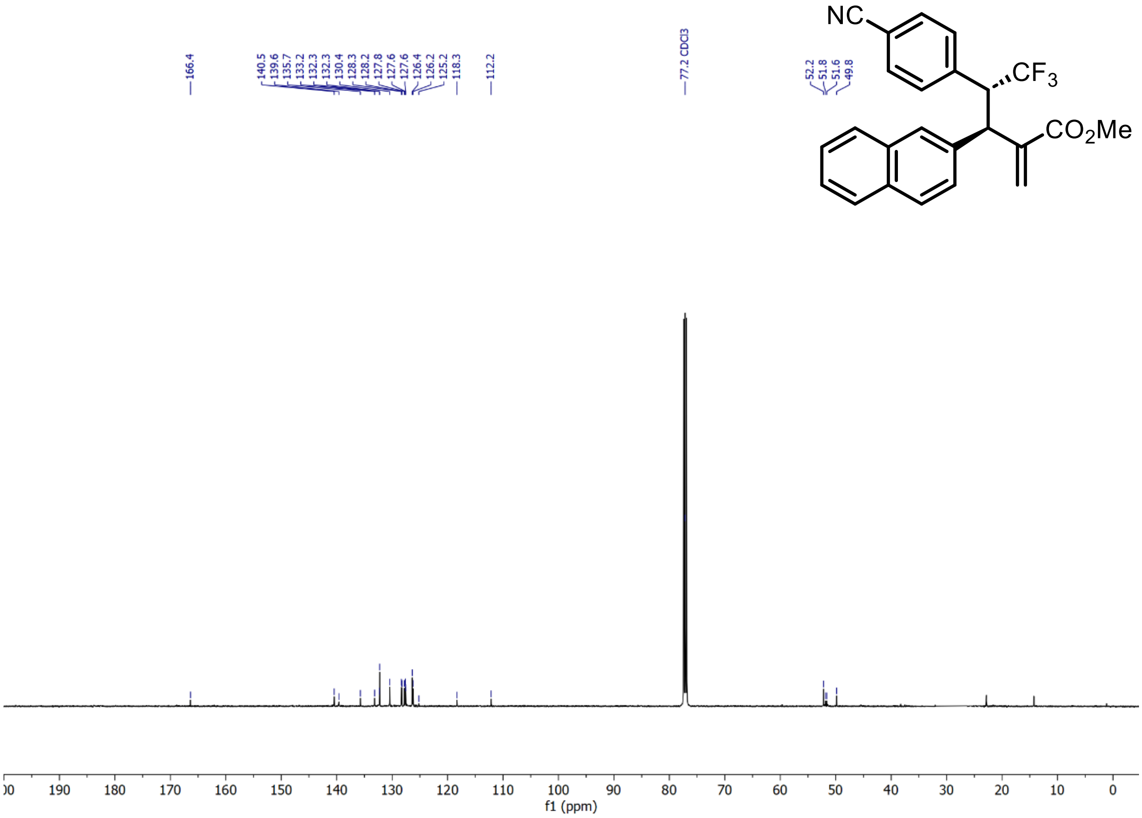

^{13}C NMR (126 MHz, $CDCl_3$) spectra of **3l**.

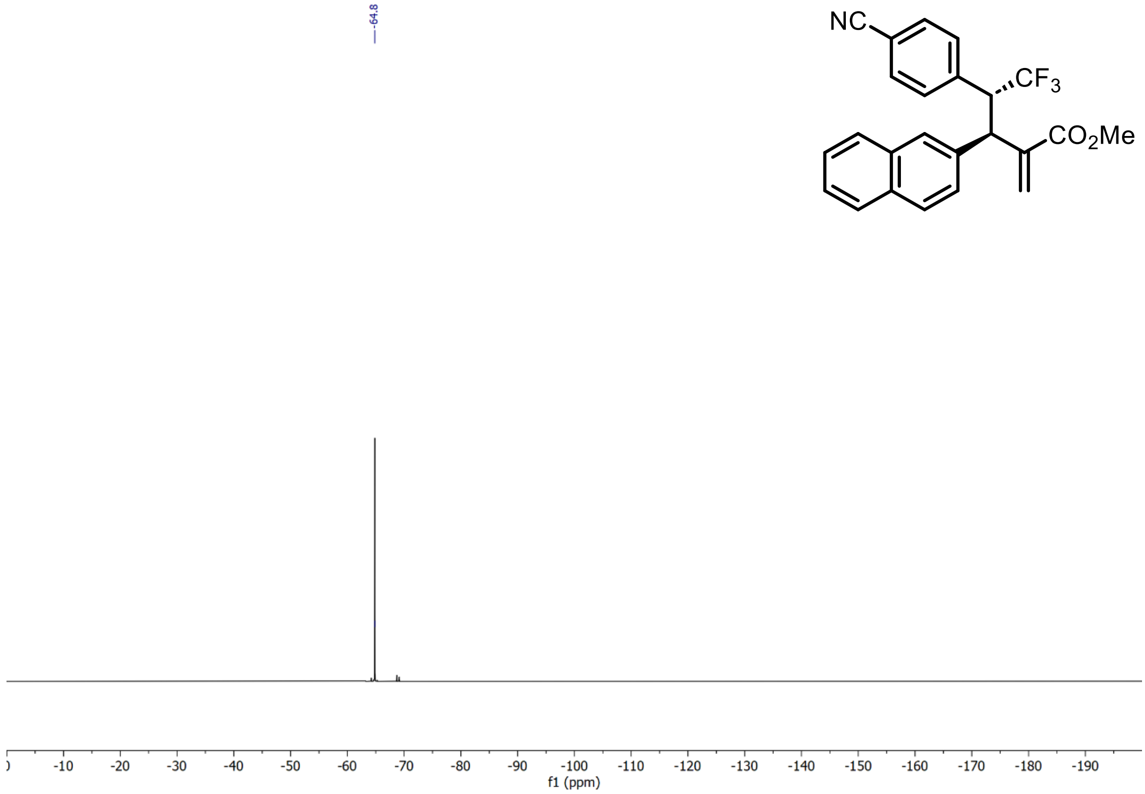

^{19}F NMR (471 MHz, $CDCl_3$) spectra of **3l**.

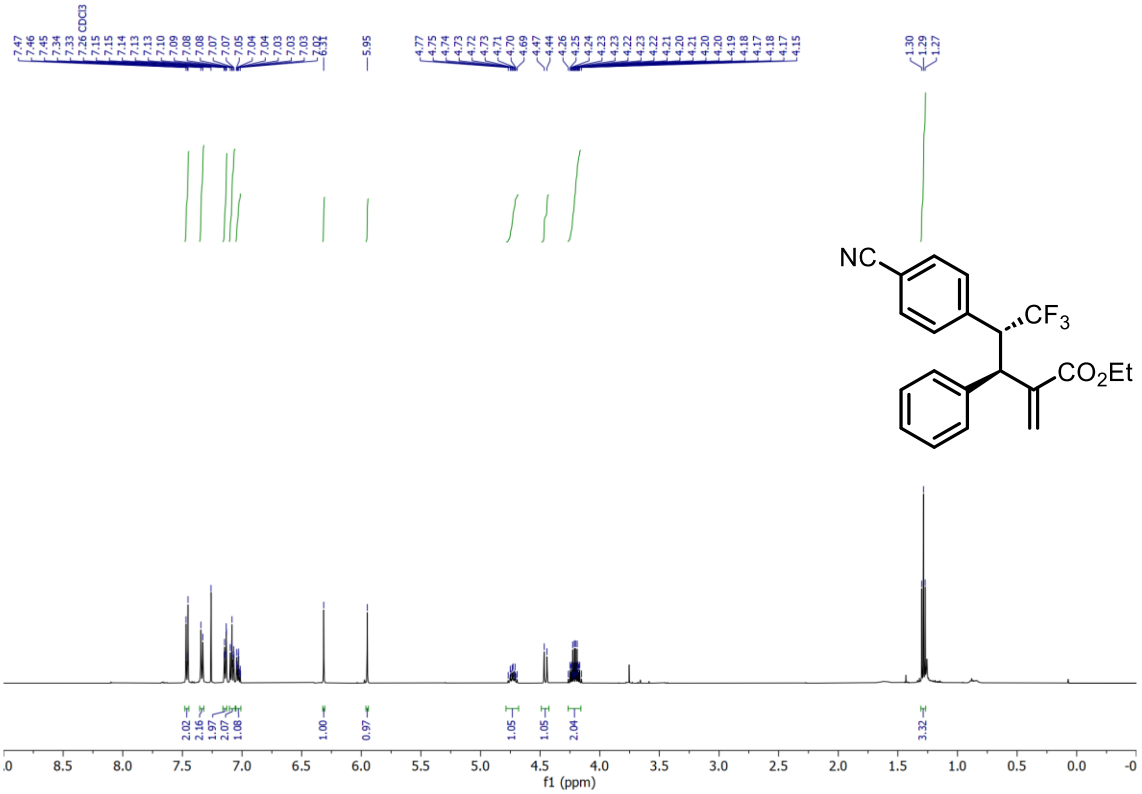

^{1}H NMR (500 MHz, $CDCl_3$) spectra of **3m**.

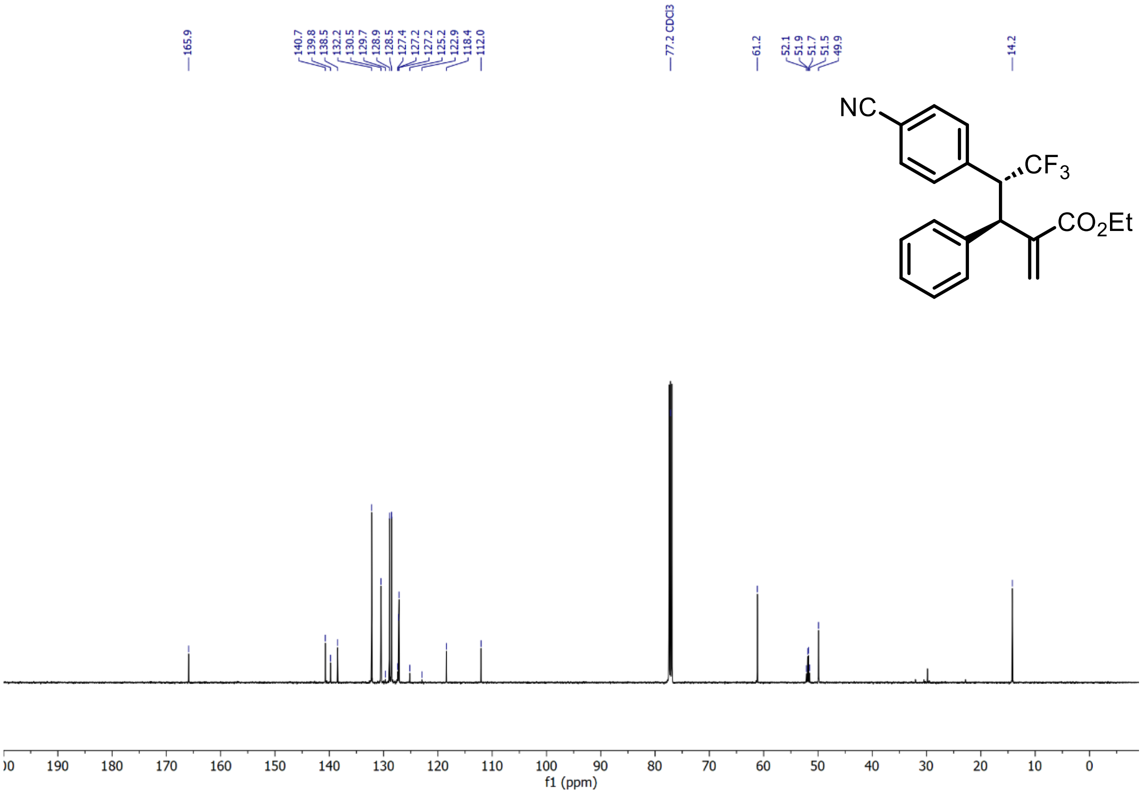

^{13}C NMR (126 MHz, $CDCl_3$) spectra of **3m**.

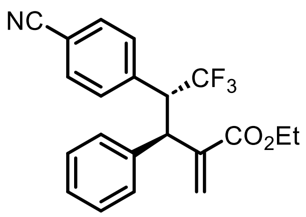

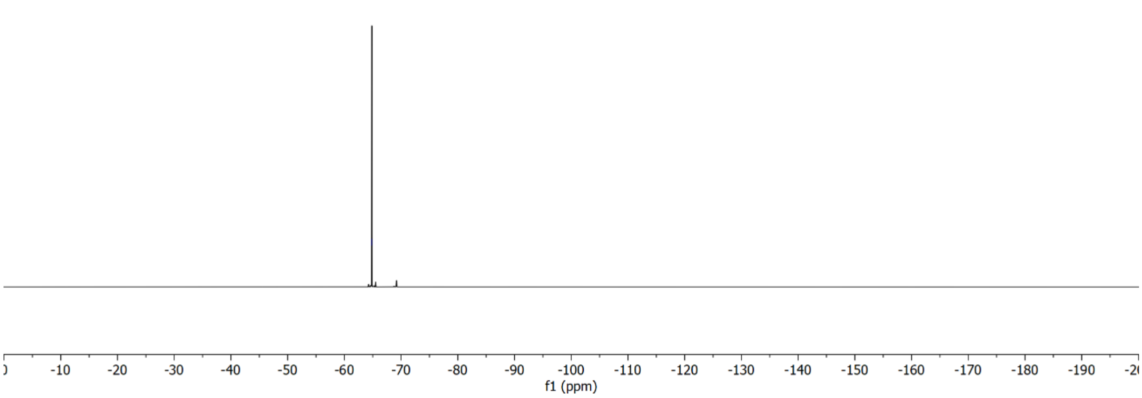

^{19}F NMR (471 MHz, $CDCl_3$) spectra of **3m**.

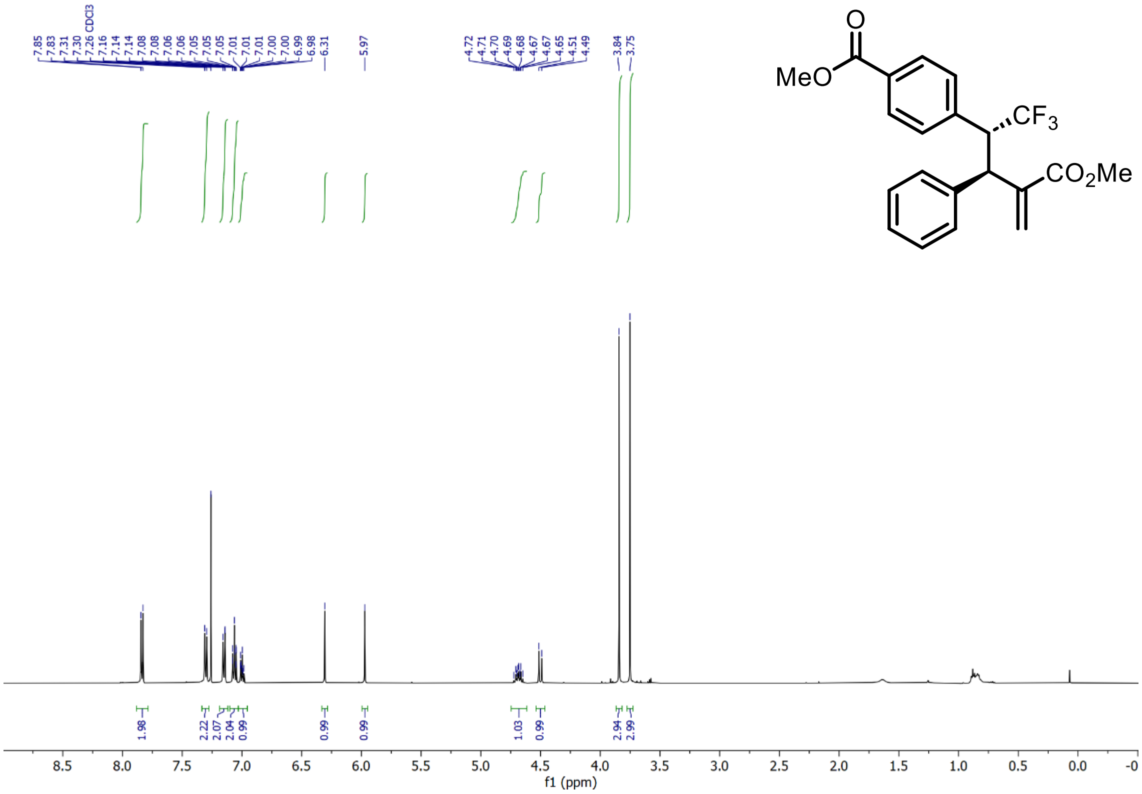

^{1}H NMR (400 MHz, $CDCl_3$) spectra of **3n**.

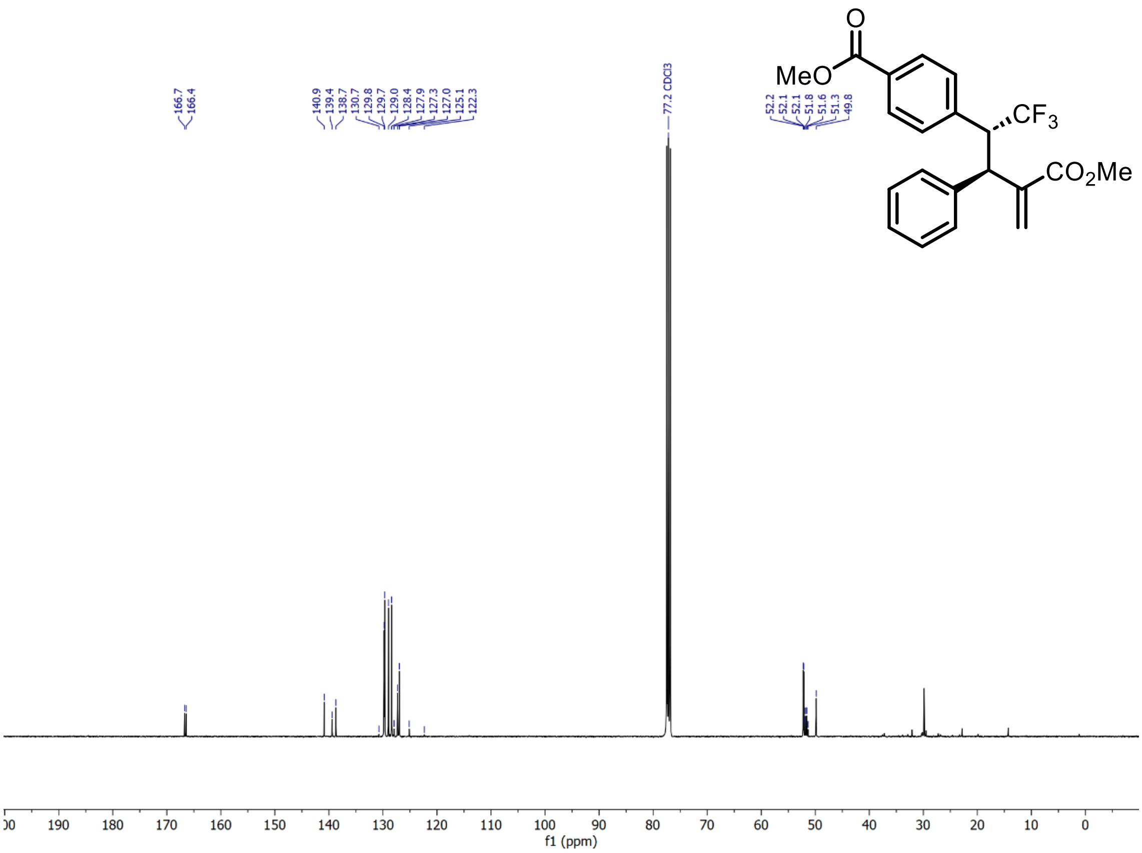

^{13}C NMR (101 MHz, $CDCl_3$) spectra of **3n**.

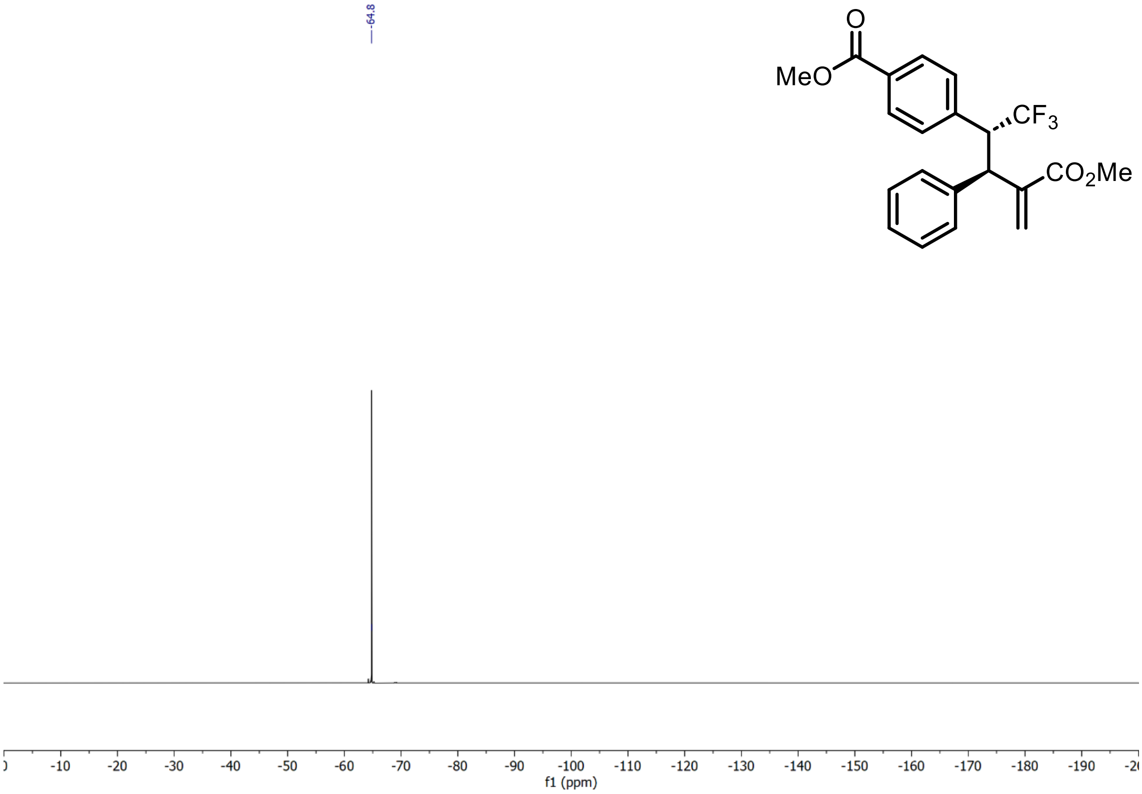

^{19}F NMR (471 MHz, $CDCl_3$) spectra of **3n**.

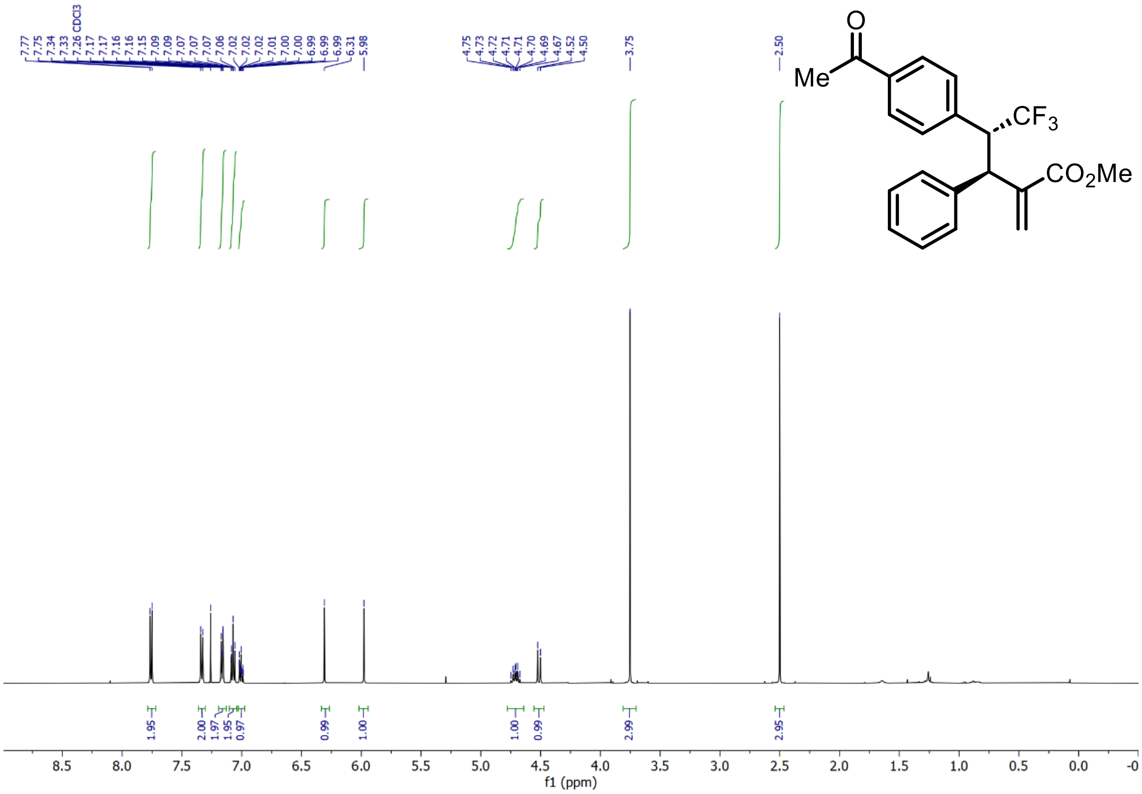

^1H NMR (400 MHz, $CDCl_3$) spectra of **3o**.

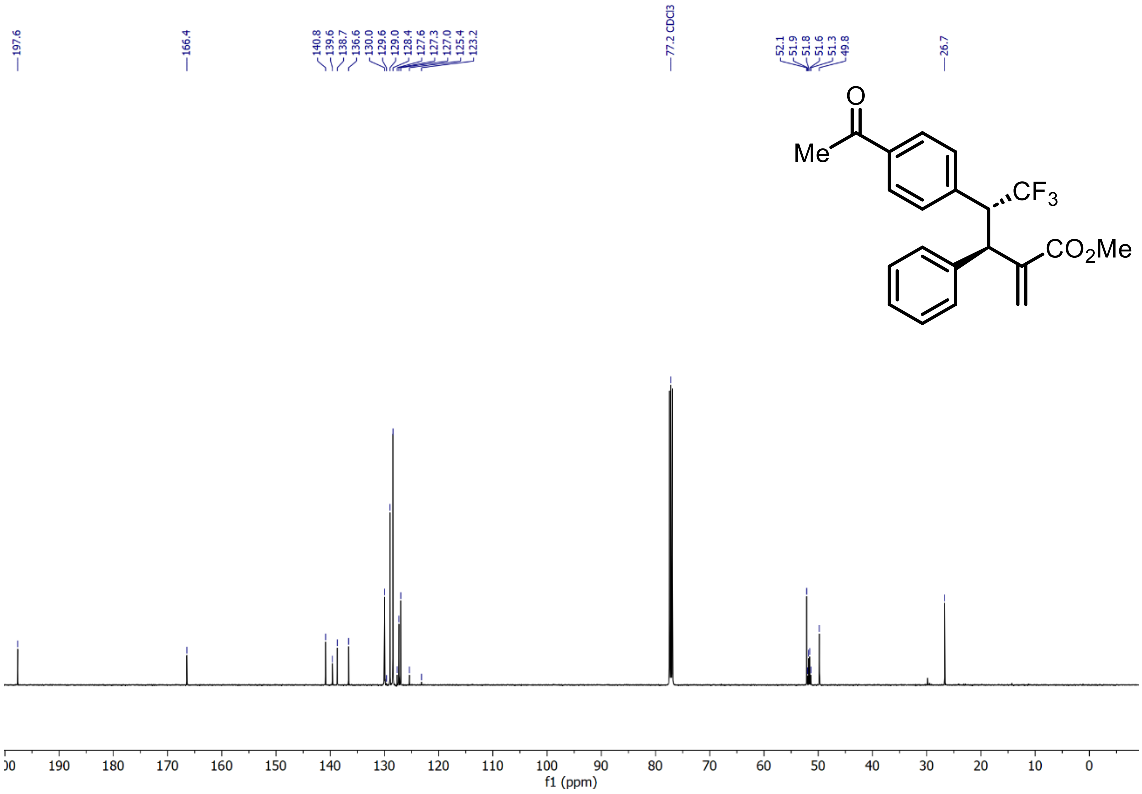

^{13}C NMR (101 MHz, $CDCl_3$) spectra of **3o**.

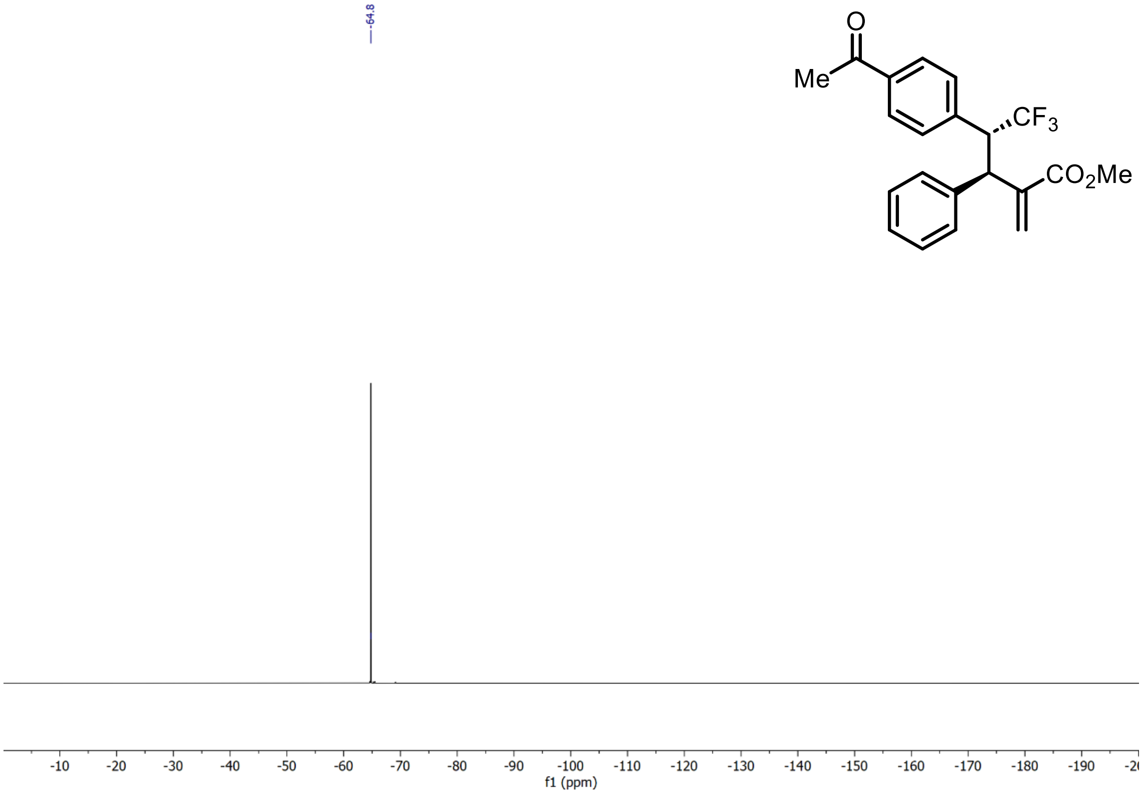

^{19}F NMR (471 MHz, $CDCl_3$) spectra of **3o**.

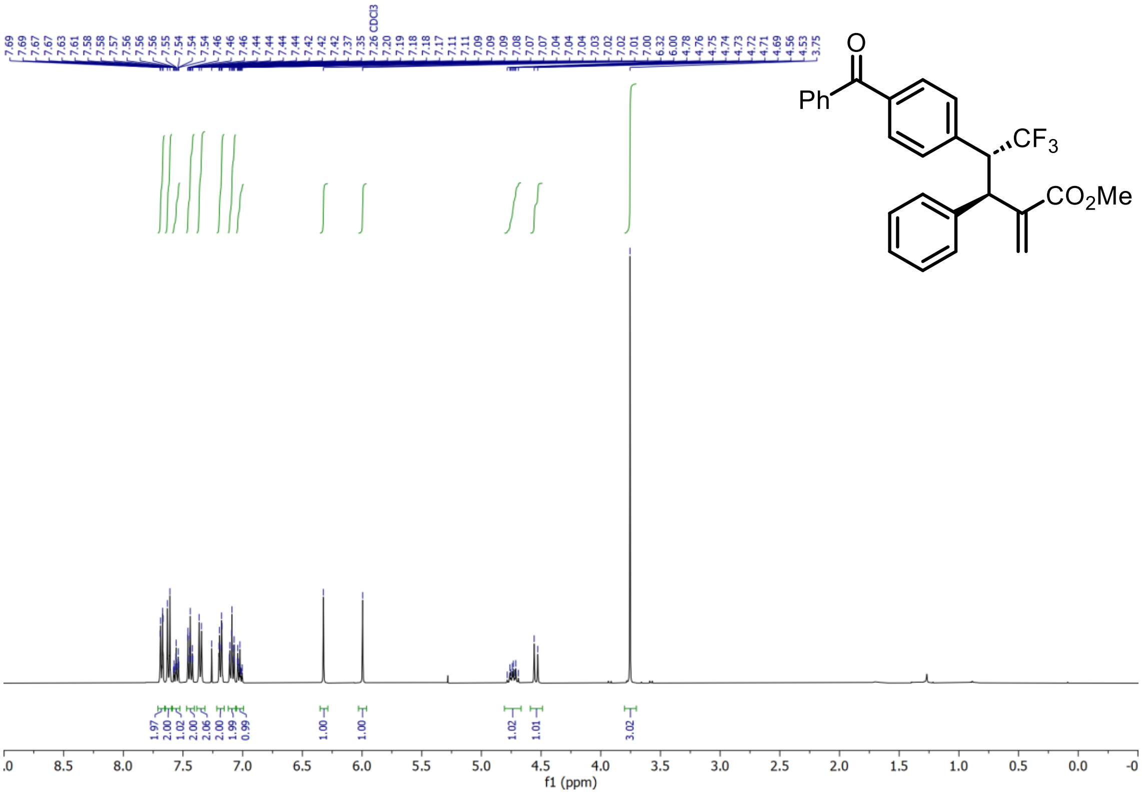

^{1}H NMR (400 MHz, $CDCl_3$) spectra of **3p**.

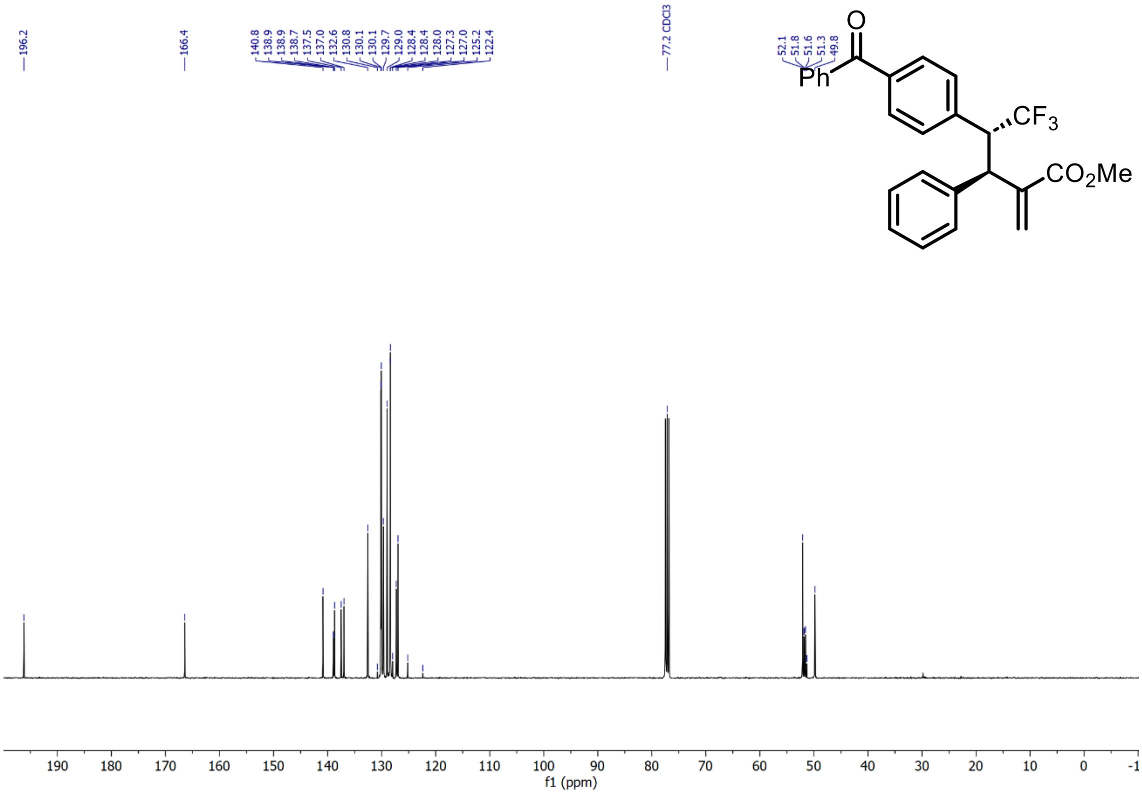

^{13}C NMR (101 MHz, $CDCl_3$) spectra of **3p**.

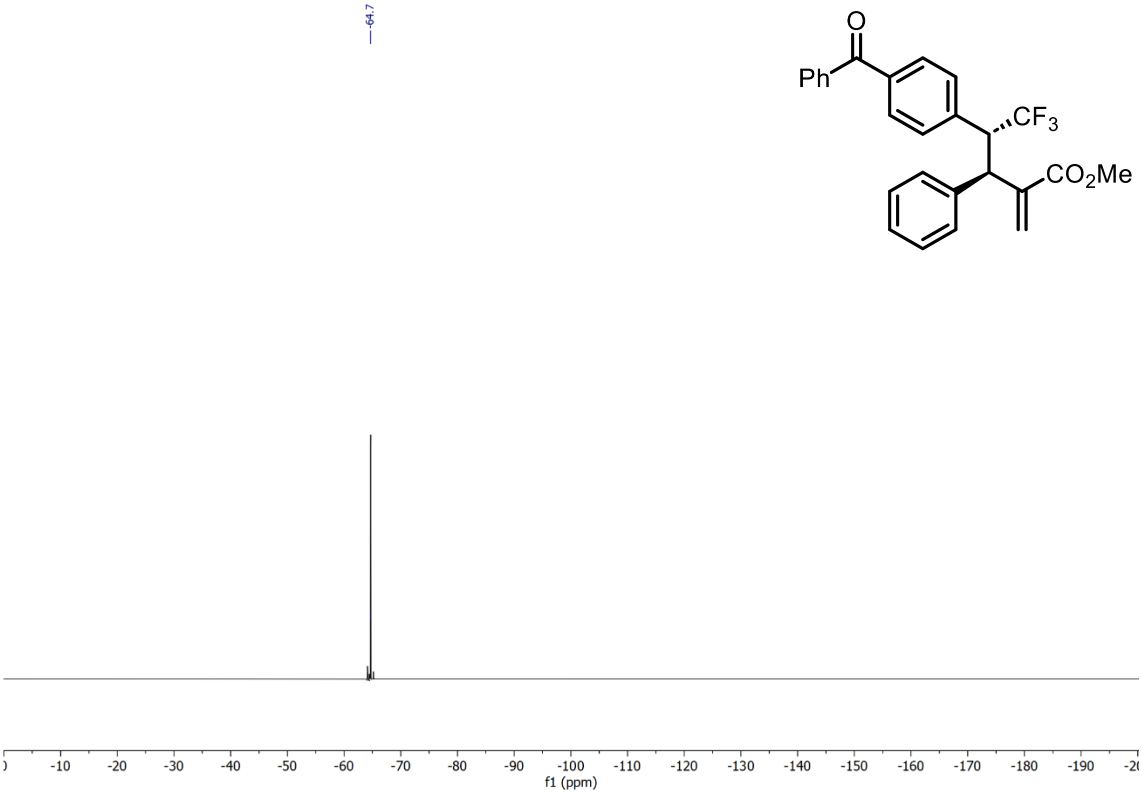

^{19}F NMR (376 MHz, $CDCl_3$) spectra of **3p**.

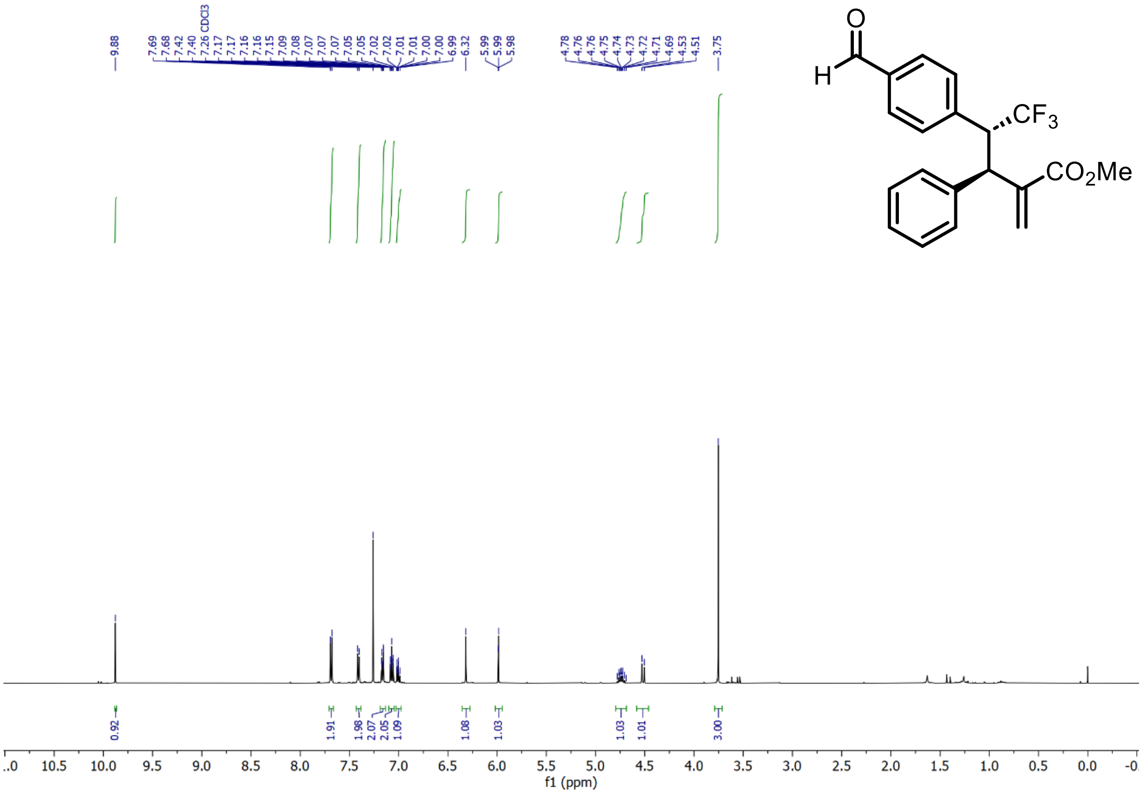

^{1}H NMR (500 MHz, $CDCl_3$) spectra of **3q**.

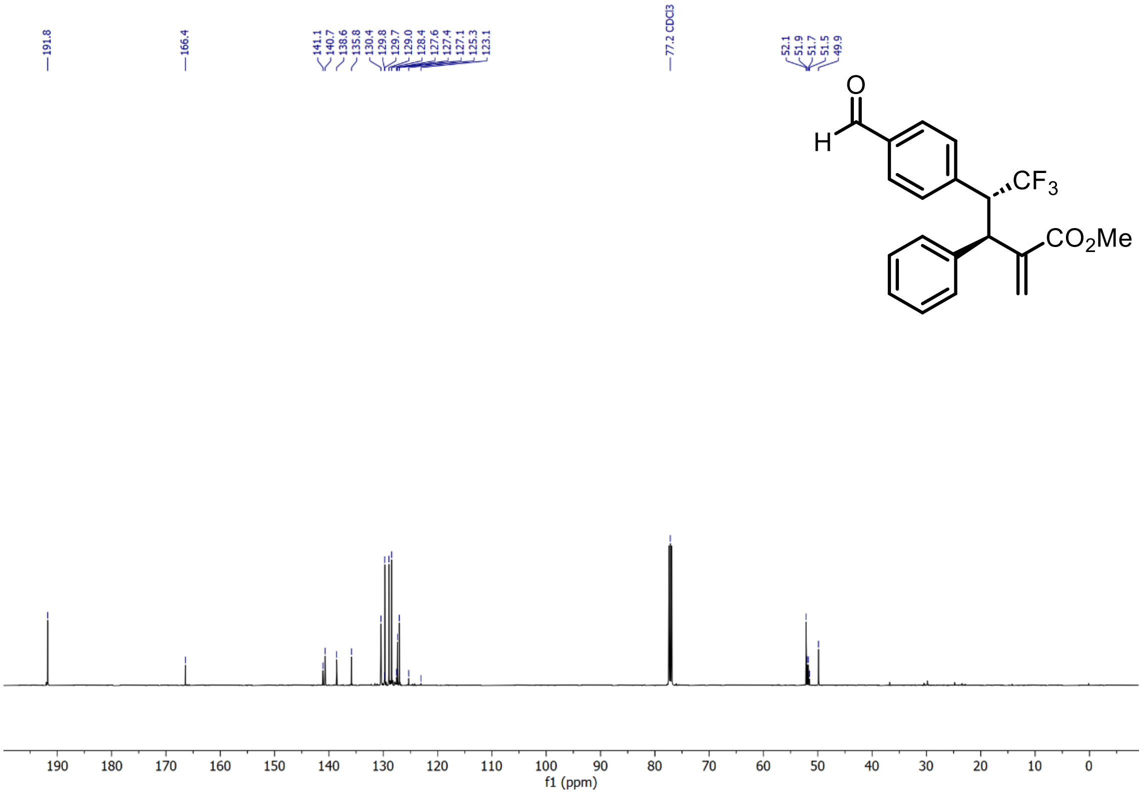

^{13}C NMR (126 MHz, $CDCl_3$) spectra of **3q**.

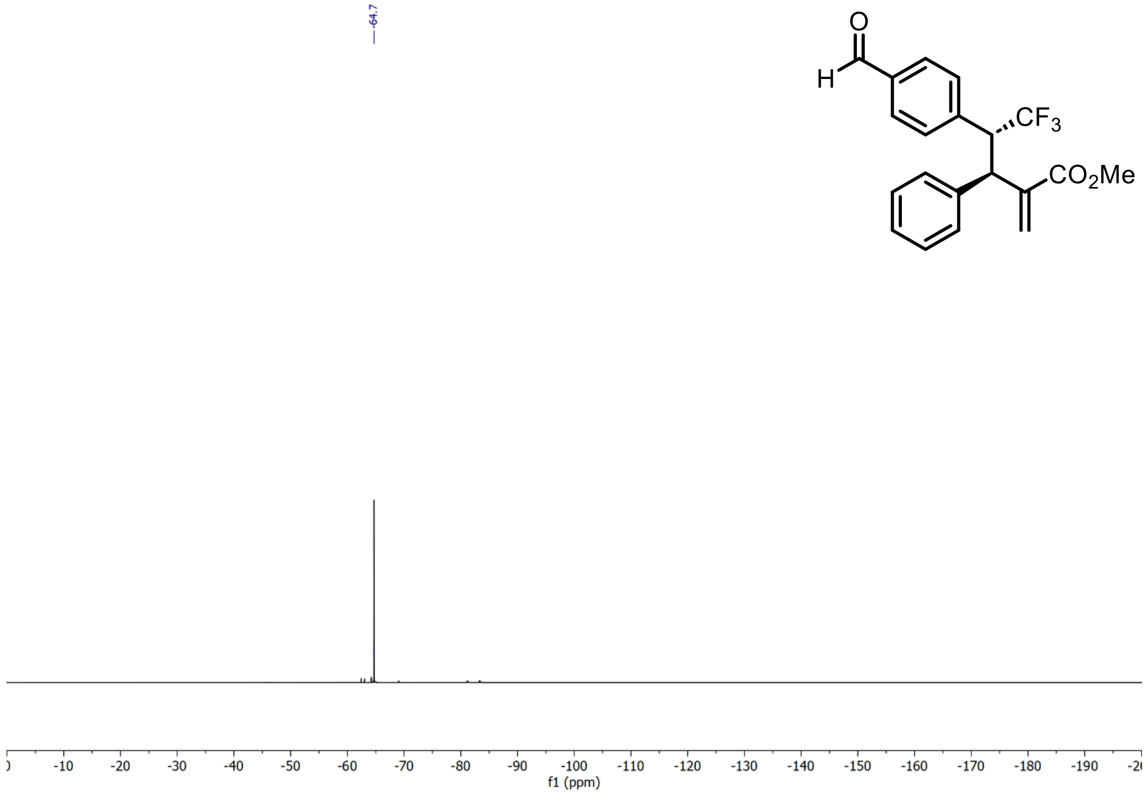

^{19}F NMR (471 MHz, $CDCl_3$) spectra of **3q**.

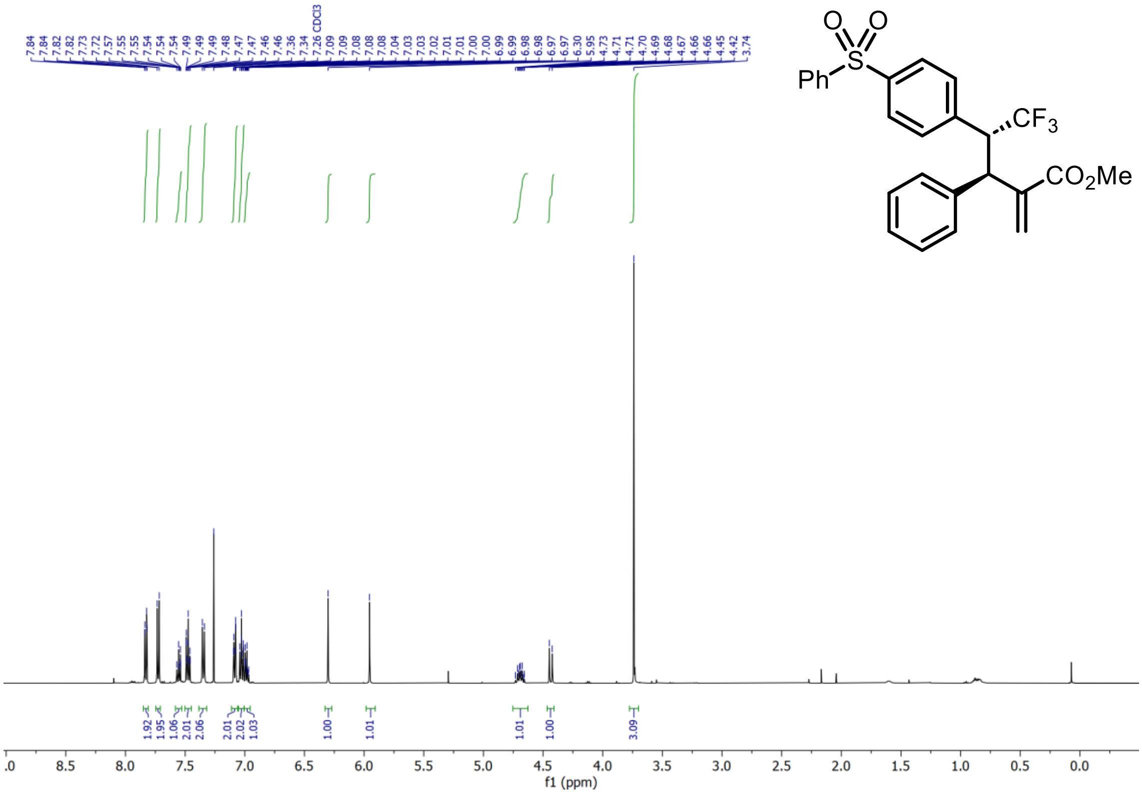

^{1}H NMR (500 MHz, $CDCl_3$) spectra of **3r**.

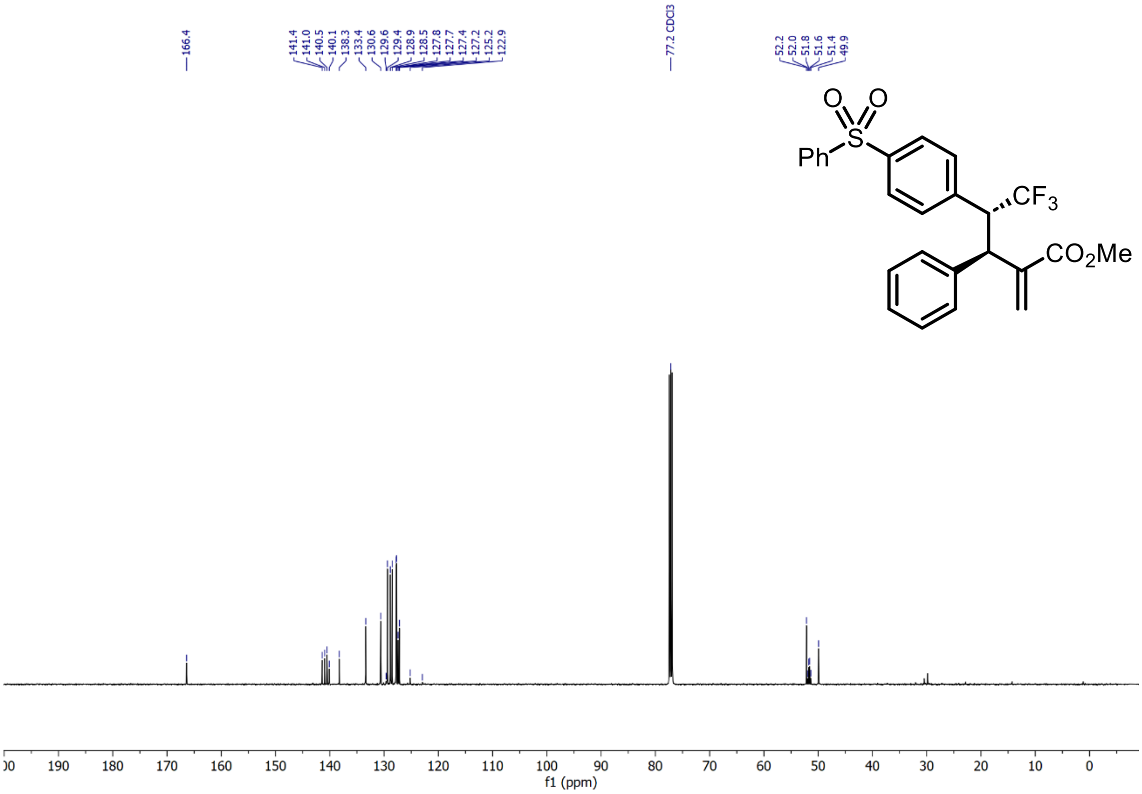

^{13}C NMR (126 MHz, $CDCl_3$) spectra of **3r**.

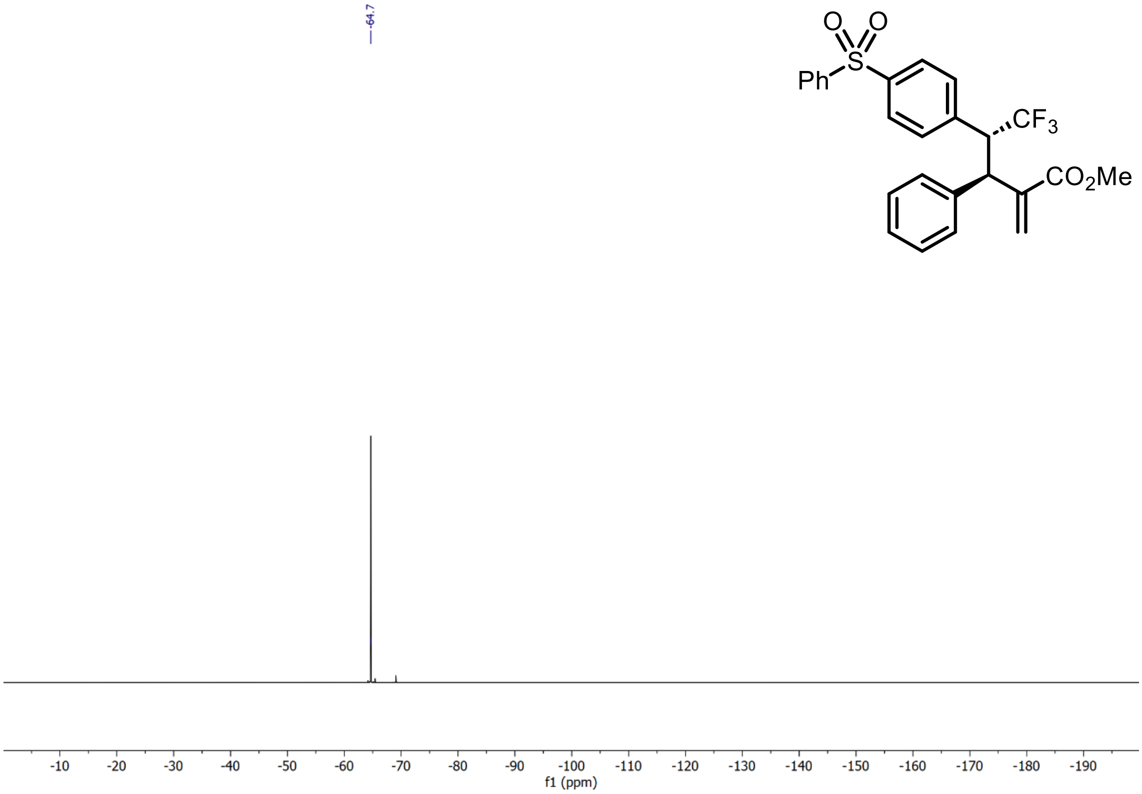

^{19}F NMR (471 MHz, $CDCl_3$) spectra of **3r**.

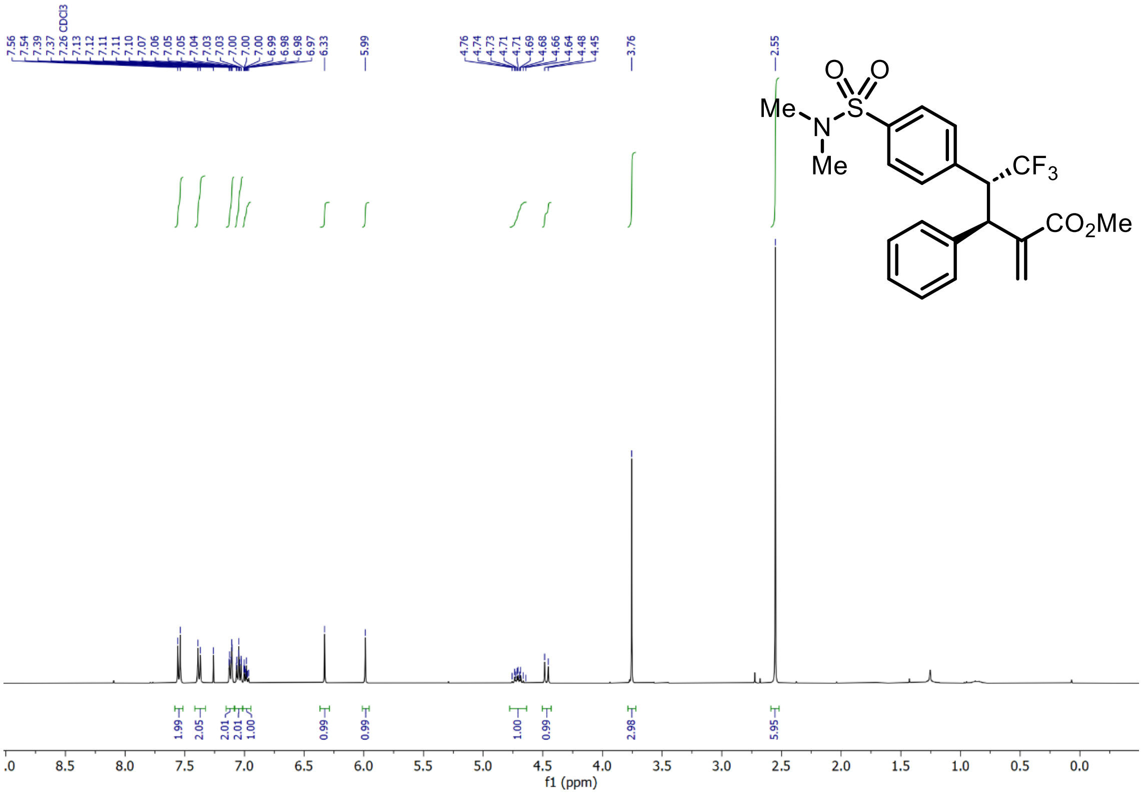

^{1}H NMR (400 MHz, $CDCl_3$) spectra of **3s**.

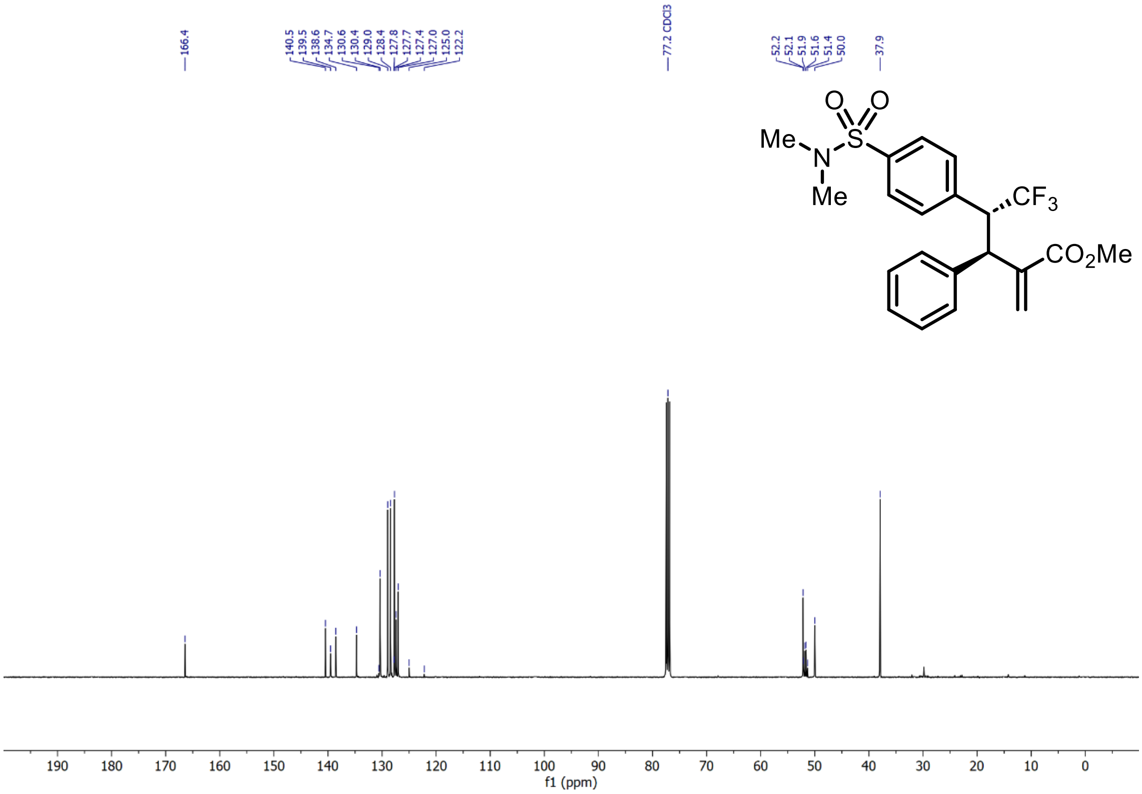

^{13}C NMR (101 MHz, $CDCl_3$) spectra of **3s**.

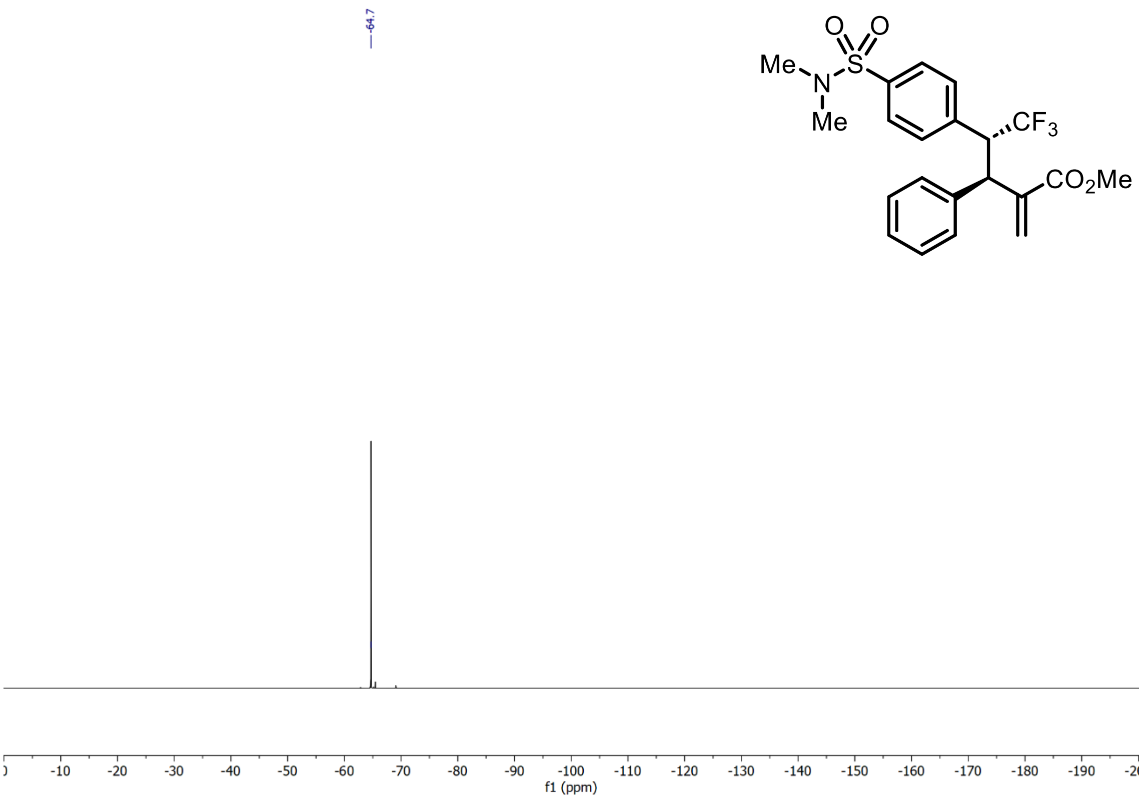

^{19}F NMR (471 MHz, $CDCl_3$) spectra of **3s**.

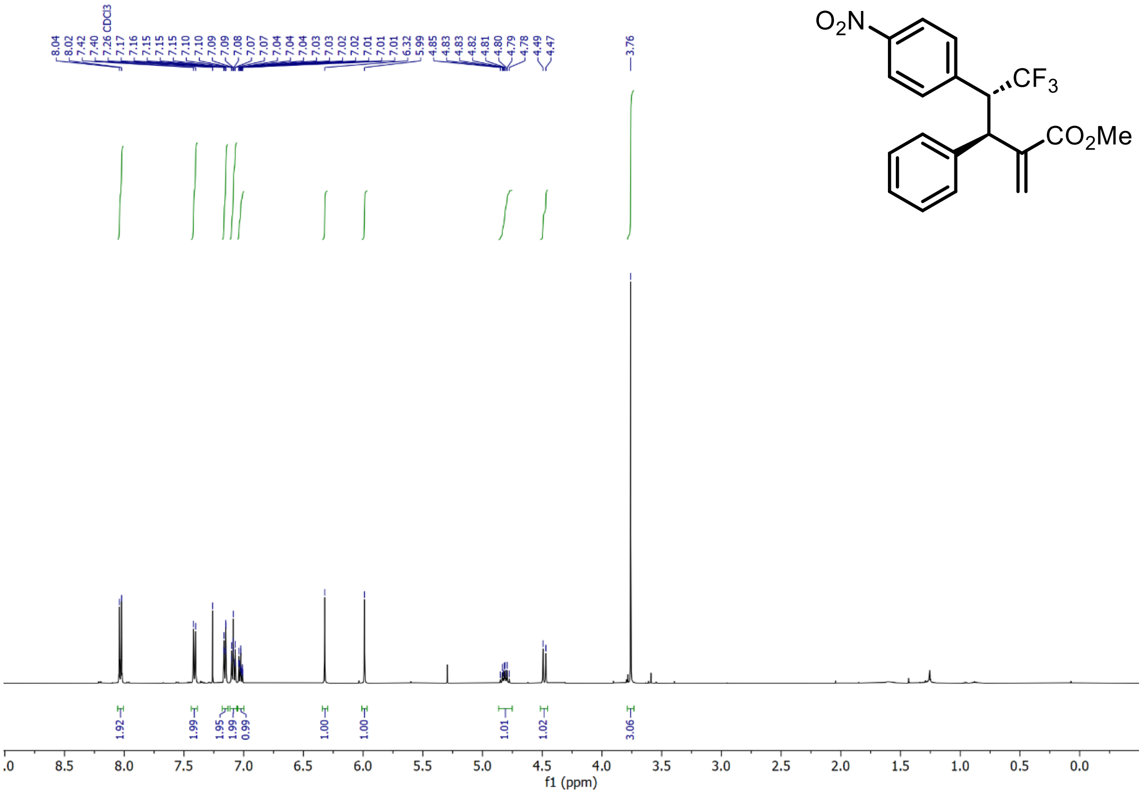

^{1}H NMR (400 MHz, $CDCl_3$) spectra of **3t**.

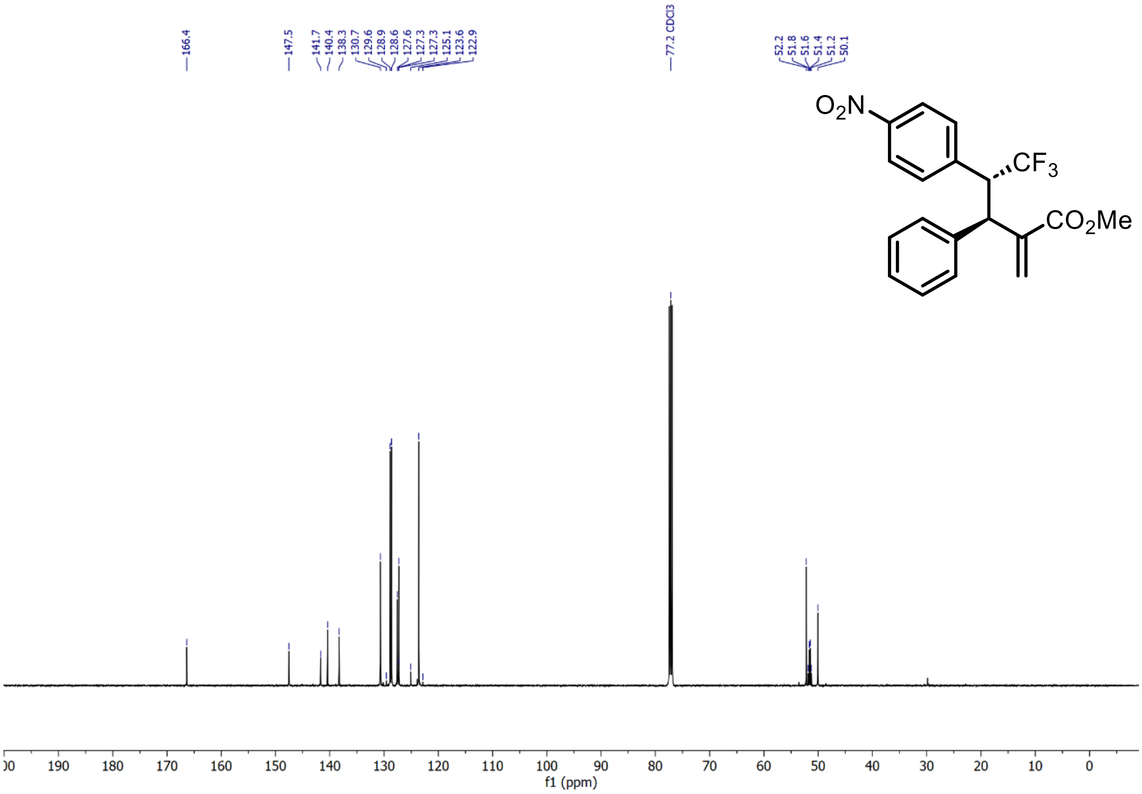

^{13}C NMR (101 MHz, $CDCl_3$) spectra of **3t**.

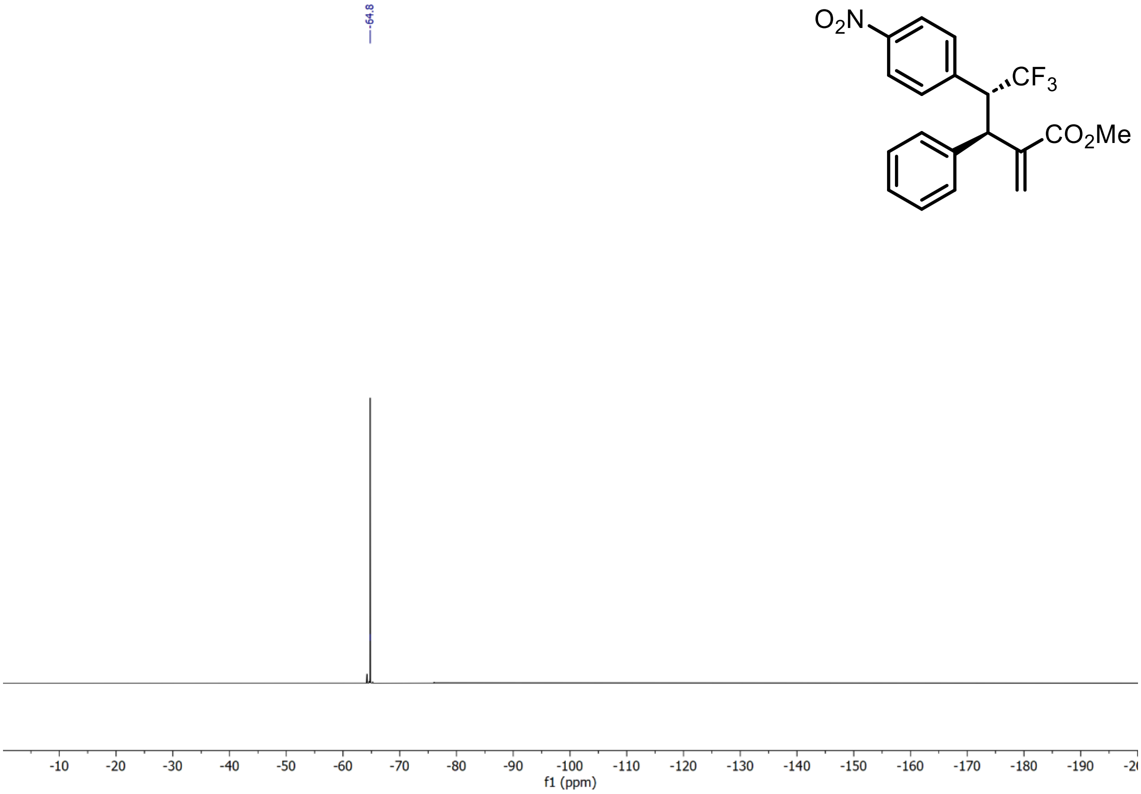

^{19}F NMR (471 MHz, $CDCl_3$) spectra of **3t**.

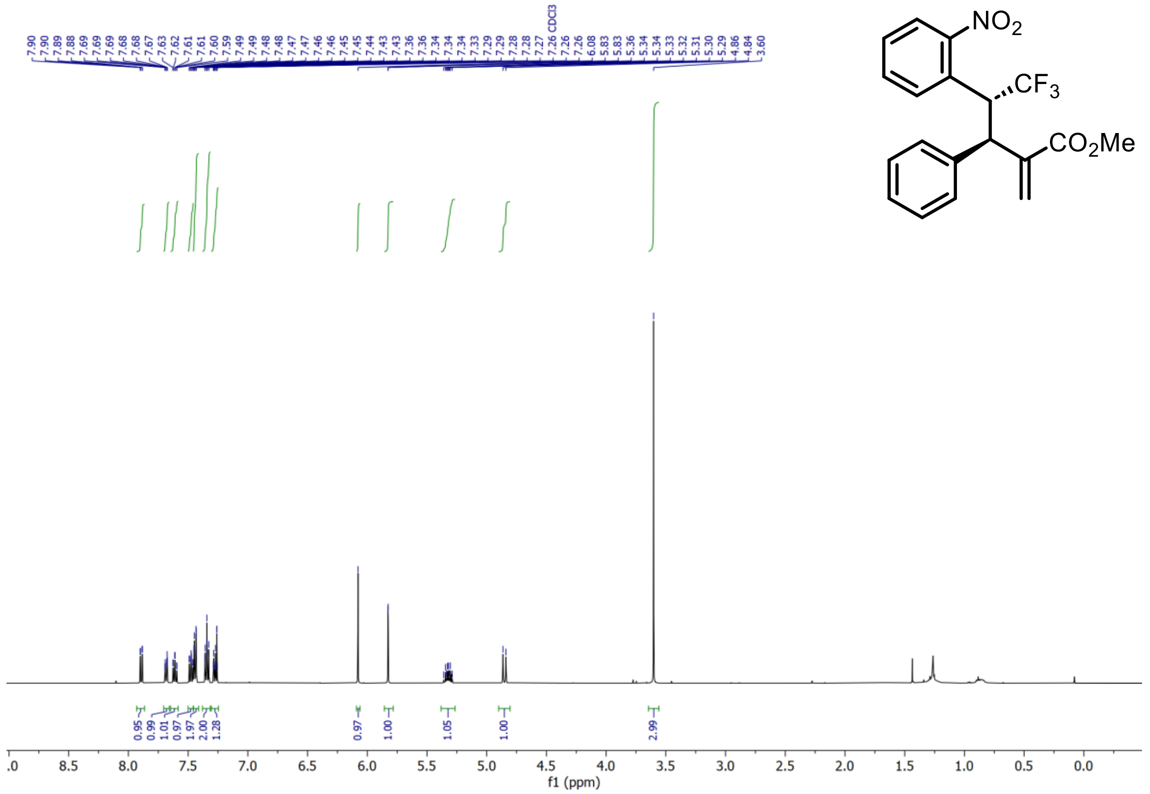

^{1}H NMR (500 MHz, CDCl3) spectra of **3u**.

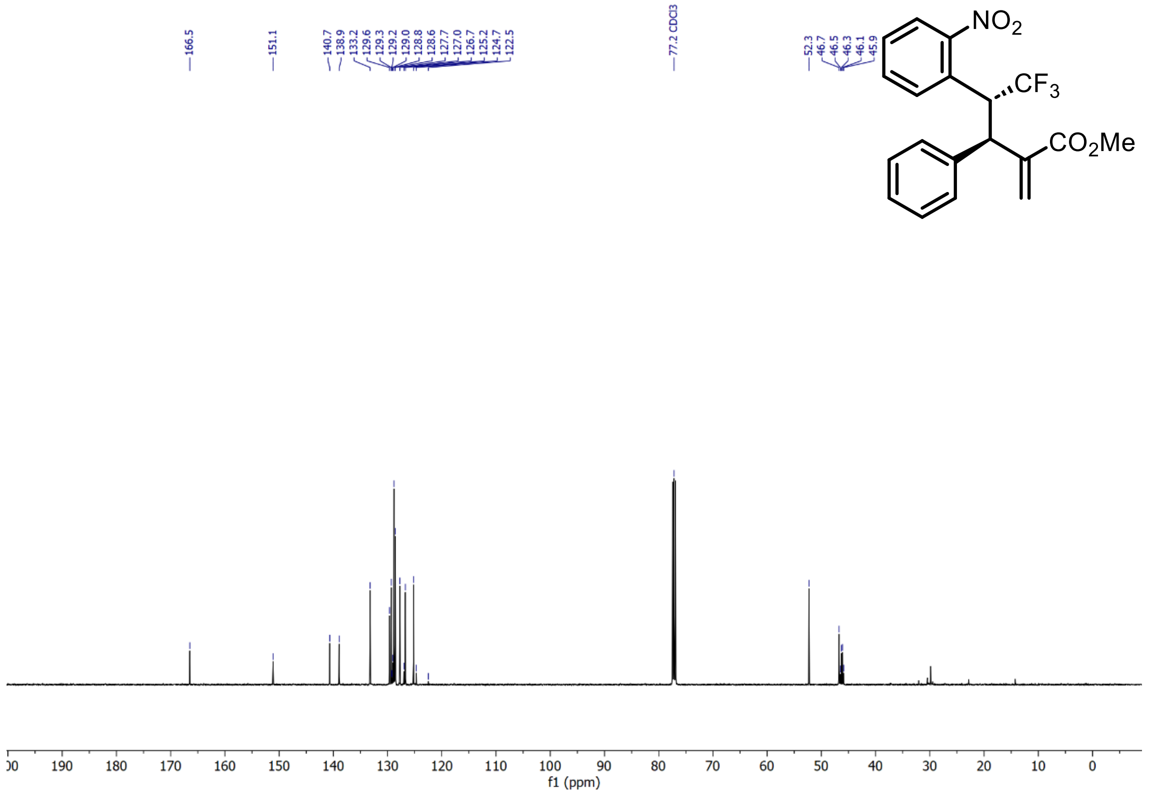

^{13}C NMR (126 MHz, CDCl3) spectra of **3u**.

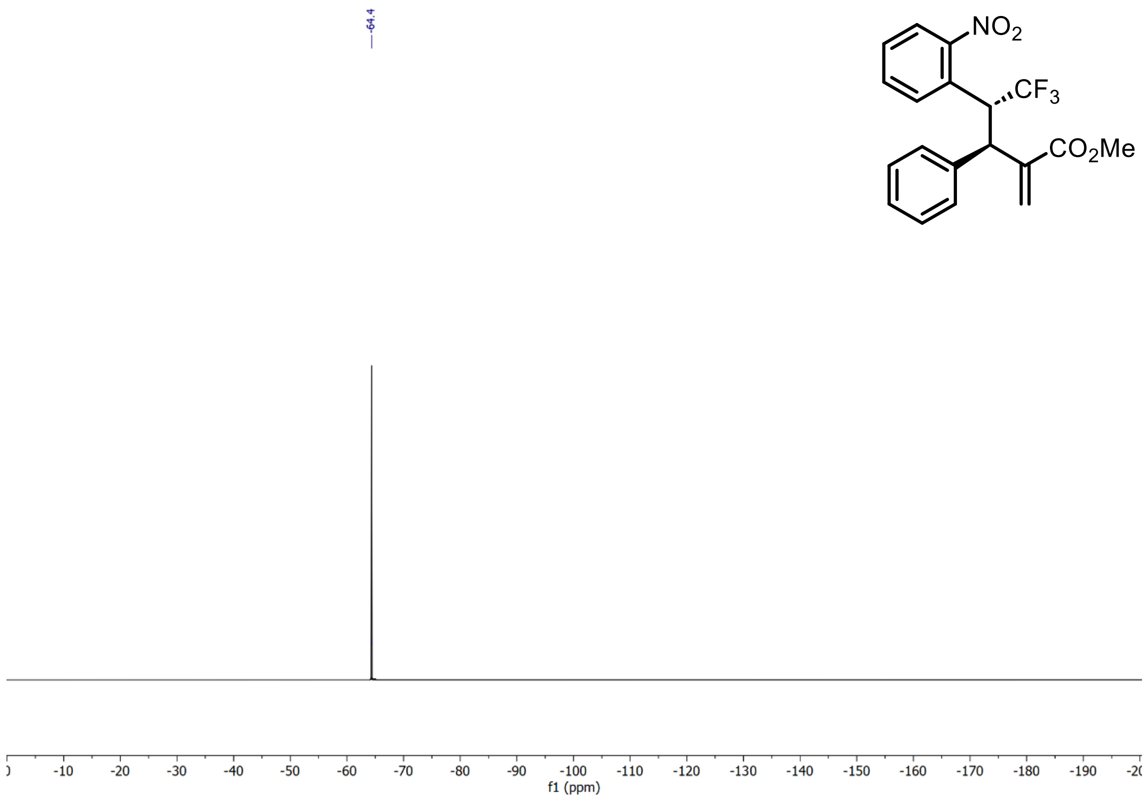

^{19}F NMR (471 MHz, $CDCl_3$) spectra of **3u**.

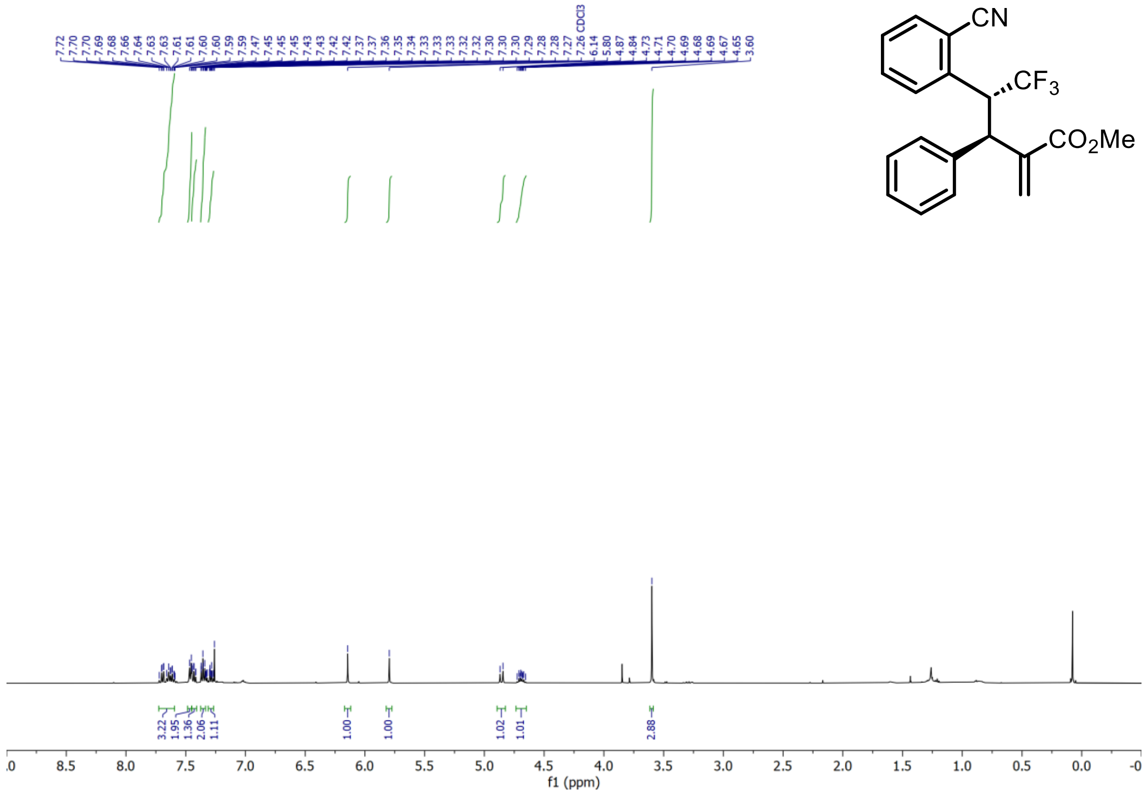

^{1}H NMR (500 MHz, $CDCl_3$) spectra of **3v**.

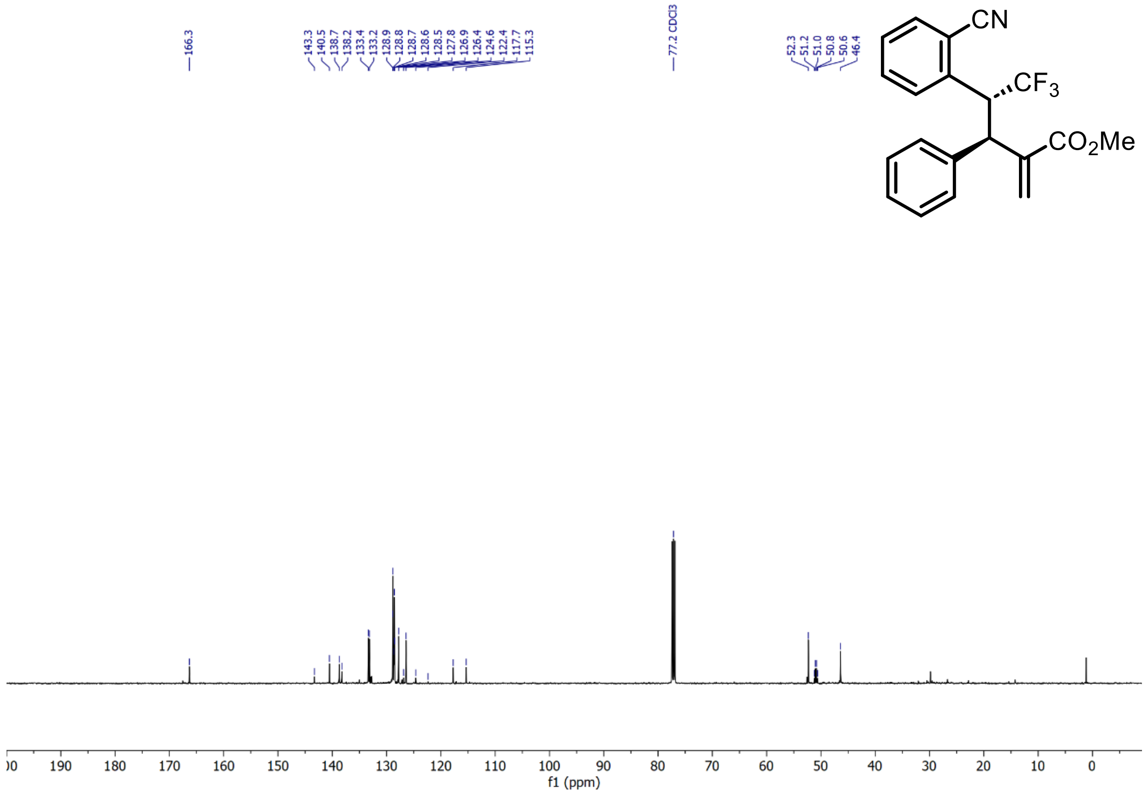

^{13}C NMR (126 MHz, $CDCl_3$) spectra of **3v**.

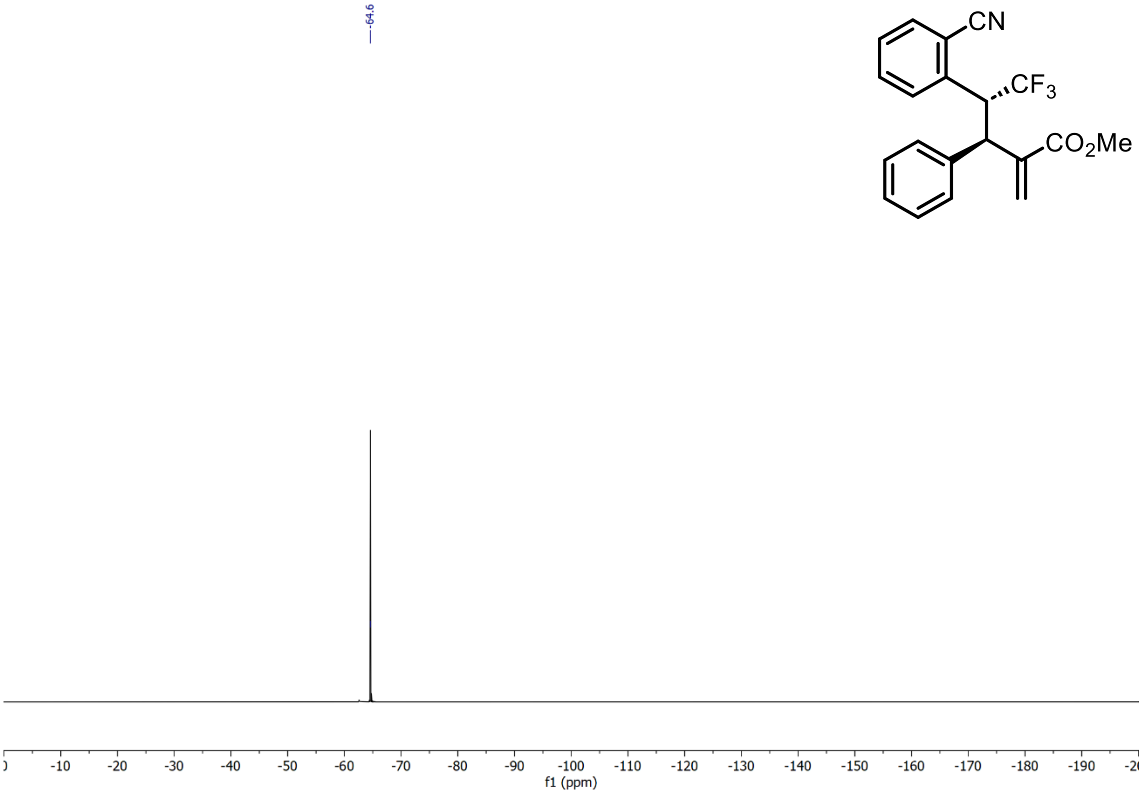

^{19}F NMR (471 MHz, $CDCl_3$) spectra of **3v**.

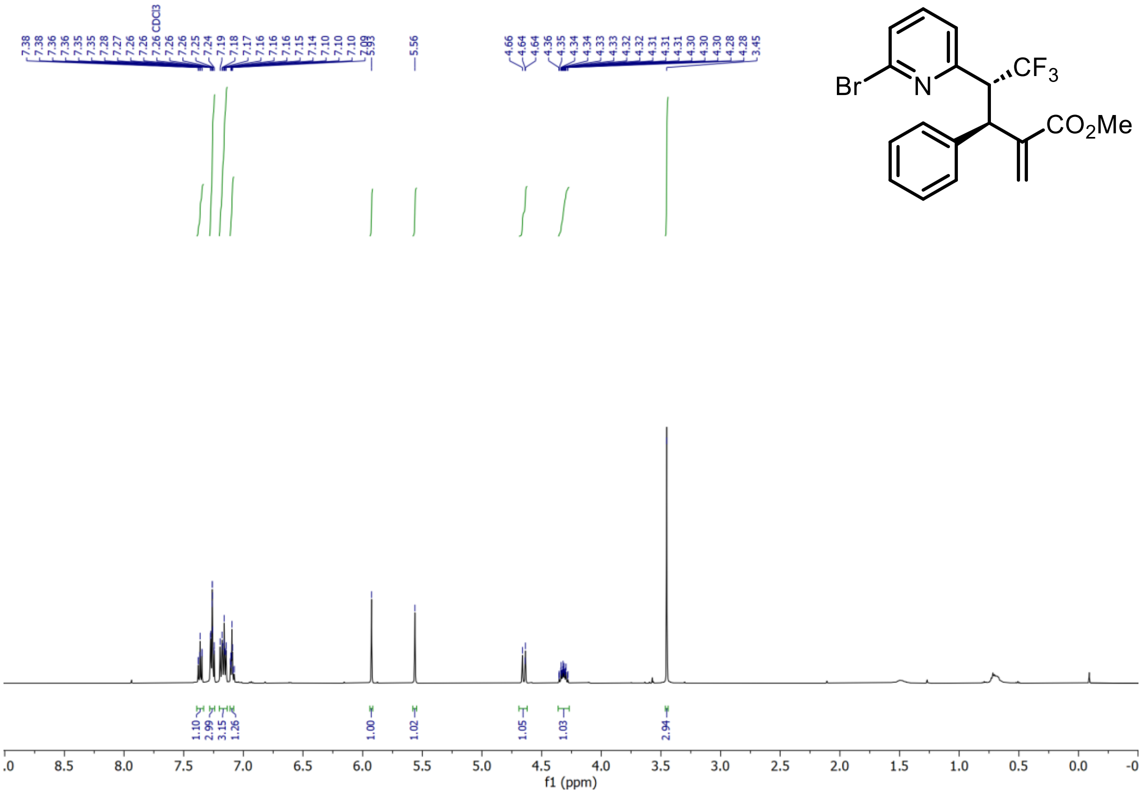

^{1}H NMR (400 MHz, $CDCl_3$) spectra of **3w**.

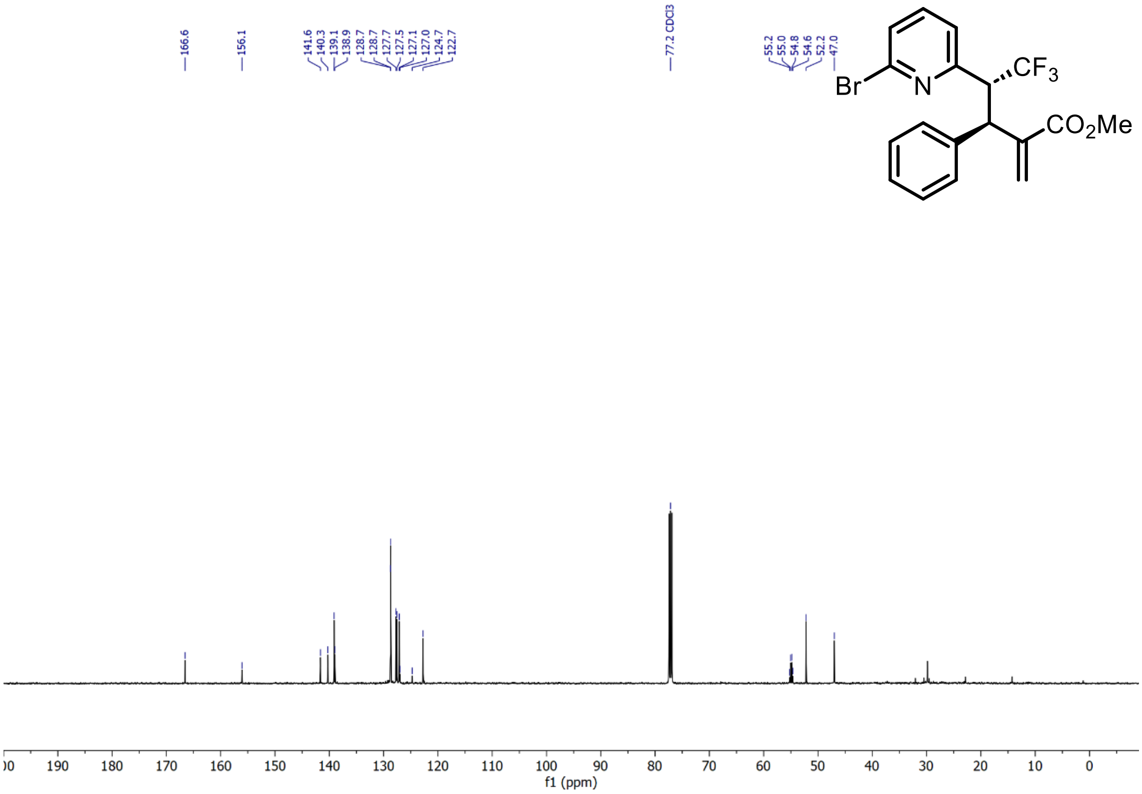

^{13}C NMR (101 MHz, $CDCl_3$) spectra of **3w**.

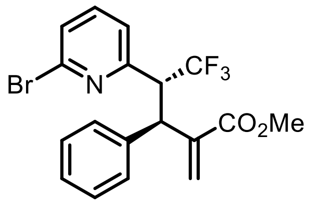

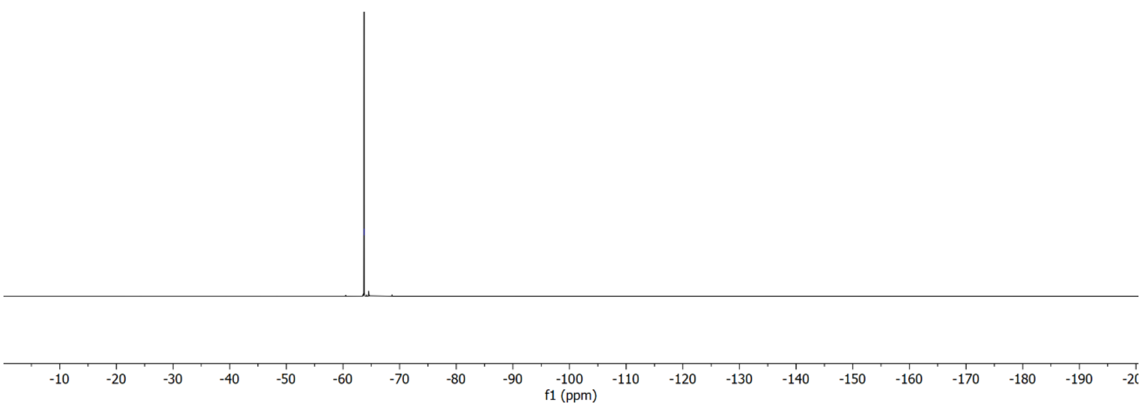

^{19}F NMR (376 MHz, $CDCl_3$) spectra of **3w**.

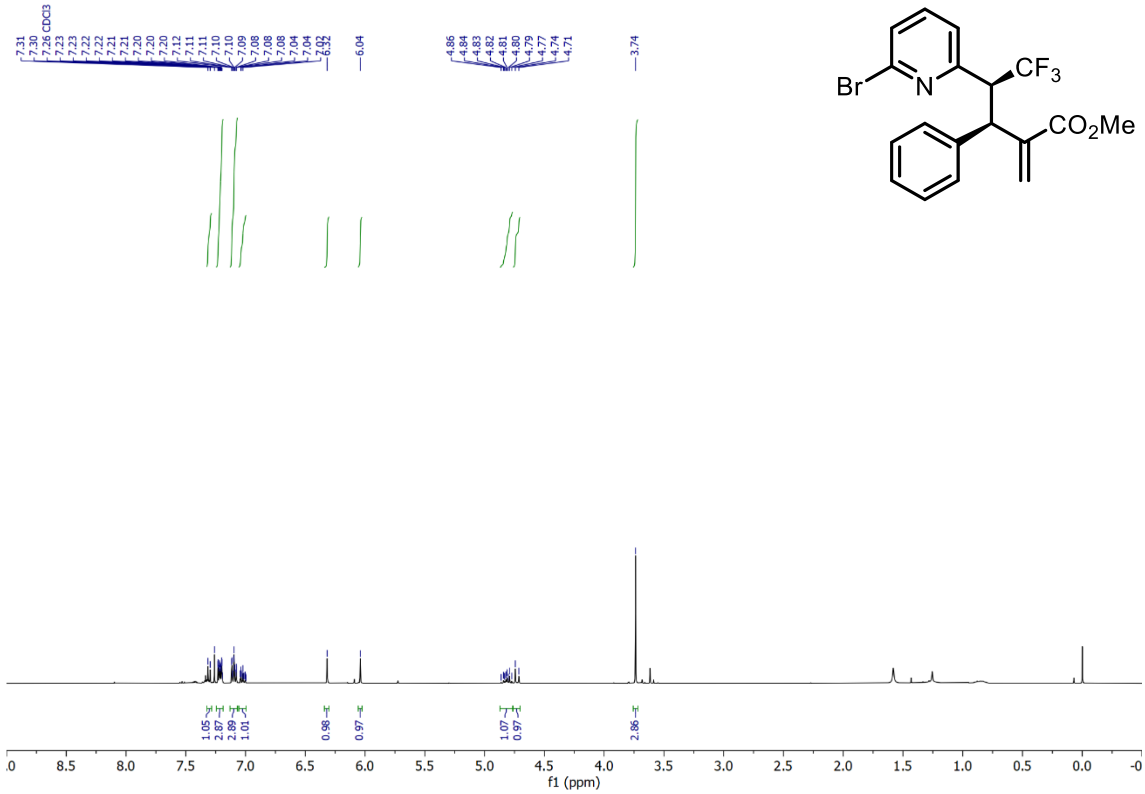

^{1}H NMR (400 MHz, $CDCl_3$) spectra of **3w'**.

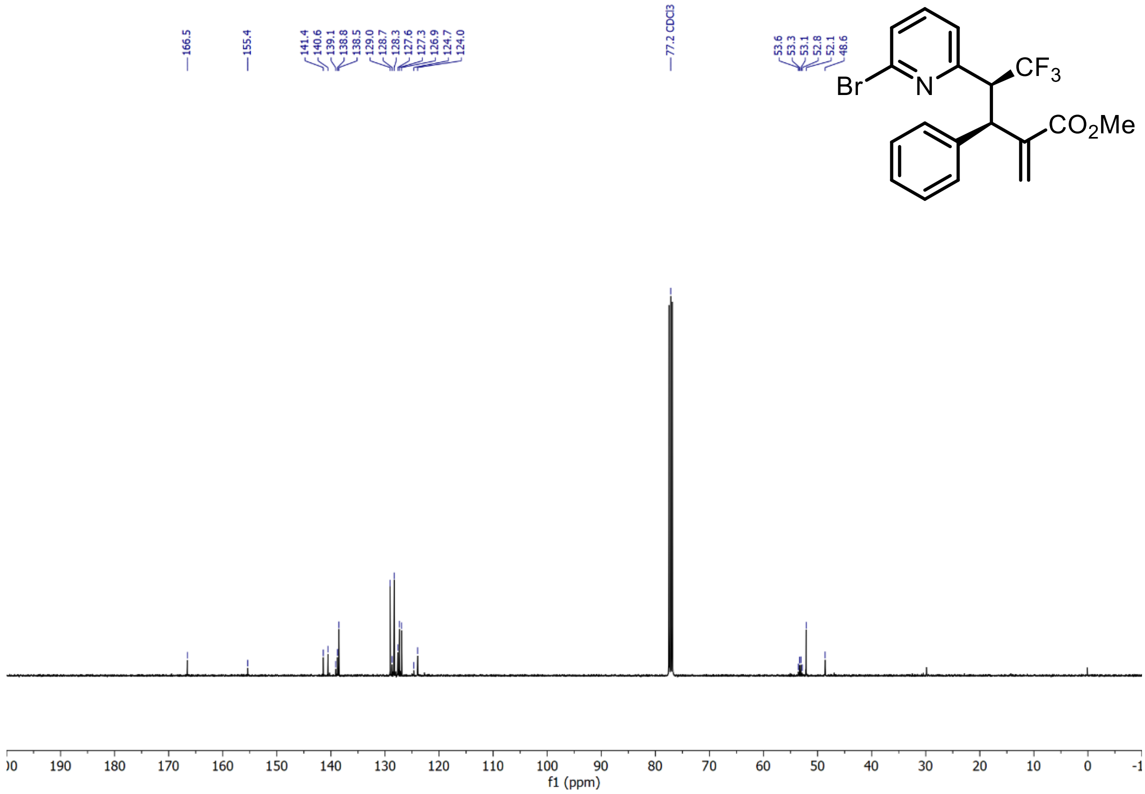

^{13}C NMR (101 MHz, $CDCl_3$) spectra of **3w'**.

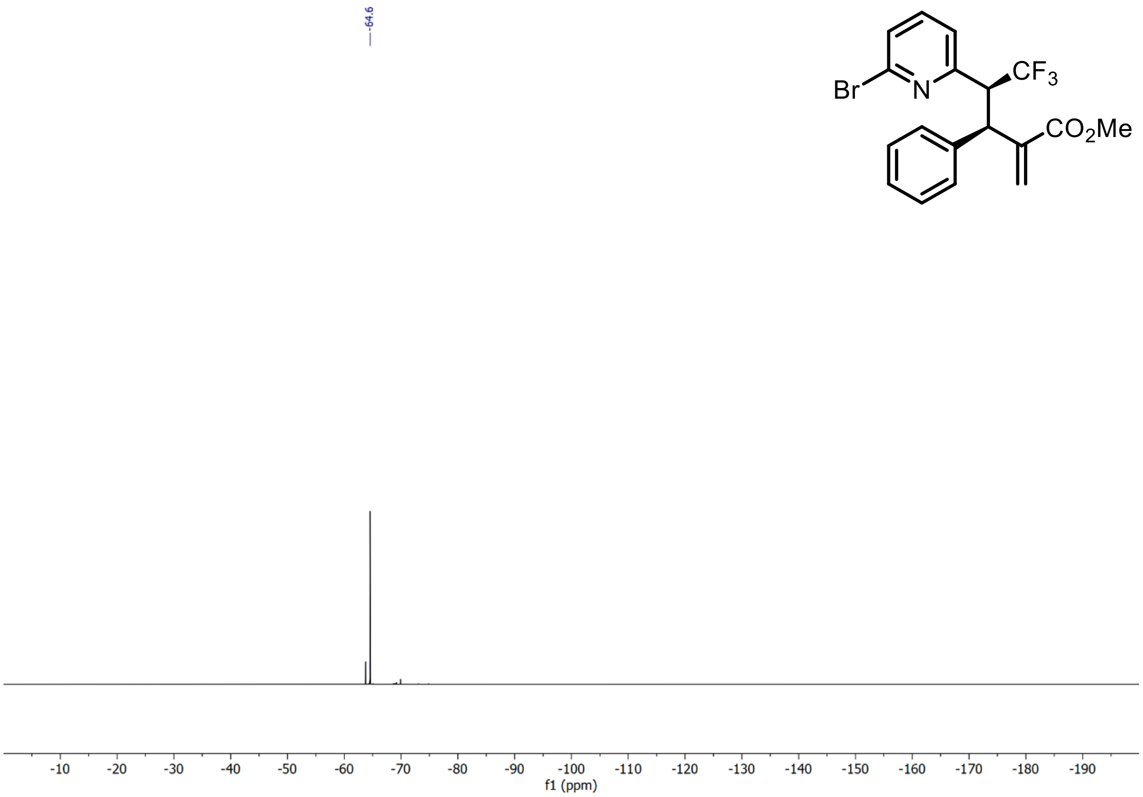

^{19}F NMR (376 MHz, $CDCl_3$) spectra of **3w'**.

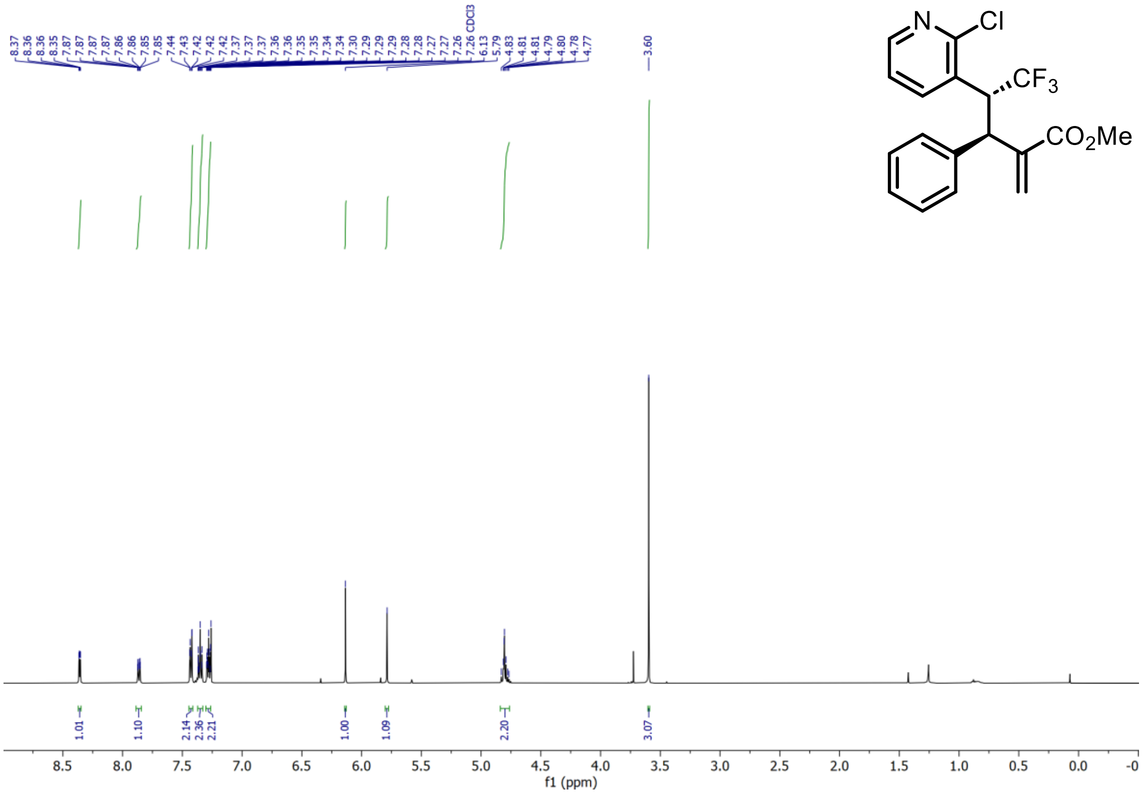

^{1}H NMR (500 MHz, $CDCl_3$) spectra of **3x**.

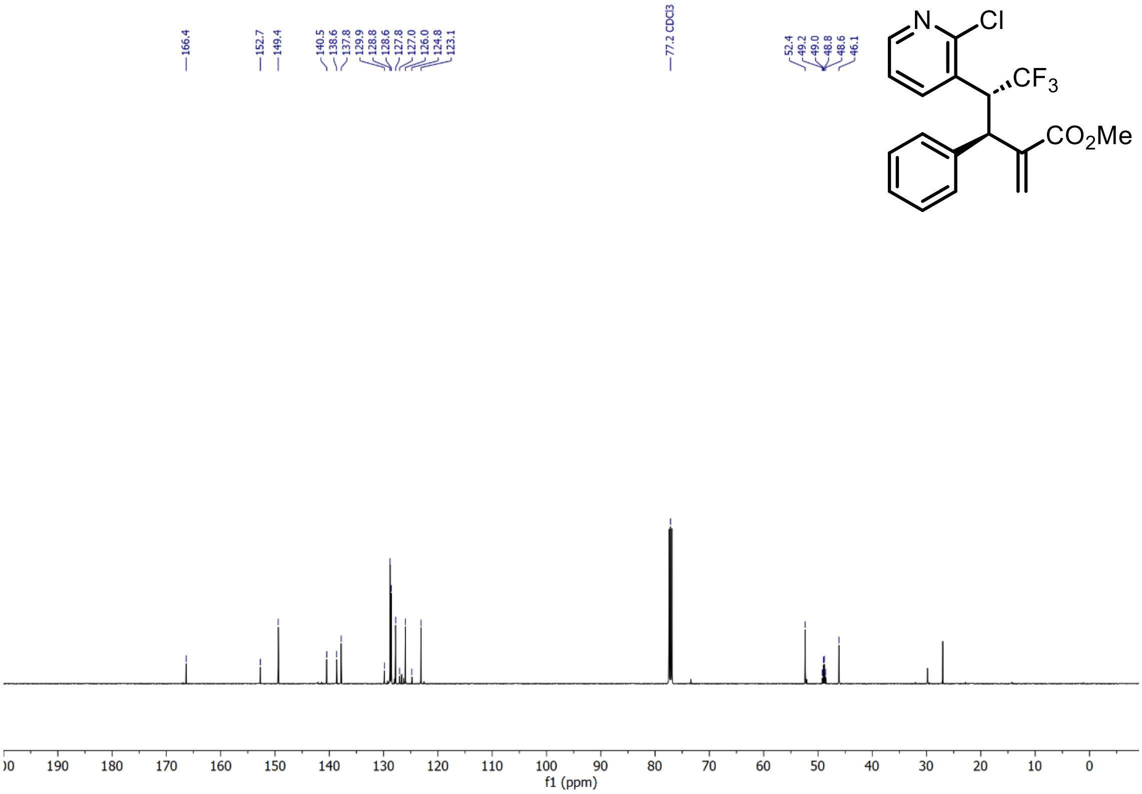

^{13}C NMR (126 MHz, $CDCl_3$) spectra of **3x**.

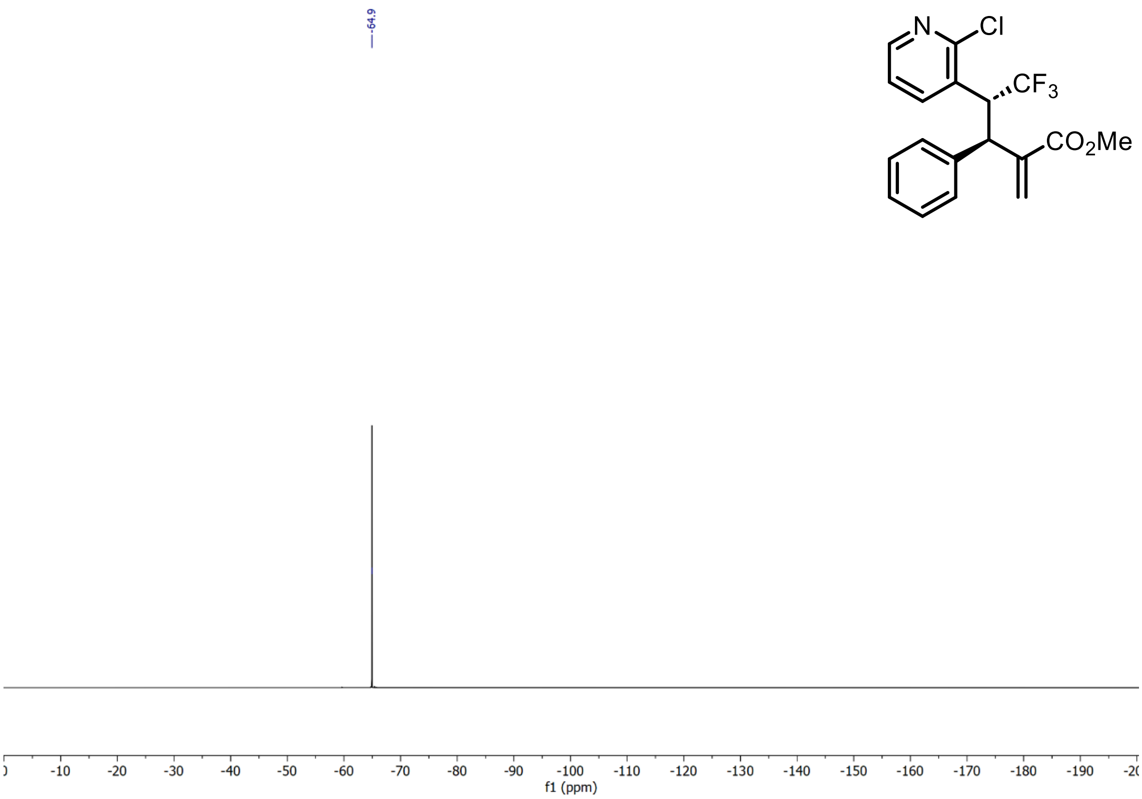

^{19}F NMR (471 MHz, $CDCl_3$) spectra of **3x**.

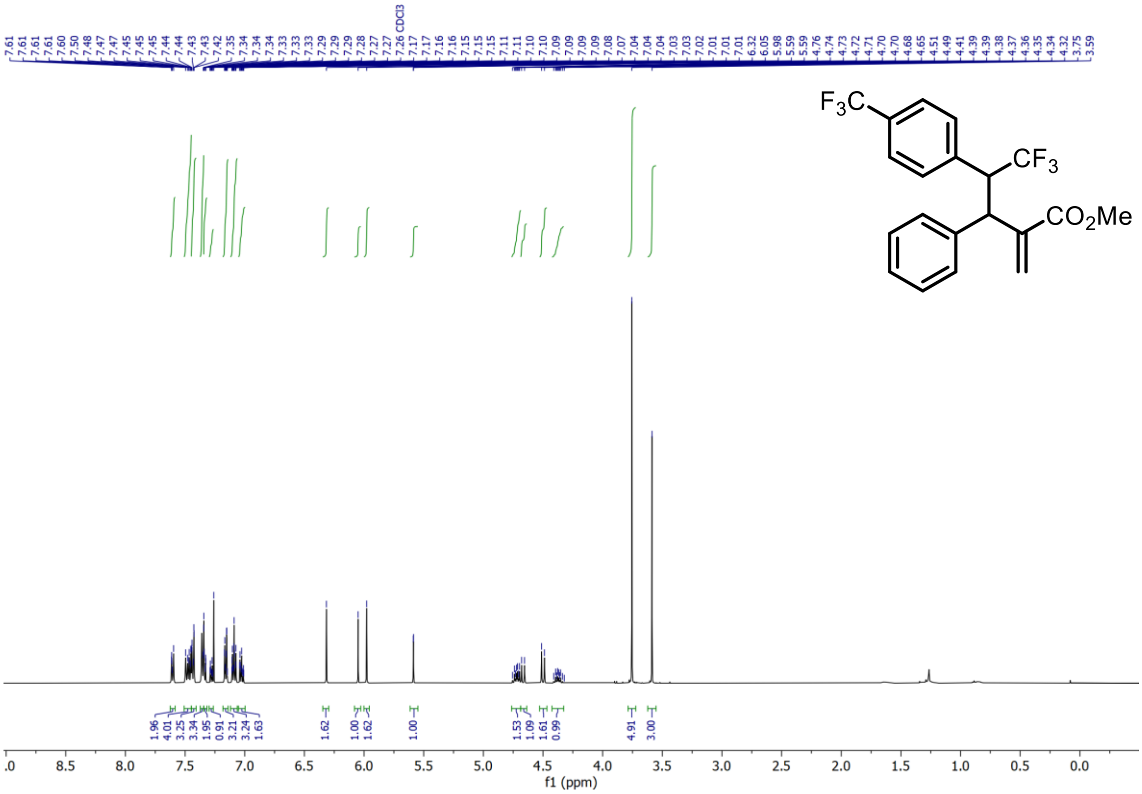

^{1}H NMR (500 MHz, $CDCl_3$) spectra of **3y** (mixture of diastereomers).

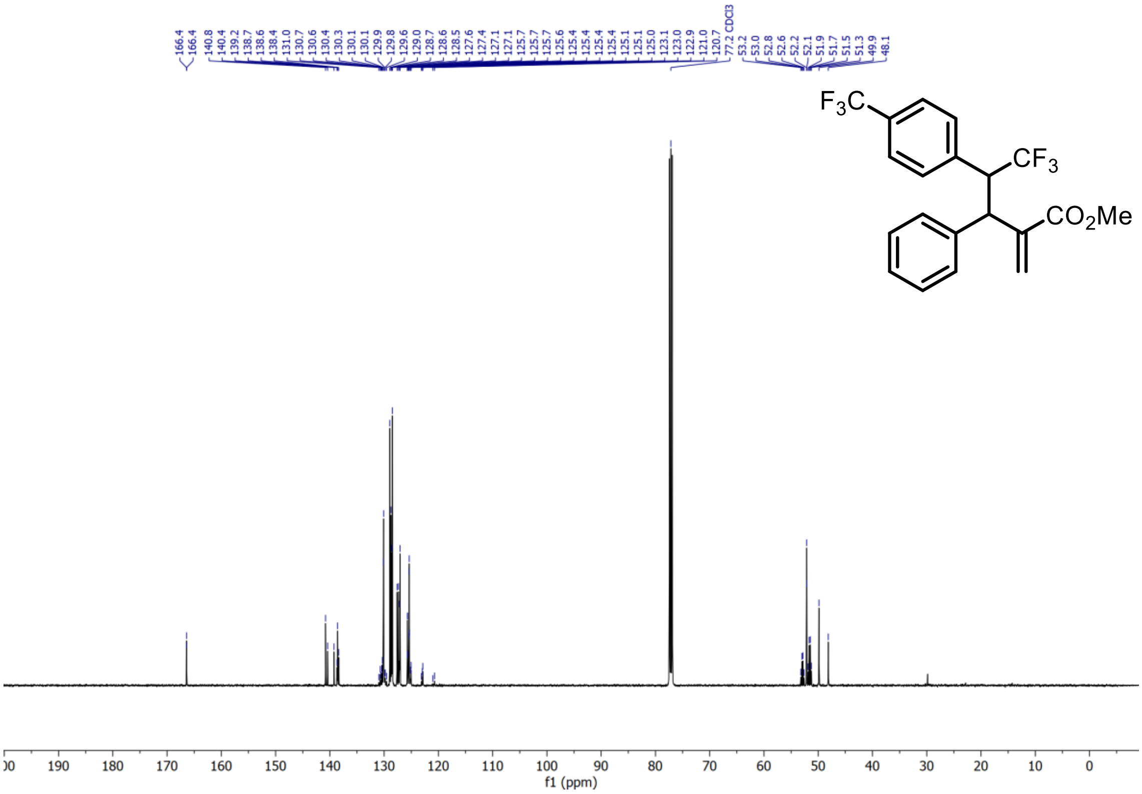

^{13}C NMR (126 MHz, $CDCl_3$) spectra of **3y** (mixture of diastereomers).

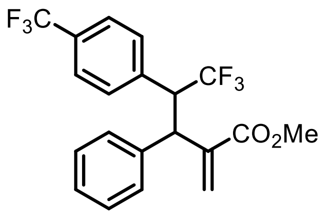

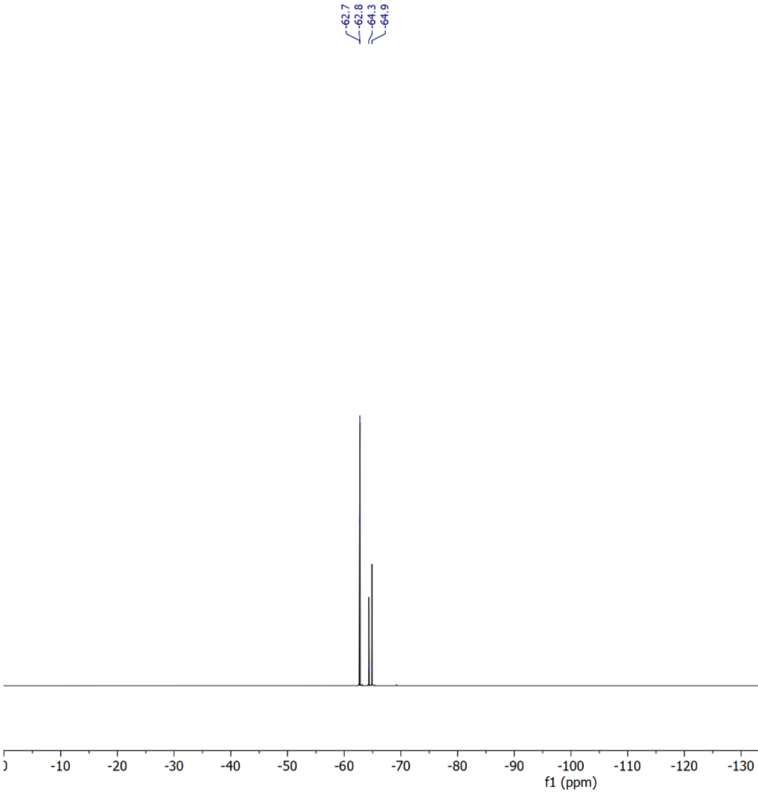

^{19}F NMR (471 MHz, $CDCl_3$) spectra of **3y** (mixture of diastereomers).

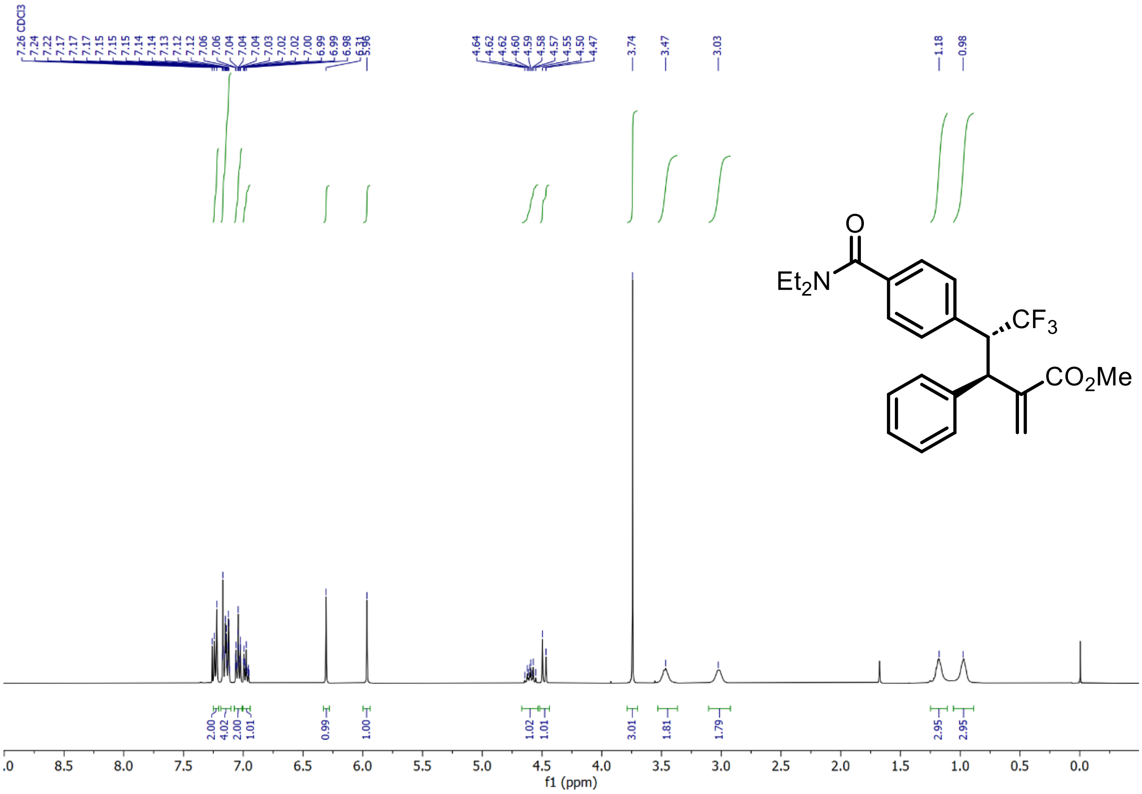

^{1}H NMR (400 MHz, $CDCl_3$) spectra of **3z**.

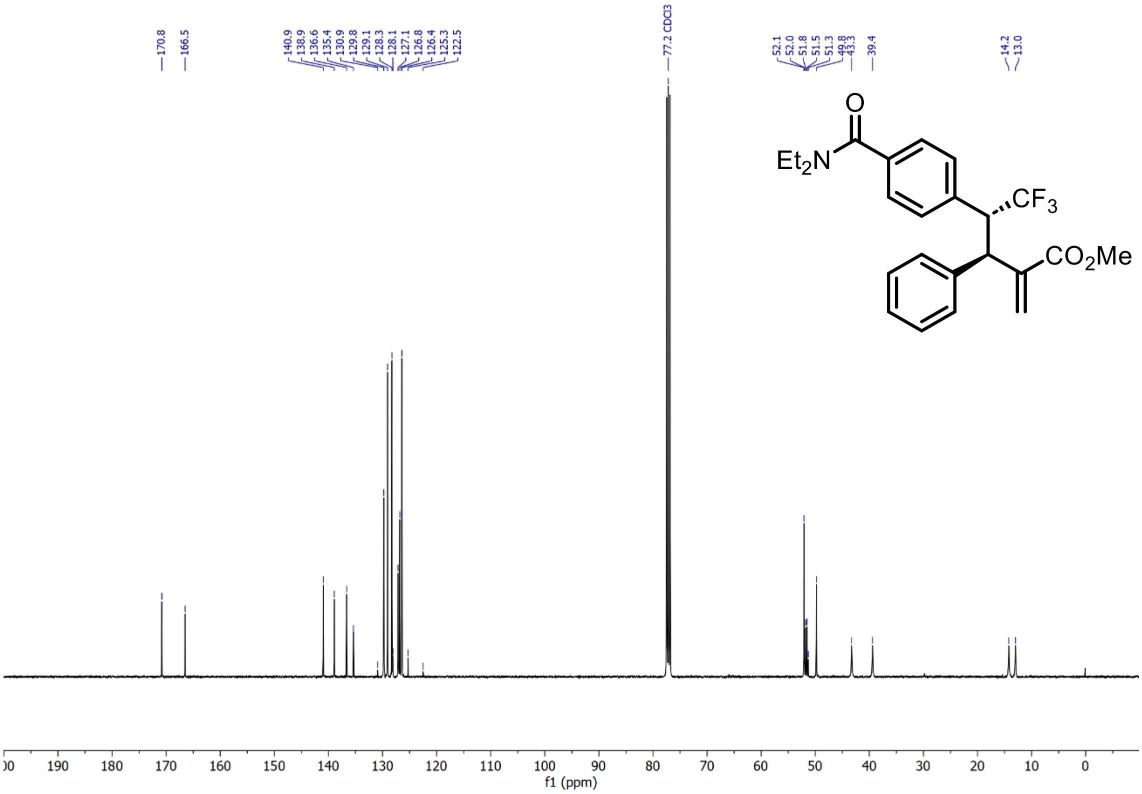

^{13}C NMR (101 MHz, $CDCl_3$) spectra of **3z**.

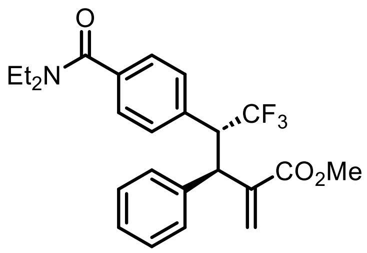

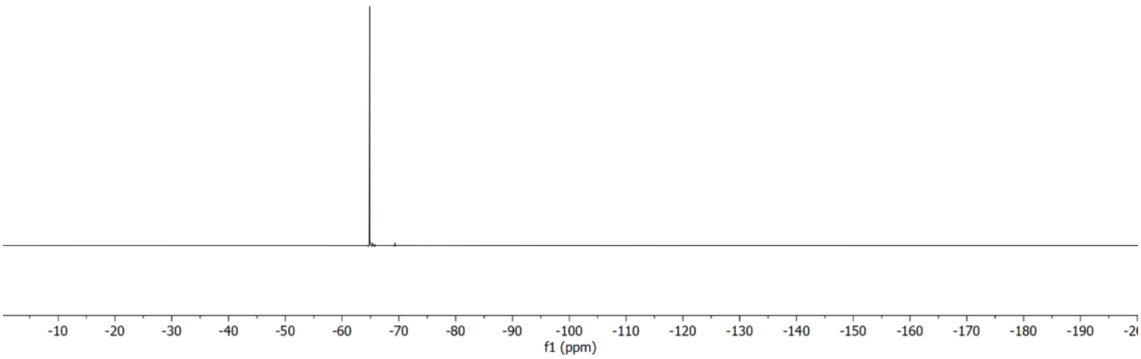

^{19}F NMR (376 MHz, $CDCl_3$) spectra of **3z**.

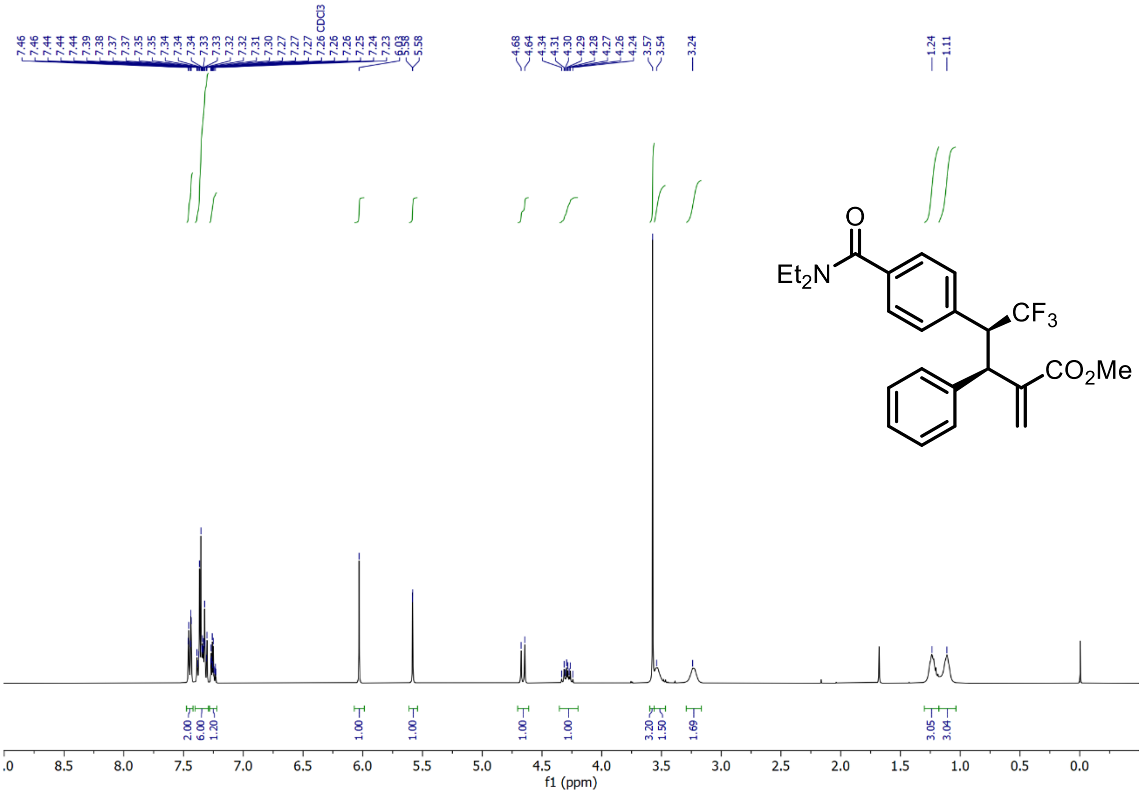

^{1}H NMR (400 MHz, $CDCl_3$) spectra of **3z’**.

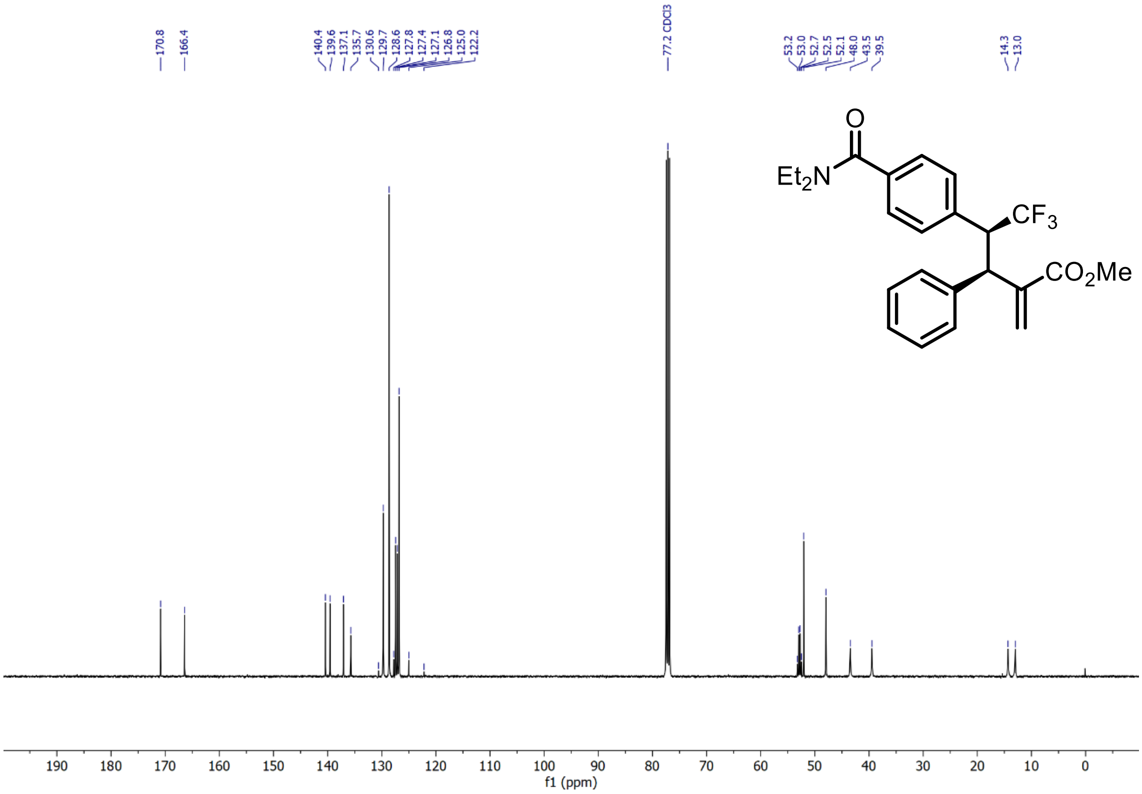

^{13}C NMR (101 MHz, $CDCl_3$) spectra of **3z’**.

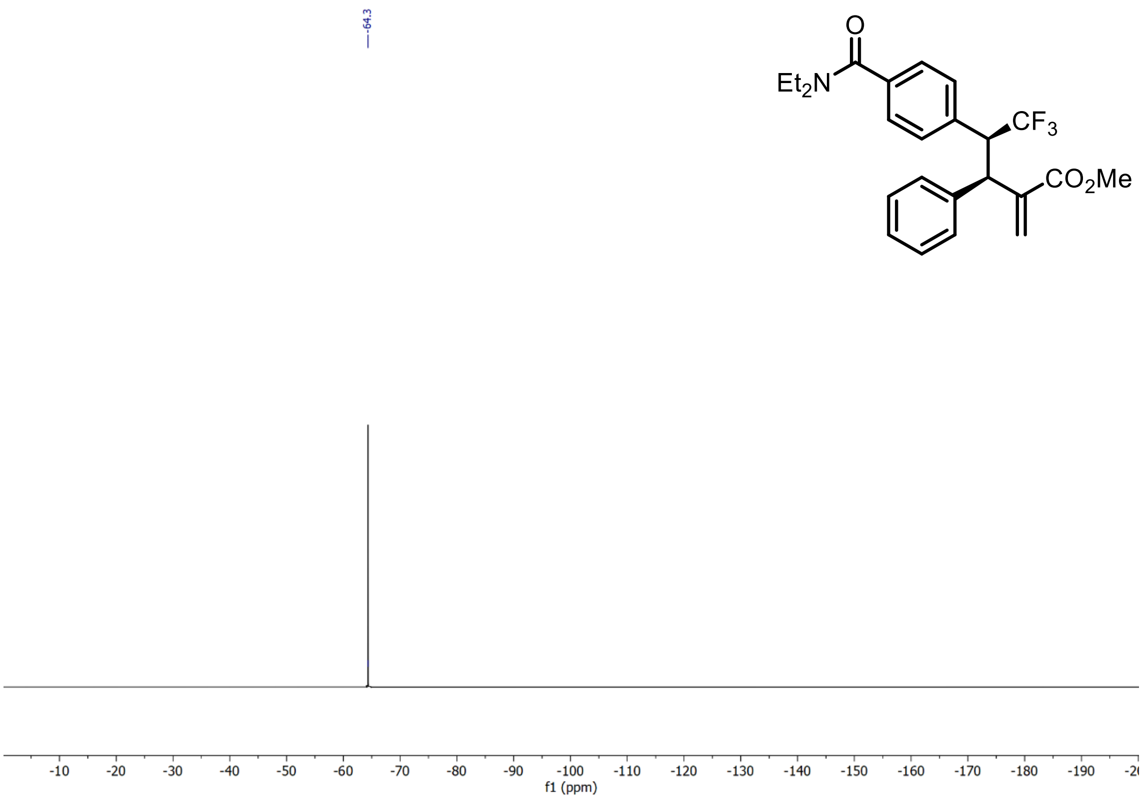

^{19}F NMR (376 MHz, $CDCl_3$) spectra of **3z'**.

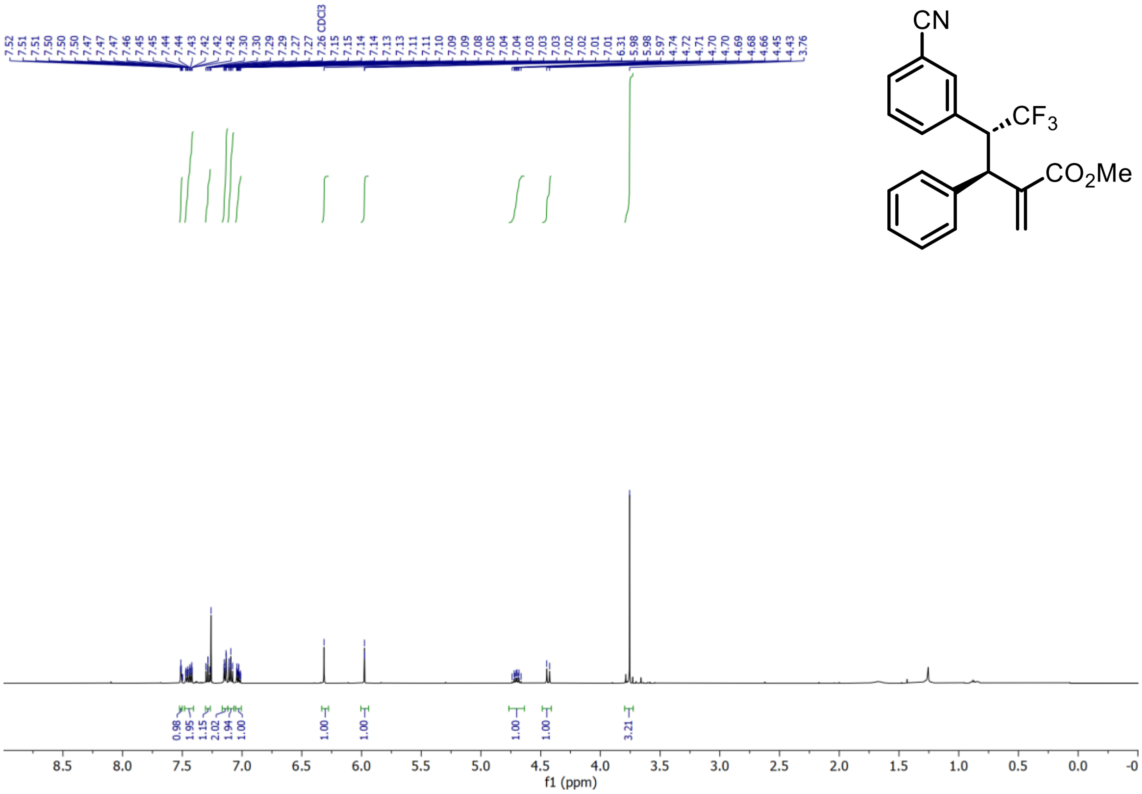

^{1}H NMR (500 MHz, $CDCl_3$) spectra of **3aa**.

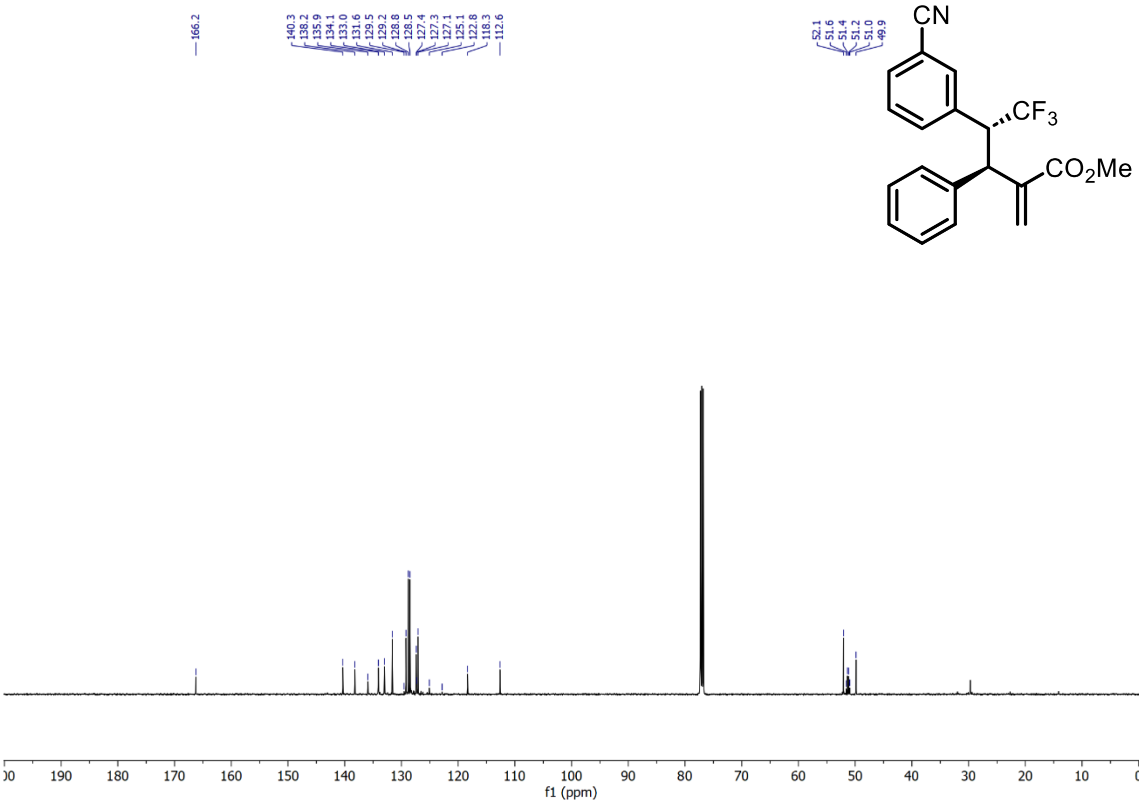

^{13}C NMR (126 MHz, $CDCl_3$) spectra of **3aa**.

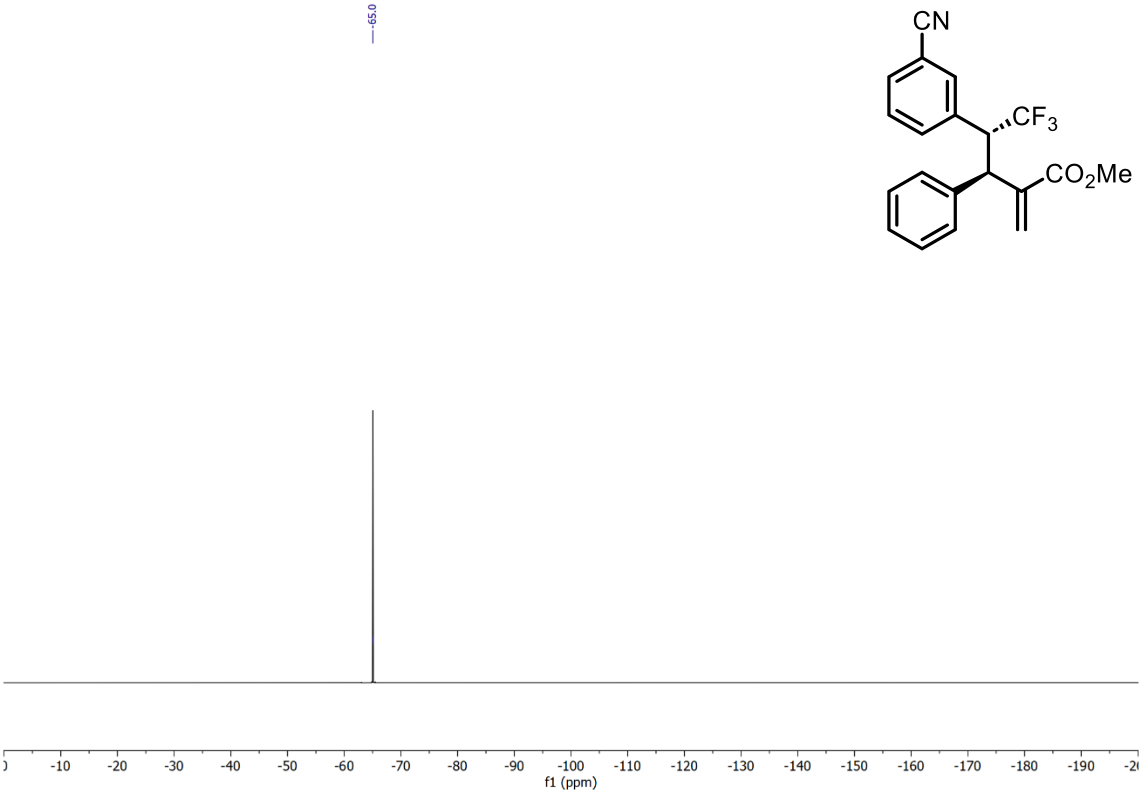

^{19}F NMR (471 MHz, $CDCl_3$) spectra of **3aa**.

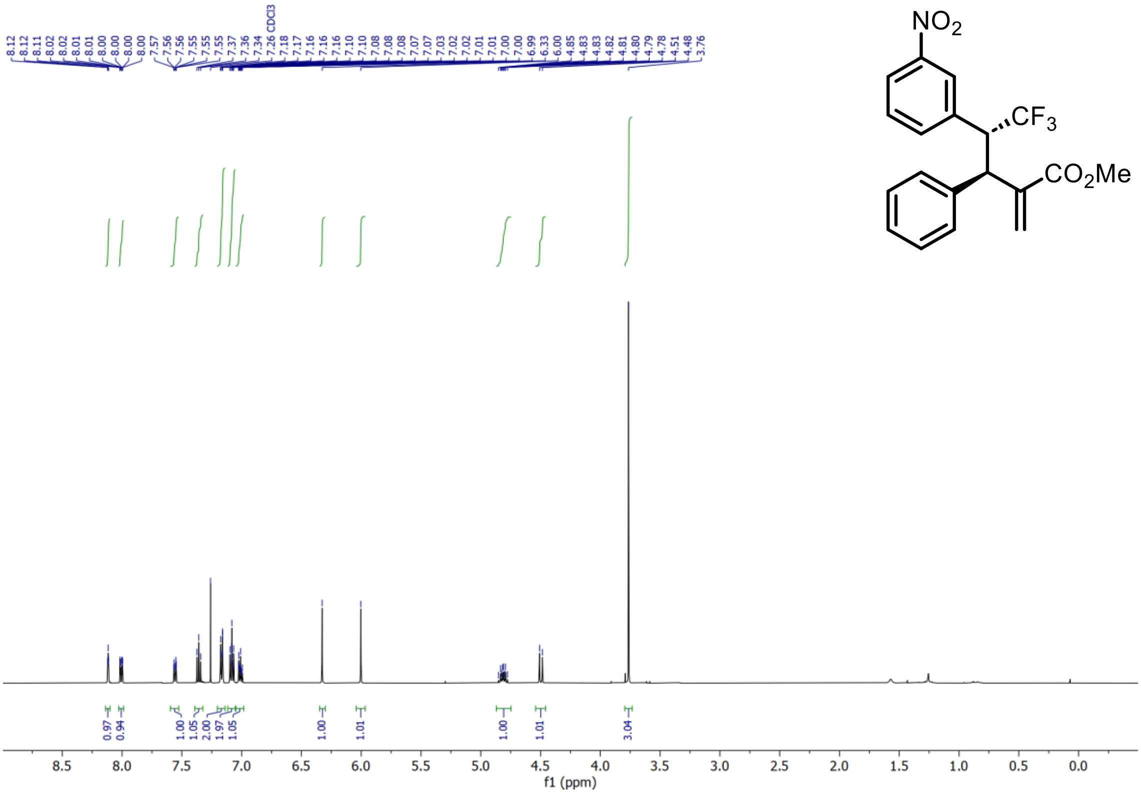

^{1}H NMR (500 MHz, $CDCl_3$) spectra of **3ab**.

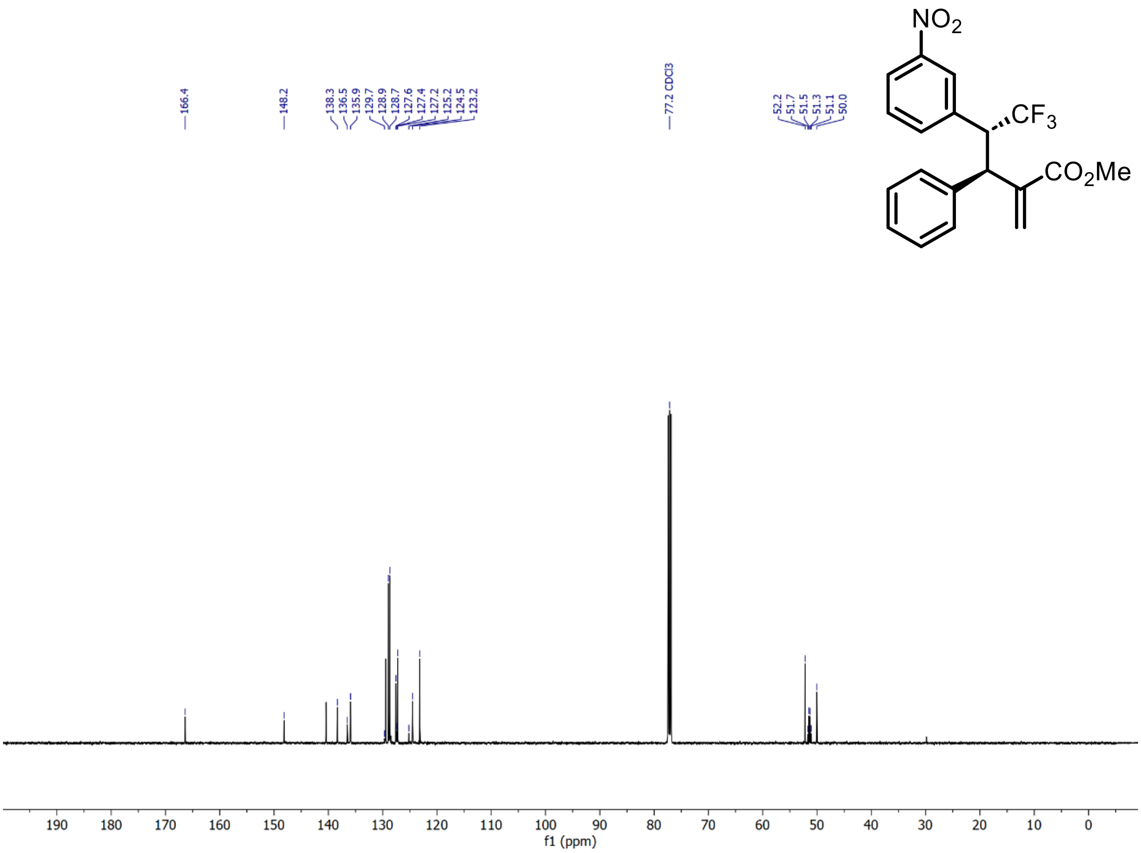

^{13}C NMR (126 MHz, $CDCl_3$) spectra of **3ab**.

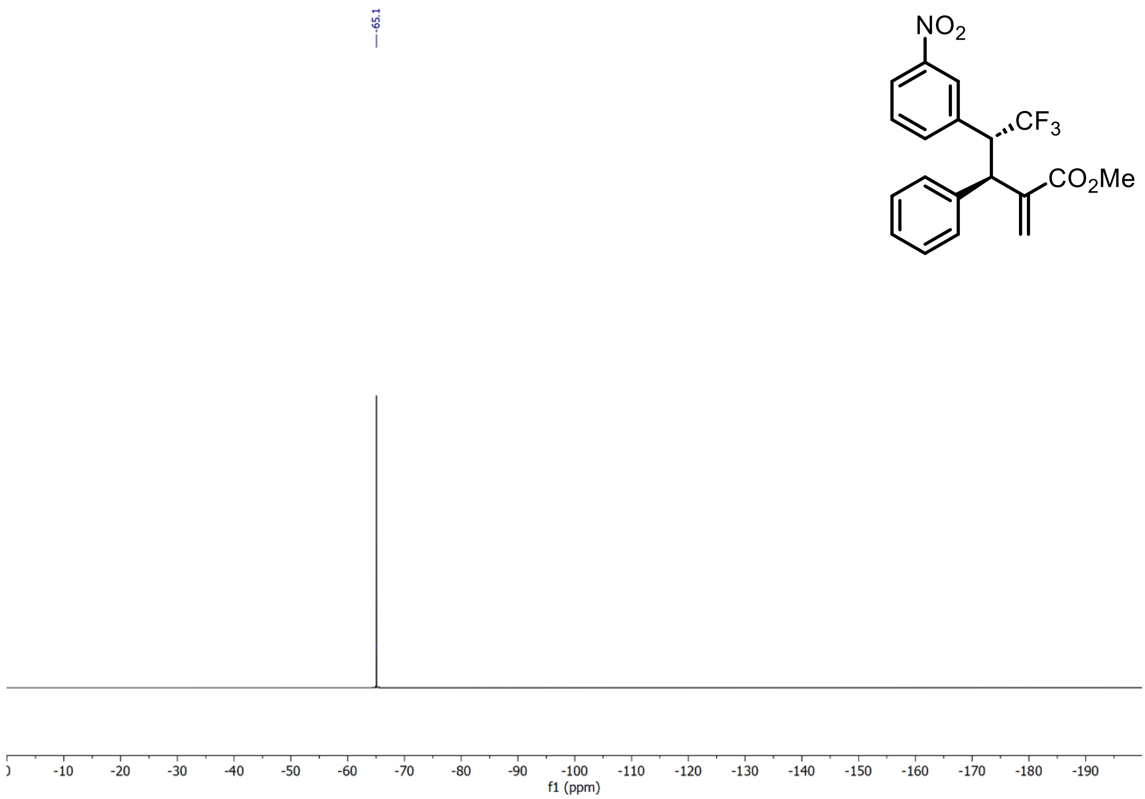

^{19}F NMR (471 MHz, $CDCl_3$) spectra of **3ab**.

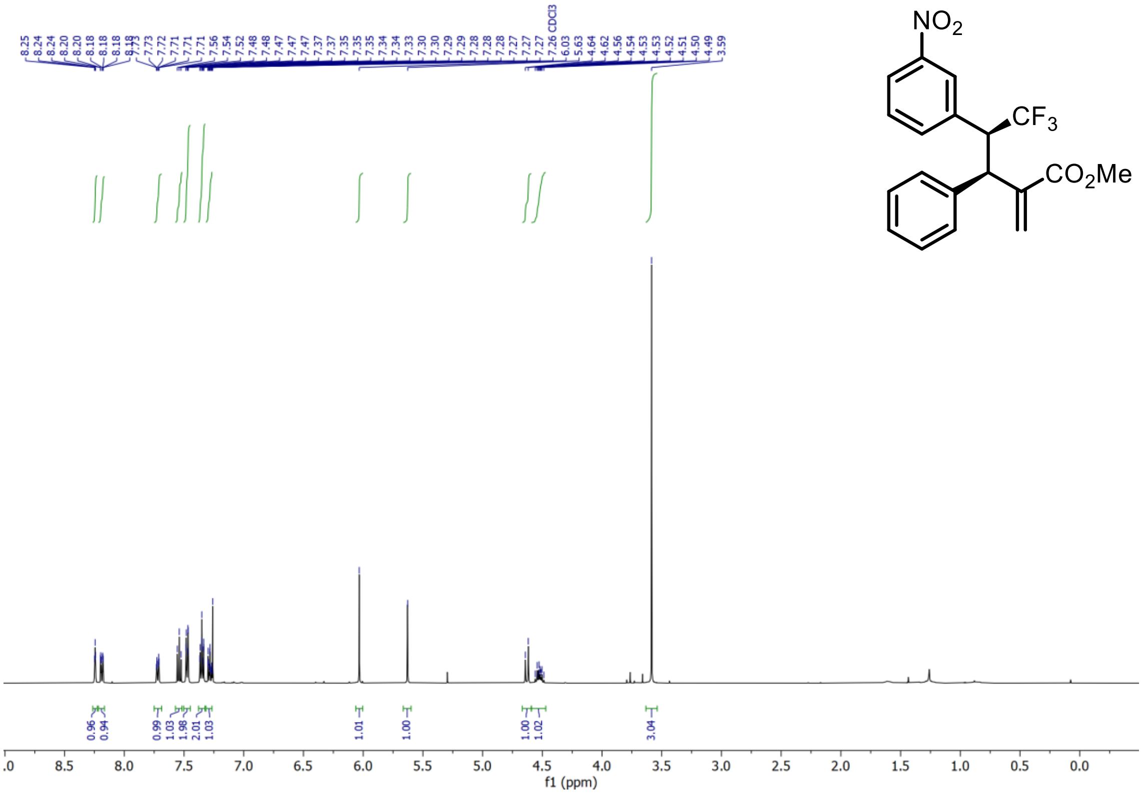

^{1}H NMR (500 MHz, CDCl3) spectra of **3ab’**.

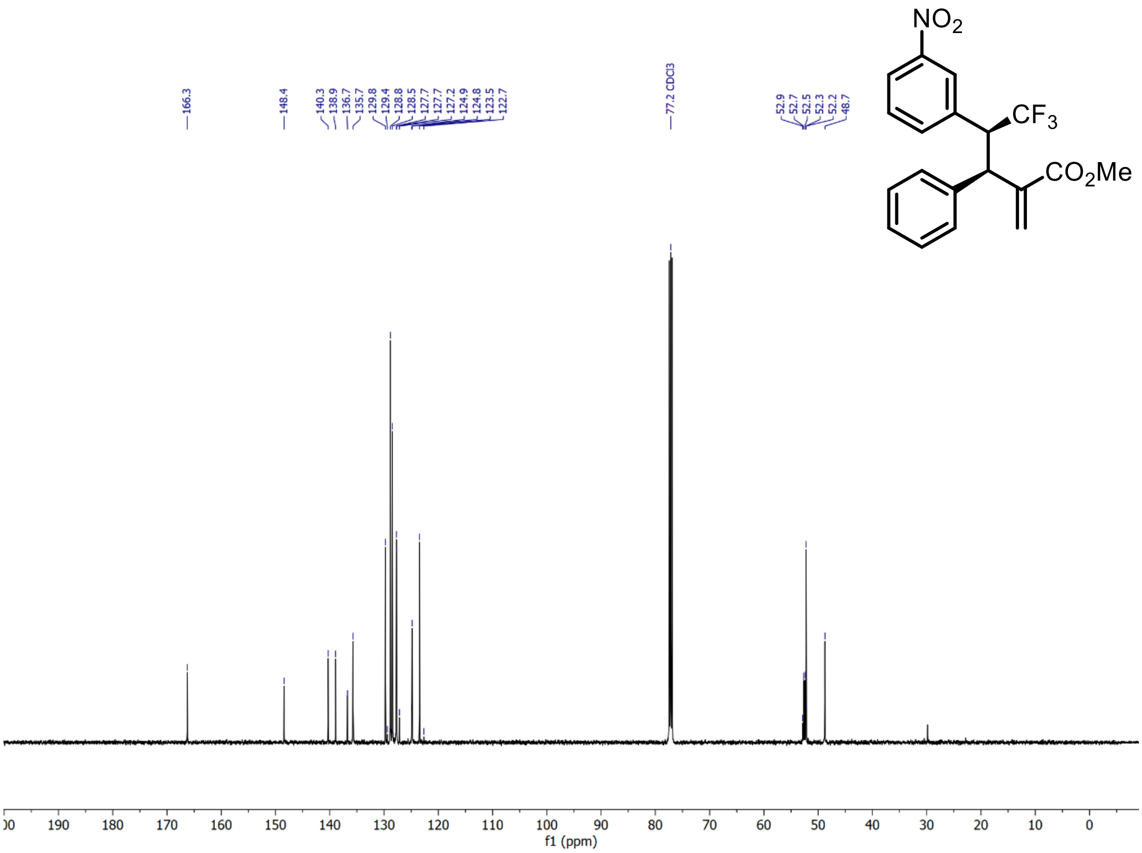

^{13}C NMR (126 MHz, CDCl3) spectra of **3ab’**.

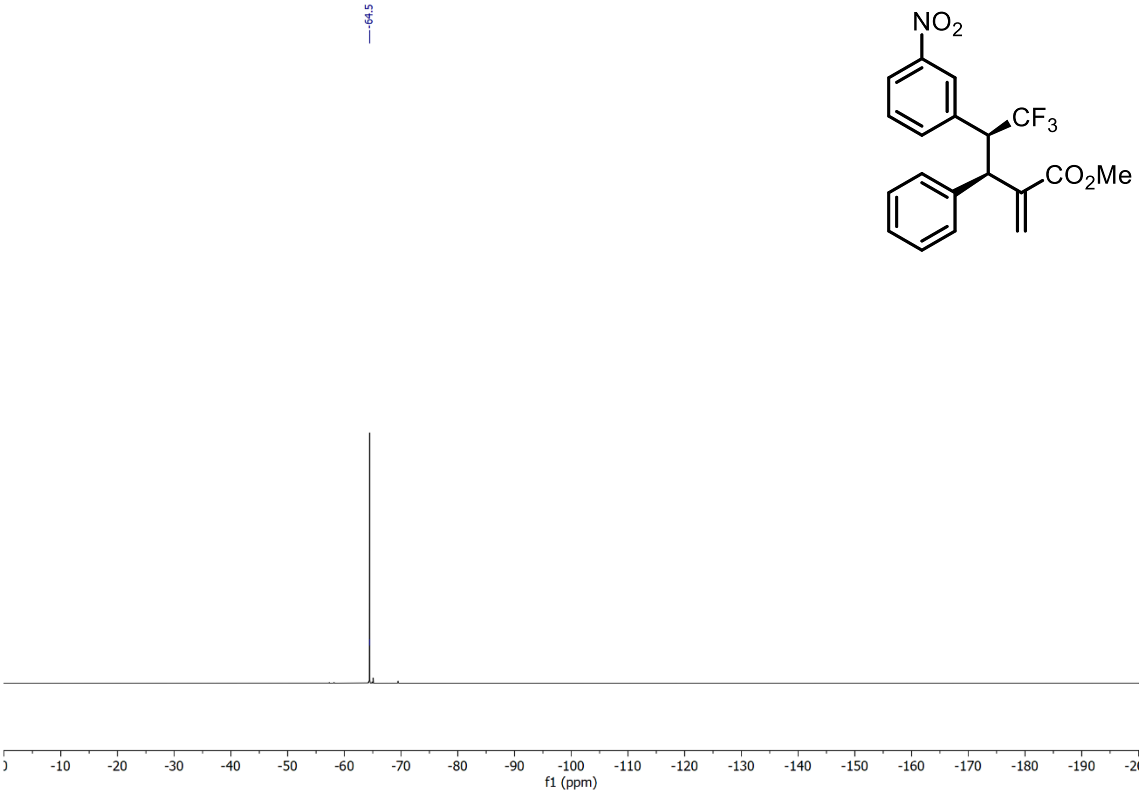

^{19}F NMR (471 MHz, $CDCl_3$) spectra of **3ab’**.

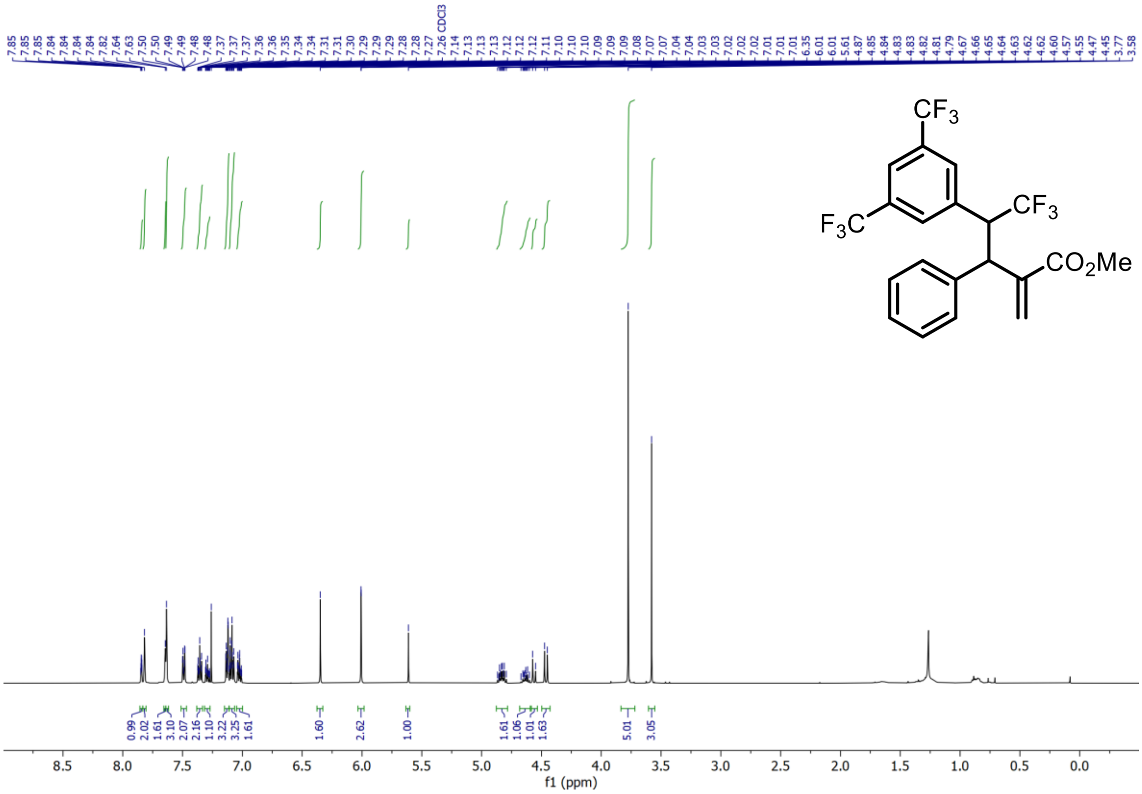

^{1}H NMR (500 MHz, $CDCl_3$) spectra of **3ac** (mixture of diastereomers).

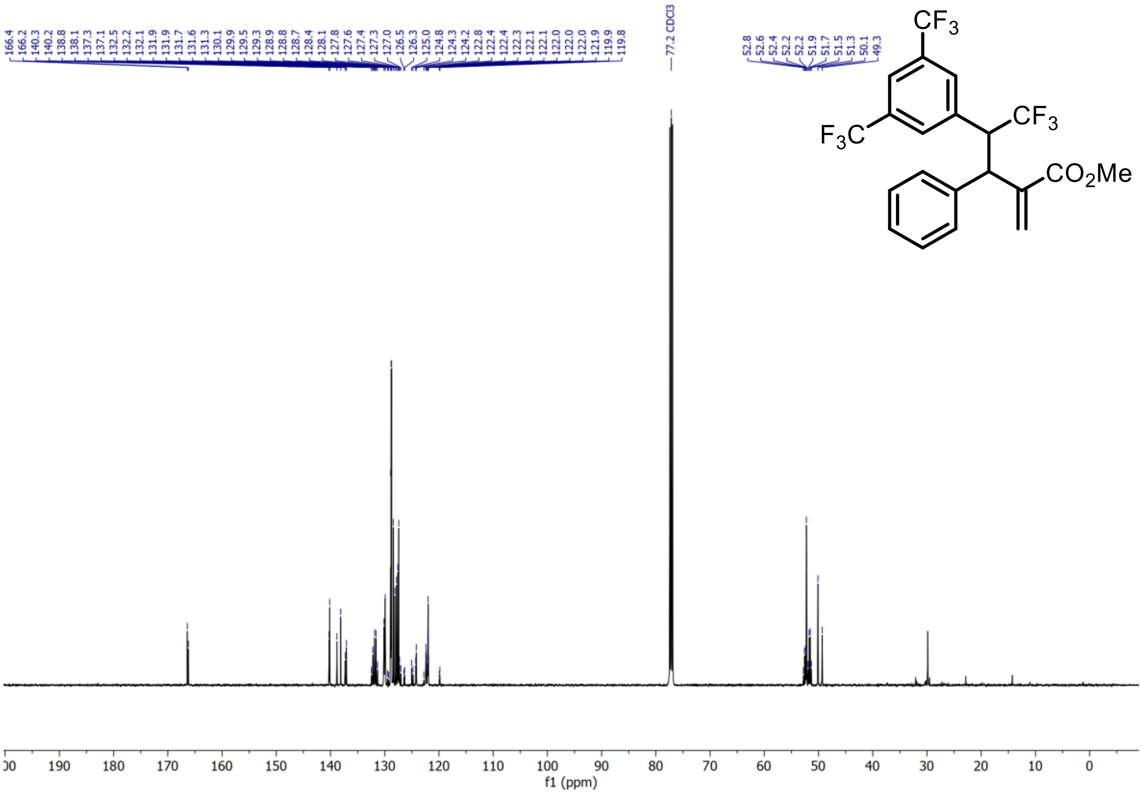

^{13}C NMR (126 MHz, $CDCl_3$) spectra of **3ac** (mixture of diastereomers).

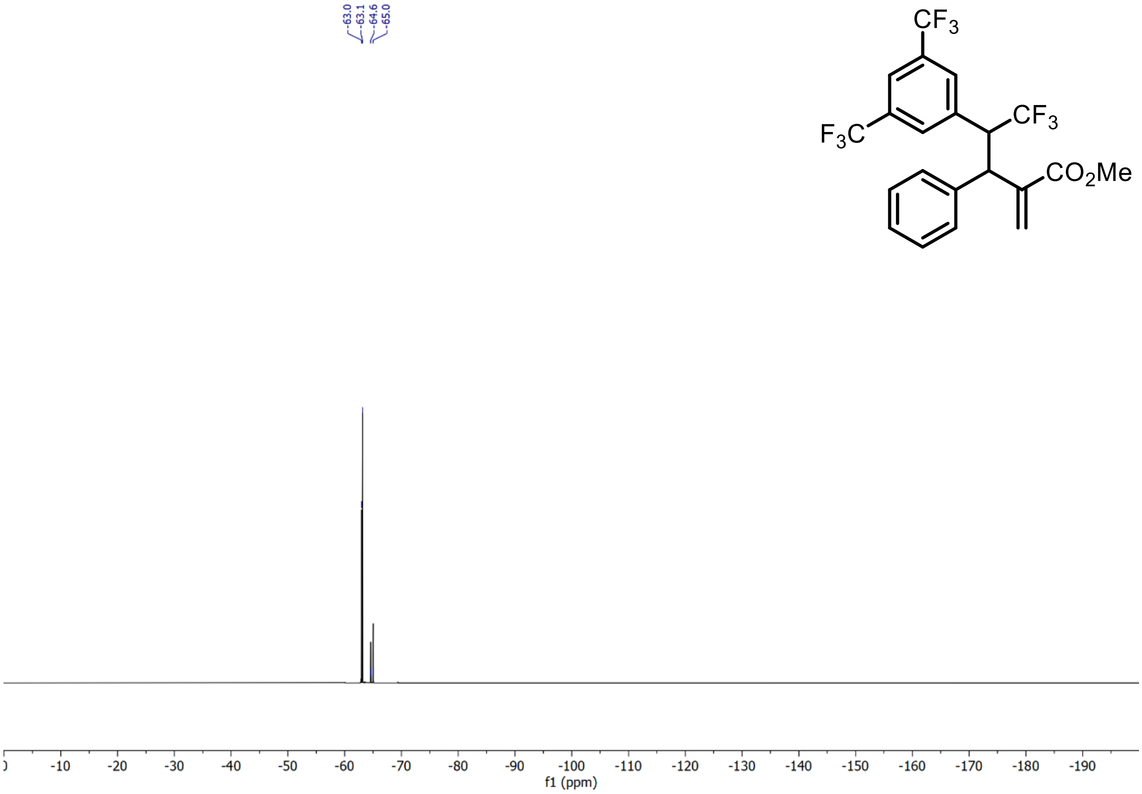

^{19}F NMR (471 MHz, $CDCl_3$) spectra of **3ac** (mixture of diastereomers).

M.3. NMR spectra of α-alkylation products 4

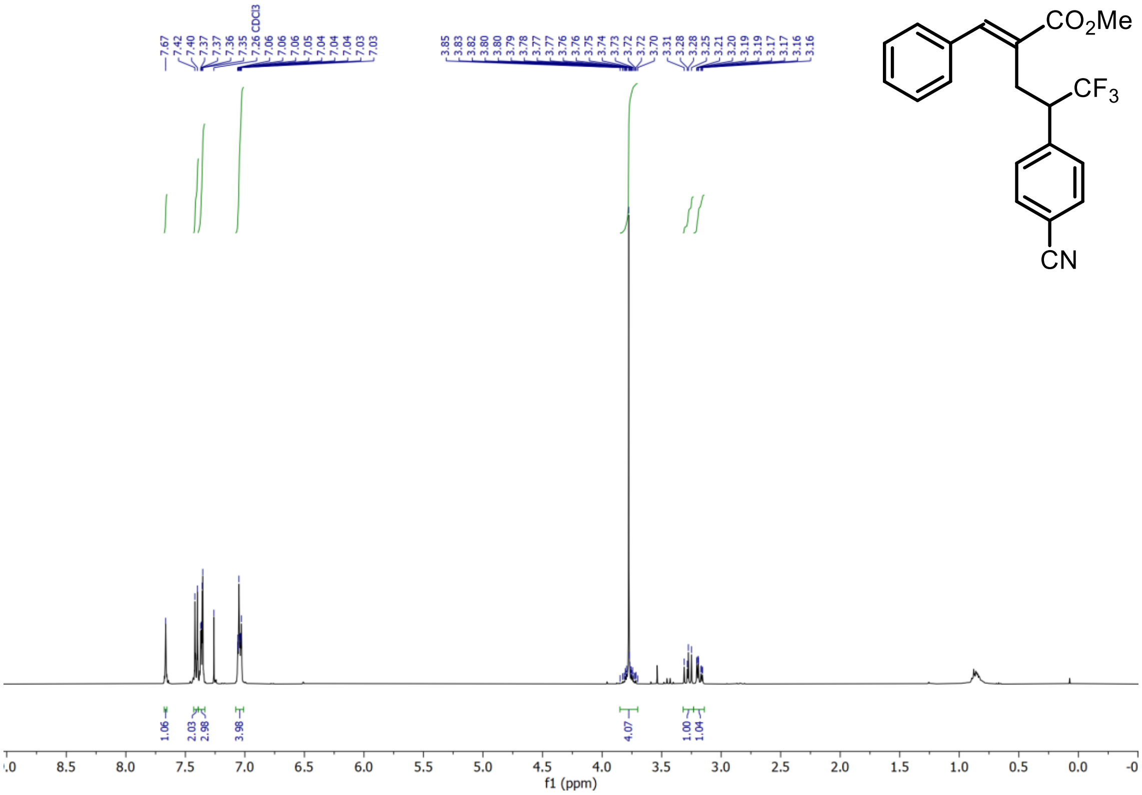

^{1}H NMR (400 MHz, $CDCl_3$) spectra of **4a**.

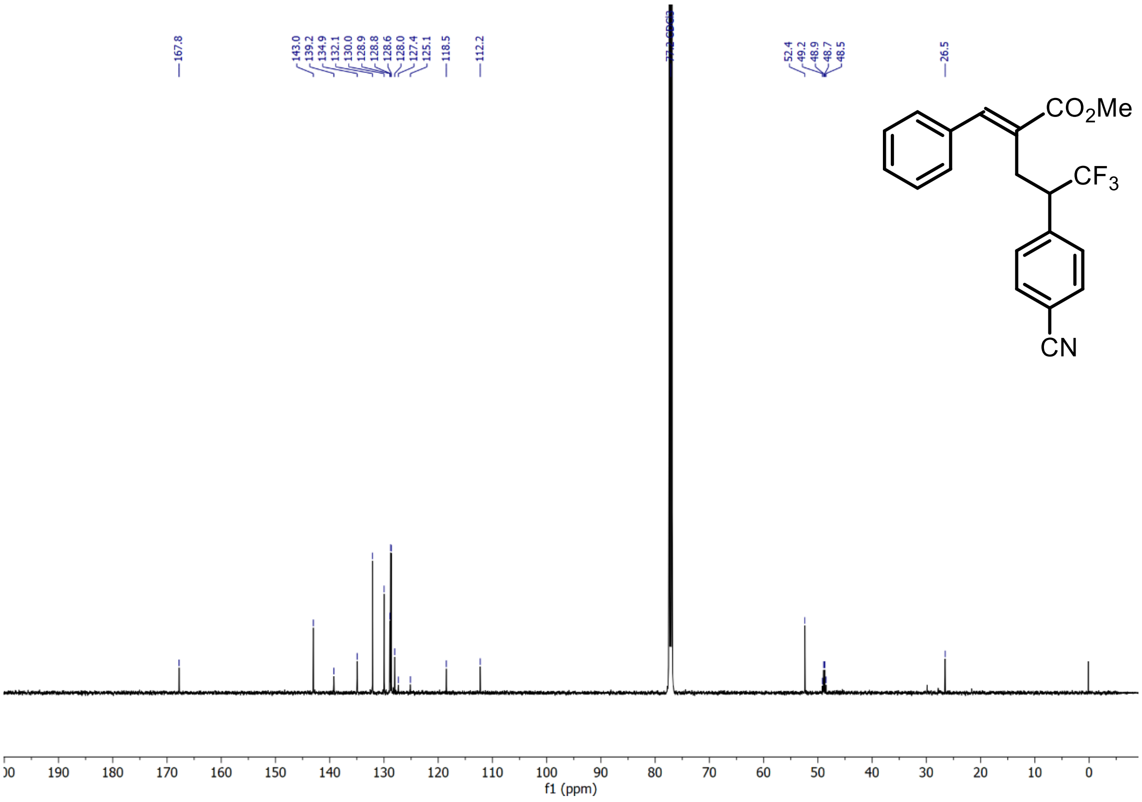

^{13}C NMR (126 MHz, $CDCl_3$) spectra of **4a**.

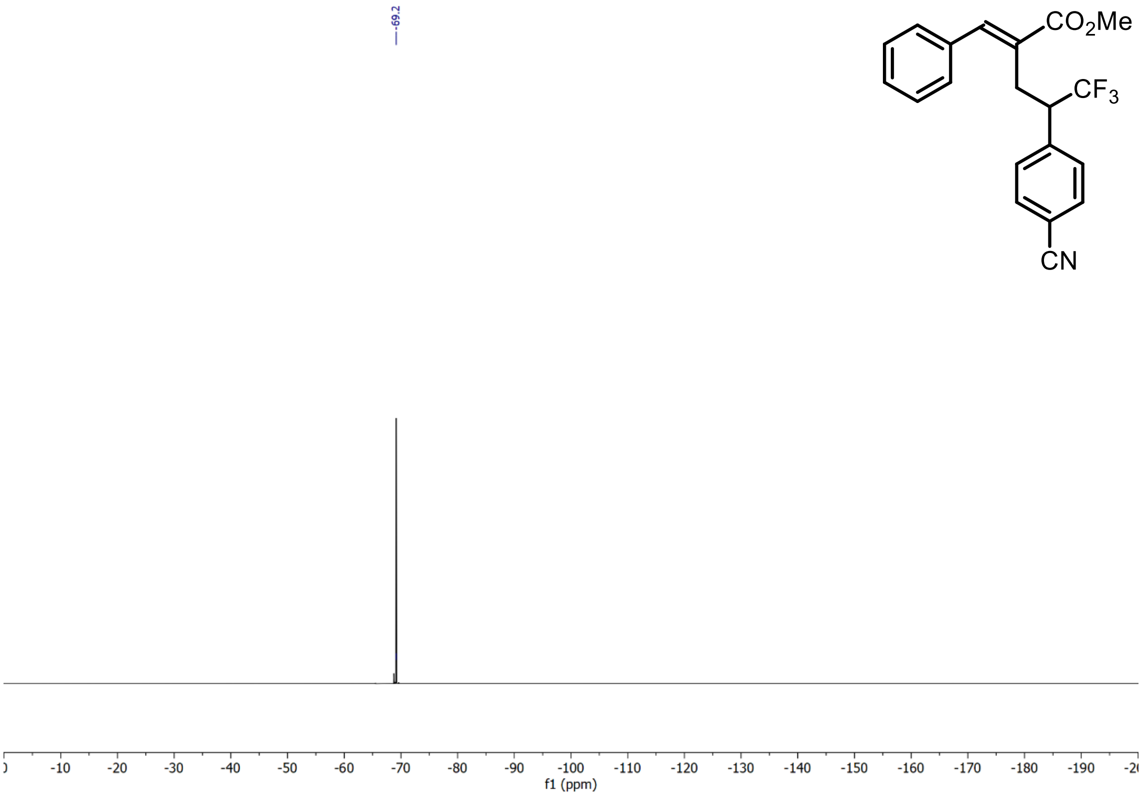

^{19}F NMR (471 MHz, $CDCl_3$) spectra of **4a**.

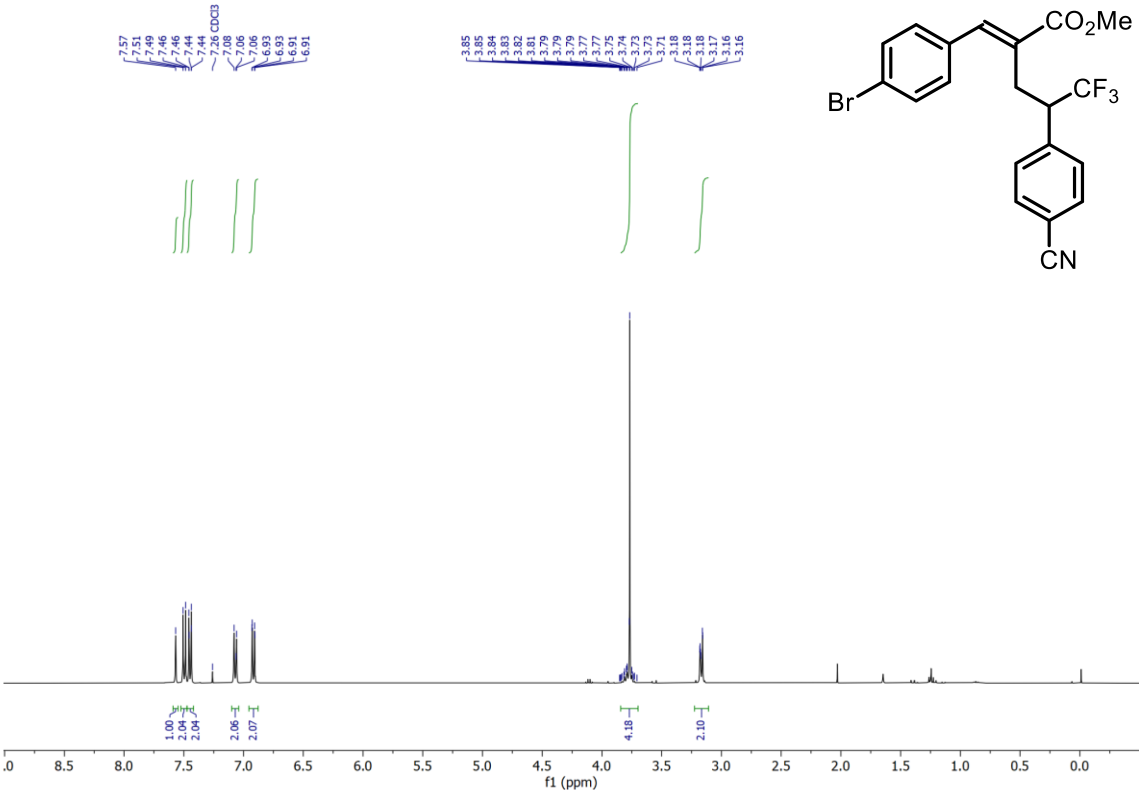

^{1}H NMR (400 MHz, $CDCl_3$) spectra of **4b**.

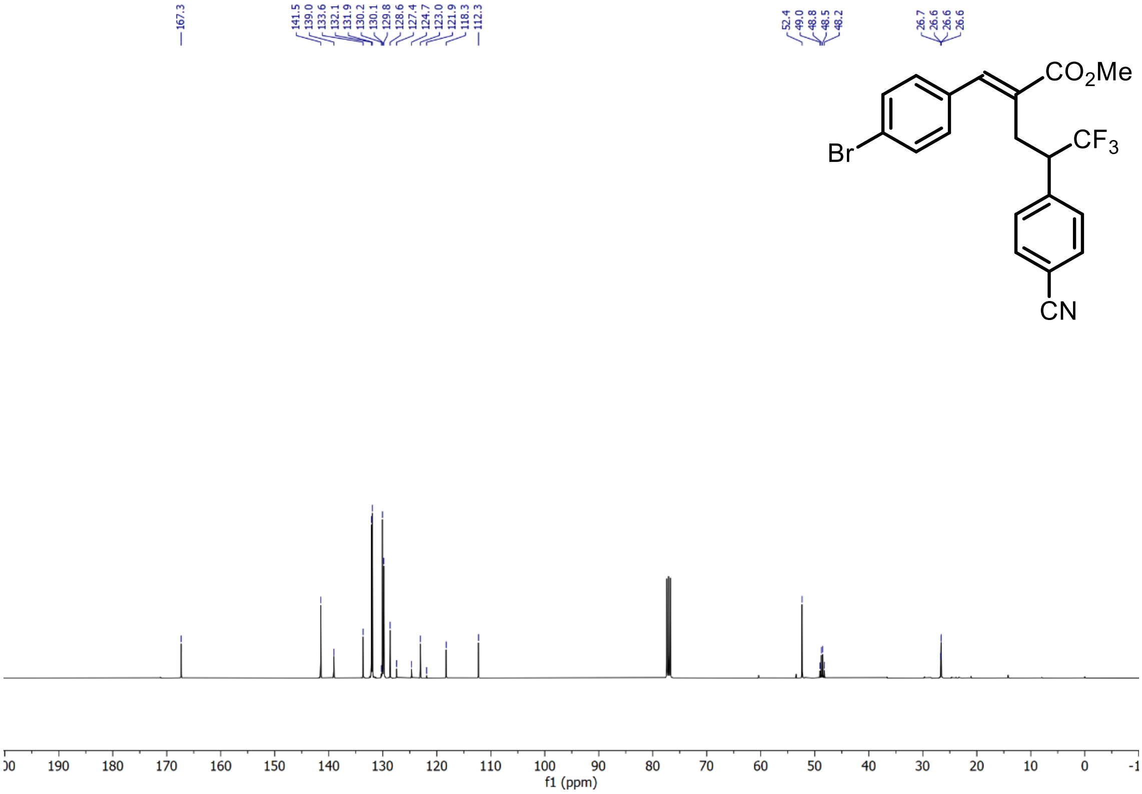

^{13}C NMR (101 MHz, $CDCl_3$) spectra of **4b**.

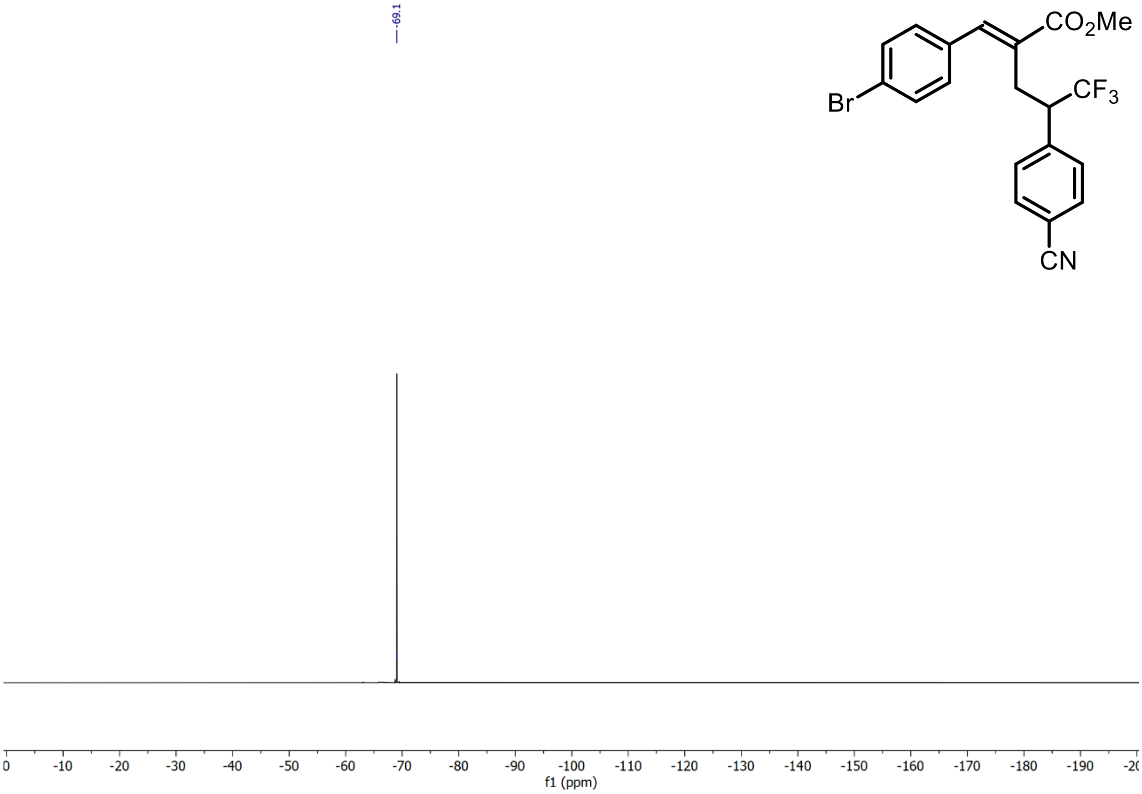

^{19}F NMR (471 MHz, $CDCl_3$) spectra of **4b**.

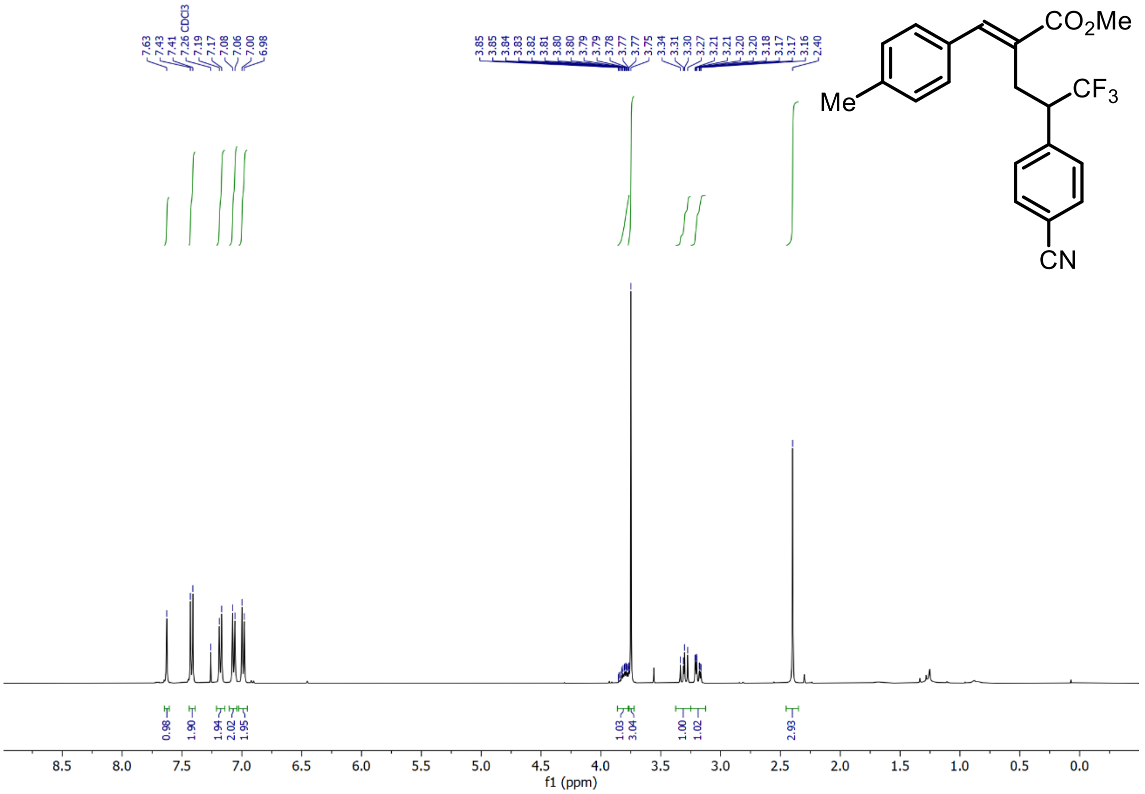

^{1}H NMR (400 MHz, $CDCl_3$) spectra of **4c**.

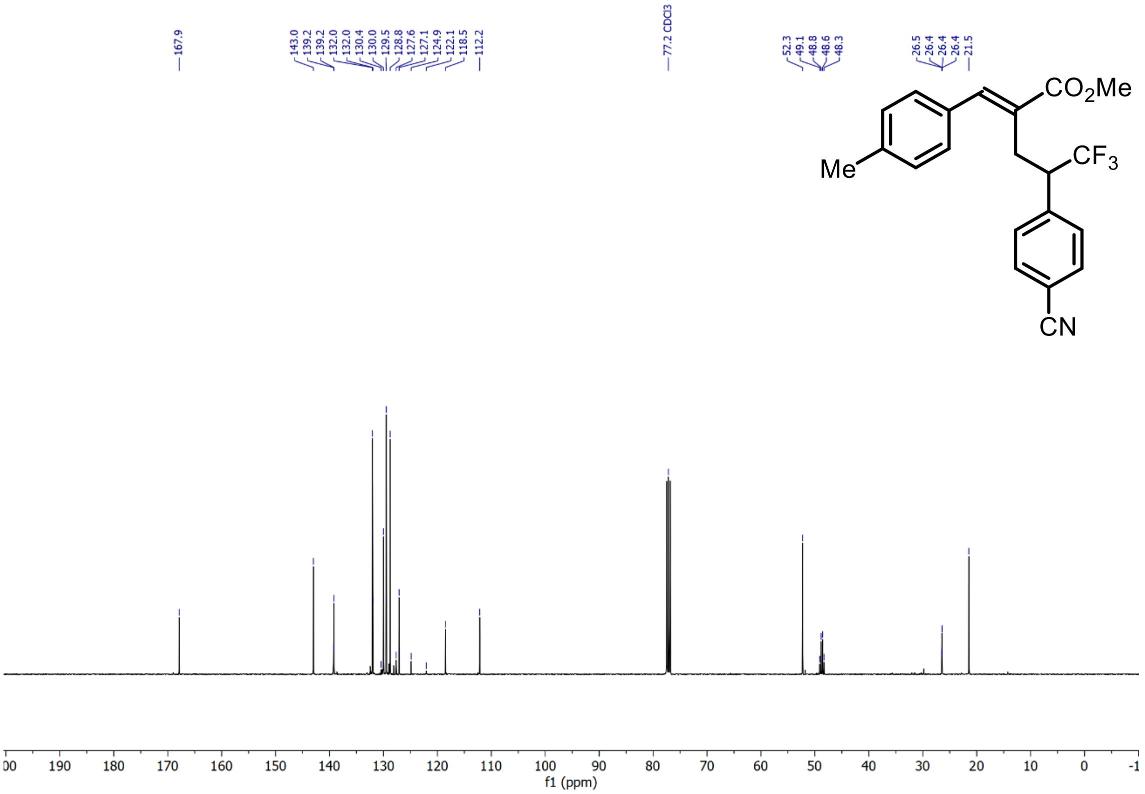

^{13}C NMR (101 MHz, $CDCl_3$) spectra of **4c**.

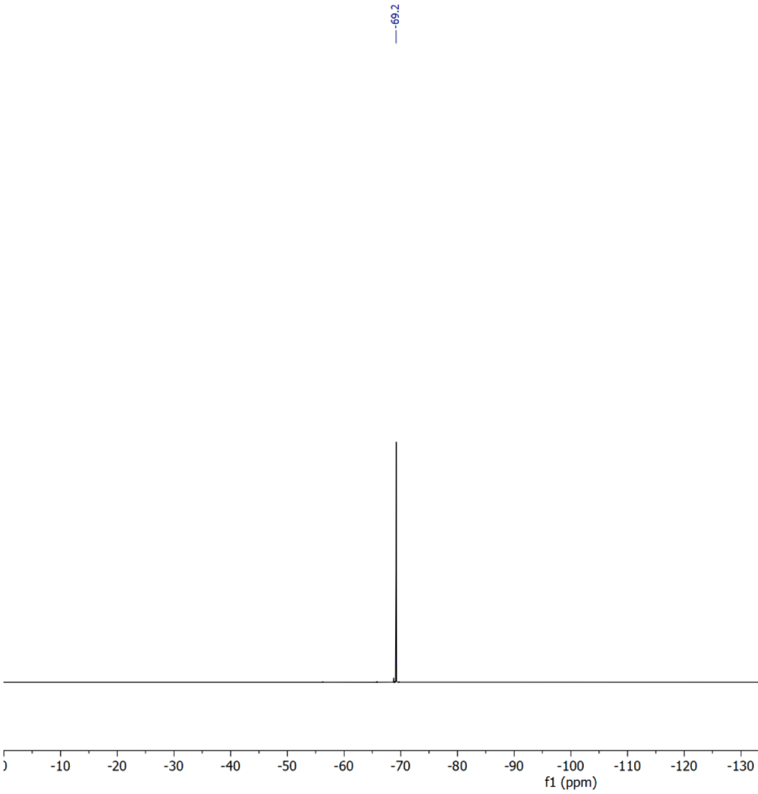

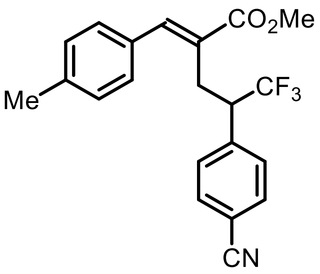

^{19}F NMR (376 MHz, $CDCl_3$) spectra of **4c**.

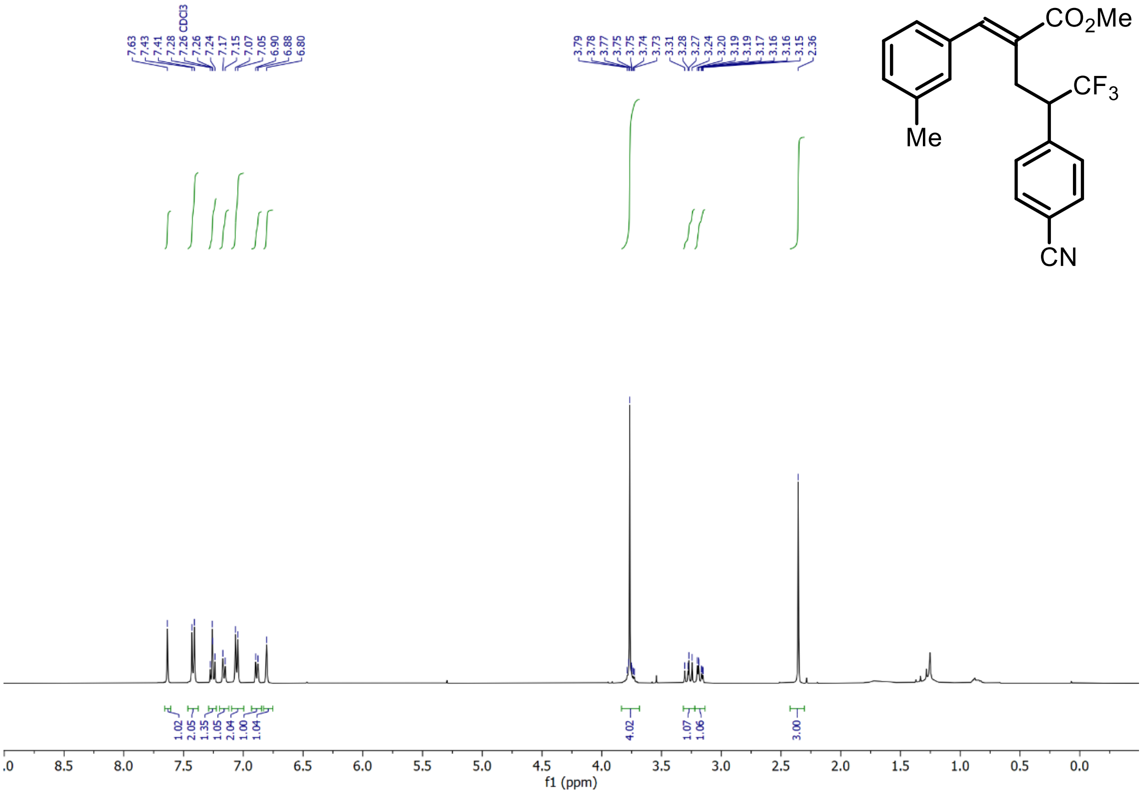

^{1}H NMR (400 MHz, $CDCl_3$) spectra of **4d**.

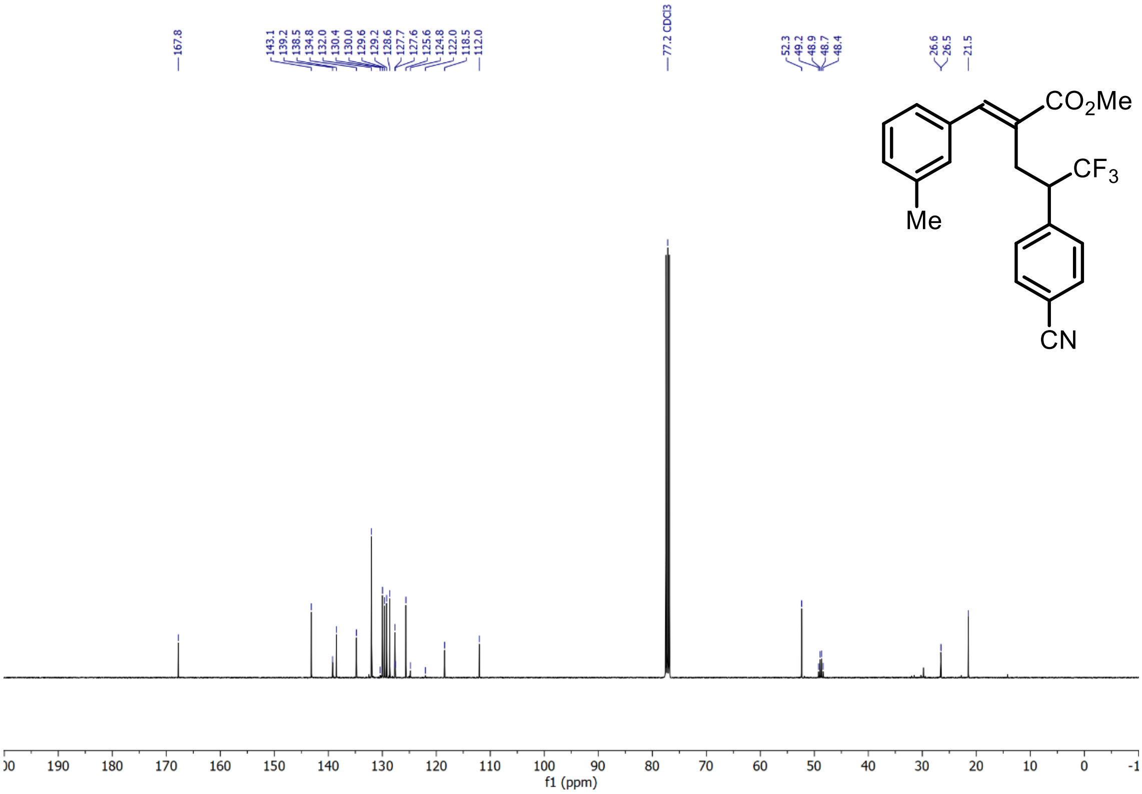

^{13}C NMR (101 MHz, $CDCl_3$) spectra of **4d**.

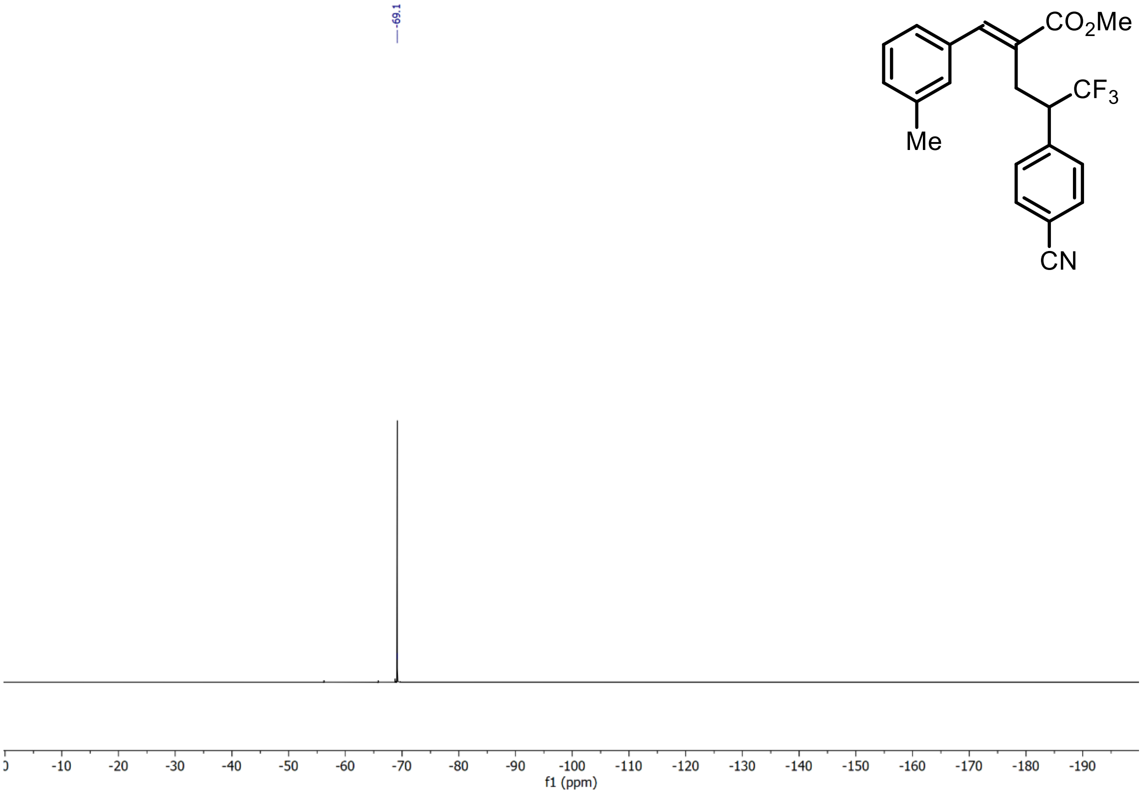

^{19}F NMR (376 MHz, $CDCl_3$) spectra of **4d**.

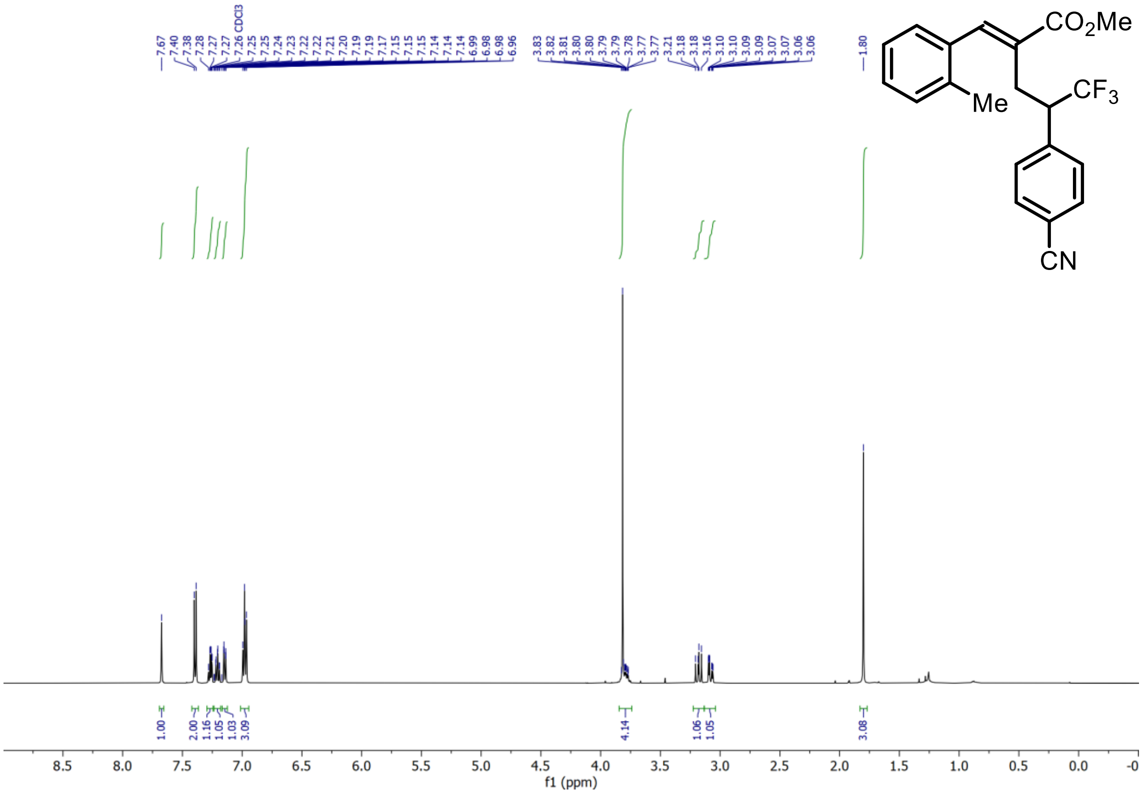

^{1}H NMR (500 MHz, $CDCl_3$) spectra of **4e**.

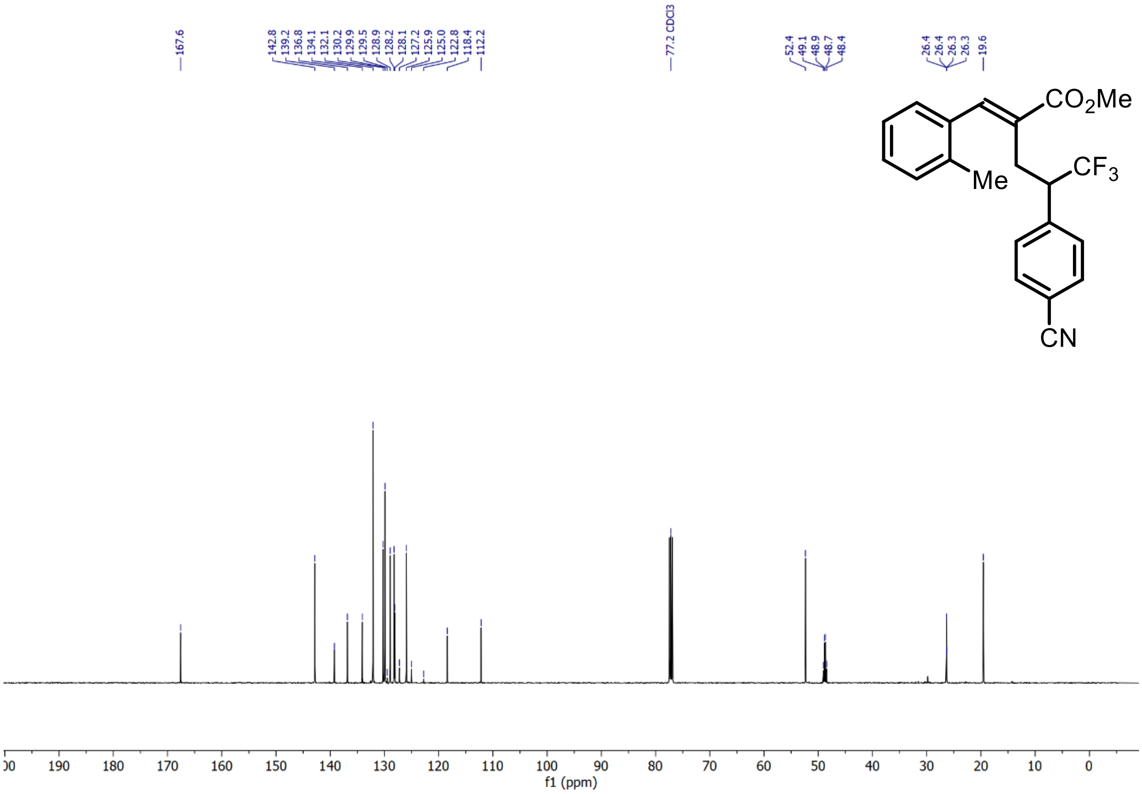

^{13}C NMR (126 MHz, $CDCl_3$) spectra of **4e**.

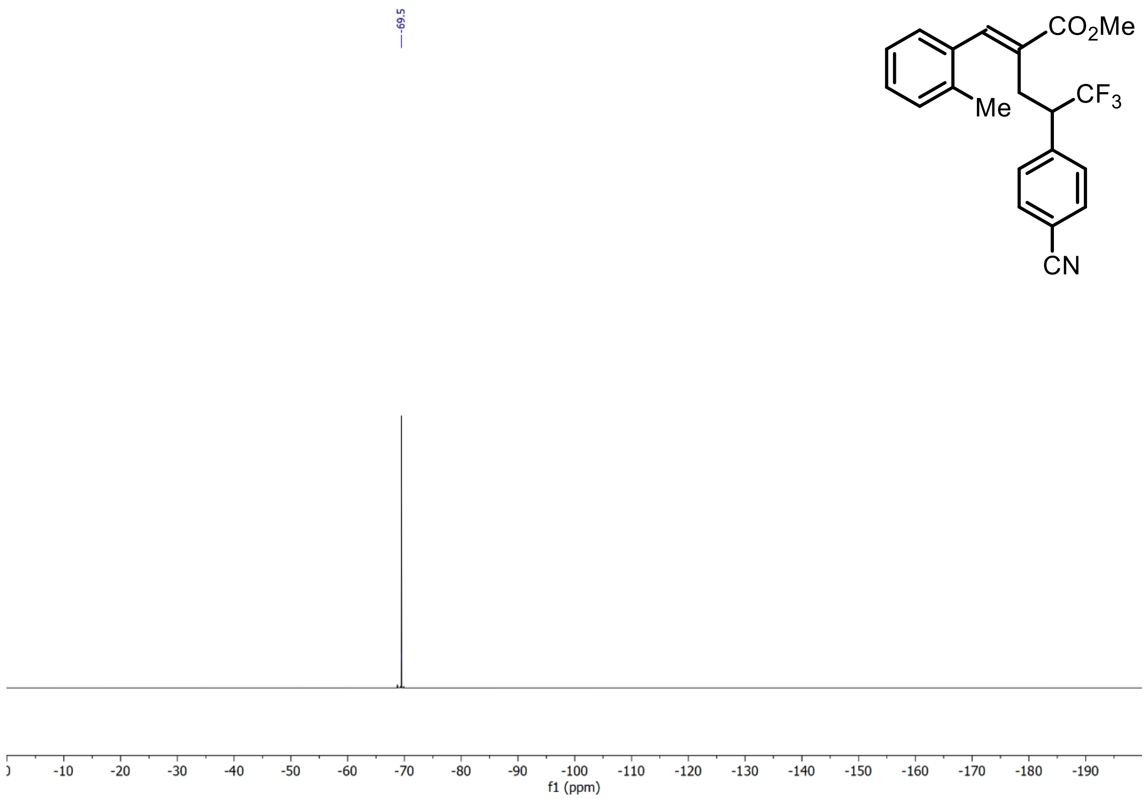

^{19}F NMR (471 MHz, $CDCl_3$) spectra of **4e**.

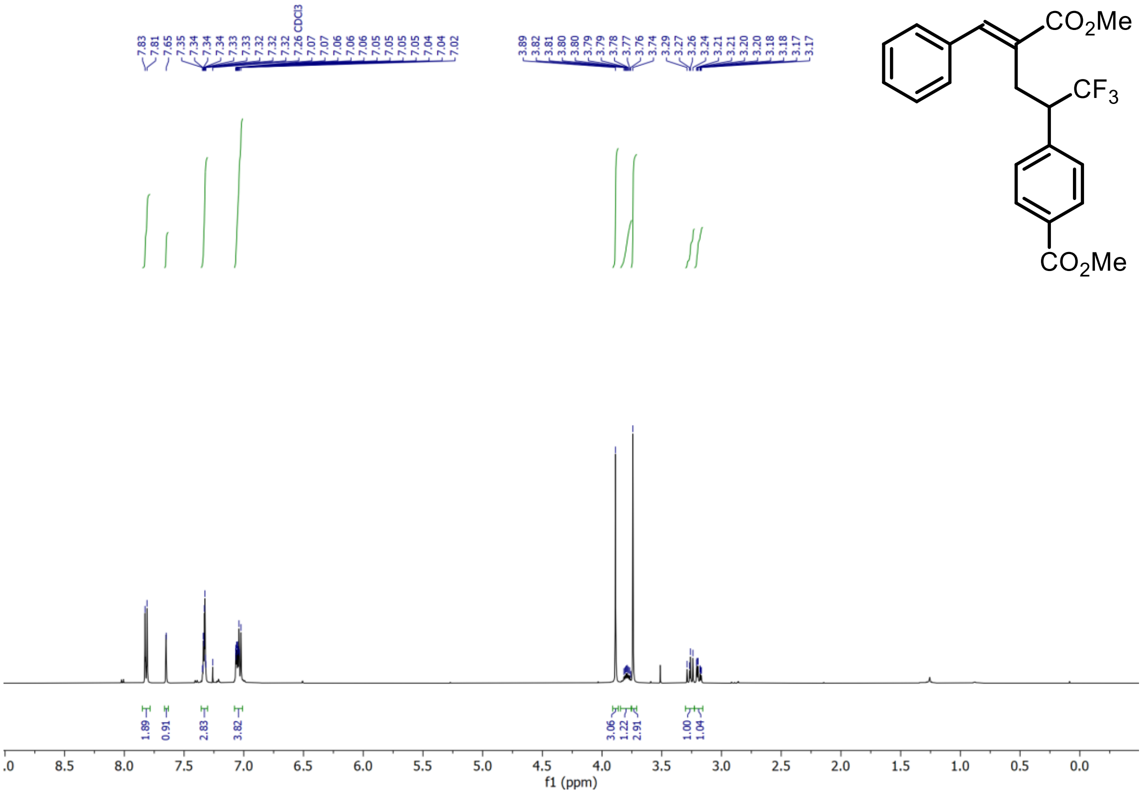

^{1}H NMR (500 MHz, CDCl₃) spectra of **4f**.

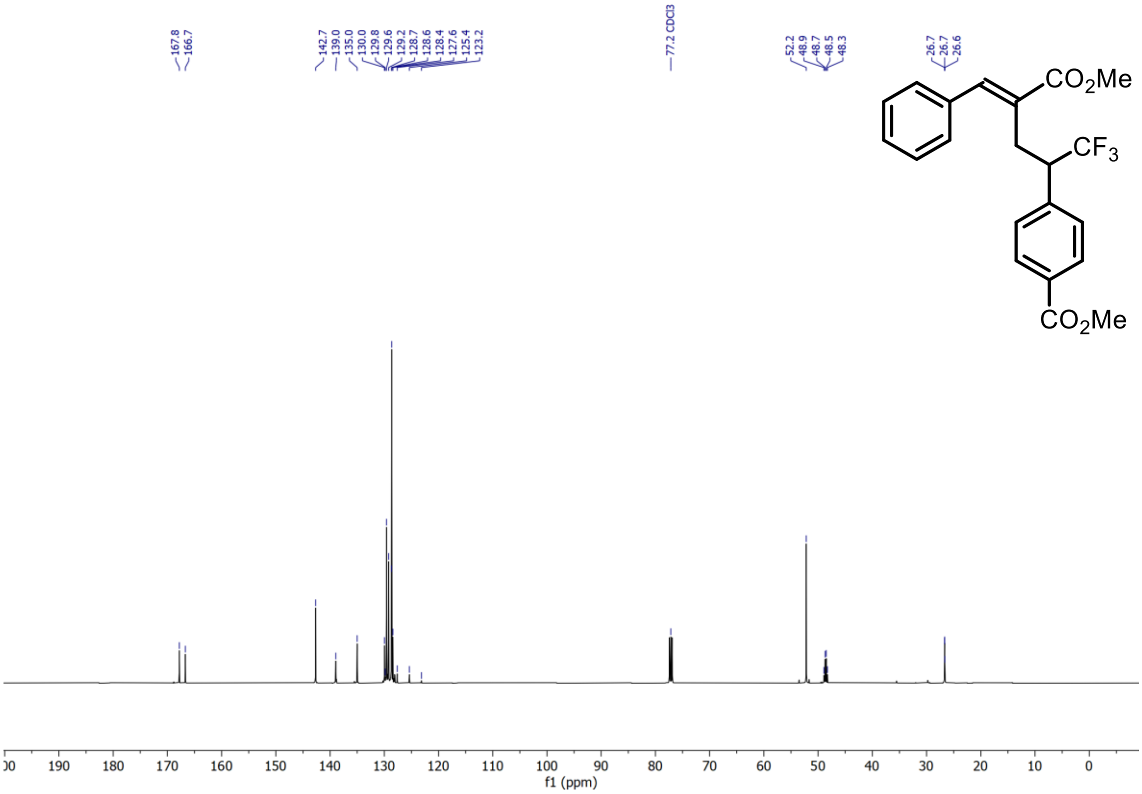

^{13}C NMR (126 MHz, CDCl₃) spectra of **4f**.

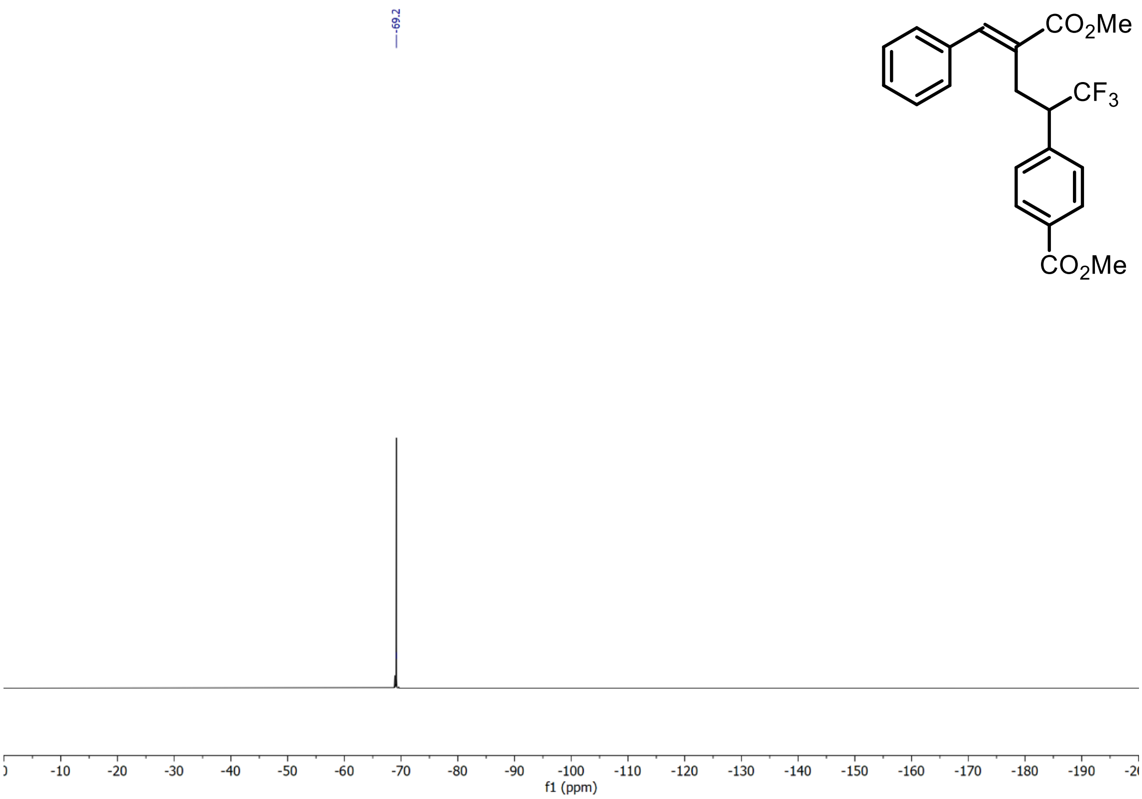

^{19}F NMR (471 MHz, $CDCl_3$) spectra of **4f**.

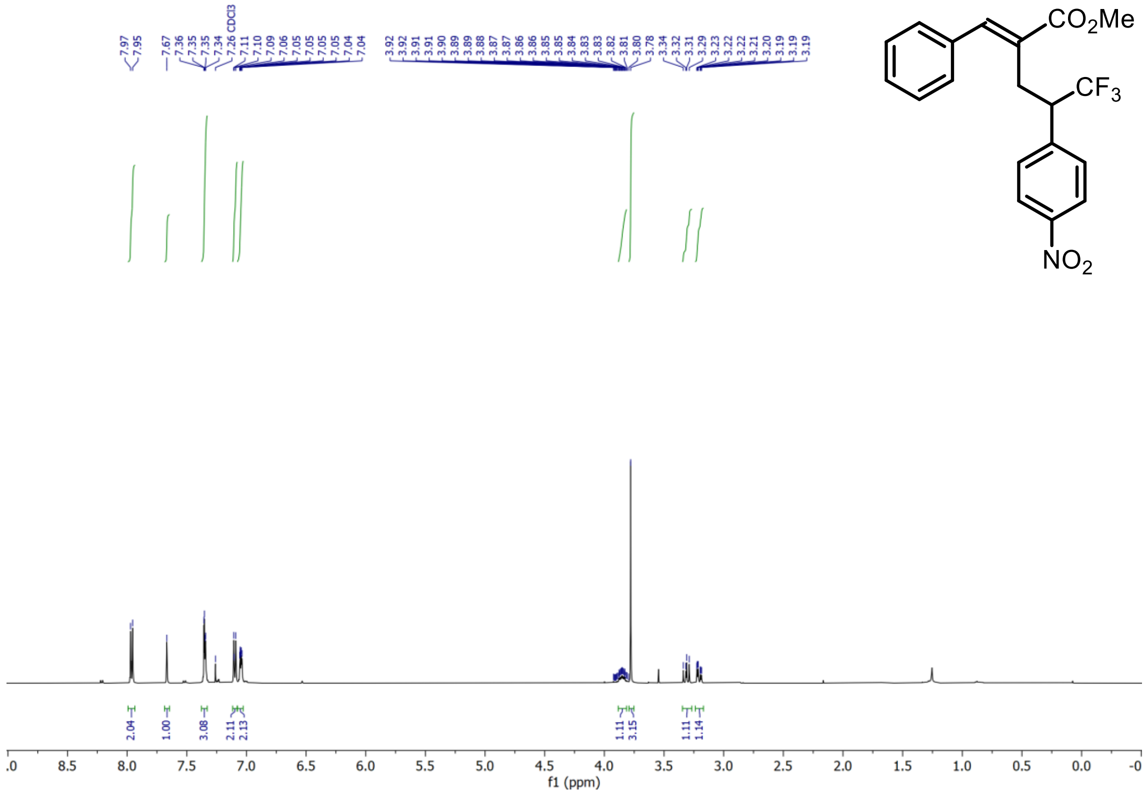

^{1}H NMR (500 MHz, $CDCl_3$) spectra of **4g**.

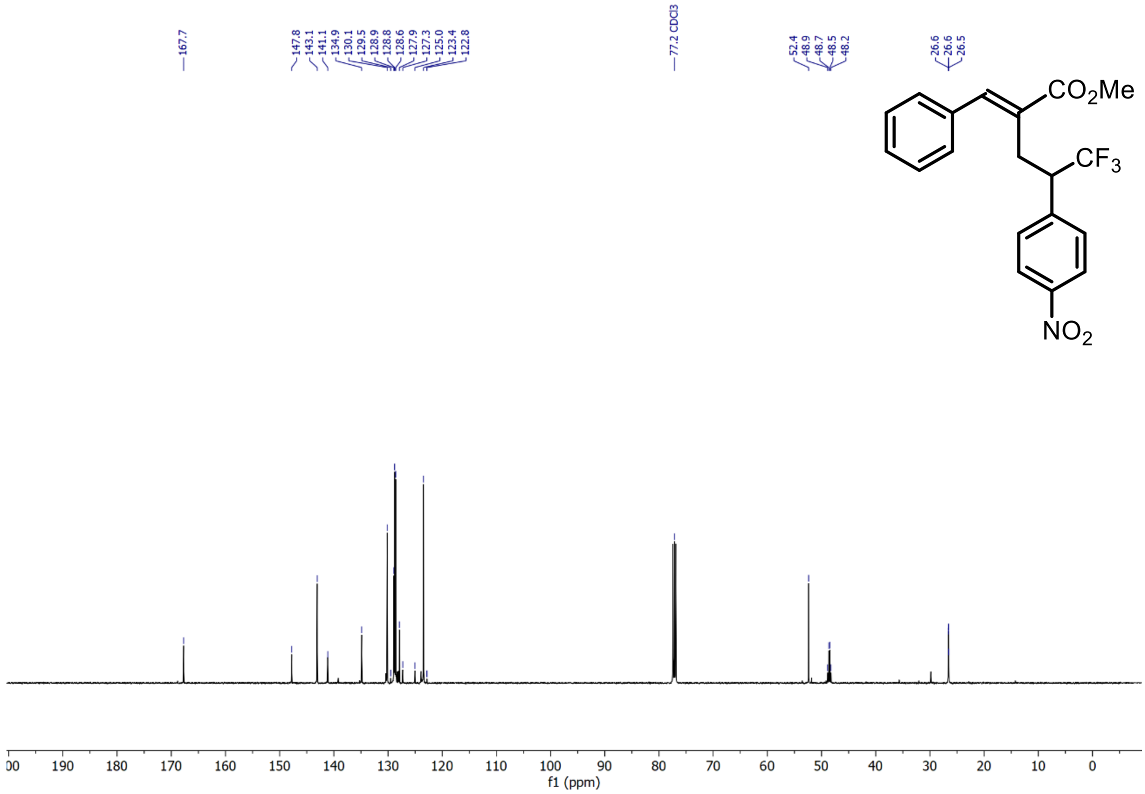

^{13}C NMR (126 MHz, $CDCl_3$) spectra of **4g**.

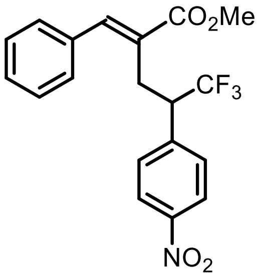

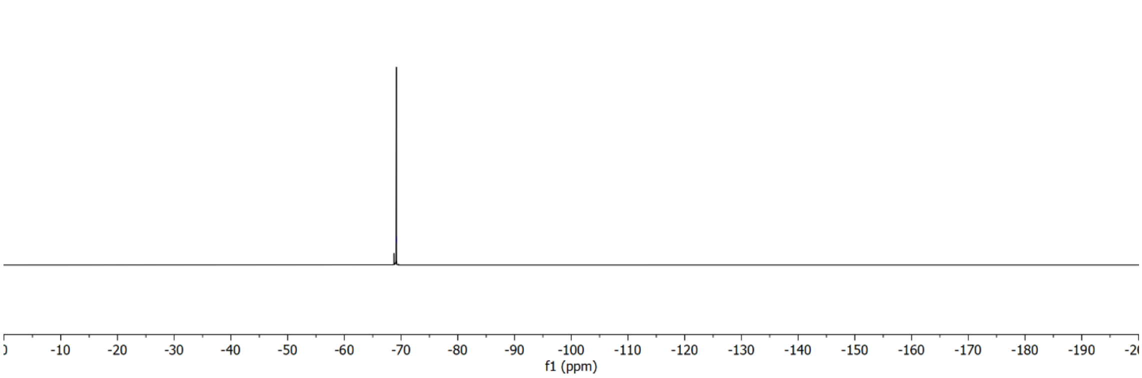

^{19}F NMR (471 MHz, $CDCl_3$) spectra of **4g**.

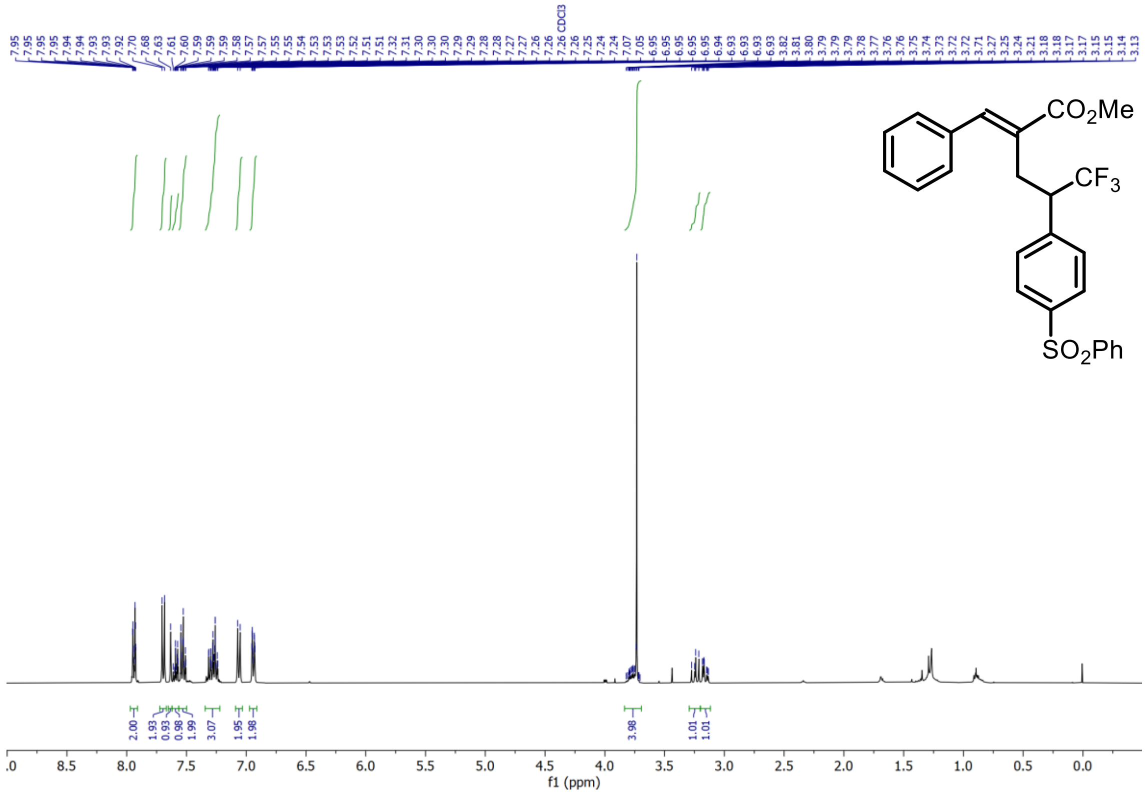

^{1}H NMR (400 MHz, $CDCl_3$) spectra of **4h**.

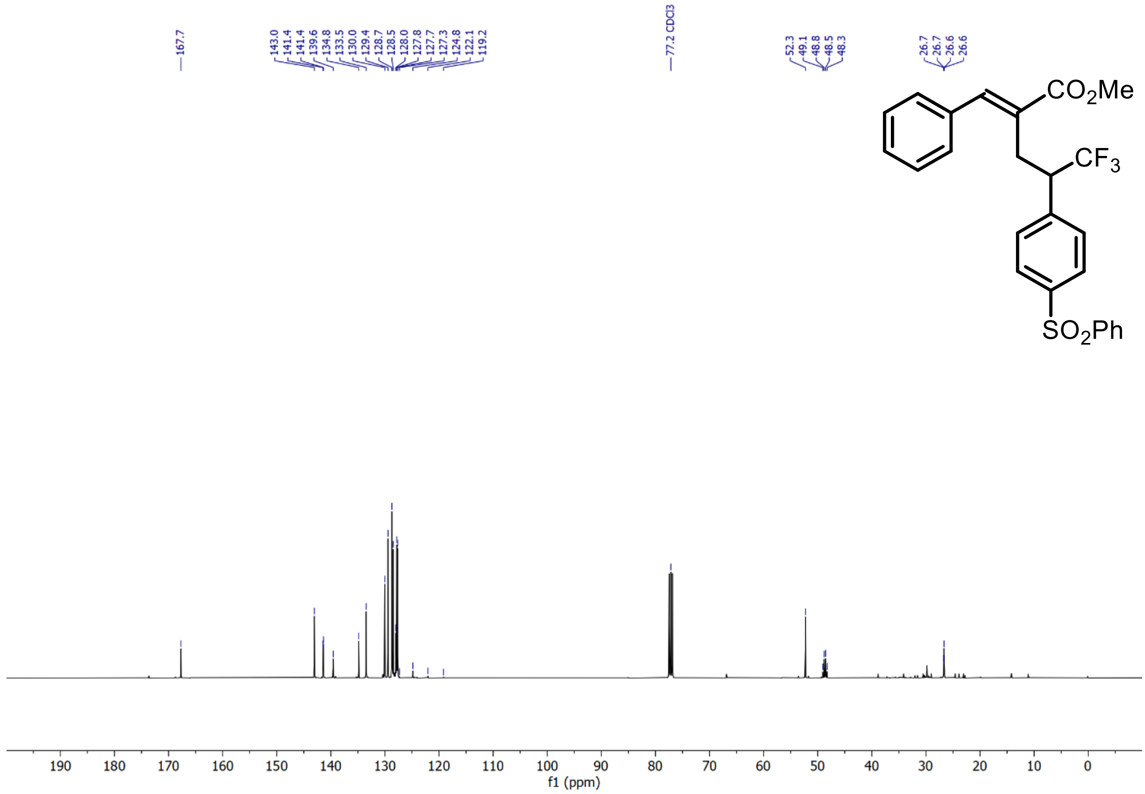

^{13}C NMR (101 MHz, $CDCl_3$) spectra of **4h**.

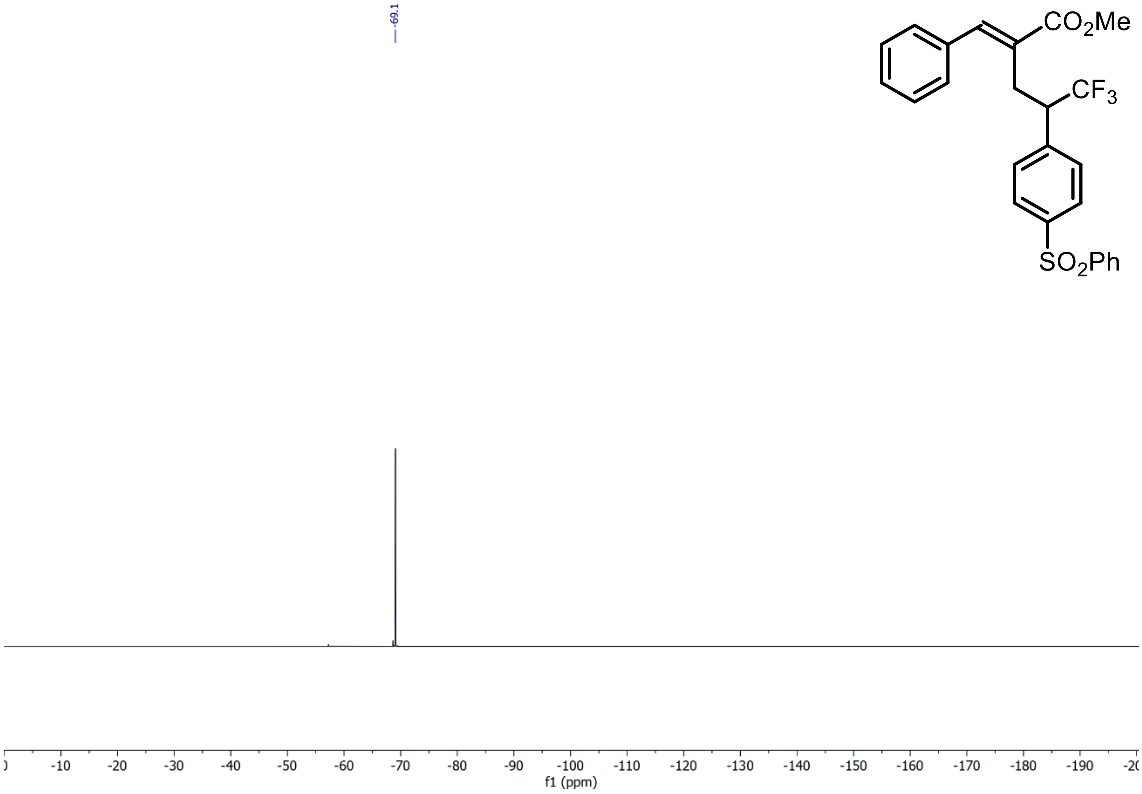

^{19}F NMR (471 MHz, $CDCl_3$) spectra of **4h**.

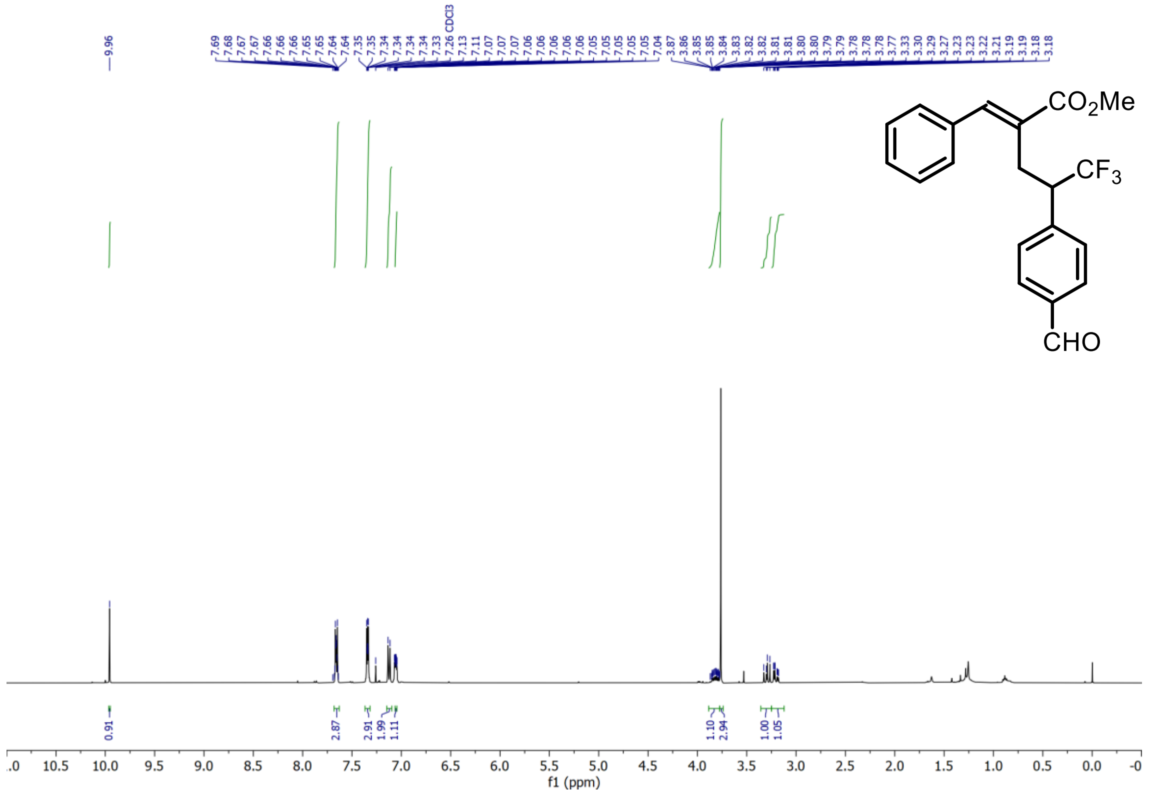

^{1}H NMR (400 MHz, $CDCl_3$) spectra of **4i**.

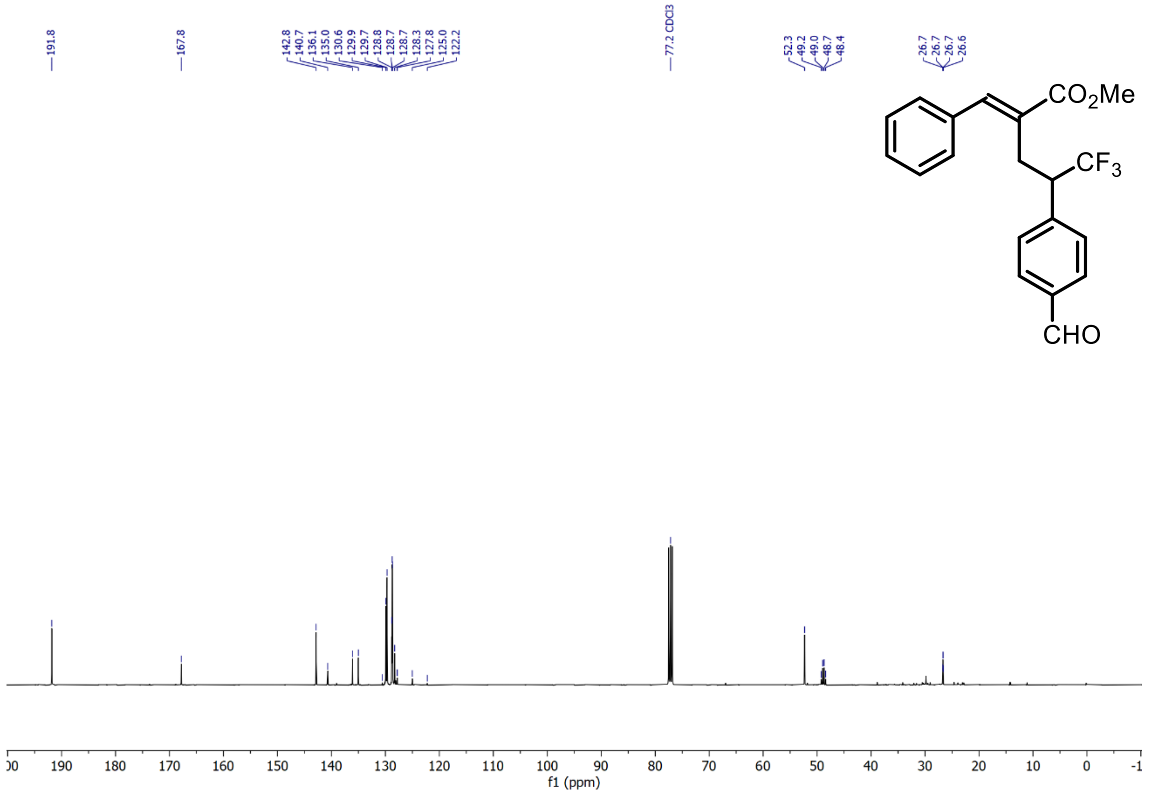

^{13}C NMR (101 MHz, $CDCl_3$) spectra of **4i**.

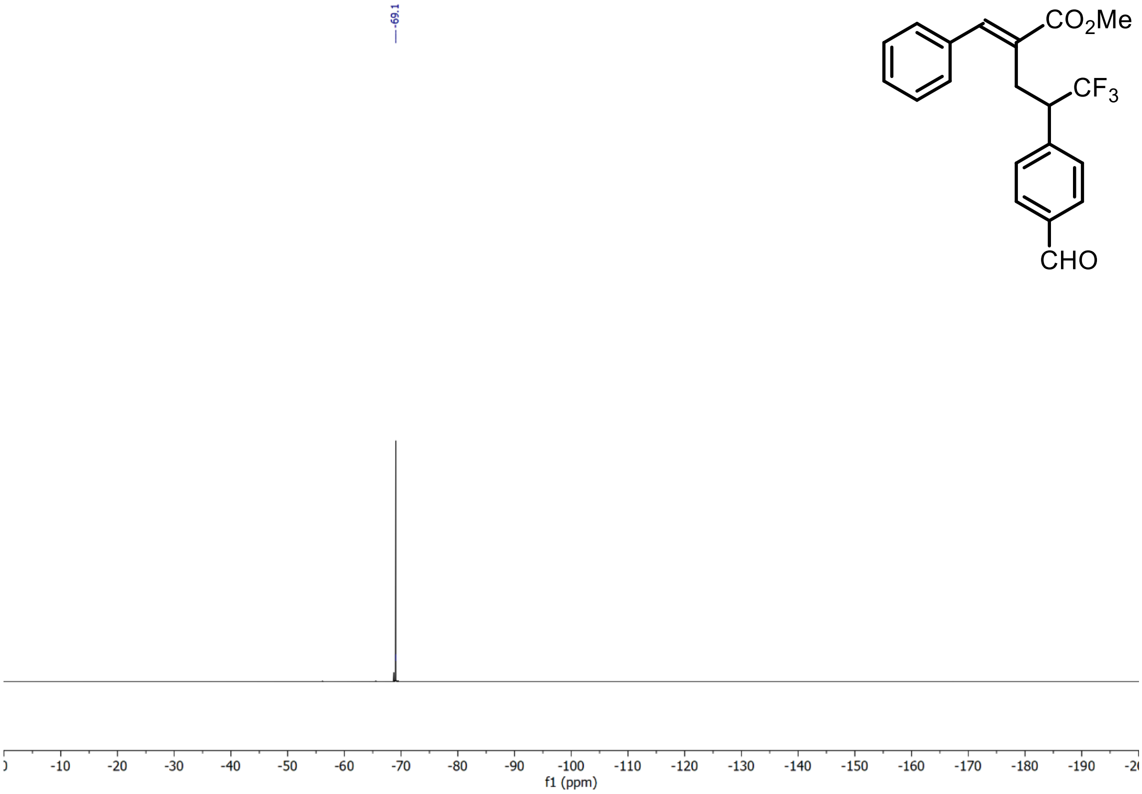

^{19}F NMR (471 MHz, $CDCl_3$) spectra of **4i**.

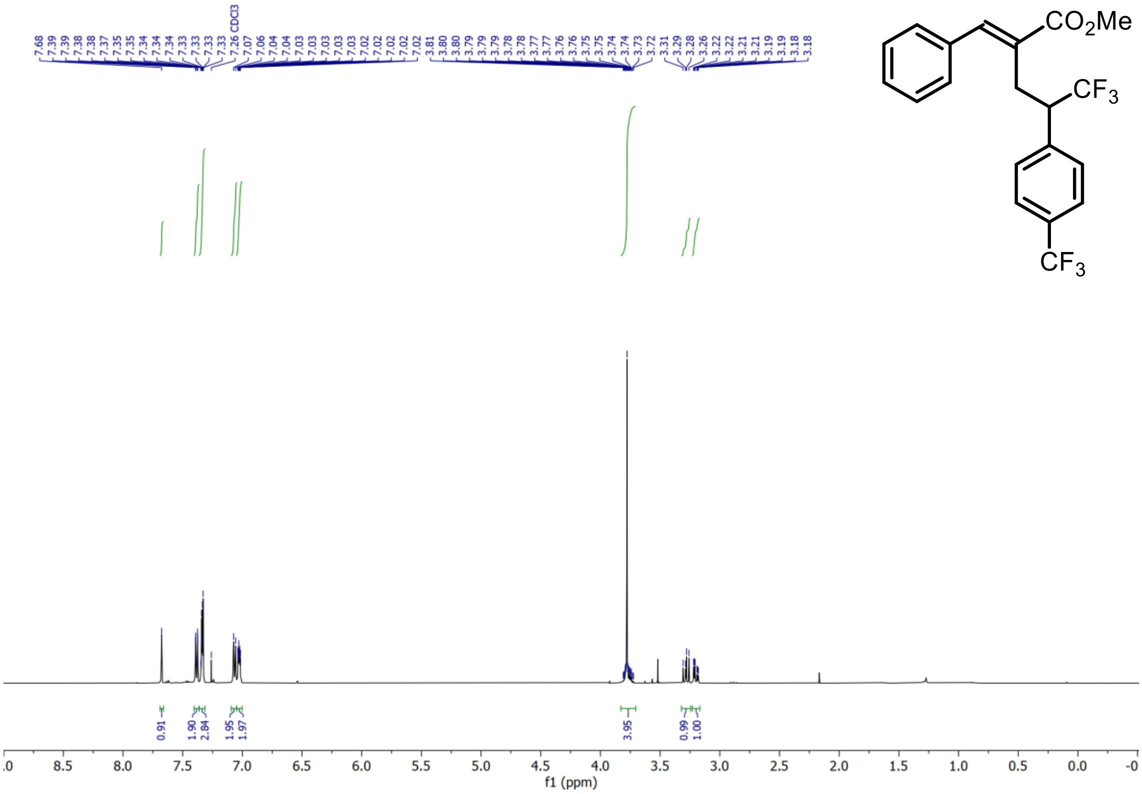

^{1}H NMR (500 MHz, $CDCl_3$) spectra of **4j**.

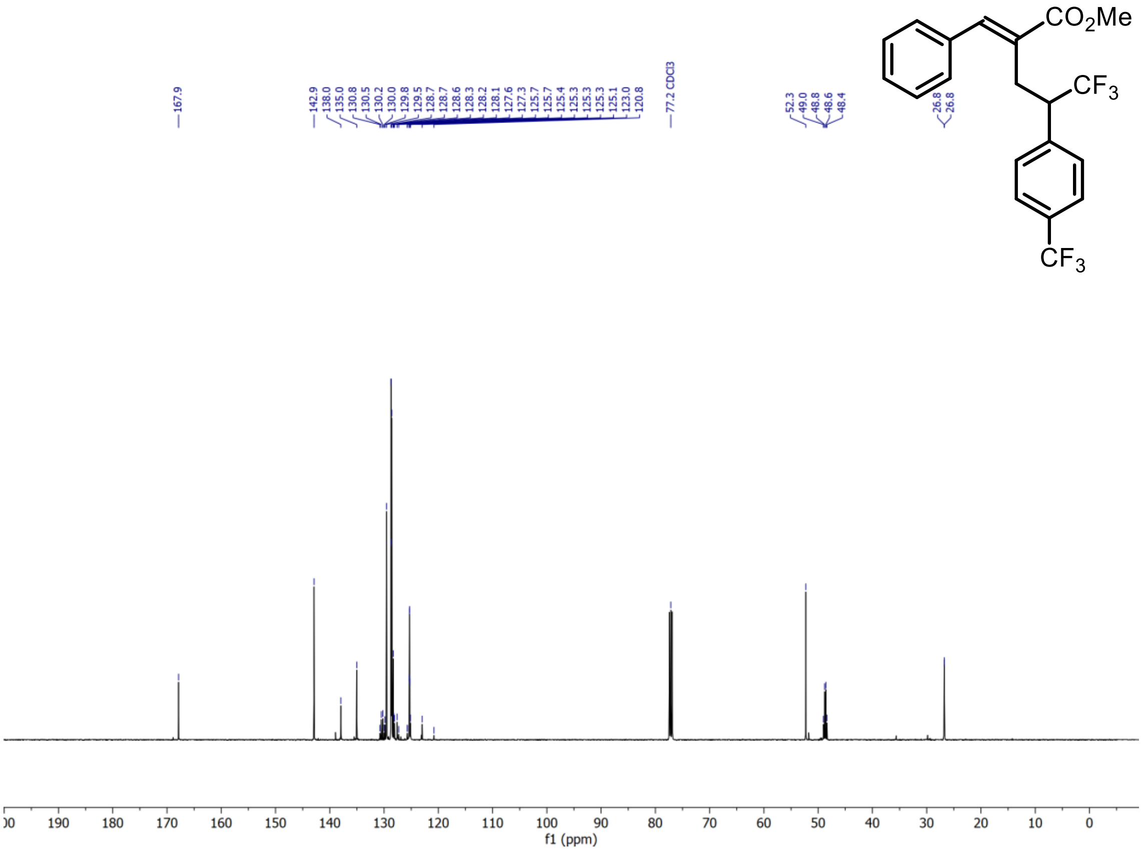

^{13}C NMR (126 MHz, $CDCl_3$) spectra of **4j**.

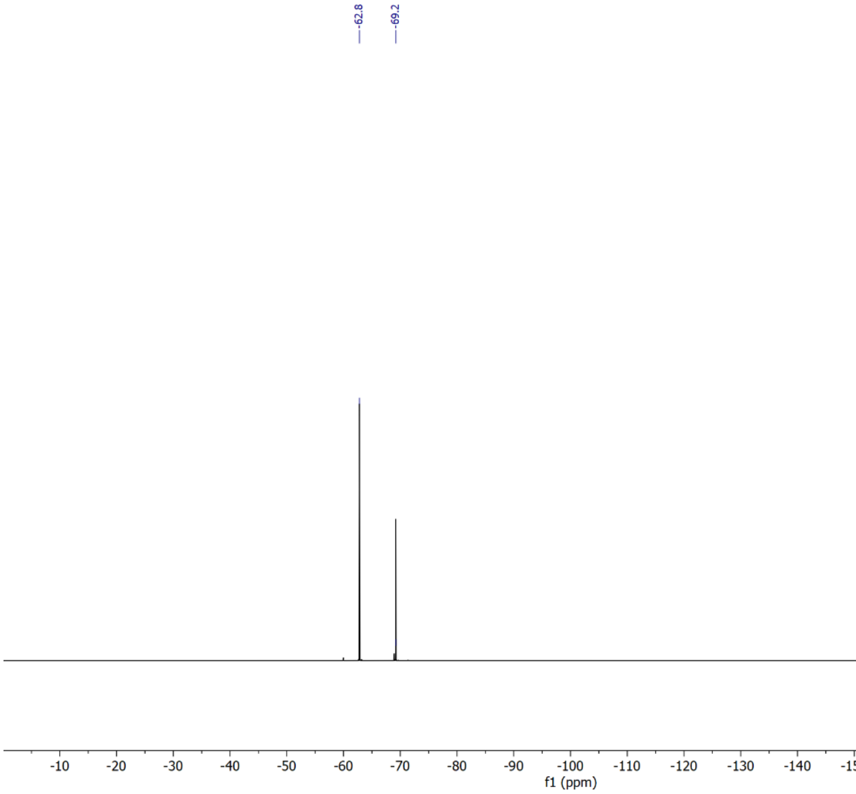

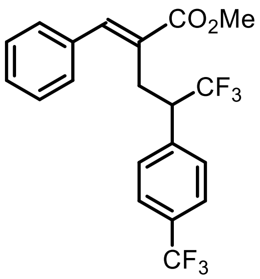

^{19}F NMR (471 MHz, $CDCl_3$) spectra of **4j**.

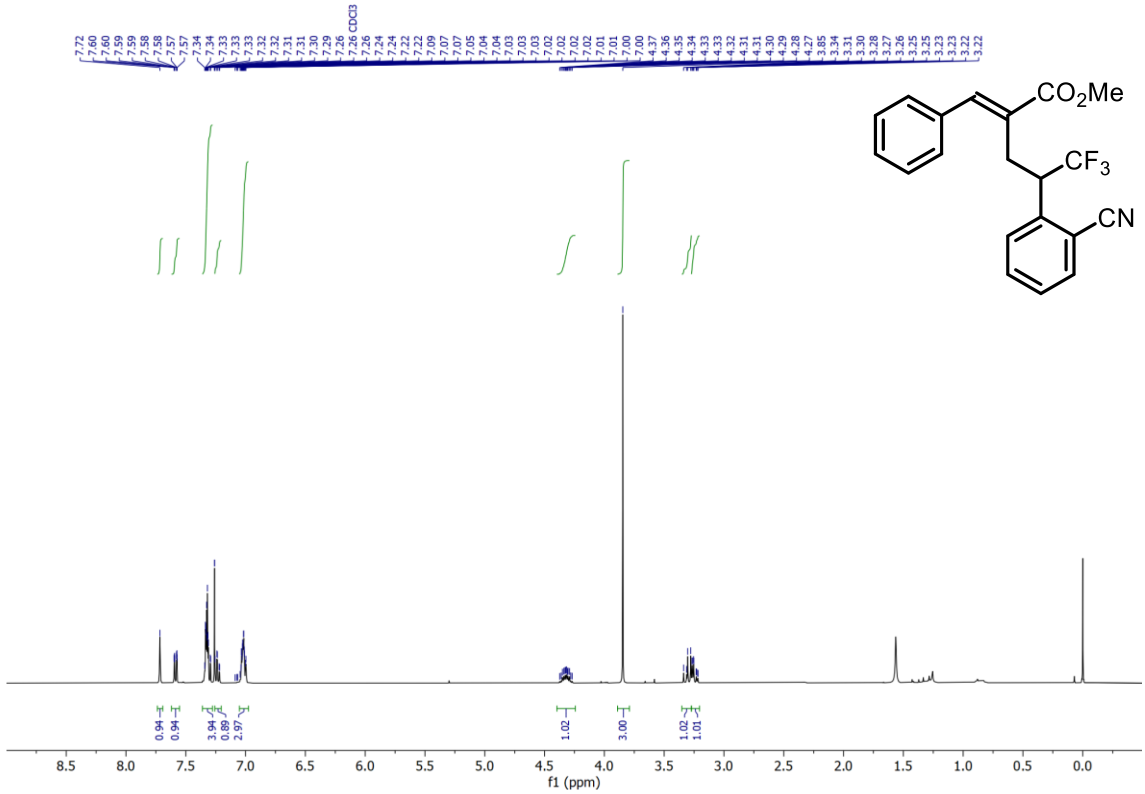

^{1}H NMR (400 MHz, $CDCl_3$) spectra of **4k**.

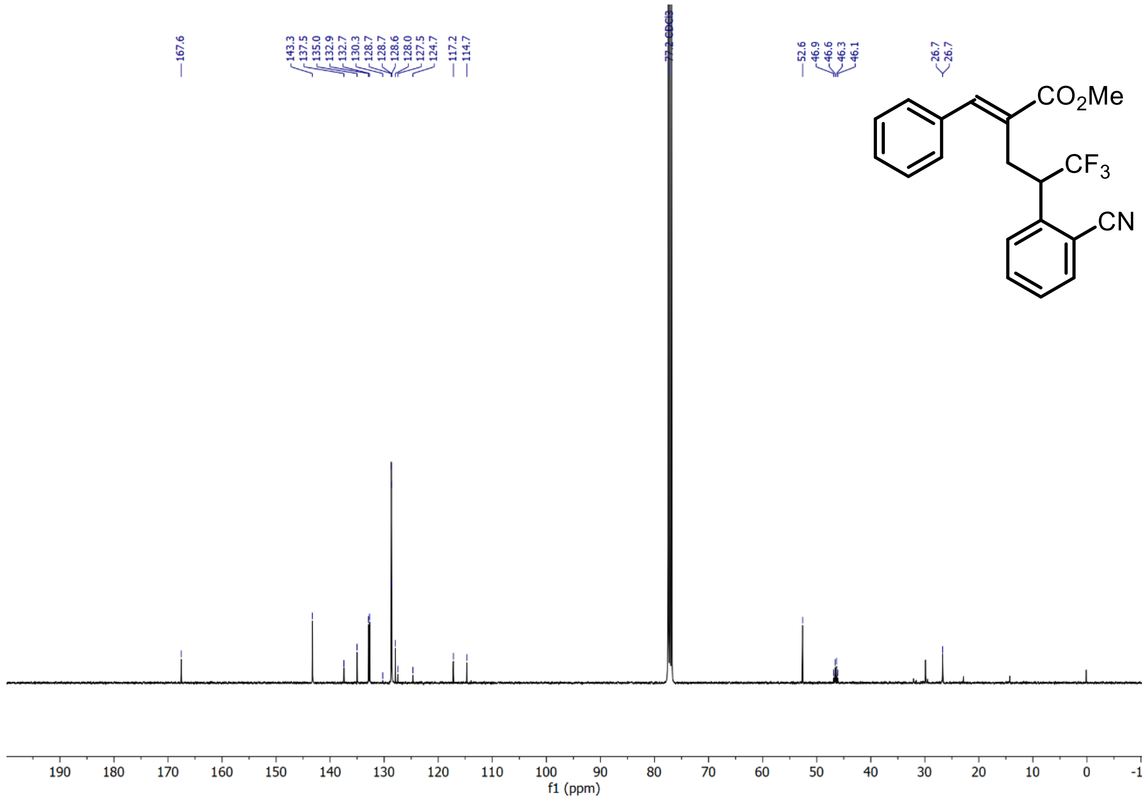

^{13}C NMR (101 MHz, $CDCl_3$) spectra of **4k**.

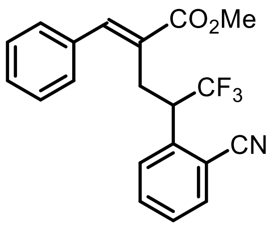

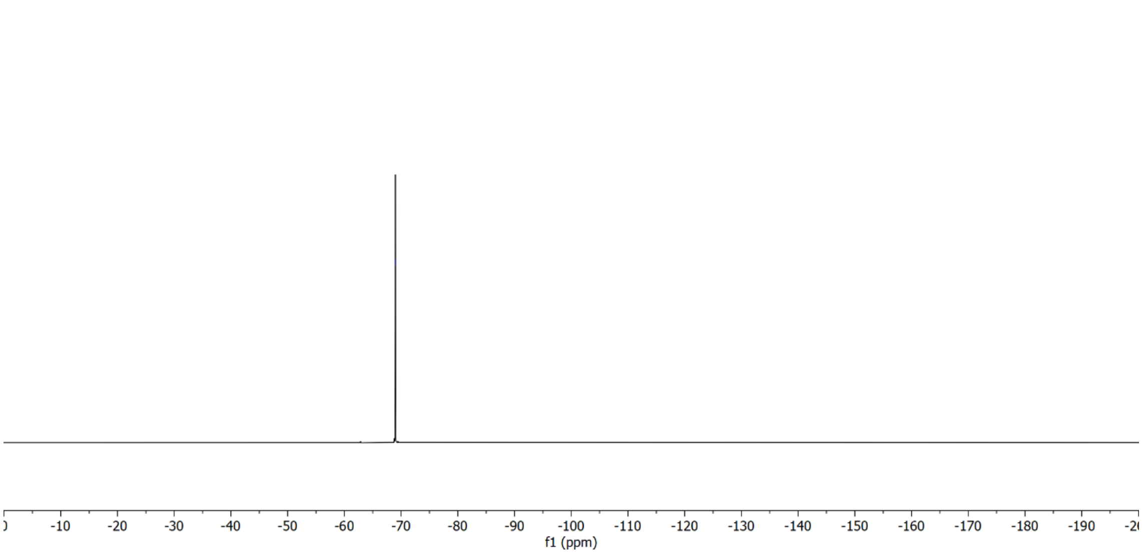

^{19}F NMR (471 MHz, $CDCl_3$) spectra of **4k**.

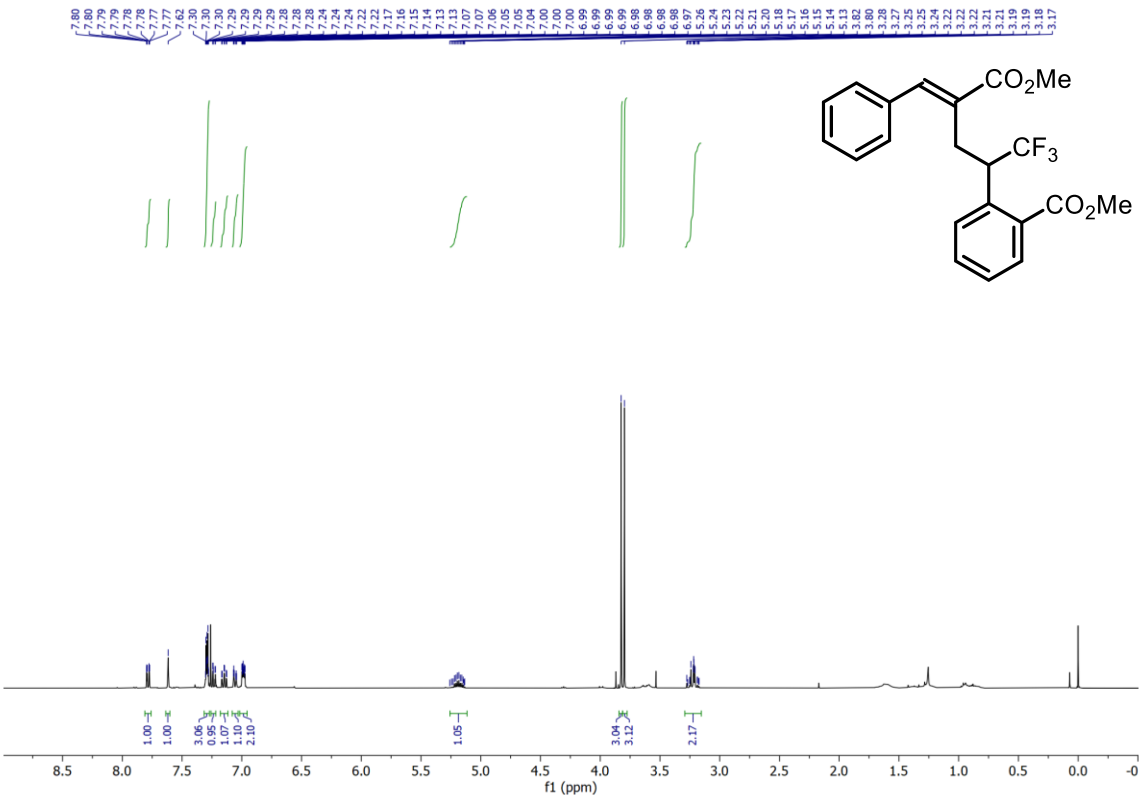

^{1}H NMR (400 MHz, $CDCl_3$) spectra of **4l**.

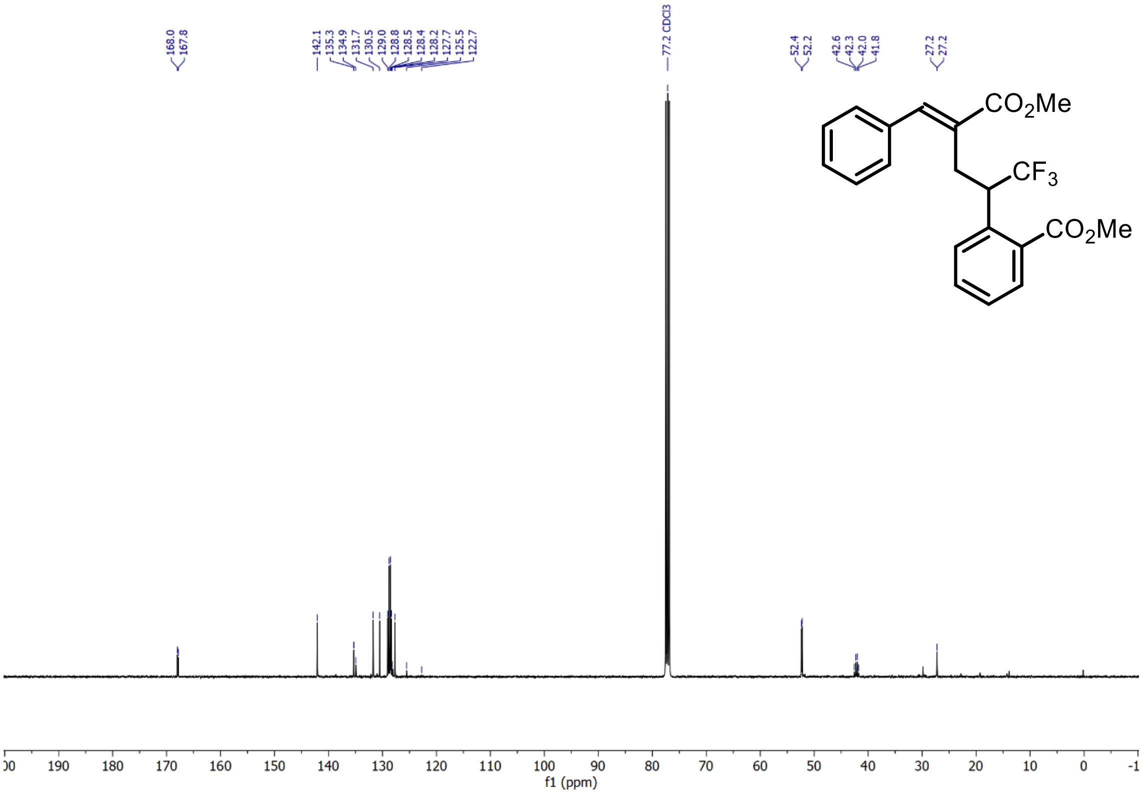

^{13}C NMR (101 MHz, $CDCl_3$) spectra of **4l**.

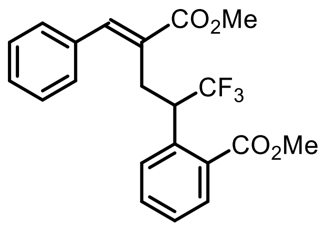

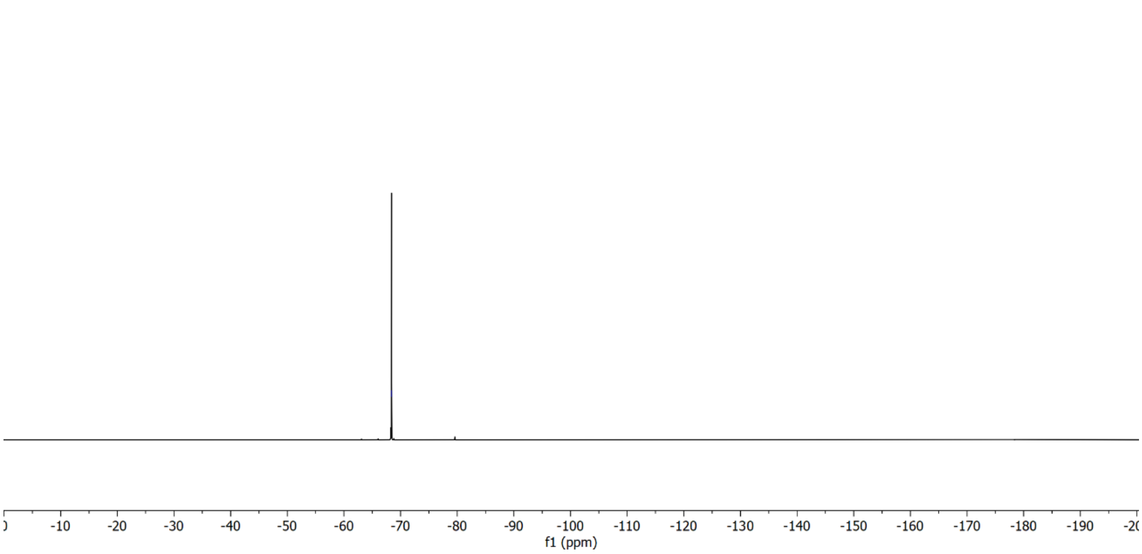

^{19}F NMR (471 MHz, $CDCl_3$) spectra of **4l**.

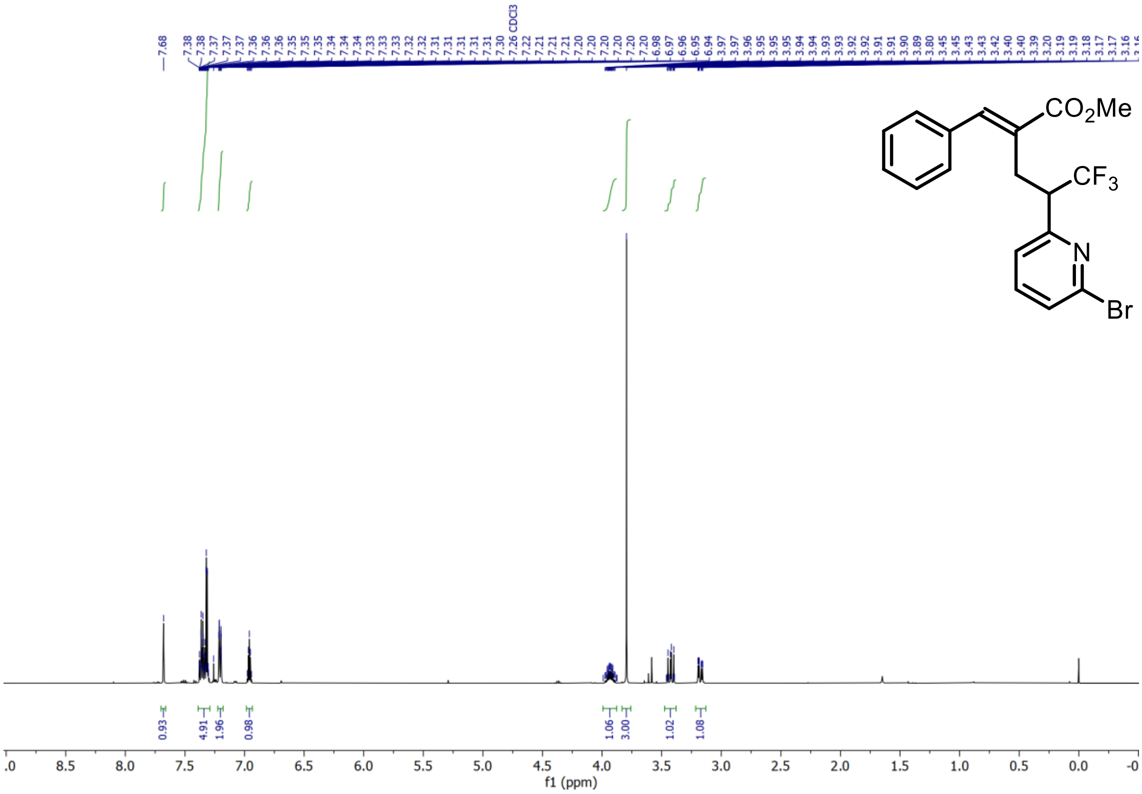

^{1}H NMR (500 MHz, $CDCl_3$) spectra of **4m**.

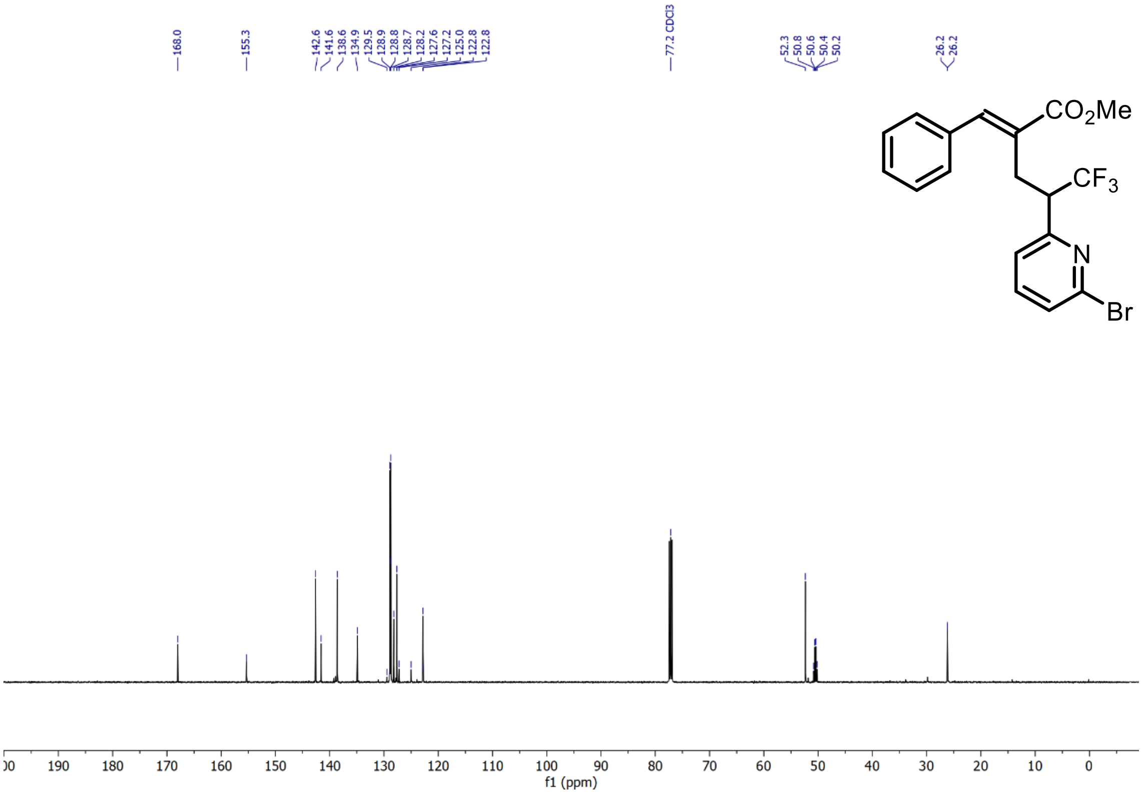

^{13}C NMR (126 MHz, $CDCl_3$) spectra of **4m**.

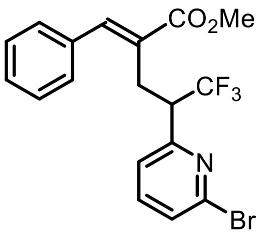

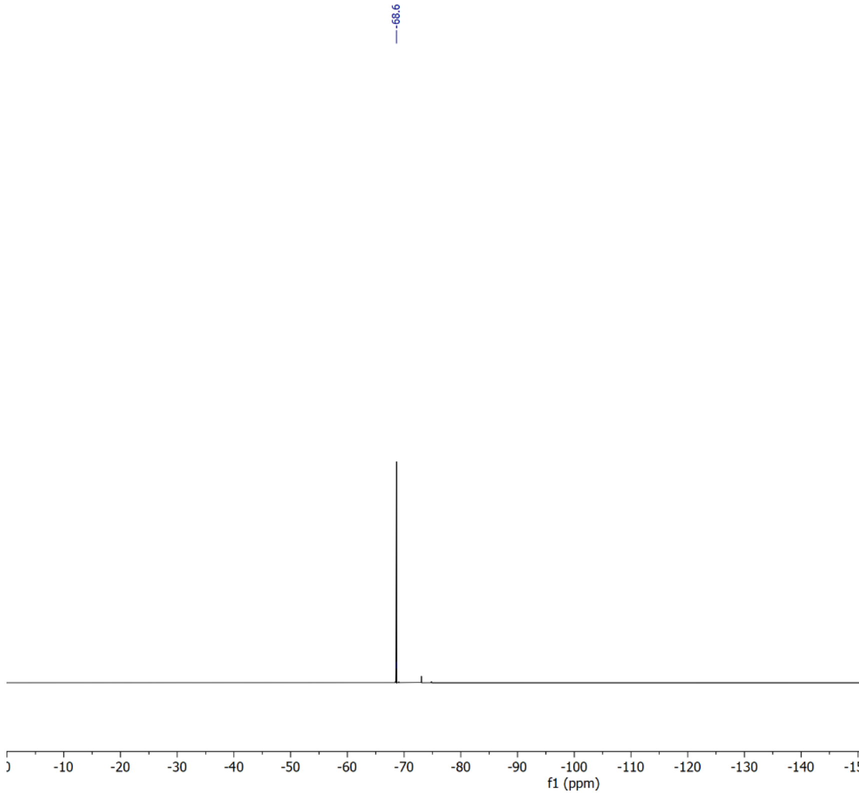

^{19}F NMR (471 MHz, $CDCl_3$) spectra of **4m**.

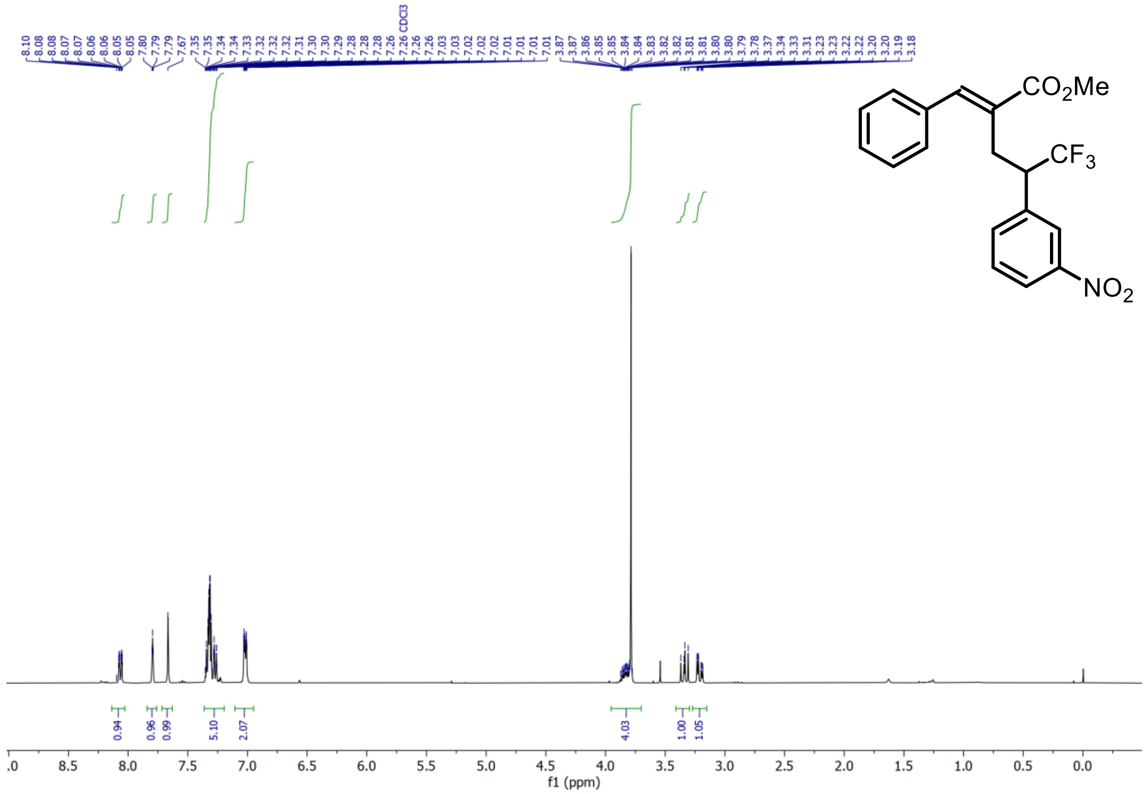

^{1}H NMR (400 MHz, $CDCl_3$) spectra of **4n**.

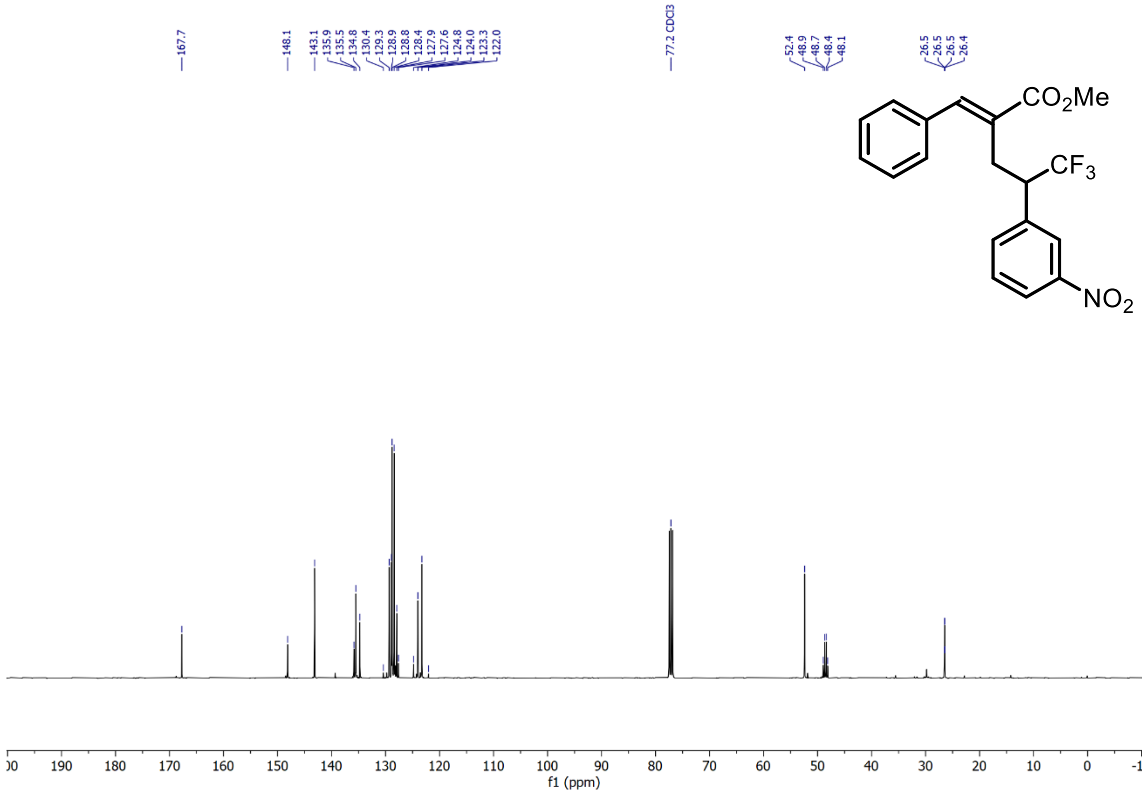

^{13}C NMR (101 MHz, $CDCl_3$) spectra of **4n**.

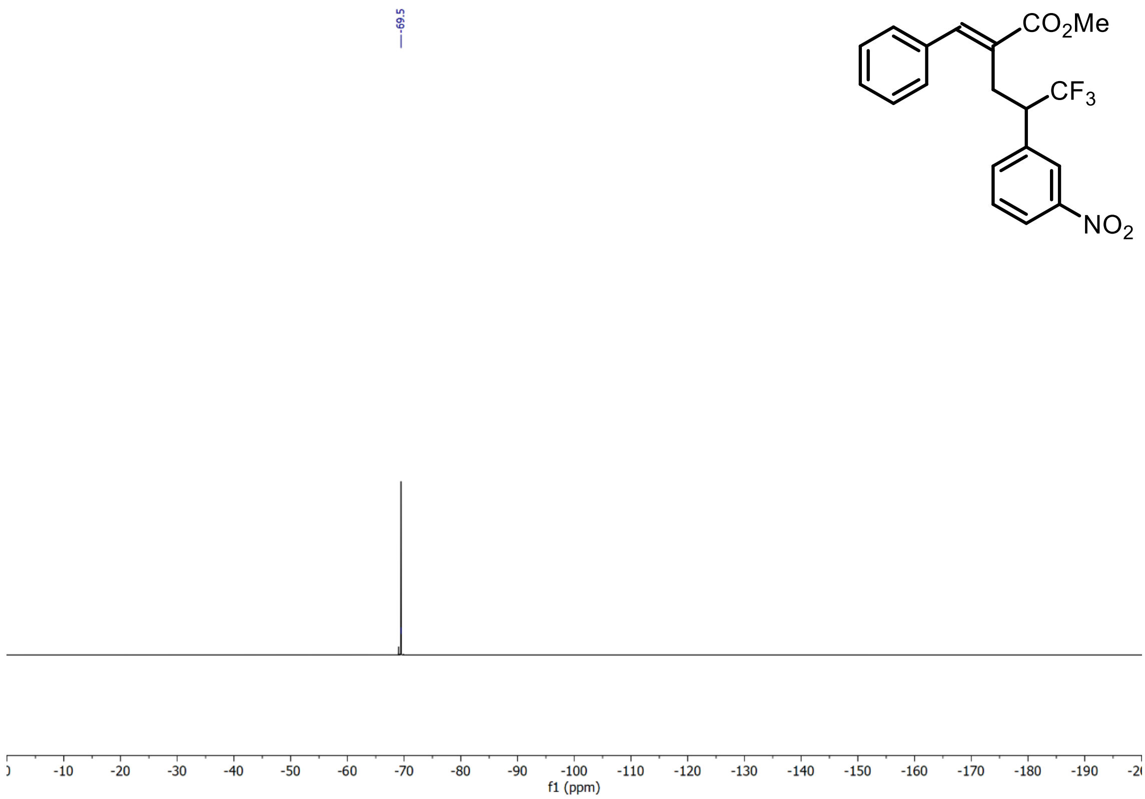

^{19}F NMR (471 MHz, $CDCl_3$) spectra of **4n**.

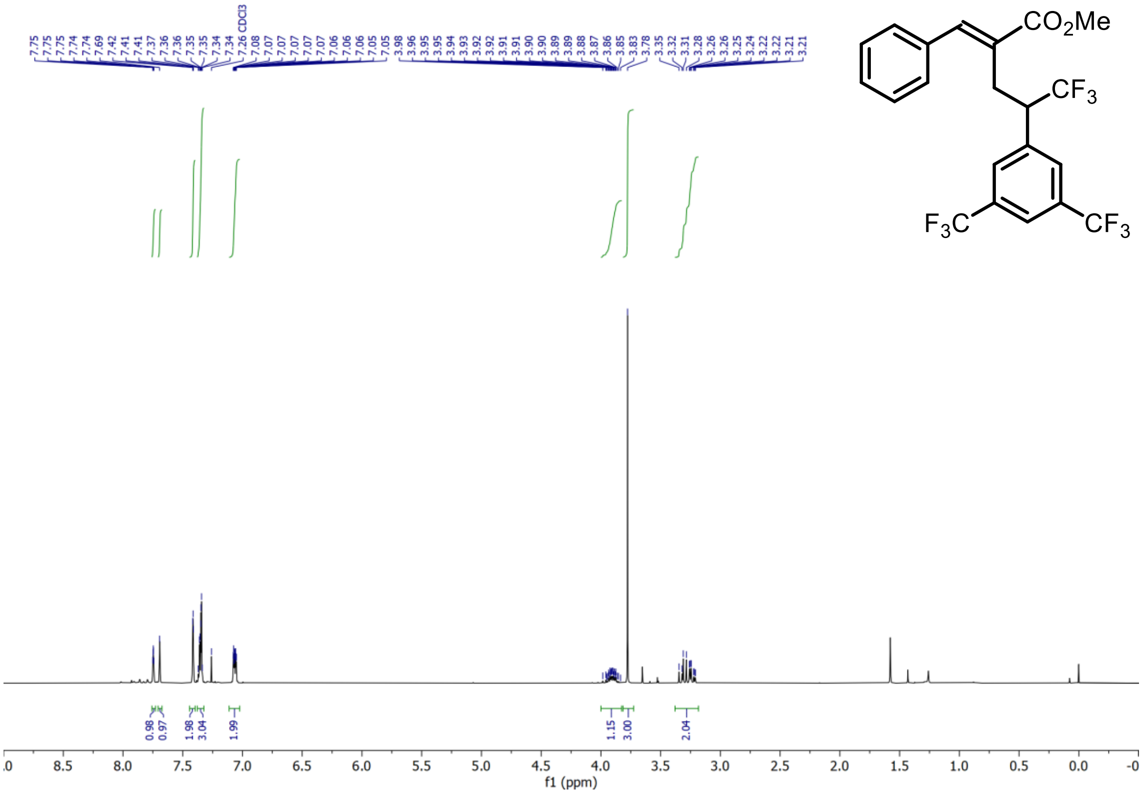

^{1}H NMR (400 MHz, $CDCl_3$) spectra of **4o**.

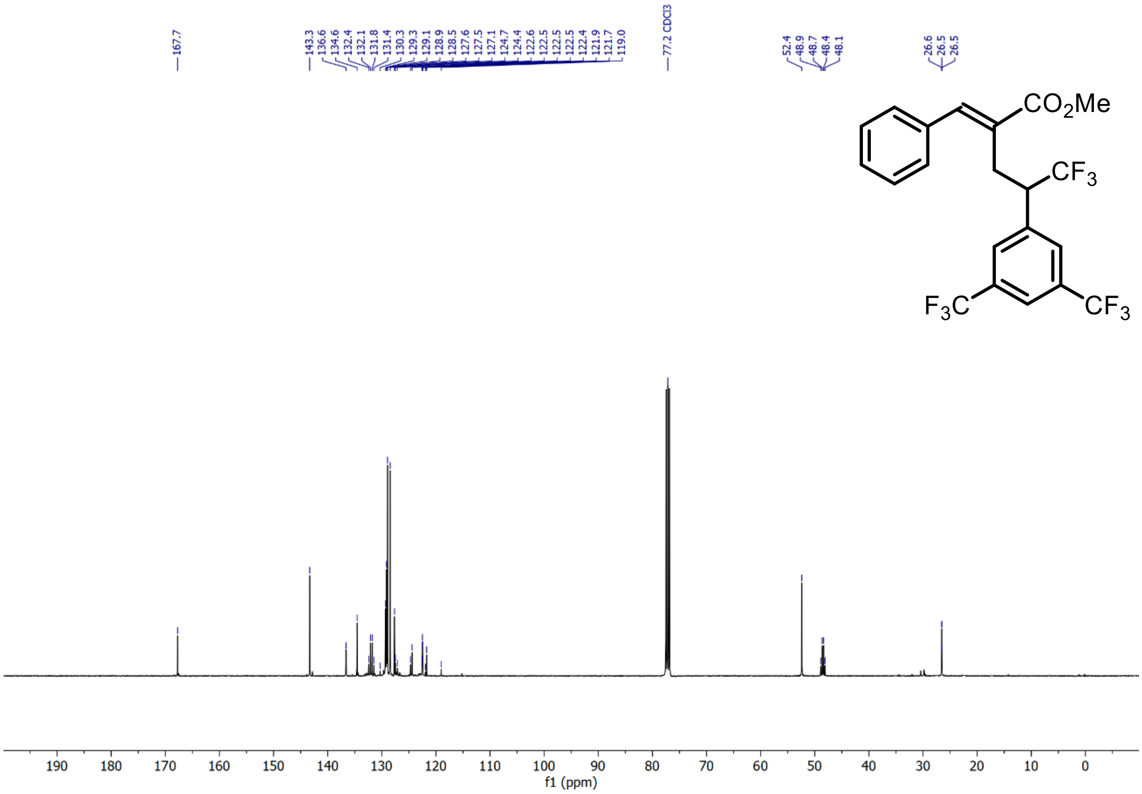

^{13}C NMR (101 MHz, $CDCl_3$) spectra of **4o**.

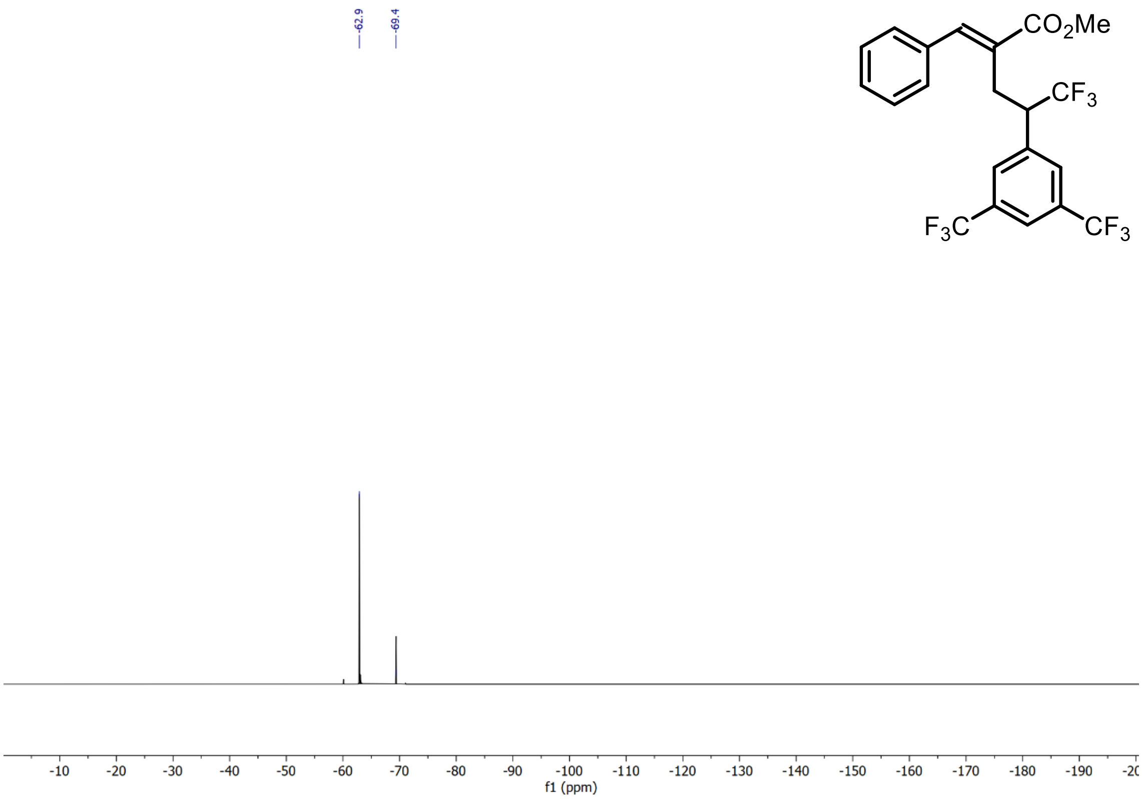

^{19}F NMR (471 MHz, $CDCl_3$) spectra of **4o**.

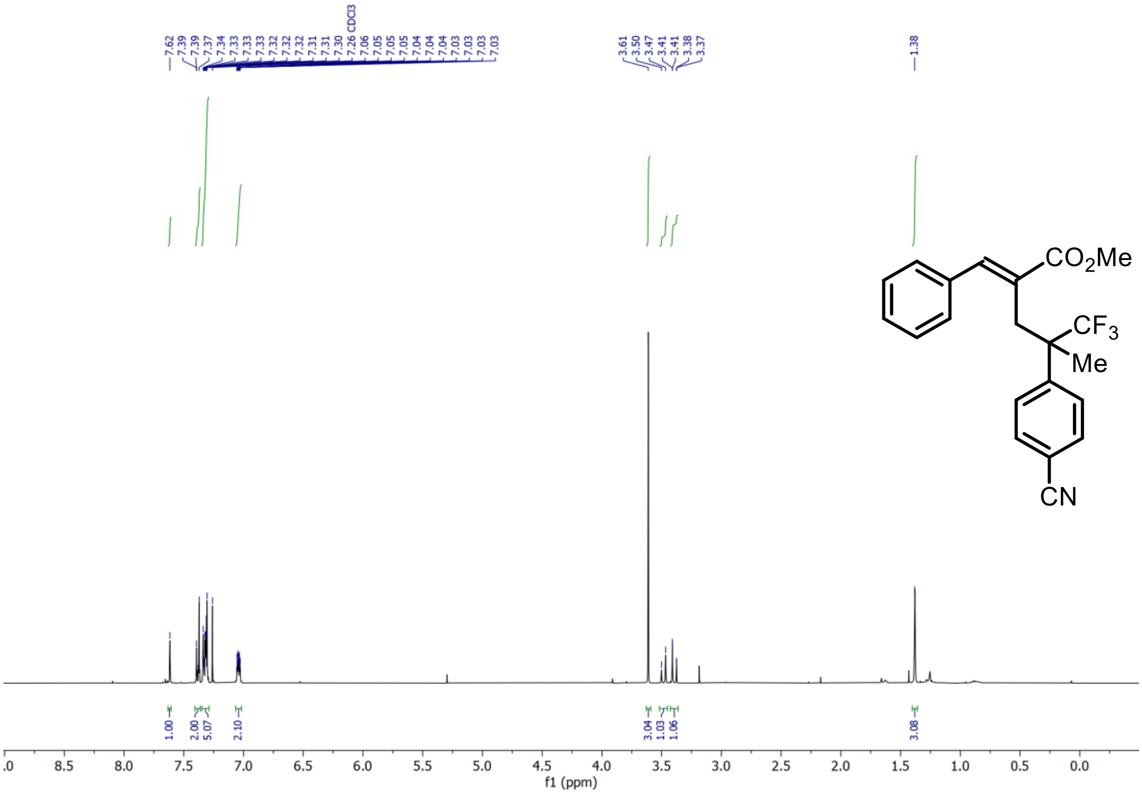

^{1}H NMR (400 MHz, $CDCl_3$) spectra of **4p**.

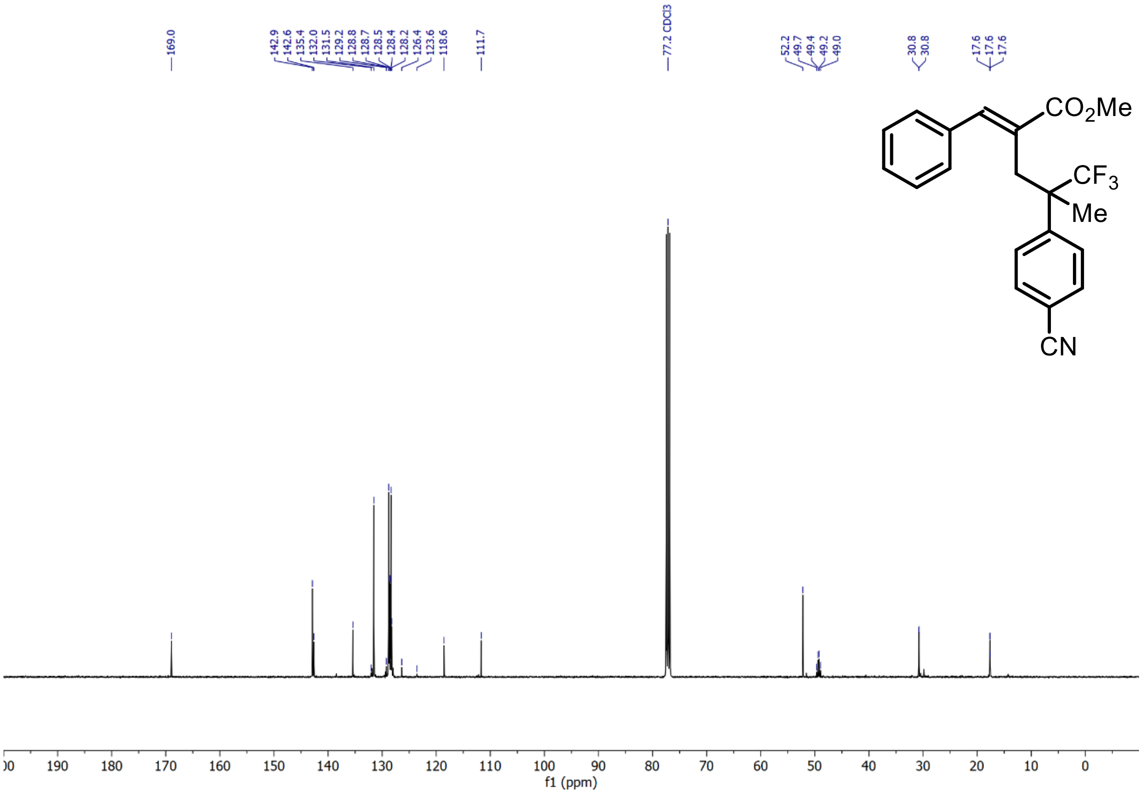

^{13}C NMR (101 MHz, $CDCl_3$) spectra of **4p**.

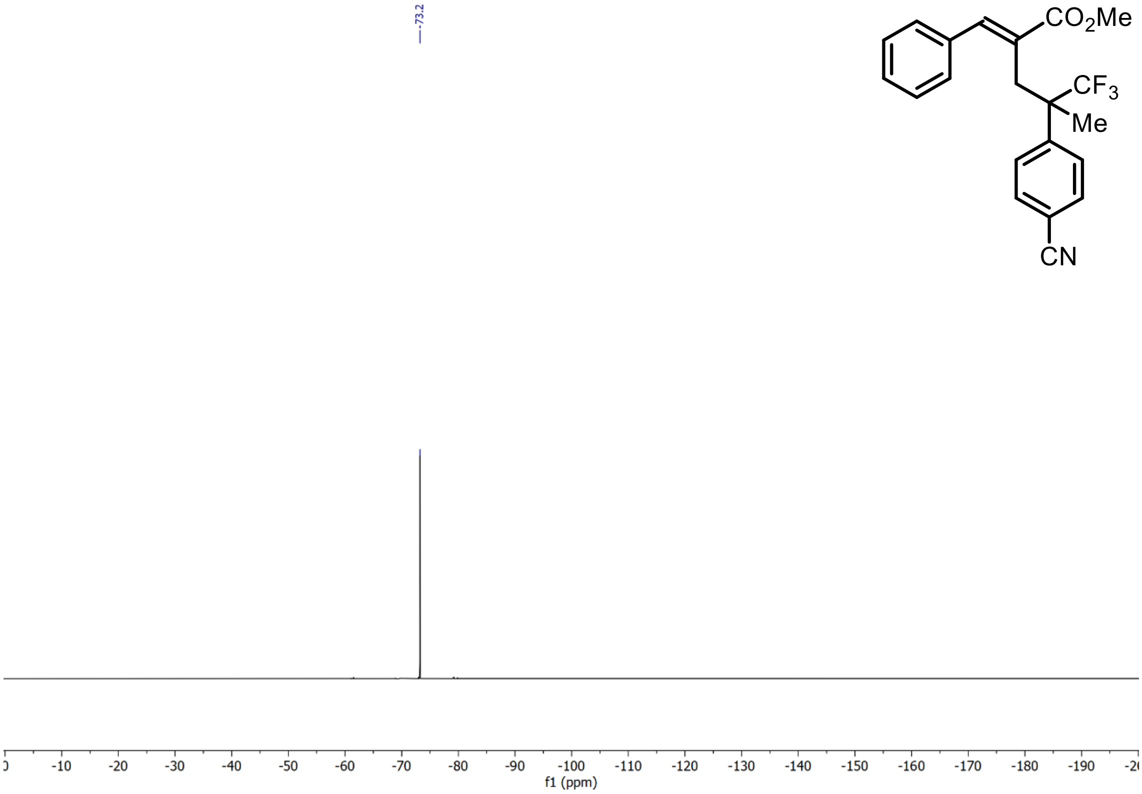

^{19}F NMR (376 MHz, $CDCl_3$) spectra of **4p**.

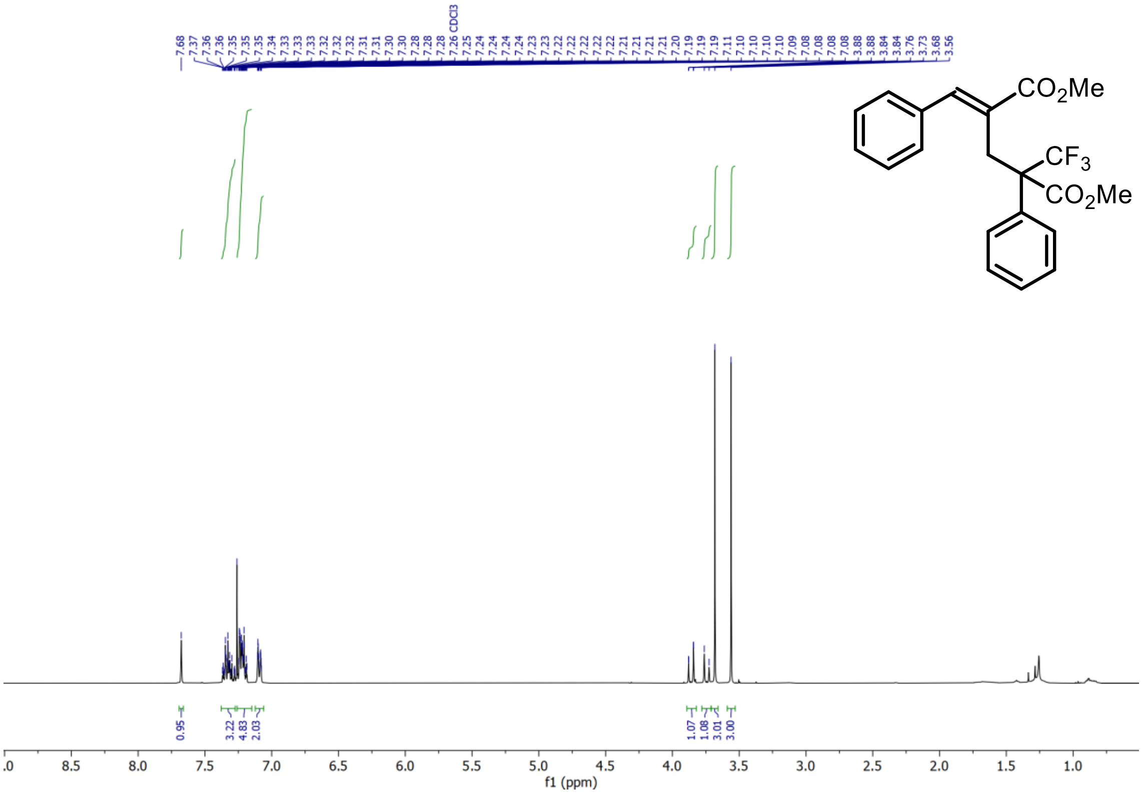

^{1}H NMR (400 MHz, $CDCl_3$) spectra of **4q**.

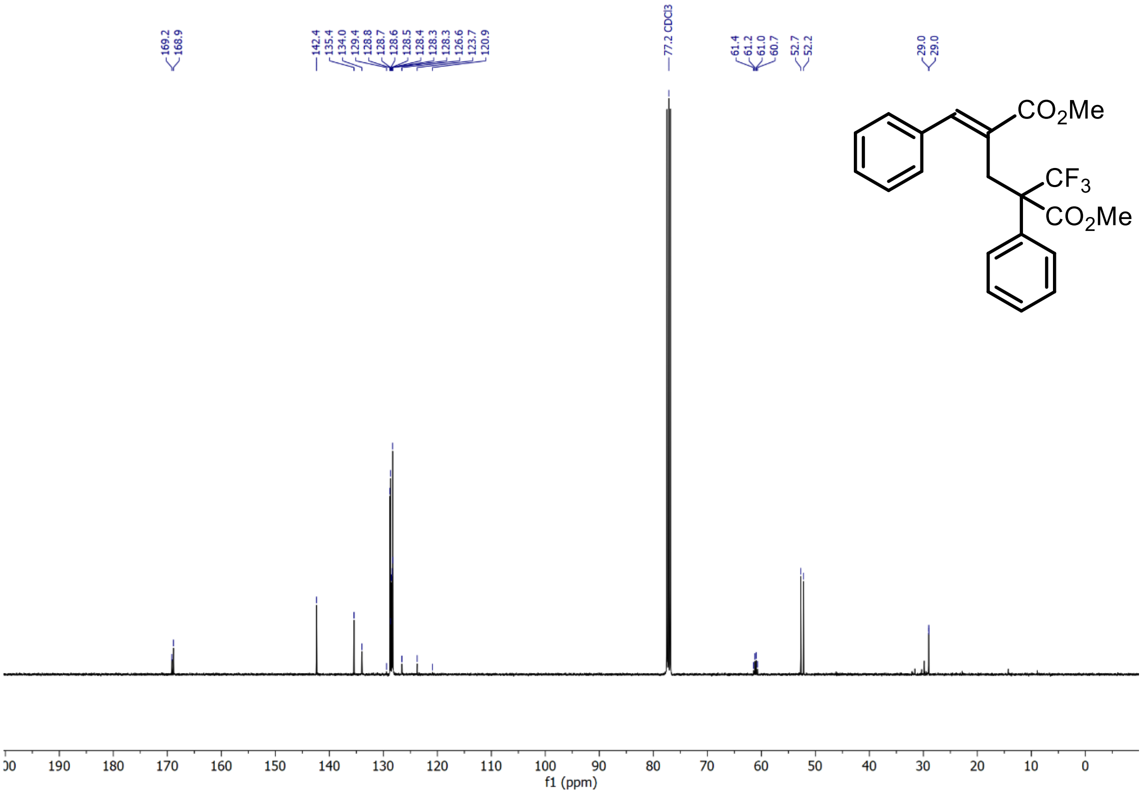

^{13}C NMR (101 MHz, $CDCl_3$) spectra of **4q**.

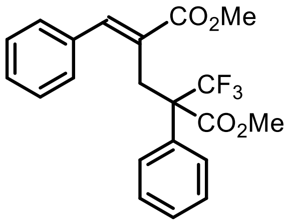

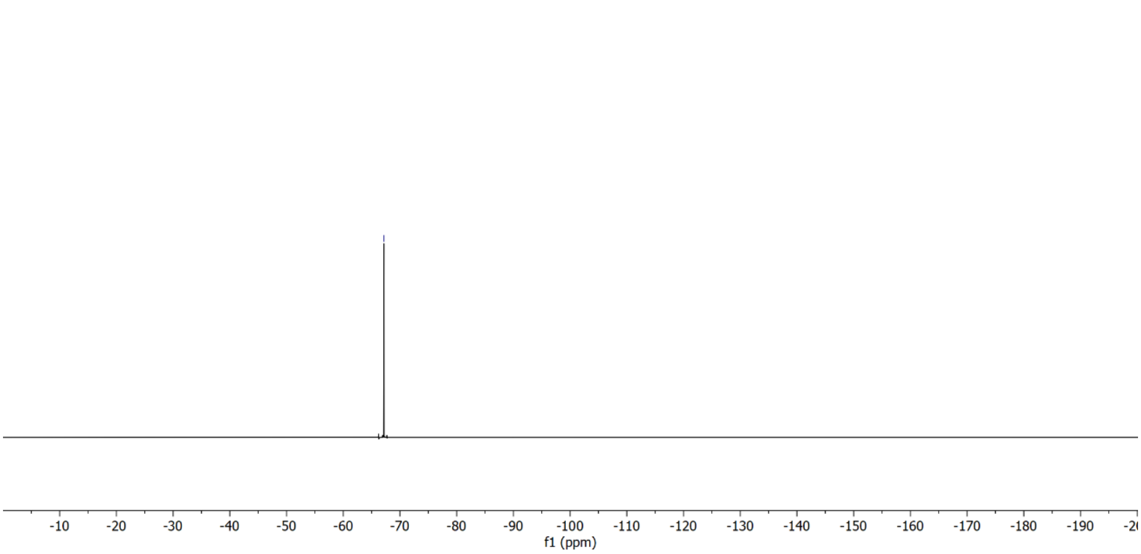

^{19}F NMR (376 MHz, $CDCl_3$) spectra of **4q**.

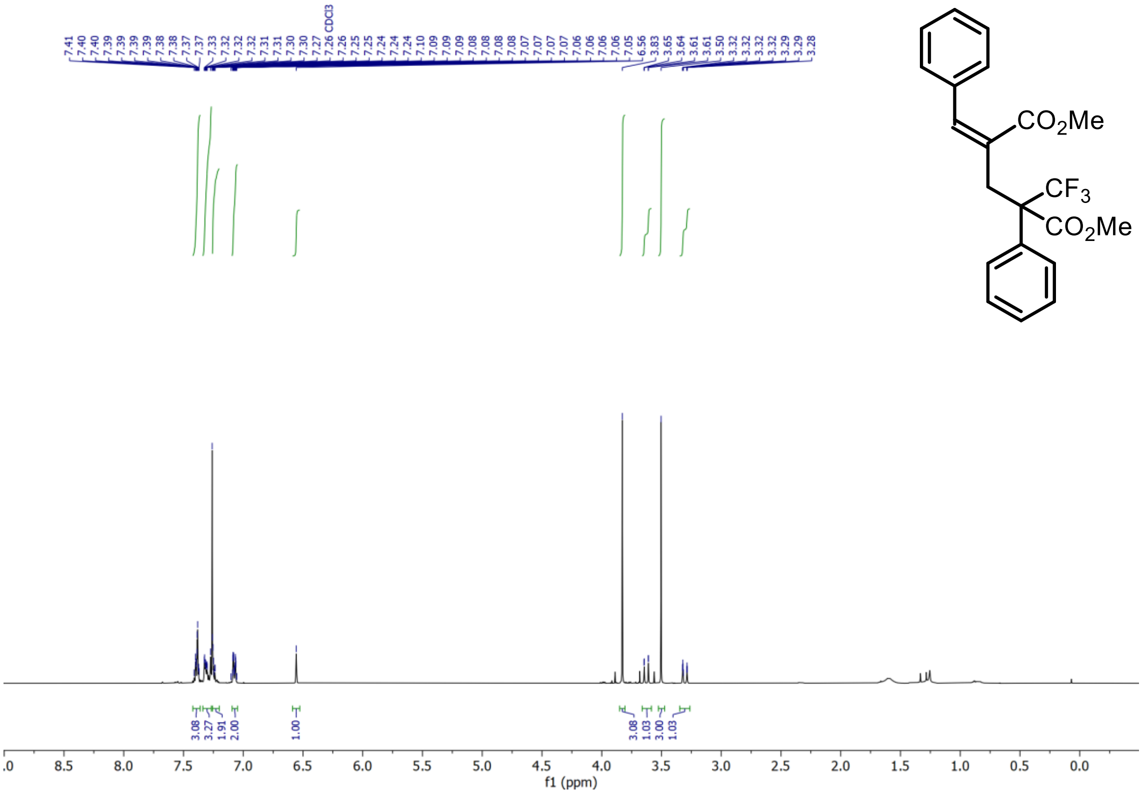

^{1}H NMR (400 MHz, $CDCl_3$) spectra of **4q’**.

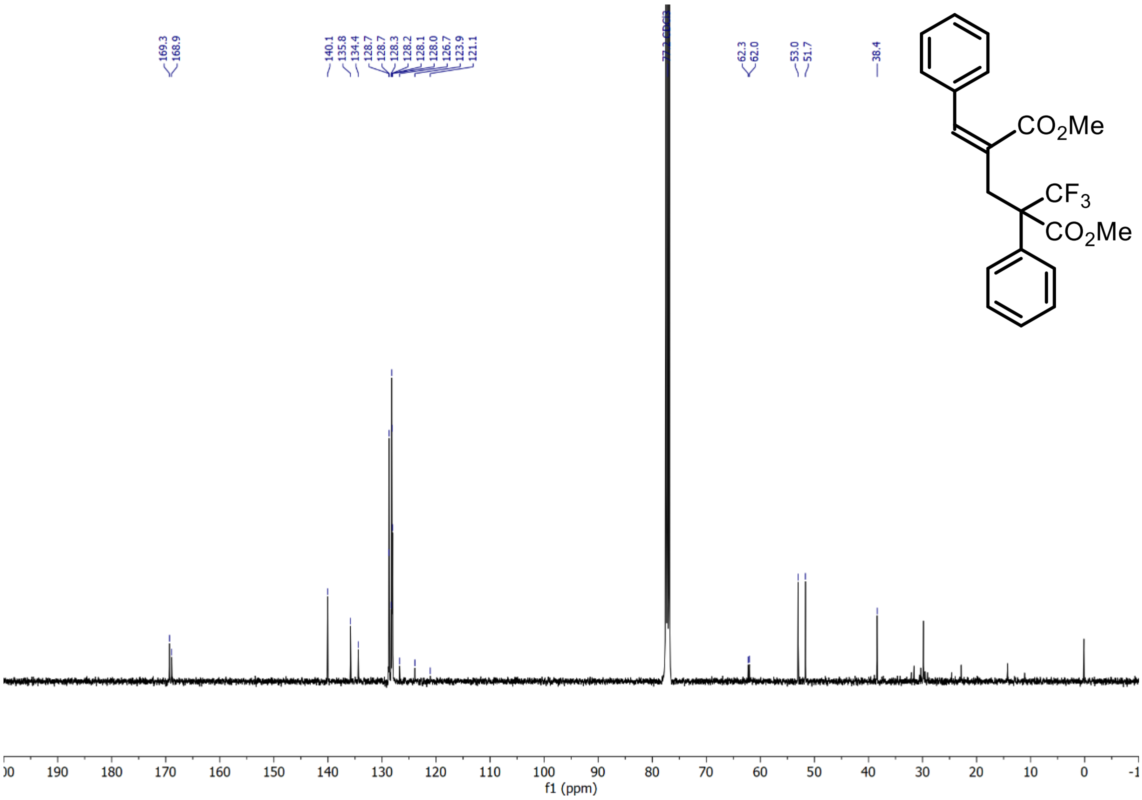

^{13}C NMR (101 MHz, $CDCl_3$) spectra of **4q’**.

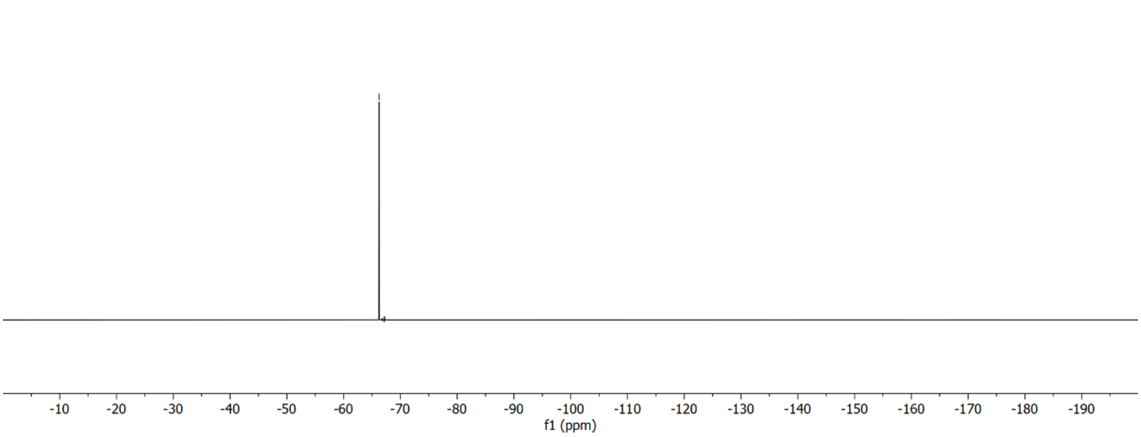

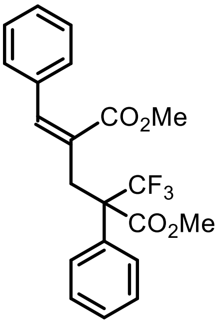

^{19}F NMR (376 MHz, $CDCl_3$) spectra of **4q'**.

M.4. NMR spectra of derivatisation products S4, 6 and S5

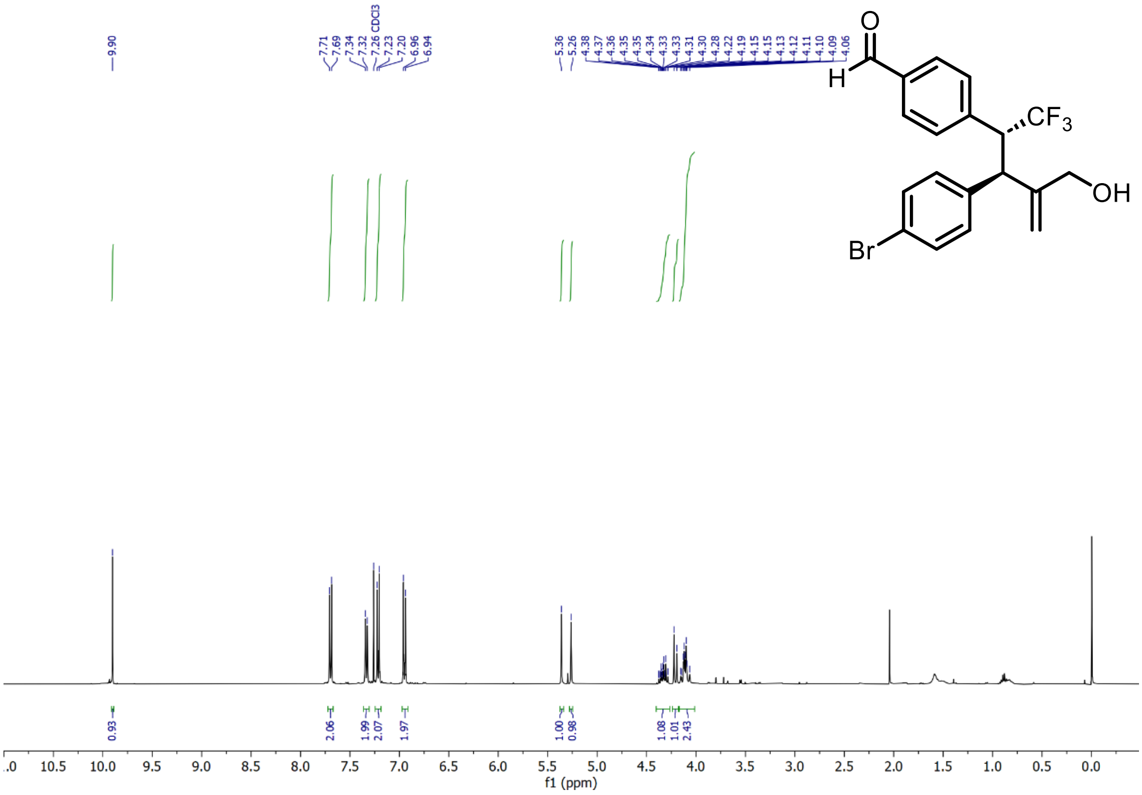

^{1}H NMR (400 MHz, $CDCl_3$) spectra of **S4**.

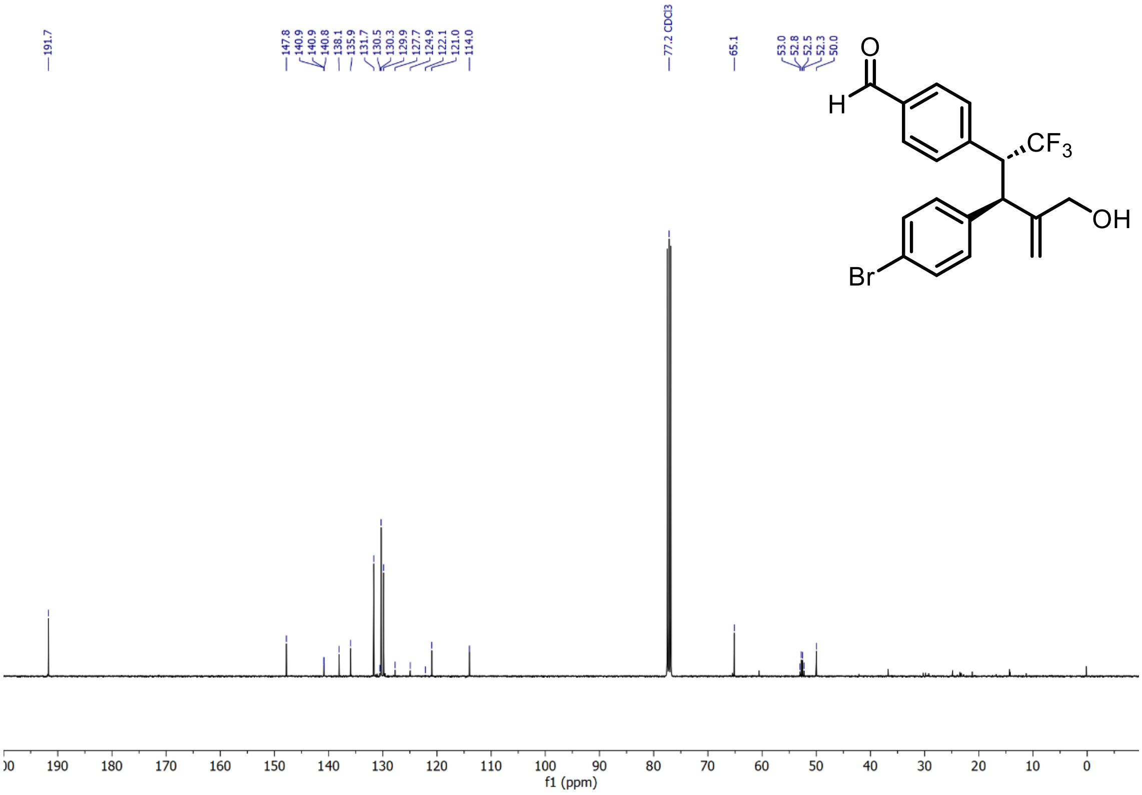

^{13}C NMR (101 MHz, $CDCl_3$) spectra of **S4**.

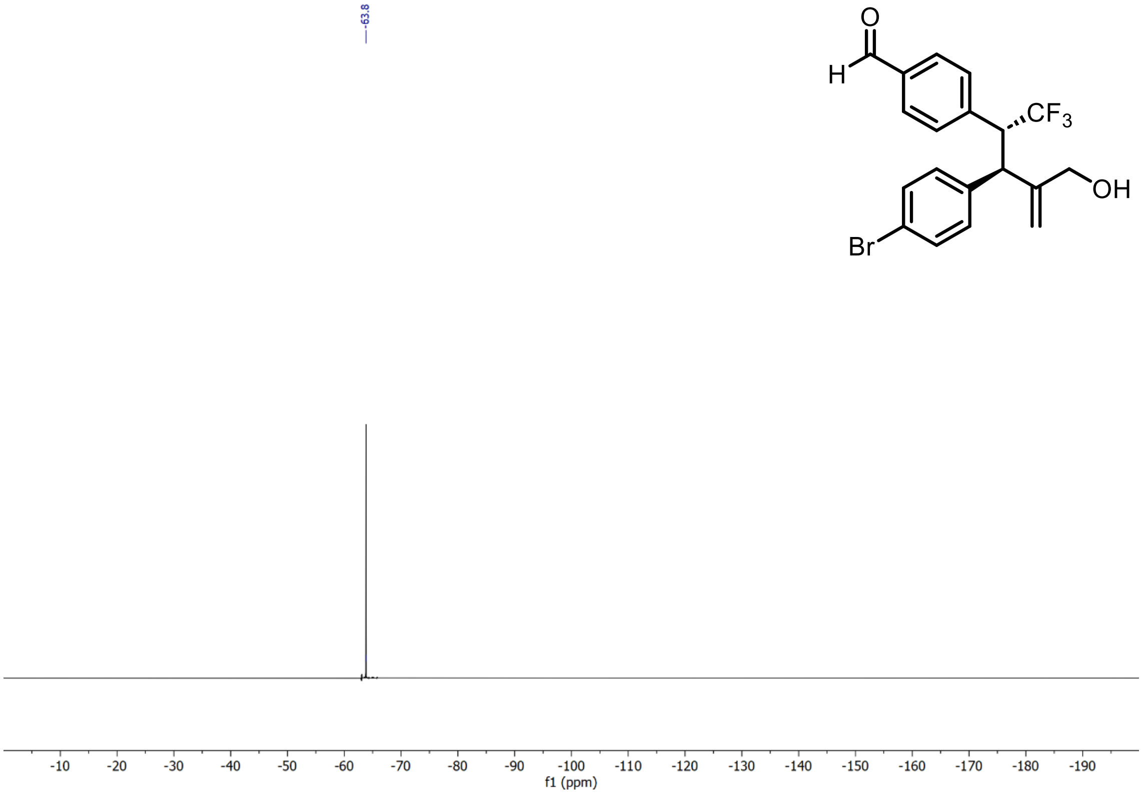

^{19}F NMR (376 MHz, $CDCl_3$) spectra of **S4**.

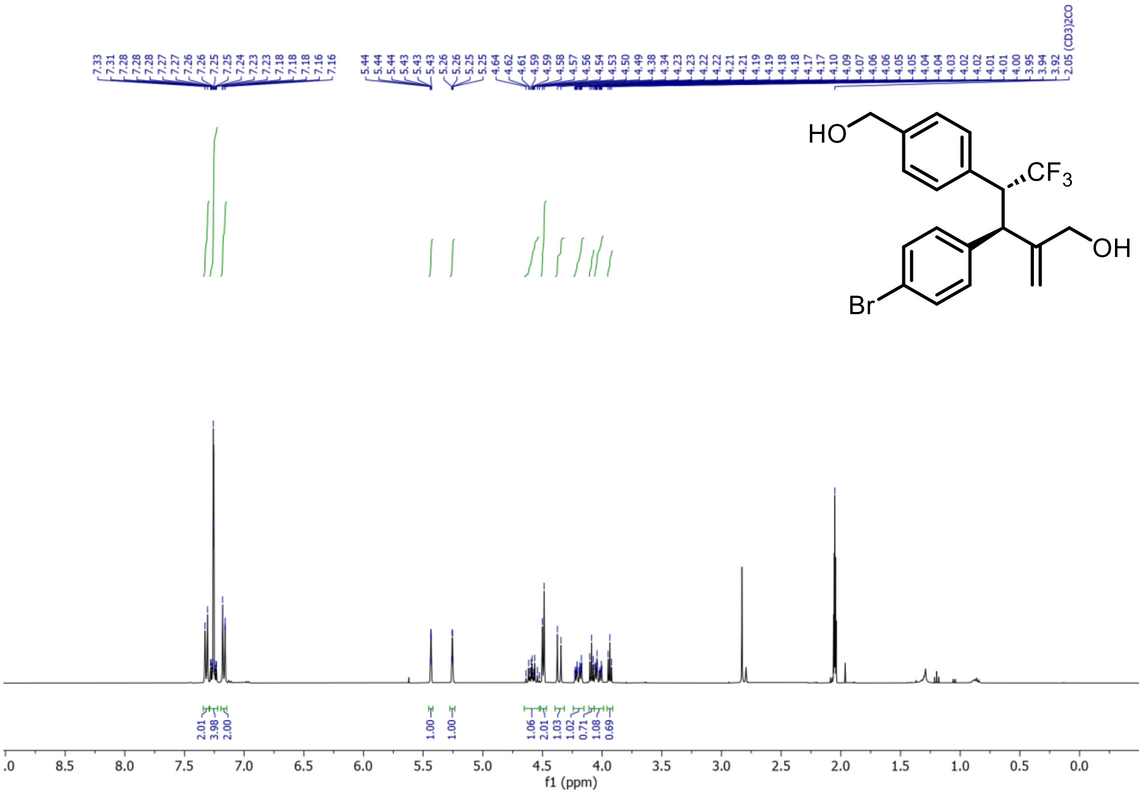

^{1}H NMR (400 MHz, Acetone_d$_6$) spectra of **6**.

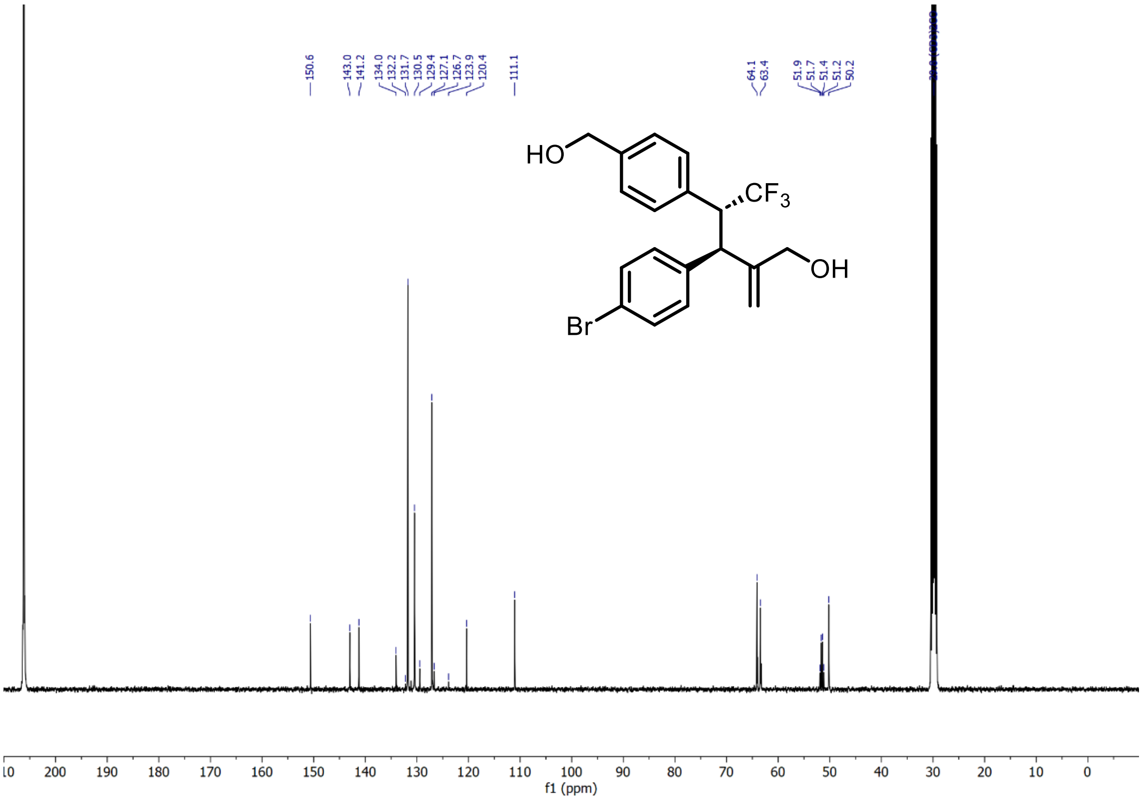

^{13}C NMR (101 MHz, Acetone_d$_6$) spectra of **6**.

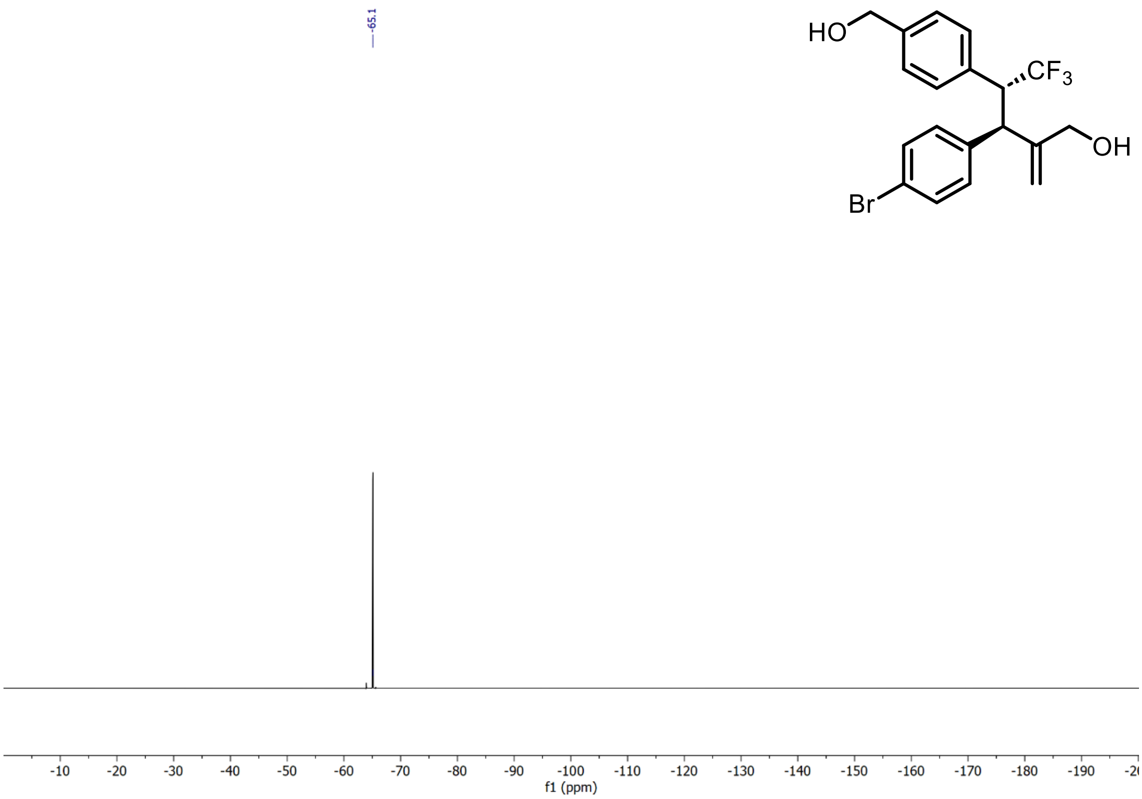

^{19}F NMR (376 MHz, Acetone_d$_6$) spectra of **6**.

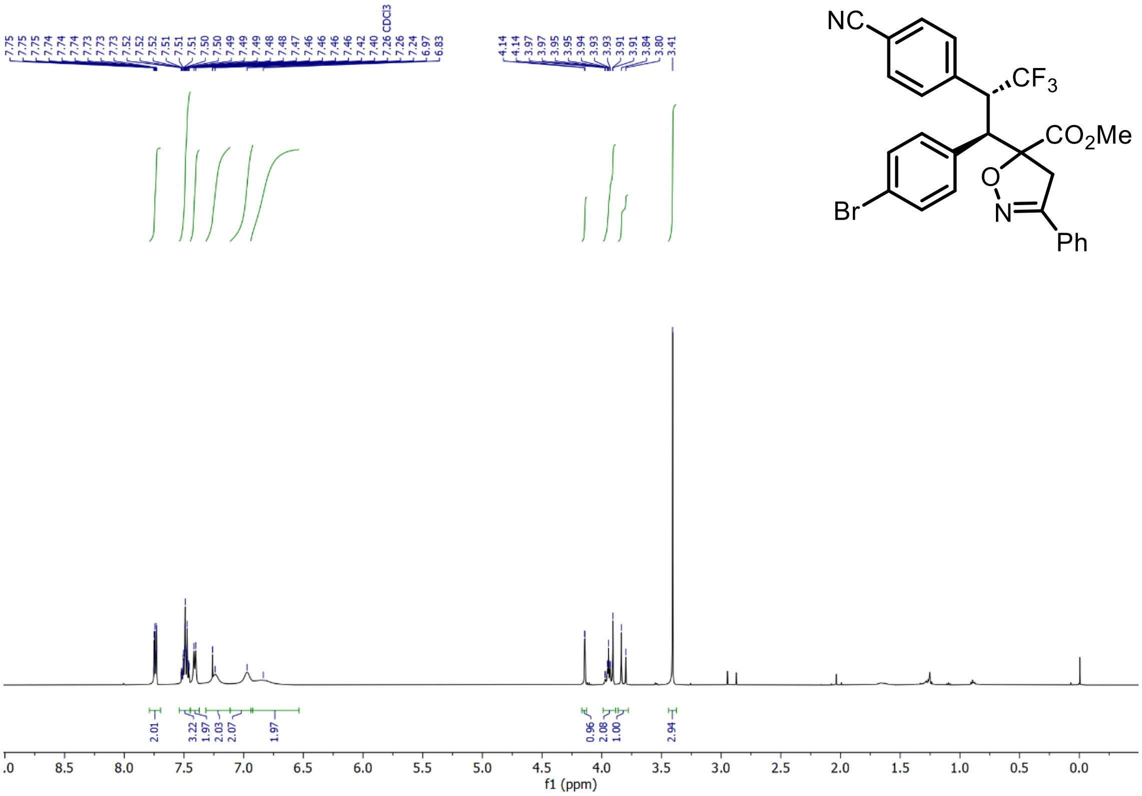

^{1}H NMR (500 MHz, $CDCl_3$) spectra of **S5**.

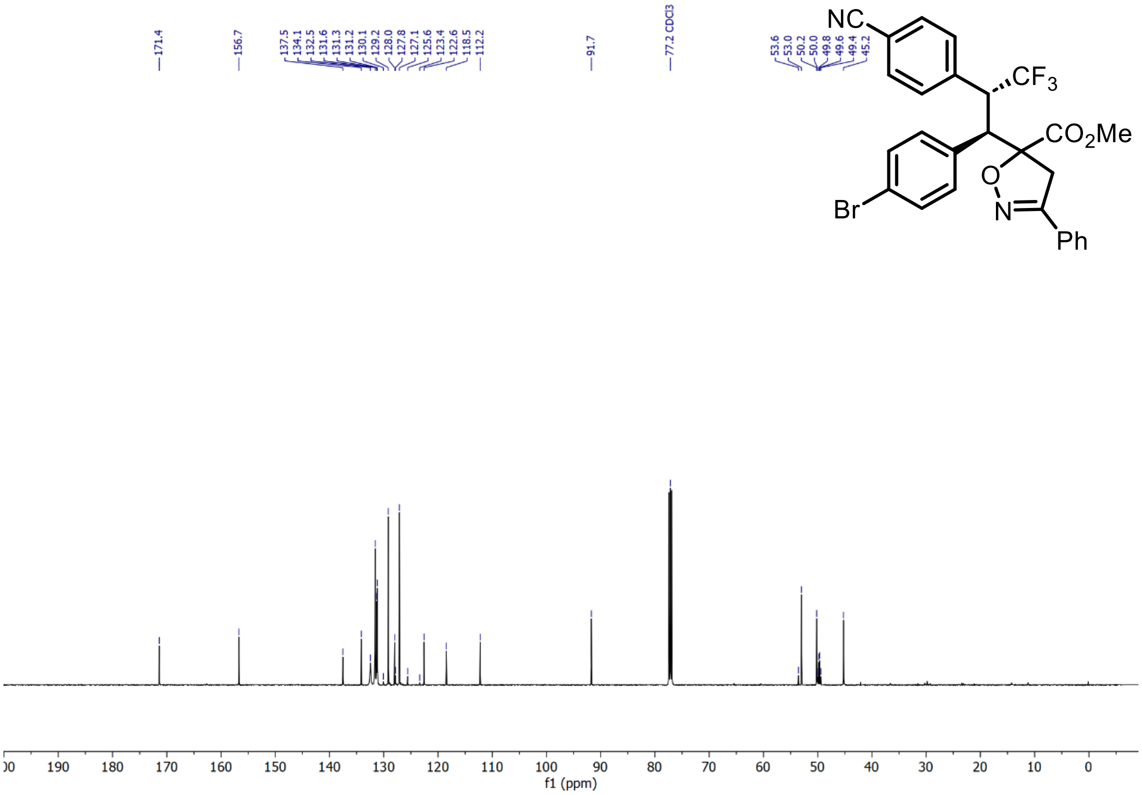

^{13}C NMR (126 MHz, $CDCl_3$) spectra of **S5**.

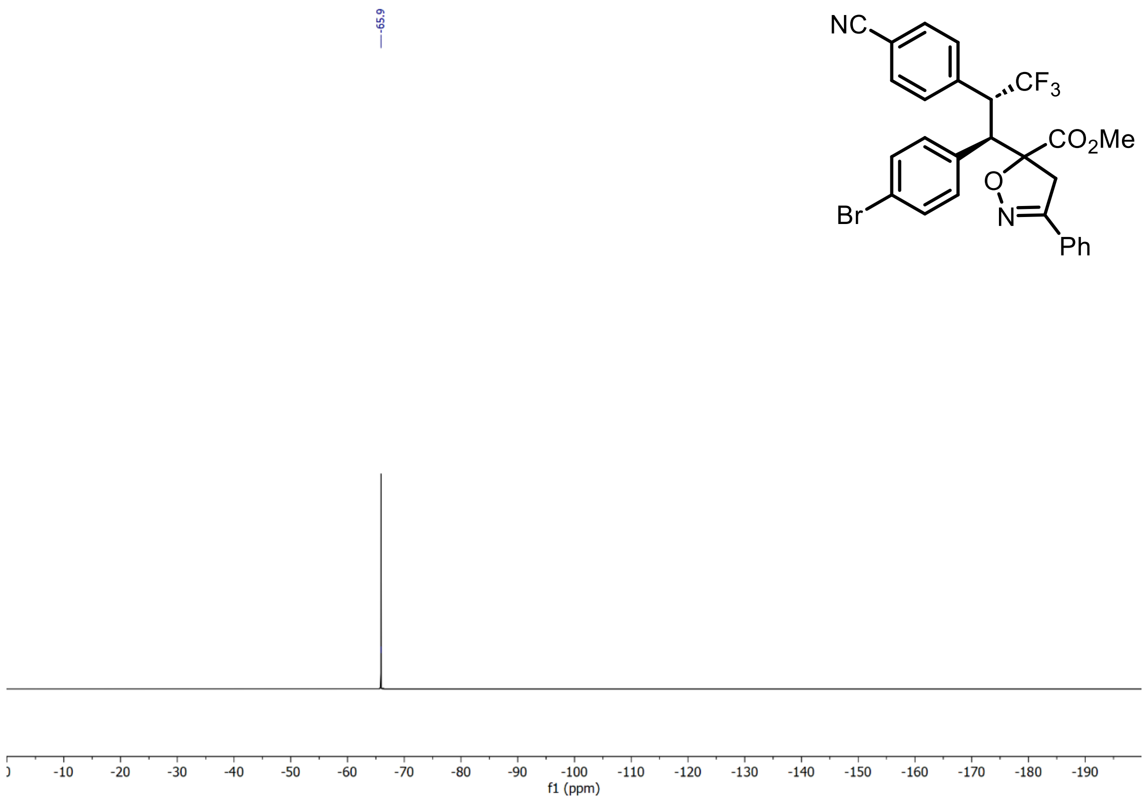

^{19}F NMR (471 MHz, $CDCl_3$) spectra of **S5**.

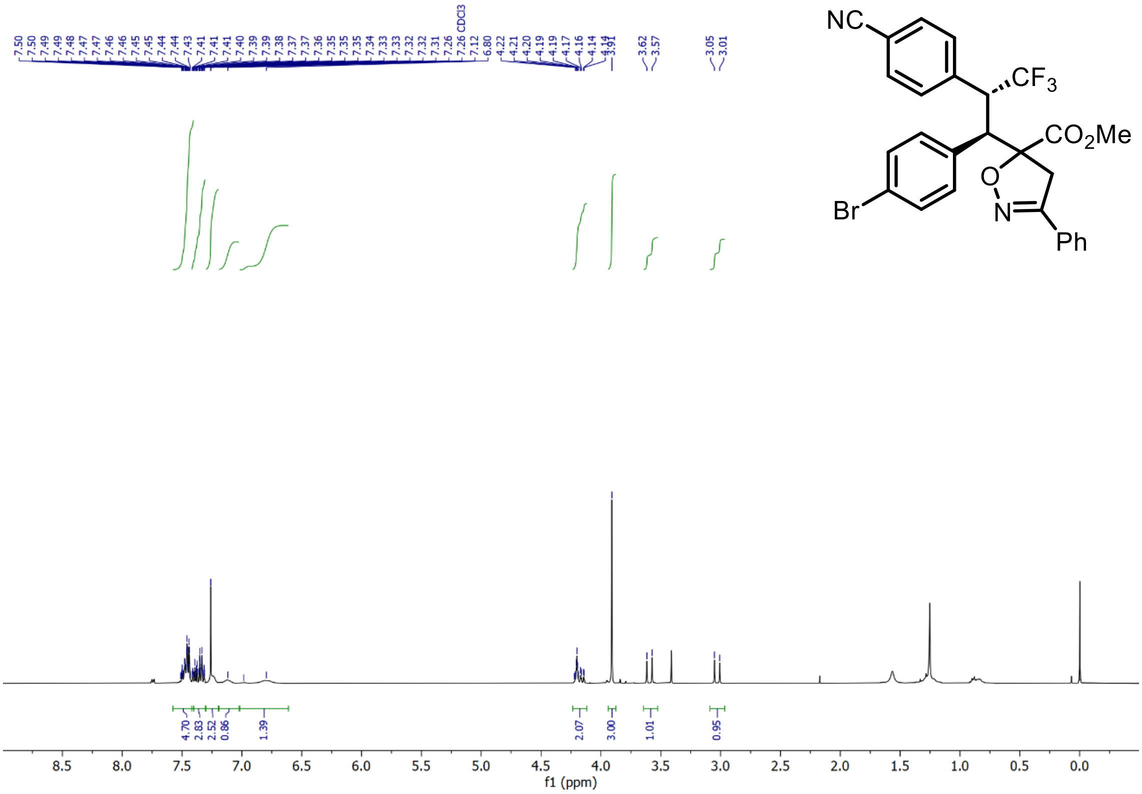

^{1}H NMR (400 MHz, $CDCl_3$) spectra of **S5'**.

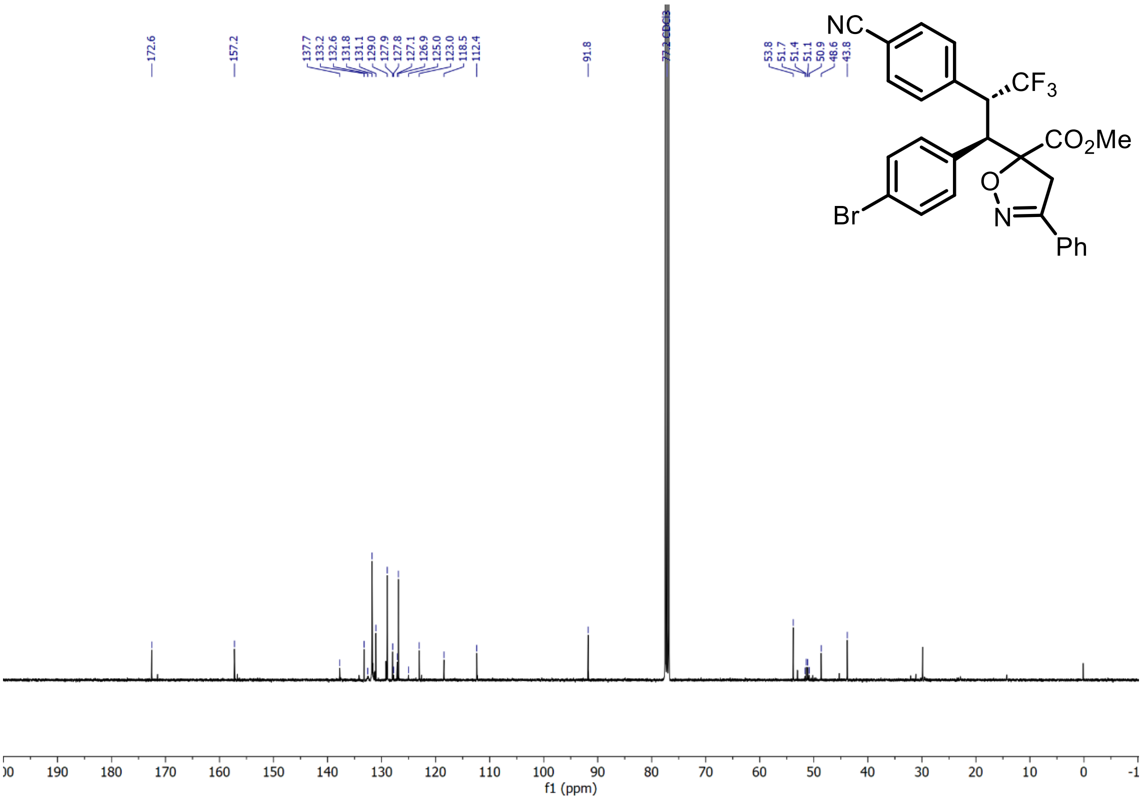

^{13}C NMR (101 MHz, $CDCl_3$) spectra of **S5'**.

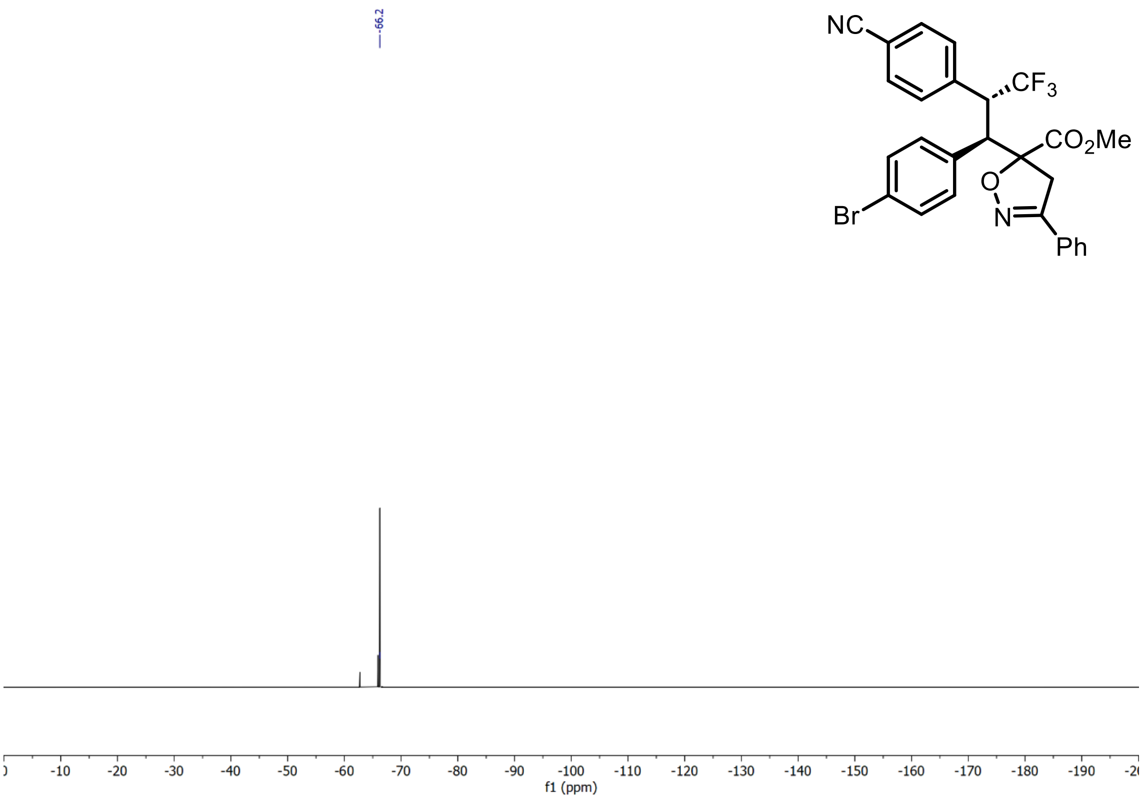

^{19}F NMR (471 MHz, $CDCl_3$) spectra of **S5’**.

N. Chiral HPLC Chromatograms

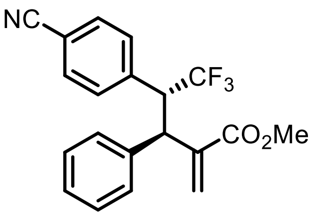

3a

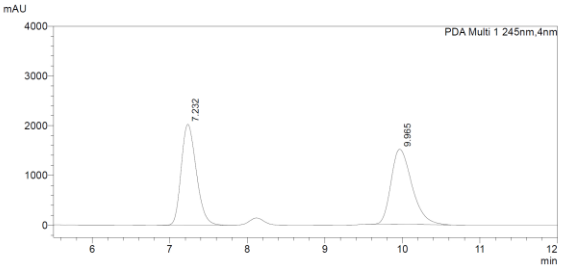

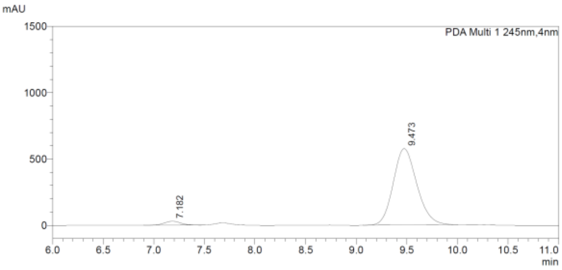

<Peak Table>
PDA Ch1 245nm

Peak#	Ret. Time	Area	Height	Conc.	Unit	Mark	Name
1	7.232	27336564	2027320	49.086			
2	9.965	28354860	1506338	50.914			
Total		55691424	3533658				

<Peak Table>
PDA Ch1 245nm

Peak#	Ret. Time	Area	Height	Conc.	Unit	Mark	Name
1	7.182	341267	30148	3.511			
2	9.473	9378956	576315	96.489			
Total		9720223	606463				

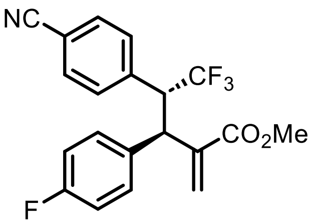

3b

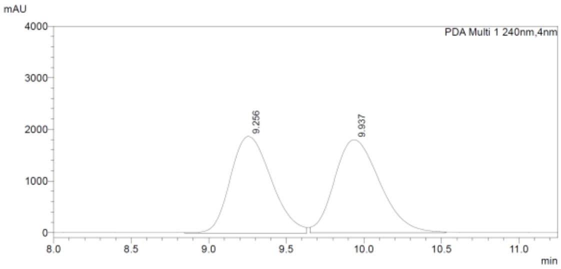

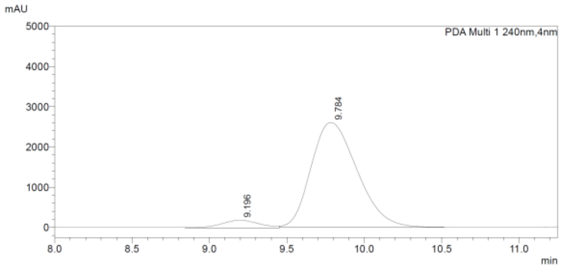

<Peak Table>
PDA Ch1 240nm

Peak#	Ret. Time	Area	Height	Conc.	Unit	Mark	Name
1	9.256	34492844	1872125	48.466			
2	9.937	36675621	1800311	51.534			
Total		71168465	3672436				

<Peak Table>
PDA Ch1 240nm

Peak#	Ret. Time	Area	Height	Conc.	Unit	Mark	Name
1	9.196	3005808	184213	5.239			
2	9.784	54363329	2611926	94.761			
Total		57369137	2796139				

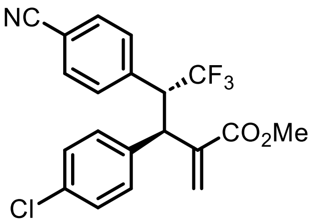

3c

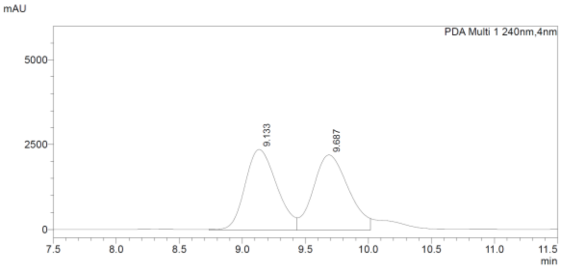

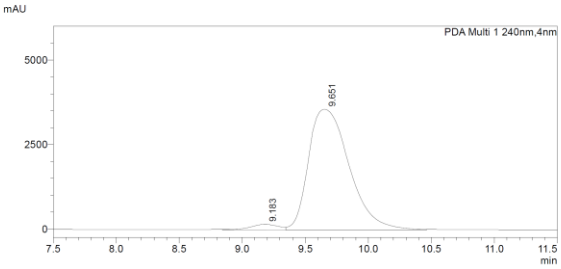

<Peak Table>
PDA Ch1 240nm

Peak#	Ret. Time	Area	Height	Conc.	Unit	Mark	Name
1	9.133	41659610	2377365	49.178			
2	9.687	43053008	2214922	50.822			
Total		84712618	4592287				

<Peak Table>
PDA Ch1 240nm

Peak#	Ret. Time	Area	Height	Conc.	Unit	Mark	Name
1	9.183	2501673	163098	3.034			
2	9.651	79962609	3564657	96.966			
Total		82464282	3727755				

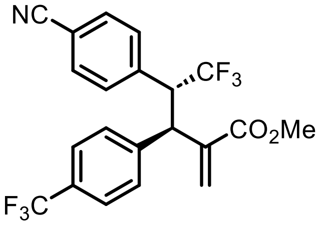

3d

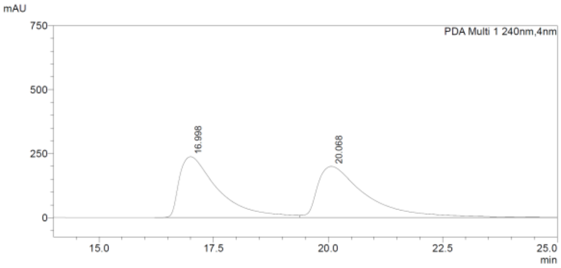

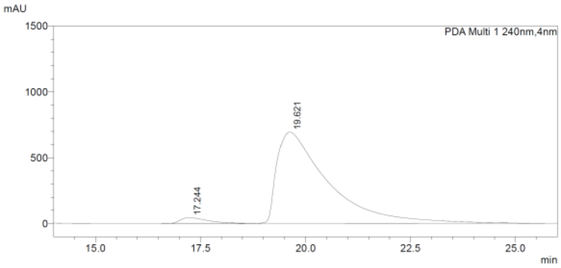

<Peak Table>
PDA Ch1 240nm

Peak#	Ret. Time	Area	Height	Conc.	Unit	Mark	Name
1	16.998	14122114	237339	48.561			
2	20.068	14959163	199471	51.439		SV	
Total		29081277	436809				

<Peak Table>
PDA Ch1 240nm

Peak#	Ret. Time	Area	Height	Conc.	Unit	Mark	Name
1	17.244	2145723	45177	3.498			
2	19.621	59201084	693416	96.502		V	
Total		61346806	738593				

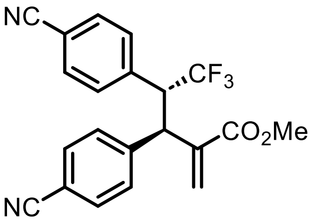

3e

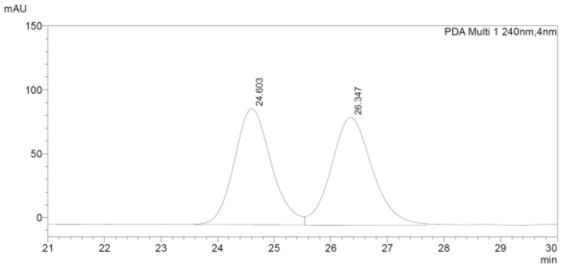

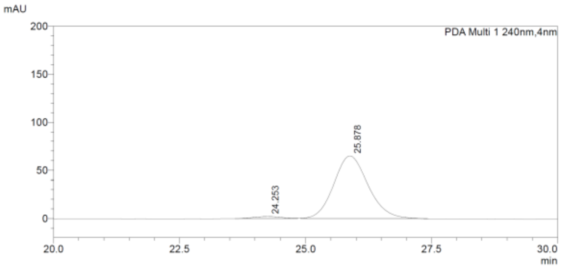

<Peak Table>

PDA Ch1 240nm

Peak#	Ret. Time	Area	Height	Conc.	Unit	Mark	Name
1	24.603	4119122	90897	49.047			
2	26.347	4279133	84504	50.953			
Total		8398254	175400				

<Peak Table>

PDA Ch1 240nm

Peak#	Ret. Time	Area	Height	Conc.	Unit	Mark	Name
1	24.253	90242	2283	2.825			
2	25.878	3104468	65052	97.175			
Total		3194709	67335				

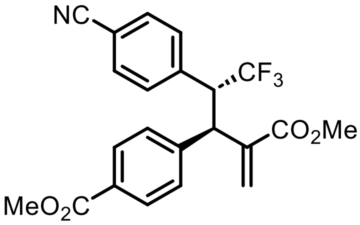

3f

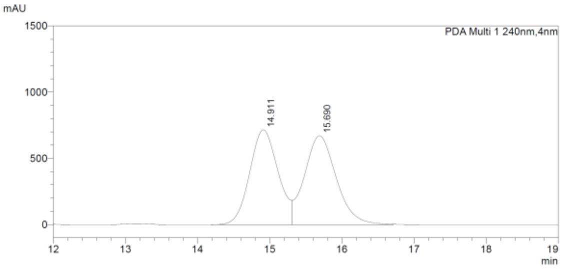

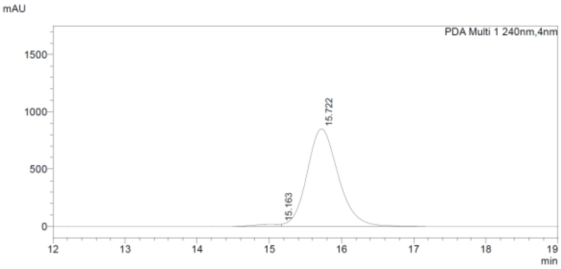

<Peak Table>

PDA Ch1 240nm

Peak#	Ret. Time	Area	Height	Conc.	Unit	Mark	Name
1	14.911	19663448	716739	48.771			
2	15.690	20654710	672047	51.229			
Total		40318158	1388786				

<Peak Table>

PDA Ch1 240nm

Peak#	Ret. Time	Area	Height	Conc.	Unit	Mark	Name
1	15.163	461976	19357	1.770			
2	15.722	25645633	850663	98.230			
Total		26107609	870019				

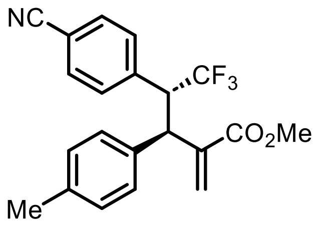

3g

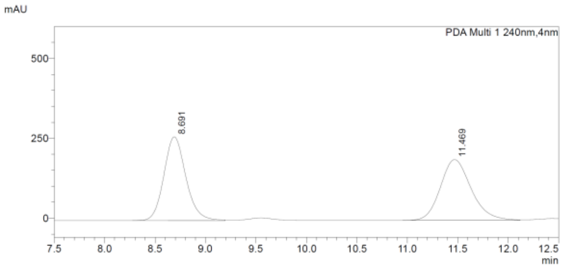

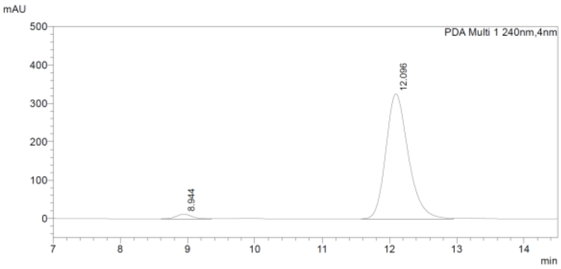

<Peak Table>

PDA Ch1 240nm

Peak#	Ret. Time	Area	Height	Conc.	Unit	Mark	Name
1	8.691	3991738	262497	49.576			
2	11.469	4059958	190471	50.424			
Total		8051696	452968				

<Peak Table>

PDA Ch1 240nm

Peak#	Ret. Time	Area	Height	Conc.	Unit	Mark	Name
1	8.944	187324	12028	2.418			
2	12.096	7561213	325552	97.582			
Total		7748537	337580				

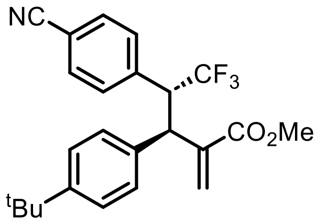

3h

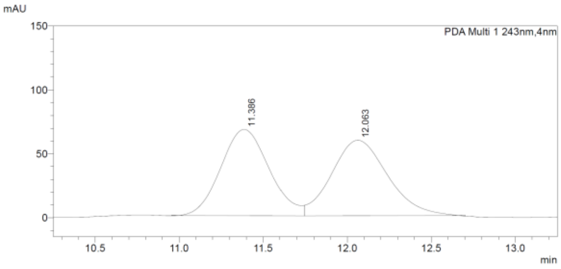

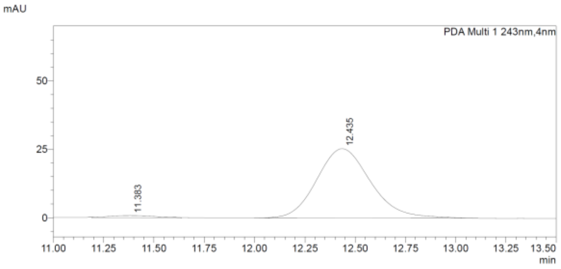

<Peak Table>

PDA Ch1 243nm

Peak#	Ret. Time	Area	Height	Conc.	Unit	Mark	Name
1	11.386	1355871	67509	49.811			
2	12.063	1366141	59105	50.189			
Total		2722011	126613				

<Peak Table>

PDA Ch1 243nm

Peak#	Ret. Time	Area	Height	Conc.	Unit	Mark	Name
1	11.383	13008	808	2.683			
2	12.435	471771	25307	97.317			
Total		484779	26115				

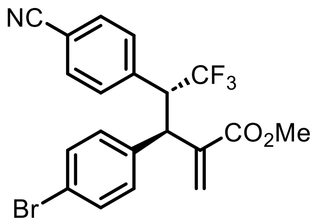

3i

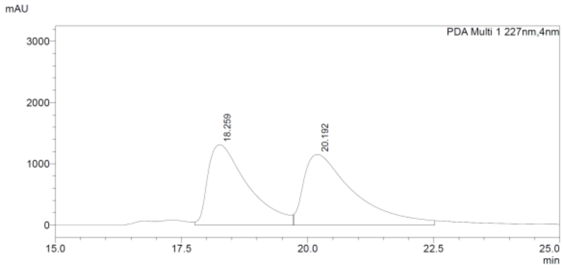

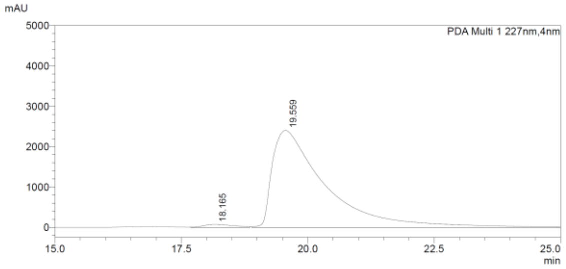

<Peak Table>

PDA Ch1 227nm

Peak#	Ret. Time	Area	Height	Conc.	Unit	Mark	Name
1	18.259	72997927	1316894	47.461			
2	20.192	80808696	1158292	52.539			
Total		153806622	2475186				

<Peak Table>

PDA Ch1 227nm

Peak#	Ret. Time	Area	Height	Conc.	Unit	Mark	Name
1	18.165	3081436	75581	1.709			
2	19.559	177228502	2402774	98.291			
Total		180309938	2478354				

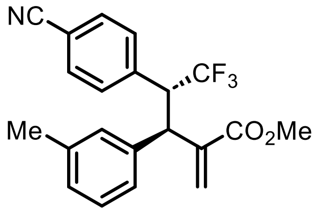

3j

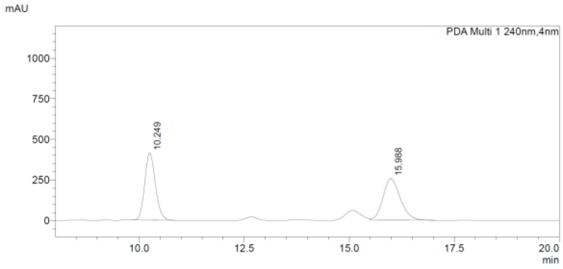

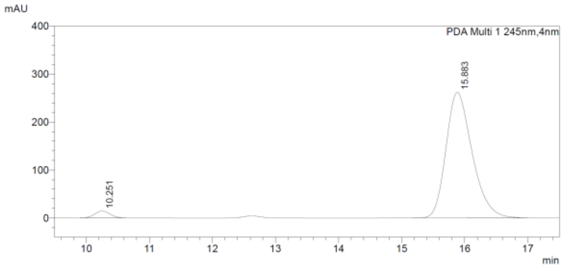

<Peak Table>

PDA Ch1 240nm

Peak#	Ret. Time	Area	Height	Conc.	Unit	Mark	Name
1	10.249	7303886	412879	50.174			
2	15.988	7253291	255254	49.826			
Total		14557177	668133				

<Peak Table>

PDA Ch1 245nm

Peak#	Ret. Time	Area	Height	Conc.	Unit	Mark	Name
1	10.251	248998	14487	3.198			
2	15.883	7537909	261822	96.802			
Total		7786907	276309				

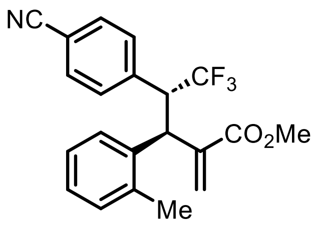

3k

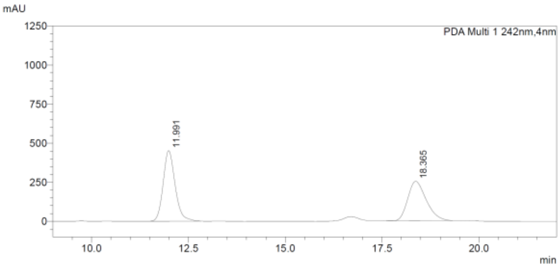

<Peak Table>

PDA Ch1 242nm

Peak#	Ret. Time	Area	Height	Conc.	Unit	Mark	Name
1	11.991	9703550	452424	53.869			
2	18.365	8309643	252323	46.131			
Total		18013193	704747				

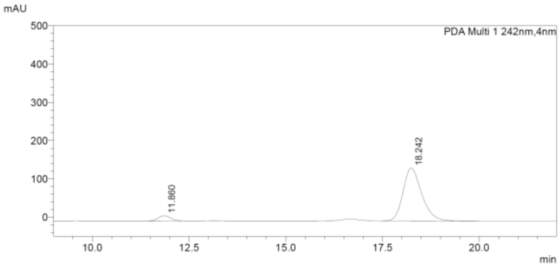

<Peak Table>

PDA Ch1 242nm

Peak#	Ret. Time	Area	Height	Conc.	Unit	Mark	Name
1	11.860	301580	13711	6.138			
2	18.242	4611964	137663	93.862			
Total		4913544	151373				

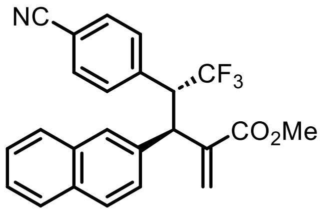

3l

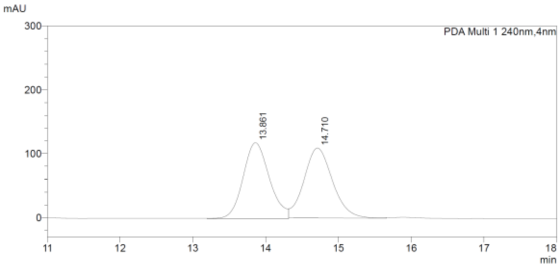

<Peak Table>

PDA Ch1 240nm

Peak#	Ret. Time	Area	Height	Conc.	Unit	Mark	Name
1	13.861	2967080	118244	49.858			
2	14.710	2984000	109242	50.142			
Total		5951080	227485				

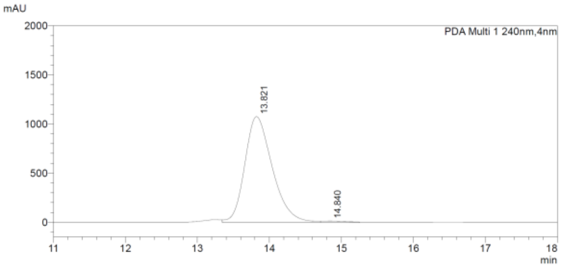

<Peak Table>

PDA Ch1 240nm

Peak#	Ret. Time	Area	Height	Conc.	Unit	Mark	Name
1	13.821	28577118	1076477	99.043			
2	14.840	276037	12571	0.957			
Total		28853154	1089049				

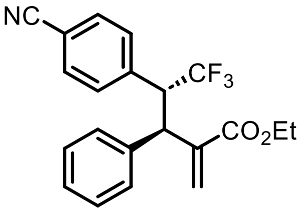

3m

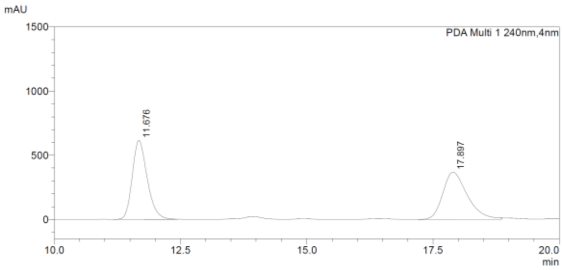

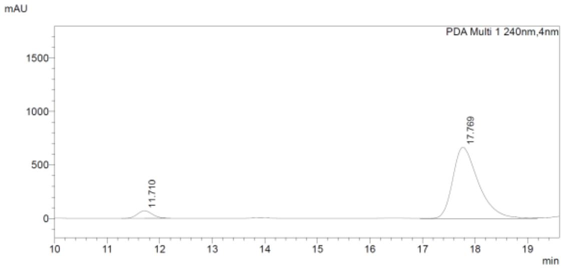

<Peak Table>
PDA Ch1 240nm

Peak#	Ret. Time	Area	Height	Conc.	Unit	Mark	Name
1	11.676	12637442	614176	50.261			
2	17.897	12506433	368716	49.739			
Total		25143875	982893				

<Peak Table>
PDA Ch1 240nm

Peak#	Ret. Time	Area	Height	Conc.	Unit	Mark	Name
1	11.710	1415337	71155	5.873			
2	17.769	22682313	664376	94.127			
Total		24097650	735531				

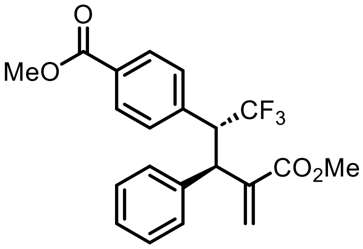

3n

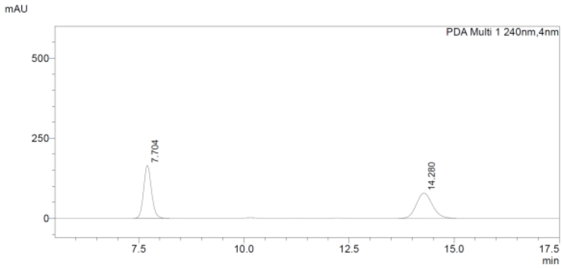

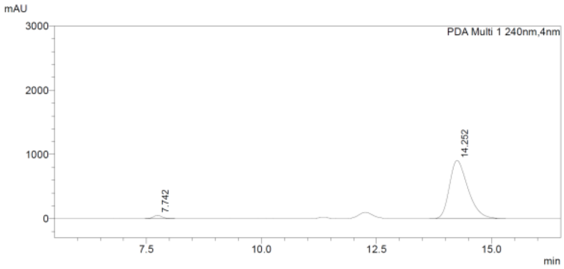

<Peak Table>
PDA Ch1 240nm

Peak#	Ret. Time	Area	Height	Conc.	Unit	Mark	Name
1	7.704	2148807	165047	50.834			
2	14.280	2078267	78857	49.166			
Total		4227074	243903				

<Peak Table>
PDA Ch1 240nm

Peak#	Ret. Time	Area	Height	Conc.	Unit	Mark	Name
1	7.742	665068	51293	2.569			
2	14.252	25219881	905788	97.431			
Total		25884949	957081				

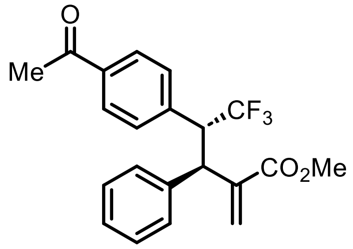

3o

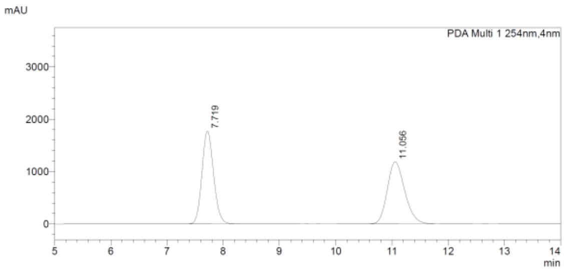

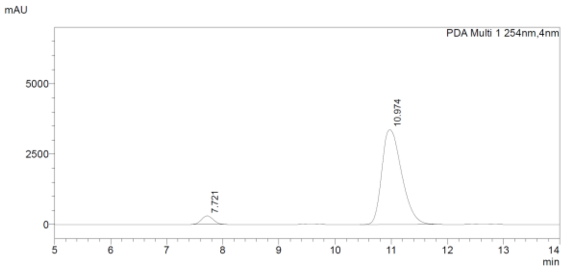

<Peak Table>

PDA Ch1 254nm

Peak#	Ret. Time	Area	Height	Conc.	Unit	Mark	Name
1	7.719	25548653	1771264	49.921			
2	11.056	25629360	1184234	50.079			
Total		51178013	2955499				

<Peak Table>

PDA Ch1 254nm

Peak#	Ret. Time	Area	Height	Conc.	Unit	Mark	Name
1	7.721	4560357	305370	5.203			
2	10.974	83087124	3363169	94.797			
Total		87647481	3668540				

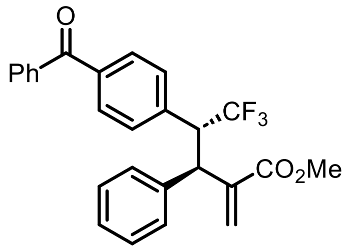

3p

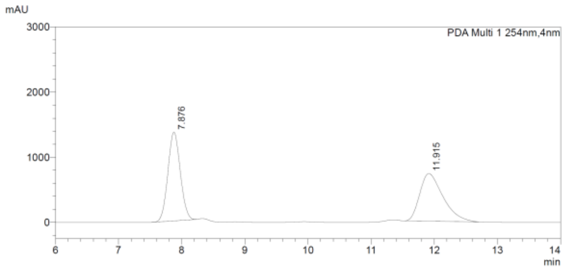

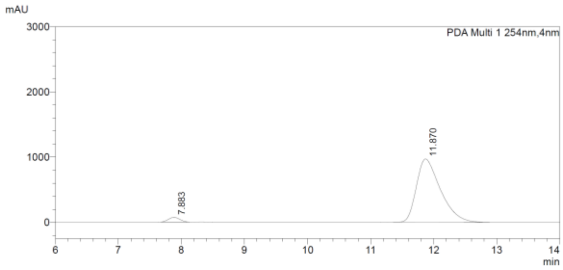

<Peak Table>

PDA Ch1 254nm

Peak#	Ret. Time	Area	Height	Conc.	Unit	Mark	Name
1	7.876	18589271	1361668	49.879			
2	11.915	18679689	731548	50.121			
Total		37268960	2093216				

<Peak Table>

PDA Ch1 254nm

Peak#	Ret. Time	Area	Height	Conc.	Unit	Mark	Name
1	7.883	914169	71840	3.447			
2	11.870	25606652	967034	96.553			
Total		26520821	1038874				

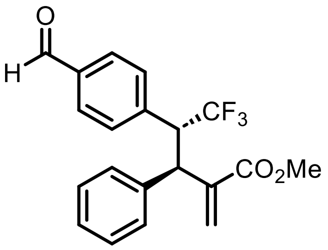

3q

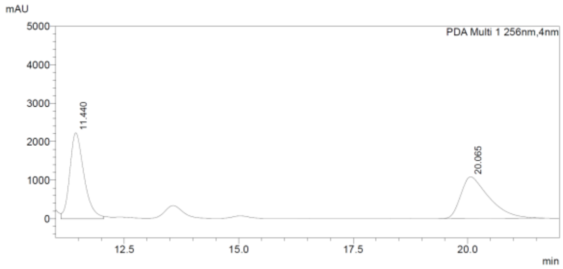

<Peak Table>

PDA Ch1 256nm

Peak#	Ret. Time	Area	Height	Conc.	Unit	Mark	Name
1	11.440	48372135	2230991	51.037			
2	20.065	46406150	1074833	48.963			
Total		94778285	3305824				

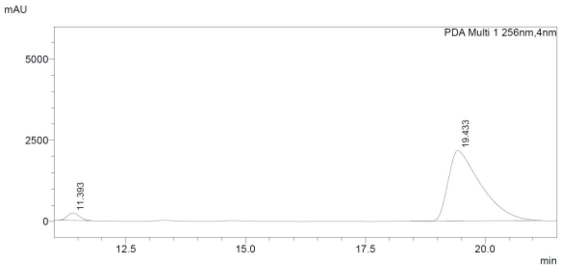

<Peak Table>

PDA Ch1 256nm

Peak#	Ret. Time	Area	Height	Conc.	Unit	Mark	Name
1	11.393	4077153	222975	3.824			
2	19.433	102546855	2160548	96.176			
Total		106624008	2383523				

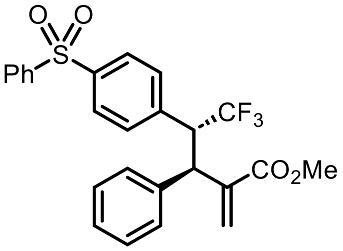

3r

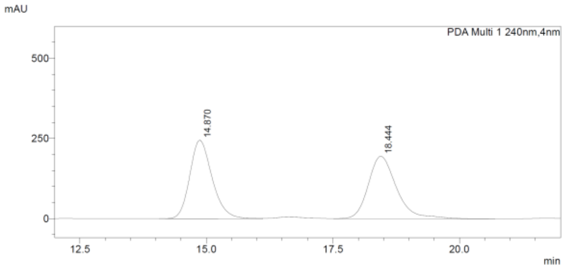

<Peak Table>

PDA Ch1 240nm

Peak#	Ret. Time	Area	Height	Conc.	Unit	Mark	Name
1	14.870	7557596	244650	49.866			
2	18.444	7598163	194984	50.134			
Total		15155759	439634				

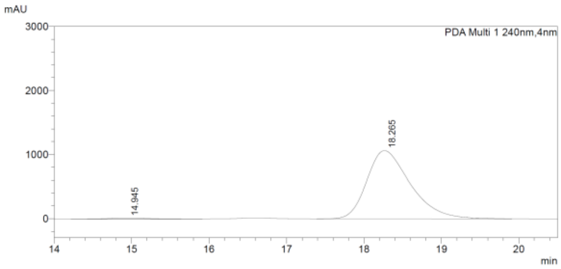

<Peak Table>

PDA Ch1 240nm

Peak#	Ret. Time	Area	Height	Conc.	Unit	Mark	Name
1	14.945	422344	11586	1.004			
2	18.265	41634347	1062448	98.996			
Total		42056691	1074035				

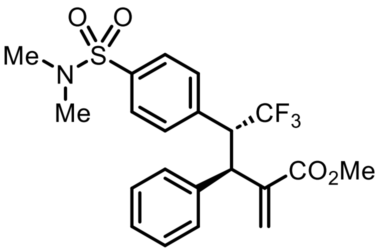

3s

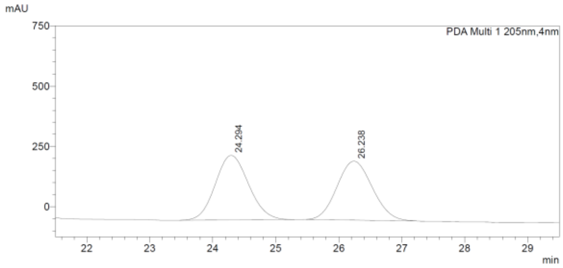

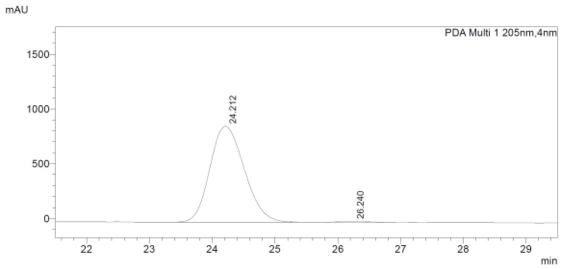

<Peak Table>

PDA Ch1 205nm

Peak#	Ret. Time	Area	Height	Conc.	Unit	Mark	Name
1	24.294	9857858	267570	50.873			
2	26.238	9519700	244151	49.127			
Total		19377558	511721				

<Peak Table>

PDA Ch1 205nm

Peak#	Ret. Time	Area	Height	Conc.	Unit	Mark	Name
1	24.212	33362821	874142	99.418			
2	26.240	195379	5758	0.582			
Total		33558200	879899				

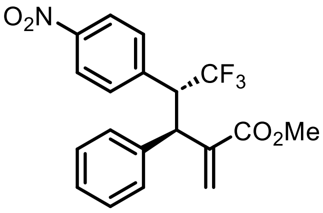

3t

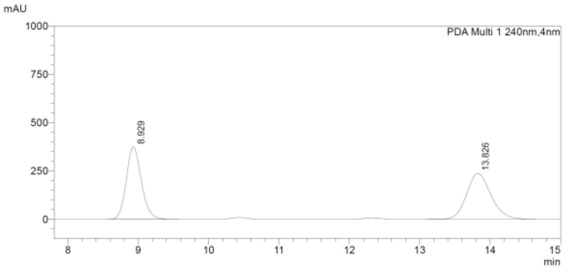

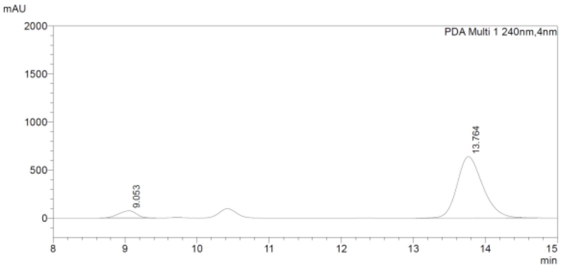

<Peak Table>

PDA Ch1 240nm

Peak#	Ret. Time	Area	Height	Conc.	Unit	Mark	Name
1	8.929	5571730	374328	49.338			
2	13.826	5721344	236250	50.662			
Total		11293074	610579				

<Peak Table>

PDA Ch1 240nm

Peak#	Ret. Time	Area	Height	Conc.	Unit	Mark	Name
1	9.053	1317493	76202	7.671			
2	13.764	15857046	638677	92.329			
Total		17174540	714879				

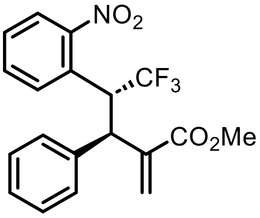

3u

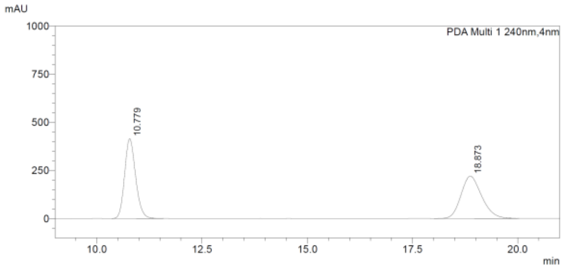

<Peak Table>
PDA Ch1 240nm

Peak#	Ret. Time	Area	Height	Conc.	Unit	Mark	Name
1	10.779	7544218	413238	50.123			
2	18.873	7507126	218651	49.877			
Total		15051344	631889				

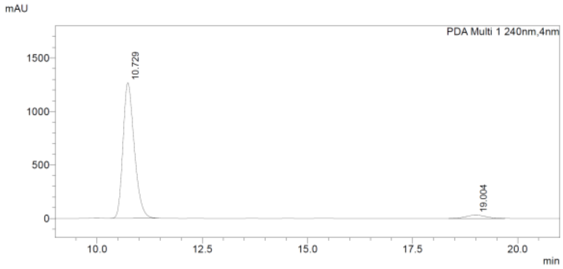

<Peak Table>
PDA Ch1 240nm

Peak#	Ret. Time	Area	Height	Conc.	Unit	Mark	Name
1	10.729	23790249	1268148	95.868			
2	19.004	1025259	31093	4.132			
Total		24815507	1299241				

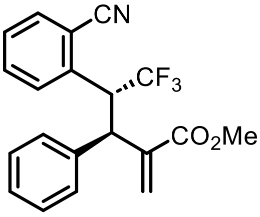

3v

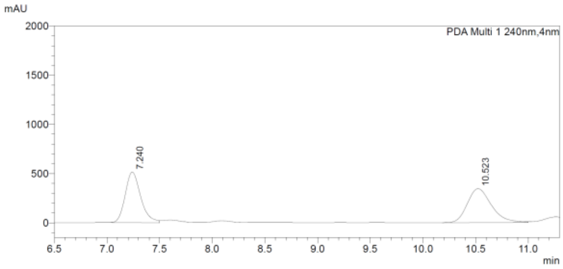

<Peak Table>
PDA Ch1 240nm

Peak#	Ret. Time	Area	Height	Conc.	Unit	Mark	Name
1	7.240	5310232	515794	48.985			
2	10.523	5530251	345131	51.015			
Total		10840483	860925				

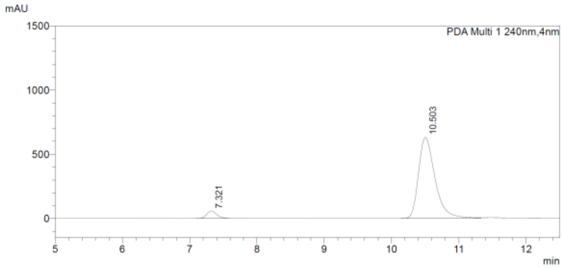

<Peak Table>
PDA Ch1 240nm

Peak#	Ret. Time	Area	Height	Conc.	Unit	Mark	Name
1	7.321	602345	57763	5.287			
2	10.503	10790330	631829	94.713			
Total		11392676	689592				

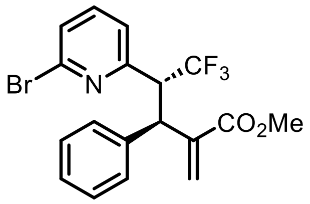

3w

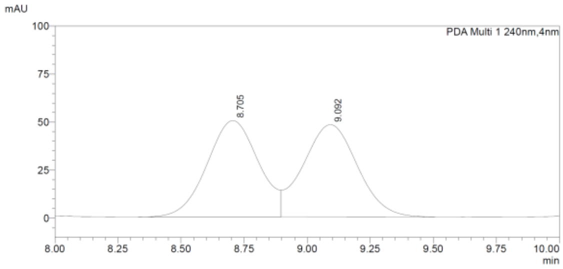

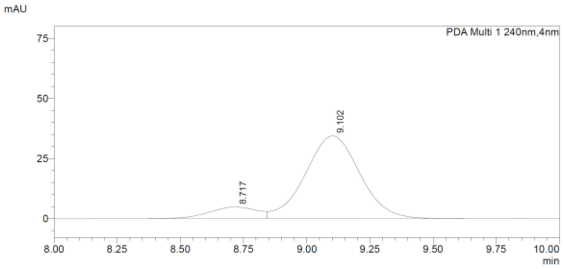

<Peak Table>
PDA Ch1 240nm

Peak#	Ret. Time	Area	Height	Conc.	Unit	Mark	Name
1	8.705	718871	50241	50.045			
2	9.092	717564	48032	49.955			
Total		1436435	98273				

<Peak Table>
PDA Ch1 240nm

Peak#	Ret. Time	Area	Height	Conc.	Unit	Mark	Name
1	8.717	64227	4809	11.078			
2	9.102	515544	34413	88.922			
Total		579770	39222				

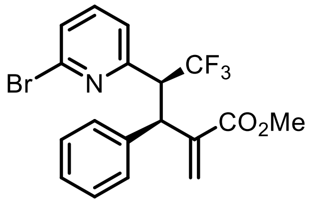

3w'

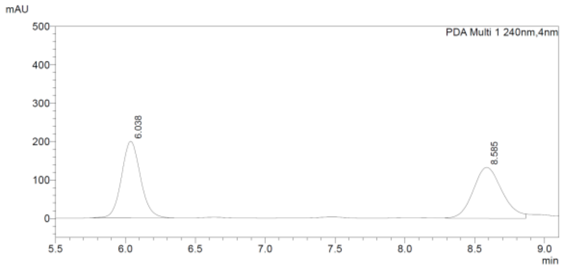

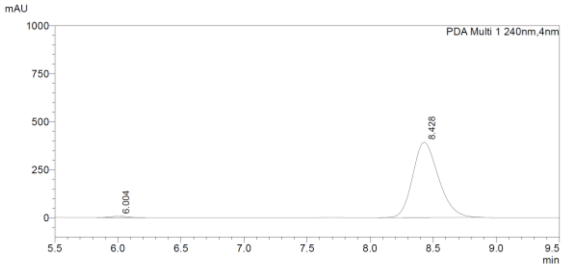

<Peak Table>
PDA Ch1 240nm

Peak#	Ret. Time	Area	Height	Conc.	Unit	Mark	Name
1	6.038	1888572	199682	49.699			
2	8.585	1911434	132364	50.301			
Total		3800006	332046				

<Peak Table>
PDA Ch1 240nm

Peak#	Ret. Time	Area	Height	Conc.	Unit	Mark	Name
1	6.004	68115	7381	1.203			
2	8.428	5592344	392780	98.797			
Total		5660459	400160				

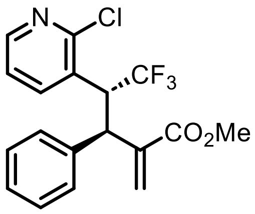

3x

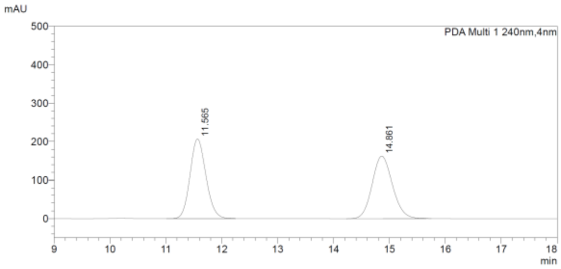

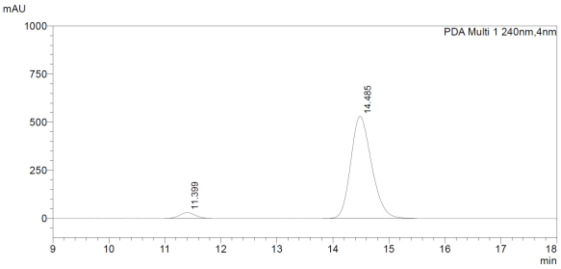

<Peak Table>
PDA Ch1 240nm

Peak#	Ret. Time	Area	Height	Conc.	Unit	Mark	Name
1	11.565	4095950	206989	49.923			
2	14.861	4108507	162385	50.077			
Total		8204457	369375				

<Peak Table>
PDA Ch1 240nm

Peak#	Ret. Time	Area	Height	Conc.	Unit	Mark	Name
1	11.399	568087	29582	3.976			
2	14.485	13718988	530888	96.024			
Total		14287075	560469				

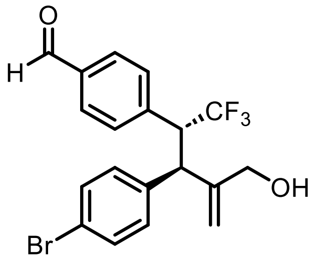

S4

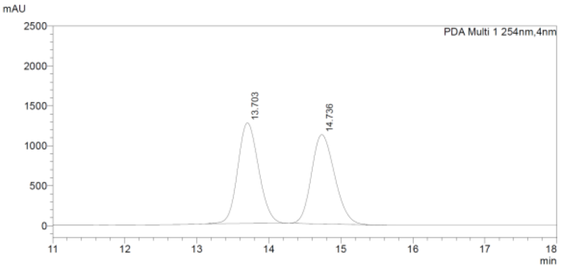

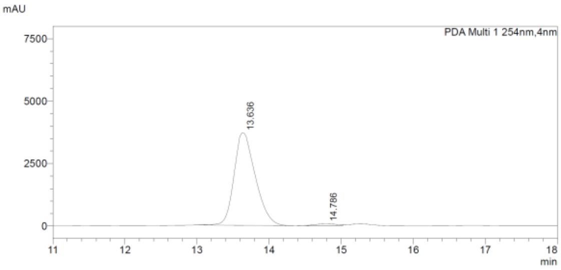

<Peak Table>
PDA Ch1 254nm

Peak#	Ret. Time	Area	Height	Conc.	Unit	Mark	Name
1	13.703	25336572	1256967	50.349			
2	14.736	24985629	1114881	49.651			
Total		50322201	2371848				

<Peak Table>
PDA Ch1 254nm

Peak#	Ret. Time	Area	Height	Conc.	Unit	Mark	Name
1	13.636	75901498	3700841	97.779			
2	14.786	1724241	79297	2.221			
Total		77625739	3780138				

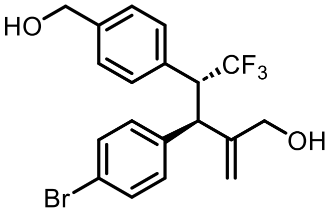

6

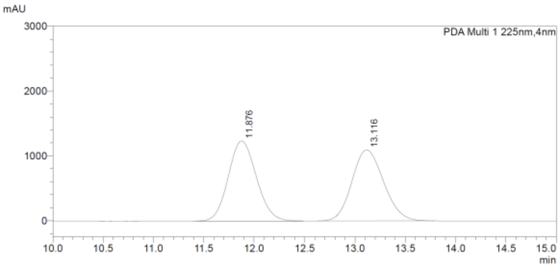

<Peak Table>

PDA Ch1 225nm

Peak#	Ret. Time	Area	Height	Conc.	Unit	Mark	Name
1	11.876	23913824	1231176	49.969			
2	13.116	23943929	1091326	50.031			
Total		47857753	2322502				

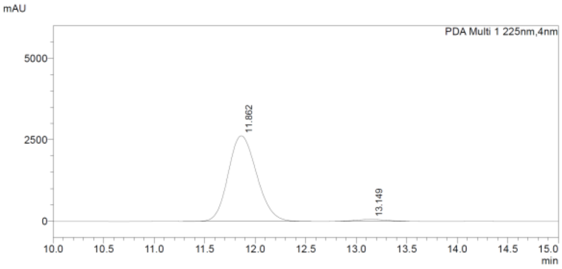

<Peak Table>

PDA Ch1 225nm

Peak#	Ret. Time	Area	Height	Conc.	Unit	Mark	Name
1	11.862	51261047	2619038	97.853			
2	13.149	1124937	55144	2.147			
Total		52385984	2674183				

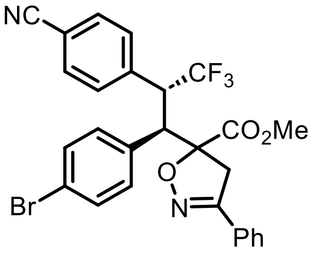

S5

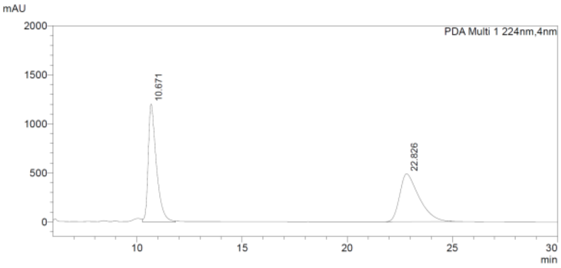

<Peak Table>

PDA Ch1 224nm

Peak#	Ret. Time	Area	Height	Conc.	Unit	Mark	Name
1	10.671	32529574	1202526	50.994			
2	22.826	31261847	488244	49.006			
Total		63791421	1690769				

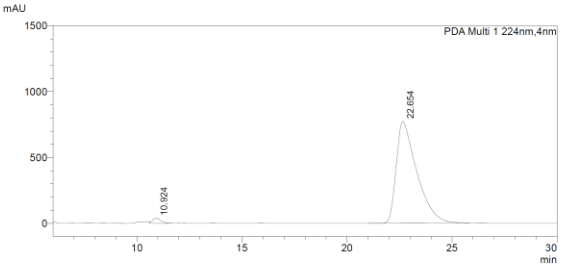

<Peak Table>

PDA Ch1 224nm

Peak#	Ret. Time	Area	Height	Conc.	Unit	Mark	Name
1	10.924	1116661	40353	2.082			
2	22.654	52524271	772655	97.918			
Total		53640932	813008				

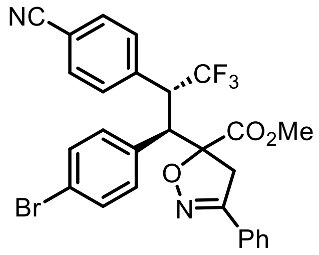

S5'

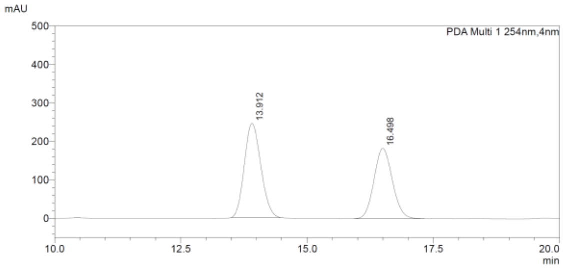

<Peak Table>

PDA Ch1 254nm

Peak#	Ret. Time	Area	Height	Conc.	Unit	Mark	Name
1	13.912	5563174	244808	54.682			
2	16.498	4610433	182293	45.318			
Total		10173607	427101				

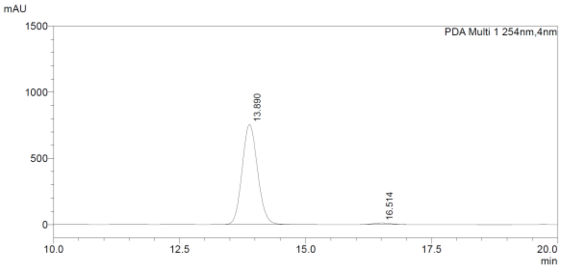

<Peak Table>

PDA Ch1 254nm

Peak#	Ret. Time	Area	Height	Conc.	Unit	Mark	Name
1	13.890	16020780	753917	98.205			
2	16.514	292799	12142	1.795			
Total		16313579	766059				
